# Absence of Intermediates in the BINOL-Derived Mg(II)/Phosphate-Catalyzed Desymmetrizative Ring Expansion of 1-Vinylcyclobutanols

**DOI:** 10.1021/acs.joc.1c02699

**Published:** 2021-12-20

**Authors:** Estefania Capel, Marta Rodríguez-Rodríguez, Uxue Uria, Manuel Pedron, Tomas Tejero, Jose L. Vicario, Pedro Merino

**Affiliations:** † University of the Basque Country (UPV/EHU), P.O. Box 644, 48080 Bilbao, Spain; ‡ Instituto de Biocomputación y Física de Sistemas Complejos (BIFI), Universidad de Zaragoza, 50009 Zaragoza, Spain; § Instituto de Sintesis y Catalisis Homogenea (ISQCH), Universidad de Zaragoza-CSIC, 50009 Zaragoza, Spain

## Abstract

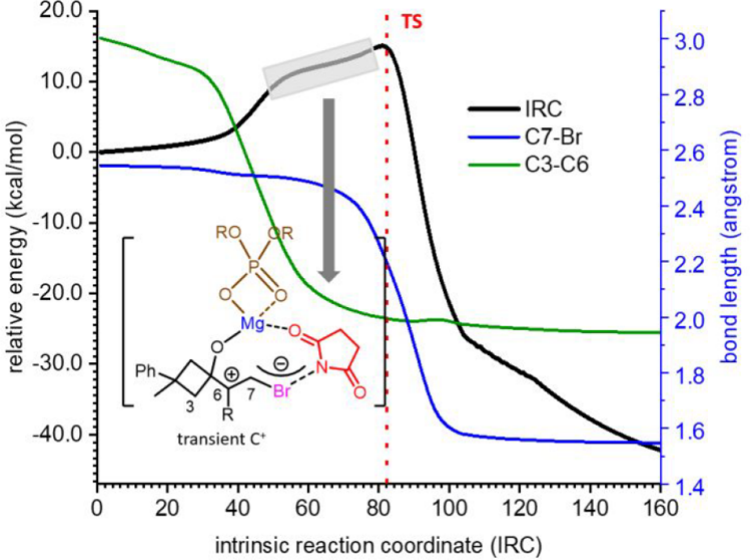

The catalyzed desymmetrizative ring expansion of alkenylcyclobutanols promoted by halofunctionalization of the alkene moiety with *N*-bromosuccinimide has been experimentally and computationally studied. The reaction yields highly enantioenriched cyclopentanones bearing two all-carbon quaternary stereocenters, one of them being generated in the rearrangement of the cyclobutane ring and the other by enantioselective desymmetrization. The reaction is competitive with the formation of a spiroepoxide, but it turns completely selective toward the cyclopentanone when a chiral bisphosphonium magnesium salt is employed as a catalyst. Mechanistic studies support the formation of an ion pair leading to a complex with only a unit of phosphoric acid, which is the resting state of the catalytic cycle. Calculations reproduce in an excellent way the observed reactivity and predict the effect exerted by the substituents of the aromatic ring linked to the double bond. The computational studies also revealed the reaction as a highly asynchronous concerted process taking place as one kinetic step but in two stages: (i) halogenation of the double bond and (ii) rearrangement of the cyclobutane. No intermediates are present in the reaction as energy minima. The experimental scope of the reaction further confirms the predictions for the observed reactivity and selectivity.

## Introduction

**Scheme 1:**
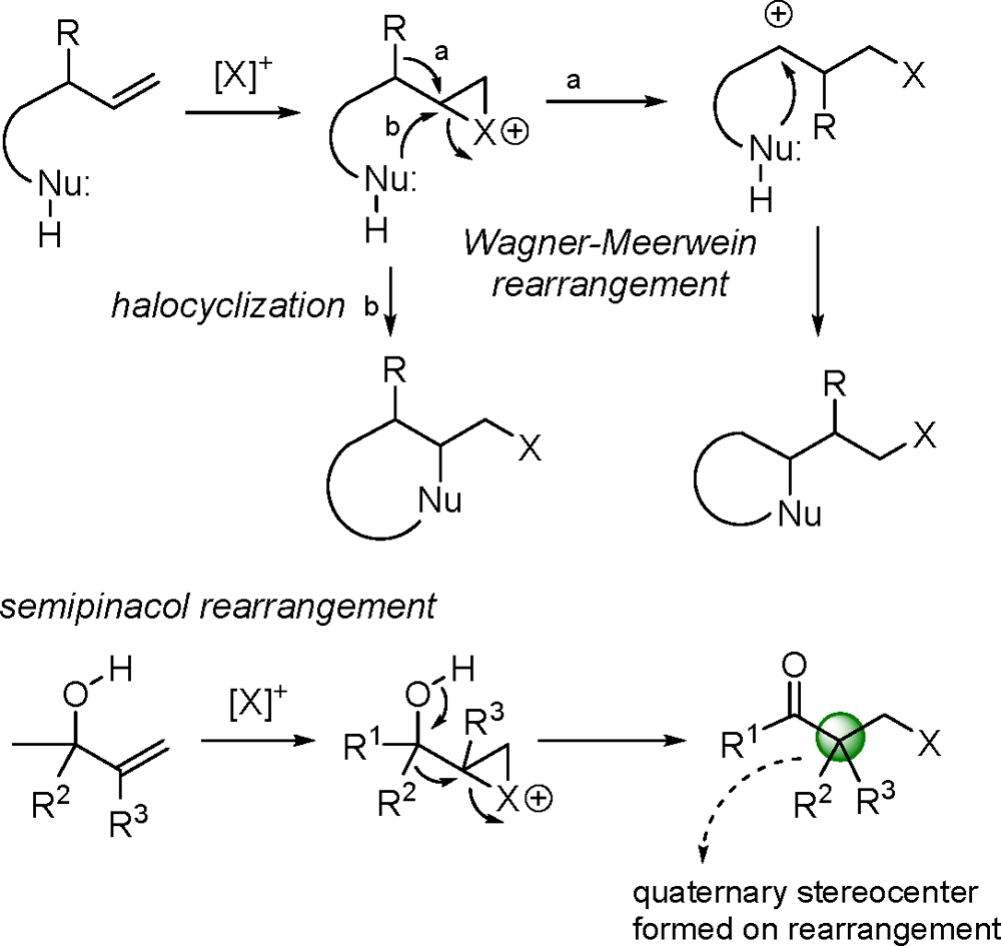
Halofunctionalizations, Semipinacol Rearrangement, and Application to Enantioselective Desymmetrization of Alkenyl Cyclobutanols

The enantioselective halofunctionalization of alkenes has led to the discovery of a vast array of different reactions of great synthetic utility.^1^ In most of these processes, the halogenation initiates a Wagner–Meerwein rearrangement followed by the capture of the intermediate carbocation/haliranium ion by a nucleophile.^2^ The intramolecular version of halofunctionalization (halocyclization),^3^ including halolactonization,^4^ has been extensively studied. Of particular interest are the reactions in which the electron-deficient carbon (not necessarily a carbocation) is vicinal to an oxygen-containing carbon (the case of allylic alcohols).^5^ In this case, the 1,2 migration of a carbon–carbon bond generates a carbonyl group consisting of a semipinacol rearrangement.^6^ When the electron-deficient carbon generated during the halogenation step becomes tertiary, thus better supporting the positive charge, the resulting product is a carbonyl compound with a quaternary center in the α position (Scheme 1; R^2^ and R^3^ ≠ H).^7^

In this respect, the stereoselective construction of carbon atoms with four C-bonded substituents remains a current scientific challenge for synthetic chemists, despite the intensive activity in the field in recent years.^8^ While research in this area has been directed mainly toward the stereoselective formation of one of the C–C bonds that forms the quaternary stereocenter, the possibility of performing the desymmetrization of an achiral compound that already contains the quaternary carbon atom also shows up as an appealing alternative.^9^

**Scheme 2:**
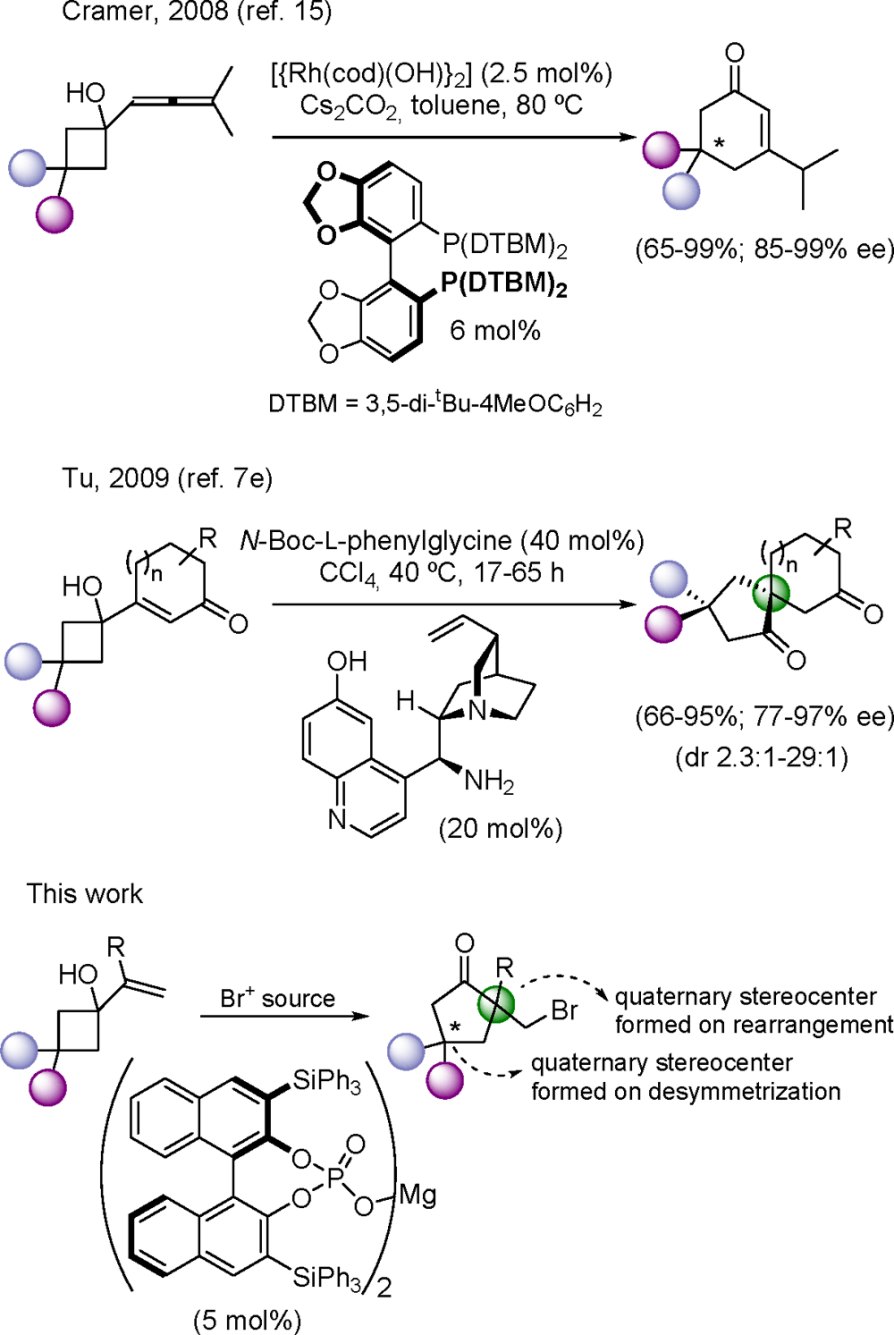
Catalytic Asymmetric Desymmetrizative Semipinacol Rearrangements from Cyclobutanols

This approach leads to easier formation of the starting material through standard methodologies in comparison with the challenging nature of the enantioselective bond formation process when a quaternary stereogenic center is generated. In this context, the semipinacol rearrangement becomes an excellent approach because an additional quaternary center can be formed during the rearrangement. In particular, we have directed our interest to the semipinacol rearrangement of alkenyl cyclobutanol derivatives,^6,10^ for which there are also several enantioselective versions.^6,11^ The ring expansion of cyclobutane-containing symmetric substrates leads to cyclopentane scaffolds containing carbon quaternary stereocenters^12^ of interest due to the presence of this motif in a variety of natural products and active pharmaceutical ingredients.^13^ Although some examples of this reaction involving desymmetrization have already been described,^11a,14^ only two reports have made use of 3,3-disubstituted cyclobutan-1-ols as starting materials that lead to the formation of cyclopentanone adducts with an additional quaternary stereocenter arising as a consequence of the desymmetrization process (Scheme 2). One of these examples converts allenylcyclobutanols into cyclohexenones under Rh catalysis,^15^ and the second makes use of iminium catalysis to activate α-(1-hydroxycyclobut-1-yl)-substituted enones to form spiro[4.5]decane-1,7-dione derivatives.^7e^

With these precedents in mind, we envisaged the possibility of using 3,3-disubstituted-1-alkenylcyclobutanols as suitable substrates to perform catalytic enantioselective semipinacol rearrangement initiated by the electrophilic bromination of the alkene moiety (Scheme 2).^16^ This reaction provides 2,2,4,4-tetrasubstituted cyclopentanones with two all-carbon quaternary stereocenters, as a result of both the new stereocenter formed across the rearrangement process and the newly developed one resulting from the desymmetrization of the starting material. Also, different mechanisms can operate depending on the reaction and catalyst type, so the range of possible mechanisms is still an open question.

The reaction mechanism of the semipinacol rearrangement of alkenyl cyclobutanols to give cyclopentanones promoted by halogenation of the double bond has been studied by Alexakis and co-workers, who suggested, on the basis of kinetic measurements, the formation of an intermediate carbocation in the fluorination-driven rearrangement and the presence of a bromiranium intermediate in bromination-driven reactions.^14b^ Other related mechanistic studies include the enantioselective rearrangement of alkenyl cyclobutanols promoted by sulfenylation of the double bond that have been computationally studied by Bao, Tu, Chen, and co-workers, suggesting the formation of a thiiranium intermediate.^16a^ These authors have also carried out DFT calculations for tandem Nazarov cyclizations/semipinacol rearrangement postulating the formation of carbocationic intermediate species.^17^ On the contrary, Zhou, Shao, and co-workers carried out computational studies for the Lewis base-catalyzed semipinacol rearrangement of hydroxycyclobutyl enones, suggesting the absence of intermediates in the rearrangement step.^18^ No further computational mechanistic studies of the halogen-promoted semipinacol rearrangement have been reported to discuss the nature of intermediates.

**Figure 1:**
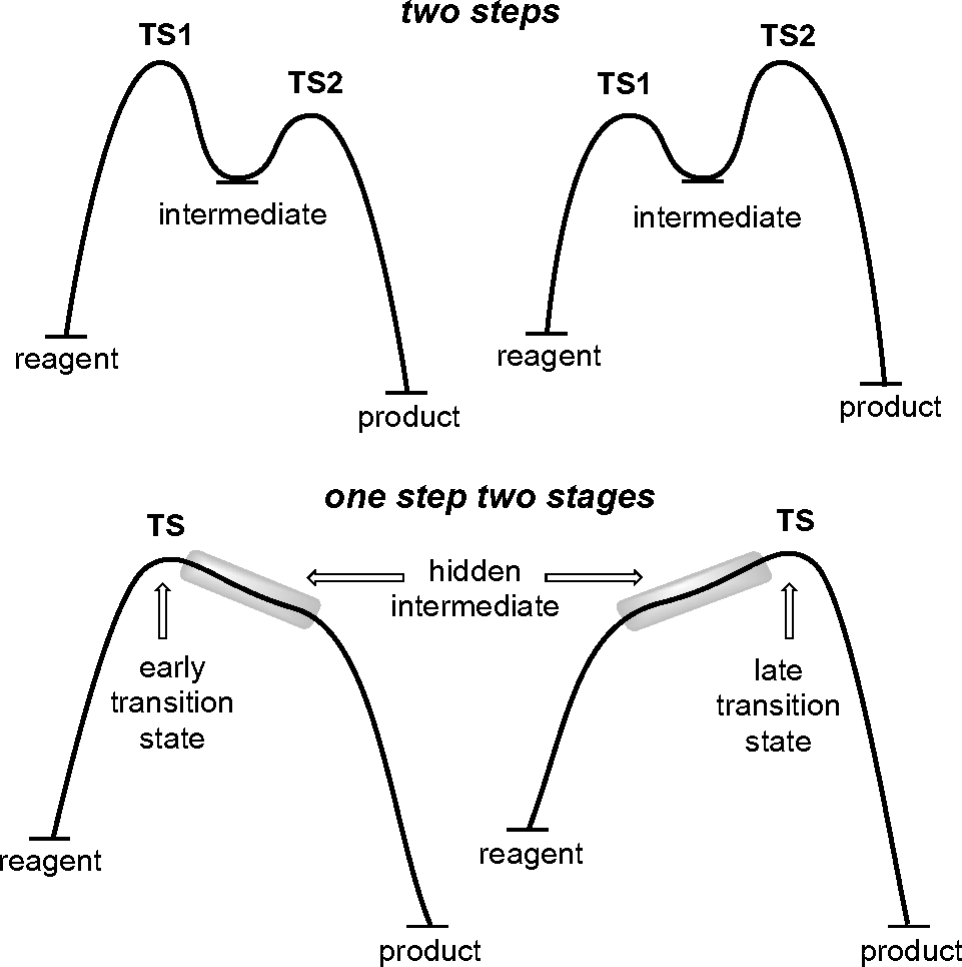
Typical reaction coordinates for two-step reactions with the first step as the rate-determining step (left) or the second (right) (top). Reaction coordinates for highly asynchronous concerted reactions taking place in one kinetic step but with two or more events featuring early (left) or late (right) transition states and the possibility of a hidden intermediate (bottom).

In principle, the reaction is expected to proceed via a haliranium intermediate (or a highly stabilized β-halocarbenium ion in agreement with previous studies^19^) and thus can adopt a profile in which the rate-limiting step is either the first (Figure 1, top left) or the second (Figure 1, top right). The formation of haliranium intermediates has been studied in the past^20^ to rationalize the stereochemical and structure–reactivity characteristics of the halogenation of the double bonds,^21^ and it has been determined that the formation of the haliranium ion is reversible.^22^ However, in some instances, the intermediate is not revealed as a minimum but as a transient species, so-called hidden intermediates,^23^ which can have physical implications that can be observed experimentally, as we recently demonstrated.^24^ In such cases, only one transition state exists and the reaction can be considered as a concerted but very asynchronous process, actually a one-step, two-stage process,^25^ presenting either an early or a late transition state (Figure 1, bottom). The high asynchronicity of these processes is revealed by reaction coordinates with low slope areas and/or shoulders. In these cases, the reaction mechanism is better studied in terms of reaction phases^23^ for which the topological ELF^26^ analysis^27^ is a very useful and illustrative tool (see below).

In this work, we report the catalytic asymmetric semipinacol rearrangement of 1-vinylcyclobutanols, initiated by NBS as a bromine source and catalyzed by a BINOL-derived Mg(II) phosphate, to provide 2,2,4,4-tetrasubstituted cyclopentanones. We also study quantum mechanically the process by using conventional techniques as well as topological approaches (ELF^27^ and NCI^28^) and quasi-classical direct dynamic calculations.^29^

## Results and Discussion

**Scheme 3:**
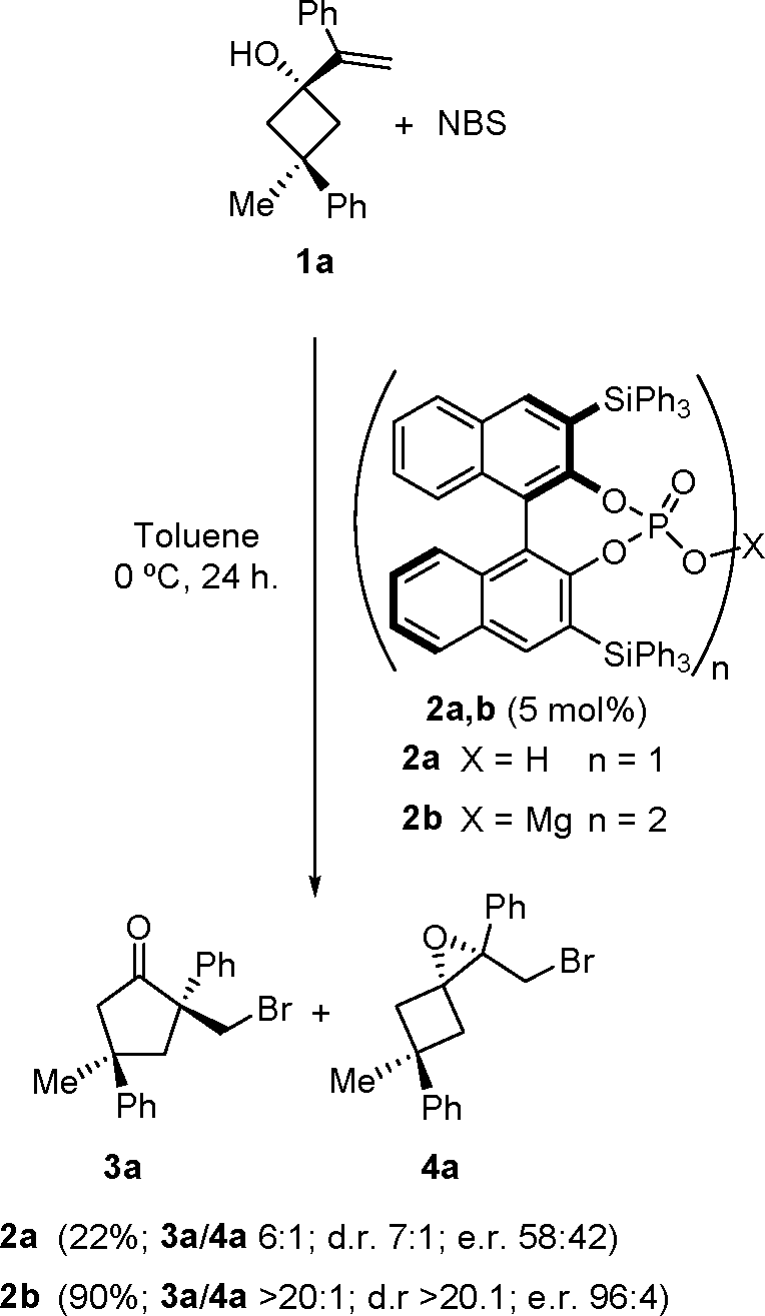
Enantioselective Desymmetrization of Alkenylcyclobutanol **1a**

We started our work by searching the appropriate conditions for the reaction employing cyclobutanol **1a** as a model substrate and using *N*-bromosuccinimide (NBS) to trigger the semipinacol rearrangement (Scheme 3).

In a first approach, BINOL-based chiral phosphoric acids, such as **2a**, were surveyed as catalysts for this transformation, due to their reported proficiency in promoting other semipinacol rearrangements that do not involve desymmetrization.^16a,30^ However, after a variety of these typee of catalysts have been tested (Table 1), none of them provided good results, typically leading to a 6:1 mixture of the expected **3a** and **4a**, a side product that results from the intramolecular attack of the alcohol on the brominated intermediate, in low yield (22%), moderate diastereoselectivity (dr 7:1), and low enantioselectivity (er 58:42).^31^

**Table 1:**
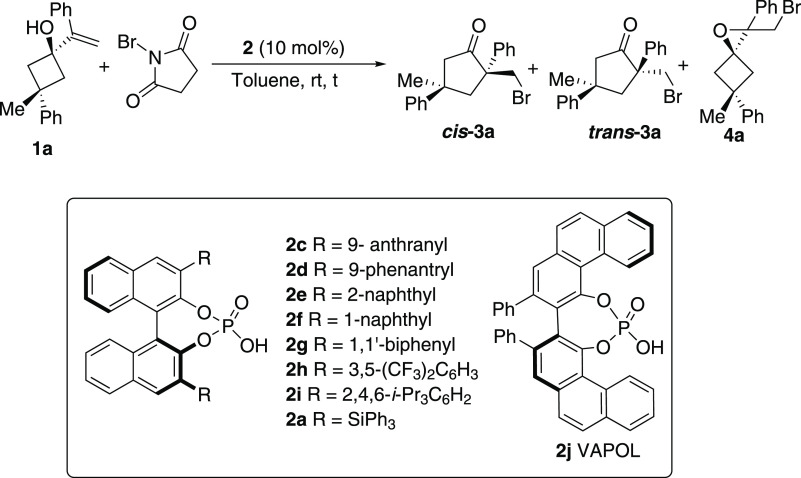
Screening of Chiral Phosphoric Acids^a^

entry	catalyst	conversion	global yield (%)^b^	**3a**:**4a** ratio^c^	dr **3a** (*cis*:*trans*)^c^	ee (%) (*cis*-**3a**/*trans*-**3a**/**4a**)^d^
1	**2c**	100	81	1.2:1	6:1	50/4/50
2	**2d**	90	80	2:1	5:1	20/19/11
3	**2e**	90	77	2:1	5:1	13/17/24
4	**2f**	80	69	1.2:1	5:1	14/14/5
5	**2g**	79	51	2:1	5:1	3/17/13
6	**2h**	100	83	5:1	4:1	38/61/18
7	**2i**	68	53	4:1	5:1	37/10/29
8	**2a**	28	25	6:1	7:1	16/18/15
9	**2j**	78	79	2.5:1	4:1	3/13/9

a Reactions were carried out with 0.10 mmol of cyclobutanol **1a** and 0.11 mmol of NBS in toluene (0.2 M) at room temperature.

b Yields refer to the mixture of isolated products by column chromatography.

c Ratio calculated in the reaction crude by ^1^H NMR.

d Calculated by HPLC on a chiral stationary phase.

However, when we moved to evaluate several of the corresponding alkaline metal phosphates as catalysts (see the Supporting Information), the performance of the reaction improved remarkably in terms of both the yield of isolated cyclopentanone **3a** and stereoselectivity. Remarkably, magnesium phosphate **2b** performed excellently in the reaction, without observing the formation of epoxide side product **4a** and providing **3a** in high yield and high diastereo- and enantioselectivity (Scheme 3).^32^ However, because the formation of the epoxide is always possible in addition to the desired cyclopentanone, we initiated a series of mechanistic studies to determine the factors influencing the formation of both products.

**Figure 2:**
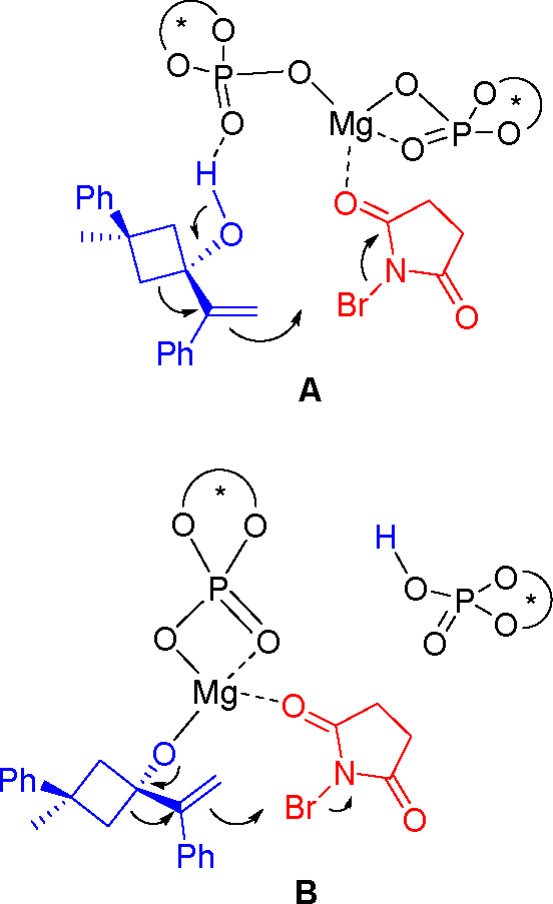
Models of addition.

Two modes of action can be suggested for the catalyst. The first is the concomitant double bond halogenation and H transfer from the alcohol to the catalyst (Figure 2, **A**), as proposed by Masson and co-workers.^32^ A similar cooperative model has been reported for a Pd-bis-phosphate catalyst.^33^ The second is coordination of the alcohol to the metal atom, promoted by deprotonation with loss of a phosphate unit, and then halogenation of the double bond (Figure 2, **B**).

**Figure 3:**
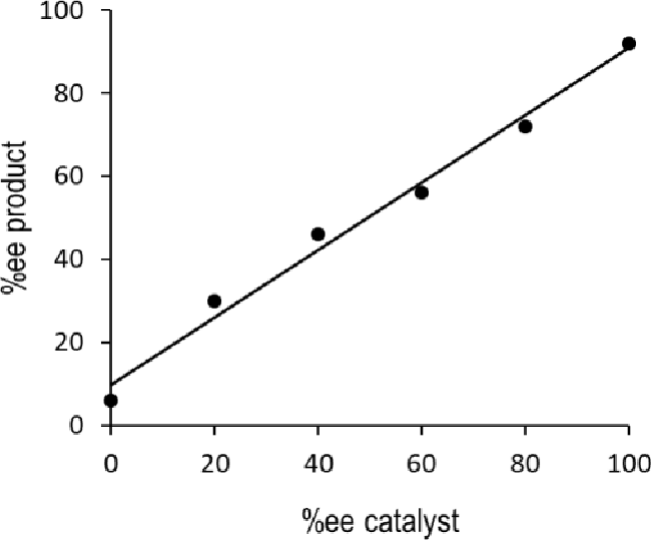
Linear effects collected from the reaction of **1a** with NBS in the presence of preformed catalyst **2b**.

Nonlinear effect experiments were performed to assess the possible presence of one or two phosphoric units in the rate-determining step (rds) and to evaluate the possibility of ion pair formation. The reaction between **1a** and NBS was carried out employing catalyst **2b** under 2.5 mol % catalyst loading formed *in situ* from the magnesium salt and the chiral phosphoric acid at various enantiomeric ratios. From the collected data, the enantioselectivity of the reaction showed a clear linear dependence of the enantiopurity of the catalyst (Figure 3), which is consistent with the hypothesis that only one phosphate unit is present in the rds. Moreover, recording of ^31^P NMR spectra of the preformed catalyst using different enantiomeric ratios of the phosphoric acid showed two signals with integration similar to the used enantiomeric ratio of the phosphoric acid [catalyst **2b** formed from enantiopure phosphoric acid showed only one signal (for details, see the Supporting Information)].

The exchange of the phosphate units revealed by ^31^P NMR supports the equilibrium with an ion pair as suggested in Figure 2. This observation together with the observed linearity points to the participation of one phosphoric unit in the rds of the reaction pointing in favor of model **B**.

**Scheme 4:**
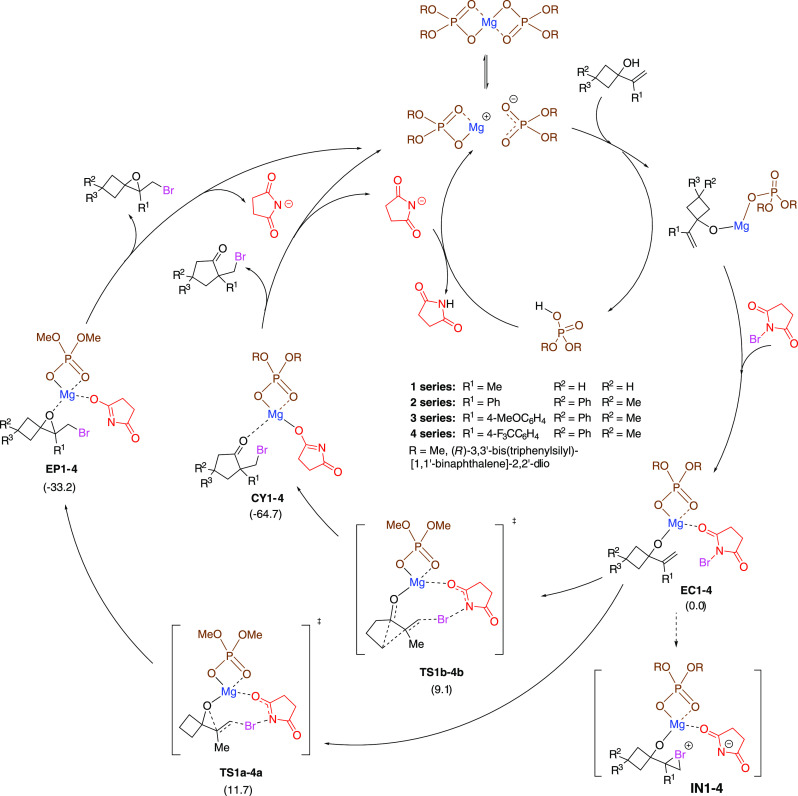
Catalytic Cycle Proposed for the Reaction of **1a** with NBS to Give Cyclopentanones **CY1–4** The alternative cycle to afford epoxides **EP1–4** is also indicated. Intermediates **IN1–4** could not be located at any level of theory. The values between brackets are relative free energies corresponding to preliminary studies with the **1** series calculated at the wb97xd/def2tzvp/pcm=toluene//wb97xd/def2svp level of theory (for details, see the Supporting Information).

Once the plausible presence of one phosphate unit in the rds was confirmed giving validity to model **B**, we turned our attention to the course of the reaction. We proposed a catalytic cycle, and initially, we used a simple model without substituents, replacing the phenyl group with a methyl group and with an achiral catalyst (Scheme 4, **1** series). We computationally studied the whole transformation of the coordinated reagents to the catalyst (encounter complex **EC1**) into the products. All attempts to locate intermediate **IN1** failed, and the structure converged either to the final product (epoxide **EP1**/cyclopentanone **CY1**) or to encounter complex **EC1**.

On the contrary, after a deep exploration of the potential energy surface (PES), transition structures **TS1a** and **TS1b**, corresponding to the formation of the epoxide and the cyclopentanone, respectively [those approaches correspond to the more stable ones (for details, see the Supporting Information)], were located. The IRC of **TS1a** and **TS1b** identified **EC1** as the starting point, and **EP1** and **CY1** as the final points, confirming that the reaction proceeds in one kinetic step without observable intermediates as energy minima. The observed barriers for **TS1a** and **TS1b** were 11.7 and 9.1 kcal/mol, respectively.

**Figure 4:**
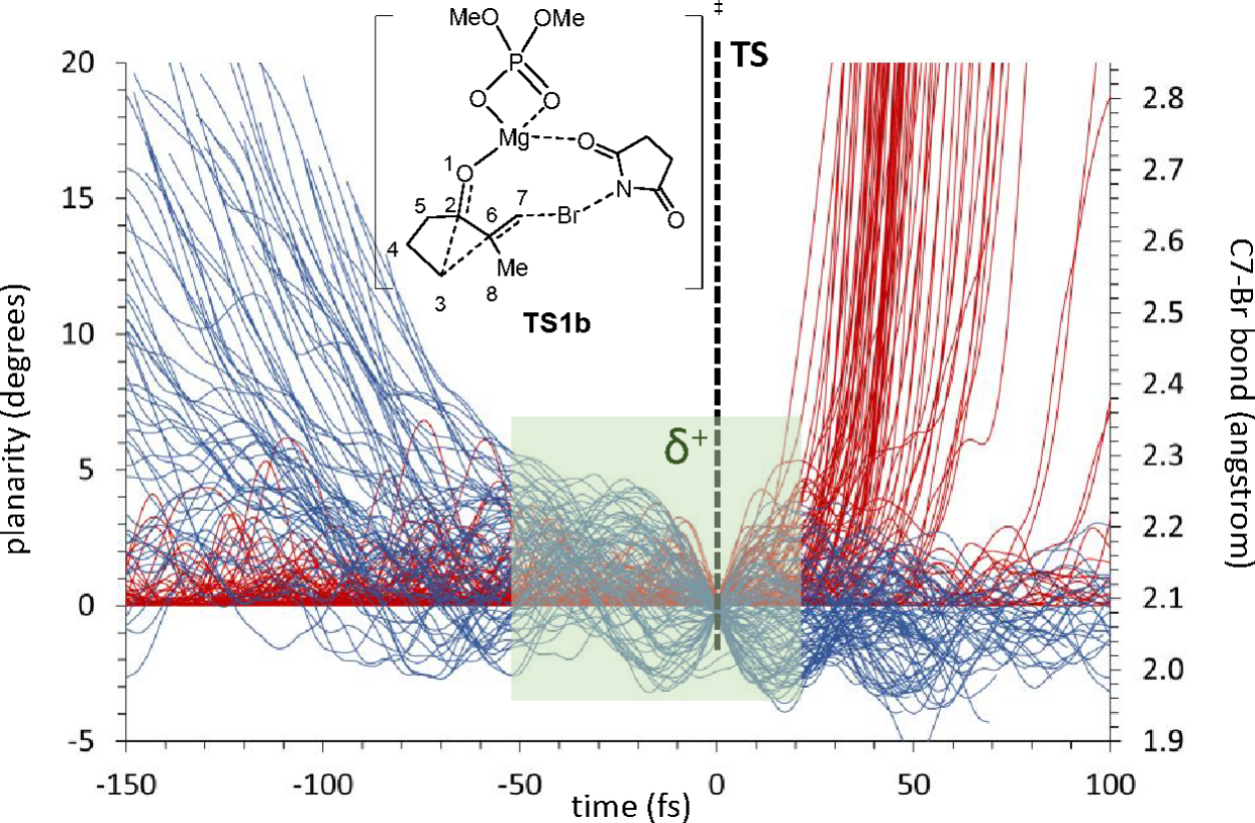
Representation of the C7–Br distance (blue lines) and planarity of C6 {red lines; as 360 – [<(2,6,7) + <(7,6,8) + <(8,6,2)]} for 130 trajectories starting from **TS1b**. The green area indicates, approximately, the minimum time in which only C6 is a sp^2^ carbon, thus corresponding to a carbocation.

Quasi-classical direct dynamic calculations were performed, using PROGDYN,^34^ on **TS1b**, to estimate the half-time in which C6 remains as a sp^2^ carbon after C7 became sp^3^ (formation of the C7–Br bond). Upon comparison of the planarity of C6 (from the start of the reaction to the rearrangement) with the formation of the C7–Br bond, it is possible to estimate a duration of at least ∼70 fs for the period in which only C6 remains planar, corresponding to the transient carbocation (Figure 4).

**Figure 5:**
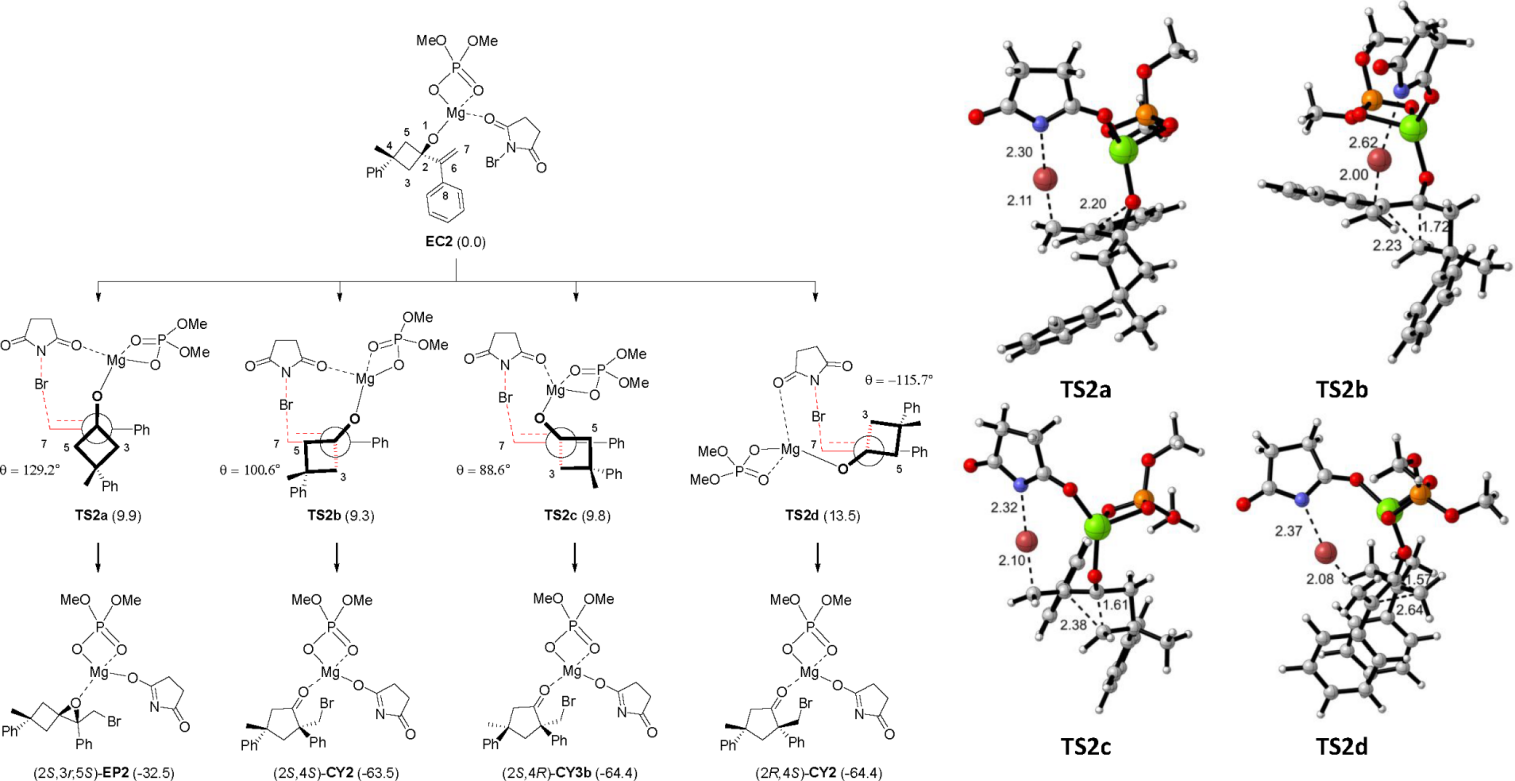
Viable approaches for the reaction of **EC2** leading to **3a** and **4a**. Newmann projections for **TS2a–d** are given along the C2–C6 bond. Breaking and forming bonds are colored red. θ refers to the C7–C6–C2–C3 dihedral angle. Relative free energy values (in brackets) are given in kilocalories per mole (for details, see the Supporting Information).

However, in the real substrate, the presence of an aromatic ring instead of a methyl group should contribute to the notable increase in the stability of a possible carbocationic intermediate. For this reason, and due to the required presence of substituents in the cyclobutane ring for the desymmetrization and the diastereoselectivity, we move to the real substrate, still with the achiral catalyst, to study the course of the reaction. Once the attack of the bromine atom is fixed by a given face of the double bond (we are not yet studying the chiral induction), there are six possible approaches from encounter complex **EC2** to the products (four for cyclopentanone **CY2** and two for epoxide **EP2**) but only four (three for **CY2** and one for **EP2**) are possible due to the coordination of the substrate and the reagent (NBS) to the catalyst (Figure 5).

Again, any attempt to locate an intermediate failed. The four transition structures **TS2a–d** were located, and the geometry was optimized at the wb97xd/def2svp level of theory. More accurate energy values were obtained through single-point calculations at the wb97xd/def2tzvp/pcm=toluene level. Even at this level, the differences between the transition structures are very close, most of them with a <1.0 kcal/mol difference, i.e., within the experimental error of DFT, but in agreement with the observed realization (depending on the reaction conditions) of both epoxide and cyclopentanone.

**Figure 6:**
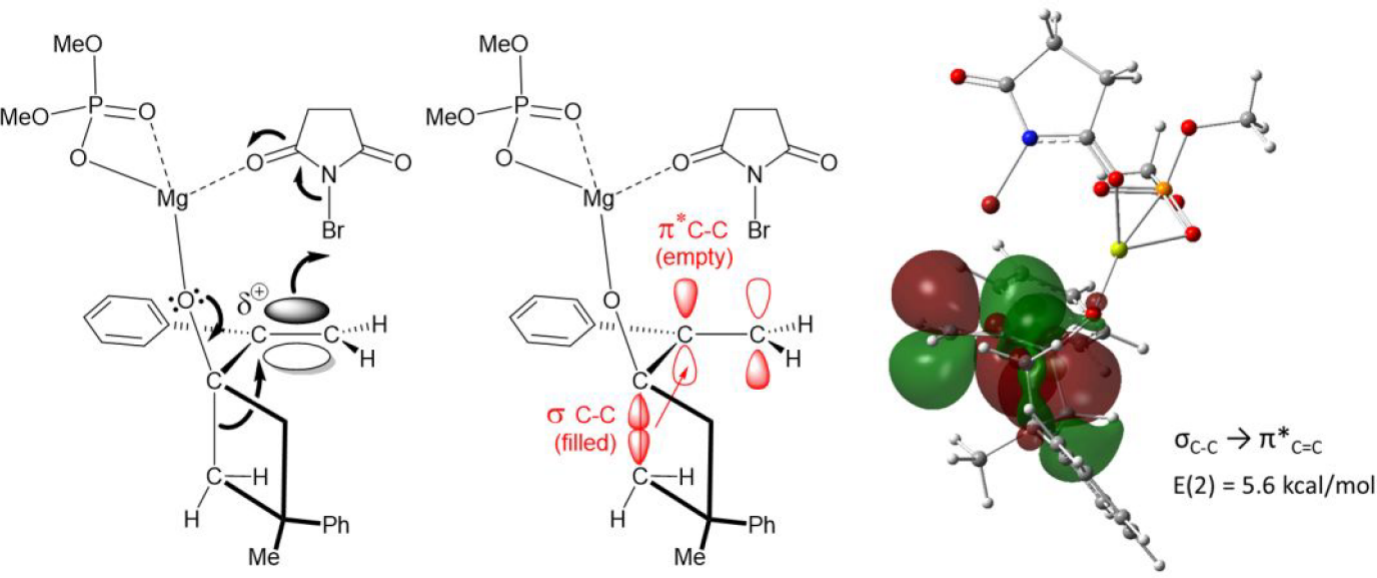
Hyperconjugative effect (σ_C–C_ → π*_C=C_) for the eclipsed conformation leading to TS1b. The second-order perturbation theory analysis of the Fock matrix in NBO donor–aceptor interactions in encounter complex **ECb** gave a stabilizing *E*(2) value of 5.6 kcal/mol.

Interestingly, in **TS2a**, leading to the epoxide, the cyclobutane ring adopts a staggered conformation with respect to the double bond (C7–C6–C2–C3 dihedral angle of 129.2°). On the contrary, for **TS2b** and **TS2c**, leading to isomeric cyclopentanones, and close in energy, an eclipsed conformation is observed (C7–C6–C2–C3 dihedral angles of 100.6° for **TS2b** and 88.6° for **TS2c**). The less stable **TS2d** (C7–C6–C2–C3 dihedral angle of −115.7°) cannot adopt such an eclipsed conformation for steric reasons, resulting in a notable increase in the energy barrier. The eclipsed conformation observed in **TS2b** and **TS2c** placing the rearranging bond at ∼90° with respect to the double bond facilitates a stabilizing hyperconjugative effect of the breaking C–C bond over the double bond [donation of σ(C–C) to π*(C=C) (Figure 6)].

We will focus our attention on the reactions leading to the experimentally observed compounds **EP2** and (2*S*,4*S*)-**CY2**.^35^ A common feature for both IRCs obtained from **TS2a** and **TS2b** in the initial steps is that the attack of the bromine atom on the double bond takes place in an almost perpendicular orientation with respect to C7, clearly promoting the development of a positive charge in C6, stabilized by the aromatic ring. This observation agrees with previous calculations on 2-halo-1-phenylethyl cations^19a^ and rules out the formation of a bromiranium intermediate even as a transient species.

The required alignment between the entering bromine atom (Br–C7 axis) and the rearranging carbon (C3–C2 axis) both at ∼90° with respect to the double bond (C6–C7) observed for **TS2b** and due to the hyperconjugative effect mentioned above confirms the concertedness of the process in which all atoms participating in bond breaking and formation are involved together in an only kinetic step but not acting simultaneously. The ELF analysis applied to an IRC represents the evolution of the electron density (electron population) during the whole reaction. As a consequence, it is possible to analyze the concertedness of the reaction by establishing the moment at which a given bond is broken or formed as well as to analyze changes in the electronic population in bonds and atoms with lone pairs, and to evaluate the presence of transient species.

**Figure 7:**
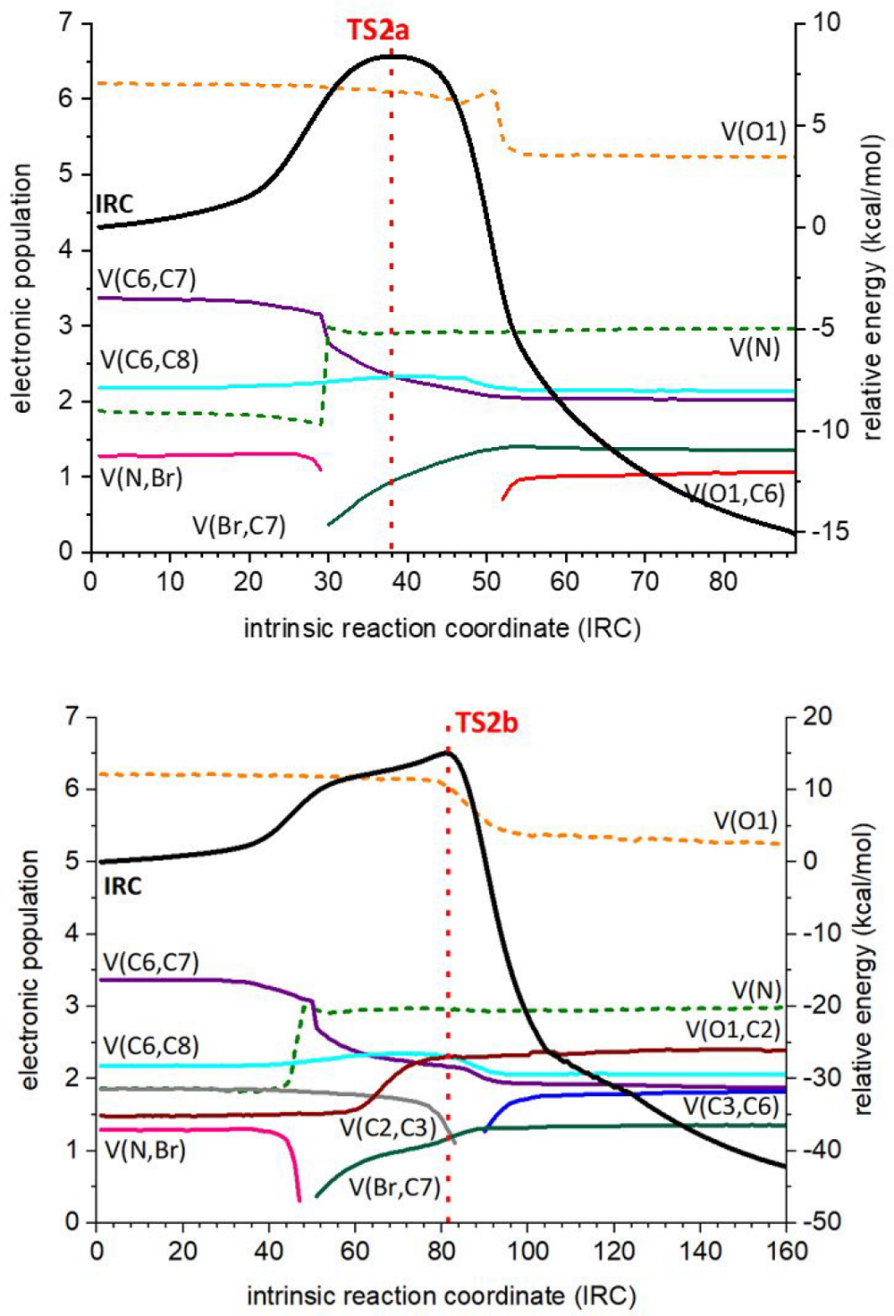
ELF analyses for the reaction of **EC2** (R = Ph) through **TS2a** (top) and **TS2b** (bottom) to give epoxide **EP2** and (2*S*,4*S*)-**CY2**, respectively. The numbering refers to that given in Figure 5. Black traces correspond to IRC. Colored dotted traces refer to lone pairs (monosynaptic basins), and colored plain traces to bonds (disynaptic basins). The vertical red line indicates the transition state. Only those representative atoms and bonds are shown (for the full set of data, see the Supporting Information).

In the case of **TS2a**, leading to epoxide **EP2**, the oxygen atom can attack only by the same face as bromine. The ELF analysis (Figure 7, top) shows the transfer of the bromine atom (breaking of the N–Br bond and formation of the C7–Br bond) at point 29, which is confirmed by the increase in the electron population of V(N).

The formation of the epoxide [appearance of basin V(O1,C6) and loss of the electron population of V(O1)] takes place at point 52, leaving some space for the development of a positive charge at C6. The C6=C7 bond shows a clear loss of electron population when the C7–Br bond is formed (point 30) that continues until it becomes a single bond, just when the O1–C6 bond is formed (point 52). In other words, the direct attack of the bromine atom on C7 induces the collapse of the oxygen atom to capture the emerging transient positive charge.^36^

The ELF analysis corresponding to the IRC of **TS2b** (Figure 7, bottom) gives strong evidence of a transient carbocation (considered as a highly polarized species in which the charge is built up on C6) as it can be advanced by the presence of a shoulder before the transition structure. While the C7–Br bond is formed at point 51, the rearrangement starts at point 83 with the breaking of the C2–C3 bond and ends at point 90 with the formation of the C3–C6 bond. Consequently, we can say that between points 51 and 83 we have a transient carbocation, which is clearly stabilized by the aromatic ring. Indeed, a slight increase in the electron population for the C6–C8 bond can be appreciated, in that interval, corresponding to the delocalization of the positive charge by resonance that increases the bond order between those atoms.

**Figure 8:**
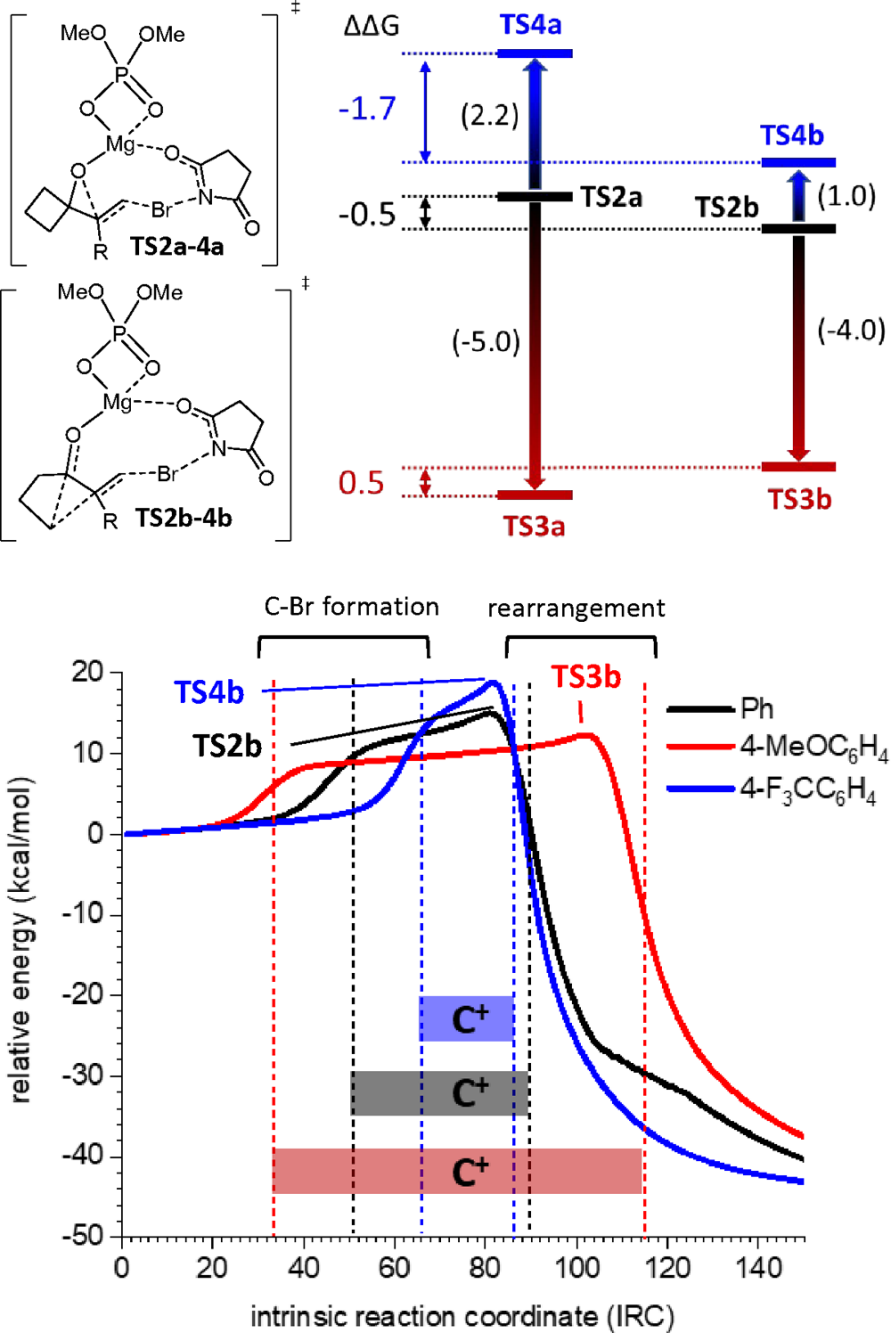
(De)stabilization of **TS2a** (left) and **TS2b** (right) (black traces) upon introduction of MeO (**TS3a** and **T3b**; red traces) and CF_3_ (**TS4a** and **T4b**; blue traces) groups at the *para* position in the aromatic ring linked to the double bond (top). Numbers in brackets indicate the gap (ΔΔ*G* in kilocalories per mole) between the different energy barriers for R = Ph (black), 4-MeOC_6_H_4_ (red), and 4-F_3_CC_6_H_4_ (blue). Colored numbers indicate the relative energies (ΔΔ*G* in kilocalories per mole) between the two series **a** and **b** leading to epoxide and cyclopentanone, respectively. IRCs for **TS2b** (black trace), **TS3b** (red trace), and **TS4b** (blue trace) (bottom). The first dashed line indicates the formation of the C7–Br bond; the second one indicates the rearrangement [according to ELF analyses (see the Supporting Information)]. The corresponding intervals, represented as horizontal bands, indicate the extension of the transient carbocation during the intrinsic reaction coordinate. The superposition of IRCs has been done from the starting point at which the N–Br bond completely formed.

A comparison between the IRCs of **TS1b** (R = Me) and **TS2b** (R = Ph) (see the Supporting Information) evidences the stabilizing effect of the aromatic ring by extending the area of the IRC corresponding to the transient carbocation. To evaluate how substitution of the aromatic ring might affect the reaction, we expand the study to 4-methoxy (R = 4-MeOC_6_H_4_)- and 4-trifluoromethyl (R = 4-F_3_CC_6_H_4_)-substituted substrates at the aromatic ring linked to C6. We located all of the transition structures and evaluated the differences between energy barriers. It is noteworthy that for **EC3** (R = 4-MeOC_6_H_4_), although the values were too close even at the 3ξ level of theory, **TS3a**, corresponding to the obtention of the epoxide, was 0.5 kcal more stable than **TS3b**, corresponding to the formation of the cyclopentanone, predicting that for this substrate epoxide could be obtained preferentially. Moreover, the calculated energy barriers for **EC3** (4.8 and 5.3 kcal/mol for **TS3a** and **TS3b**, respectively) were lower than for **EC2** (9.9 and 9.3 kcal/mol for **TS2a** and **TS2b**, respectively) as expected for the increased level of stabilization of the developing charge at C6. For **EC4** (R = 4-F_3_CC_6_H_4_), the barrier was the highest (12.0 and 10.3 kcal/mol for **TS4a** and **TS4b**, respectively) as a consequence of the electron-deficient character of the substituent, which should shorten the half-life of the transient carbocation. On the contrary, in this case, **TS4b** was found to be 1.7 kcal/mol more stable than **TS4a**, predicting the preferential realization of the corresponding cyclopentanone. As illustrated in Figure 8, the presence of electron-donor substituents stabilizes the transient carbocation and lowers the energy barrier. The formation of the epoxide, i.e., **TS3a**, is more sensitive to this effect than the formation of the cyclopentanone (**TS3b**), and consequently, the former is more stabilized than the latter, resulting in a reversed trend toward the epoxide as the preferred product. Similarly, the destabilization exerted by the trifluoromethyl group affects more **TS4a**, which now is 1.7 kcal/mol less stable than **TS4b**, predicting the realization of the cyclopentanone preferentially.

Therefore, it is possible to conclude that the more the donor is the substituent, the more stable the transient carbocation and the larger the amount of epoxide obtained, predicting a direct correlation between the stability of the cationic species and the amount of epoxide. In fact, for **EC1a** with a methyl group instead of an aromatic one, the gap between **TS1a** and **TS1b** is −2.6 kcal/mol as expected for a nonstabilized transient carbocation.

**Figure 9:**
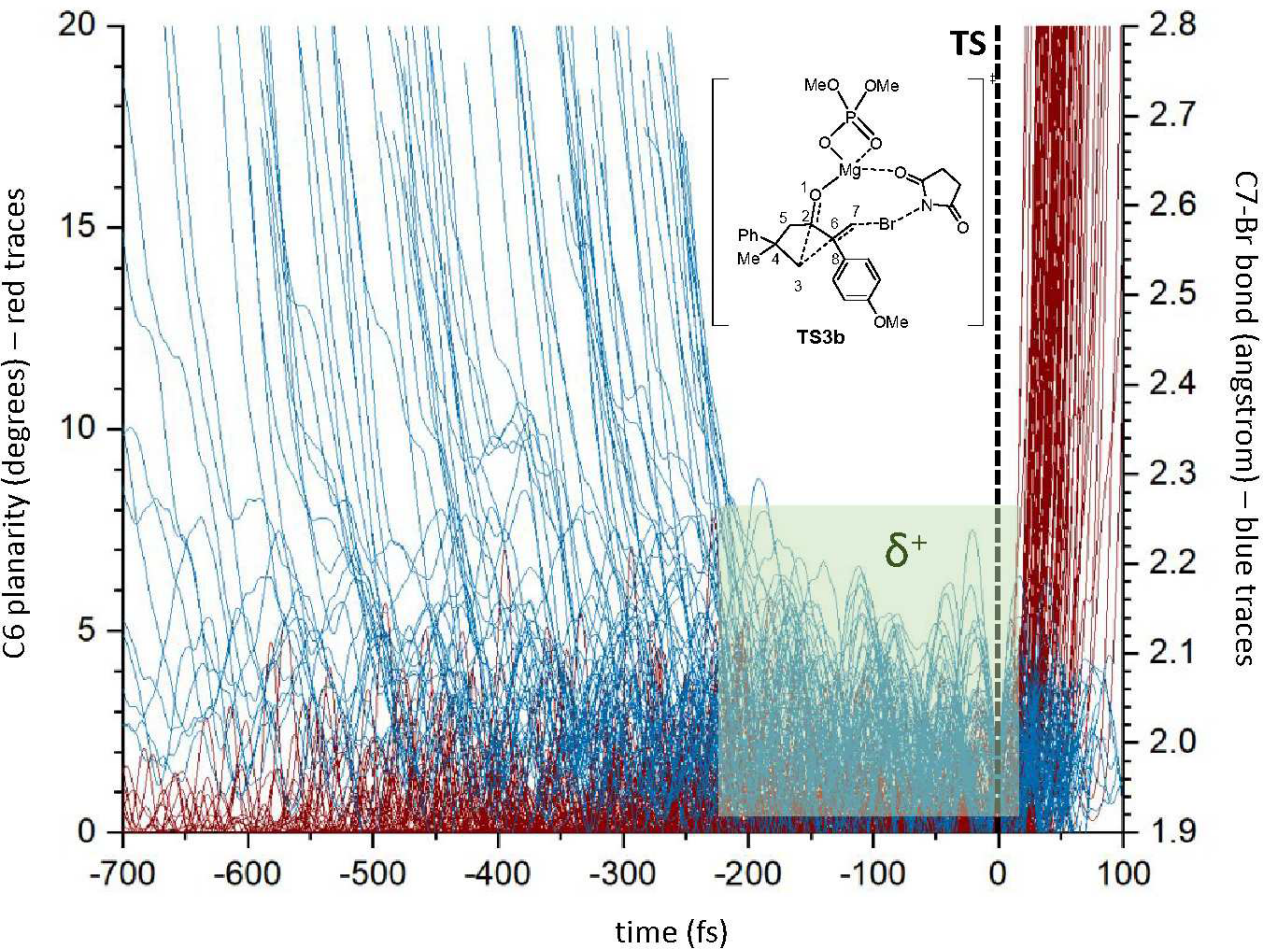
Representation of the C7–Br distance (blue traces) and C6 planarity {red lines; defined in degrees as 360 – [<(2,6,7) + <(7,6,8) + <(8,6,2)]} for 102 trajectories starting from **TS3b**. By comparison of the planarity of C6 (from the start of the reaction until the rearrangement) with the formation of the C7–Br bond, it is possible to estimate the time in which C6 remains planar. The green area represents the minimum time in which only C6 is planar and, consequently, a carbocationic sp^2^ carbon.

The ELF analyses of the IRCs corresponding to **TS3b** and **TS4b** corroborate a large area of existence for a carbocationic species in the first case (methoxy group) and a small area for the second (trifluoromethyl group). The points in which the C7–Br bond is formed and the rearrangement takes place in each case, according to ELF analysis, illustrating the carbocationic area, are given in Figure 8 (bottom) for the superimposed IRCs (for details on these ELF analyses, see the Supporting Information). This comparison also evidences that the more the donor is the substituent of the aromatic ring, the earlier is the formation of the C–Br bond. For **EC2** (R = Ph) and **EC4** (R = 4-F_3_CC_6_H_4_), the rearrangement takes place almost at the same time, resulting in a larger area for the existence of the carbocation in the case of **EC2**. For **EC3** (R = 4-MeOC_6_H_4_), the rearrangement takes place still later, showing a platform corresponding to an almost planar PES that can be assigned to a “hidden carbocation”. Accordingly, the half-time of that transient carbocation should be higher than that found for the unsubstituted model (see Figure 4). In fact, quasi-classical direct dynamic calculations on **TS3b** afforded a window for the carbocationic species of a minimum of 200 fs but with trajectories showing windows of ≤600 fs (Figure 9).

As illustrated in Figure 9 while the rearrangement (hybridization to sp^3^ of C6; end of red traces just after the TS) is concentrated in a small time window for all of the trajectories, the formation of the C7–Br bond (blue trajectories) is spread over a large time window (from 900 to 200 fs), reflecting the planarity observed in the IRC and showing a half-life for the carbocation of 550 ± 350 fs.

We finally forced the reaction to take place through a two-step mechanism by stabilizing to a great extent the carbocation replacing the aromatic ring with a dimethylamino group (R = NMe_2_ in Figure 8). This group maximizes the stability of the carbocation by actually forming an intermediate iminium ion clearly identified as a energy minimum. This substrate, according to the observed trend for the aromatic substitution, has a low barrier with an almost complete preference for the formation of the epoxide, which takes place in an almost barrierless manner (for details, see the Supporting Information).

**Figure 10:**
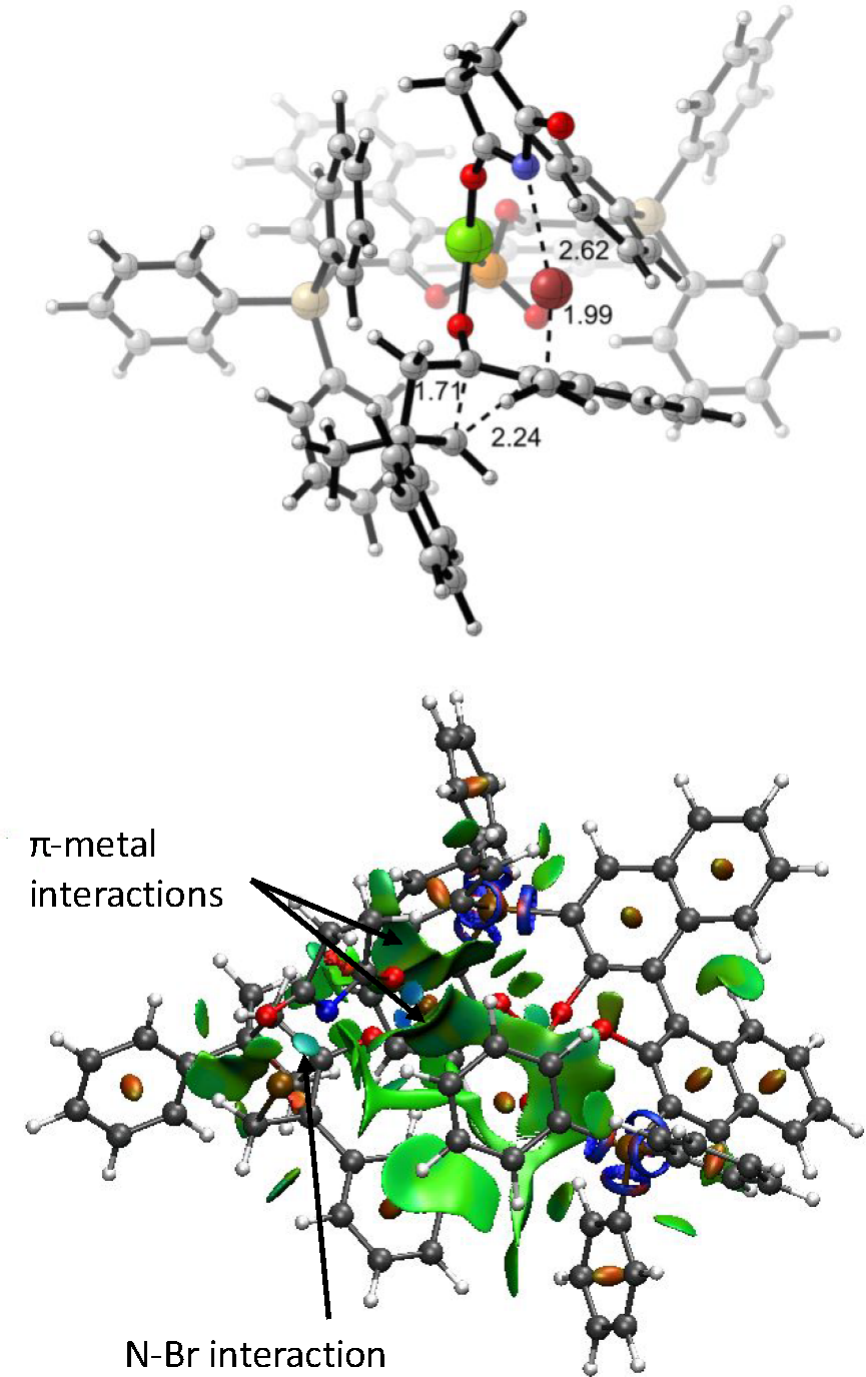
Preferred transition structure **TS2b1** for the reaction between **1a** and NBS, leading to (2*R*,4*R*)-**CY2** (top). NCI analysis (bottom).

Once the concertedness of the reaction had been determined, we studied the real substrate with the real catalyst. Introduction of the chiral catalyst duplicates the number of possible transition structures indicated in Figure 5 (see the Supporting Information). We located the eight transition structures leading to the stereoisomers of **EP2** and **CY2** and found the most stable one, **TS2b1**, corresponds to the formation of (2*R*,4*R*)-**CY2** (Figure 10). The closest one was **TS2a1**, corresponding to the formation of (2*R*,3*R*,5*R*)-**EP2**, which was 2.3 kcal/mol less stable, confirming the preference for the realization of the cyclopentanone in good agreement with the experimental results.

A close inspection of the transition structures with the real catalyst (**TS2a1** and **TS2b1**) showed a disposition of the atoms almost identical to that observed in the corresponding achiral models (**TS2a** and **TS2b**), suggesting that the role of the bis(triphenylsilyl)BINOL moiety is essentially steric (for the rest of the transition structures, see the Supporting Information). Thus, we can confirm an early transition structure for the formation of the epoxide and a late transition structure for the formation of the cyclopentanone. We can confirm the vibration corresponding to the imaginary frequency. In the case of **TS2a1**, it mostly affects the formation of the C–Br bond with little implication of the forming C–O bond. On the contrary, for **TS2b1** the vibration mainly corresponds to the rearrangement, suggesting that the C–Br bond has already been formed. However, the ultimate proof of the concertedness of the reaction arises from the NCI analysis of **TS2b1**. Despite the fact that the C–Br bond can be considered completely formed, a clear interaction between the succinimide nitrogen and the bromine atom remains (Figure 10), indicating that the full process has not yet concluded. The barrier for the reaction was found to be 7.6 kcal/mol for the formation of the epoxide (2*R*,3*R*,5*R*)-**EP2** (through **TS2a1**) and 5.3 kcal/mol for the formation of cyclopentanone (2*R*,4*R*)-**CY2** through **TS2b1**.

**Scheme 5:**
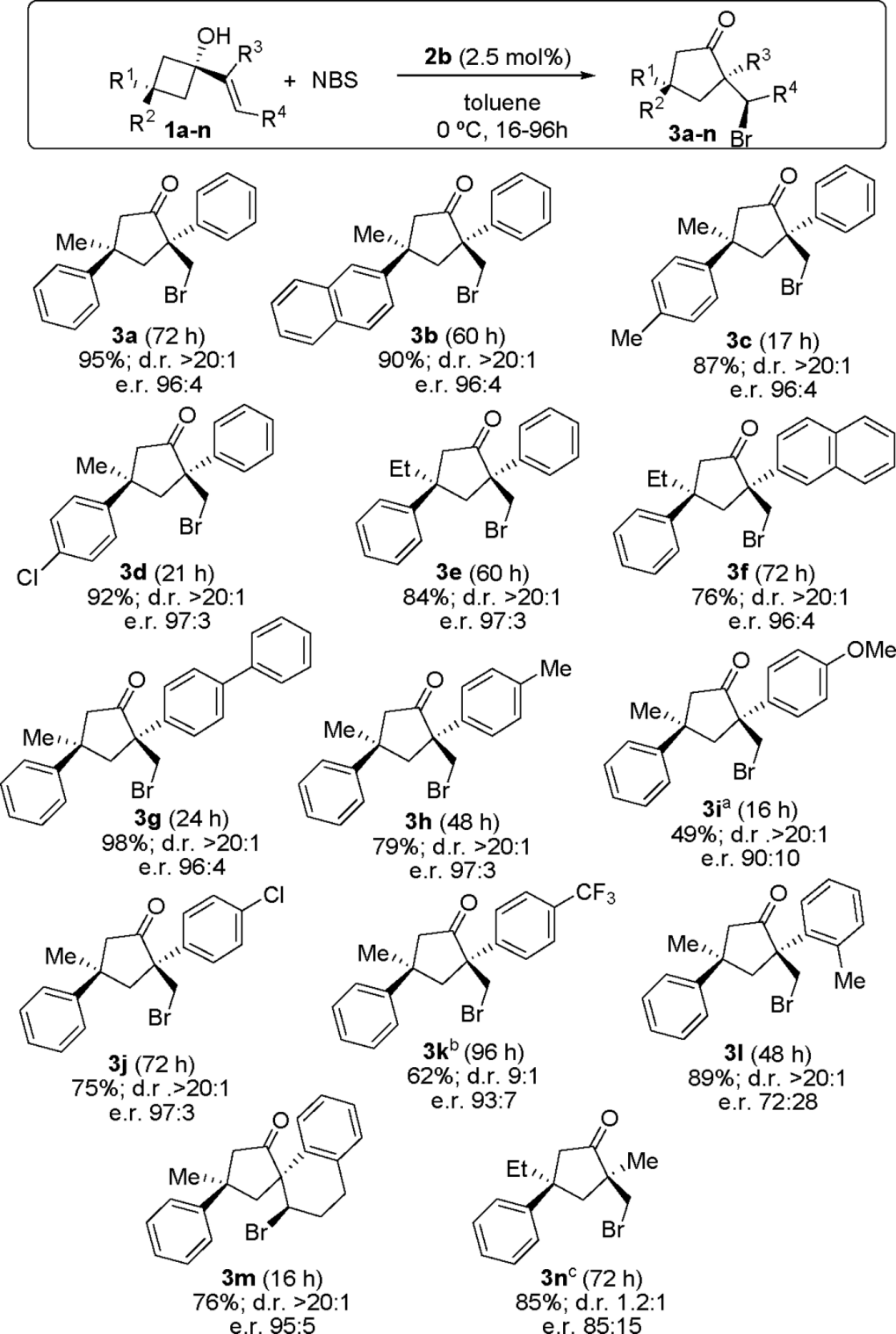
Scope of the Reaction Epoxide **4i** was isolated in 47% yield. The reaction was carried out with 5 mol % catalyst. The other diastereoisomer was obtained with er 86:14. Reactions carried out with 0.1 mmol of **1**, 0.11 mmol of NBS, and 2.5 mol % **2** in toluene (0.2 M) at 0 °C for 16–96 h. Yields refer to pure isolated products. dr values were determined by NMR analysis of crude reaction mixtures, and er values were determined by HPLC on a chiral stationary phase.

The calculations presented above clearly evidenced the influence of the substituents at the aromatic ring as a consequence of the effect in the stabilization of a transient carbocationic species, predicting that the more the donor is the substituent, the greater the amount of epoxide obtained. To verify this prediction, as well as the limitations of the methodology, we decided to explore the scope of the reaction employing the optimized conditions described in Scheme 5 with a selection of substituted compounds, varying position 3 of the starting cyclobutanol and/or the aryl substituent of the alkenyl moiety.

As one can see in this scheme, the reaction performed well when aryl substituents of different electronic properties were placed at position 3 of the starting cyclobutanol, providing the corresponding cyclopentanones **3a–d** in high yield and as single diastereoisomers with a very high er. The same approach was applied with respect to the possibility of increasing the size of the methyl substituent at this position (compounds **3e** and **3f**). The reaction also performed well when cyclobutanol substrates incorporating more π-extended aryl substituents at the alkenyl moiety such as naphthyl (compound **3f**) or biphenyl (compound **3g**) were employed. The influence of the substituents at this aryl substituent was also evaluated, observing that incorporating electron-rich aryl substituents (compounds **3h** and **3i**) resulted in the formation of increasing amounts of undesired epoxide **4** as calculations predicted. The formation of the epoxide was not detected in any other case. Substrates with electron-poor aryl substituents at this position reacted more slowly (compounds **3j** and **3k**), which also led to the use of higher catalyst loading for obtaining synthetically useful yields for the case of **3k**. Despite this, in all cases the final cyclopentanone rearrangement products were obtained with high diastereo- and enantioselectivity. Moving to a more sterically hindered *o*-tolyl substituent at this position led to a slight decrease in enantioselectivity (compound **3l**), although the cyclopentanone adduct could be isolated as a single diastereoisomer in high yield. Using a cyclic dihydronaphthalenyl moiety was also possible, maintaining the excellent performance of the reaction (compound **3m**). Finally, substrate **1n**, in which the aryl moiety of the alkenyl substituent had been replaced with a methyl group, was also found to perform well in the reaction, although a slightly lower enantioselectivity was also observed for the corresponding adduct **3n**, which was also formed as a mixture of diastereoisomers. The absolute configuration of adduct **3j** was unambiguously assigned by X-ray analysis of a monocrystalline sample, and this was extended to all adducts **3** obtained on the basis of a mechanistic analogy.

**Figure 11:**
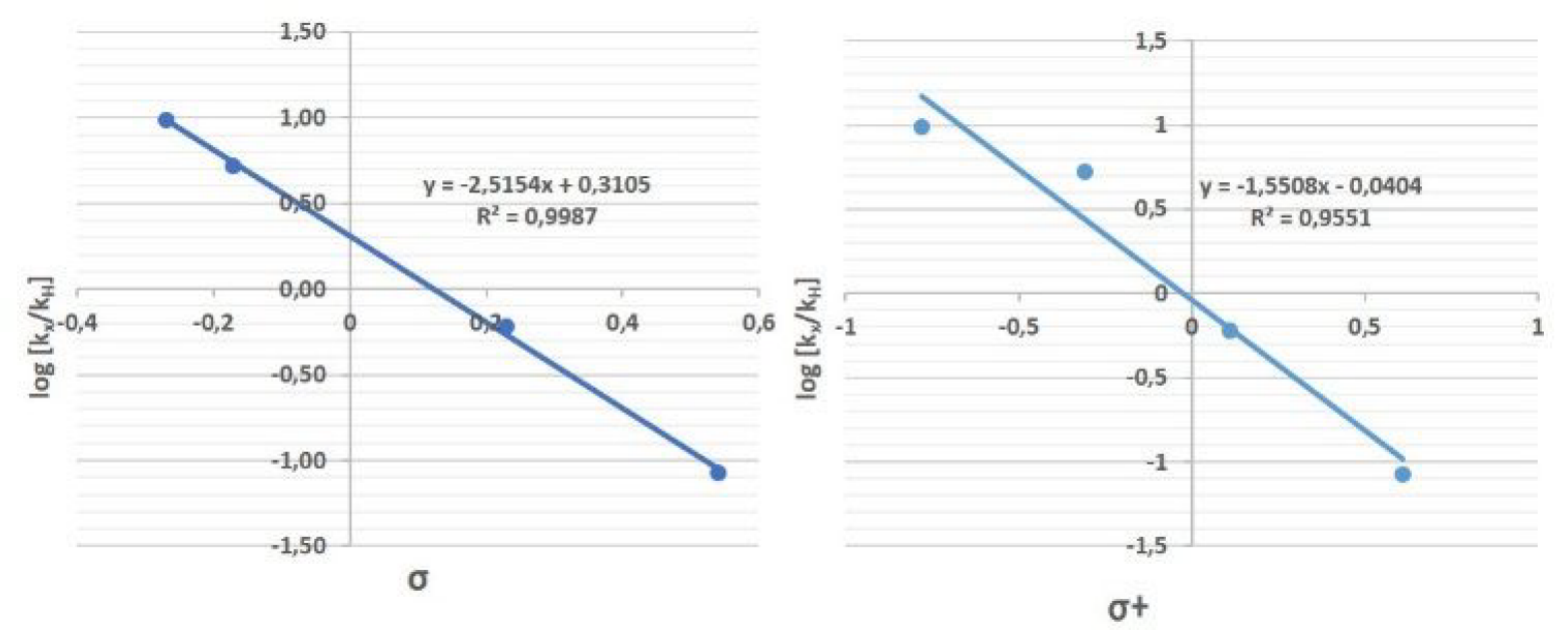
Hammet plot obtained using substrates **3h–3k**.

The effect of the aryl substituents at the alkenyl moiety was also correlated with Hammett parameters. As one can see in Figure 11, a good linear correlation was observed for the four examples studied, corresponding to sensitivity constants ρ = −2.52 and ρ^+^ = −1.55, showing the importance of resonance and inductive effects in this bromination/semipinacol reaction. The negative sensitivity constants are consistent with the generation of a positive charge in the transition structure of the rds. In addition, ρ values are not extremely large, which is also in concordance with a concerted but highly asynchronous mechanism.

## Conclusions

In summary, this study demonstrates that the catalyzed desymmetrizative ring expansion of alkenylcyclobutanols promoted by halofunctionalization of the alkene moiety with NBS using a magnesium phosphate as the catalyst takes place in one kinetic step but two stages through the formation of a hidden carbocationic intermediate with a duration of ≤200 fs. In the studied reaction, the presence of electron-donor substituents increases the stability of transient cationic species favoring the formation of an undesired byproduct altering the regioselectivity of the reaction in addition to the reactivity (reactions with electron-donor substituents are slower than the others).

By using a chiral catalyst introducing two units of a chiral phosphoric acid, very good enantioselectivities are obtained. However, linearity studies support the idea that only one phosphate unit is present in the rds pointing to a mechanism in which the oxygen atom of the alcohol is directly coordinated to the magnesium atom. A good scope was found for the reaction, demonstrating its versatility for the formation of cyclopentanones incorporating quaternary stereocenters as highly enantioenriched materials and also confirming the calculation predictions for reactivity and selectivity. Several mechanisms are possible for halofunctionalization of alkenes, including stepwise processes in which either carbocations or haliranium ions can be formed, and concerted reactions that take place in two stages through the formation of transient carbocations.

## Experimental Section

### General Methods and Materials

#### NMR

Monodimensional and/or bidimensional nuclear magnetic resonance proton and carbon spectra (^1^H and ^13^C NMR, respectively) were acquired at 25 °C on a Bruker AC-300 spectrometer (300 MHz for ^1^H and 75.5 MHz for ^13^C) and a Bruker AC-500 spectrometer (500 MHz for ^1^H and 125.7 MHz for ^13^C) at the indicated temperature. Chemical shifts (δ) are reported in parts per million relative to residual solvent signals (CHCl_3_, 7.26 ppm for ^1^H NMR; CDCl_3_, 77.16 ppm for ^13^C NMR), and coupling constants (*J*) in hertz. The following abbreviations are used to indicate the multiplicity in NMR spectra: s, singlet; d, doublet; t, triplet; q, quartet; app, apparent; m, multiplet; bs, broad signal. ^13^C NMR spectra were acquired in a broad band decoupled mode using DEPT experiments (distorsionless enhancement by polarization transfer) for assigning different types of carbon environments. Selective nOe, NOESY, COSY, HSQC, and HMBC experiments were acquired to confirm a precise molecular configuration and to assist in deconvoluting complex multiplet signals.

#### IR

Infrared (IR) spectra were recorded on a Jasco FT/IR 4100 instrument (ATR), in the interval between 4000 and 400 cm^–1^ with a 4 cm^–1^ resolution. Only characteristic bands are given in each case.

#### MS

Mass spectra (MS) were recorded on an Agilent 7890A gas chromatograph coupled to an Agilent 5975C quadrupole mass spectrometer under electronic impact ionization (EI) at 70 eV. The obtained data are presented in mass units (*m*/*z*), and the values found in brackets belong to the relative intensities comparing to the base peak (100%).

#### HRMS

High-resolution mass spectra were recorded on an Acquity UPLC instrument coupled to a QTOF mass spectrometer (SYNAPT G2 HDMS) using electrospray ionization (ESI+).

#### HPLC

The enantiomeric ratio (er) of the products was determined by high-performance liquid chromatography on a chiral stationary phase in a Waters 2695 chromatograph coupled to a Waters 2998 photodiode array detector. Daicel Chiralpak AS-H and Chiralcel OD-3 and IA columns (0.46 cm × 25 cm) were used; specific conditions are indicated for each case.

#### Melting Points

Melting points were measured in a Buchi B-540 apparatus in open capillary tubes and are uncorrected.

#### Optical Rotations [α]_D_^20^

They were measured at 20 °C on a Jasco P-2000 polarimeter with a sodium lamp at 589 nm and a path of length of 1 dm. The solvent and concentration are specified in each case.

#### X-ray

Data were collected using an Agilent Supernova diffractometer equipped with an Atlas CCD area detector and a Cu Kα microfocus source with multilayer optics (λ = 1.54184 Å; 250 μm full width at half-maximum beam size). The sample was kept at 150 K with an Oxford Cryosystems Cryostream 700 cooler. The quality of the crystals was checked under a polarizing microscope, and a suitable crystal or fragment was mounted on a Mitegen Micromount using Paratone N inert oil and transferred to the diffractometer.

#### Miscellaneous

Analytical grade solvents and commercially available reagents were used without further purification. Anhydrous solvents were purified and dried with activated molecular sieves prior to use.^37^ For reactions carried out under inert conditions, the argon was previously dried through a column of P_2_O_5_ and a column of KOH and CaCl_2_. All of the glassware was dried for 12 h prior to use in an oven at 140 °C and allowed to cool under a dehumidified atmosphere.^38^ Reactions at lower temperatures were carried out using a Termo Haake EK90 refrigerator. Reactions were monitored using analytical thin layer chromatography (TLC), in precoated silica-backed plates (Merck Kiesegel 60 F254). These were visualized by ultraviolet irradiation, *p*-anisaldehyde, phosphomolybdic acid, or potassium permanganate dips.^39^ For flash chromatography, Silicycle 40–63, 230–400 mesh silica gel was used.^40^ For the removal of the solvents under reduced pressure, Büchi R-2 series rotatory evaporators were used.

### Typical Procedure for the Synthesis of **1a**

To a cold solution, 0 °C, of (1*R*,3*R*)-3-methyl-3-phenyl-1-(1-phenylvinyl)cyclobutan-1-ol **1a** (26.4 mg, 0.1 mmol) and magnesium 2,6-bis(triphenylsilyl)dinaphtho[2,1-*d*:1′,2′-*f*][1,3,2]dioxaphosphepin-4-olate 4-oxide **2b** (4.3 mg, 0.0025 mmol) in toluene (0.50 mL) under an Ar atmosphere was added *N*-bromosuccinimide (1.1 equiv, 19.6 mg, 0.11 mmol) in one portion. The mixture was left to stir at 0 °C for 72 h until full conversion by TLC. Once the reaction had reached completion, aqueous saturated Na_2_S_2_O_3_ (1 mL) was added, the layers were separated, and the aqueous layer was extracted with Et_2_O (3 × 2 mL), dried with Na_2_SO_4_, filtered, and concentrated under vacuum. The crude was purified with a short plug of a silica gel column using 9:1 PE/EtOAc to obtain pure **3a** as a colorless oil (32.7 mg, 0.095 mmol, 95%, dr >20:1, 92% ee) by flash chromatography (9:1 PE/EtOAc). The reaction was also carried out on a larger scale. Following the same procedure, **3a** was isolated as a colorless oil (336.2 mg, 0.98 mmol, 98%, dr >20:1, 92% ee) starting from 3-methyl-3-phenyl-1-(1-phenylvinyl)cyclobutan-1-ol (264 mg, 1.00 mmol) and **2b** (43 mg, 0.025 mmol) in toluene (0.5 mmol) and NBS (196 mg, 1.10 mmol). The enantiomeric excess of the product was determined by HPLC using a Chiralpack OD-3 column (99:1 hexane/*i*PrOH, flow rate of 1 mL/min, tr_major_ = 13.1 min, tr_minor_ = 20.4 min). [α]_D_^25^ = +21.1 (*c* = 0.5, CHCl_3_). *R_f_* = 0.36 (95:5 PE/EtOAc). ^1^H NMR (300 MHz, CDCl_3_): δ 7.61–7.55 (m, 2H, C_Arom_–H), 7.46–7.22 (m, 8H, C_Arom_–H), 3.64 (d, *J* = 10.1 Hz, 1H, C**H**_**a**_H_b_Br), 3.47 (d, *J* = 10.1 Hz, 1H, CH_a_**H**_**b**_Br), 3.09 (d, *J* = 13.6 Hz, 1H, C^1^**H**_**a**_H_b_), 3.03 (dd, *J* = 13.6, 1.7 Hz, 1H, C^1^H_a_**H**_**b**_), 2.90 (d, *J* = 18.3 Hz, 1H, C^2^**H**_**a**_H_b_), 2.73 (dd, *J* = 18.3, 1.7 Hz, 1H, C^2^H_a_**H**_**b**_), 1.12 (s, 3H, CH_3_). ^13^C{^1^H} NMR (75.5 MHz, CDCl_3_): δ 216.2 (C=O), 149.0 (C_Arom_–C), 140.1 (C_Arom_–C), 128.9 (2xC_Arom_–H), 128.7 (2xC_Arom_–H), 127.8 (C_Arom_–H), 126.6 (2xC_Arom_–H), 126.4 (C_Arom_–H), 125.3 (2xC_Arom_–H), 57.9 (**C**–CH_2_Br), 52.9 (C^1^H_2_), 45.6 (C^2^H_2_), 41.1 (**C**CH_2_), 40.9 (CH_2_Br), 31.2 (CH_3_). IR (ATR): 3020 (=C–H), 2958 (C–H), 1739 (C=O), 1643 cm^–1^ (C=C). MS (EI): *e*/*z* (%) 263.2 (34), 205.1 (25), 145.1 (39), 117.1 (100). HRMS (ESI^+^): *m*/*z* calcd for [C_19_H_19_OBr + Na]^+^, 365.0517; found, 365.0507 [M + Na]^+^.

### Computational Methods

All of the calculations were performed using the Gaussian09 program.^41^ Computations were performed using the wb97xd functional^42^ in conjunction with standard basis sets def2SVP and def2TZVP.^43^ Full geometry optimizations were performed at the wb97xd/def2SVP level. Single-point calculations using the def2TZVP basis set were carried out over optimized geometries to obtain the energy values. Solvent effects (toluene) were considered using the PCM model.^44^ Benchmarking with additional levels was achieved for the purpose of comparison with experimental results; these levels include functionals m062^45^x and b3lyp,^46^ including Grimme’s correction^47^ and basis sets cc-pvdz^48^ and 6-31G(d,p).^49^ Single-point calculations using cc-pvtz and 6-311G(d,p) basis sets were carried out over optimized geometries to obtain the energy values (for details, see the Supporting Information). The nature of stationary points was defined on the basis of calculations of normal vibrational frequencies (force constant Hessian matrix). Minimum energy pathways for the reactions studied were found by gradient descent of transition states in the forward and backward direction of the transition vector (IRC analysis).^50^ Analytical second derivatives of the energy were calculated to classify the nature of every stationary point, to determine the harmonic vibrational frequencies, and to provide zero-point vibrational energy corrections. The thermal and entropic contributions to the free energies were also obtained from the vibrational frequency calculations, using the unscaled frequencies. The free energy was corrected by substracing the *S_trans_* contribution and considering a 1 M concentration. The real model considering catalyst **2** results in a practical limit placing the system out of reach of a full DFT approach (155 atoms, 690 electrons). For that reason, the multilayer (our own N-layered integrated molecular orbital and molecular mechanics) ONIOM scheme^51^ was used as implemented in Gaussian09 to study the reaction catalyzed by **2**. The entire molecular system was partitioned into two layers, a DFT system treated at the wb97xd/def2SVP level of theory and a semiempirical system.^52^ The semiempirical system consists of the triphenylsilyl substituents of the catalyst (38 atoms). The DFT system consists of the rest of the molecule (117 atoms). Via combination of semiempirical and DFT, the speed of the calculations is dramatically increased without compromising the qualitative analysis for this many-atom system. Structural representations were generated using CYLView.^53^

#### Direct Dynamic Simulations

Downhill molecular dynamics trajectories were run using Singleton’s PROGDYN package,^54^ which interfaces with Gaussian 09 to calculate force constants at each step, every 1 fs, using DFT [ωb97xd/6-31G(d) level of theory in the gas phase] and propagating the nuclei classically. Trajectories were terminated when they had propagated for a total of 1000 fs in either the forward or backward direction, or the following geometric stop criteria were met (see Figure 5). When the N–Br distance dropped below 1.86 Å, the reactant was said to have formed. When the O1–C10 distance dropped below 1.47 Å, the epoxide was said to have formed. The cyclopentanone was said to have formed when the C3–C10 distance dropped below 1.55 Å.

#### ELF Analysis

The electronic structures of stationary points were analyzed by the topological analysis of the gradient field of the electron localization function (ELF)^27a,55^ developed by Silvi and Savin.^56^ The ELF study was performed with TopMod^57^ using the corresponding wave functions of all of the structures of the IRC.

#### NCI Calculations

NCI (noncovalent interactions) were computed using the methodology previously described.^28a^ Quantitative data were obtained with NCIPLOT4.^28c^ A density cutoff of ρ = 0.5 au was applied, and isosurfaces of *s*(**r**) = 0.5 were colored by sign(λ_2_)ρ in the [−0.03, 0.03] au range using VMD software.^58^ Plots of *s*(**r**) versus sign(λ_2_)ρ(**r**) were generated with gnuplot.^59^

## Supporting Information Available

Screening of reaction conditions, full experimental procedures, characterization data, nonlinear effect study, Hammett study, NMR spectra for new compounds, X-ray analysis of **3j**, HPLC traces, computational studies, direct molecular dynamics, optimized geometries, energies, energy profiles, benchmarking, and Cartesian coordinates (PDF)

## Accession Codes

CCDC 2091677 contains the supplementary crystallographic data for this paper. These data can be obtained free of charge via www.ccdc.cam.ac.uk/data_request/cif, or by emailing data_request@ccdc.cam.ac.uk, or by contacting The Cambridge Crystallographic Data Centre, 12 Union Road, Cambridge CB2 1EZ, UK; fax: +44 1223 336033.
