# Supplementary material for: Absence of Intermediates in the BINOL-Derived Mg(II)/Phosphate-Catalyzed Desymmetrizative Ring Expansion of 1-Vinylcyclobutanols
Source: J Org Chem. 2021 Dec 20;87(1):693–707. doi: 10.1021/acs.joc.1c02699 (PMC8749959; doi:10.1021/acs.joc.1c02699)
Supplement: Supplementary file 1 — jo1c02699_si_001.pdf [file jo1c02699_si_001.pdf]

Absence of Intermediates in the BINOL-derived Mg(II)/Phosphate-Catalyzed Desymmetrizative Ring Expansion of 1-Vinylcyclobutanols

Estefania Capel,[a] Marta Rodríguez-Rodríguez, [a] Uxue Uria,*[a] Manuel Pedron,[b] Tomas Tejero,[c] Jose L. Vicario*[a] and Pedro Merino*[b]

[a] University of the Basque Country (UPV/EHU), P.O. Box 644, 48080 Bilbao (Spain).
E-mail: uxue.uria@ehu.eus. joseluis.vicario@ehu.eus
[b] Instituto de Biocomputación y Física de Sistemas Complejos (BIFI). Universidad de Zaragoza, 50009 Zaragoza, Spain.
E-mail: pmerino@unizar.es
[c] Instituto de Sintesis y Catalisis Homogenea (ISQCH). Universidad de Zaragoza-CSIC, 50009 Zaragoza, Spain.

Supporting Information

Table of Contents

Table of Contents S2

General Information S4

Screening of reaction conditions S6

Synthesis and characterization of starting materials 1a-n. S9

- Synthesis of α-vinylarenes i-v. S9
- Synthesis of 3,3-substitutedcyclobutanones I-V. S10
- Synthesis of 1-bromovinylarenes VI-XIV. S12
- Synthesis of 3,3-disubstituted-1-(1-arylvinyl)cyclobutan-1-ols, 1a-1n. S14

Synthesis and characterization of products 3a-3n. S25

Synthesis and characterization of products 4a and 3a S38

Synthesis and characterization of catalysts 2b and 2k-2m. S40

Non linear effect study. S43

Hammett study S44

X-Ray analysis of product 3j S46

^{1}H, $^{13}C\{^{1}H\}$ (75.5 MHz), ^{19}F and ^{31}P NMR spectra of cyclobutanones, 1a-n, 3a-n, 4a, 4i and catalysts 2a-b and 2k-m in $CDCl_3$). S50

^{31}P NMR (122 MHz, $CDCl_3$) spectra of catalyst mixtures for non linear effect study.... .... S95

HPLC analysis S98

Computational Studies S112

The Catalyst S112

Unsubstituted Model S113

Real model with Achiral Catalyst S119

Optimized geometries S120

Intrinsic reaction coordinates S122

Energies S124

Benchmarking S125

Energy profiles S128

NCI Analysis S130

ELF Analysis S133

Stabilization of the intermediate S150

Real Model with Real Catalyst S153

Molecular Dynamic Simulations S157

Cartesian Coordinates S162

General Information

Analytical grade solvents and commercially available reagents were used without further purification. *Anhydrous solvents* were purified and dried with activated molecular sieves prior to use.[1] For reactions carried out under inert conditions, the argon was previously dried through a column of P_2O_5 and $CaCl_2$. All the glassware was dried for 12 hours prior to use in an oven at 140ºC, and allowed to cool under a dehumidified atmosphere. Reactions were monitored using analytical thin layer chromatography (TLC), in pre-coated silica-backed plates (Merck Kiesegel 60 F254). *Flash column chromatography* was performed on standard silica gel (Silicycle 40-63, 230-400 mesh) using standard visualizing agents: UV fluorescence (254 and 366 nm), potassium permanganate/Δ and phosphomolybdic acid stains (PMA)/Δ. For the removal of the solvents under reduced pressure Büchi R series rotatory evaporators were used. For precision weighting Sartorius Analytical Balance Practum 224-1S was used (±0.1 mg). *NMR spectra* were recorded at 25ºC on a Bruker AC-300 spectrometer (300 MHz for ^{1}H and 75.5 MHz for ^{13}C). ^{1}H NMR and ^{13}C{^{1}H} NMR chemical shifts (δ) are reported in ppm with the solvent (or TMS) resonance as the internal standard ($CHCl_3$: 7.26 ppm (^{1}H)) and ($CDCl_3$: 77.16 ppm (^{13}C). Data are reported as follows: chemical shift, multiplicity (d = doublet, t = triplet, q = quartet, br = broad, m = multiplet), coupling constants (Hz) and integration. *High resolution mass spectra (HRMS)* were recorded using an Aquity UPLC coupled to a QTOF mass spectrometer (SYNAPT G2 HDMS) using electrospray ionization (ESI+). *GC-MS analyses* were performed on an Agilent 7890A gas chromatograph coupled to an Agilent 5975 quadrupole mass spectrometer under electronic impact ionization (EI) 70 eV. *Melting points (M.p.)* were measured in a Stuart SMP30 apparatus in open capillary tubes and are uncorrected. The enantiomeric excess (e.e.) of the products was determined by *High Performance Liquid Chromatography* on a chiral stationary phase in a Waters chromatograph coupled to a Waters photodiode array detector. Daicel Chiralpak AS-H and Chiralcel IA and OD-3 columns (0.46 x 25 cm) were used; specific conditions are indicated for each case. *Specific optical rotations* ($[\alpha]_D^{20}$) were measured at 20ºC on a Jasco P-2000 polarimeter with sodium lamp at 589 nm and a path of length of 1 dm. Solvent and concentration are specified in each case. *Infrared spectra* (IR) were measured

[1](a) W. L. F. Armarego, C. L. L. Chai. Purification of Laboratory Chemicals, 7th ed.; Elsevier: Oxford, 2012. (b) D. B. G. Williams, M. Lawton, *J. Org. Chem.* **2010**, *75*, 8351.

in a Jasco FT/IR 4100 (ATR) in the interval between 4000 and 600 cm^{-1} with a 4 cm^{-1} resolution. *X-ray* data collections were performed in an Agilent Supernova diffractometer equipped with an Atlas CCD area detector, and a CuKα micro-focus source with multilayer optics (λ = 1.54184 Å, 250 μm FWHM beam size). The sample was kept at 150 K with an Oxford Cryosystems Cryostream 700 cooler. The quality of the crystals was checked under a polarizing miscroscope, and a suitable crystal or fragment was mounted on a Mitegen MicromountTM using Paratone N inert oil and transferred to the diffractometer.

Screening of reaction conditions

Table S 1. Screening of brominating agents.[a]

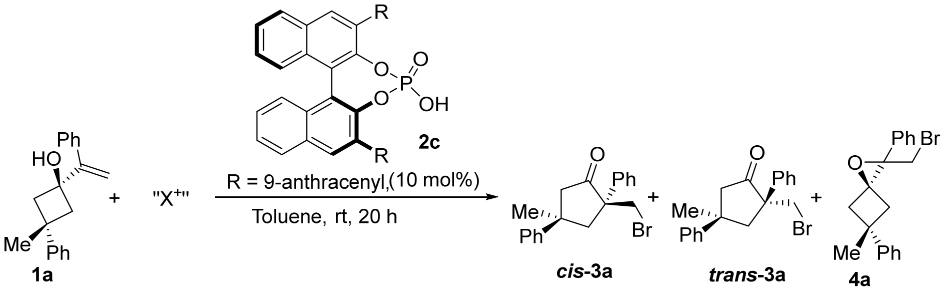

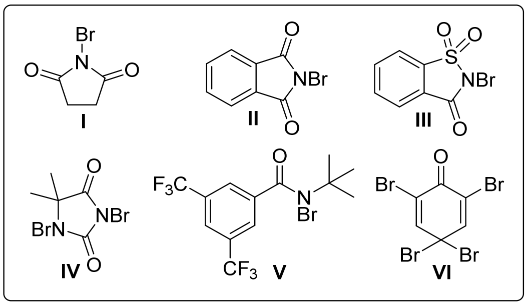

Entry	"X+"	Conversion	Global Yield (%)[b]	Ratio (3a:4a)[c]	dr 3a (*cis*: *trans*)[c]	ee (%) (*cis*-3a/*trans*-3a/4a)[d]
**1**	**I**	Complete	81	1.2:1	6:1	50/5/50
**2**	**II**	31	n.d.	1:1.5	1:-	50/-/50
**3**	**III**	66	61	6:1	2:1	33/40/25
**4**	**IV**	95	70	1.3:1	3:1:	40/74/22
**6**	**V**	83	74	5:1	4:1	30/40/25
**7**	**VI**	78	49	1:1	1:-	22/-/30

[a] Reactions were carried out with 0.10 mmol of ciclobutanol **1a** and 0.11 mmol of NBS in toluene (0.2 M) at room temperature. [b] Yields refer to the mixture of isolated products by column chromatography. [c] Ratio calculated in reaction crude by ^{1}H-RMN [d] Calculated by HPLC on chiral stationary phase.

Table S 2. Screening of metal phosphates.[a]

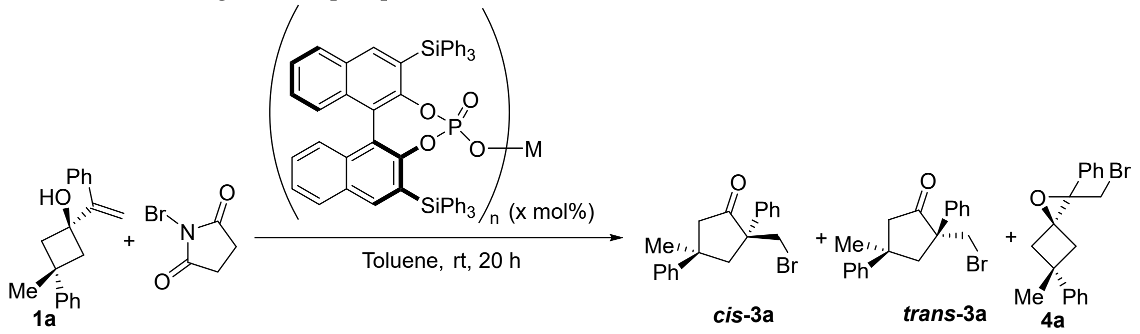

Entry	x mol %	Cat.	M^{n+}	Global Yield (%)[b]	Ratio ($3a:4a_{trans}$)[c]	dr 3a (*cis*:*trans*)[c]	ee *cis*-3a (%)[d]
**1**[e]	5	**2b**	Mg^{2+}	89	>20:1	16:1	87
**2**[e]	5	**2k**	Ca^{2+}	87	19.5:1	7:1	60
**3**[f]	10	**2l**	Na^{+}	60	7:1	10:1	35
**4**[f]	10	**2m**	K^{+}	81	13:1	5.5:1	66

[a] Reactions were carried out with 0.10 mmol of ciclobutanol **1a** and 0.11 mmol of NBS in toluene (0.2 M) at room temperature. [b] Yields refer to the mixture of isolated products by column chromatography. [c] Ratio calculated in reaction crude by ^{1}H-RMN [d] Calculated by HPLC on chiral stationary phase. [e] Reactions carried out with 5 mol % of chiral phosphate. [f] Reactions carried out with 10 mol % of chiral phosphate.

Table S 3. Solvent screening.[a]

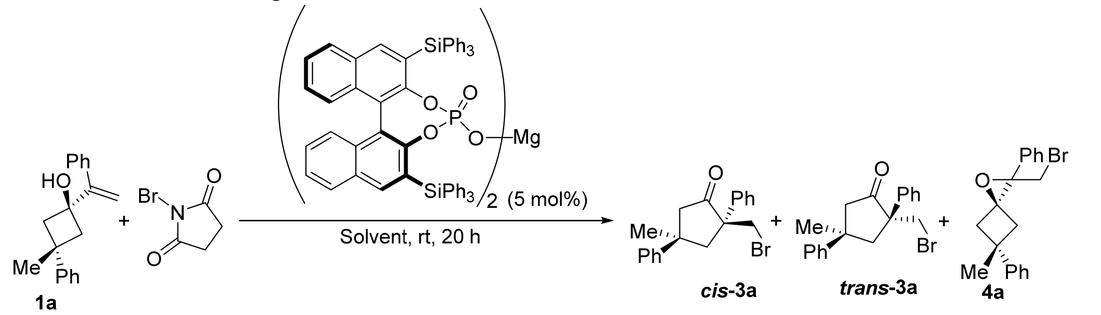

Entry	Solvent	Global Yield (%)[b]	Ratio (3a:4a)[c]	dr 3a (*cis*:*trans*)[c]	ee *cis*-3a (%)[d]
**1**	**Toluene**	89	>20:1	16:1	87
**2**	**DCM**	84	3.7:1	10:1	Racemic
**3**	**AcOEt**	73	2.7:1	7:1	32
**4**	**THF**	73	3.5:1	6:1	6
**5**	***o*-xylene**	83	>20:1	14:1	79
**6**	***m*-xylene**	84	>20:1	13:1	87
**7**	**Mesitylene**	81	>20:1	11:1	88

[a] Reactions were carried out with 0.10 mmol of ciclobutanol **1a** and 0.11 mmol of NBS in toluene (0.2 M) at room temperature. [b] Yields refer to the mixture of isolated products by column chromatography. [c] Ratio calculated in reaction crude by ^{1}H-RMN. [d] Calculated by HPLC on chiral stationary phase.

Table S 4. Effect of temperature.[a]

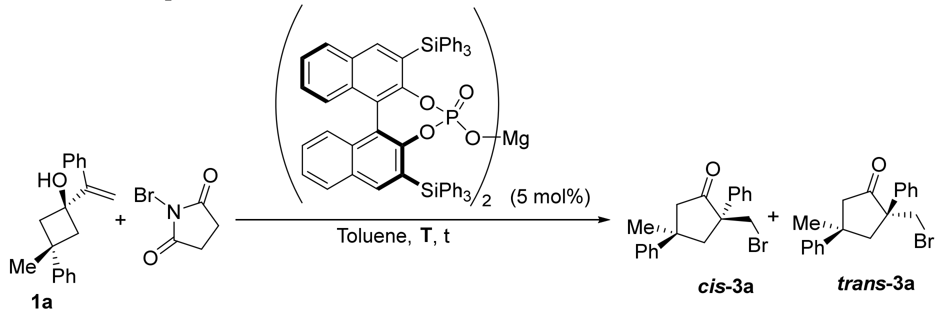

Entry	T (ºC)	t (h)	Yield. 3a (%)[b]	Ratio 3a (*cis*:*trans*)[c]	ee *cis*-3a (%)[d]
**1**	**rt**	20	89	16:1	87
**7**	**50**	8	88	9.5:1	87
**8**	**0**	20	90	>20:1	91
**9**	**-20**	48	89	>20:1	92
**10[e]**	**0**	72	95	>20:1	92

[a] Reactions were carried out with 0.10 mmol of ciclobutanol **1a** and 0.11 mmol of NBS in toluene (0.2 M). In all cases the ketone:epoxide ratio was > 20:1. [b] Yields refer to the mixture of isolated products by column chromatography. [c] Ratio calculated in reaction crude by ^{1}H-RMN. [d] Calculated by HPLC on chiral stationary phase. [e] Reaction carried out with 2.5 mol % of chiral phosphate **2b.**

Synthesis and characterization of starting materials 1a-n.

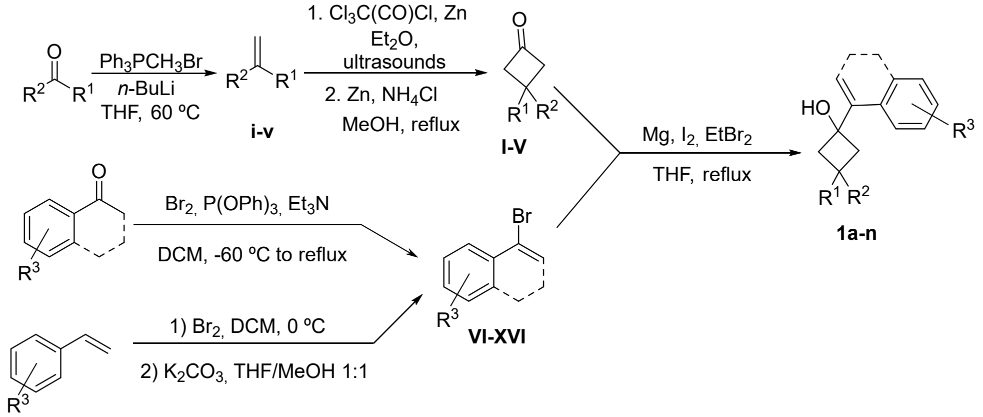

Scheme S 1. General overview of the synthesis of starting materials **1a-n**.

- **Synthesis of α-vinylarenes i-v.**

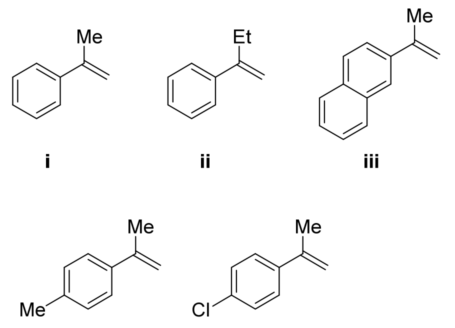

Figure S 1. α-vinylarenes employed for the synthesis of cyclobutanones.

Compound **i** was purchased from commercial sources and used without further purification. **ii-v**[2] are reported compounds and were prepared by Wittig olefination of the corresponding ketones following the procedures described in the literature[3].

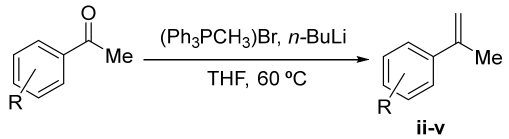

Scheme S 2. Synthesis of α-vinylarenes from carbonyl compounds **ii-v**.

[2]J. Huang, G. Hu, S. An, D. Chen, M. Li, P. Li, *J. Org. Chem.* **2019**, *84*, 9758−9769.
[3]L. Zhang, W. R. Dolbier, B. Sheeller, K. U. Ingold, *J. Am. Chem. Soc.* **2002**, *124*, 6362−6366.

- **Synthesis of 3,3-substitutedcyclobutanones I-V.**

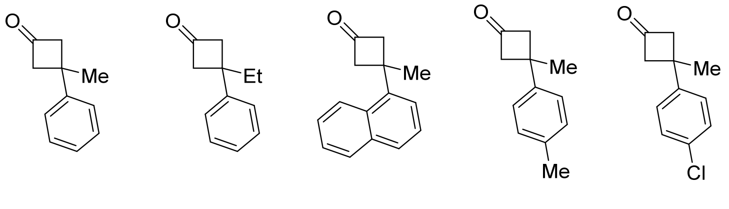

Figure S 2. 3,3-disubstitutedcyclobutanones employed for the synthesis of cyclobutanols.

I[4], **II**[5] and **IV-V**[6] are reported compounds. Compounds **I-V** were prepared following the general procedure A[7] previously described.

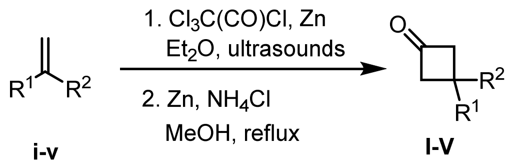

Scheme S 3. General procedure A for the synthesis of cyclobutanones.

General procedure A for the synthesis of cyclobutanones.

In a two necked flask provided with a condenser with dry Et_2O (250 mL) and Zn (4 equiv., 7.8 g, 120 mmol) under Ar atmosphere, α-methylstyrene (3.90 mL, 30 mmol) was added followed by the dropwise addition of trichloroacetyl chloride (2 equiv., 6.80 mL, 60 mmol) under sonication. The mixture was kept under sonication for 5 h until a brown solution was obtained. Then, the solids were filtrated off through a plug of Celita® and washed with Et_2O (100 mL). The filtrate was washed with H_2O (2 x 50 mL), saturated aq. $NaHCO_3$ (4 x 50 mL) and brine, dried with Na_2SO_4 and concentrated under vacuum. The crude was passed through a short pad of silica gel with PE:EtOAc 9:1 to obtain 2,2-dichloro-3-methyl-3-phenylcyclobutan-1-one (6.051g, 26.5 mmol) pure enough for the next reaction.

MeOH (70 mL) was added into a flask containing the crude of the previous reaction followed by the addition of ammonium chloride (2.5 equiv., 3.4 g, 63 mmol). The mixture

---

[4] K. S. Petersen, B. M. Stoltz, *Tetrahedron*, **2011**, *67*, 4352-4357.

[5] (a) W.T. Brady, T. C. Cheng, *J. Organomet. Chem.*, **1977**, *137*, 287-292. (b) T. Seiser, N. Cramer, *Angew. Chem. Int. Ed.* **2008**, *47*, 9294.

[6] T. Matsuda, I. Yuihara, *Chem. Commun.*, **2015**, *51*, 7393-7396.

[7] F. Le Vaillant, M. Garreau, S. Nicolai, G. Gryn'ova, C. Corminboeuf, J. Waser, *Chem. Sci.*, **2018**, *9*, 5883-5889.

was cooled to 0 ºC and Zn (5 equiv., 8.2 g, 126 mmol) was added portion wise under stirring. The flask was provided with a condenser and let stirring at 70 ºC employing a heating mantle overnight. The solids were filtered off trough a plug of Celite®. The filtrate was concentrated under vacuum and diluted with Et_2O (20 mL) and aq. 1M HCl (20 mL). The layers were separated, and the aqueous layer was extracted with Et_2O (3 x 20 mL). The crude was purified with PE:EtOAc 9:1 to obtain 3-methyl-3-phenylcyclobutan-1-one (2.6 g, 16.25 mmol) as a colorless oil.

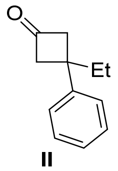

II

3-ethyl-3-phenylcyclobutan-1-one **II**[4]

Following the general procedure A, **II** was purified by flash chromatography (PE:EtOAc 9:1) and obtained as an oil (650 mg; 3.73 mmol; 40 %) starting from α-ethylstyrene (1.2 g, 9.14 mmol), trichloroacethyl chloride (2.0 mL, 18.3 mmol), Zn (2.4 g; 36.6 mmol) in Et_2O (15.0 mL). Rf = 0.48 (PE:EtOAc = 9:1). **^{1}H NMR** (300 MHz, $CDCl_3$) δ 7.41–7.34 (m, 3H, C_{Arom}-H), 7.31– 7.21 (m, 2H, C_{Arom}-H), 3.46–3.35 (m, 2H, C^1H_2), 3.19–3.08 (m, 1H, C^2H_2), 1.88 (q, J = 7.3 Hz, 1H, $\mathbf{CH_2}CH_3$), 0.78 (t, J = 7.3 Hz, 3H, $CH_2\mathbf{CH_3}$). **^{13}C{^{1}H} NMR** (75.5 MHz, $CDCl_3$) δ 206.6 (CO), 145.9 (C_{Arom}-C), 128.3 (2xC_{Arom}-H), 126.8 (2xC_{Arom}-C), 126.2 (C_{Arom}-H), 57.2 (2xCH_2), 38.4 (**C**CH_2), 36.0 ($\mathbf{CH_2}CH_3$), 9.8 ($CH_2\mathbf{CH_3}$). **IR** (ATR) cm^{-1}: 1716.34 (C=O). **MS** (EI) m/z (%): 173.1 (M^+, 1), 132.1 (71), 117.1 (100).

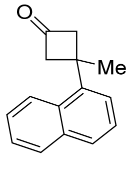

III

3-methyl-3-(naphthalen-1-yl)cyclobutan-1-one **III**

Following the genera procedure A, **III** was purified by flash chromatography (PE:EtOAc 9:1) obtained as a white solid (1.0 g; 4.76 mmol; 45 %) starting from 2-(prop-1-en-2-yl)naphthalene (1.8 g, 10.57 mmol), trichloroacethyl chloride (2.4 mL, 21.14 mmol), Zn (2.8 g; 42.3 mmol) in Et_2O (42.0 mL). Rf = 0.52 (PE:EtOAc = 9:1). **M.p.** = 42-43 ºC. **^{1}H**

NMR (300 MHz, $CDCl_3$) δ 7.92–7.80 (m, 3H, C_{Arom}-H), 7.74 (d, *J* = 1.8 Hz, 1H, C_{Arom}-H), 7.57–7.41 (m, 3H, C_{Arom}-H), 3.67–3.53 (m, 2H, C^1H_2), 3.29–3.13 (m, 2H, C^2H_2), 1.71 (s, 3H, CH_3). **^{13}C{^{1}H} NMR** (75.5 MHz, $CDCl_3$) δ 206.5 (C=O), 145.5 (C_{Arom}-C), 133.3 (C_{Arom}-C), 131.9 (C_{Arom}-C), 128.6 (C_{Arom}-H), 127.7 (C_{Arom}-H), 127.6 (C_{Arom}-H), 126.4 (C_{Arom}-H), 125.8 (C_{Arom}-H), 124.4 (C_{Arom}-H), 123.7 (C_{Arom}-H), 59.3 ($2xCH_2$), 34.1 (**C**CH_2), 30.9 (CH_3). **IR** (ATR) cm^{-1}: 1781.9. **MS** (EI) m/z (%): 210.2 (M^+, 98), 168.2 (100), 153.1 (99), 128.1 (62). **HRMS** (ESI^+): m/z calculated for $[C_{15}H_{14}ONa]^+$ 233.0942; found 233.0940 $[M+Na]^+$

- **Synthesis of 1-bromovinylarenes VI-XIV.**

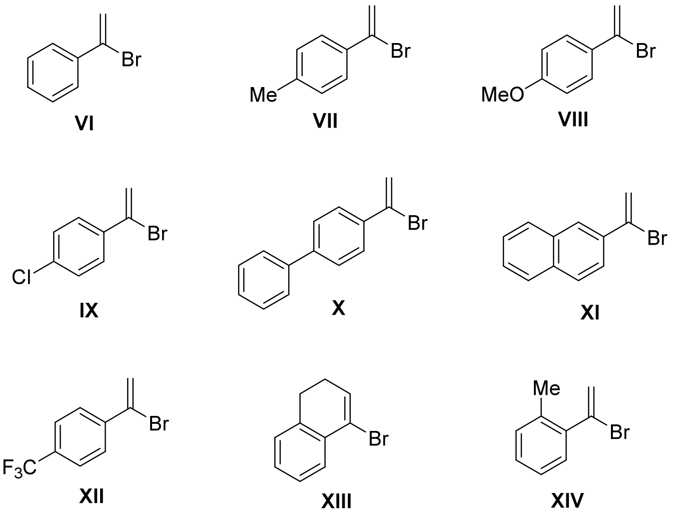

Figure S 3. 1-Bromovinylarenes employed for the formation of organomagnesium compounds.

VI was obtained from commercial sources and used without previous purification. Compounds **VII-XII**[8] , **XIII**[9] and **XIV**[8] are reported compounds.

VII-XI and **XIII-XIV** were prepared following the general procedure B[10] and **XII** following the general procedure C[11] described in the literature.

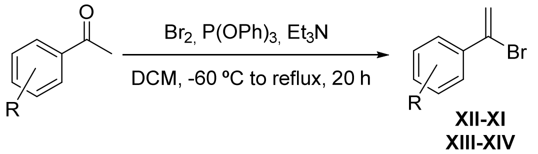

Scheme S 4. General procedure B for the synthesis of 1-bromovinylarenes.

---

[8] S. Alazet, J. Preindl, R. Simonet-Davin, S. Nicolai, A. Nanchen, T. Meyer, J. Waser, *J. Org. Chem.* **2018**, 83, 12334-12356.
[9] E. Napolitano, R. Fiaschi, E. Mastrorilli. *Synthesis*, **1986**, *2*, 122-125.
[10] A. Spaggiari, D.Vaccari, P. Davoli, G. Torre, F. Prati, *J. Org. Chem.* **2007**, *72*, 2216-2219.
[11] A. V. Chernykh, D. S. Radchenko, A. V. Chernykh, I. S. Kondratov, N. A. Tolmachova, O. P. Datsenko, M. A. Kurkunov, S. X. Zozulya, Y. P. Kheylik, K. Bartels, C. G. Daniliuc, G. Haufe. Eur. *J. Org. Chem.* **2015**, 29, 6466-6471.

General procedure B for the synthesis of 1-bromovinylarenes.

To an oven dry two necked flask provided with a stir bar and a condenser, bromine (1.25 equiv., 0.64 mL, 12.5 mmol) was added to a stirring solution of triphenylphosphite (1.1 equiv., 3 mL, 11 mmol) in anhydrous dichloromethane (33 mL) at -60 ºC under Ar atmosphere. A pail orange solution was formed. Anhydrous triethylamine (1.35 equiv., 1.90 mL, 13.5 mmol) and 1-(p-tolyl)ethan-1-one (1.34 mL, 10 mmol) were also added. The resulting reaction mixture was stirred 18 h while warming to rt and then was refluxed employing an aluminium heating block for 2h. The reaction was cooled to rt, the solvent was removed under vacuum and the crude was purified by silica gel column chromatography with PE:EtOAc 95:5 to obtain 1-(1-bromovinyl)-4-methylbenzene as a pale yellow oil (928 mg, 4.71 mmol, 47 %).

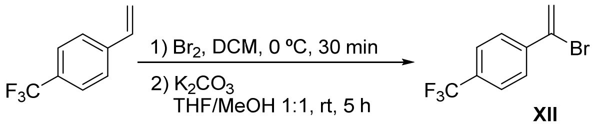

XII

Scheme S 5. General procedure C for the synthesis of 1-bromovinylarene **XII**.

General procedure C for the synthesis of 1-bromovinylarenes.

Bromine (1.2 equiv, 14 mmol, 0.72 mL) was slowly added, over a period of 5 minutes, to a solution of 1-(trifluoromethyl)-4-vinylbenzene (11.7 mmol, 2.1 g) in anhydrous dichloromethane (23.4 mL) at 0 ºC. The reaction mixture was stirred at this temperature for 30 minutes, controlling by TLC that the starting material was totally consumed. The reaction was quenched with a saturated solution of $Na_2S_2O_3$ (15 mL) and the product was extracted with dichloromethane (3x 30mL). The combined organic layers were washed with brine and dried over Na_2SO_4. After filtering the drying agent, the solvent was evaporated and the crude mixture was directly dissolved in THF/MeOH (1:1) (15 mL: 15 mL), followed by the addition of K_2CO_3 (2 equiv, 23.4 mmol, 3.2 g). The reaction mixture was stirred at room temperature for 5 h. The solvent was evaporated, and water was added (15 mL). The product was extracted with pentane (3 x 20 mL), those extracts were washed with brine and dried over Na_2SO_4. The drying agents was removed by filtration and the solvent was evaporated. The desired product was purified by silica gel column chromatography using petroleum ether as eluent to obtain 1-(1-bromovinyl)-4-(trifluoromethyl)benzene (8.3 mmol, 2.09 g) in a 72% isolated yield.

- **Synthesis of 3,3-disubstituted-1-(1-arylvinyl)cyclobutan-1-ols, 1a-1n.**

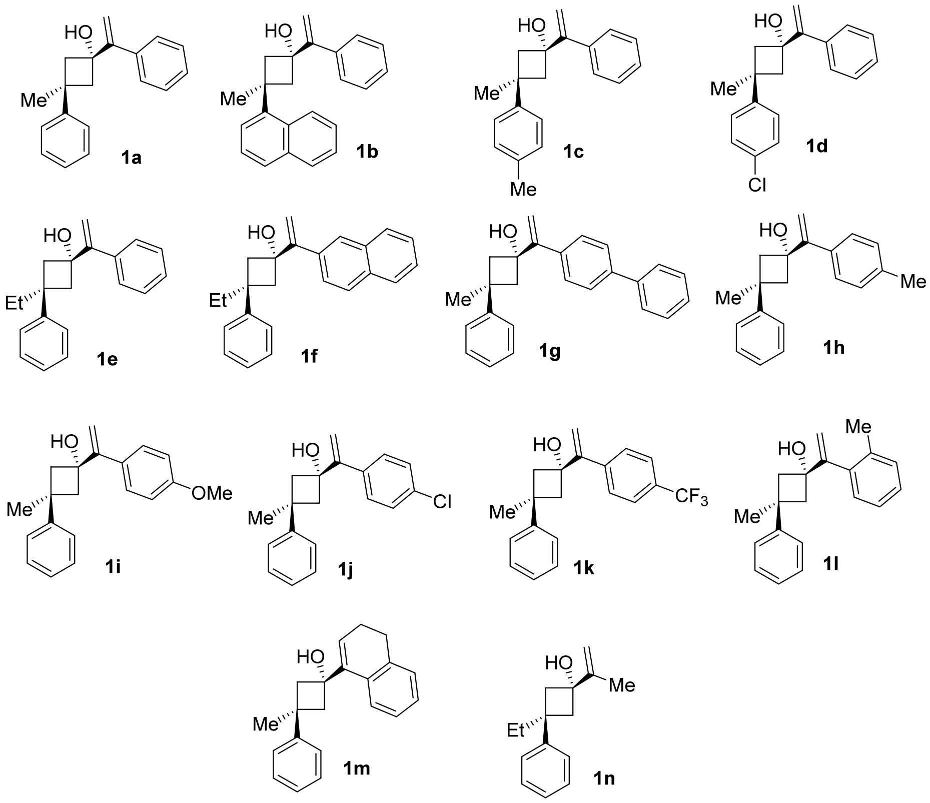

Figure S 4. Synthetized 3,3-disubstituted-1-(1-arylvinyl)cyclobutan-1-ol **1a**$_{trans}$-**1n**$_{trans}$.

Compounds **1a-1m** were prepared according to the general procedure D. Compound **1n** was prepared according to the described procedure E.[12]

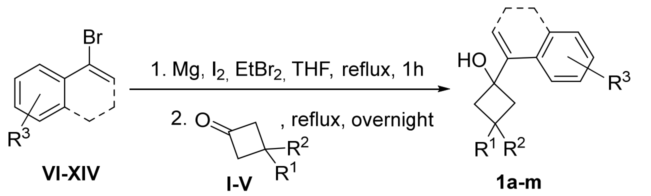

Scheme S 6. General procedure D for the synthesis of 3,3-disubstituted-1-(1-arylvinyl)ciclobutan-1-ol **1a-1m**.

<u>General procedure D for the synthesis of 3,3-disubstituted-1-(1-arylvinyl)ciclobutan-1-ols **1a-1m**.</u>

To an oven dried two necked flask provided with a condenser and stir bar, one crystal of iodine and 1,2-dibromoethane (0.4 equiv., 0.21 mL, 2.50 mmol) were added to a suspension of Mg (3 equiv., 450 mg, 18.75 mmol) in THF (20 mL) under Ar atmosphere and was heated to reflux employing an aluminium heating block for at least 10 mins.

[12] R. J. Phipps, L. McMurray, S. Ritter, H. A. Duong, M. J. Gaunt, *J. Am. Chem. Soc.* **2012**, *134*, 10773–10776.

Thereafter, a drop of the corresponding bromoalkene was added to the refluxing mixture in order to start the formation of organomagnesium compound. Once the bubbling starts, the rest of bromoalkene **VI-XIV** (1.3 equiv., 8.13 mmol) in THF (5 mL) was added dropwise keeping the bubbling and the reaction was refluxed employing an aluminium heating block for1 h. Then a solution of ketone **I-V** (6.25 mmol) in THF (5 mL) was added dropwise and the reaction mixture was refluxed overnight. After being cooled to room temperature it was quenched with saturated aq. NH_4Cl and extracted with Et_2O (3 x 20 mL), the combined organic layers were washed with brine, dried with Na_2SO_4, filtered and concentrate under vacuum. The crude was purified by silica gel column chromatography using PE:EtOAc (95:5 to 9:1) as eluent. Further purification with PE:DCM (1:1 to 3:7) was necessary to separate the diastereoisomers.

<u>Procedure E for the synthesis of 3-ethyl-3-phenyl-1-(prop-1-en-2-yl)cyclobutan-1-ol **1n**</u>

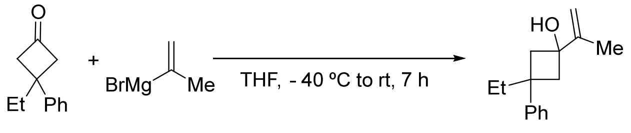

II   1n

Scheme S 7. Synthesis of 3,3-disubstituted-1-(prop-1-en-2-yl)ciclobutan-1-ol **1n**.

3-Ethyl-3-phenylcyclobutanone (300 mg, 1.72 mmol) was dissolved in 1.25 mL of anhydrous THF under Ar atmosphere. The solution was cooled to –40ºC and isopropenylmagnesium bromide (5.16 mL, 0.5 M in THF) was added to the solution drop by drop over 20 minutes. The reaction was allowed to stir at room temperature for 7 h. After this time, it was quenched with 2 mL of a saturated solution of NH_4Cl. The product was extracted from the aqueous phase with Et_2O (3 x 5 mL) and the combined organic phases were washed with brine, dried over Na_2SO_4 and concentrated *in vacuo*. The crude was purified by silica gel column chromatography using PE:DCM (70:30) as eluent to obtain *trans* diastereoisomer as a transparent oil (126 mg; 0.58 mmol; 34%) used in the next reaction and the *cis* one (78 mg; 0.36 mmol; 21 %).

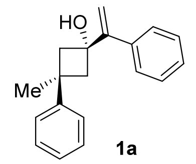

3-methyl-3-phenyl-1-(1-phenylvinyl)cyclobutan-1-ol **1a**

The product was prepared following the general procedure D, obtaining a mixture of *cis* and *trans* diastereoisomers (1.510 g, 5.69 mmol, 83%) with a dr$_{trans:cis}$ = 1.4:1. The *trans* isomer **1a** was isolated as a transparent oil (780 mg; 2.95 mmol; 43 %) by flash chromatography (PE:DCM = 1:1 to 3:7) starting from 1-bromovinylstyrene **VI** (1.06 mL; 8.13 mmol), Mg (450 mg; 18.75 mmol), THF (30.00 mL) and 3-methyl-3-phenylcyclobutanone **I** (1.000 g; 6.25 mmol). Rf = 0.4 (PE:DCM= 4:6). **^{1}H NMR** (500 MHz, $CDCl_3$) δ 7.37 (d, *J* = 7.1 Hz, 2H, C_{Arom}-H), 7.30 – 7.15 (m, 5H, C_{Arom}-H), 7.06 (t, *J* = 6.3 Hz, 3H, C_{Arom}-H), 5.21 – 5.17 (s, 1H, C=C**H**$_a$H$_b$), 5.13 (s, 1H, C=CH$_a$**H**$_b$), 2.79 (d, *J* = 13.3 Hz, 2H, C^1H_2), 2.42 (d, *J* = 13.4 Hz, 2H, C^2H_2), 1.59 (s, 3H, CH_3). **^{13}C{^{1}H} NMR** (75.5 MHz, $CDCl_3$) δ 153.7 (**C**=CH_2), 151.9 (C_{Arom}-C), 138.8 (C_{Arom}-C), 128.2 (2xC_{Arom}-H), 128.2 (2xC_{Arom}-H), 127.6 (C_{Arom}-H), 127.5 (2xC_{Arom}-H), 125.3 (C_{Arom}-H), 125.2 (2xC_{Arom}-H), 113.0 (C=**CH_2**), 74.0 (C-OH), 47.1 (2xCH_2), 35.8 (**C**-CH_2), 32.4 (CH_3). **IR** (ATR) cm^{-1}: 3648 (O-H), 3024 (=C-H), 2961 (C-H), 1625 (C=C), 1450 (CH_3). **MS** (EI) m/z (%): 263.2 (M^+, 34). **HRMS** (ESI^+): m/z calculated for $[C_{19}H_{20}O\ -H_2O+H]^+$ 247.1488; found 247.1487 $[M-H_2O+H]^+$.

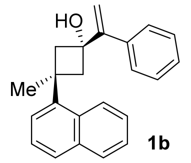

3-methyl-3-(naphthalen-2-yl)-1-(1-phenylvinyl)cyclobutan-1-ol **1b**

The product was prepared following the general procedure D, obtaining a mixture of *cis* and *trans* diastereoisomers (396 mg, 1.26 mmol, 88 %) with a dr$_{trans:cis}$ = 1.2:1. The *trans* isomer **1b** was isolated as a white solid (198 mg; 0.63 mmol; 44 %) by flash chromatography (PE:DCM = 4:6) starting from 1-bromovinylstyrene **VI** (0.24 mL; 1.86 mmol), Mg (103 mg; 4.29 mmol), THF (7 mL), and 3-methyl-3-(naphthalen-1-yl)cyclobutan-1-one **III** (300 mg; 1.43 mmol). Rf = 0.4 (PE:DCM = 4:6). **M.p.** = 115-118 ºC. **^{1}H NMR** (300 MHz, $CDCl_3$) δ 7.87 – 7.77 (m, 3H, C_{Arom}-H), 7.58 (d, *J* = 1.7 Hz, 1H, C_{Arom}-H), 7.55 – 7.44 (m, 4H, C_{Arom}-H), 7.36 (m, 4H, C_{Arom}-H), 5.29 (d, *J* = 0.7 Hz,

1H, C=C**H$_a$**H$_b$), 5.27 (d, *J* = 0.7 Hz, 1H, C=CH$_a$**H$_b$**), 3.03 (d, *J* = 13.4 Hz, 2H, C^1H_2), 2.63 (d, *J* = 13.3 Hz, 2H, C^2H_2), 2.10 (s, 1H, OH), 1.80 (s, 3H, CH_3). **^{13}C{^{1}H} NMR** (75.5 MHz, $CDCl_3$) δ 153.7 (**C**=CH_2), 149.0 (C_{Arom}-C), 138.9 (C_{Arom}-C), 133.3 (C_{Arom}-C), 131.6 (C_{Arom}-C), 128.3 (2xC_{Arom}-H), 128.0 (C_{Arom}-H), 127.7 (2xC_{Arom}-H), 127.5 (3xC_{Arom}-H), 126.0 (C_{Arom}-H), 125.3 (C_{Arom}-H), 124.5 (C_{Arom}-H), 122.9 (C_{Arom}-H), 113.1 (C=**CH_2**), 73.9 (C-OH), 47.2 (2xCH_2), 35.9 (**C**CH_2), 32.3 (CH_3). **IR** (ATR) cm^{-1}: 3547 (O-H), 3022 (=C-H), 2961 (C-H), 1625 (C=C), 1442 (CH_3). **MS** (EI) m/z (%): 168.1 (100), 153.1 (29), 128.1 (26), 103.1 (21). **HRMS** (ESI^+): m/z calculated for $[C_{23}H_{22}O + Na]^+$ 337.1568; found 337.1563 $[M+Na]^+$.

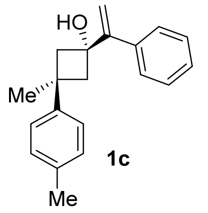

3-methyl-1-(1-phenylvinyl)-3-(p-tolyl)cyclobutan-1-ol **1c**.

The product was prepared following the general procedure D, obtaining a mixture of *cis* and *trans* diastereoisomers (416 mg, 1.49 mmol, 82%) with a $dr_{trans:cis}$ = 1.3:1. The *trans* isomer **1c** was isolated as a transparent oil (0.67 mmol; 188 mg; 39 %) by flash chromatography (PE:DCM = 6:4) starting from 1-bromovinylstyrene **VI** (0.29 mL; 2.24 mmol), Mg (125 mg; 5.16 mmol), THF (8.3 mL), and 3-methyl-3-(p-tolyl)cyclobutan-1-one **IV** (300 mg; 1.72 mmol). Rf = 0.20 (PE:DCM = 60:40). **^{1}H NMR** (300 MHz, $CDCl_3$) δ 7.49–7.42 (m, 2H, C_{Arom}-H), 7.36–7.27 (m, 3H, C_{Arom}-H), 7.10 (d, *J* = 8.2 Hz, 2H, C_{Arom}-H), 7.04 (d, *J* = 8.2 Hz, 2H, C_{Arom}-H), 5.25 (s, 1H, C=C**H$_a$**H$_b$), 5.21 (s, 1H, C=CH$_a$**H$_b$**), 2.85 (d, *J* = 13.5 Hz, 2H, C^1H_2), 2.48 (d, *J* = 13.5 Hz, 2H, C^2H_2), 2.31 (s, 3H, C_{Arom}-CH_3), 1.93 (s, 1H, OH), 1.66 (s, 3H, CH_3). **^{13}C{^{1}H} NMR** (75.5 MHz, $CDCl_3$) δ 153.7 (**C**=CH_2), 148.9 (C_{Arom}-C), 138.8 (C_{Arom}-C), 134.7 (**C_{Arom}**-CH_3), 128.8 (2xC_{Arom}-H), 128.2 (2xC_{Arom}-H), 127.6 (C_{Arom}-H), 127.4 (2xC_{Arom}-H), 125.0 (2xC_{Arom}-H), 112.9 (C=**CH_2**), 73.9 (C-OH), 47.2 (2xCH_2), 35.4 (**C-**CH_3), 32.4 (CH_3), 20.9 (C_{Arom}-**CH_3**). **IR** (ATR) cm^{-1}: 3427 (O-H), 2974 (C-H), 1626 (C=C). **MS** (EI) m/z (%): 132.1 (100), 165.0 (15), 245.1 (15), 277.9 (M^+, 2). **HRMS** (ESI^+): m/z calculated for $[C_{20}H_{22}O-H_2O+H]^+$ 261.1643; found 261.1635 $[M-H_2O+H]^+$.

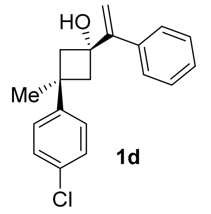

1d

3-(4-chlorophenyl)-3-methyl-1-(1-phenylvinyl)cyclobutan-1-ol **1d**

The product was prepared following the general procedure D, obtaining a mixture of *cis* and *trans* diastereoisomers (377 mg, 1.26 mmol, 35%) with a $dr_{trans:cis}$ = 1.7:1. The *trans* isomer **1d** was isolated as a transparent oil (0.16 mmol; 47.6 mg; 10 %) by flash chromatography (PE:DCM = 7:3) starting from 1-bromovinylstyrene **VI** (0.26 mL; 2.03 mmol), Mg (114 mg; 4.68 mmol), THF (7.5 mL), and 3-(4-chlorophenyl)-3-methylcyclobutan-1-one **V** (300 mg; 1.56 mmol). Rf = 0.19 (PE:DCM = 6:4). **^{1}H NMR** (300 MHz, $CDCl_3$) δ 7.39–7.33 (m, 2H, C_{Arom}-H), 7.27–7.19 (m, 3H, C_{Arom}-H), 7.18–7.12 (m, 2H, C_{Arom}-H), 7.01–6.94 (m, 2H, C_{Arom}-H), 5.18 (s, 1H, C=C$\mathbf{H_a}$$H_b$), 5.13 (s, 1H, C=C$H_a$$\mathbf{H_b}$), 2.74 (dd, *J* = 13.4, 2.6 Hz, 2H, C^1H_2), 2.41 (dd, *J* = 13.4, 2.6 Hz, 2H, C^2H_2), 1.80 (bs, 1H, OH), 1.56 (s, 3H, CH_3). **^{13}C{^{1}H} NMR** (75.5 MHz, $CDCl_3$) δ 153.5 (**C**=CH_2), 150.2 (C_{Arom}-C), 138.7 (C_{Arom}-C), 131.0 ($\mathbf{C_{Arom}}$-Cl), 128.2 (4xC_{Arom}-H), 127.7 (C_{Arom}-H), 127.4 (2xC_{Arom}-H), 126.7 (2xC_{Arom}-H), 113.1 (C=$\mathbf{CH_2}$), 73.7 (C-OH), 47.2 (2xCH_2), 35.5 (**C**-CH_3), 32.2 (CH_3). **IR** (ATR) cm^{-1}: 3423 (O-H), 2972 (C-H), 2926 (C-H), 1626 (C=C). **MS** (EI) m/z (%): 264.8 (6), 215 (13), 151.9 (93), 115.1 (96), 77 (100). **HRMS** (ESI^+): m/z calculated for $[C_{19}H_{19}ClO-H_2O+H]^+$ 281.1097; found 281.1094 $[M-H_2O+H]^+$.

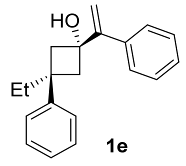

1e

3-ethyl-3-phenyl-1-(1-phenylvinyl)cyclobutan-1-ol **1e**

The product was prepared following the general procedure D, obtaining a mixture of *cis* and *trans* diastereoisomers (288.8 mg, 1.03 mmol, 91 %) with a $dr_{trans:cis}$ = 1:1. The *trans* isomer **1e** was isolated as a white solid (146 mg; 0.52 mmol; 46 %) by flash chromatography (PE:DCM = 4:6) starting from 1-bromovinylstyrene **VI** (0.20 mL; 1.49 mmol), Mg (83 mg; 3.45 mmol), THF (6 mL), and 3-ethyl-3-phenylcyclobutanone **II** (200 mg; 1.15 mmol). Rf = 0.4 (PE:DCM = 4:6). **M.p.** = 48 -50 ºC. **^{1}H NMR** (300 MHz, $CDCl_3$) δ 7.46 (m, 2H, C_{Arom}-H), 7.32 (m, 5H, C_{Arom}-H), 7.18 (m, 1H, C_{Arom}-H), 7.09 (m, 1H, C_{Arom}-H), 5.25 (d, *J* = 0.7 Hz, 1H, C=C$\mathbf{H_a}$$H_b$), 5.23 (d, J = 0.7 Hz, 1H, C=C$H_a$$\mathbf{H_b}$),

2.85 (dd, J = 11.0, 2.3 Hz, 2H, C^1H_2), 2.54 (dd, J = 11.0, 2.3 Hz, 2H, C^2H_2), 2.07 (q, J = 7.3 Hz, 2H, **CH_2**CH_3), 1.96 (s, 1H, OH), 0.68 (td, J = 7.3, 0.8 Hz, 2H, CH_2**CH_3**). **$^{13}C\{^1H\}$ NMR** (75.5 MHz, $CDCl_3$) δ 153.7 (**C**=CH_2), 148.9 (C_{Arom}-C), 138.9 (C_{Arom}-C), 128.2 (2xC_{Arom}-H), 127.7 (2xC_{Arom}-H), 127.6 (C_{Arom}-H), 127.5 (2xC_{Arom}-H), 126.3 (2xC_{Arom}-H), 125.2 (C_{Arom}-H), 113.1 (C=**CH_2**), 74.1 (C-OH), 45.7 (2xCH_2), 39.6 (**C**-CH_2), 36.7 (**CH_2**CH_3), 8.8 (CH_2**CH_3**). **IR** (ATR) cm^{-1}: 3553 (O-H), 3028 (=C-H), 2945 (C-H), 1620 (C=C), 1442 (CH_3). **MS** (EI) m/z (%): 146.1 (30), 132.1 (72), 117.1 (100). **HRMS** (ESI^+): m/z calculated for $[C_{20}H_{22}O-H_2O+H]^+$ 261.1643; found 261.1642 $[M-H_2O+H]^+$.

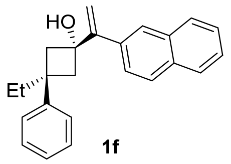

1f

3-ethyl-1-(1-(naphthalen-2-yl)vinyl)-3-phenylcyclobutan-1-ol **1f**

The product was prepared following the general procedure D, obtaining a mixture of *cis* and *trans* diastereoisomers (333 mg, 1.01 mmol, 59%) with a $dr_{trans:cis}$ = 1.2:1. The *trans* isomer **1f** was isolated as a white solid (0.53 mmol; 173 mg; 31 %) by flash chromatography (PE:DCM = 7:3 to 1:1) starting from 2-(1-bromovinyl)naphthalene **XI** (522 mg; 2.24 mmol), Mg (125 mg; 5.16 mmol), THF (11.2 mL), and 3-ethyl-3-phenylcyclobutanone **II** (300 mg; 1.72 mmol). Rf = 0.19 (PE:DCM = 6:4). M.p. = 75.3–76.1 ºC. **^{1}H NMR** (300 MHz, $CDCl_3$) δ 7.91–7.88 (m, 1H, C_{Arom}-H), 7.84–7.77 (m, 2H, C_{Arom}-H), 7.76 (s, 1H, C_{Arom}-H), 7.56 (dd, J = 8.5, 1.8 Hz, 1H, C_{Arom}-H), 7.50–7.42 (m, 2H, C_{Arom}-H), 7.29–7.20 (m, 3H, C_{Arom}-H), 7.17–7.10 (m, 1H, C_{Arom}-H), 7.08–7.02 (m, 2H, C_{Arom}-H), 5.36 (s, 1H, C=C**H_a**H_b), 5.30 (s, 1H, C=CH_a**H_b**), 2.86 (d, J = 13.5 Hz, 2H, C^1H_2), 2.57 (d, J = 13.4 Hz, 2H, C^2H_2), 2.06 (q, J = 7.4 Hz, 2H, **CH_2**CH_3) 2.01 (s, 1H, OH), 0.66 (t, 3H, J = 7.4 Hz, 3H, CH_3). **$^{13}C\{^1H\}$ NMR** (75.5 MHz, $CDCl_3$) δ 153.9 (**C**=CH_2), 149.0 (C_{Arom}-C), 136.5 (C_{Arom}-C), 133.5 (C_{Arom}-C), 133.0 (C_{Arom}-C), 128.5 (C_{Arom}-H), 128.0 (2xC_{Arom}-H), 127.9 (C_{Arom}-H), 127.8 (C_{Arom}-H), 126.6 (2xC_{Arom}-H), 126.3 (C_{Arom}-H), 126.2 (C_{Arom}-H), 126.1 (C_{Arom}-H), 125.5 (C_{Arom}-H), 113.9 (C=**CH_2**), 74.3 (C-OH), 46.0 (2xCH_2), 39.9 (**C**CH_2), 37.0 (C_{Arom}-**CH_2**-CH_3), 9.1 (C_{Arom}-CH_2-**CH_3**). **IR** (ATR) cm^{-1}: 3438 (O-H), 2964 (C-H), 2929 (C-H), 1598 (C=C). **MS** (EI) m/z (%): 328.2 (M, 12), 178.0 (43), 152 (100). **HRMS** (ESI^+): m/z calculated for $[C_{23}H_{22}O+Na]^+$ 337.1541; found 337.1553 $[M+Na-14]^+$.

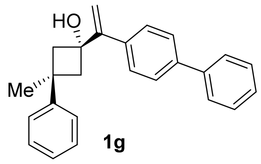

1g

1-(1-([1,1'-biphenyl]-4-yl)vinyl)-3-methyl-3-phenylcyclobutan-1-ol **1g**

The product was prepared following the general procedure D, obtaining a mixture of *cis* and *trans* diastereoisomers (301 mg, 0.88 mmol, 47%) with a $dr_{trans:cis}$ = 1.6:1. The *trans* isomer **1g** was isolated as a white solid (0.41 mmol; 141 mg; 22 %) by flash chromatography (PE:DCM = 6:4 to 1:1) starting from 4-(1-bromovinyl)-1,1'-biphenyl **X** (630 mg; 2.43 mmol), Mg (136 mg; 5.61 mmol), THF (8.9 mL), and 3-methyl-3-phenylcyclobutanone **I** (300 mg; 1.87 mmol). Rf = 0.28 (PE:DCM = 1:1). **M.p.** = 97.5-98.3 ºC. **^{1}H NMR** (300 MHz, $CDCl_3$) δ 7.55–7.49 (m, 2H, C_{Arom}-H), 7.46 (s, 4H, C_{Arom}-H), 7.40–7.32 (m, 2H, C_{Arom}-H), 7.30–7.16 (m, 3H, C_{Arom}-H), 7.11–7.03 (m, 3H, C_{Arom}-H), 5.24 (s, 1H, C=C$\mathbf{H_a}$$H_b$), 5.16 (s, 1H, C=C$H_a$$\mathbf{H_b}$), 2.82 (d, *J* = 13.4, 2.6 Hz, 2H, C^1H_2), 2.46 (d, *J* = 13.4, 2.6 Hz, 2H, C^2H_2), 1.90 (bs, 1H, OH), 1.61 (s, 3H, CH_3). **^{13}C{^{1}H} NMR** (75.5 MHz, $CDCl_3$) δ 153.2 (**C**=CH_2), 151.8 (C_{Arom}-C), 140.7 (C_{Arom}-C), 140.4 (C_{Arom}-C), 137.7 (C_{Arom}-C), 128.8 (2xC_{Arom}-H), 128.2 (2xC_{Arom}-H), 127.8 (2xC_{Arom}-H), 127.3 (C_{Arom}-H), 127.0 (2xC_{Arom}-H), 126.9 (2xC_{Arom}-H), 125.3 (C_{Arom}-H), 125.1 (2xC_{Arom}-H), 112.9 (C=$\mathbf{CH_2}$), 74.0 (C-OH), 47.2 (2xCH_2), 35.9 (**C**CH_3), 32.4 (C_{Arom}-$\mathbf{CH_3}$). **IR** (ATR) cm^{-1}: 3438 (O-H), 2971 (C-H), 2925 (C-H), 1601 (C=C). **MS** (EI) m/z (%): 340.1 (M, 18), 222.1 (48), 178.0 (100). **HRMS** (ESI^+): m/z calculated for $[C_{25}H_{24}O-H_2O+H]^+$ 323.1800; found 323.1790 $[M-H_2O+H]^+$.

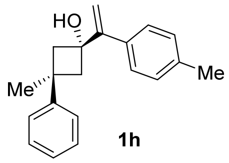

1h

3-methyl-3-phenyl-1-(1-(p-tolyl)vinyl)cyclobutan-1-ol **1h**

The product was prepared following the general procedure D, obtaining a mixture of *cis* and *trans* diastereoisomers (467.4 mg, 1.674 mmol, 75 %) with a $dr_{trans:cis}$ = 1.5:1. The *trans* isomer **1h** was isolated as a yellow oil (0.96 mmol; 268 mg; 43 %) by flash chromatography (PE:DCM = 1:1 to 3:7) starting from 1-(1-bromovinyl)-4-methylbenzene **VII** (424 mg; 2.154 mmol), Mg (163 mg; 6.81 mmol), THF (11.00 mL), and 3-methyl-3-phenylcyclobutanone **I** (363 mg; 2.27 mmol). Rf = 0.4 (PE:DCM =4:6). **^{1}H NMR** (300 MHz, $CDCl_3$) δ 7.42 (d, *J* = 8.2 Hz, 2H, C_{Arom}-H), 7.32 (q, *J* = 7.8 Hz, 2H, C_{Arom}-H), 7.23 – 7.15 (m, 5H, C_{Arom}-H), 5.29 (d, *J* = 0.5 Hz, 1H, C=C$\mathbf{H_a}$$H_b$), 5.22 (d, *J* =

0.5 Hz, 1H, C=CH$_a$**H$_b$**), 2.92 (dd, J = 10.8, 2.6 Hz, 2H, C^1H_2), 2.58 (dd, J = 10.8, 2.6 Hz, 2H, C^2H_2), 2.40 (s, 3H, C$_{Arom}$-CH$_3$), 2.04 (s, 1H, OH), 1.73 (s, 3H, CH$_3$). **^{13}C{^{1}H} NMR** (75.5 MHz, CDCl$_3$) δ 153.5 (**C**=CH$_2$), 151.9 (C$_{Arom}$-C), 137.4 (C$_{Arom}$-C), 135.9 (C$_{Arom}$-C), 128.9 (2xC$_{Arom}$-H), 128.2 (2xC$_{Arom}$-H), 127.4 (2xC$_{Arom}$-H), 125.3 (C$_{Arom}$-H), 125.2 (2xC$_{Arom}$-H), 112.3 (C=**CH$_2$**), 74.0 (C-OH), 47.2 (2xCH$_2$), 35.8 (**C**-CH$_3$), 32.4 (C$_{Arom}$-**CH$_3$**), 21.2 (C-**CH$_3$**). **IR** (ATR) cm^{-1}: 3482 (O-H), 3022 (=C-H), 2951 (C-H), 1630 (C=C), 1452 (CH$_3$). **MS** (EI) m/z (%): 278.2 (M$^+$, 23), 160.1 (57), 145 (100), 132 (44). **HRMS** (ESI$^+$): m/z calculated for $[C_{20}H_{22}O-H_2O+H]^+$ 261.1643; found 261.1642 $[M-H_2O+H]^+$.

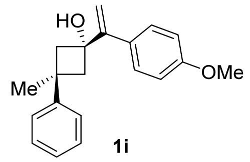

1i

1-(1-(4-methoxyphenyl)vinyl)-3-methyl-3-phenylcyclobutan-1-ol **1i**.

The product was prepared following the general procedure D, obtaining a mixture of *cis* and *trans* diastereoisomers (809 mg, 2.735 mmol, 88 %) with a dr$_{trans:cis}$ = 1.2:1. The *trans* isomer **1l** was isolated as a yellow solid (340 mg; 1.15 mmol; 37%) by flash chromatography (PE:DCM = 4:6) starting from 1-(1-bromovinyl)-4-methoxybenzene **VIII** (633 mg; 2.97 mmol), Mg (225 mg; 9.38 mmol), THF (15.00 mL), and 3-methyl-3-phenylcyclobutanone **I** (500 mg; 3.13 mmol). Rf = 0.4 (PE:DCM = 4:6). **M.p.** = 44-46 ºC. **^{1}H NMR** (300 MHz, CDCl$_3$) δ 7.33 (d, J = 8.9 Hz, 2H, 2xC$_{Arom}$-H), 7.18 (dd, J = 6.4, 1.0 Hz, 2H, 2xC$_{Arom}$-H), 7.10 – 7.02 (m, 3H, 3xC$_{Arom}$-H), 6.77 (d, J = 8.9 Hz, 2H, 2xC$_{Arom}$-H), 5.12 (d, J = 0.6 Hz, 1H, C=C**H$_a$**H$_b$), 5.05 (d, J = 0.4 Hz, 1H, C=CH$_a$**H$_b$**), 3.73 (s, 3H, OCH$_3$), 2.83 – 2.73 (m, 2H, C^1H_2), 2.47 – 2.36 (m, 2H, C^2H_2), 1.80 (bs, 1H, OH), 1.59 (s, 3H, CH$_3$). **^{13}C{^{1}H} NMR** (75.5 MHz, CDCl$_3$) δ 159.2 (**C**=CH$_2$), 153.0 (C$_{Arom}$-C), 152.0 (C$_{Arom}$-C), 131.1 (C$_{Arom}$-C), 128.6 (2xC$_{Arom}$-H), 128.2 (2xC$_{Arom}$-H), 125.2 (C$_{Arom}$-H), 125.1 (2xC$_{Arom}$-H), 113.6 (2xC$_{Arom}$-H), 111.6 (C=**CH$_2$**), 74.0 (C-OH), 55.3 (OCH$_3$), 47.2 (2xCH$_2$), 35.8 (**C**-CH$_3$), 32.4 (CH$_3$). **IR** (ATR) cm^{-1}: 3518 (O-H), 3021 (=C-H), 2949 (C-H), 1640 (C=C), 1448 (CH$_3$). **MS** (EI) m/z (%): 294.2 [M$^+$, 11], 295.2 [M$^+$, 3], 176.1 (98), 161.1 (24), 133.1 (100), 117.1 (63). **HRMS** (ESI$^+$): m/z calculated for $[C_{20}H_{22}O_2-H_2O+H]^+$ 277.1592; found 277.1594 $[M-H_2O+H]^+$.

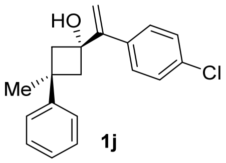

1j

1-(1-(4-chlorophenyl)vinyl)-3-methyl-3-phenylcyclobutan-1-ol **1j**

The product was prepared following the general procedure D, obtaining a mixture of *cis* and *trans* diastereoisomers (308 mg, 1.034 mmol, 33 %) with a $dr_{trans:cis}$ = 1.1:1. The *trans* isomer **1j** was isolated as a transparent oil (140 mg; 0.47 mmol; 15 %) by flash chromatography (PE:DCM = 4:6) starting from 1-(1-bromovinyl)-4-chlorobenzene **IX** (646 mg; 2.97 mmol), Mg (225 mg; 9.38 mmol), THF (15.00 mL), and 3-methyl-3-phenylcyclobutanone **I** (570 mg; 3.125 mmol). Rf = 0.4 (PE:DCM = 4:6). **^{1}H NMR** (300 MHz, $CDCl_3$) δ 7.44 (d, *J* = 8.7 Hz, 2H, C_{Arom}-H), 7.37 – 7.27 (m, 4H, C_{Arom}-H), 7.24 – 7.14 (m, 3H, C_{Arom}-H), 5.28 (s, 1H, C=C$\mathbf{H_a}$$H_b$), 5.26 (s, 1H, C=C$H_a$$\mathbf{H_b}$), 2.92 – 2.80 (m, 2H, C^1H_2), 2.60 – 2.44 (m, 2H, C^2H_2), 1.99 (s, 1H, OH), 1.70 (s, 3H, CH_3). **^{13}C{^{1}H} NMR** (75.5 MHz, $CDCl_3$) δ 152.7 (**C**=CH_2), 151.6 (C_{Arom}-C), 137.3 (C_{Arom}-C), 133.5 (C_{Arom}-C), 128.8 (2xC_{Arom}-H), 128.3 (2xC_{Arom}-H), 128.2 (2xC_{Arom}-H), 125.4 (C_{Arom}-H), 125.1 (2xC_{Arom}-H), 113.5 (C=$\mathbf{CH_2}$), 73.8 (C-OH), 47.0 (2xCH_2), 35.8 (**C**CH_3), 32.5 (CH_3). **IR** (ATR) cm^{-1}: 3567 (O-H), 3030 (=C-H), 2952 (C-H), 1643 (C=C), 1439 (CH_3). **MS** (EI) m/z (%): 145.1 (65), 137 (14), 118 (100). **HRMS** (ESI^+): m/z calculated for $[C_{19}H_{19}ClO-H_2O+H]^+$ 281.1097; found 281.1089 $[M-H_2O+H]^+$.

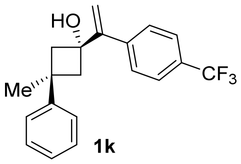

1k

3-methyl-3-phenyl-1-(1-(4-(trifluoromethyl)phenyl)vinyl)cyclobutan-1-ol **1k**

The product was prepared following the general procedure D, obtaining a mixture of *cis* and *trans* diastereoisomers (221 mg; 0.67 mmol; 36%) with a $dr_{trans:cis}$ = 3.3:1. The *trans* isomer **1k** was isolated as a yellow oil (156 mg; 0.47 mmol; 25 %) by flash chromatography (PE:DCM = 8:2 to 7:3 to 1:1) starting from 1-(1-bromovinyl)-4-(trifluoromethyl)benzene **XII** (610 mg; 2.43 mmol), Mg (136 mg; 5.61 mmol), THF (9 mL), and 3-methyl-3-phenylcyclobutan-1-one **I** (300 mg; 1.87 mmol). Rf = 0.25 (PE:DCM = 70:30). **^{1}H NMR** (300 MHz, $CDCl_3$) δ 7.48 (s, 4H, C_{Arom}-H), 7.24–7.15 (m, 2H, C_{Arom}-H), 7.10–7.00 (m, 3H, C_{Arom}-H), 5.22 (d, *J* = 1.4 Hz, 2H, C=CH_2), 2.75 (d, *J* = 13.4 Hz, 2H, C^1H_2), 2.41 (d, *J* = 13.4 Hz, 2H, C^2H_2), 1.87 (s, 1H, OH), 1.58 (s, 3H, CH_3). **^{19}F NMR** (282 MHz, $CDCl_3$) δ —62.5. **^{13}C{^{1}H} NMR** (75.5 MHz, $CDCl_3$) δ 152.7

(C=CH_2), 151.4 (C_{Arom}-C), 142.5 (C_{Arom}-C), 129.5 (q, J_{C-F} = 32.3 Hz, $\mathbf{C}_{Arom}$-CF_3), 128.2 (2xC_{Arom}-H), 127.7 (2xC_{Arom}-H), 125.4 (C_{Arom}-H), 125.1 (2xC_{Arom}-H), 125.0 (q, J_{C-F} = 3.8 Hz, 2xC_{Arom}-H), 124.0 (q, J_{C-F} = 272.0 Hz, CF_3), 114.6 (C=$\mathbf{CH_2}$), 73.7 (C-OH), 47.0 (2xCH_2), 35.8 (**C**CH_3), 32.5 (CH_3). **IR** (ATR) cm^{-1}: 3416 (O-H), 2953 (C-H), 2927 (C-H), 1616 (C=C). **MS** (EI) m/z (%): 314.1 (M-H_2O, 100). **HRMS** (ESI^+): m/z calculated for $[C_{20}H_{19}OF_3\text{-}H_2O+H]^+$ 315.1361; found 315.1353 $[M\text{-}H_2O+H]^+$.

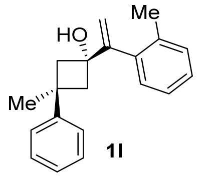

1l

3-methyl-3-phenyl-1-(1-(o-tolyl)vinyl)cyclobutan-1-ol **1l**

The product was prepared following the general procedure D, obtaining a mixture of *cis* and *trans* diastereoisomers (364 mg; 1.31 mmol; 70%) with a $dr_{trans:cis}$ = 1.7:1. The *trans* isomer **1l** was isolated as a transparent oil (0.46 mmol; 129 mg; 25 %) by flash chromatography (PE:DCM = 6:4 to 1:1) starting from 1-(1-bromovinyl)-2-methylbenzene **XIV** (479 mg; 2.43 mmol), Mg (136 mg; 5.61 mmol), THF (10 mL), and 3-methyl-3-phenylcyclobutanone **I** (300 mg; 1.87 mmol). Rf = 0.36 (PE:DCM = 60:40). **^{1}H NMR** (300 MHz, $CDCl_3$) δ 7.32–7.24 (m, 2H, C_{Arom}-H), 7.21–7.09 (m, 7H, C_{Arom}-H), 5.37 (d, J = 1.0 Hz, 1H, C=$C\mathbf{H_a}H_b$), 4.92 (d, J = 1.0 Hz, 1H, C=$CH_a\mathbf{H_b}$), 2.87 (dd, J = 13.4, 2.6 Hz, 2H, C^1H_2), 2.34 (dd, J = 13.4, 2.6 Hz, 2H, C^2H_2), 2.20 (s, 3H, C_{Arom}-CH_3), 1.82 (s, 1H, OH), 1.63 (s, 3H, CH_3). **^{13}C{^{1}H} NMR** (75.5 MHz, $CDCl_3$) δ 153.5 (**C**=CH_2), 151.7 (C_{Arom}-C), 139.4 (C_{Arom}-C), 136.2 (C_{Arom}-C), 130.3 (C_{Arom}-H), 128.8 (C_{Arom}-H), 128.2 (2xC_{Arom}-H), 127.3 (C_{Arom}-H), 125.3 (C_{Arom}-H), 125.2 (C_{Arom}-H), 125.1 (2xC_{Arom}-H), 113.8 (C=$\mathbf{CH_2}$), 74.2 (C-OH), 47.2 (2xCH_2), 35.6 (**C**-CH_3), 32.2 (C_{Arom}-$\mathbf{CH_3}$), 20.5 (CH_3). **IR** (ATR) cm^{-1}: 3416 (O-H), 2951 (C-H), 2924 (C-H), 1600 (C=C). **MS** (EI) m/z (%): 260 (M-H_2O, 6), 145 (23), 117 (100). **HRMS** (ESI^+): m/z calculated for $[C_{20}H_{22}O\text{-}H_2O+H]^+$ 261.1643; found 261.1635 $[M\text{-}H_2O+H]^+$.

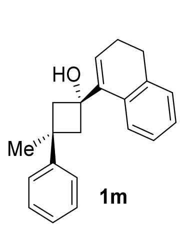

1m

1-(3,4-dihydronaphthalen-1-yl)-3-methyl-3-phenylcyclobutan-1-ol **1m**

The product was prepared following the general procedure D, obtaining a mixture of *cis* and *trans* diastereoisomers (364 mg, 1.245 mmol, 81 %) with a $dr_{trans:cis}$ = 1.4:1. The *trans* isomer **1m** was isolated as a transparent oil (184 mg; 0.63 mmol; 41 %) by flash chromatography (PE:DCM = 4:6) starting from 4-bromo-1,2-dihydronaphthalene **XIII** (306 mg; 1.47 mmol), Mg (111 mg; 4.64 mmol), THF (7.70 mL), and 3-methyl-3-phenylcyclobutanone **I** (250 mg; 1.55 mmol). Rf = 0.3 (PE:DCM = 1:1). **^{1}H NMR** (300 MHz, $CDCl_3$) δ 7.71 (d, *J* = 7.3 Hz, 1H, C_{Arom}-H), 7.48 – 7.34 (m, 2H, C_{Arom}-H), 7.32 – 7.19 (m, 6H, C_{Arom}-H), 6.10 (t, *J* = 4.7 Hz, 1H, C=**CH**), 3.15 – 3.04 (app dd, *J* = 13.3, 2.4 Hz , 2H, **C^1H_2**), 2.85 – 2.66 (m, 4H, **C^2H_2** and $C_{Arom}CH_2$), 2.38 – 2.15 (m, 2H, C_{Arom}-CH_2**CH_2** and -OH), 1.83 (s, 3H, CH_3). **^{13}C{^{1}H} NMR** (75.5 MHz, $CDCl_3$) δ 152.0 (C_{Arom}-C), 141.5 (**C**=CH), 137.4 (C_{Arom}-C), 132.3 (C_{Arom}-C), 128.2 (2xC_{Arom}-H), 127.9 (C_{Arom}-H), 126.9 (C_{Arom}-H), 126.2 (C_{Arom}-H), 125.6(C_{Arom}-H), 125.3 (2xC_{Arom}-H), 125.2 (C_{Arom}-H), 73.4 (C-OH), 47.6 (2x**CH_2**), 36.4 (**C**-CH_2), 32.7 (CH_3), 28.2 (CArom-CH_2), 23.0 (C_{Arom}-CH_2**CH_2**). **IR** (ATR) cm^{-1}: 3562 (O-H), 3022 (=C-H), 2954 (C-H), 1635 (C=C), 1437 (CH_3). **MS** (EI) m/z (%): 172.1 (90), 154.1 (41) 128 (100). **HRMS** (ESI^+): m/z calculated for $[C_{21}H_{22}O-H_2O+H]^+$ 273.1643; found 273.1644 $[M-H_2O+H]^+$.

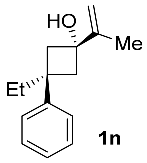

1n

3-ethyl-3-phenyl-1-(prop-1-en-2-yl)cyclobutan-1-ol **1n**

The product was prepared following the procedure E, obtaining a mixture of *cis* and *trans* diastereoisomers (242 mg; 1.12 mmol; 65%) with a $dr_{trans:cis}$ = 1.1:1. The *trans* isomer **1n** was obtained as a transparent oil (126 mg; 0.58 mmol; 34%) by flash chromatography (PE:DCM 7:3) starting from isopropenylmagnesium bromide (5.2 mL, 0.5 M in THF), THF (1.25 mL) and 3-ethyl-3-phenylcyclobutanone **II** (300 mg, 1.72 mmol). Rf = 0.29 (PE:DCM = 6:4). **^{1}H NMR** (300 MHz, $CDCl_3$) δ 7.33–7.24 (m, 2H, C_{Arom}-H), 7.19–7.09 (m, 3H, C_{Arom}-H), 4.84 (s, 1H, C=C**H_a**H_b), 4.73 (t, *J* = 1.4 Hz, 1H, C=CH_a**H_b**), 2.74 (d, *J* = 13.5 Hz, 2H, C^1H_2), 2.32 (d, *J* = 13.5 Hz, 2H, C^2H_2), 2.01 (q, *J* = 7.3 Hz, 2H, C**H_2**-CH_3), 1.70 (s, 3H, CH_3), 0.65 (t, *J* = 7.3 Hz, 3H, CH_2-C**H_3**). **^{13}C{^{1}H} NMR** (75.5 MHz, $CDCl_3$) δ 149.2 (C_{Arom}-**C**), 148.9 (**C**=CH_2), 127.7 (2xC_{Arom}-H), 126.3 (2xC_{Arom}-H), 125.2 (C_{Arom}-H), 109.7 (C=**CH_2**), 74.1 (C-OH), 44.2 (2xCH_2), 39.2 (**C**CH_2CH_3), 36.9 (**CH_2**-CH_3), 17.4 (CH_3), 8.7 (CH_2-**CH_3**). **IR** (ATR) cm^{-1}: 3364 (O-H), 2967 (C-H), 2929 (C-H),

1600 (C=C). **MS** (EI) m/z (%): 198 (M-H_2O, 10), 169 (30), 117 (100). **HRMS** (ESI^+): m/z calculated for $[C_{15}H_{20}O-H_2O+H]^+$ 199.1487; found 199.1485 $[M-H_2O+H]^+$.

Synthesis and characterization of products 3a-3n.

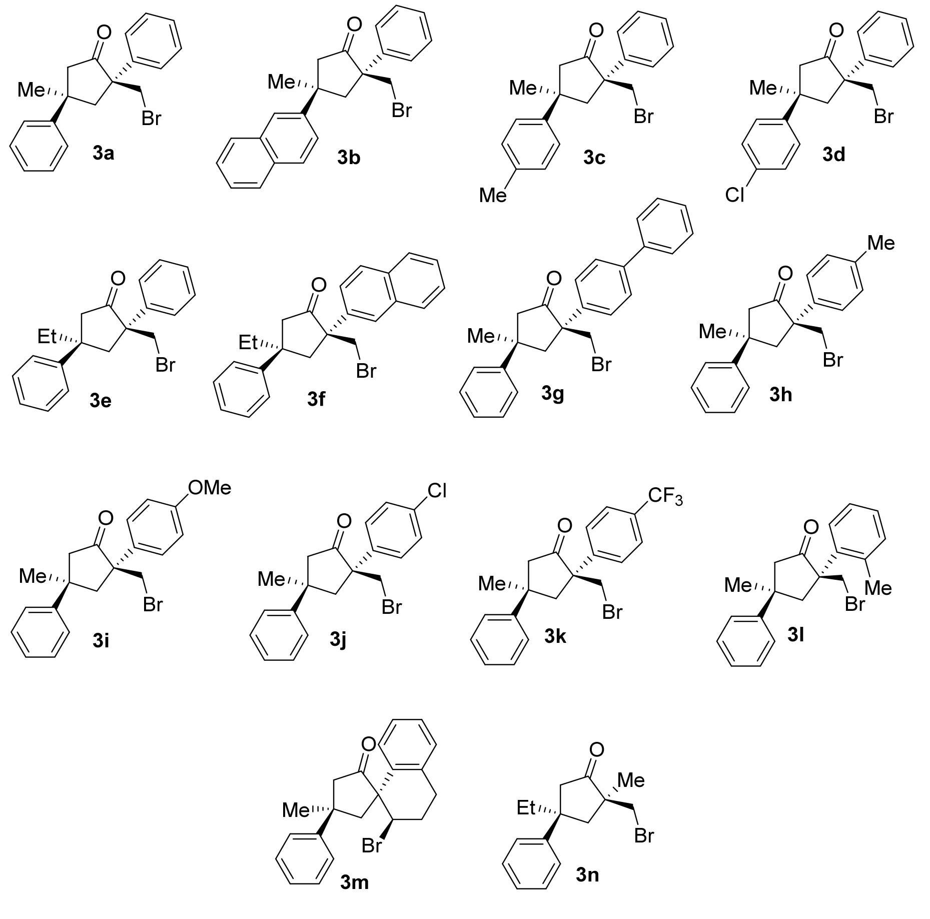

Figure S 5. Synthetized d 2,2,3,3-tetrasubstetutedcyclopentanones **3a-3n**.

All compounds were prepared according to the general procedure F.

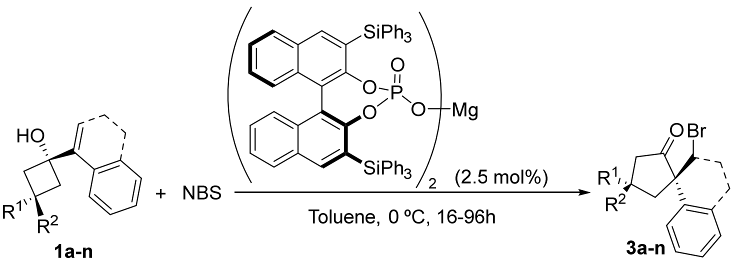

Scheme S 8. General procedure for the synthesis of 2,2,3,3-tetrasubstitutedcyclopentanones **3a-3n**.

General procedure F for the synthesis of 2,2,3,3-tetrasubstitutedcyclopentanones.

To a cold solution, 0 ºC, of (1*r*,3*r*)-3-methyl-3-phenyl-1-(1-phenylvinyl)cyclobutan-1-ol **1a** (26.4 mg, 0.1 mmol) and magnesium 2,6-bis(triphenylsilyl)dinaphtho[2,1-d:1',2'-f][1,3,2]dioxaphosphepin-4-olate 4-oxide **2b** (4.3 mg; 0.0025 mmol) in toluene (0.50 mL)

under Ar atmosphere, *N*-bromosuccinimide (1.1 equiv., 19.6 mg, 0.11 mmol) was added in one portion. The mixture was let stirring at 0 ºC for 16-96 h until full conversion by TLC. Once the reaction was finished, aq. std. $Na_2S_2O_3$ (1 mL) was added, the layers were separated and the aqueous layer was extracted with Et_2O (3 x 2 mL), dried with Na_2SO_4, filtered and concentrated under vacuum. The crude was purified by a short plug of silica gel column using PE:EtOAc 9:1.

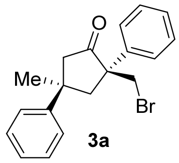

3a

(2*R*-4*R*)-2-(bromomethyl)-4-methyl-2,4-diphenylcyclopentan-1-one **3a**

Following the general procedure F, **3a** was isolated as a colorless oil (32.7 mg; 0.095 mmol; 95 %; dr>20:1; 92 %ee) by flash chromatography (PE:EtOAc 9:1), starting from 3-methyl-3-phenyl-1-(1-phenylvinyl)cyclobutan-1-ol (26.4 mg; 0.10 mmol) and **2b** (4.3 mg; 0.0025 mmol) in toluene (0.05 mmol) and NBS (19.6 mg; 0.11 mmol). The enantiomeric excess of product was determined by HPLC using Chiralpack OD-3 column [Hexane: *i*PrOH 99:1; flow = 1 mL/min; tr_{major} = 13.1 min.; tr_{minor} = 20.4 min.]. $[\alpha]_D^{25}$ = +21.1 (c = 0.5, $CHCl_3$). **Rf** = 0.36 (PE:EtOAc = 95:5) **^{1}H NMR** (300 MHz, $CDCl_3$) δ 7.61–7.55 (m, 2H, C_{Arom}-H), 7.46–7.22 (m, 8H, C_{Arom}-H), 3.64 (d, *J* = 10.1 Hz, 1H, C$\mathbf{H_a}$$H_b$Br), 3.47 (d, *J* = 10.1 Hz, 1H, C$H_a$$\mathbf{H_b}$Br), 3.09 (d, *J* = 13.6 Hz, 1H, $C^1\mathbf{H_a}H_b$), 3.03 (dd, *J* = 13.6, 1.7 Hz, 1H, $C^1H_a\mathbf{H_b}$), 2.90 (d, *J* = 18.3 Hz, 1H, $C^2\mathbf{H_a}H_b$), 2.73 (dd, *J* = 18.3, 1.7 Hz, 1H, $C^2H_a\mathbf{H_b}$), 1.12 (s, 3H, CH_3). **^{13}C{^{1}H} NMR** (75.5 MHz, $CDCl_3$) δ 216.2 (C=O), 149.0 (C_{Arom}-C), 140.1 (C_{Arom}-C), 128.9 (2xC_{Arom}-H), 128.7 (2xC_{Arom}-H), 127.8 (C_{Arom}-H), 126.6 (2xC_{Arom}-H), 126.4 (C_{Arom}-H), 125.3 (2xC_{Arom}-H), 57.9 (**C**-CH_2Br), 52.9 (C^1H_2), 45.6 (C^2H_2), 41.1 (**C**CH_2), 40.9 (CH_2Br), 31.2 (CH_3). **IR** (ATR) cm^{-1}: 3020 (=C-H), 2958 (C-H), 1739 (C=O), 1643 (C=C). **MS** (EI) e/z (%): 263.2 (34), 205.1 (25), 145.1 (39), 117.1 (100). **HRMS** (ESI^+): m/z calculated for $[C_{19}H_{19}OBr+Na]^+$ 365.0517; found 365.0507 $[M+Na]^+$.

Bigger scale: Following the general procedure F, **3a** was isolated as a colorless oil (336.2 mg; 0.98 mmol; 98 %; dr>20:1; 92 %ee) starting from 3-methyl-3-phenyl-1-(1-phenylvinyl)cyclobutan-1-ol (264 mg; 1.00 mmol) and **2b** (43 mg; 0.025 mmol) in toluene (0.5 mmol) and NBS (196 mg; 1.10 mmol).

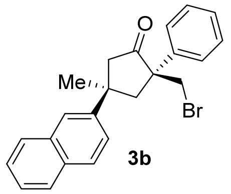

(2R,4R)-2-(bromomethyl)-4-methyl-4-(naphthalen-1-yl)-2-phenylcyclopentan-1-one **3b**

Following the general procedure F, **3b** was isolated as a white solid (35.4 mg; 0.090 mmol; 90 %; dr>20:1; 92 %ee) by flash chromatography (PE:EtOAc 95:5), starting from 3-methyl-3-(naphthalen-1-yl)-1-(1-phenylvinyl)cyclobutan-1-ol **1b** (31.4 mg; 0.10 mmol) and **2b** (4.3 mg; 0.0025 mmol) in toluene (0.50 mL) and NBS (19.6 mg; .0.11 mmol). The enantiomeric excess of product was determined by HPLC using Chiralpack OD-3 column [Hexane: *i*PrOH 99:1; flow = 1 mL/min.; tr_{major} = 12.1 min.; tr_{minor} = 14.4 min.]. **[α]**$_D^{24}$ = +27.6 (c = 0.4, $CHCl_3$). **Rf** = 0.43 (PE:EtOAc = 95:5). **M.p.** = 115-118 ºC. **^{1}H NMR** (300 MHz, $CDCl_3$) δ 7.92 – 7.81 (m, 3H, C_{Arom}-H), 7.76 (d, J = 1.8 Hz, 1H, C_{Arom}-H), 7.65 – 7.58 (m, 2H, C_{Arom}-H), 7.58 – 7.47 (m, 3H, C_{Arom}-H), 7.46 – 7.37 (m, 2H, C_{Arom}-H), 7.34 (dt, J = 5.0, 2.0 Hz, 1H, C_{Arom}-H), 3.65 (d, J = 10.1 Hz, 1H, C$\mathbf{H_a}$$H_b$Br), 3.49 (d, J = 10.1 Hz, 1H, C$H_a$$\mathbf{H_b}$Br), 3.21 (d, J = 13.7 Hz, 1H, $C^1$$\mathbf{H_a}$$H_b$), 3.12 (dd, J = 13.7, 1.9 Hz, 1H, $C^1$$H_a$$\mathbf{H_b}$), 3.02 (d, J = 18.3 Hz, 1H, $C^2$$\mathbf{H_a}$$H_b$), 2.83 (dd, J = 18.3, 1.9 Hz, 1H, $C^2$$H_a$$\mathbf{H_b}$), 1.22 (s, 3H, CH_3). **^{13}C{^{1}H} NMR** (75.5 MHz, $CDCl_3$) δ 216.07 (C=O), 146.18 (C_{Arom}-C), 140.13 (C_{Arom}-C), 133.30 (C_{Arom}-C), 132.05 (C_{Arom}-C), 128.94 (2xC_{Arom}-H), 128.56 (C_{Arom}-H), 127.92 (C_{Arom}-H), 127.78 (C_{Arom}-H), 127.51 (C_{Arom}-H), 126.66 (2x C_{Arom}-H), 126.33 (C_{Arom}-H), 125.84 (C_{Arom}-H), 124.31 (C_{Arom}-H), 123.30 (C_{Arom}-H), 58.03 (**C**CH_2Br), 52.96 (C^1H_2), 45.52 (C^2H_2), 41.20 (CH_2), 40.90 (CH_2Br), 31.07 (CH_3). **IR** (ATR) cm^{-1}: 3022 (=C-H), 2956 (C-H), 1739 (C=O), 1456 (CH_2), 1446 (CH_3). **MS** (EI) m/z (%): 392.1 (M^+, 4), 195.1 (55), 168.1 (88), 152.1 (75), 128.1 (59), 117.1 (100). **HRMS** (ESI^+): m/z calculated for $[C_{23}H_{21}OBr+Na]^+$ 415.0673; found 415.0669 $[M+Na]^+$.

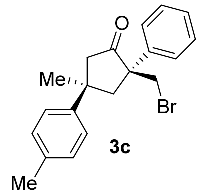

(2R,4R)-2-(bromomethyl)-4-methyl-2-phenyl-4-(p-tolyl)cyclopentan-1-one **3c**

Following the general procedure F, **3c** was isolated as a yellowish oil (31 mg; 0.09 mmol; 87 %; dr>20:1; 92 %ee) by flash chromatography (PE:EtOAc = 95:5), starting from 3-methyl-1-(1-phenylvinyl)-3-(p-tolyl)cyclobutan-1-ol **1c** (27.8 mg; 0.10 mmol) and **2b**

(4.3 mg; 0.0025 mmol) in toluene (0.5 mL) and NBS (19.6 mg; 0.11 mmol). The enantiomeric excess of product was determined by HPLC using Chiralpack OD3 column [Hexane: *i*PrOH 99:1; flow = 1 mL/min; tr_{minor} = 9.9 min.; tr_{major} = 11.6 min.]. **[α]**$_D^{20}$= +30.2 (*c*=1, $CHCl_3$). **Rf** = 0.59 (PE:EtAcO = 95:5). **^{1}H NMR** (300 MHz, $CDCl_3$) δ 7.52–7.46 (m, 2H, C_{Arom}-H), 7.34–7.26 (m, 2H, C_{Arom}-H), 7.26–7.21 (m, 1H, C_{Arom}-H), 7.21–7.14 (m, 2H, C_{Arom}-H), 7.14–7.07 (m, 2H, C_{Arom}-H), 3.54 (d, *J* = 10.1 Hz, 1H, C$\mathbf{H_a}$$H_b$Br), 3.37 (d, *J* = 10.1 Hz, 1H, C$H_a$$\mathbf{H_b}$Br), 2.98 (d, *J* = 13.6 Hz, 1H, $C^1$$\mathbf{H_a}$$H_b$), 2.91 (dd, *J* = 13.6, 1.7 Hz, 1H, $C^1$$H_a$$\mathbf{H_b}$), 2.79 (d, *J* = 18.2 Hz, 1H, $C^2$$\mathbf{H_a}$$H_b$), 2.62 (dd, *J* = 18.2 Hz, 1H, $C^2$$H_a$$\mathbf{H_b}$), 2.27 (s, 3H, CH_3-C_{Ar}), 1.03 (s, 3H, CH_3). **^{13}C{^{1}H} NMR** (75.5 MHz, $CDCl_3$) δ 216.3 (C=O), 146.0 (C_{Arom}), 140.2 (C_{Arom}), 136.0 (C_{Arom}), 129.3 (2xC_{Arom}-H), 128.9 (2xC_{Arom}-H), 127.7 (C_{Arom}-H), 126.6 (2xC_{Arom}-H), 125.2 (2xC_{Arom}-H), 58.0 (**C**-CH_2Br), 53.1 (C^1H_2), 45.6 (C^2H_2), 40.9 (CH_2Br), 40.7 (**C**CH_3), 31.3 (**C**CH_3), 20.9 (CH_3). **IR** (ATR) cm^{-1}: 1739 (C=O). **MS** (EI) e/z (%): 277.1 (12), 132 (43), 115 (100). **HRMS** (ESI^+): m/z calculated for $[C_{20}H_{21}OBr+Na]^+$ 379.0673; found 379.0672 $[M+Na]^+$.

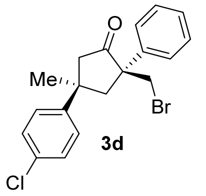

3d

(2*R*,4*R*)-2-(bromomethyl)-4-(4-chlorophenyl)-4-methyl-2-phenylcyclopentan-1-one **3d**

Following the general procedure F, **3d** was isolated as a yellowish solid (35 mg; 0.09 mmol; 92 %; dr>20:1; 94 %ee) by FC (PE:EtOAc = 95:5), starting from (1*r*,3*r*)-3-(4-chlorophenyl)-3-methyl-1-(1-phenylvinyl)cyclobutan-1-ol **3d** (29.9 mg; 0.10 mmol) and **2b** (4.3 mg; 0.0025 mmol) in toluene (0.5 mL) and NBS (19.6 mg; 0.11 mmol). The enantiomeric excess of product was determined by HPLC using Chiralpack OD3 column [Hexane: *i*PrOH 99:1; flow = 1 mL/min; tr_{minor} = 14.9 min.; tr_{major} = 15.9 min.]. **[α]**$_D^{20}$= +23.2 (*c*=1, $CHCl_3$). **Rf** = 0.56 (PE:EtAcO = 95:5). **M.p.** = 97.3–98.6. **^{1}H NMR** (300 MHz, $CDCl_3$) *δ* 7.59–7.52 (m, 2H, C_{Arom}-H), 7.43–7.25 (m, 7H, C_{Arom}-H), 3.65 (d, *J* = 10.1 Hz, 1H, C$\mathbf{H_a}$$H_b$Br), 3.45 (d, *J* = 10.1 Hz, 1H, C$H_a$$\mathbf{H_b}$Br), 3.05 (d, *J* = 13.6 Hz, 1H, $C^1$$\mathbf{H_a}$$H_b$), 2.99 (d, *J* = 13.6 Hz, 1H, $C^1$$H_a$$\mathbf{H_b}$), 2.83 (d, *J* = 18.2 Hz, 1H, $C^2$$\mathbf{H_a}$$H_b$), 2.70 (dd, *J* = 18.2, 1.4 Hz, 1H, $C^2$$H_a$$\mathbf{H_b}$), 1.09 (s, 3H, CH_3). **^{13}C{^{1}H} NMR** (75.5 MHz, $CDCl_3$) *δ* 215.7 (C=O), 147.6 (C_{Arom}-C), 139.9 (C_{Arom}-C), 132.2 (C_{Arom}-C), 129.0 (2xC_{Arom}-H), 128.8 (2xC_{Arom}-H), 127.9 (C_{Arom}-H), 126.8 (2xC_{Arom}-H), 126.6 (2xC_{Arom}-H), 58.0 (**C**-CH_2Br), 52.9 (C^1H_2), 45.5 (C^2H_2), 40.8 (**C**-CH_3), 40.7 (CH_2Br), 31.1 (CH_3). **IR** (ATR)

cm^{-1}: 1741 (C=O), 1096 (C_{Ar}-Cl). **MS** (EI) e/z (%): 297.1 (6), 152 (41), 115 (100). **HRMS** (ESI^+): m/z calculated for $[C_{19}H_{18}OClBr+Na]^+$ 399.0127; found 399.0116 $[M+Na]^+$.

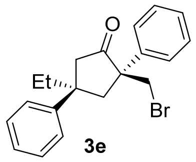

3e

(2*R*,4*R*)-2-(bromomethyl)-4-ethyl-2,4-diphenylcyclopentan-1-one **3e**

Following the general procedure F, **3e** was isolated as a colorless oil (30 mg; 0.084 mmol; 84 %; dr>20:1; 93 %ee) starting from 3-ethyl-3-phenyl-1-(1-phenylvinyl)cyclobutan-1-ol **1e** (27.8 mg; 0.10 mmol) and **2b** (4.3 mg; 0.0025 mmol) in toluene (0.50 mL) and NBS (19.6 mg; 0.11 mmol). The enantiomeric excess of product was determined by HPLC using Chiralpack OD-3 column [Hexane: *i*PrOH 99:1; flow = 1 mL/min.; tr_{minor} = 11.7 min.; tr_{major} = 18.0 min.]. $[\alpha]_D^{24}$ = +5.2 (c = 0.5, $CHCl_3$). **Rf** = 0.48 (PE:EtOAc = 95:5). **^{1}H NMR** (300 MHz, $CDCl_3$) δ 7.61 – 7.54 (m, 2H, C_{Arom}-H), 7.37 (m, 5H, C_{Arom}-H), 7.27 (m, 3H, C_{Arom}-H), 3.50 (d, *J* = 10.2 Hz, 1H, C$\mathbf{H_a}$$H_b$Br), 3.44 (d, *J* = 10.2 Hz, 1H, C$H_a$$\mathbf{H_b}$Br) 3.10 (dd, *J* = 13.7, 1.7 Hz, 1H, $C^1\mathbf{H_a}H_b$), 3.00 (d, *J* = 13.7 Hz, 1H, $C^1H_a\mathbf{H_b}$), 2.90 (d, *J* = 18.1 Hz, 1H, $C^2\mathbf{H_a}H_b$), 2.78 (dd, *J* = 18.0, 1.7 Hz, 1H, $C^2H_a\mathbf{H_b}$), 1.54 – 1.35 (m, 2H, $\mathbf{CH_2}CH_3$), 0.46 (t, *J* = 7.3 Hz, 3H, $CH_2\mathbf{CH_3}$). **^{13}C{^{1}H} NMR** (75.5 MHz, $CDCl_3$) δ 215.9 (C=O), 146.1 (C_{Arom}-C), 140.0 (C_{Arom}-C), 128.8 (2xC_{Arom}-H), 128.4 (2xC_{Arom}-H), 127.7 (C_{Arom}-H), 126.6 (2xC_{Arom}-H), 126.4 (C_{Arom}-H), 126.4 (2xC_{Arom}), 57.3 (**C**CH_2Br), 51.1 (C^1H_2), 45.1 (**C**CH_3), 42.9 (C^2H_2), 41.1 (CH_2Br), 35.4 ($\mathbf{CH_2}CH_3$), 8.9 ($CH_2\mathbf{CH_3}$). **IR** (ATR) cm^{-1}: 3022 (=C-H), 2962 (C-H), 1738 (C=O), 1636 (C=C), 1465 (CH_2), 1446 (CH_3). **MS** (EI) m/z (%): 277.2 (22), 235.2 (16), 159.1 (20), 131.1 (42), 117.1 (100). **HRMS** (ESI^+): m/z calculated for $[C_{20}H_{21}OBrNa]^+$ 379.0673; found 379.0670 $[M+Na]^+$.

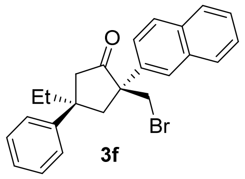

3f

(2*R*,4*R*)-2-(bromomethyl)-4-ethyl-2-(naphthalen-2-yl)-4-phenylcyclopentan-1-one **3f**

Following the general procedure F, **3f** was isolated as a yellowish oil (31 mg; 0.08 mmol; 76 %; dr>20:1; 92 %ee) by FC (PE:EtOAc = 95:5), starting from 3-ethyl-1-(1-(naphthalen-2-yl)vinyl)-3-phenylcyclobutan-1-ol **1f** (32.8 mg; 0.10 mmol) and **2b** (4.3 mg; 0.0025 mmol) in toluene (0.5 mL) and NBS (19.6 mg; 0.11 mmol). The enantiomeric

excess of product was determined by HPLC using Chiralpack IA column [Hexane: *i*PrOH 99:1; flow = 1 mL/min; tr_{minor} = 11.4 min.; tr_{major} = 14.4 min.]. **[α]**$_D^{20}$= +35.1 (*c*=1, $CHCl_3$). **Rf** = 0.47 (PE:EtOAc = 95:5). **^{1}H NMR** (300 MHz, $CDCl_3$) δ 7.94 (d, *J* = 1.8 Hz, 1H, C_{Arom}-H), 7.83–7.72 (m, 3H, C_{Arom}-H), 7.61 (dd, *J* = 8.7, 2.0 Hz, 1H, C_{Arom}-H), 7.46–7.38 (m, 2H, C_{Arom}-H), 7.34–7.26 (m, 2H, C_{Arom}-H), 7.24–7.14 (m, 3H, C_{Arom}-H), 3.50 (d, *J* = 10.2 Hz, 1H, C$\mathbf{H_a}$$H_b$Br), 3.45 (d, *J* = 10.2 Hz, 1H, C$H_a$$\mathbf{H_b}$Br), 3.16 (dd, *J* = 13.7, 1.7 Hz, 1H, $C^1$$\mathbf{H_a}$$H_b$), 2.99 (d, *J* = 13.7 Hz, 1H, $C^1$$H_a$$\mathbf{H_b}$), 2.85 (d, *J* = 18.1 Hz, 1H, $C^2$$\mathbf{H_a}$$H_b$), 2.75 (dd, *J* = 18.1, 1.7 Hz, 1H, $C^2$$H_a$$\mathbf{H_b}$), 1.40 (dq, *J* = 13.9, 7.3 Hz, 1H, C$\mathbf{H_a}$$H_b$$CH_3$), 1.30 (dq, *J* = 13.9, 7.3 Hz, 1H, C$H_a$$\mathbf{H_b}$$CH_3$), 0.35 (t, *J* = 7.3 Hz, 3H, CH_3). **^{13}C{^{1}H} NMR** (75.5 MHz, $CDCl_3$) δ 216.0 (C=O), 146.2 (C_{Arom}-C), 137.3 (C_{Arom}-C), 133.2 (C_{Arom}-C), 132.5 (C_{Arom}-C), 128.6 (C_{Arom}-H), 128.4 (2xC_{Arom}-H), 128.3 (C_{Arom}-H), 127.5 (C_{Arom}-H), 126.5 (C_{Arom}-H), 126.4 (2xC_{Arom}-H), 126.4 (2xC_{Arom}-H), 125.8 (C_{Arom}-H), 124.3 (C_{Arom}-H), 57.4 (**C**-CH_2Br), 51.1 (C^1H_2), 45.2 (**C**-CH_2CH_3), 43.0 (C^2H_2), 40.9 (CH_2Br), 35.4 (**CH_2**CH_3), 8.9 (CH_2**CH_3**). **IR** (ATR) cm^{-1}: 1736 (C=O). **MS** (EI) e/z (%): 408.1 (M+H, 7), 195.1 (45), 165 (100). **HRMS** (ESI^+): m/z calculated for $[C_{24}H_{23}OBr+Na]^+$ 429.0830; found 429.0825 $[M+Na]^+$.

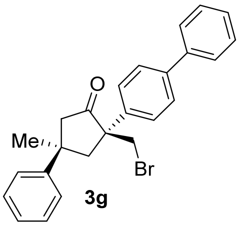

3g

(2*R*,4*R*)-2-([1,1'-biphenyl]-4-yl)-2-(bromomethyl)-4-methyl-4-phenylcyclopentan-1-one **3g**

Following the general procedure F, **3g** was isolated as a amorphous white solid (41 mg; 0.10 mmol; 98 %; dr>20:1; 92% ee) by FC (PE:EtOAc = 95:5), starting from 1-(1-([1,1'-biphenyl]-4-yl)vinyl)-3-methyl-3-phenylcyclobutan-1-ol **1g** (34 mg; 0.10 mmol) and **2b** (4.3 mg; 0.0025 mmol) in toluene (0.5 mL) and NBS (19.6 mg; 0.11 mmol). The enantiomeric excess of product was determined by HPLC using Chiralpack IA column [Hexane: *i*PrOH 99:1; flow = 1 mL/min; tr_{minor} = 17.7 min.; tr_{major} = 35.4 min.]. **[α]**$_D^{20}$= +44.9 (*c*=1, $CHCl_3$). **Rf** = 0.37 (PE:EtAcO = 95:5). **^{1}H NMR** (300 MHz, $CDCl_3$) *δ* 7.71–7.58 (m, 6H, C_{Arom}-H), 7.52–7.30 (m, 8H, C_{Arom}-H), 3.68 (d, *J* = 10.2 Hz, 1H, C$\mathbf{H_a}$$H_b$Br), 3.51 (d, *J* = 10.2 Hz, 1H, C$H_a$$\mathbf{H_b}$Br), 3.13 (d, *J* = 13.8 Hz, 1H, $C^1$$\mathbf{H_a}$$H_b$), 3.07 (dd, *J* = 13.8 Hz, 1H, $C^1$$H_a$$\mathbf{H_b}$), 2.93 (d, *J* = 18.2 Hz, 1H, $C^2$$\mathbf{H_a}$$H_b$), 2.76 (dd, *J* = 18.2 Hz, 1H, $C^2$$H_a$$\mathbf{H_b}$), 1.18 (s, 3H, CH_3). **^{13}C{^{1}H} NMR** (75.5 MHz, $CDCl_3$) *δ* 216.2 (C=O), 149.0

(C_{Arom}-C), 140.5 (C_{Arom}-C), 140.2 (C_{Arom}-C), 139.1 (C_{Arom}-C), 128.8 (2xC_{Arom}-H), 128.7 (2xC_{Arom}-H), 127.6 (C_{Arom}-H), 127.5 (2xC_{Arom}-H), 127.1 (2xC_{Arom}-H), 127.0 (2xC_{Arom}-H), 126.5 (C_{Arom}-H), 125.4 (2xC_{Arom}-H), 57.7 (**C**-CH_2Br), 53.0 (C^1H_2), 45.5 (C^2H_2), 41.1 (**C**CH_3), 40.9 (CH_2Br), 31.4 (CH_3) **IR** (ATR) cm^{-1}: 1738 (C=O). **MS** (EI) e/z (%): 418.1 (M, 3), 338.1 (5), 221 (11), 178 (100). **HRMS** (ESI^+): m/z calculated for $[C_{25}H_{23}OBr+Na]^+$ 441.0830; found 441.0830 $[M+Na]^+$.

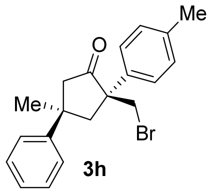

3h

(2*R*,4*R*)-2-(bromomethyl)-4-methyl-4-phenyl-2-(*p*-tolyl)cyclopentan-1-one **3h**

Following the general procedure F, **3h** was isolated as a colorless oil (28.2 mg; 0.079 mmol; 79 %; dr>20:1; 93 %ee) starting from 3-methyl-3-phenyl-1-(1-(p-tolyl)vinyl)cyclobutan-1-ol **1h** (27.8 mg; 0.10 mmol) and **2b** (4.3 mg; 0.0025 mmol) in Toluene (0.50 mL) and NBS (19.6 mg; 0.11 mmol). The enantiomeric excess of product was determined by HPLC using Chiralpack OD-3 column [Hexane: *i*PrOH 99:1; flow = 1 mL/min.; tr_{major} = 12.0 min.; tr_{minor} = 16.8 min.]. $[\alpha]_D^{25}$ = +11.9 (c = 0.7, $CHCl_3$). **Rf** = 0.39 (PE:EtOAc = 95:5). **^{1}H NMR** (300 MHz, $CDCl_3$) δ 7.45 (d, *J* = 8.3 Hz, 2H, C_{Arom}-H), 7.43–7.33 (m, 4H, C_{Arom}-H), 7.31–7.23 (m, 1H, C_{Arom}-H), 7.20 (d, *J* = 8.0 Hz, 2H, C_{Arom}-H), 3.63 (d, *J* = 10.1 Hz, 1H, C$\mathbf{H_a}H_b$Br), 3.45 (d, *J* = 10.1 Hz, 1H, C$H_a\mathbf{H_b}$Br), 3.07 (d, *J* = 13.6 Hz, 1H, $C^1\mathbf{H_a}H_b$), 3.01 (dd, *J* = 13.6, 1.7 Hz, 1H, $C^1H_a\mathbf{H_b}$), 2.88 (d, *J* = 18.3 Hz, 1H, $C^2\mathbf{H_a}H_b$), 2.72 (dd, *J* = 18.3, 1.7 Hz, 1H, $C^2H_a\mathbf{H_b}$), 2.36 (s, 3H, C_{Arom}-$\mathbf{CH_3}$), 1.13 (s, 3H, CH_3). **^{13}C{^{1}H} NMR** (75.5 MHz, $CDCl_3$) δ 215.4 (C=O), 148.1 (C_{Arom}-CH_3), 136.5 (C_{Arom}-C), 136.0 (C_{Arom}-C), 128.6 (2xC_{Arom}-H), 127.6 (2xC_{Arom}-H), 125.5 (C_{Arom}-H), 125.4 (2xC_{Arom}-H), 124.3 (2xC_{Arom}-H), 56.7 (**C**CH_2Br), 51.9 (C^1H_2) , 44.4 (C^2H_2), 40.0 (**C**CH_2), 39.9 (CH_2Br), 30.2 (C_{Arom}-$\mathbf{CH_3}$), 19.9 (CH_3). **IR** (ATR) cm^{-1}: 3018 (=C-H), 2970 (C-H), 1738 (C=O), 1642 (C=C). **MS** (EI) e/z (%): 277.2 (26), 159.1 (22), 145.2 (22), 131.1 (100). **HRMS** (ESI^+): m/z calculated for $[C_{20}H_{21}OBr+Na]^+$ 379.0673; found 379.0673 $[M+Na]^+$.

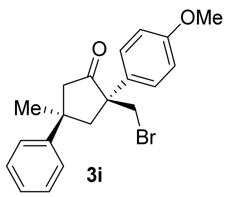

3i

(2*R*,4*R*)-2-(bromomethyl)-2-(4-methoxyphenyl)-4-methyl-4-phenylcyclopentan-1-one **3i**

Following the general procedure F, **3i** was isolated as a white solid (18.3 mg; 0.049 mmol; 49 %; dr>20:1; 80 %ee) starting from 1-(1-(4-methoxyphenyl)vinyl)-3-methyl-3-phenylcyclobutan-1-ol **1i** (29.4 mg; 0.1 mmol) and **2b** (4.3 mg; 0.0025 mmol) in toluene (0.50 mL) and NBS (19.6 mg; 0.11 mmol). The enantiomeric excess of product was determined by HPLC using Chiralpack OD-3 column [Hexane: *i*PrOH 99:1; flow = 1 mL/min.; tr_{major} = 24.5 min.; tr_{minor} = 30.8 min.]. **[α]**$_D^{25}$ = +13.2 (c = 0.7, $CHCl_3$). **Rf** = 0.37 (PE:EtOAc = 95:5). **M.p.** = 86-88 ºC. **^{1}H NMR** (300 MHz, $CDCl_3$) δ 7.52 – 7.46 (m, 2H, C_{Arom}-H), 7.41 – 7.34 (m, 4H, C_{Arom}-H), 7.30 – 7.23 (m, 1H, C_{Arom}-H), 6.96 – 6.87 (m, 2H, C_{Arom}-H), 3.82 (s, 3H, OCH_3), 3.62 (d, *J* = 10.1 Hz, 1H, $C\mathbf{H_a}H_bBr$), 3.43 (d, *J* = 10.1 Hz, 1H, $CH_a\mathbf{H_b}Br$), 3.06 (d, *J* = 13.6 Hz, 1H, $C^1\mathbf{H_a}H_b$), 2.99 (dd, *J* = 13.6, 1.9 Hz, 1H, $C^1H_a\mathbf{H_b}$), 2.88 (d, *J* = 18.3 Hz, 1H, $C^2\mathbf{H_a}H_b$), 2.72 (dd, *J* = 18.3, 1.9 Hz, 1H, $C^2H_a\mathbf{H_b}$), 1.13 (s, 3H, CH_3). **^{13}C{^{1}H} NMR** (75.5 MHz, $CDCl_3$) δ 216.5 (C=O), 159.0 (C_{Arom}-OCH_3), 149.1 (C_{Arom}-C), 131.9 (C_{Arom}-C), 128.6 (2xC_{Arom}-H), 127.8 (2xC_{Arom}-H), 126.4 (C_{Arom}-H), 125.3 (2xC_{Arom}-H), 114.2 (2xC_{Arom}-H), 57.3 (**C**CH_2Br), 55.3 (OCH_3), 53.0 (C^1H_2), 45.5 (C^2H_2), 41.0 (**C**-CH_3), 40.9 (CH_2Br), 31.2 (CH_3). **IR** (ATR) cm^{-1}: 3020 (=C-H), 2961 (C-H), 1737 (C=O), 1641 (C=C), 1472 (CH_2), 1456 (CH_3), 1253 (C-O). **MS** (EI) e/z (%): 147.1 (69), 133.1 (56.0), 121.1 (90), 91.1 (100). **HRMS** (ESI^+): m/z calculated for $[C_{20}H_{21}O_2Br+Na]^+$ 395.0623; found 395.0616 $[M+Na]^+$.

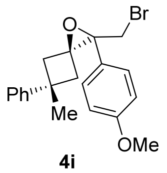

4i

2-(bromomethyl)-2-(4-methoxyphenyl)-5-methyl-5-phenyl-1-oxaspiro[2.3]hexane **4i**

Following the general procedure F, **4i** was isolated as a colorless oil (17.5 mg; 0.047 mmol; 47 %; 81 %ee) starting from 1-(1-(4-methoxyphenyl)vinyl)-3-methyl-3-phenylcyclobutan-1-ol **1i** (29.4 mg; 0.1 mmol) and **2b** (4.3 mg; 0.0025 mmol) in toluene (0.50 mL) and NBS (19.6 mg; 0.11 mmol). **Rf** = 0.38 (PE:EtOAc = 95:5). **^{1}H NMR** (300

MHz, $CDCl_3$) δ 7.32 (dd, *J* = 16.0, 8.6 Hz, 3H, C_{Arom}-H), 7.26 – 7.13 (m, 5H), 6.88 (d, *J* = 8.8 Hz, 2H, C_{Arom}-H), 3.85 – 3.78 (m, 3H, OMe + 1H, C**Ha**HbBr), 3.43 (d, *J* = 10.8 Hz, 1H, CHa**Hb**Br), 3.03 (d, *J* = 13.1 Hz, 1H, C^1**Ha**Hb), 2.60 (dt, *J* = 13.1, 1.9 Hz, 1H, C^1Ha**Hb**), 2.55 – 2.46 (m, 1H, C^2**Ha**Hb), 2.30 (d, *J* = 13.6 Hz, 1H, C^2Ha**Hb**), 1.63 (s, 3H, CH_3). **^{13}C{^{1}H} NMR** (75.5 MHz, $CDCl_3$) δ 159.21 (C_{Arom}-C), 149.04 (C_{Arom}-C), 128.36 (2xC_{Arom}-H), 127.84 (2xC_{Arom}-H), 127.80 (C_{Arom}-C), 125.90(C_{Arom}-H), 125.42 (2xC_{Arom}-H), 113.61 (C_{Arom}-H), 69.76 (**C**-CH_2Br), 63.96 (**C**-O), 55.23 (OCH_3), 42.70 (C^1H_2), 42.45 (C^2H_2), 36.83 (**C**-CH_3), 36.12 (CH_2Br), 32.14 (CH_3). **IR** (ATR) cm^{-1}: 3022 (=C-H), 2961 (C-H), 1612 (C=C). **HRMS** (ESI^+): m/z calculated for $[C_{20}H_{21}O_2Br+Na]^+$ 395.0623; found 395.0627 $[M+Na]^+$.

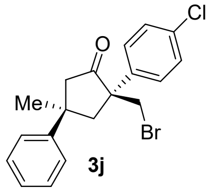

3j

(2*R*,4*R*)-2-(bromomethyl)-2-(4-chlorophenyl)-4-methyl-4-phenylcyclopentan-1-one **3j**

Following the general procedure F, **3j** was isolated as a white solid (28.4 mg; 0.075 mmol; 75 %; dr>20:1; 95 %ee) starting from 1-(1-(4-chlorophenyl)vinyl)-3-methyl-3-phenylcyclobutan-1-ol (29.9 mg; 0.10 mmol) **1j** and **2b** (4.3 mg; 0.0025 mmol) in Toluene (0.5 L) and NBS (19.6 mg; 0.11 mmol). The enantiomeric excess of product was determined by HPLC using Chiralpack OD-3 column [Hexane: *i*PrOH 99:1; flow = 1 mL/min.; tr_{minor} = 19.0 min.; tr_{major} = 30.7 min.]. **[α]**$_D^{26}$ = +391.2 (c = 0.1, $CHCl_3$). **Rf** = 0.38 (PE:EtOAc = 95:5). **M.p.** = 124-125 ºC. **^{1}H NMR** (300 MHz, $CDCl_3$) δ 7.58 – 7.51 (m, 2H, C_{Arom}-H), 7.44 – 7.33 (m, 6H, C_{Arom}-H), 7.31 – 7.23 (m, 1H, C_{Arom}-H), 3.55 (d, *J* = 10.2 Hz, 1H, C$\mathbf{H_a}$$H_b$Br), 3.42 (d, *J* = 10.2 Hz, 1H, CH_aH_bBr), 3.06 (d, *J* = 13.7 Hz, 1H, $C^1$$\mathbf{H_a}$$H_b$), 3.02 – 2.86 (m, 2H, $C^1H_a$$\mathbf{H_b}$ and $C^2$$\mathbf{H_a}$$H_b$), 2.72 (dd, *J* = 18.1, 1.9 Hz, 1H, $C^2H_a$$\mathbf{H_b}$), 1.14 (s, 3H, CH_3). **^{13}C{^{1}H} NMR** (75.5 MHz, $CDCl_3$) δ 215.8 (C=O), 148.5 (C_{Arom}-Cl), 138.6 (C_{Arom}-C), 133.4 (C_{Arom}-C), 129.0 (2xC_{Arom}-H), 128.7 (2xC_{Arom}-H), 128.1 (2xC_{Arom}-H), 126.6 (C_{Arom}-H), 125.3 (2xC_{Arom}-H), 57.3 (CCH_2Br), 52.8 (C^1H_2), 45.6 (C^2H_2), 41.1 (**C**-CH_3), 40.9 (CH_2Br), 31.3 (CH_3). **IR** (ATR) cm^{-1}: 3029 (=C-H), 2968 (C-H), 1736 (C=O), 1641 (C=C). **MS** (EI) m/z (%): 297.2 (44), 159.1 (30),153.0 (34), 152.0. (15), 151.0 (100). **HRMS** (ESI^+): m/z calculated for $[C_{19}H_{18}OBrCl+Na]^+$ 399.0127; found 399.0136 $[M+Na]^+$.

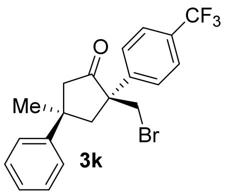

(2*R*,4*R*)-2-(bromomethyl)-4-methyl-4-phenyl-2-(4-(trifluoromethyl)phenyl)cyclopentan-1-one **3k**

Following the general procedure F, **3k** was isolated as a yellowish oil (11.4 mg; 0.03 mmol; 62 %; dr:9:1; 86 %ee) by FC (PE:EtOAc = 95:5), starting from (1r,3r)-3-methyl-3-phenyl-1-(1-(4-(trifluoromethyl)phenyl)vinyl)cyclobutan-1-ol **1k** (14.9 mg; 0.045 mmol) and **2b** (4.0 mg; 0.0022 mmol) in toluene (0.22 mL) and NBS (8.72 mg; 0.049 mmol). The enantiomeric excess of product was determined by HPLC using Chiralpack OD3 column [Hexane: *i*PrOH 99:1; flow = 1 mL/min; tr_{minor} = 22.5 min.; tr_{major} = 33.7 min.]. **[α]**$_D^{20}$= +20.8 (*c*=1, $CHCl_3$). **Rf** = 0.51 (PE:EtAcO = 95:5). **^{1}H NMR** (300 MHz, $CDCl_3$) δ 7.74 (d, *J* = 8.6 Hz, 2H, C_{Arom}-H), 7.64 (d, *J* = 8.6 Hz, 2H, C_{Arom}-H), 7.43–7.34 (m, 4H, C_{Arom}-H), 7.34–7.29 (m, 1H, C_{Arom}-H), 3.53 (d, *J* = 10.2 Hz, 1H, C$\mathbf{H_a}$$H_b$Br), 3.44 (d, *J* = 10.2 Hz, 1H, C$H_a$$\mathbf{H_b}$Br), 3.09 (d, *J* = 13.8 Hz, 1H, $C^1$$\mathbf{H_a}$$H_b$), 3.02 (d, *J* = 13.8 Hz, 1H, $C^1$$H_a$$\mathbf{H_b}$) 2.96 (d, *J* = 18.2 Hz, 1H, $C^2$$\mathbf{H_a}$$H_b$), 2.72 (dd, *J* = 18.2, 1.7 Hz, 1H, $C^2$$H_a$$\mathbf{H_b}$), 1.14 (s, 3H, CH_3). **^{19}F NMR** (282 MHz, $CDCl_3$) δ -62.7. **^{13}C{^{1}H} NMR** (75.5 MHz, $CDCl_3$) δ 215.4 (C=O), 148.2 (C_{Arom}-C), 144.1 (C_{Arom}-C), 130.0 (q, $J_{C\text{-}F}$ = 32.5 Hz, $\mathbf{C_{Arom}}$-CF_3,), 128.8 (2xC_{Arom}-H), 127.2 (2xC_{Arom}-H), 126.7 (C_{Arom}-H), 125.7 (q, $J_{C\text{-}F}$ = 3.8 Hz, 2xC_{Arom}-H), 125.3 (2xC_{Arom}-H), 123.8 (q, $J_{C\text{-}F}$ = 265.5 Hz, CF_3,), 57.6 (**C**-CH_2Br), 52.8 (C^1H_2), 45.7 (C^2H_2), 41.1 (**C**CH_3), 40.8 (CH_2Br), 31.3 (CH_3). **IR** (ATR) cm^{-1}: 1740 (C=O). **MS** (EI) e/z (%): 331.1 (5), 289.1 (19), 185 (77), 117. (100). **HRMS** (ESI$^+$): m/z calculated for $[C_{20}H_{18}OF_3Br+Na]^+$ 433.0391; found 433.0390 $[M+Na]^+$.

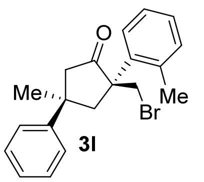

(2*R*,4*R*)-2-(bromomethyl)-4-methyl-4-phenyl-2-(o-tolyl)cyclopentan-1-one **3l**

Following the general procedure F, **3l** was isolated as a yellowish oil (32 mg; 0.09 mmol; 89 %; dr>20:1; 45 %ee) by FC (PE:EtOAc = 95:5), starting from 3-methyl-3-phenyl-1-(1-(o-tolyl)vinyl)cyclobutan-1-ol **1l** (27.8 mg; 0.10 mmol) and **2b** (4.3 mg; 0.0025 mmol) in toluene (0.5 mL) and NBS (19.6 mg; 0.11 mmol). The enantiomeric excess of product was determined by HPLC using Chiralpack OD3 column [Hexane: *i*PrOH 99.5:0.5; flow

= 0.5 mL/min; tr_{major} = 19.3 min.; tr_{minor} = 20.5 min.]. **[α]**$_D^{20}$= +20.3 (*c*=1, $CHCl_3$). **Rf** = 0.56 (PE:EtAcO = 95:5). **^{1}H NMR** (300 MHz, $CDCl_3$) δ 7.14–6.95 (m, 7H, C_{Arom}-H), 6.90–6.81 (m, 2H, C_{Arom}-H), 3.85 (d, *J* = 10.6 Hz, 1H, C**H$_a$**H$_b$Br), 3.66 (d, *J* = 10.6 Hz, 1H, CH$_a$**H$_b$**Br), 3.08 (d, *J* = 16.4 Hz, 1H, C^1**H$_a$**H$_b$), 2.98 (s, 2H, C^2H_2), 2.49 (d, *J* = 16.4 Hz, 1H, C^{1}H$_a$**H$_b$**), 2.31 (s, 3H, C_{Arom}-**CH$_3$**), 1.43 (s, 3H, CH_3). **^{13}C{^{1}H} NMR** (75.5 MHz, $CDCl_3$) δ 215.3 (C=O), 149.1 (C_{Arom}-C), 139.5 (C_{Arom}-C), 135.5 (C_{Arom}-C), 133.1 (C_{Arom}-H), 128.3 (2xC_{Arom}-H), 127.6 (C_{Arom}-H), 126.9 (C_{Arom}-H), 126.1 (C_{Arom}-H), 126.0 (C_{Arom}-H), 125.2 (2xC_{Arom}-H), 58.8 (**C**-CH_2Br), 51.5 (C^1H_2), 47.7 (C^2H_2), 40.0 (**C**CH_3), 36.1 (CH_2Br), 32.0 (C_{Arom}-**CH$_3$**), 21.7 (CH_3). **IR** (ATR) cm^{-1}: 1741 (C=O). **MS** (EI) e/z (%): 277.1 (2), 159.1 (16), 131.0 (48), 115 (100). **HRMS** (ESI^+): m/z calculated for $[C_{20}H_{21}OBr+Na]^+$ 379.0673; found 379.0671 $[M+Na]^+$.

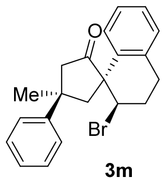

3m

(1*R*,2′*R*,4*R*)-2'-bromo-4-methyl-4-phenyl-3',4'-dihydro-2'H-spiro[cyclopentane-1,1'-naphthalen]-2-one **3m**

Following the general procedure F, **3m** was isolated as a colorless oil (28.0 mg; 0.076 mmol; 76 %; dr>20:1; 90 %ee) starting from 1-(3,4-dihydronaphthalen-1-yl)-3-methyl-3-phenylcyclobutan-1-ol **1m** (29.0 mg; 0.10 mmol) and **2b** (4.3 mg; 0.0025 mmol) in toluene (0.50 mL) and NBS (19.6 mg; 0.11 mmol). The enantiomeric excess of product was determined by HPLC using Chiralpack OD-3 column [Hexane: *i*PrOH 99:1; flow = 1 mL/min.; tr_{major} = 16.6 min.; tr_{minor} = 24.2 min.]. **[α]**$_D^{20}$ = +20.0 (c = 0.3, $CHCl_3$). **Rf** = 0.38 (PE:EtOAc = 95:5). **^{1}H NMR** (300 MHz, $CDCl_3$) δ 7.47 – 7.33 (m, 4H, C_{Arom}-H), 7.33 – 7.16 (m, 3H, C_{Arom}-H), 7.16 – 7.06 (m, 2H, C_{Arom}-H), 4.22 (dd, *J* = 5.6, 3.0 Hz, 1H, CHBr), 3.52 (d, *J* = 17.0 Hz, 1H; C^1**H$_a$**H$_b$), 3.24 (ddd, *J* = 17.1, 9.6, 6.9 Hz, 1H, C_{Arom}-C**H$_a$**H$_b$), 3.02 (d, *J* = 14.6 Hz, 1H, C^2**H$_a$**H$_b$), 2.82 (ddd, *J* = 17.1, 5.7, 4.4 Hz, 1H, C_{Arom}-CH$_a$**H$_b$**), 2.71 (d, *J* = 17.1 Hz, 1H, C^{1}H$_a$**H$_b$**), 2.63 (d, *J* = 14.5 Hz, 1H, C^{2}H$_a$**H$_b$**), 2.45 – 2.15 (m, 2H, C_{Arom}-CH_2**CH$_2$**), 1.53 (s, 3H, CH_3). **^{13}C{^{1}H} NMR** (75.5 MHz, $CDCl_3$) δ 213.5 (C=O), 149.4 (C_{Arom}-C), 135.6 (C_{Arom}-C), 135.5 (C_{Arom}-C), 129.1 (C_{Arom}-H), 128.9 (C_{Arom}-H), 128.9 (2xC_{Arom}-H), 127.0 (C_{Arom}-H), 126.6 (C_{Arom}-H), 126.6 (C_{Arom}-H), 125.2 (2xC_{Arom}-H), 59.1 (**C**-CHBr), 55.3 (C^1H_2), 54.9 (CHBr), 51.2 (C^2H_2), 39.2 (**C**-CH_3), 34.2 (CH_3), 27.8 (C_{Arom}-**CH$_2$**), 26.1 (C_{Arom}-CH_2**CH$_2$**). **IR** (ATR) cm^{-1}: 3022 (=C-

H), 2957 (C-H), 1744 (C=O), 1636 (C=C). **MS** (EI) m/z (%): 288.2 (44), 170.1 (44), 141.1 (100). **HRMS** (ESI^+): m/z calculated for $[C_{21}H_{22}OBr]^+$ 369.0854; found 369.0848 $[M+H]^+$.

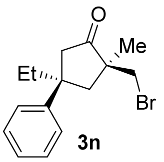

3n

(2*S*,4*R*)-2-(bromomethyl)-4-ethyl-2-methyl-4-phenylcyclopentan-1-one **3n**

Following the general procedure F, **3n** was isolated as a yellowish oil (25.2 mg; 0.08 mmol; 85 %; dr: 1.2:1; 1st diastereomer 70 %ee and 2nd diastereomer 72 %ee) by FC (PE:EtOAc = 95:5), starting from 3-ethyl-3-phenyl-1-(prop-1-en-2-yl)cyclobutan-1-ol (21.6 mg; 0.10 mmol) **1n** and **2b** (4.3 mg; 0.0025 mmol) in toluene (0.5 mL) and NBS (19.6 mg; 0.11 mmol). The enantiomeric excess of product was determined by HPLC using Chiralpack OD3 column [Hexane: *i*PrOH 99:1; flow = 0.5 mL/min. 1st diastereomer: tr_{major} = 14.8 min.; tr_{major} = 18.8 min. 2nd diastereomer: tr_{minor} = 18.2 min.; tr_{major} = 25.7 min.]. $[\alpha]_D^{20}$= -36.7 (*c*=1, $CHCl_3$). **Rf** = 0.48 (PE:EtAcO = 95:5). The spectral data is given for a mixture 1.2:1 of both diastereomers: **^{1}H NMR** (300 MHz, $CDCl_3$) δ 7.32—7.12 (m, 5H, C_{Arom}-H of each diastereomers), 3.39 (d, *J* = 10.0 Hz, 1H, C**H_a**H_bBr minor), 3.22 (d, *J* = 10.0 Hz, 1H, CH_a**H_b**Br minor), 3.11 (d, *J* = 10.0 Hz, 1H, C**H_a**H_bBr major), 3.05 (dd, *J* = 17.5, 1.8 Hz, 1H, C^1**H_a**H_b minor), 2.96 (d, *J* = 10.0 Hz, 1H, CH_a**H_b**Br major), 2.89 (d, *J* = 17.4 Hz, 1H, C^1**H_a**H_b major), 2.64 (d, *J* = 13.8 Hz, 1H, C^2**H_a**H_b major), 2.59 (d, *J* = 17.4 Hz, 1H, C^1H_a**H_b** major), 2.50 (d, *J* = 13.4 Hz, 1H, C^2**H_a**H_b minor), 2.40 (d, *J* = 17.5 Hz, 1H, C^1H_a**H_b** minor), 2.22 (dd, *J* = 13.4, 1.8 Hz, 1H, C^2H_a**H_b** minor), 2.11 (d, *J* = 13.8 Hz, 1H, C^2H_a**H_b** major), 1.89 (dq, *J* = 13.8, 7.4 Hz, 1H, C**H_a**H_bCH_3 minor), 1.81—1.53 (m, 3H, CH_a**H_b**CH_3 minor, C**H_a**H_bCH_3 major, CH_a**H_b**CH_3 major), 1.24 (s, 3H, CH_3 major), 0.72 (s, 3H, CH_3 minor), 0.58 (t, *J* = 7.4 Hz, 3H, CH_2CH_3 minor), 0.57 (t, *J* = 7.4 Hz, 3H, CH_2CH_3 major). **^{13}C{^{1}H} NMR** (75.5 MHz, $CDCl_3$) δ 218.3 (C=O, minor), 218.2 (C=O, major), 145.8 (C_{Arom}-C, major), 145.6 (C_{Arom}-C, minor), 128.5 (2xC_{Arom}-H of each diastereomer), 126.5 (C_{Arom}-H major), 126.5 (C_{Arom}-H minor), 126.4 (2xC_{Arom}-H of each diastereomer), 50.0 (CH_2Br, major), 49.6 (**C**-CH_2Br, major), 49.5 (**C**-CH_2Br, minor), 49.4 (CH_2Br, minor), 47.8 (C^1H_2 minor), 46.1 (C^1H_2 major), 45.0 (C-CH_2 minor), 44.9 (C-CH_2 major), 41.4 (C^2H_2 major), 40.9 (C^2H_2 minor), 37.5 (**CH_2**CH_3 minor), 36.5 (**CH_2**CH_3 major), 24.6 (CH_3 major), 24.2 (CH_3 minor), 9.5

(CH_2**CH_3**, minor), 9.1 (CH_2**CH_3**, major). **IR** (ATR) cm^{-1}: 1738 (C=O). **MS** (EI) e/z (%): 296.9 (M+H, 2), 264.9 (20), 157 (95), 55 (100). **HRMS** (ESI^+): decomposition.

Synthesis and characterization of products 4a and 3a

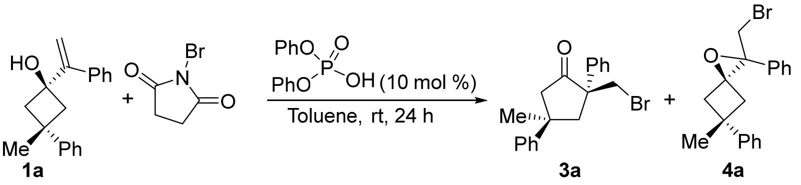

ratio **3a**:**4a** =3:1
57 % Yield

Scheme S 9. Synthesis of epoxide **4a**.

Compound **4a** was prepared by treating 3-methyl-3-phenyl-1-(1-phenylvinyl)cyclobutan-1-ol **1a** (79.2 mg; 0.3 mmol) and diphenylphosphoric acid (7.5 mg; 0.01 mmol) in toluene (1.5 mL) with NBS (64.2 mg; 0.36 mmol) at room temperature. The reaction was let stirring for 24 hours, then it was concentrated under vacuum and directly purified by column chromatography (PE:EtOAc = 95:5) to obtain 58.8 mg as a mixture of 2-(bromomethyl)-4-methyl-2,4-diphenylcyclopentan-1-one **3a** and 2-(bromomethyl)-5-methyl-2,5-diphenyl-1-oxaspiro[2.3]hexane **4a** in a 3:1 ratio. The epoxide **4a** was separated by preparative TLC employing PE:EtOAc 95:1. **Rf** = 0.36 (PE:EtOAc = 95:5). **^{1}H NMR** (300 MHz, $CDCl_3$) δ 7.41 – 7.30 (m, 5H, C_{Arom}-H), 7.29 – 7.17 (m, 5H, C_{Arom}-H), 3.88 (d, *J* = 10.9 Hz, 1H, C**H$_a$**H$_b$Br), 3.44 (d, *J* = 10.9 Hz, 1H, CH$_a$**H$_b$**Br), 3.13 – 2.94 (dt, *J* = 13.1, 1.6 Hz, 1H, C^1**H$_a$**H$_b$), 2.62 (ddd, *J* = 13.1, 2.5, 1.3 Hz, 1H, C^{1}H$_a$**H$_b$**), 2.49 (app dt, *J* = 13.4, 1.6 Hz, 1H, C^2**H$_a$**H$_b$), 2.31 (ddd, *J* = 13.4, 2.5, 1.3 Hz, 1H, C^{2}H$_a$**H$_b$**), 1.63 (s, 1H, CH_3). **^{13}C{^{1}H} NMR** (75.5 MHz, $CDCl_3$) δ 148.98 (C_{Arom}-C), 135.80 (C_{Arom}-C), 128.36 (2xC_{Arom}-H), 128.18 (2xC_{Arom}-H), 127.89 (C_{Arom}-H), 126.57 (2xC_{Arom}-H), 125.92 (C_{Arom}-H), 125.39 (2xC_{Arom}-H), 69.88 (**C**-CH_2Br), 64.20 (**C**-O), 42.67 (C^{1}H$_2$), 42.44 (C^{2}H$_2$), 36.90 (**C**-CH_3), 35.77 (CH_2Br), 32.13 (CH_3). **IR** (ATR) cm^{-1}: 3020 (=C-H), 2958 (C-H). **HRMS** (ESI$^+$): m/z calculated for $[C_{19}H_{19}OBr+Na]^+$ 365.0517; found 365.0507 $[M+Na]^+$.

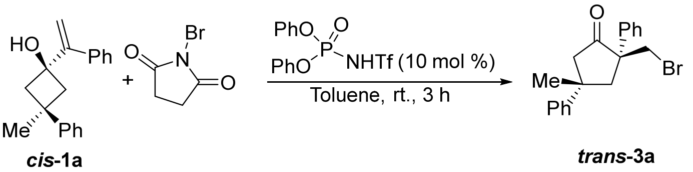

***trans*-3a**
dr = 6:1
83 % Yield

Scheme S 10. Synthesis of ketone **3a***trans*.

Compound ***trans*-3a** was prepared by treating 3-methyl-3-phenyl-1-(1-phenylvinyl)cyclobutan-1-ol ***cis*-1a** (26.4 mg; 0.1 mmol) and diphenyl

((trifluoromethyl)sulfonyl)phosphoramidate (3.8 mg; 0.01 mmol) in toluene (0.5 mL) with NBS (21.4 mg; 0.12 mmol) at room temperature. The reaction was let stirring for 3 h and then it was concentrated under vacuum and directly purified by column chromatography (PE:EtOAc = 95:5) to obtain 2-(bromomethyl)-4-methyl-2,4-diphenylcyclopentan-1-one ***trans*-3a** (28.5 mg; 0.083 mmol; 83 % yield). **Rf** = 0.36 (PE:EtOAc = 95:5). **IR** (ATR) cm^{-1}: 3020 (=C-H), 2958 (C-H), 1739 (C=O), 1643 (C=C), 1465 (CH_2), 1439 (CH_3). **MS** (EI) e/z (%): 263.2 (34), 205.1 (25), 145.1 (39), 117.1 (100). **HRMS** (ESI^+): m/z calculated for $[C_{19}H_{19}OBr+Na]^+$ 365.0517; found 365.0507 $[M+Na]^+$. **^{1}H NMR** (300 MHz, $CDCl_3$) δ 7.35 – 7.27 (m, 1H, C_{Arom}-H), 7.15 – 6.95 (m, 9H, C_{Arom}-H), 3.68 (d, *J* = 10.3 Hz, 1H, C$\mathbf{H_a}$$H_b$Br), 3.53 (d, *J* = 10.3 Hz, 1H, C$H_a$$\mathbf{H_b}$Br), 3.02 (dd, *J* = 13.9, 12.1 Hz, 2H, $C^1$$\mathbf{H_a}$$H_b$+$C^2$$\mathbf{H_a}$$H_b$), 2.81 (d, *J* = 13.8 Hz, 1H, $C^1$$H_a$$\mathbf{H_b}$), 2.47 (d, *J* = 17.3 Hz, 1H, $C^2$$H_a$$\mathbf{H_b}$), 1.47 (s, 3H, CH_3). **^{13}C{^{1}H} NMR** (75.5 MHz, $CDCl_3$) δ 215.01 (C=O), 147.73 (C_{Arom}-C), 139.13 (C_{Arom}-C), 128.38 (2xC_{Arom}-H), 128.27 (2xC_{Arom}-H), 127.28 (C_{Arom}-H), 126.53 (2xC_{Arom}-H), 126.15 (C_{Arom}-H), 125.53 (2xC_{Arom}-H), 57.87 (**C**-CH_2Br), 51.74 (C^1H_2), 46.79 (**CH_2**Br), 40.99 (C^2H_2), 40.32 (**C**-CH_3), 31.74 (CH_3).

Synthesis and characterization of catalysts 2b and 2k-2m.

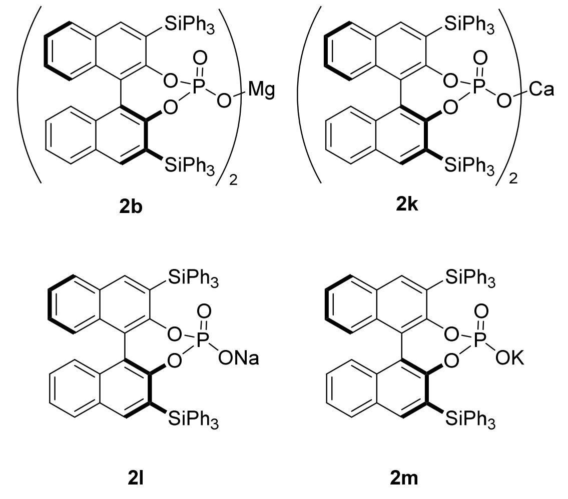

Figure S 6. Chiral phosphates salts **2b**, **2k-2m**.

All compounds were prepared according to the reported general procedure G[13]

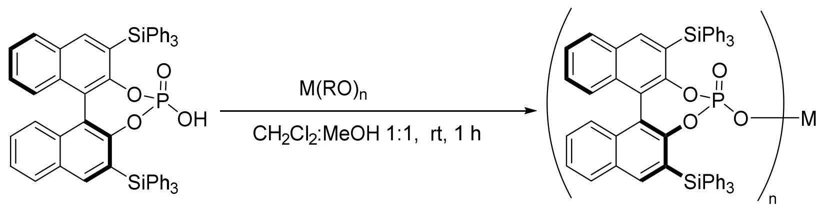

Scheme S 11. Synthesis of catalyst **2d.**

General procedure G for the synthesis of catalysts **2b, 2k-2m.**

To a flame dry test tube **2a** and Mg(O*t*Bu)$_2$, Ca(OMe)$_2$, NaOMe or KO*t*Bu were added followed by the addition of 0.3 mL each of dry CH_2Cl_2 and dry MeOH and reaction mixture was stirred for 1 h. Solvents were removed under reduced pressure to obtain catalysts **2b, 2k-2m** quantitatively as a white solid.

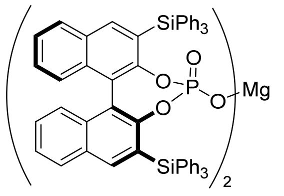

2b

Catalyst **2a** (500 mg; 0.579 mmol) was dissolved in dry mixture of CH_2Cl_2:MeOH 1:1 (11.6 mL) in a dry reaction tube and Mg(O*t*Bu)$_2$ (53 mg, 0.289 mmol) was added. The reaction mixture was let stir at room temperature for 1h, then solvents were evaporated in

[13] G K. Ingle, Y. Liang, M. G. Mormino, G. Li, F. R. Fronczek, J. C. Antilla, *Org. Lett.* **2011**, *13*, 2054–2057. M. Klussmann, L. Ratjen, S. Hoffmann, V. Wakchaure, R. Goddard, B. List, *Synlett*, **2010**, *14*, 2189-2192.

vacuum to obtain the catalyst **2b** (507 mg, 0.289 mmol; quant.) without purification. **[α]**$_D^{20}$= -167 (*c*=0.31, $CHCl_3$). **^{1}H NMR** (300 MHz, $CDCl_3$) δ 7.84 (s, 4H), 7.62 (d, *J* = 8.0 Hz, 4H), 7.58 – 7.48 (m, 24H), 7.31 – 7.10 (m, 44H), 7.03 (d, *J* = 8.4 Hz, 4H). **^{13}C{^{1}H} NMR** (75.5 MHz, $CDCl_3$) δ 152.85 (2xC_{Arom}-C), 152.73 (2xC_{Arom}-C), 141.99 (4xC_{Arom}-H), 136.75 (24xC_{Arom}-H), 134.77 (2xC_{Arom}-C), 134.76 (2xC_{Arom}-C), 134.56 (12xC_{Arom}-C), 130.35 (2xC_{Arom}-C), 130.34 (2xC_{Arom}-C), 129.60 (12xC_{Arom}-H), 128.54 (4xC_{Arom}-H), 127.85 (24xC_{Arom}-H), 127.23 (4xC_{Arom}-H), 126.76 (4xC_{Arom}-H), 125.71 (2xC_{Arom}-C), 125.66 (2xC_{Arom}-C), 124.86 (4xC_{Arom}-H), 121.75 (2xC_{Arom}-C), 121.72 (2xC_{Arom}-C). **^{31}P NMR** (122 MHz, $CDCl_3$) δ 1.27 ppm. **MALDI-LTQ-ORBITRAP**: m/z calculated for $C_{112}H_{80}O_8P_2Si_4MgNa$: 1774.4182; m/z found for $C_{112}H_{80}O_8P_2Si_4MgNa$: 1774.4166.

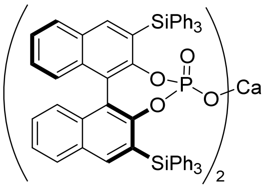

2k

Catalyst **2a** (30 mg; 0.0347 mmol) was dissolved in dry mixture of CH_2Cl_2:MeOH 1:1 (0.6 mL) in a dry reaction tube and $Ca(OMe)_2$ (1.8 mg; 0.0174 mmol) was added. The reaction mixture was let stir at room temperature for 1h, then solvents were evaporated in vacuum to obtain catalyst **2k** (30.8 mg, 0.0174 mmol; quant.) without purification. **[α]**$_D^{20}$= -105.15 (*c*= 0.96, $CHCl_3$). **^{1}H NMR** (300 MHz, $CDCl_3$) δ 7.84 (s, 4H), 7.62 (d, *J* = 8.0 Hz, 4H), 7.58 – 7.48 (m, 24H), 7.31 – 7.10 (m, 44H), 7.03 (d, *J* = 8.4 Hz, 4H). **^{13}C{^{1}H} NMR** (75.5 MHz, $CDCl_3$) δ 152.85 (2x$_{Arom}$-C), 152.73 (2xC_{Arom}-C), 142.13 (4xC_{Arom}-H), 136.84 (24xC_{Arom}-H), 134.93 (12xC_{Arom}-C), 134.67 (4xC_{Arom}-C), 130.35 (4xC_{Arom}-C), 129.42 (12xC_{Arom}-H), 128.60 (4xC_{Arom}-H), 127.73 (24xC_{Arom}-H), 127.22 (4xC_{Arom}-H), 126.80 (4xC_{Arom}-H), 125.74 (2xC_{Arom}-C), 125.70 (2xC_{Arom}-C), 124.81 (4xC_{Arom}-H), 121.93 (2xC_{Arom}-C), 121.91 (2xC_{Arom}-C). **^{31}P NMR** (122 MHz, $CDCl_3$) δ 1.83 ppm.

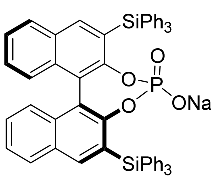

2l

Catalyst **2a** (30 mg; 0.0347 mmol) was dissolved in dry mixture of CH_2Cl_2:MeOH 1:1 (0.6 mL) in a dry reaction tube and NaOMe (1.9 mg, 0.0347mmol) was added. The

reaction mixture was let stir at room temperature for 1h, then solvents were evaporated in vacuum to obtain catalyst **2l** (30.8 mg, 0.0347 mmol; quant.) without purification. **[α]**$_D^{20}$= -159.23 (*c*=0.07, $CHCl_3$). **^{1}H NMR** (300 MHz, $CDCl_3$) δ 7.84 (s, 4H), 7.62 (d, *J* = 8.0 Hz, 4H), 7.58 – 7.48 (m, 24H), 7.31 – 7.10 (m, 44H), 7.03 (d, *J* = 8.4 Hz, 4H). **^{13}C{^{1}H} NMR** (75.5 MHz, $CDCl_3$) δ 153.20 (1xC$_{Arom}$-C), 153.08 (1xC$_{Arom}$-C), 141.83 (2xC$_{Arom}$-H), 136.75 (12xC$_{Arom}$-H), 135.00 (2xC$_{Arom}$-C), 134.88 (6xC$_{Arom}$-C), 130.37 (C$_{Arom}$-C), 129.14 (6xC$_{Arom}$-H), 128.59 (2xC$_{Arom}$-H), 127.58 (12xC$_{Arom}$-H), 127.13 (2xC$_{Arom}$-H), 127.03 (2xC$_{Arom}$-H), 126.17 (2xC$_{Arom}$-C), 126.13 (C$_{Arom}$-C), 124.66 (2xC$_{Arom}$-H), 121.78 (C$_{Arom}$-C), 121.76 (C$_{Arom}$-C). **^{31}P NMR** (122 MHz, $CDCl_3$) δ 1.49 ppm.

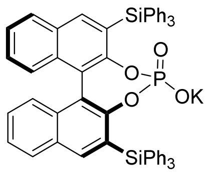

2m

Catalyst **2a** (30 mg; 0.0347 mmol) was dissolved in dry mixture of CH_2Cl_2:MeOH 1:1 (0.6 mL) in a dry reaction tube and KO*t*Bu (3.9 mg,0.0347 mmol) was added. The reaction mixture was let stir at room temperature for 1h, then solvents were evaporated in vacuumto obtain catalyst **2m** (31 mg, 0.035 mmol; quant.) without purification. **[α]**$_D^{20}$= -146.86 (*c*=0.17, $CHCl_3$). **^{1}H NMR** (300 MHz, $CDCl_3$) δ 7.84 (s, 4H), 7.62 (d, *J* = 8.0 Hz, 4H), 7.58 – 7.48 (m, 24H), 7.31 – 7.10 (m, 44H), 7.03 (d, *J* = 8.4 Hz, 4H). **^{13}C{^{1}H} NMR** (75.5 MHz, $CDCl_3$) δ 152.28 (C$_{Arom}$-C), 152.16 (C$_{Arom}$-C), 140.78 (2xC$_{Arom}$-H), 135.76 (12xC$_{Arom}$-H), 133.79 (2xC$_{Arom}$-C),133.57 (6xC$_{Arom}$-C), 129.20 (2xC$_{Arom}$-C), 128.27 (6xC$_{Arom}$-H), 127.61 (2xC$_{Arom}$-H), 126.61 (12xC$_{Arom}$-H), 126.23 (2xC$_{Arom}$-H), 125.53 (2xC$_{Arom}$-H), 125.12 (C$_{Arom}$-C), 125.08 (C$_{Arom}$-C), 123.75 (2xC$_{Arom}$-H), 120.99 (C$_{Arom}$-C), 120.96. (C$_{Arom}$-C). **^{31}P NMR** (122 MHz, $CDCl_3$) δ 1.81 ppm.

Non linear effect study.

Following the general procedure G, catalysts with different enantiomeric ratios were prepared using different ratios of *R* and *S* enantiomers of the corresponding chiral phosphoric acids (Scheme S 12).

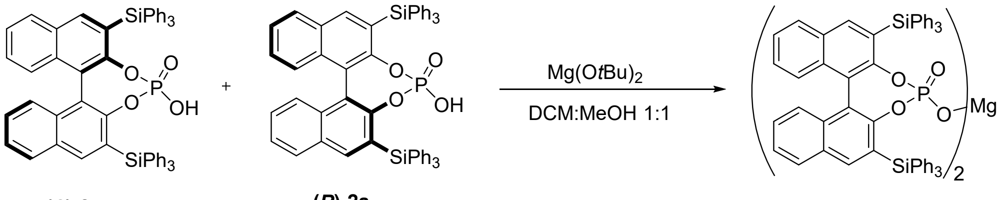

Scheme S 12. Synthesis of catalyst using different ratios of chiral phosphoric acid enantiomers.

In order to verify the formation of catalysts, ^{31}P-NMR spectra in $CDCl_3$ were recorded, observing two signals that have a similar proportion to the ratio of phosphoric acid enantiomers used in each case.

With these catalysts in hand, the model reaction was carried out under the conditions established in general procedure F, the final product was purified, and the enantiomeric excess was measured by HPLC (Table S 6).

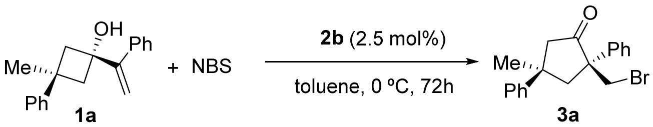

Table S 5. Non linear effect results.[a]

Entry	er catalyst (*R*):(*S*)	^{31}P-NMR signals (ppm)	ee product %[b]
1	50:50 (0)	1.84, 1.28	6
2	60:40 (20)	1.76, 1.26	30
3	70:30 (40)	1.94, 1.46	46
4	80:20 (60)	1.66, 1.09	56
5	90:10 (80)	1.65, 1.21	72
6	100:0 (100)	1.27	92

[a] Reactions carried out with 0.10 mmol of **1a**, 0.11 mmol of NBS and 2.5 mol% of **2b** with different ee ratios in toluene (0.2 M) at 0 °C for 72h. The numbers between parenthesis indicate the enaniomeric excess of catalyst **2b** [b] Product **3a** was isolated and ee was determined by HPLC on a chiral stationary phase.

Hammett study

Hammett study was carried out to analyze the effect of the substitution in *para*-position of the aromatic ring. The analysis was performed based on competition experiments between the unsubstituted cyclobutanol **1a** and its *para*-substituted equivalent **1h-k**. These competition experiments were carried out with 0.05 mmol of **1a**, 0.05 mmol of **1h-1k**, 0.055 mmol of NBS and 2.5 mol% (0.00125 mmol) of catalyst **2b** in toluene (0.25 mL) at 0 °C for 45 h. The experiments were quenched with aq. std. $Na_2S_2O_3$ (1 mL) and the products were extracted with Et_2O (3 x 2 mL). At this point, 0.05 mmol of 1,3,5-trimethoxybenzene was added as standard for subsequent NMR analysis. The mixture was dried with Na_2SO_4, filtered, and concentrated under vacuum.

These experiments were stopped at 45 h and the product ratio [$\mathbf{p_X}$]/[$\mathbf{p_H}$] was calculated by ^{1}H-NMR using 1,3,5-trimethoxybenzene as standard. This experimental ratio was approximated to rate constants ratio $\mathbf{k_X/k_H}$. [14]

Table S 6. Data obtained from competition experiments in Hammett study.

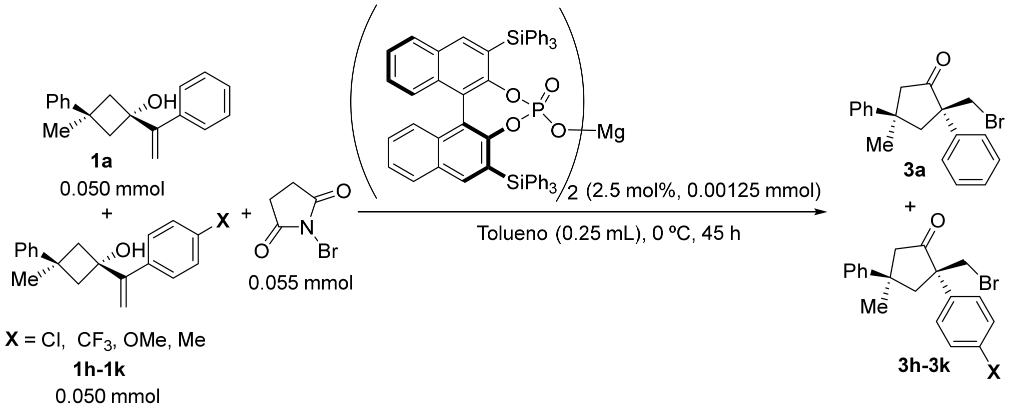

Entry	1	Substituent (X)	σ_p[a]	σ_p^+[b]	$k_{[X]}/k_{[H]}$	log [k_X]/[k_H]
**1**	**1h**	**Me**	-0,17	-0,31	5,25	0,72
**2**[c]	**1i**	**OMe**	-0,27	-0,78	9,71	0,99
**3**	**1j**	**Cl**	0,23	0,11	0,6	-0,22
**4**	**1k**	**CF_3**	0,54	0,61	0,08	-1,08

[a] Hammett constants for *para* substituents with inductive effect.[15] [b] Hammett constants for *para* substituents with resonance effect.[15]. [c] Epoxide **4i** was also considered in [$\mathbf{p_x}$] in this case.

[14] F. Romanov-Michailidis, M. Romanova-Michaelides, M. Pupier and A. Alexakis, *Chem. Eur. J.* **2015**, *21*, 5561-5583.
[15] Hammett, L. P. *J. Am. Chem. Soc.* **1937**, *59*, 96-103.

The logarithms of the rate constants ratios were plotted out with the Hammett substituent constants (σ and σ$^+$) for each substituent,[16] according to the known Hammett relationship [Eq. (1)].

$$log\left(\frac{k_X}{k_H}\right) = \rho\sigma \quad (1)$$

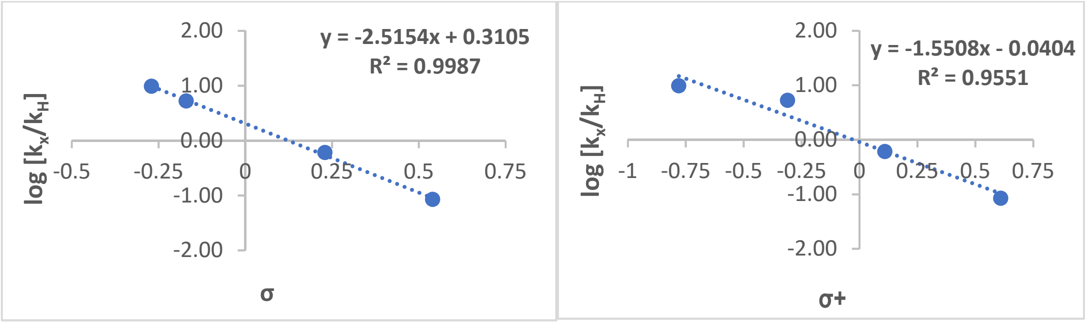

Figure S 7. Hammett plots for σ and σ^+.

[16] C. Hansch, A. Leo, R. W. Taft, *Chem. Rev.* **1991**, *91*, 165-195.

X-Ray analysis of product 3j.

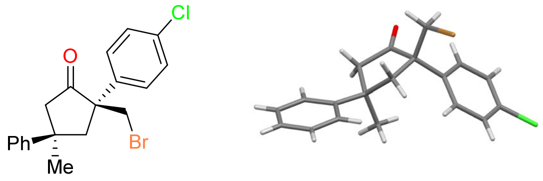

3j

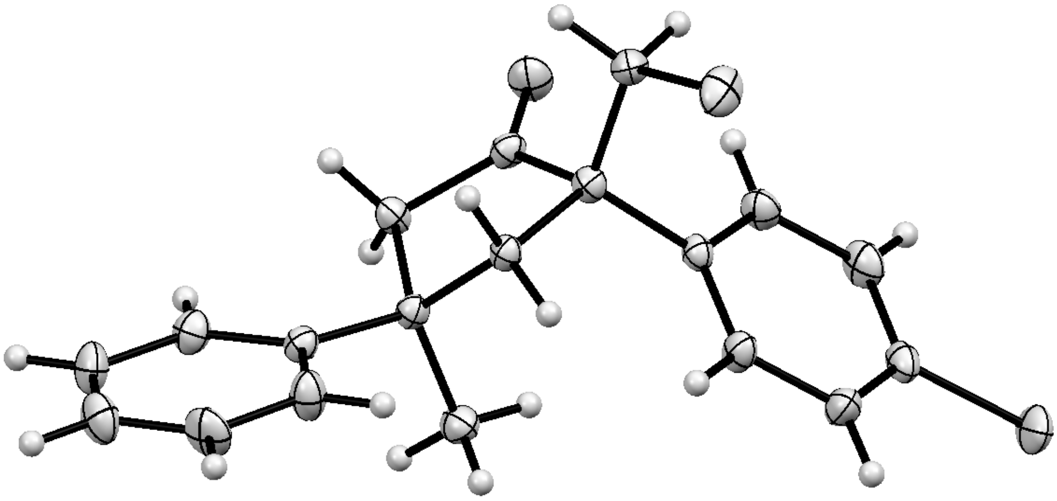

Figure S 8. X-Ray structure and ORTEP diagram (50% probability) for **3j.**

The enantiopure sample of compound **3j** was obtained following General Procedure F. Crystals of the sample were obtained after slow evaporation of a mixture of Hexane/Ethyl acetate 1:1 in a screw-top vial.

Crystal Data for $C_{19}H_{18}BrClO$ (M =377.69 g/mol) (**3j**, CCDC number 2091677): orthorhombic, space group $P2_12_12_1$ (no. 19), a = 6.83880(10) Å, b = 13.6467(2) Å, c = 17.6849(2) Å, V = 1650.48(4) Å^3, Z = 4, T = 150.01(10) K, μ(CuKα) = 4.863 mm^{-1}, $Dcalc$ = 1.520 g/cm^3, 15576 reflections measured (8.18° ≤ 2Θ ≤ 137.86°), 3075 unique (R_{int} = 0.0537, R_{sigma} = 0.0373) which were used in all calculations. The final R_1 was 0.0294 (>2sigma(I)) and wR_2 was 0.0713 (all data).

Table S 7. Crystal data and structure refinement for **3j.**

Identification code	**3j**
Empirical formula	$C_{19}H_{18}BrClO$
Formula weight	377.69
Temperature/K	150.01(10)
Crystal system	orthorhombic
Space group	$P2_12_12_1$
a/Å	6.83880(10)
b/Å	13.6467(2)
c/Å	17.6849(2)
α/°	90.00
β/°	90.00
γ/°	90.00
Volume/Å^3	1650.48(4)
Z	4
ρ_{calc}g/cm^3	1.520
μ/mm^{-1}	4.863
F(000)	768.0
Crystal size/mm^3	0.223 × 0.051 × 0.043
Radiation	CuKα (λ = 1.54184)
2Θ range for data collection/°	8.18 to 137.86
Index ranges	$-8 \le h \le 8$, $-16 \le k \le 16$, $-21 \le l \le 18$
Reflections collected	15576
Independent reflections	3075 [R_{int} = 0.0537, R_{sigma} = 0.0373]
Data/restraints/parameters	3075/0/200
Goodness-of-fit on F^2	1.058
Final R indexes [I>=2σ (I)]	R_1 = 0.0294, wR_2 = 0.0691
Final R indexes [all data]	R_1 = 0.0323, wR_2 = 0.0713
Largest diff. peak/hole / e Å^{-3}	0.33/-0.36
Flack parameter	-0.031(19)

Table S 8. Fractional Atomic Coordinates (×10^4) and Equivalent Isotropic Displacement Parameters (Å^2×10^3) for **3j**. U_{eq} is defined as 1/3 of of the trace of the orthogonalised U_{IJ} tensor.

Atom	*x*	*y*	*z*	U(eq)
Br01	4630.2(5)	3194.5(2)	5220.14(18)	28.87(10)
Cl00	5089.4(13)	6229.1(6)	8115.2(3)	31.33(19)
O003	-562(3)	5503.4(16)	4859.1(12)	25.9(5)
C004	4661(5)	6448.8(19)	3131.4(14)	16.6(5)
C005	1071(4)	5617(2)	4613.1(15)	17.4(6)
C006	4411(4)	5305.4(19)	4276.7(13)	15.4(5)
C007	5389(5)	5650(2)	5907.8(15)	20.2(6)
C008	3829(4)	6307(2)	3926.8(15)	15.9(5)
C009	3476(4)	5447(2)	5708.3(15)	16.0(6)
C00A	3591(5)	6916(2)	2569.6(16)	22.4(6)
C00B	2930(4)	5087(2)	4908.3(15)	16.2(6)
C00C	4514(5)	7168(2)	4418.2(15)	22.1(6)
C00D	4397(5)	7090(2)	1860.9(17)	27.5(7)
C00E	2504(5)	5769(2)	7005.0(17)	25.9(7)
C00F	4431(5)	5953(2)	7184.1(15)	22.1(6)
C00G	5893(5)	5895(2)	6649.2(16)	23.5(7)
C00H	2379(5)	4004(2)	4955.3(15)	20.6(6)
C00I	2040(5)	5511(2)	6264.3(17)	21.9(6)
C00J	1590(4)	6248(2)	3939.9(15)	17.5(6)
C00K	6581(5)	6176(3)	2974.3(17)	25.3(7)
C00L	7400(5)	6357(3)	2267.1(18)	29.9(7)
C00M	6308(5)	6812(3)	1712.9(16)	28.1(7)

Table S 9. Anisotropic Displacement Parameters ($Å^2 \times 10^3$) for **3j**. The Anisotropic displacement factor exponent takes the form: $-2\pi^2[h^2a^{*2}U_{11}+2hka^*b^*U_{12}+\ldots]$.

Atom	U_{11}	U_{22}	U_{33}	U_{23}	U_{13}	U_{12}
Br01	34.31(17)	21.47(15)	30.83(15)	-1.30(13)	-7.76(14)	9.04(14)
Cl00	43.7(5)	37.5(4)	12.8(3)	-3.2(3)	2.7(3)	-7.9(4)
O003	16.2(11)	33.3(11)	28.0(10)	3.8(9)	2.8(9)	1.2(9)
C004	16.2(12)	18.3(12)	15.4(11)	-2.1(9)	0.6(12)	0.5(12)
C005	14.0(13)	20.8(14)	17.3(13)	-1.4(10)	-0.4(10)	0.1(11)
C006	14.2(14)	18.6(13)	13.3(11)	-0.8(10)	1.2(10)	1.0(12)
C007	17.7(13)	28.8(14)	14.2(11)	-2.3(10)	2.9(11)	-1.1(13)
C008	14.0(13)	19.1(14)	14.8(12)	-0.6(10)	0.1(10)	0.1(12)
C009	18.2(15)	16.6(13)	13.2(12)	-0.1(10)	1.8(11)	0.6(11)
C00A	22.6(15)	25.3(16)	19.3(13)	-1.8(12)	0.5(11)	3.0(14)
C00B	14.7(14)	18.3(14)	15.5(13)	-1.6(10)	2.5(11)	1.1(10)
C00C	25.4(15)	21.7(14)	19.2(12)	-2.7(10)	1.4(12)	-3.4(13)
C00D	32.4(18)	34.5(17)	15.4(12)	2.3(11)	0.3(13)	2.8(14)
C00E	27.6(17)	30.7(17)	19.4(14)	-1.6(12)	10.0(13)	0.3(14)
C00F	32.6(18)	19.4(13)	14.1(12)	-0.6(10)	2.8(12)	-2.6(14)
C00G	23.1(17)	29.9(16)	17.6(14)	-0.8(12)	-1.4(11)	-6.2(13)
C00H	22.3(15)	19.9(14)	19.6(13)	0.6(10)	-2.3(11)	-3.8(12)
C00I	17.1(15)	27.0(16)	21.7(14)	2.9(12)	3.2(12)	-0.9(13)
C00J	14.3(14)	17.5(14)	20.6(13)	3.5(11)	1.0(10)	2.1(11)
C00K	21.0(16)	35.1(18)	19.8(14)	4.8(12)	2.3(12)	4.7(14)
C00L	23.4(17)	39.6(19)	26.8(15)	3.2(14)	12.3(14)	-1.6(15)
C00M	34.0(18)	32.2(16)	18.1(13)	1.3(14)	7.6(12)	2.1(17)

^{1}H, $^{13}C\{^{1}H\}$ (75.5 MHz), ^{19}F and ^{31}P NMR spectra of cyclobutanones, 1a-n, 3a-n, 4a, 4i and catalysts 2a-b and 2k-m in $CDCl_3$).

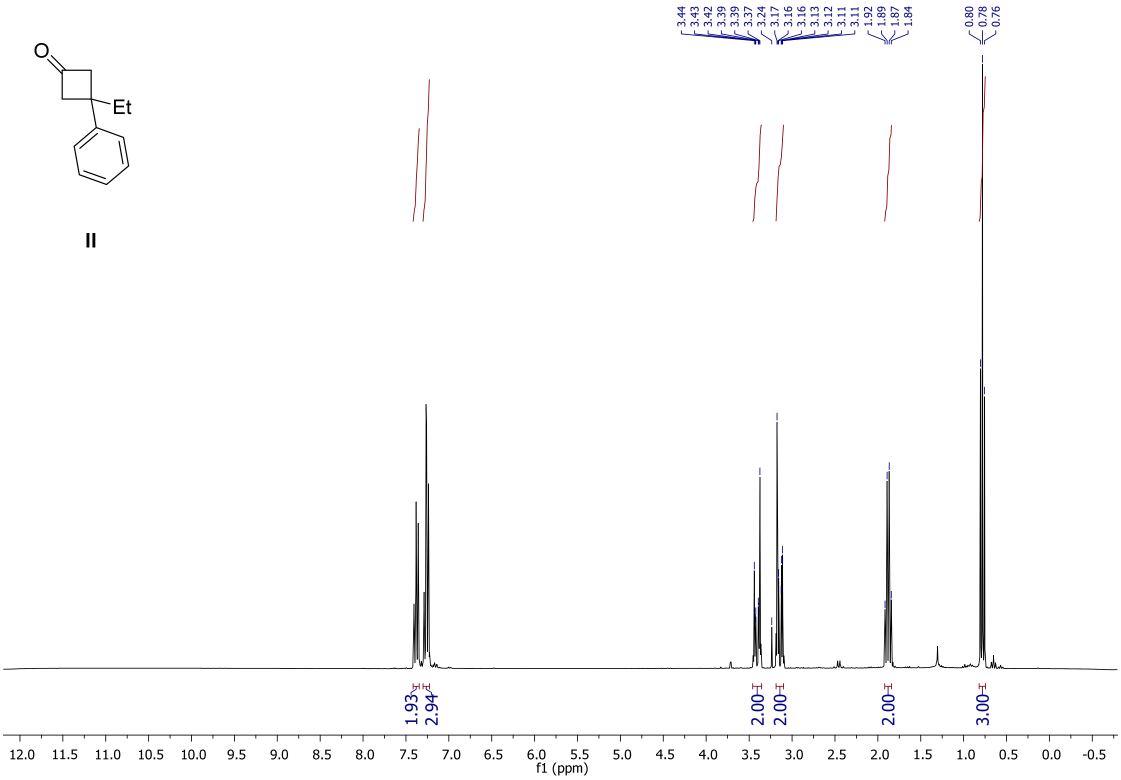

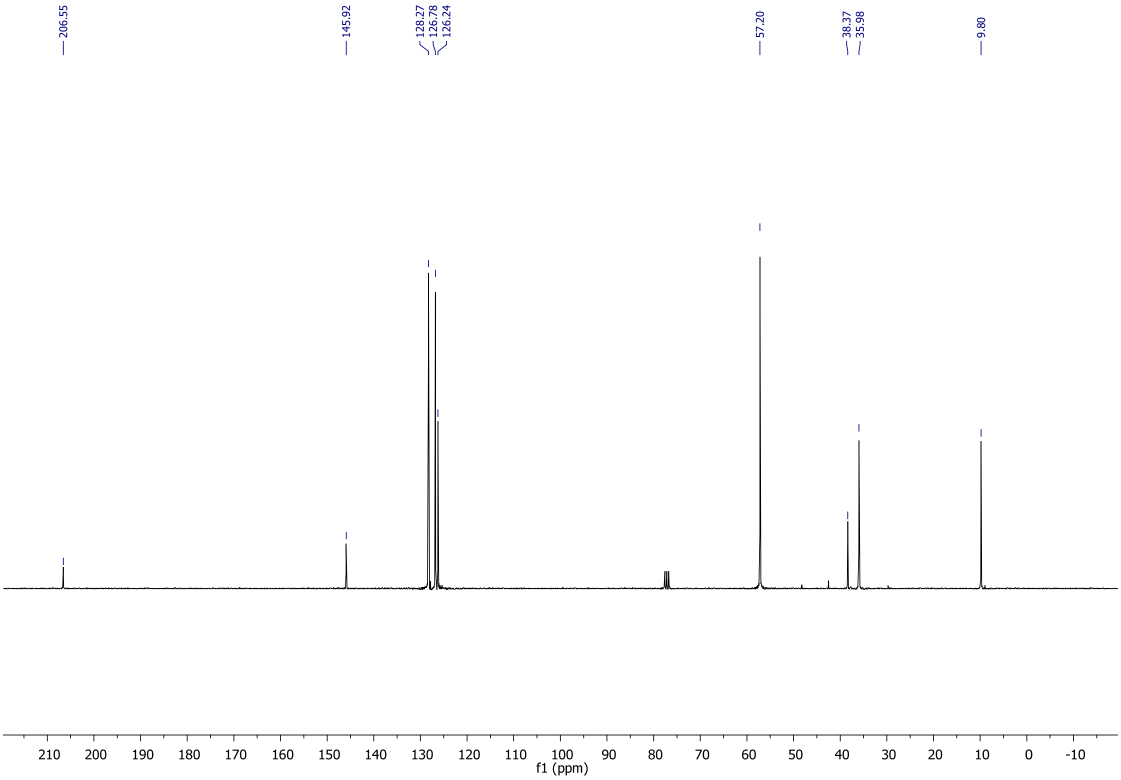

Figure S9. ^{1}H-NMR (300MHz, $CDCl_3$) and $^{13}C\{^{1}H\}$-NMR (75.5MHz, $CDCl_3$) of compound **II**

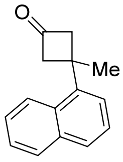

III

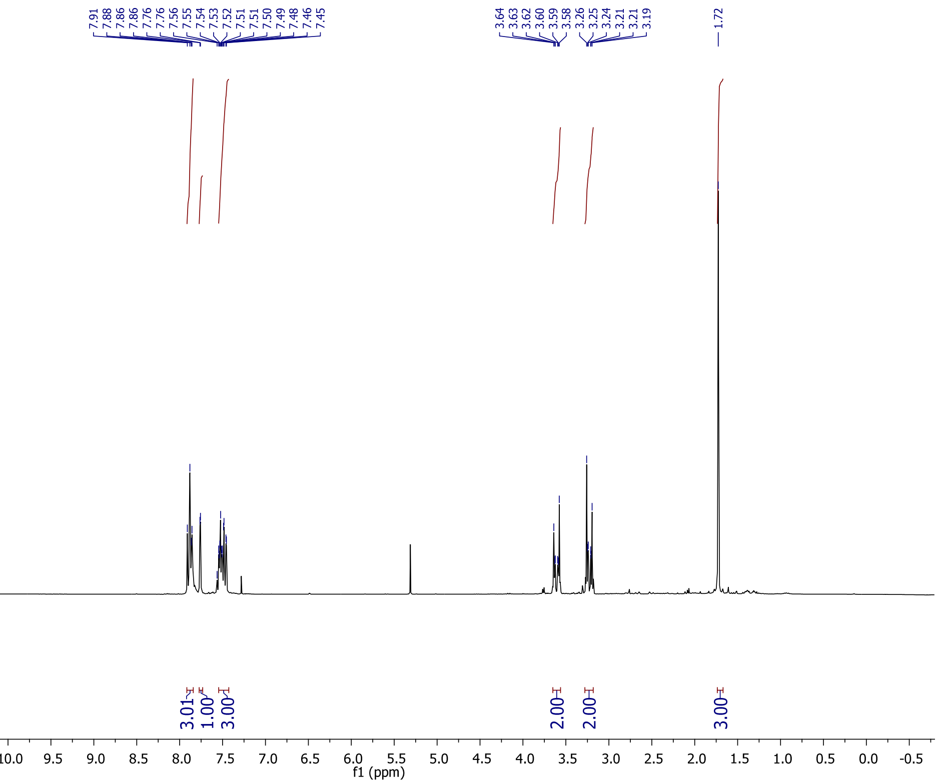

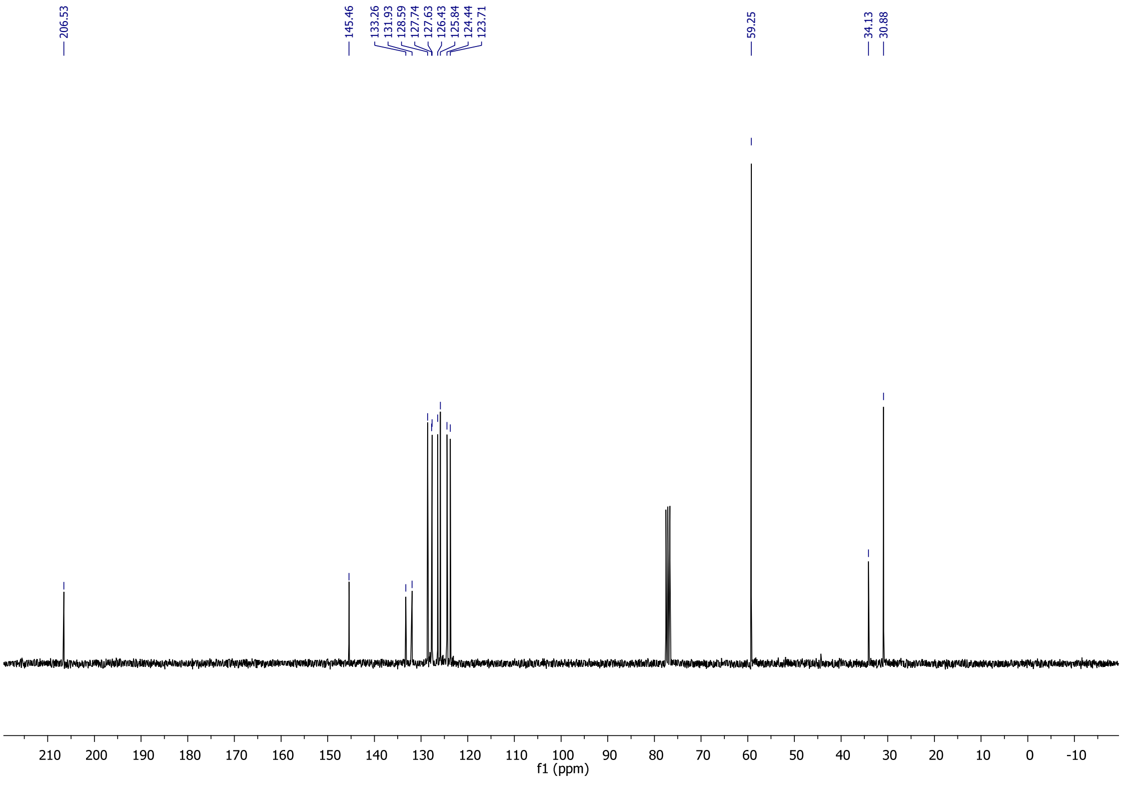

Figure S10. ^{1}H-NMR (300MHz, $CDCl_3$) and $^{13}C\{^{1}H\}$-NMR (75.5MHz, $CDCl_3$) of compound **III**

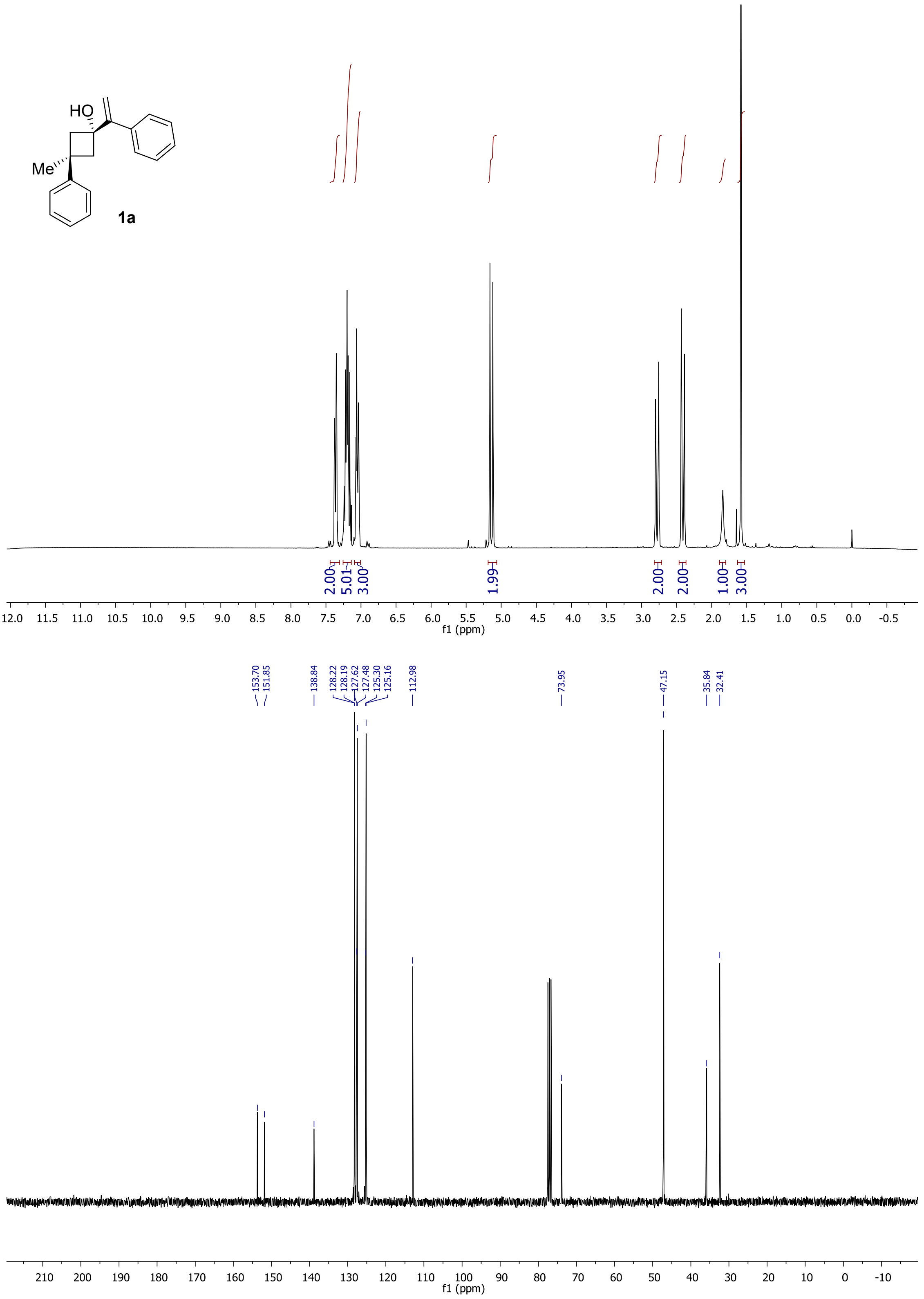

Figure S11. ^{1}H-NMR (300MHz, $CDCl_3$) and $^{13}C\{^{1}H\}$-NMR (75.5MHz, $CDCl_3$) of compound **1a**

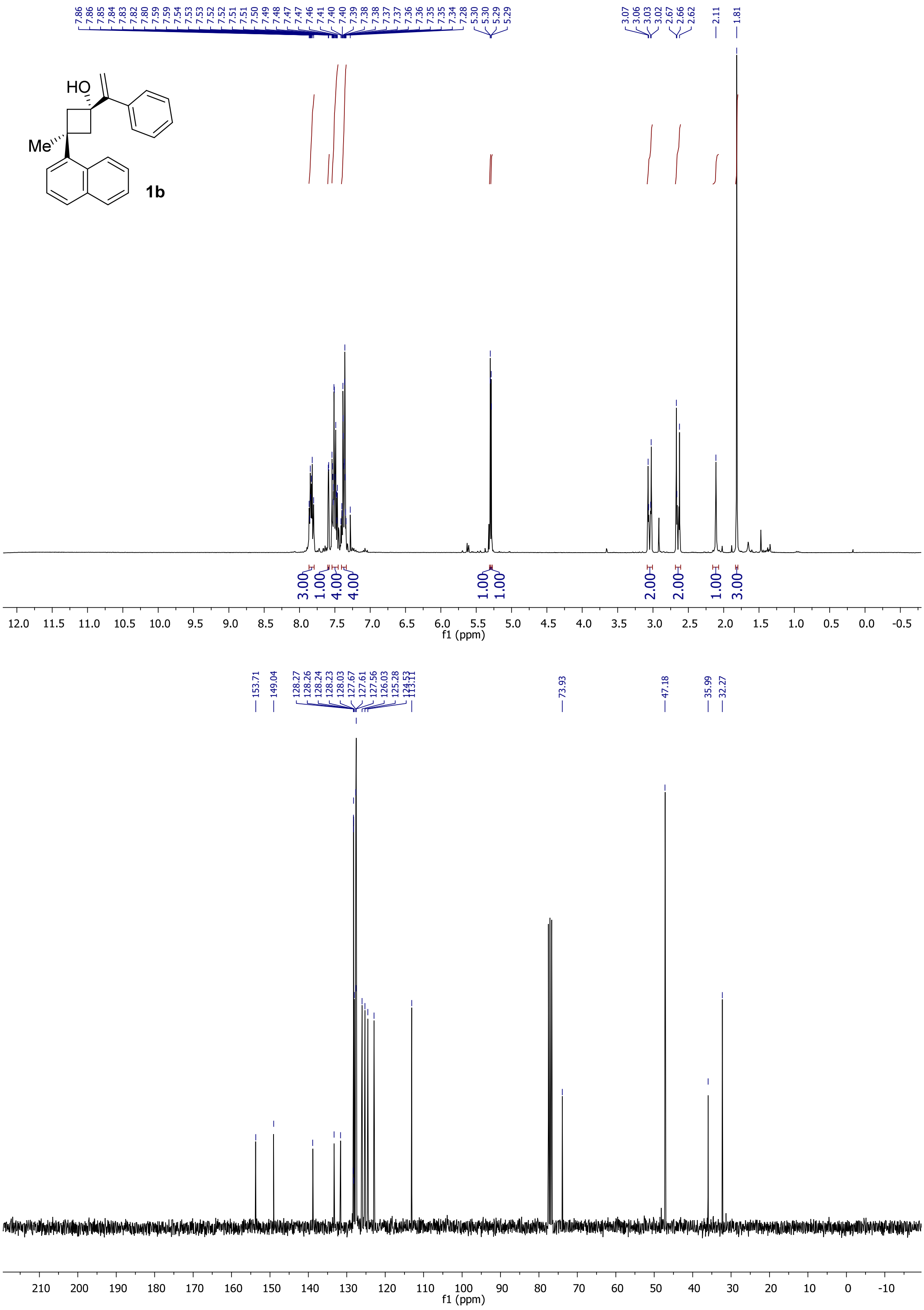

Figure S12. ^{1}H-NMR (300MHz, $CDCl_3$) and $^{13}C\{^{1}H\}$-NMR (75.5MHz, $CDCl_3$) of compound **1b**

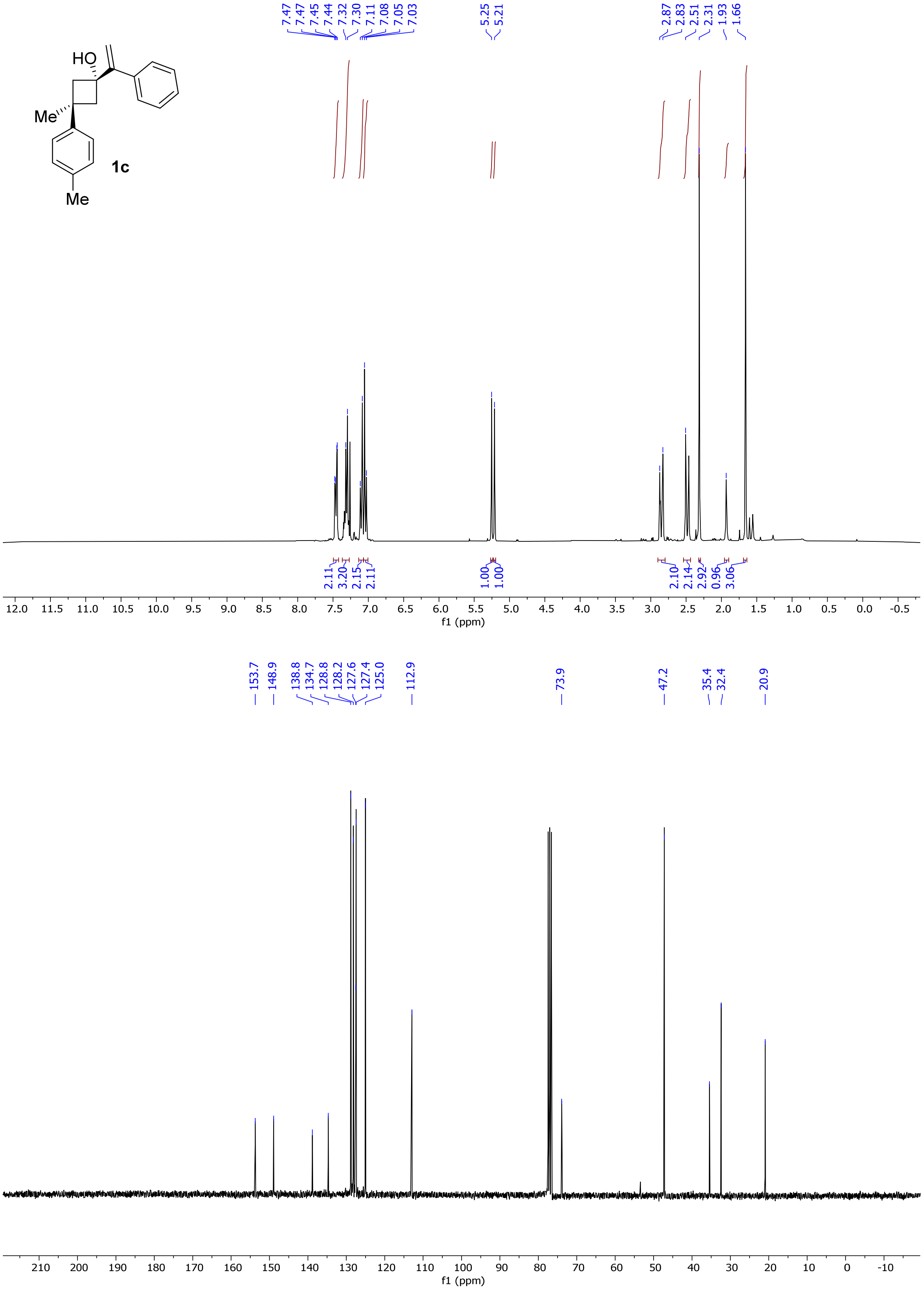

Figure S13. ^{1}H-NMR (300MHz, $CDCl_3$) and $^{13}C\{^{1}H\}$-NMR (75.5MHz, $CDCl_3$) of compound **1c**

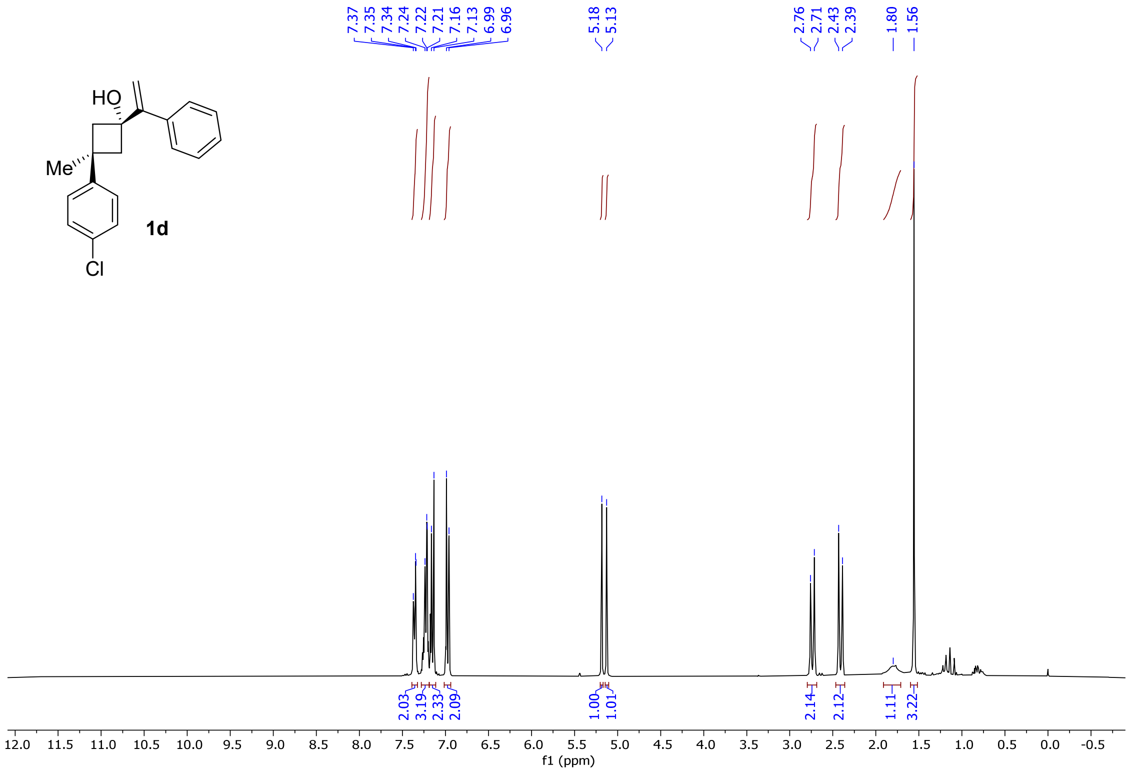

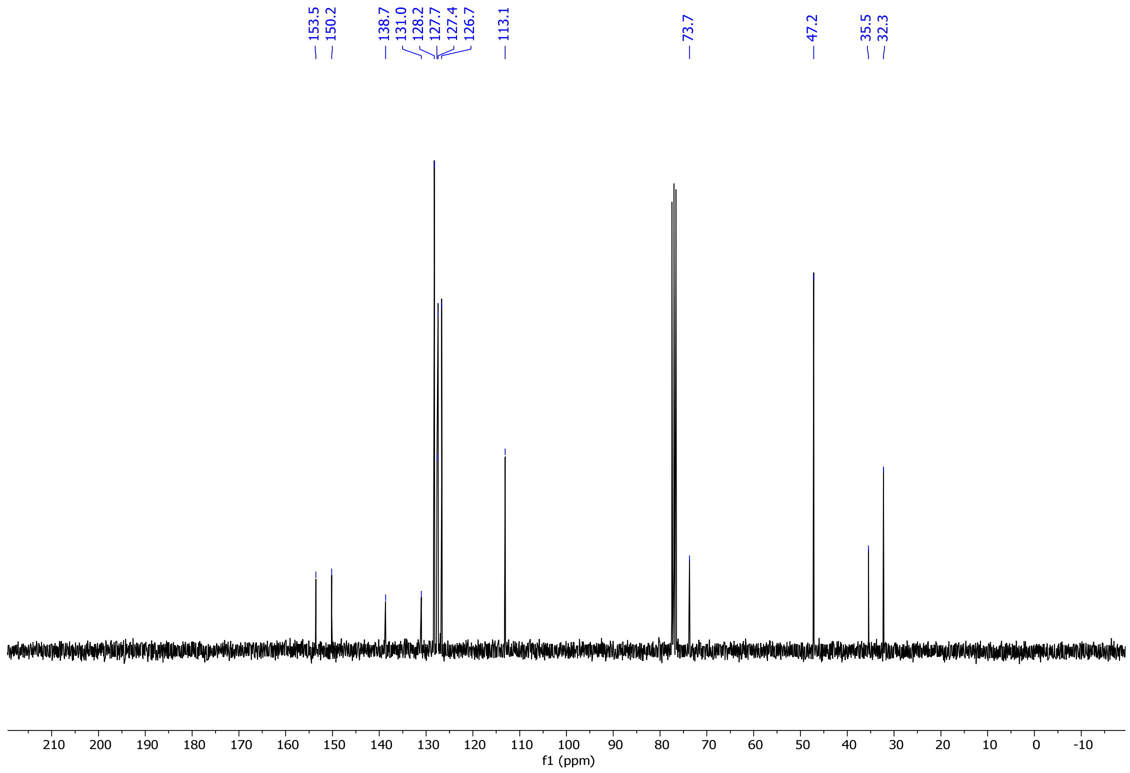

Figure S14. ^{1}H-NMR (300MHz, $CDCl_3$) and $^{13}C\{^{1}H\}$-NMR (75.5MHz, $CDCl_3$) of compound **1d**

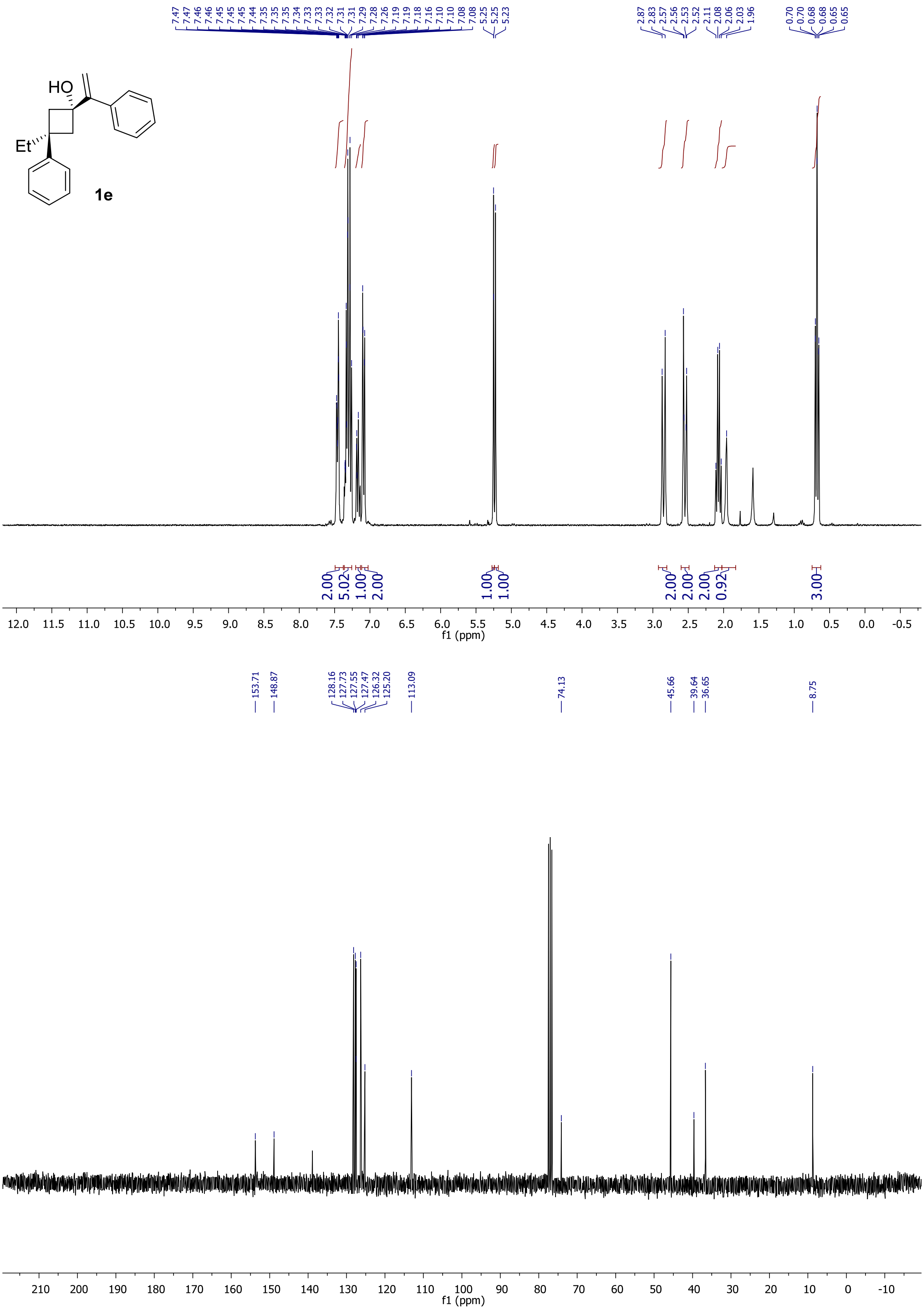

Figure S15. ^{1}H-NMR (300MHz, $CDCl_3$) and $^{13}C\{^{1}H\}$-NMR (75.5MHz, $CDCl_3$) of compound **1e**

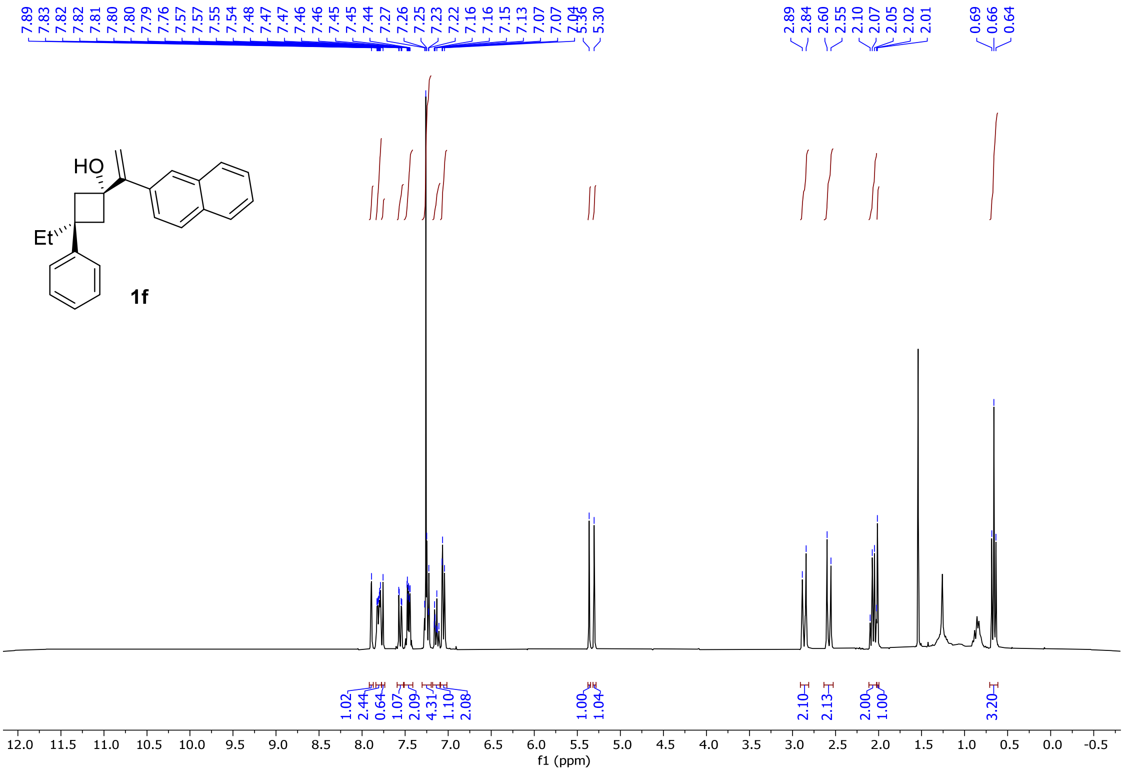

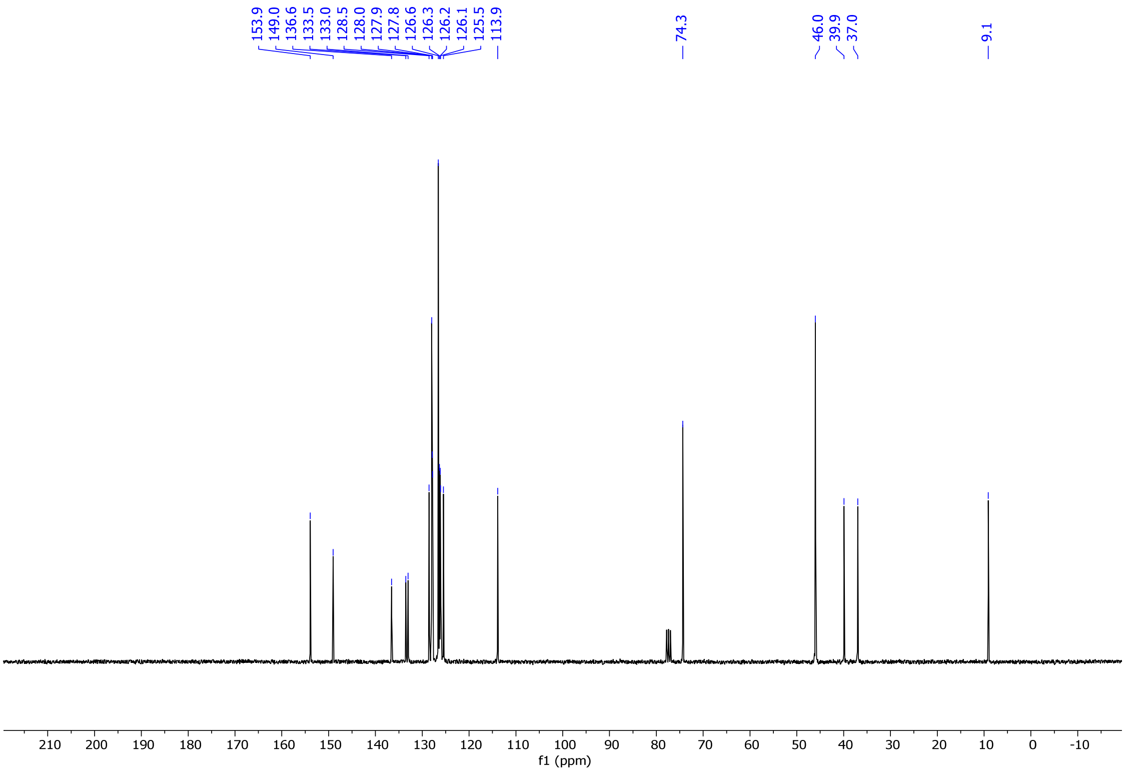

Figure S16. ^{1}H-NMR (300MHz, $CDCl_3$) and $^{13}C\{^{1}H\}$-NMR (75.5MHz, $CDCl_3$) of compound **1f**

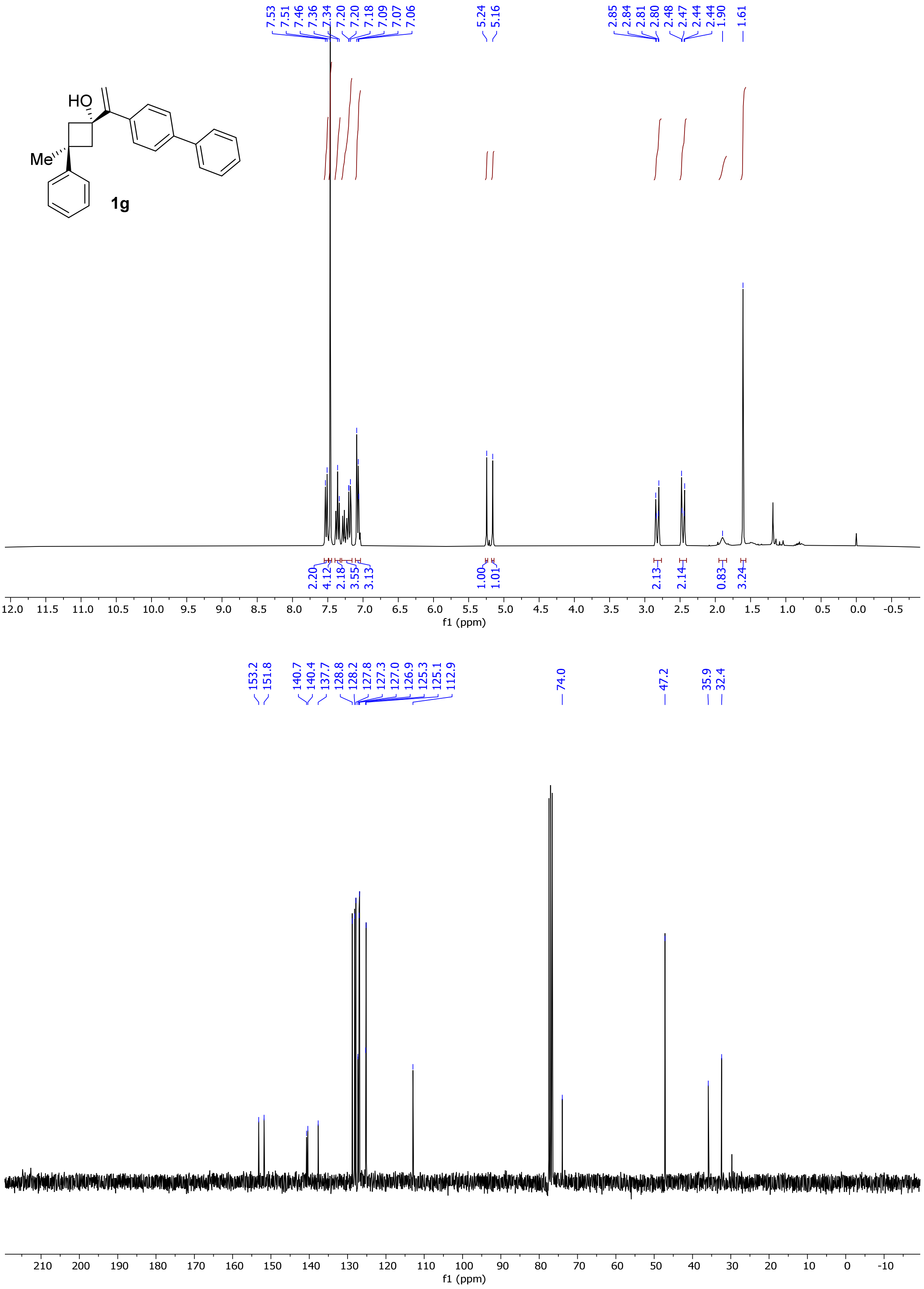

Figure S17. ^{1}H-NMR (300MHz, $CDCl_3$) and $^{13}C\{^{1}H\}$-NMR (75.5MHz, $CDCl_3$) of compound **1g**

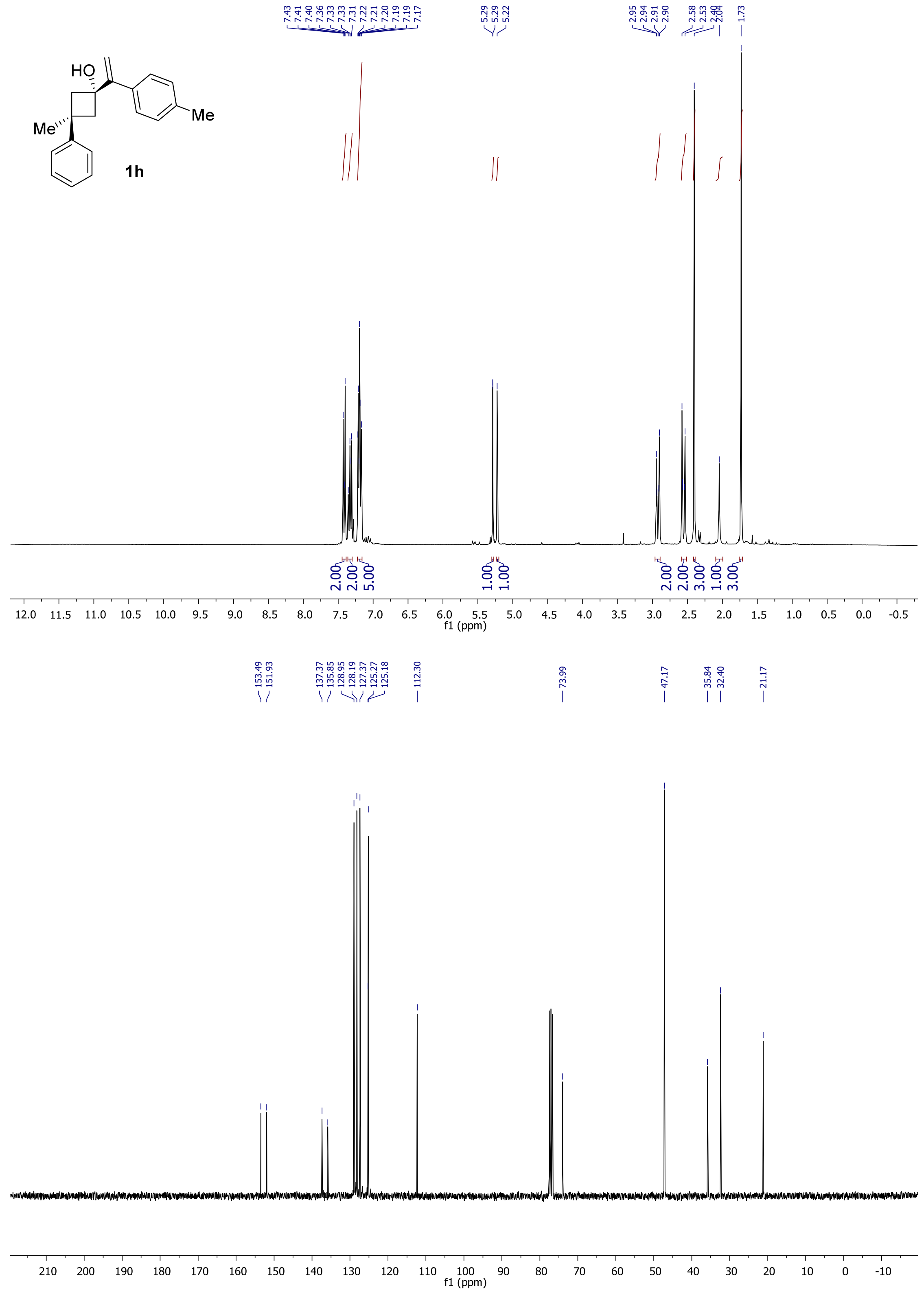

Figure S18. ^{1}H-NMR (300MHz, $CDCl_3$) and $^{13}C\{^{1}H\}$-NMR (75.5MHz, $CDCl_3$) of compound **1h**

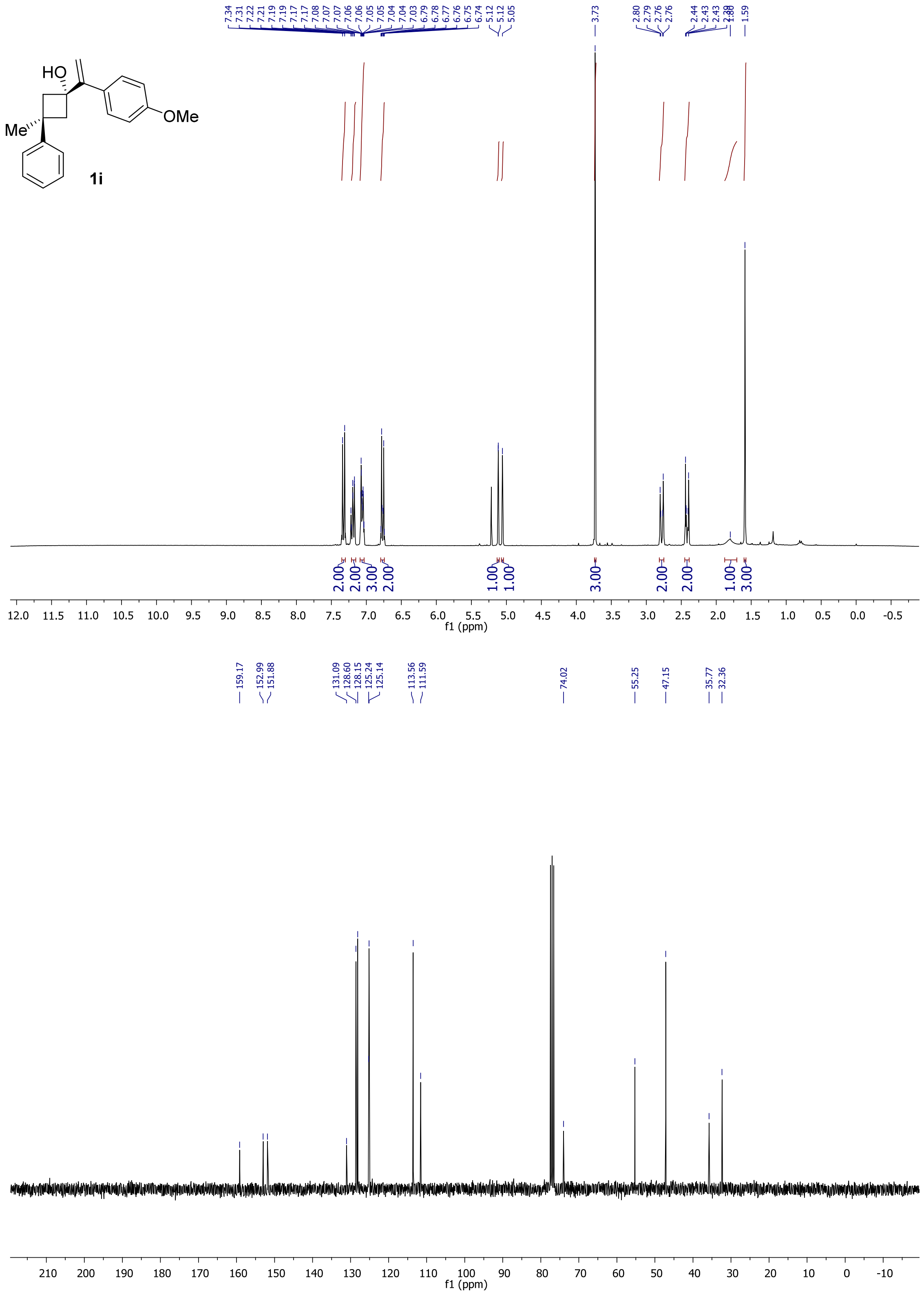

Figure S19. ^{1}H-NMR (300MHz, $CDCl_3$) and $^{13}C\{^{1}H\}$-NMR (75.5MHz, $CDCl_3$) of compound **1i**

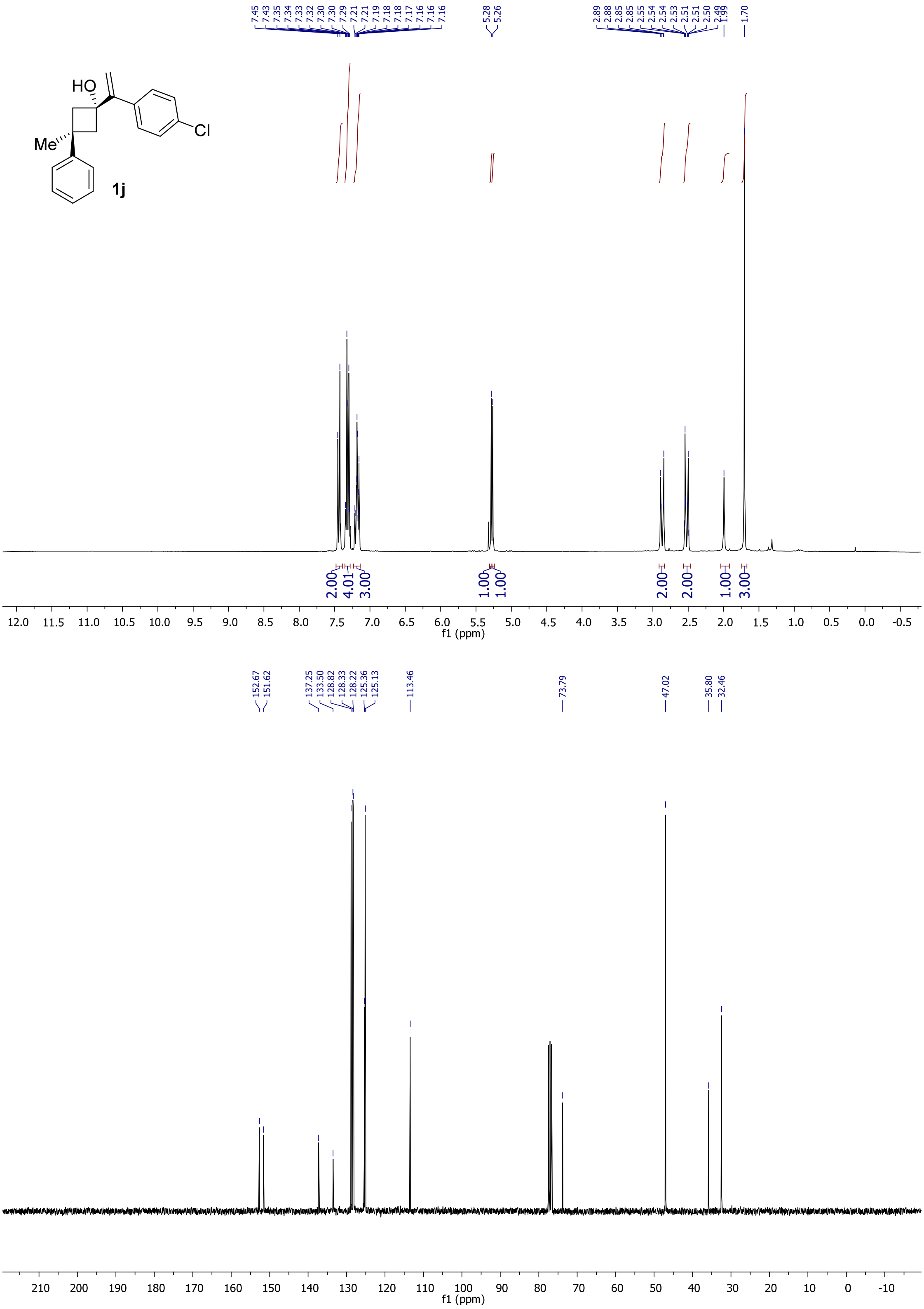

Figure S20. ^{1}H-NMR (300MHz, $CDCl_3$) and $^{13}C\{^1H\}$-NMR (75.5MHz, $CDCl_3$) of compound **1j**

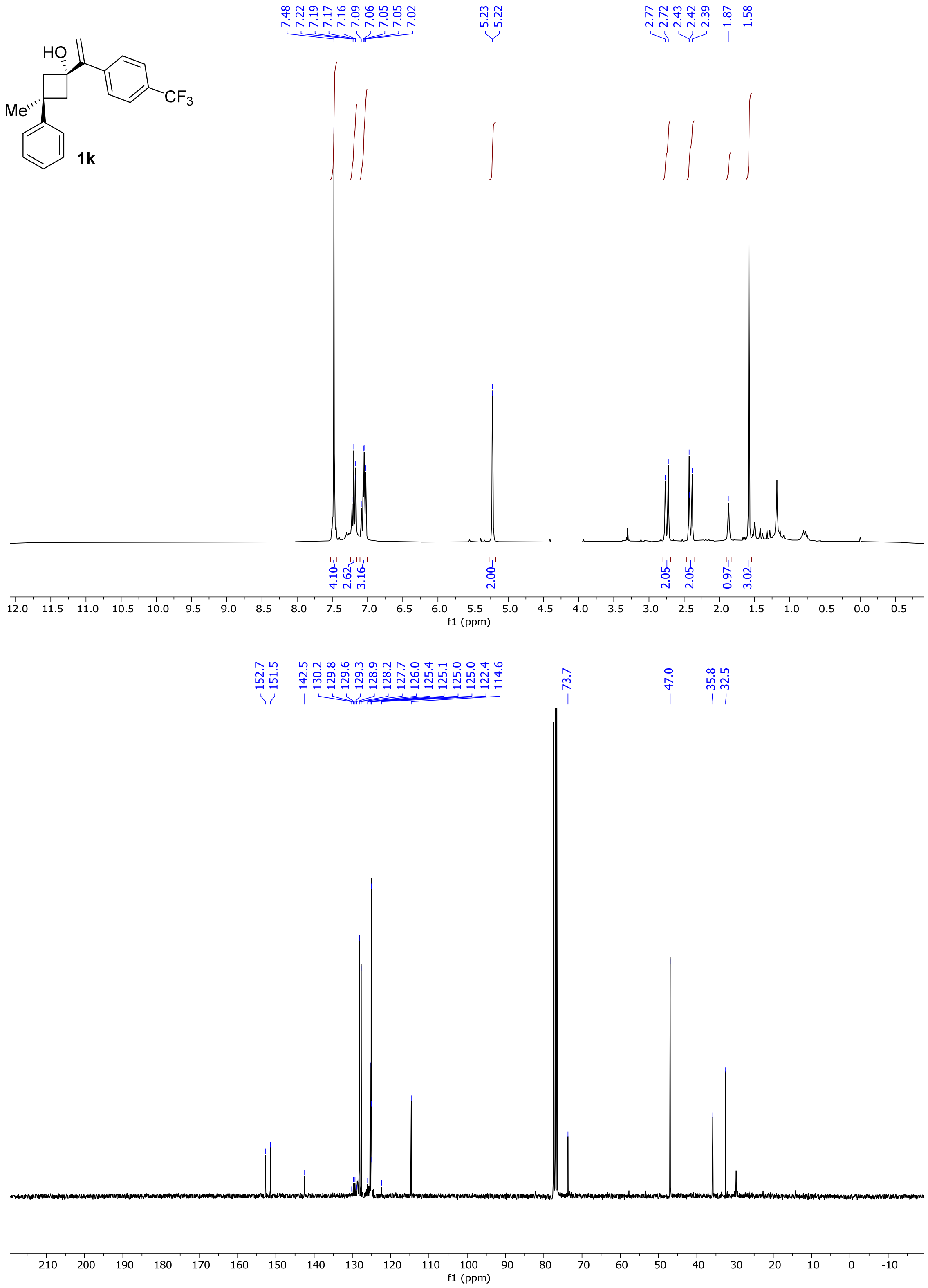

Figure S21. ^{1}H-NMR (300MHz, $CDCl_3$) and $^{13}C\{^{1}H\}$-NMR (75.5MHz, $CDCl_3$) of compound **1k**

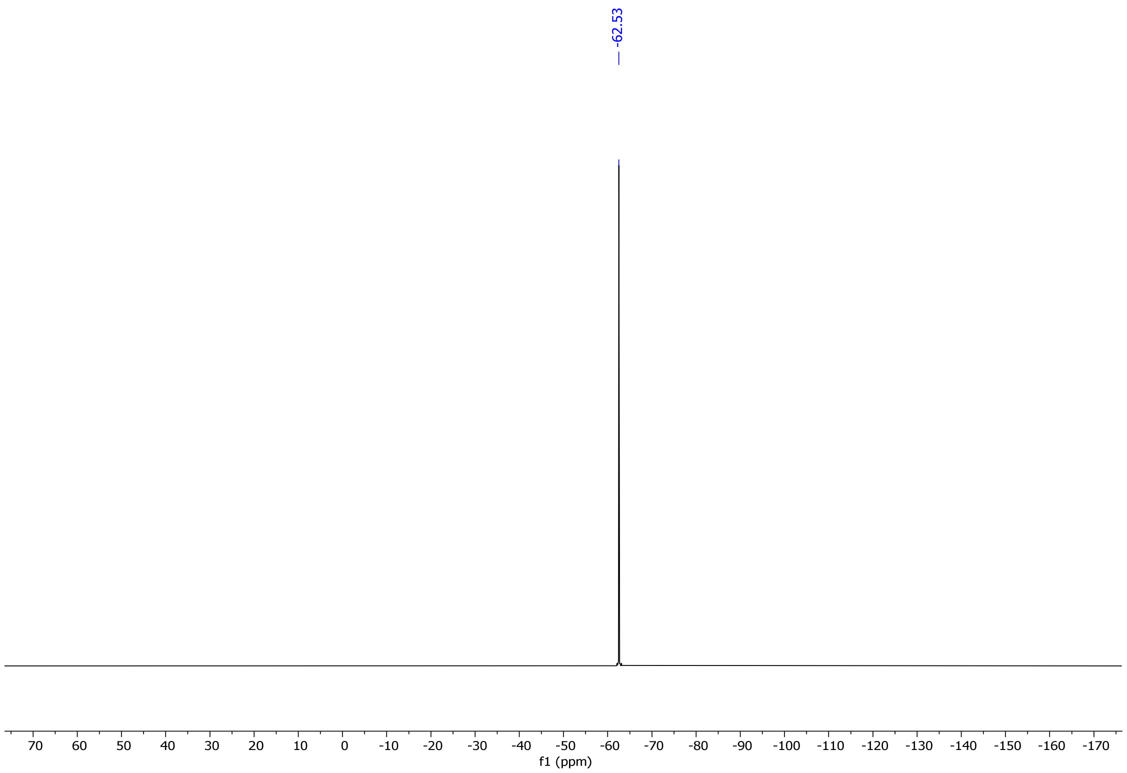

Figure S22. ^{31}P-NMR (122MHz, $CDCl_3$) of compound **1k**

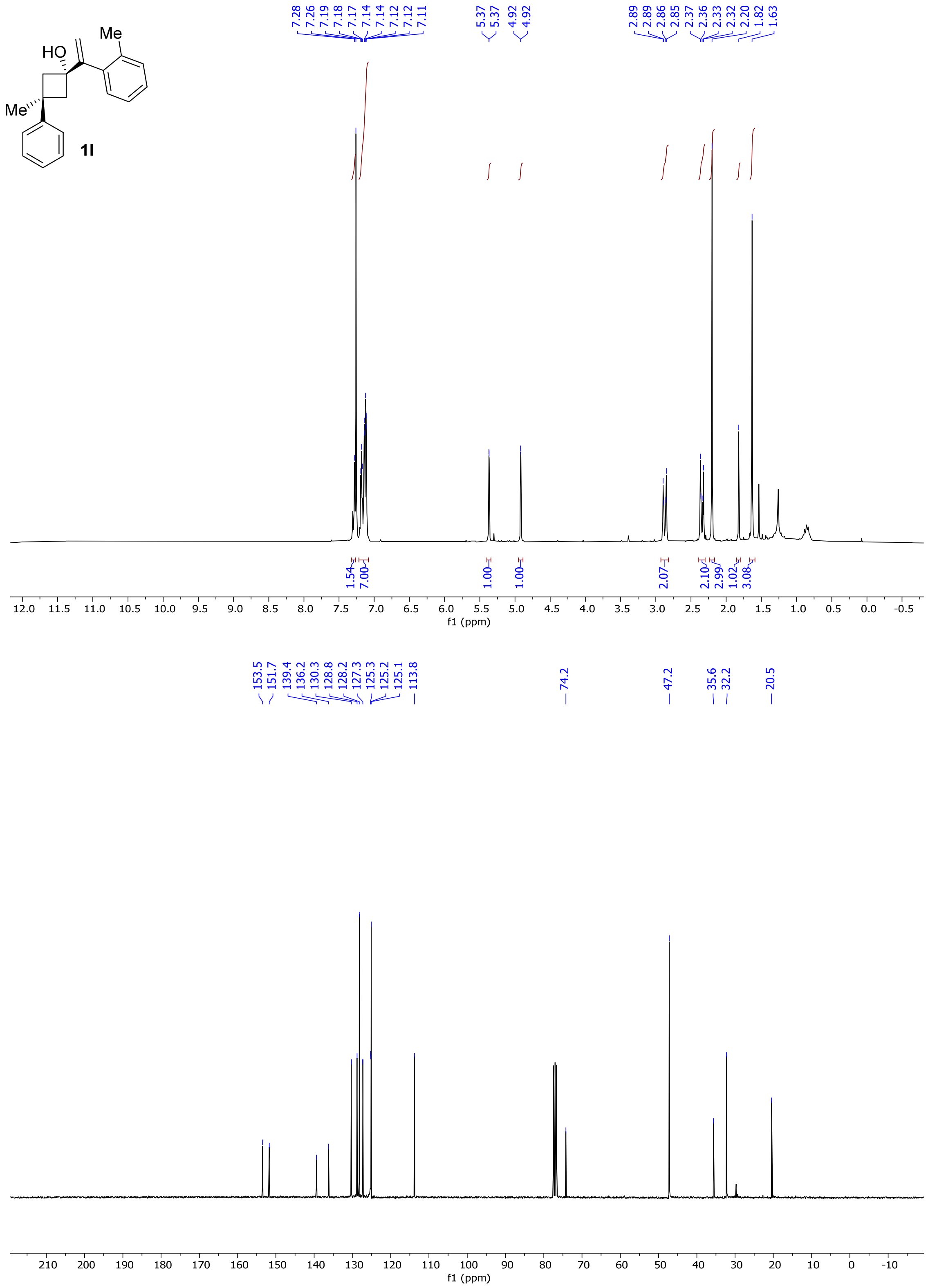

Figure S23. ^{1}H-NMR (300MHz, $CDCl_3$) and $^{13}C\{^{1}H\}$-NMR (75.5MHz, $CDCl_3$) of compound **1l**

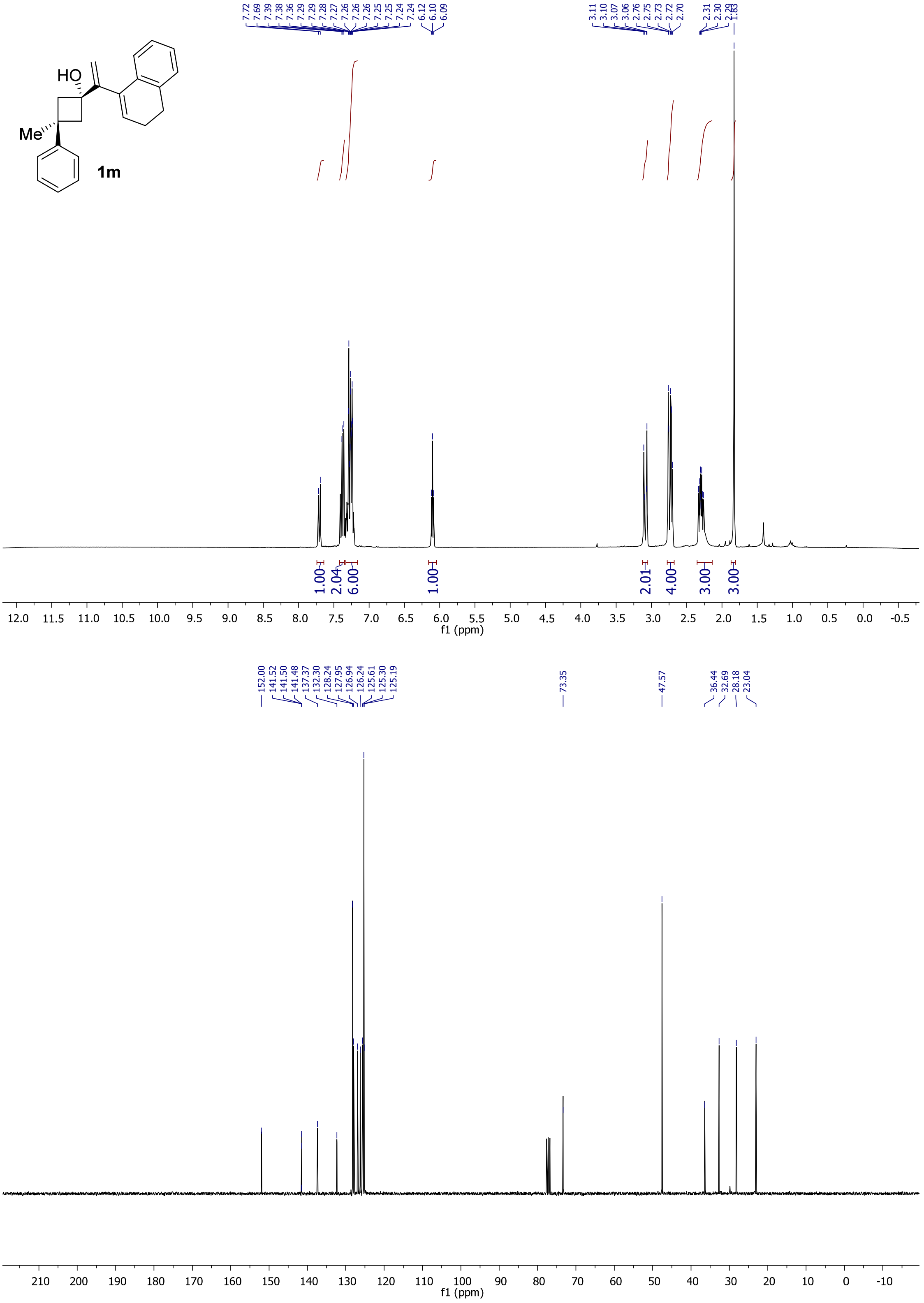

Figure S24. ^{1}H-NMR (300MHz, $CDCl_3$) and $^{13}C\{^{1}H\}$-NMR (75.5MHz, $CDCl_3$) of compound **1m**

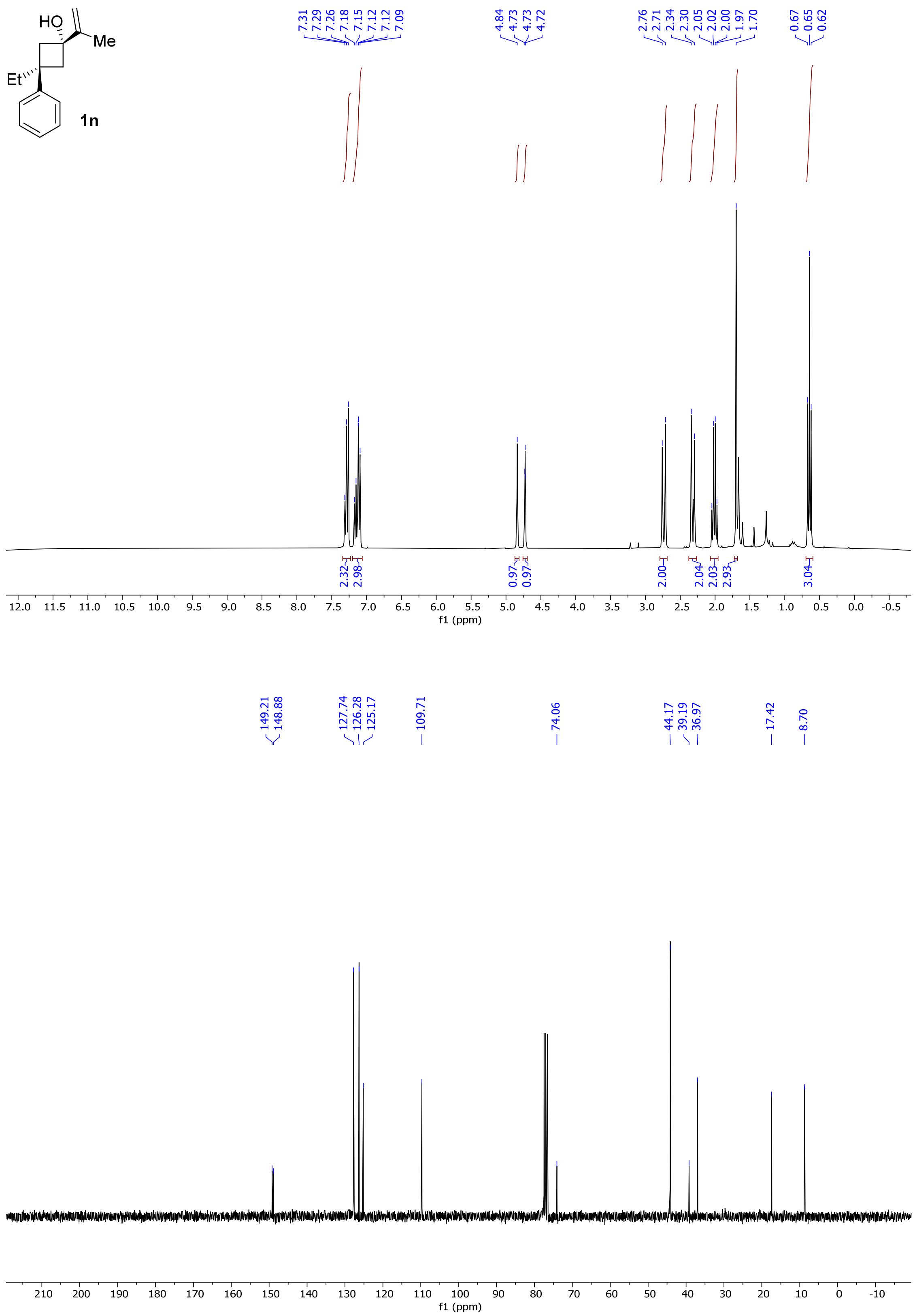

Figure S25. ^{1}H-NMR (300MHz, $CDCl_3$) and $^{13}C\{^{1}H\}$-NMR (75.5MHz, $CDCl_3$) of compound **1n**

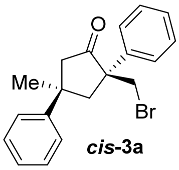

cis-3a

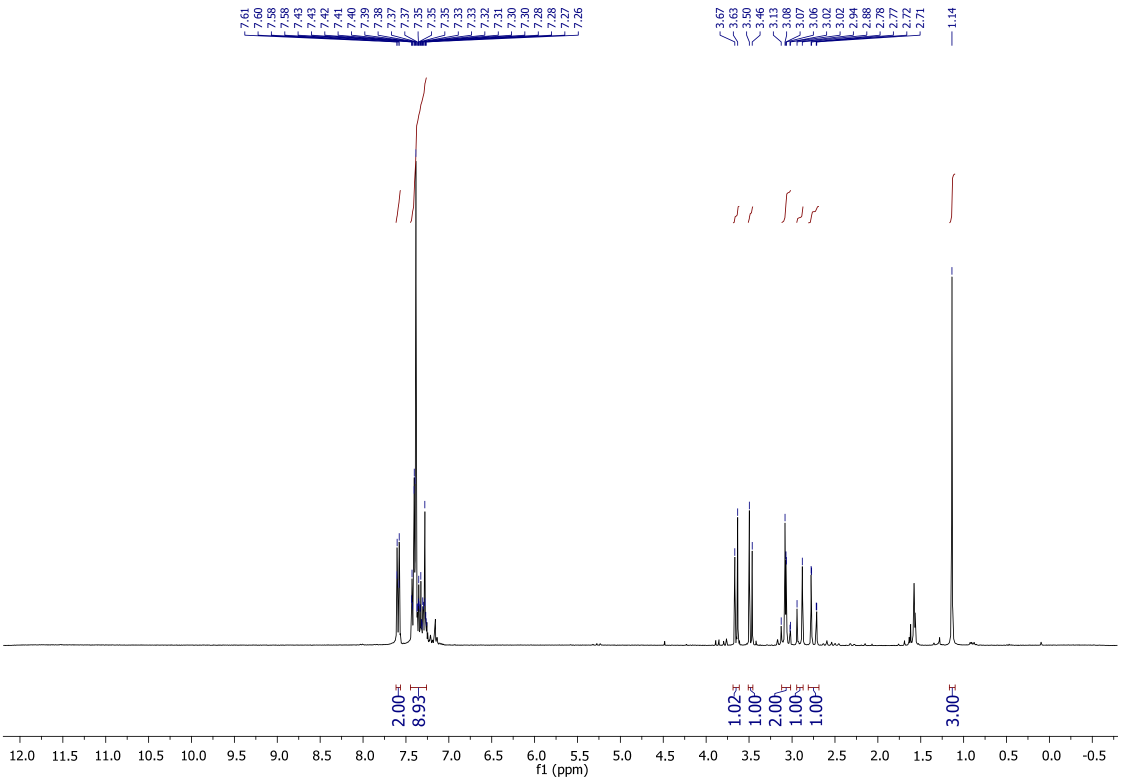

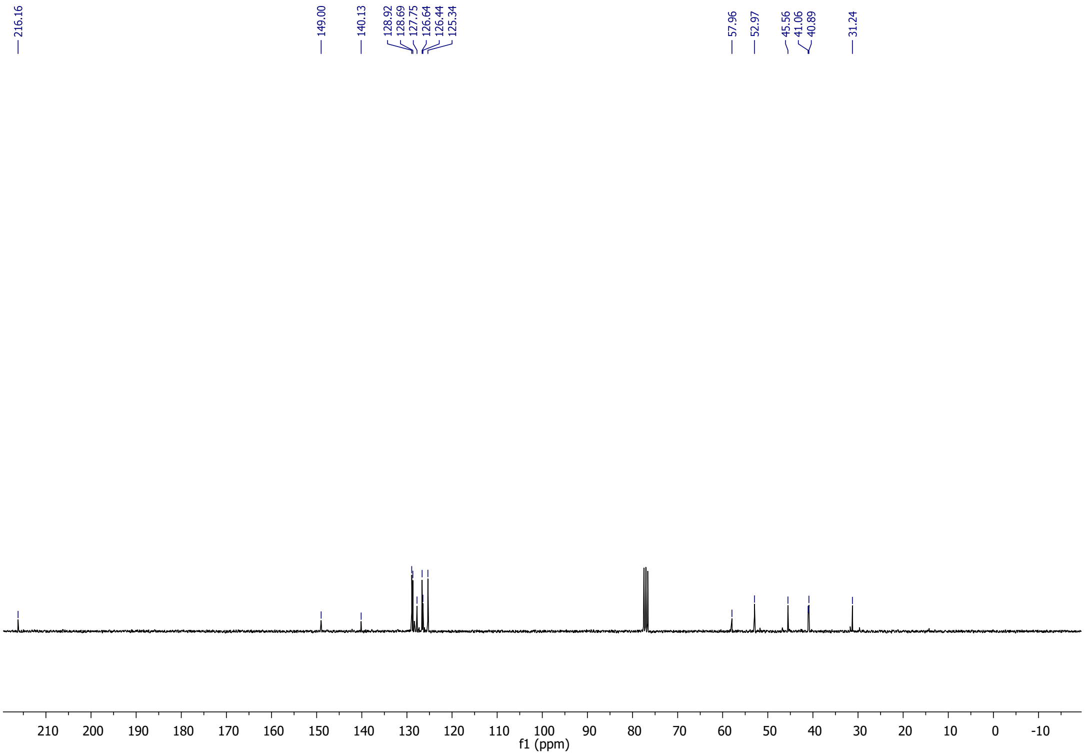

Figure S26. ^{1}H-NMR (300MHz, $CDCl_3$) and $^{13}C\{^{1}H\}$-NMR (75.5MHz, $CDCl_3$) of compound **cis-3a**

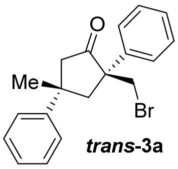

***trans*-3a**

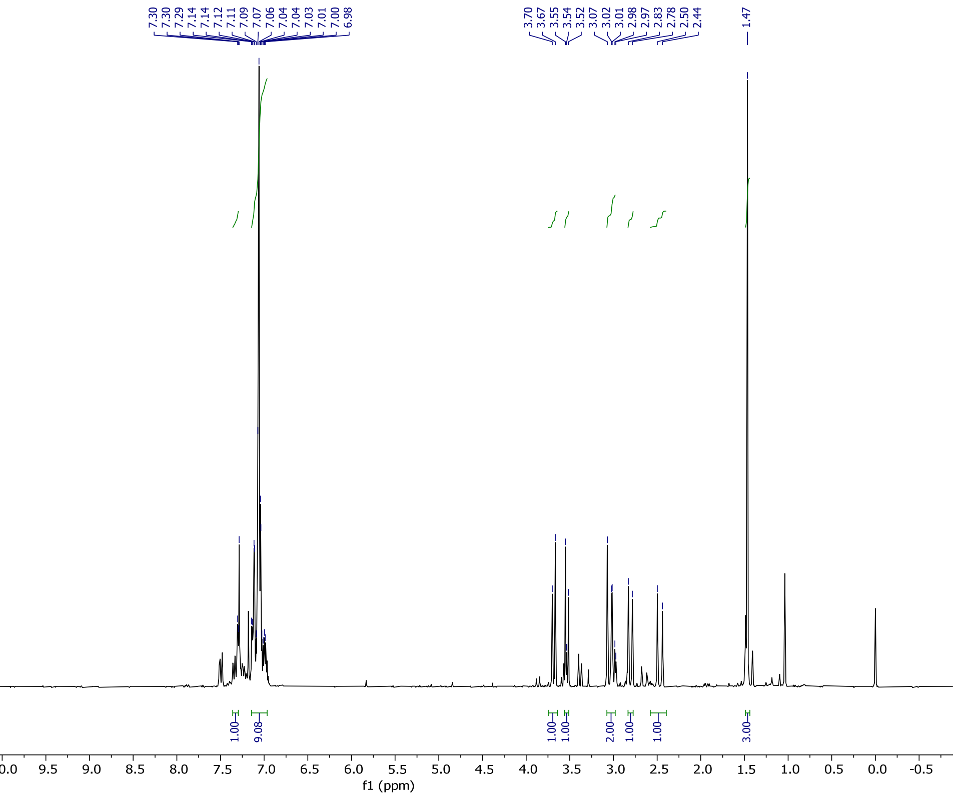

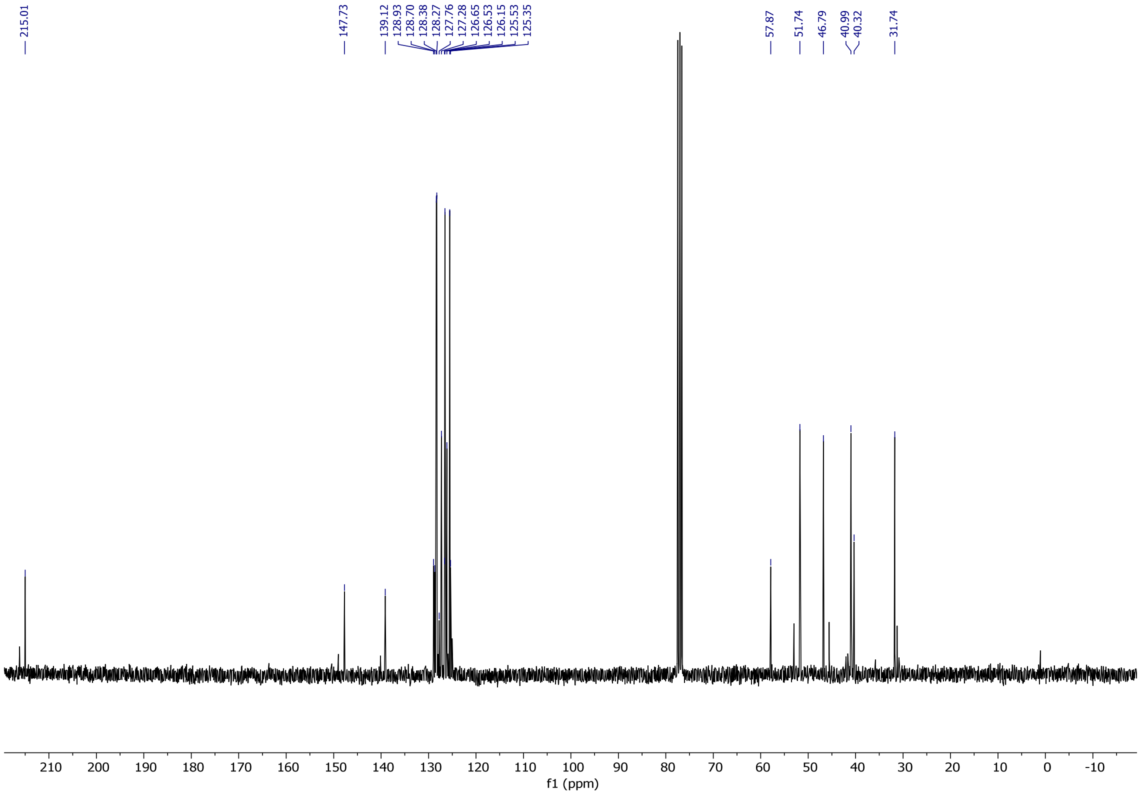

Figure S27. ^{1}H-NMR (300MHz, $CDCl_3$) and $^{13}C\{^{1}H\}$-NMR (75.5MHz, $CDCl_3$) of compound **trans-3a**

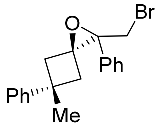

4a

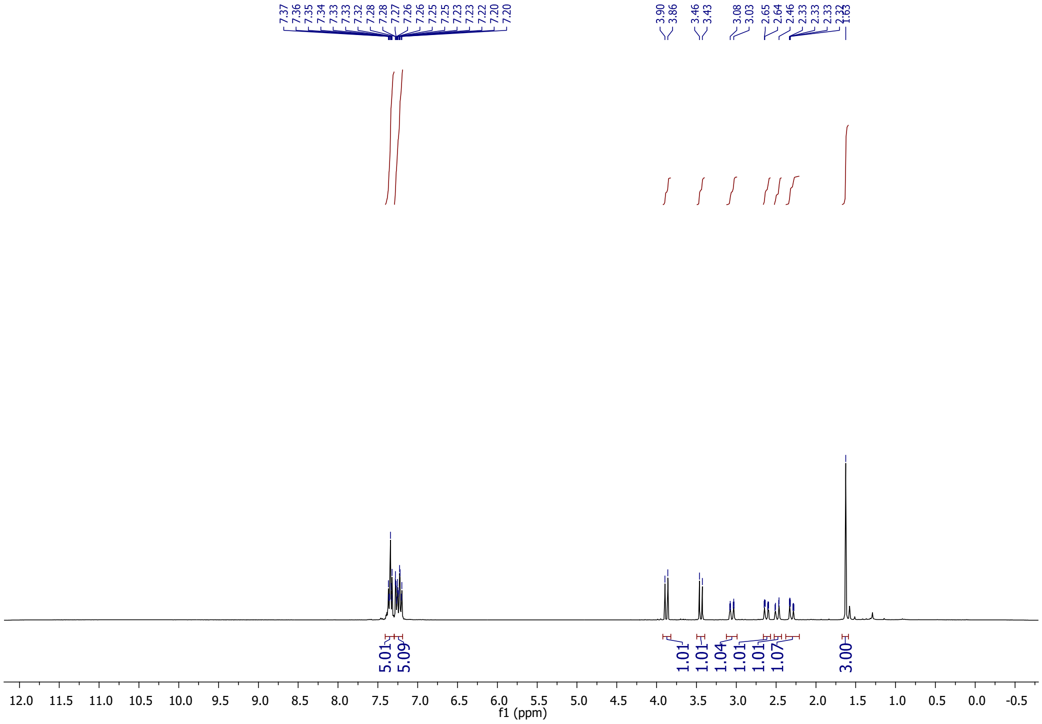

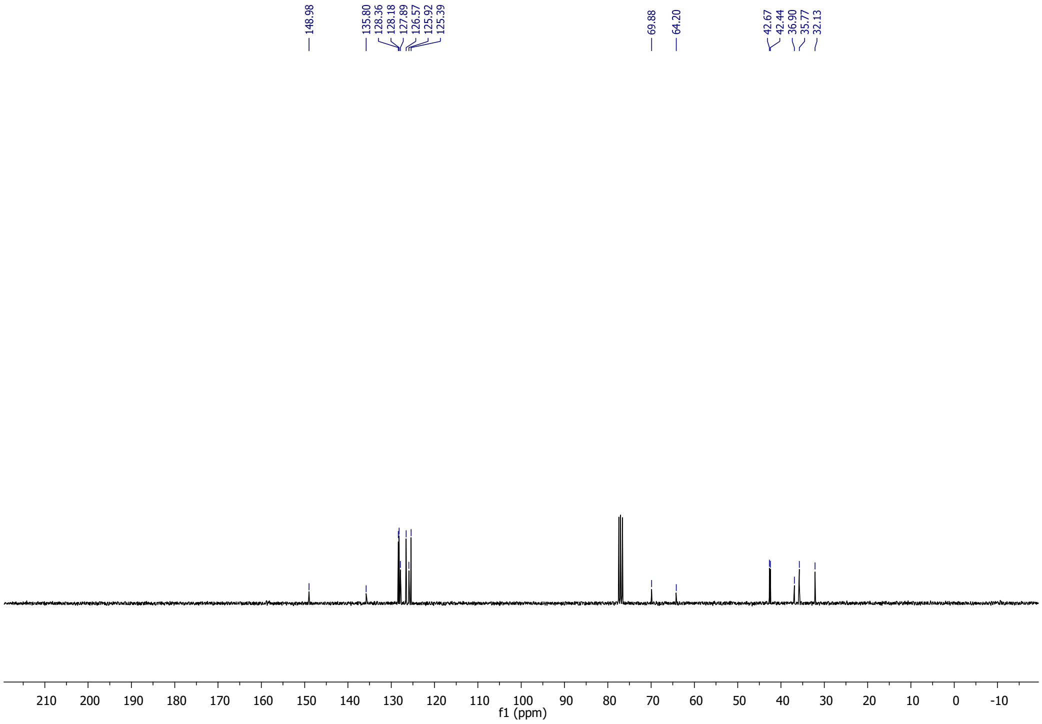

Figure S28. ^{1}H-NMR (300MHz, $CDCl_3$) and $^{13}C\{^{1}H\}$-NMR (75.5MHz, $CDCl_3$) of compound **4a**

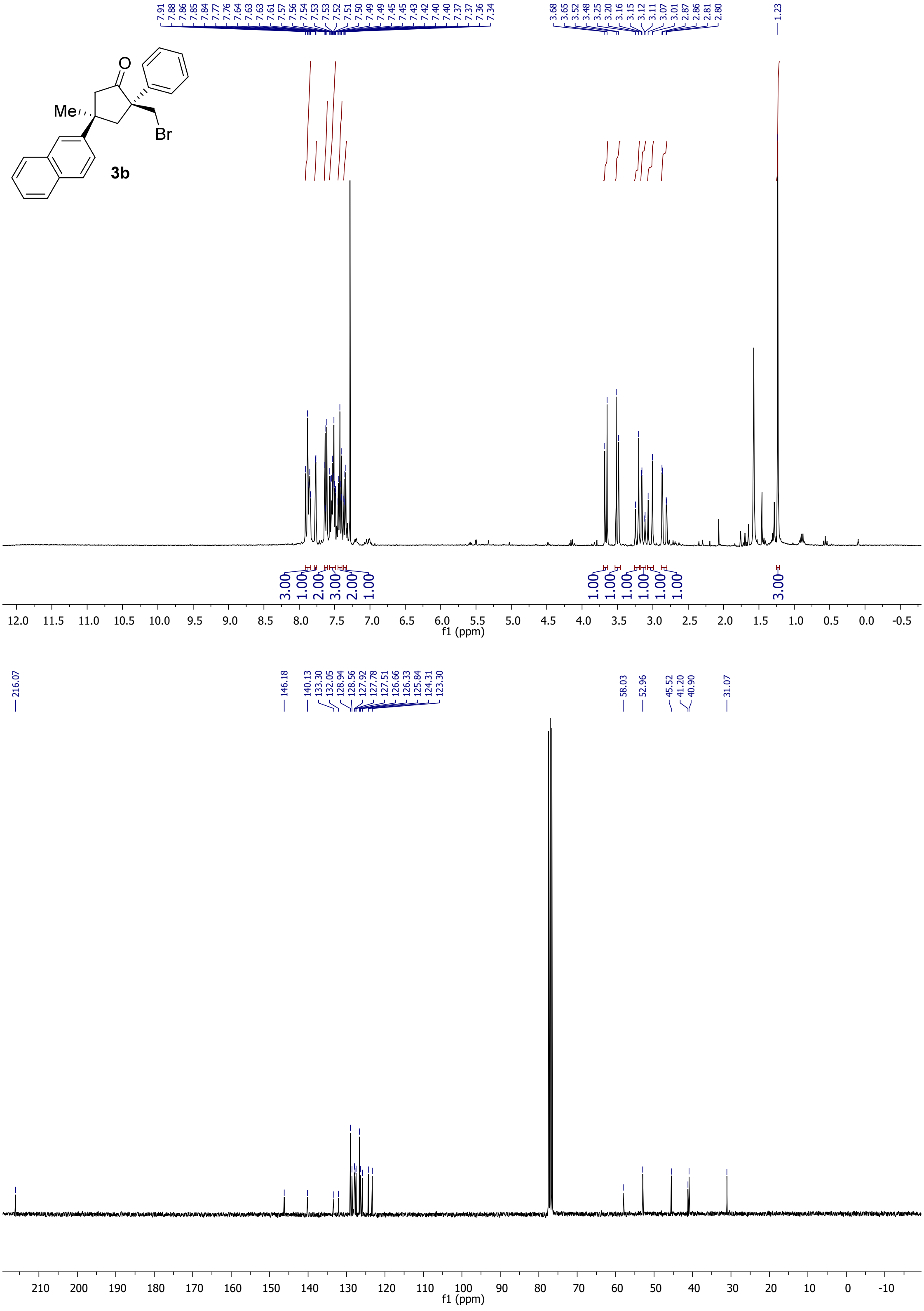

Figure S29. ^{1}H-NMR (300MHz, $CDCl_3$) and $^{13}C\{^{1}H\}$-NMR (75.5MHz, $CDCl_3$) of compound **3b**

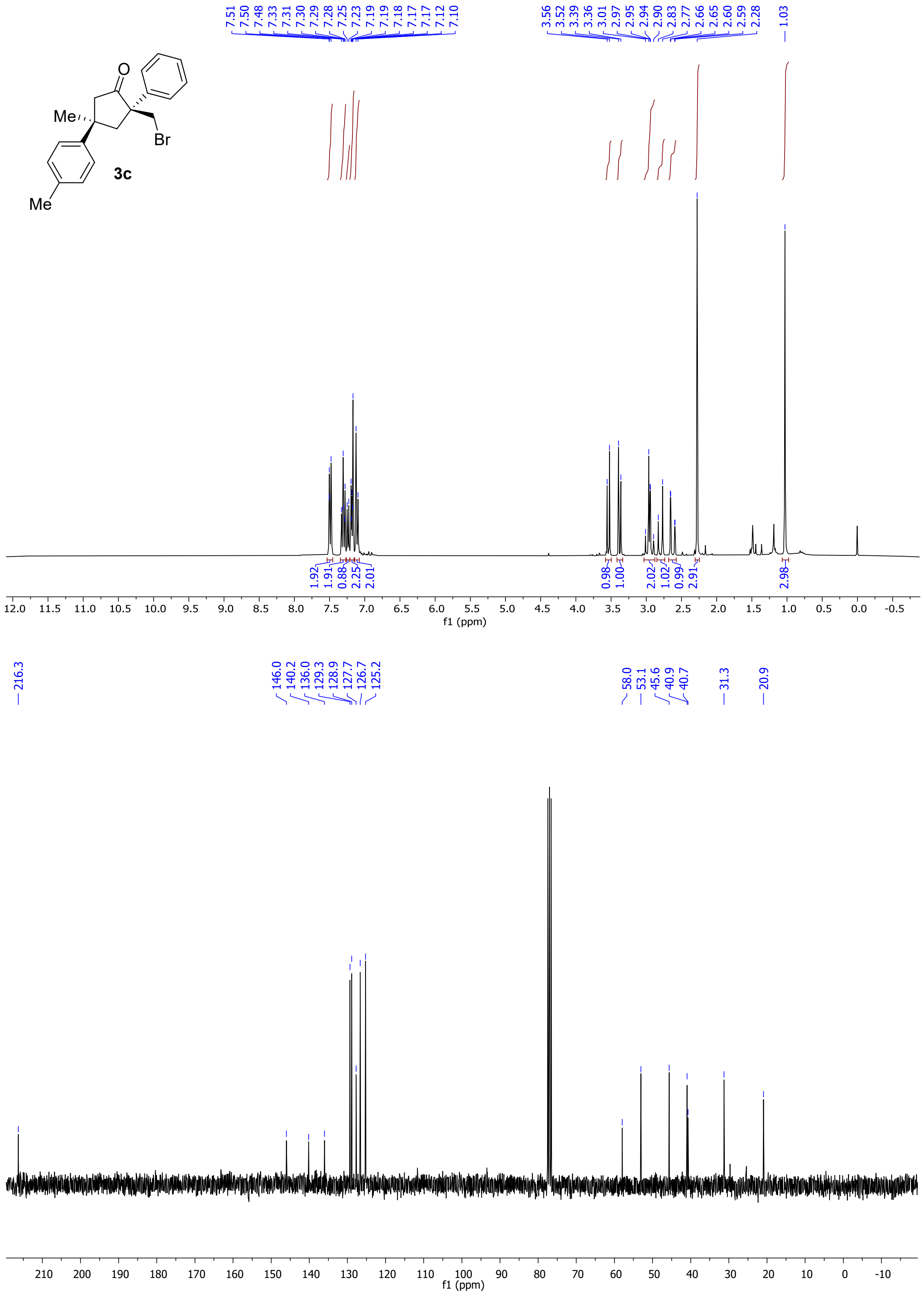

Figure S30. ^{1}H-NMR (300MHz, $CDCl_3$) and $^{13}C\{^{1}H\}$-NMR (75.5MHz, $CDCl_3$) of compound **3c**

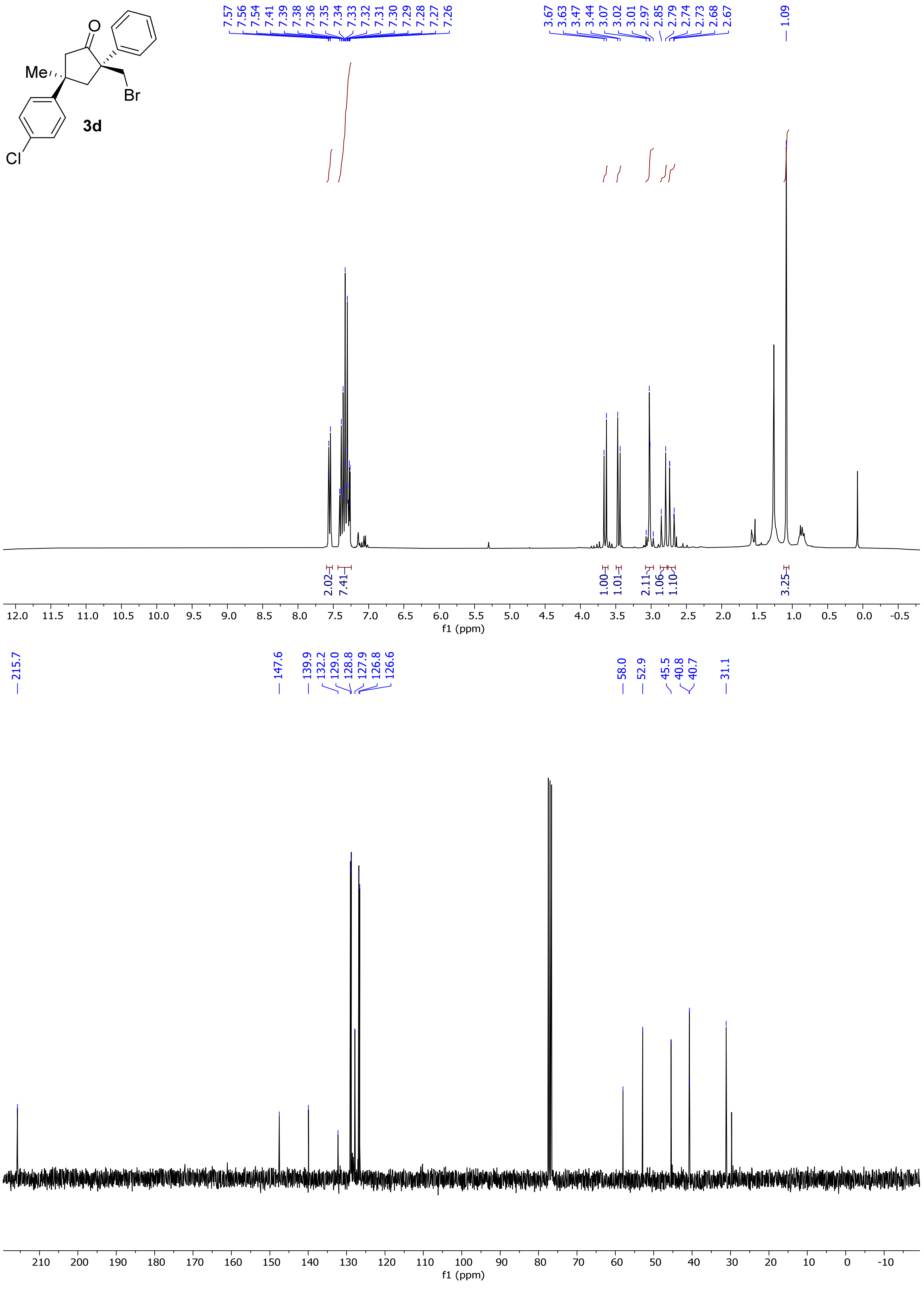

Figure S31. ^{1}H-NMR (300MHz, $CDCl_3$) and $^{13}C\{^{1}H\}$-NMR (75.5MHz, $CDCl_3$) of compound **3d**

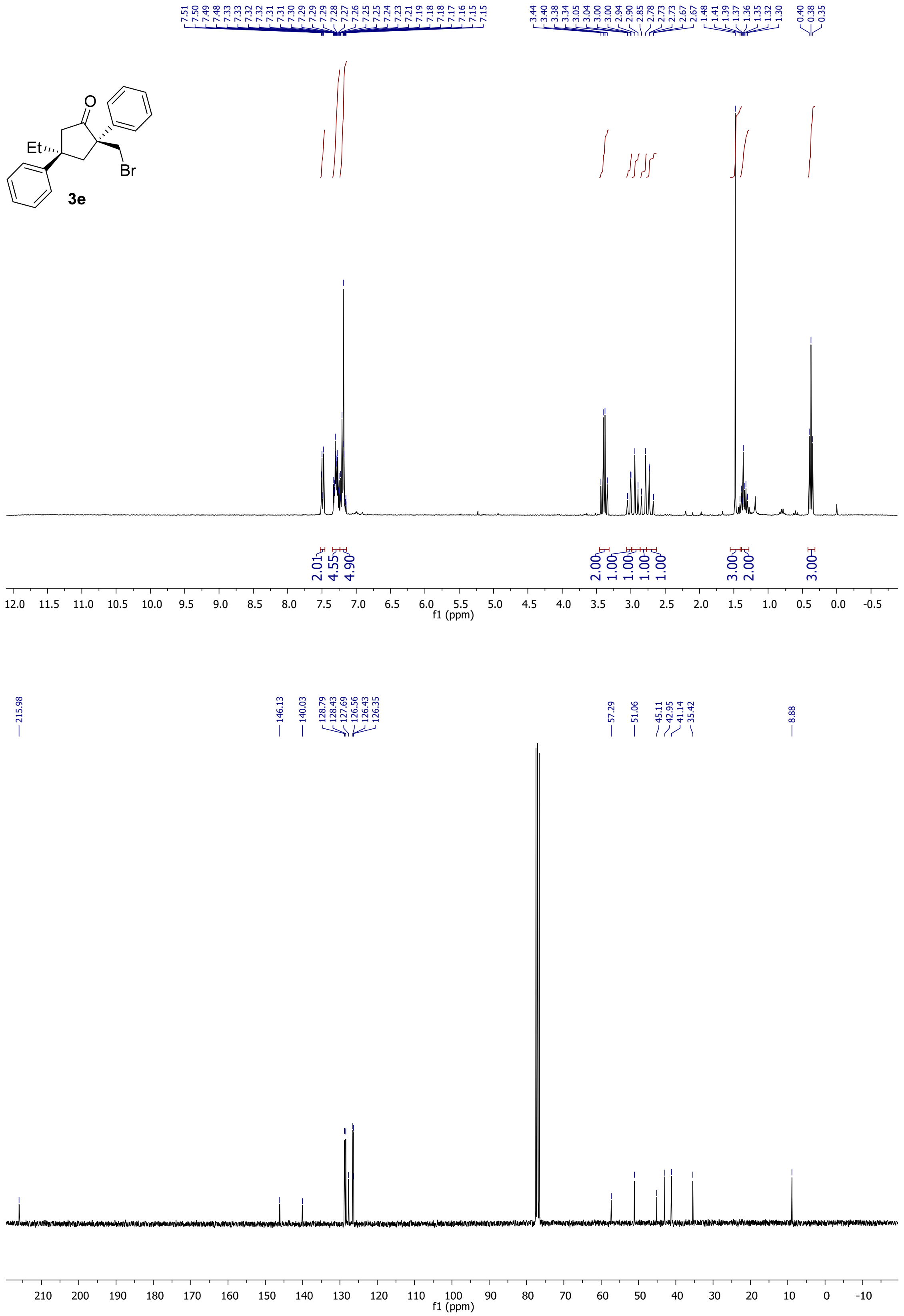

Figure S32. ^{1}H-NMR (300MHz, $CDCl_3$) and $^{13}C\{^{1}H\}$-NMR (75.5MHz, $CDCl_3$) of compound **3e**

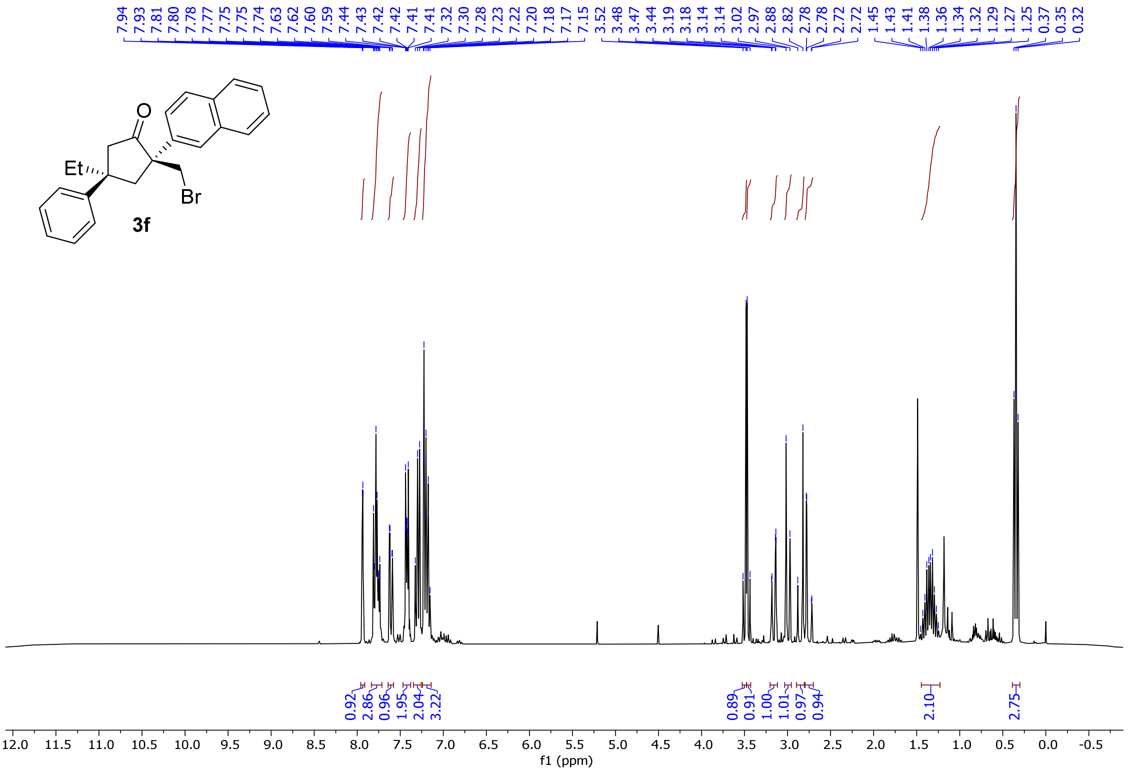

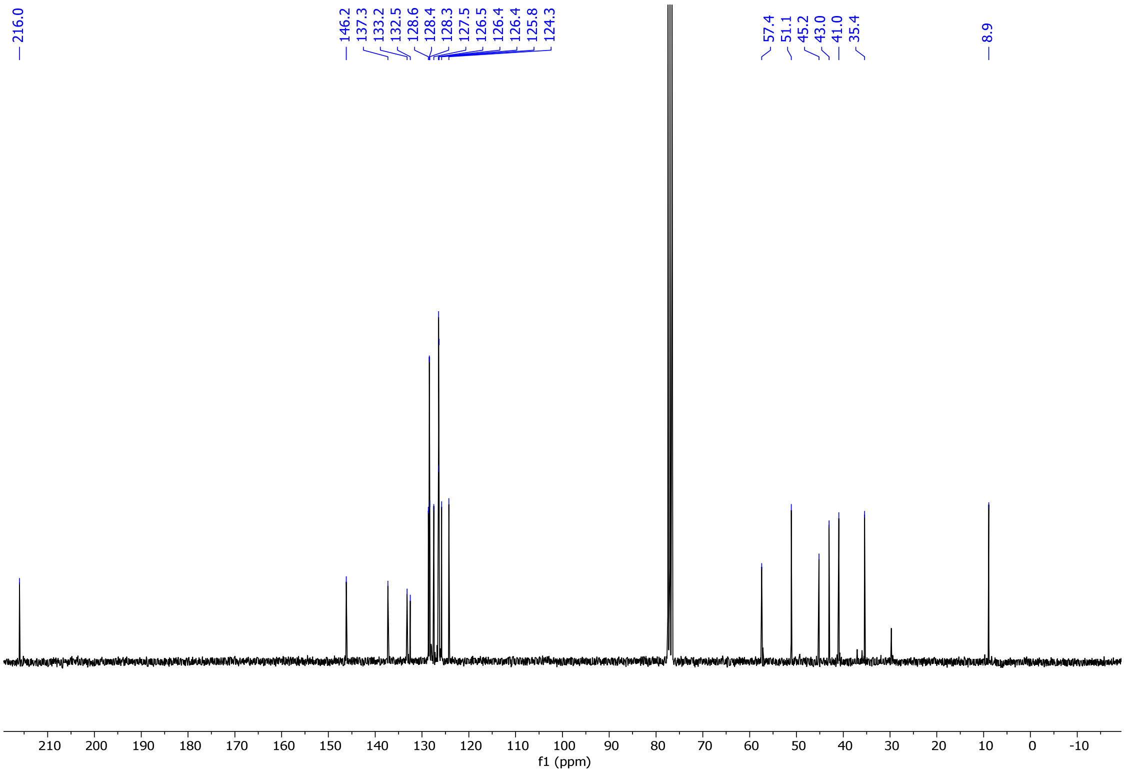

Figure S33. ^{1}H-NMR (300MHz, $CDCl_3$) and $^{13}C\{^{1}H\}$-NMR (75.5MHz, $CDCl_3$) of compound **3f**

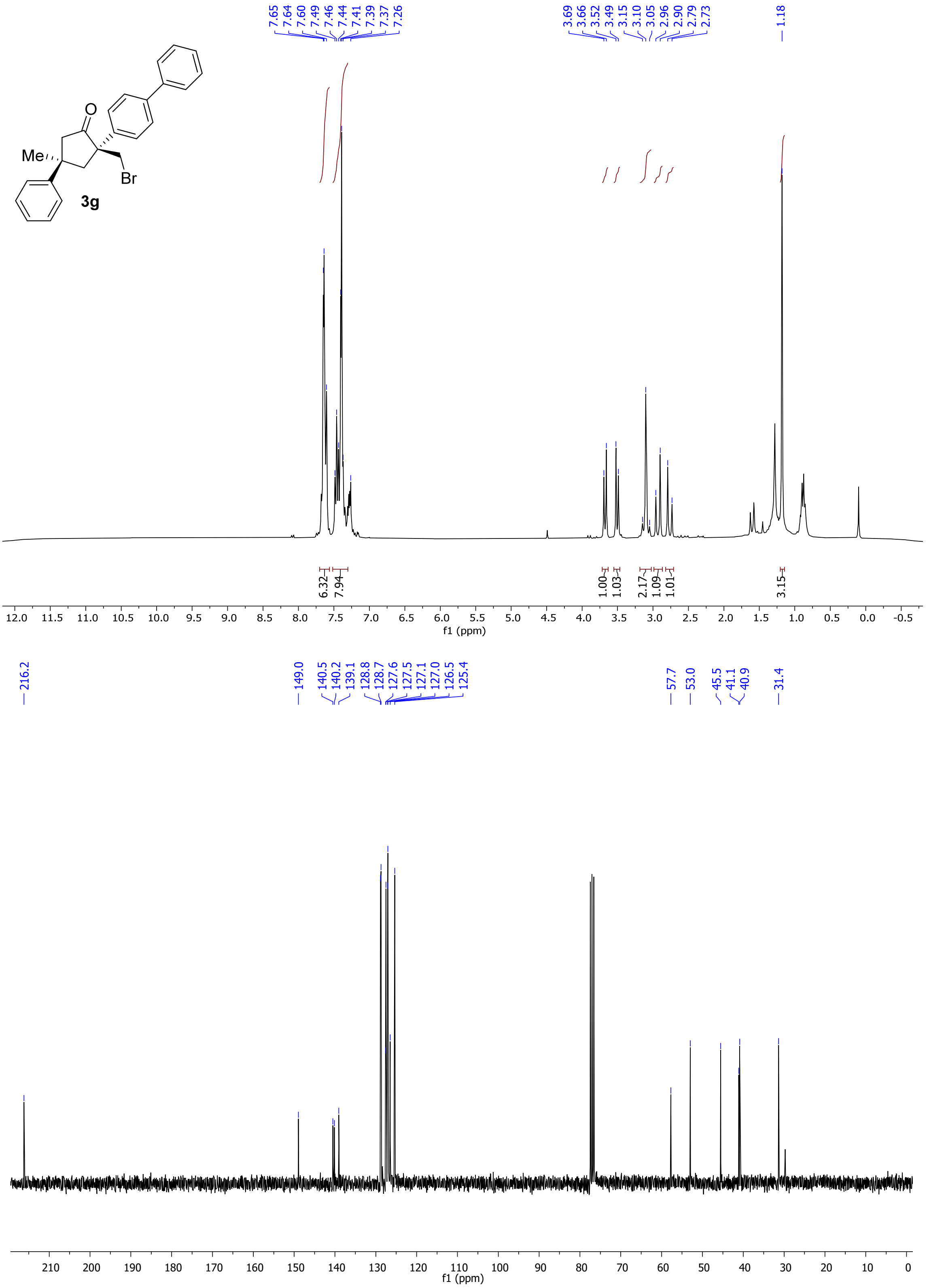

Figure S34. ^{1}H-NMR (300MHz, $CDCl_3$) and $^{13}C\{^{1}H\}$-NMR (75.5MHz, $CDCl_3$) of compound **3g**

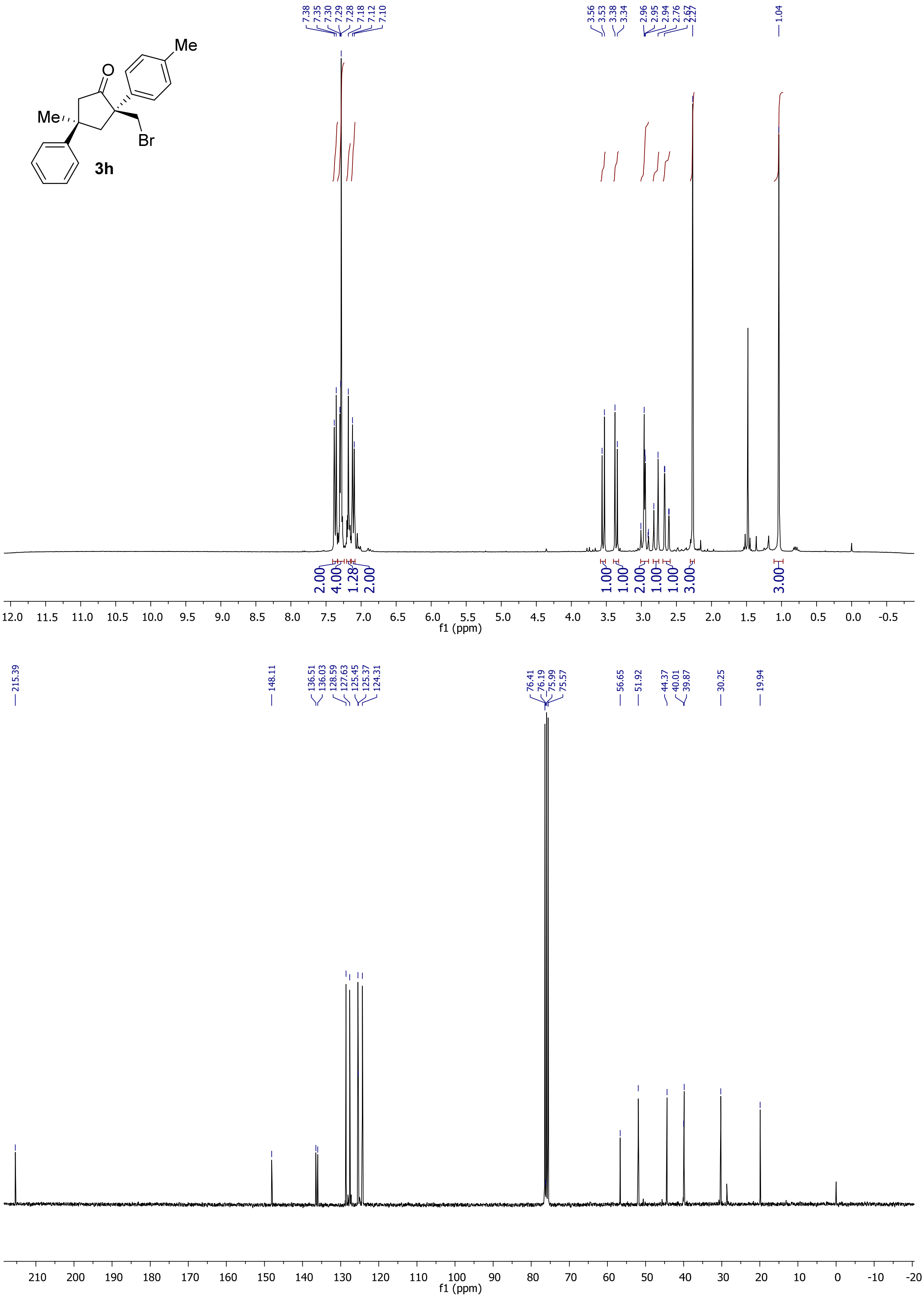

Figure S35. ^{1}H-NMR (300MHz, $CDCl_3$) and $^{13}C\{^{1}H\}$-NMR (75.5MHz, $CDCl_3$) of compound **3h**

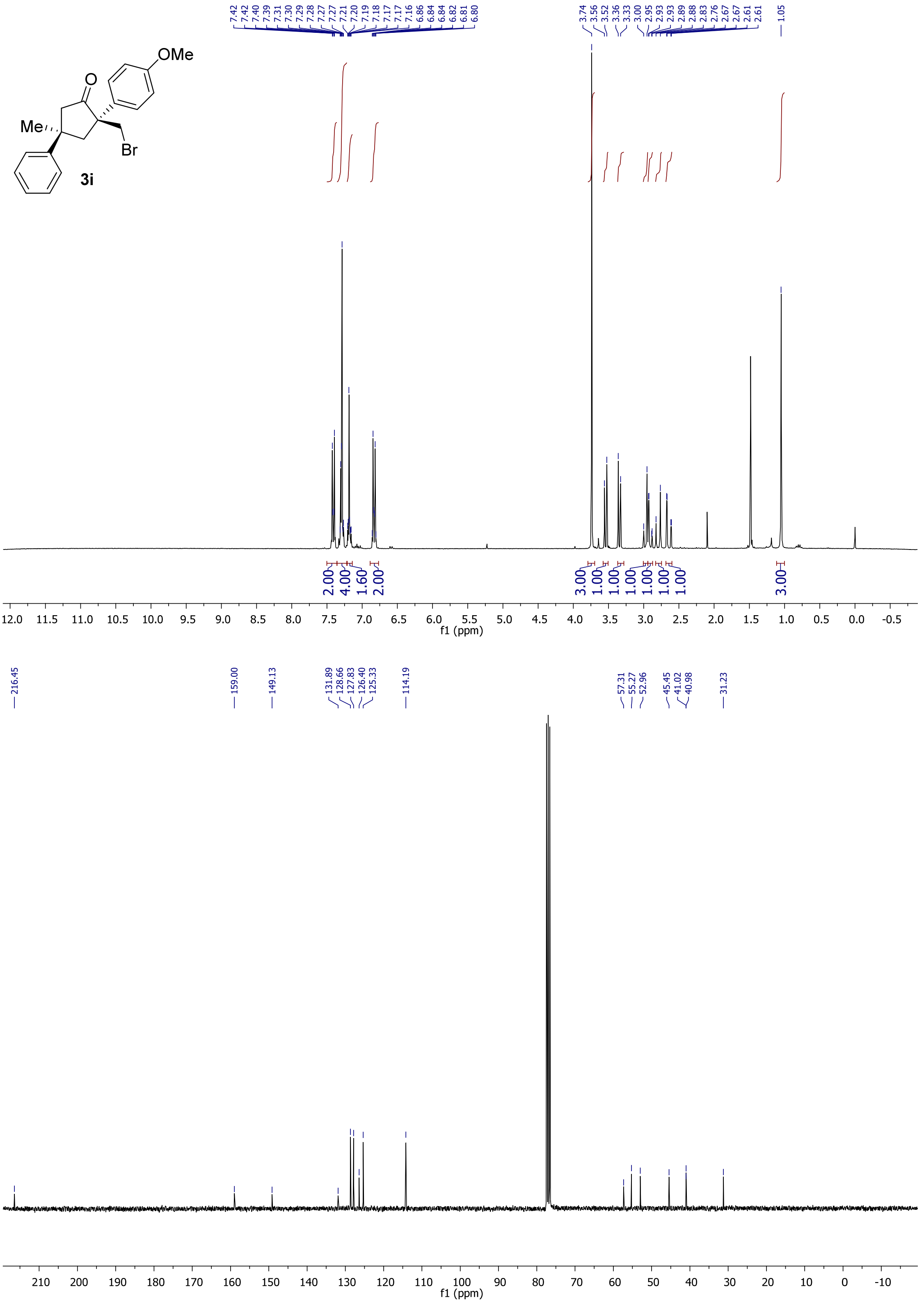

Figure S36. ^{1}H-NMR (300MHz, $CDCl_3$) and $^{13}C\{^{1}H\}$-NMR (75.5MHz, $CDCl_3$) of compound **3i**

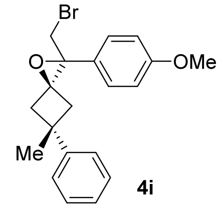

4i

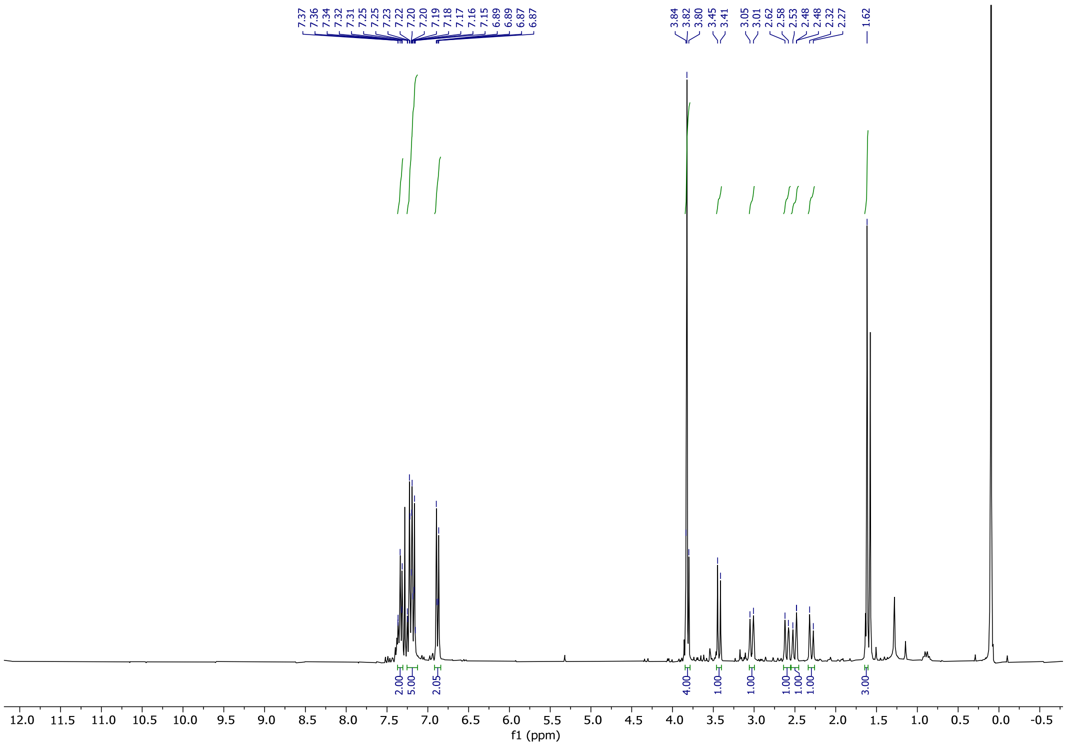

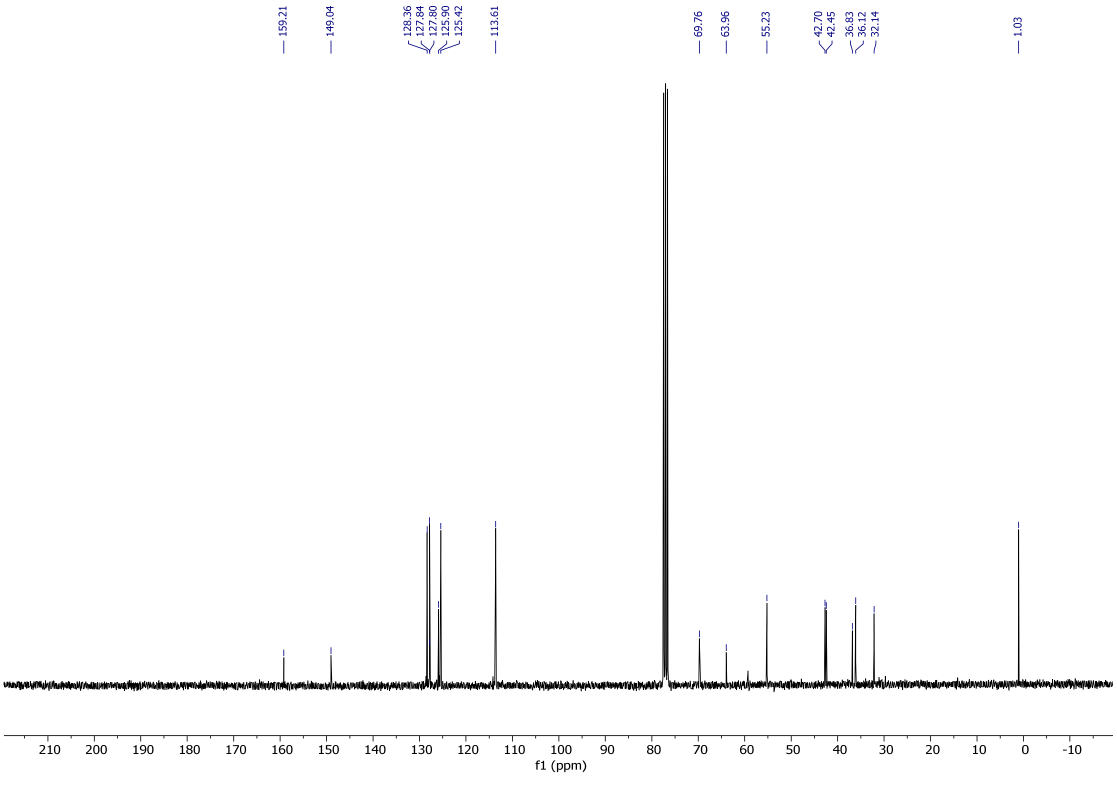

Figure S37. ^{1}H-NMR (300MHz, $CDCl_3$) and $^{13}C\{^{1}H\}$-NMR (75.5MHz, $CDCl_3$) of compound **4i**

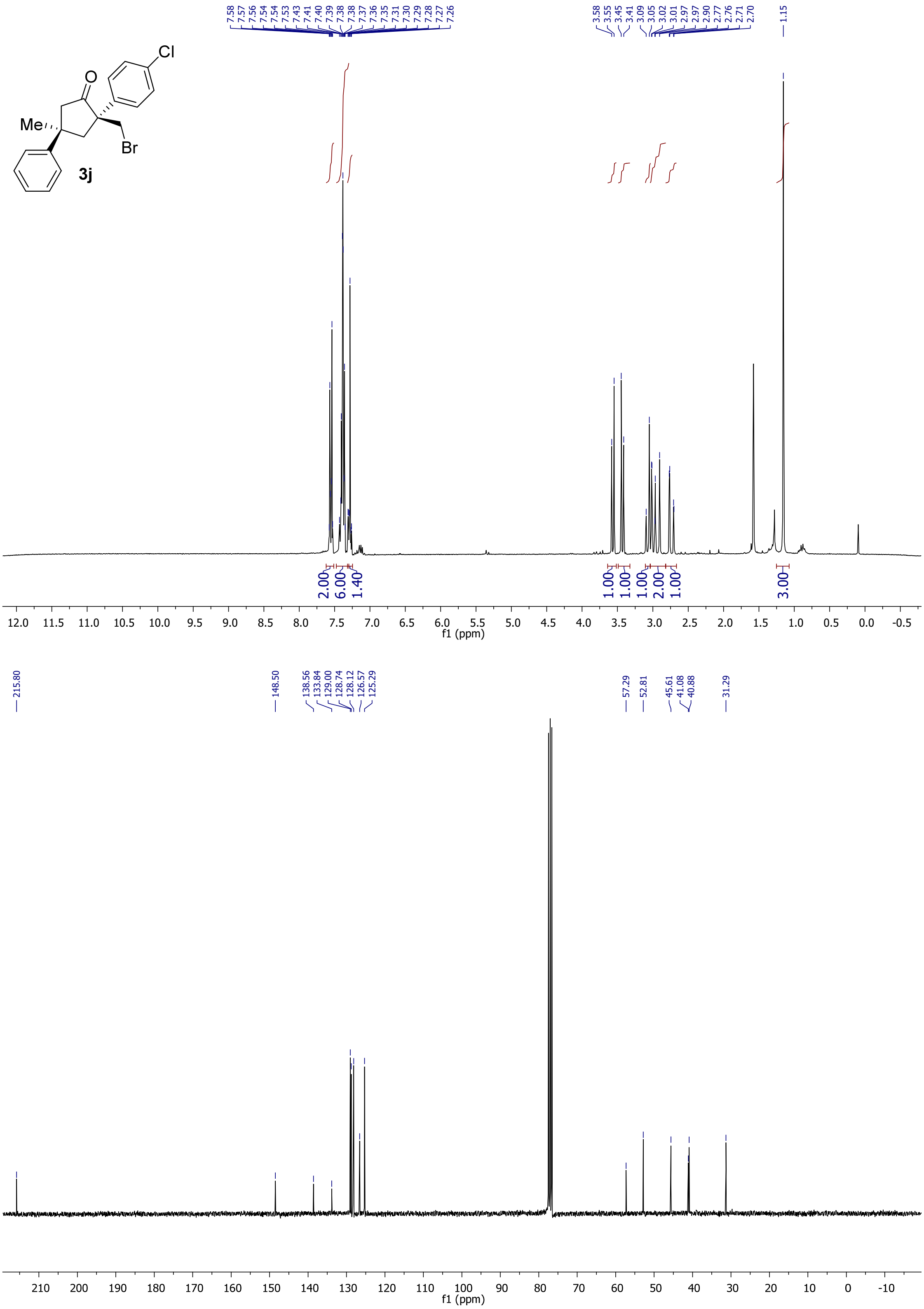

Figure S38. ^{1}H-NMR (300MHz, $CDCl_3$) and $^{13}C\{^{1}H\}$-NMR (75.5MHz, $CDCl_3$) of compound **3j**

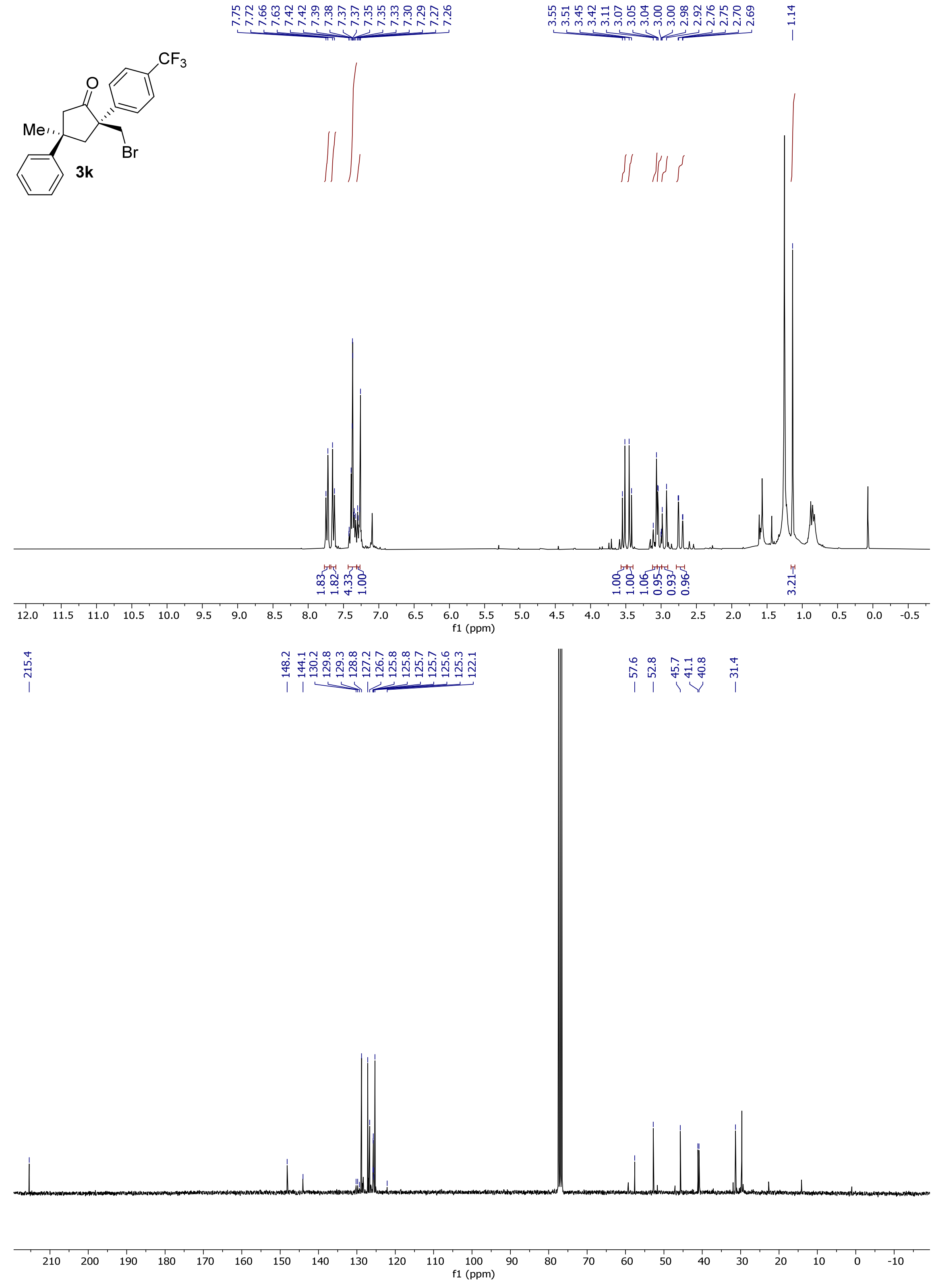

Figure S39. ^{1}H-NMR (300MHz, $CDCl_3$) and $^{13}C\{^{1}H\}$-NMR (75.5MHz, $CDCl_3$) of compound **3k**

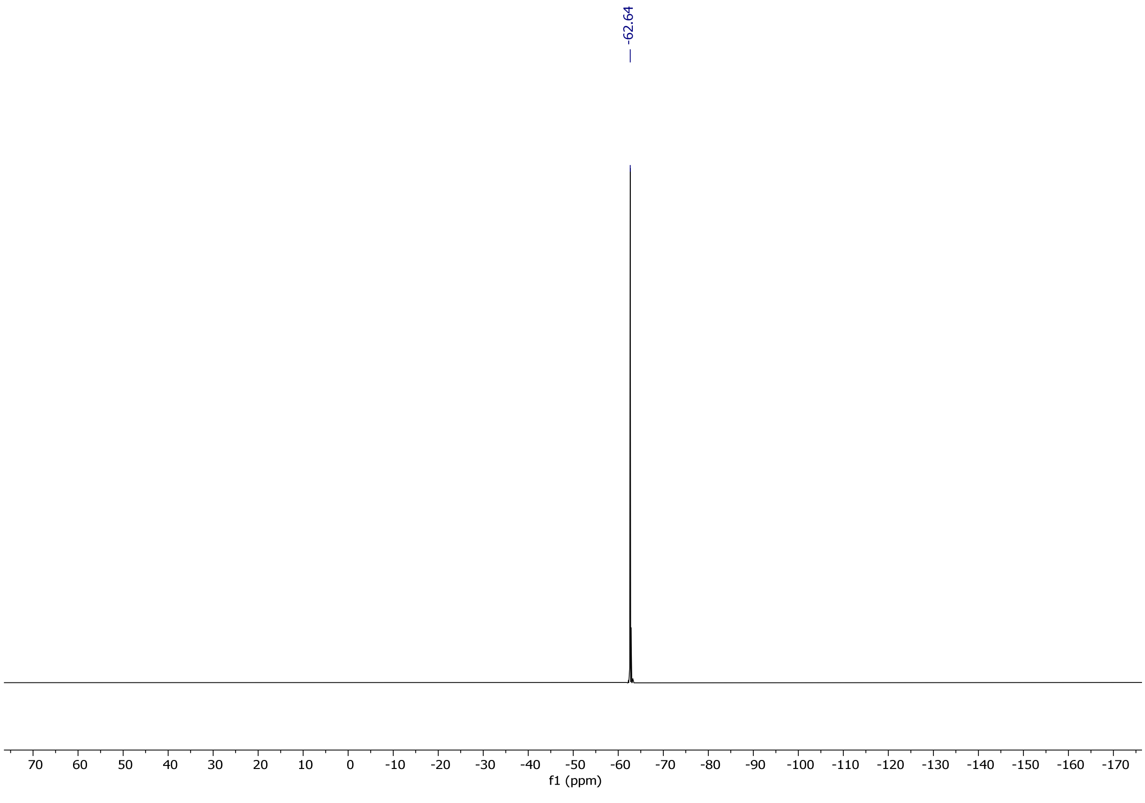

Figure S40. ^{31}P-NMR (122MHz, $CDCl_3$) of compound **3k**

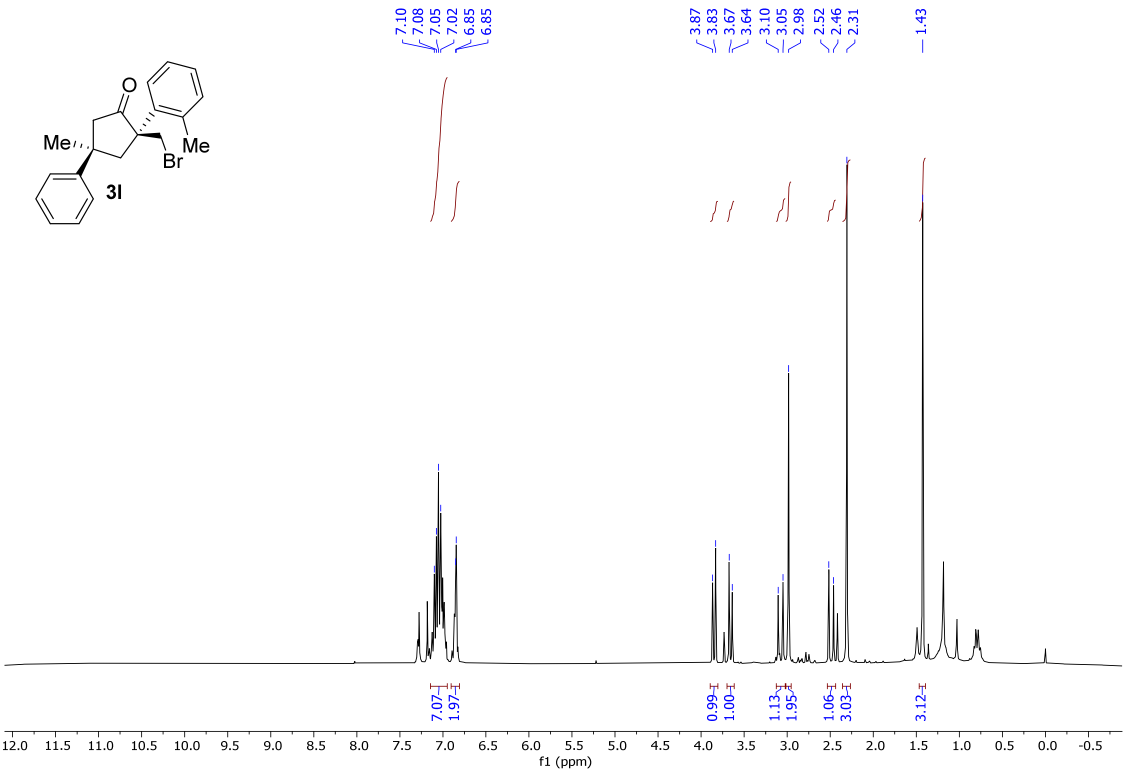

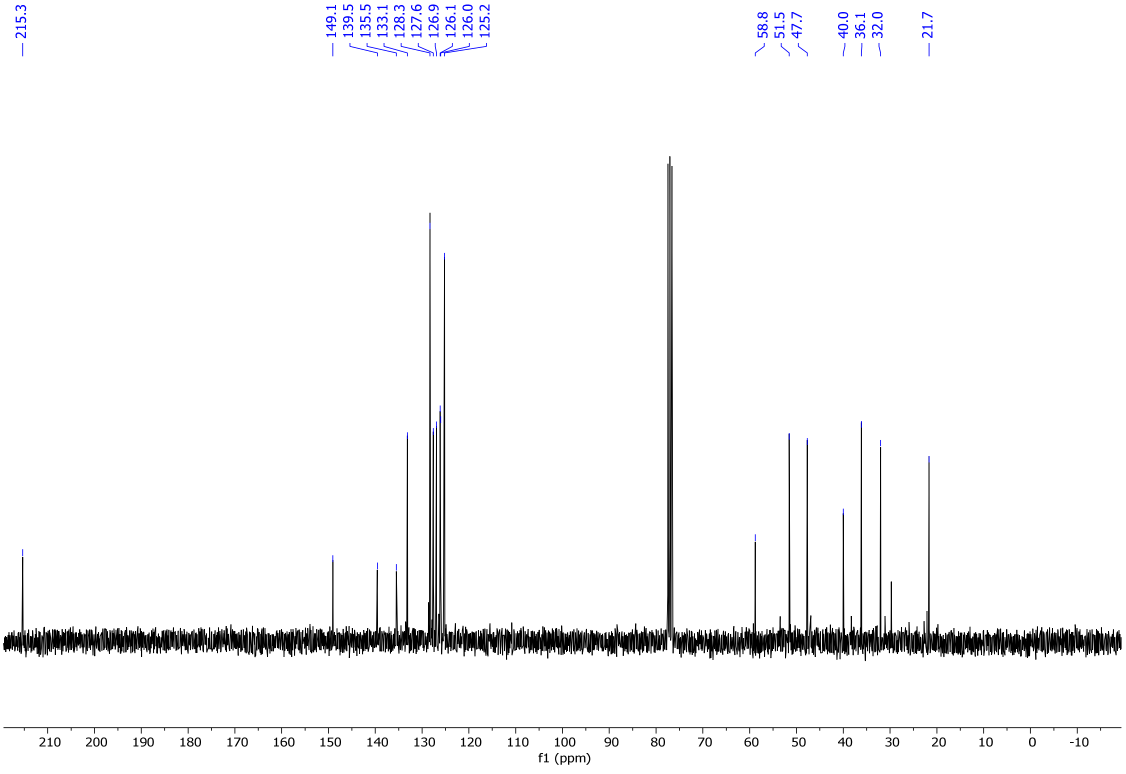

Figure S41. ^{1}H-NMR (300MHz, $CDCl_3$) and $^{13}C\{^{1}H\}$-NMR (75.5MHz, $CDCl_3$) of compound **3l**

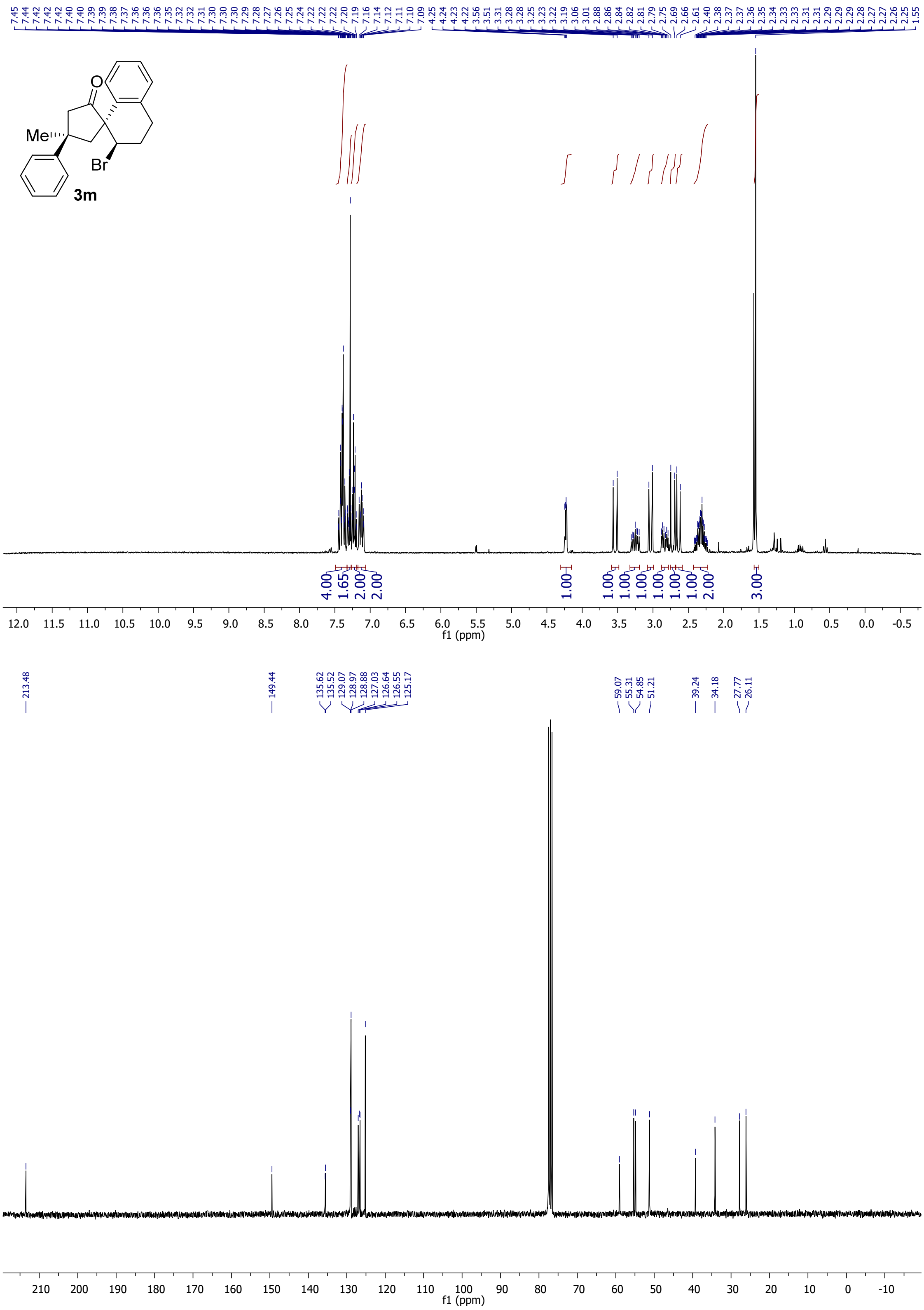

Figure S42. ^{1}H-NMR (300MHz, $CDCl_3$) and $^{13}C\{^{1}H\}$-NMR (75.5MHz, $CDCl_3$) of compound **3m**

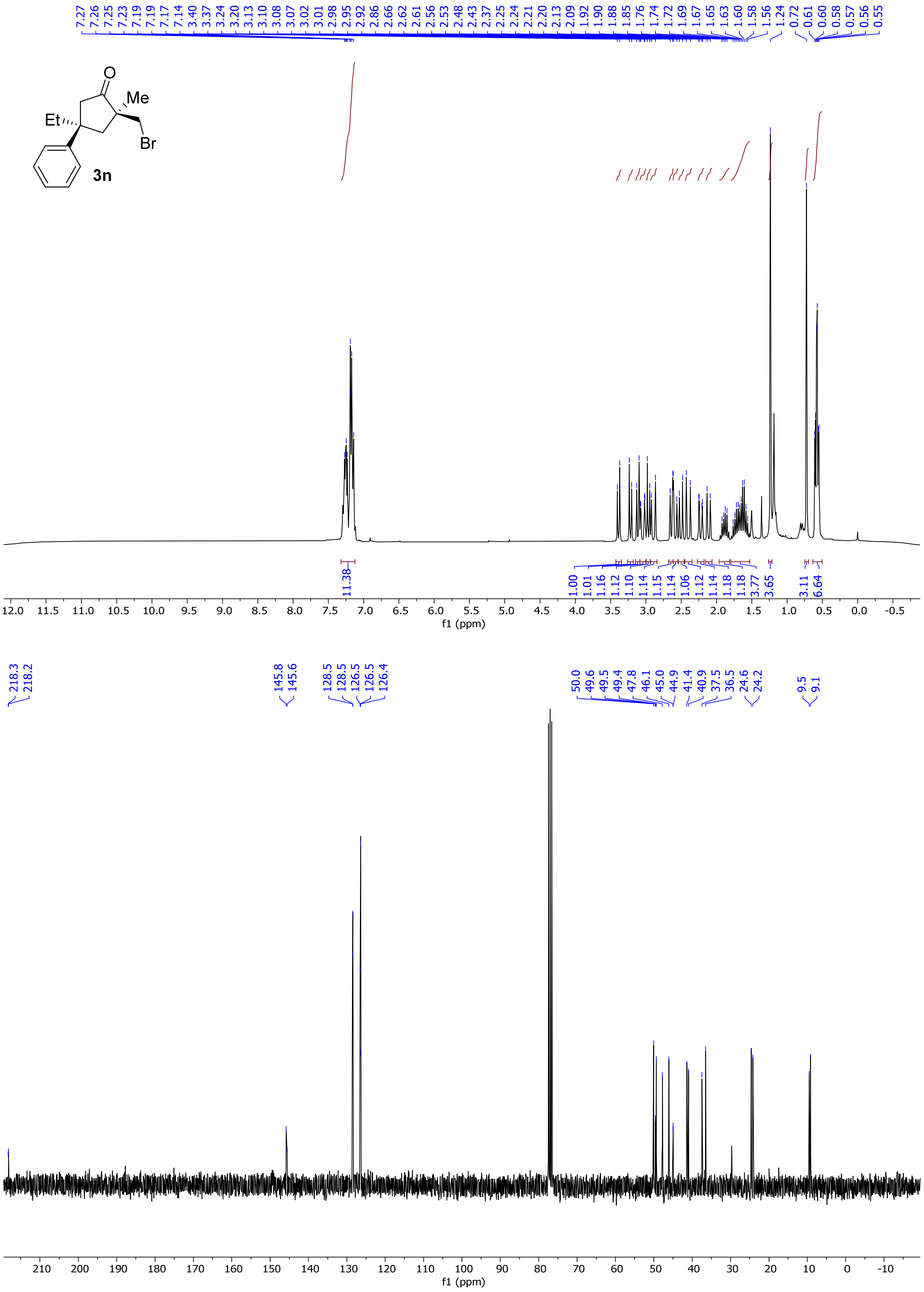

Figure S43. ^{1}H-NMR (300MHz, $CDCl_3$) and $^{13}C\{^{1}H\}$-NMR (75.5MHz, $CDCl_3$) of compound **3n**

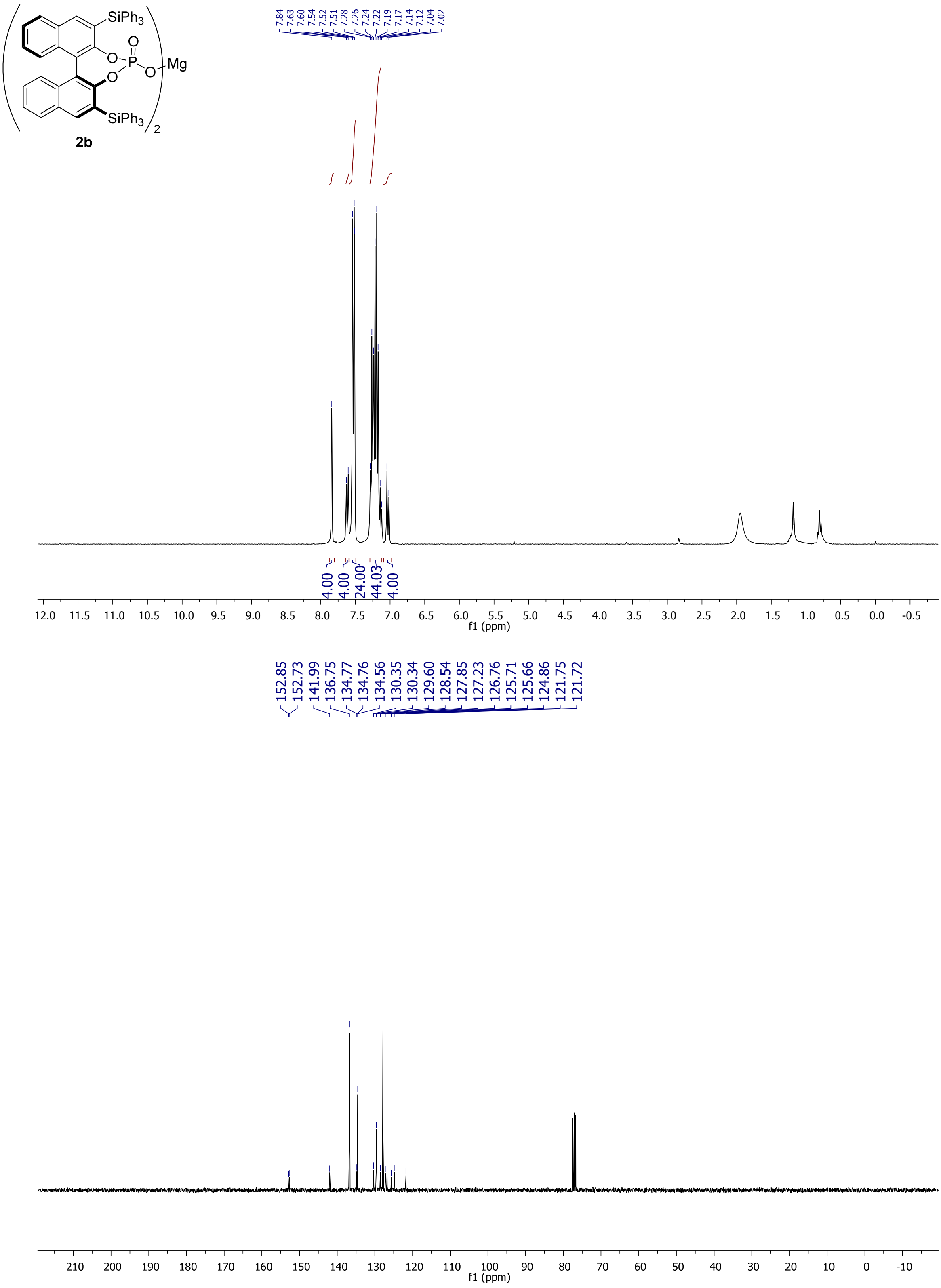

Figure S44. ^{1}H-NMR (300MHz, $CDCl_3$) and $^{13}C\{^{1}H\}$-NMR (75.5MHz, $CDCl_3$) of compound **2b**

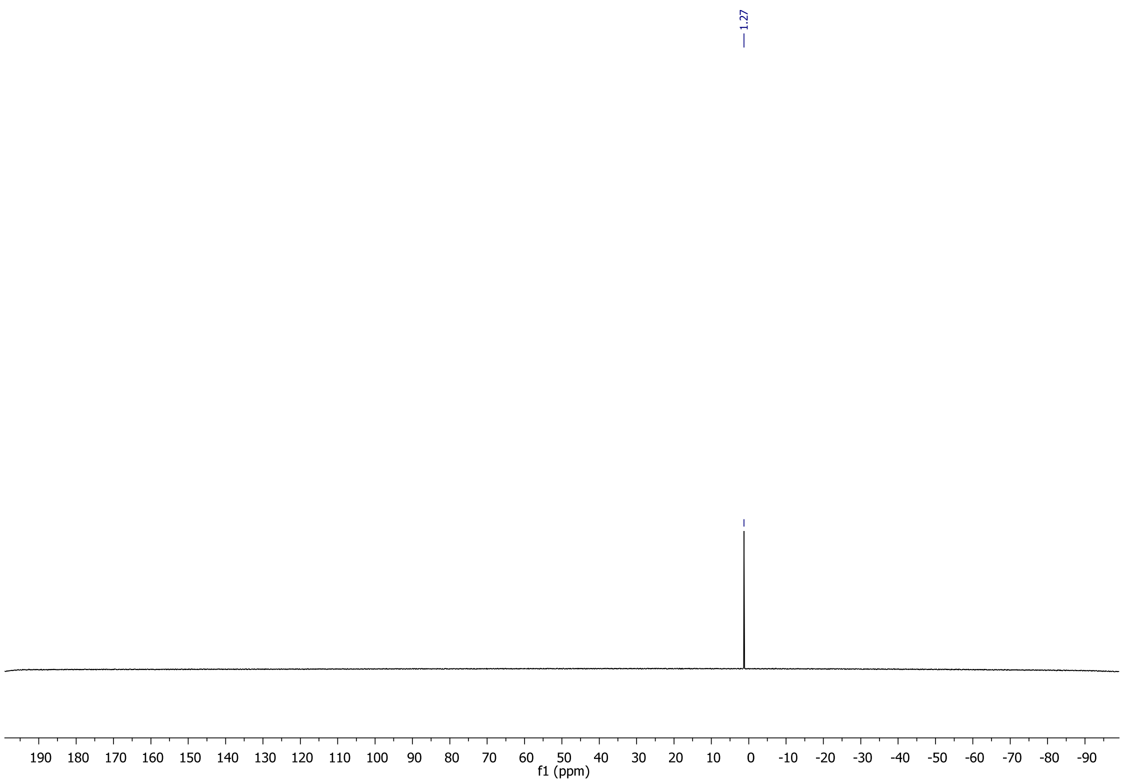

Figure S45. ^{31}P-NMR (122MHz, $CDCl_3$) of compound **2b**

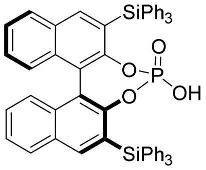

2a

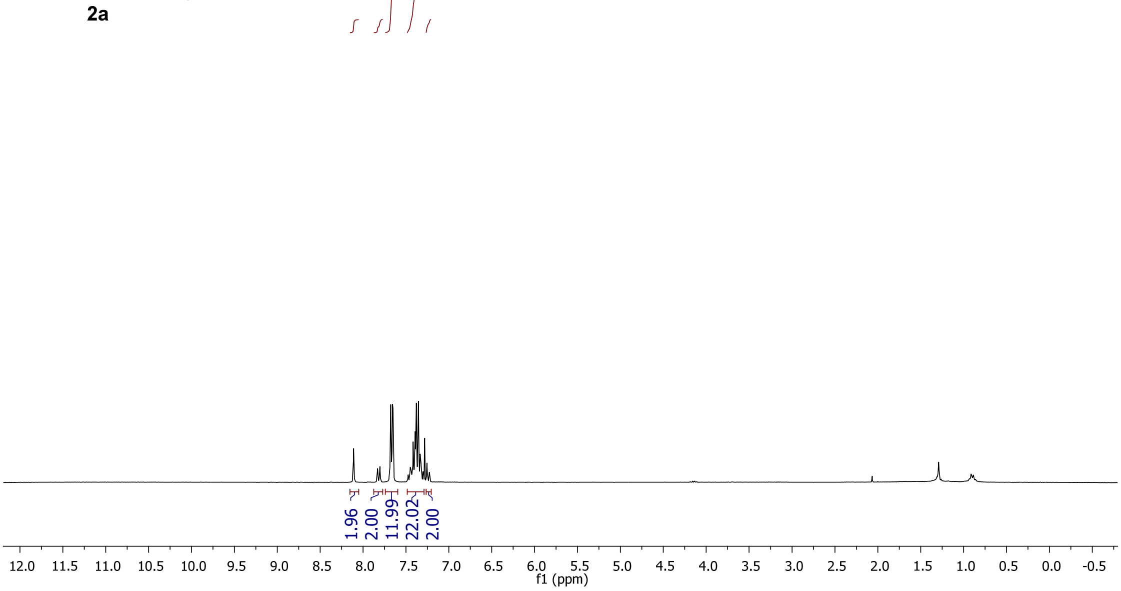

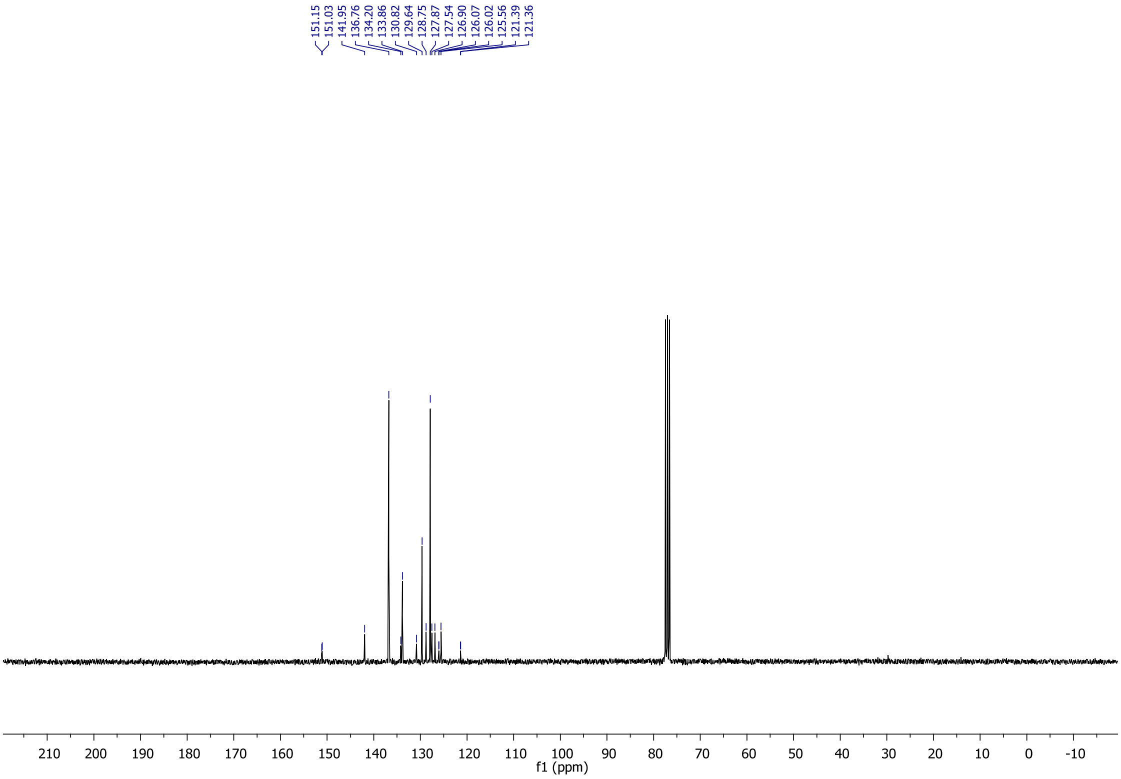

Figure S46. ^{1}H-NMR (300MHz, $CDCl_3$) and $^{13}C\{^{1}H\}$-NMR (75.5MHz, $CDCl_3$) of compound **2a**

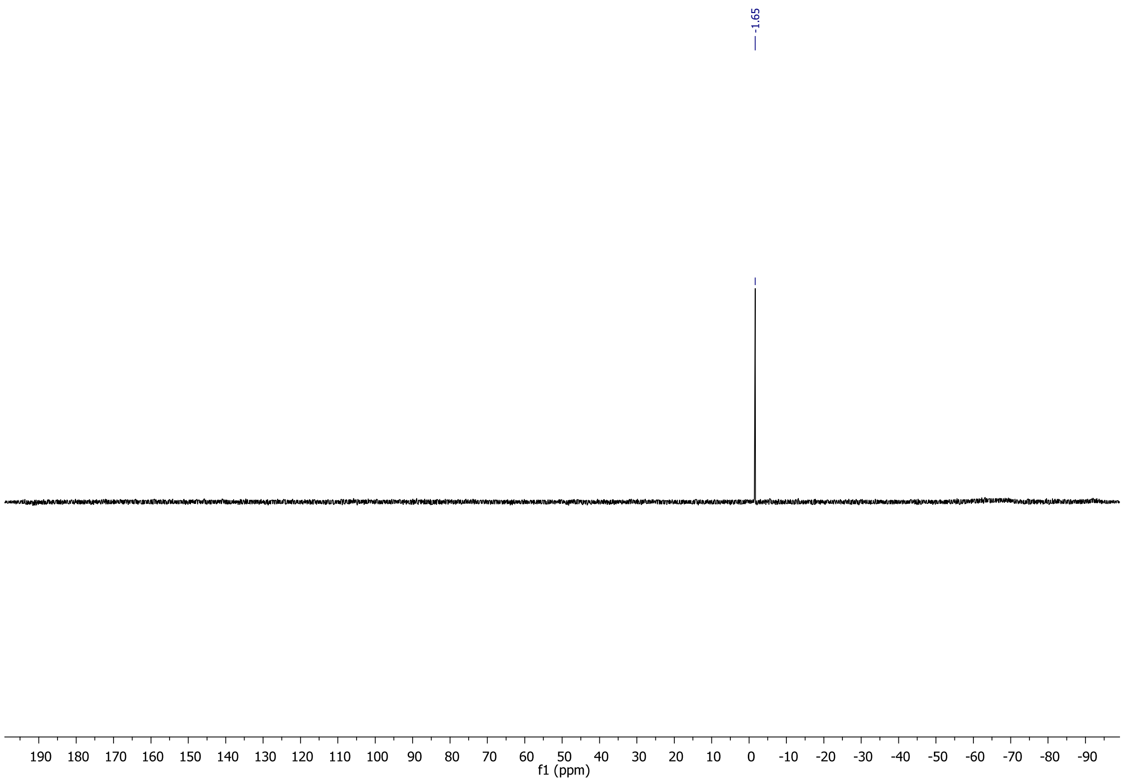

Figure S47. ^{31}P-NMR (122MHz, $CDCl_3$) of compound **2a**

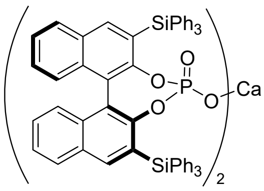

2k

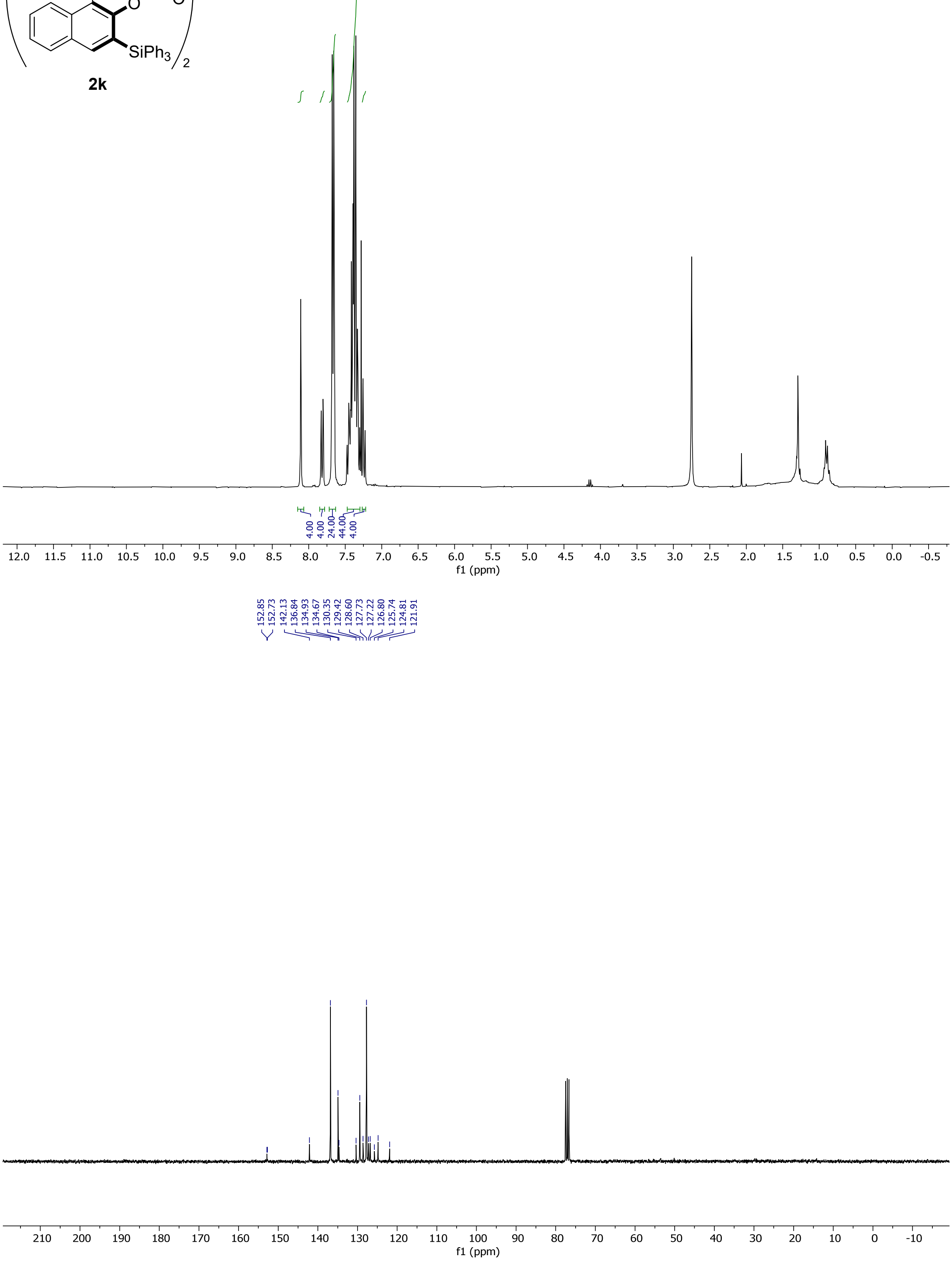

Figure S48. ^{1}H-NMR (300MHz, $CDCl_3$) and $^{13}C\{^{1}H\}$-NMR (75.5MHz, $CDCl_3$) of compound **2k**

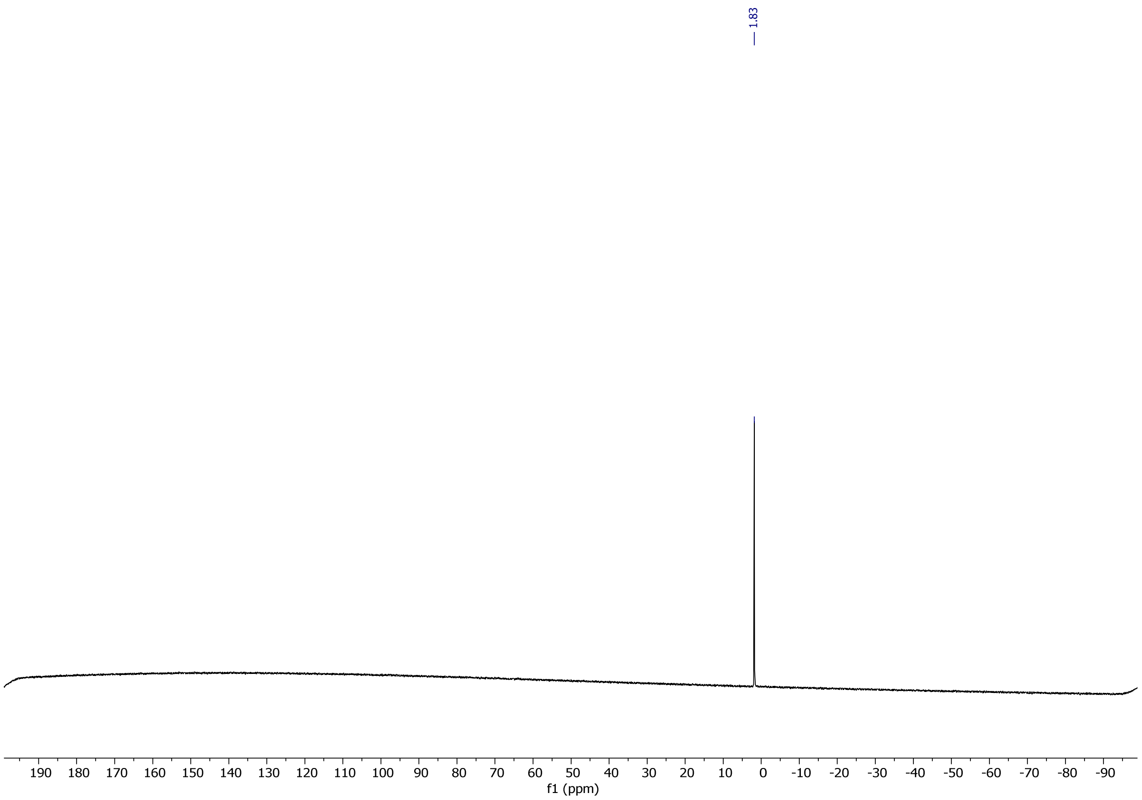

Figure S49. ^{31}P-NMR (122MHz, $CDCl_3$) of compound **2k**

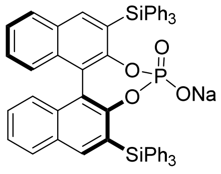

2l

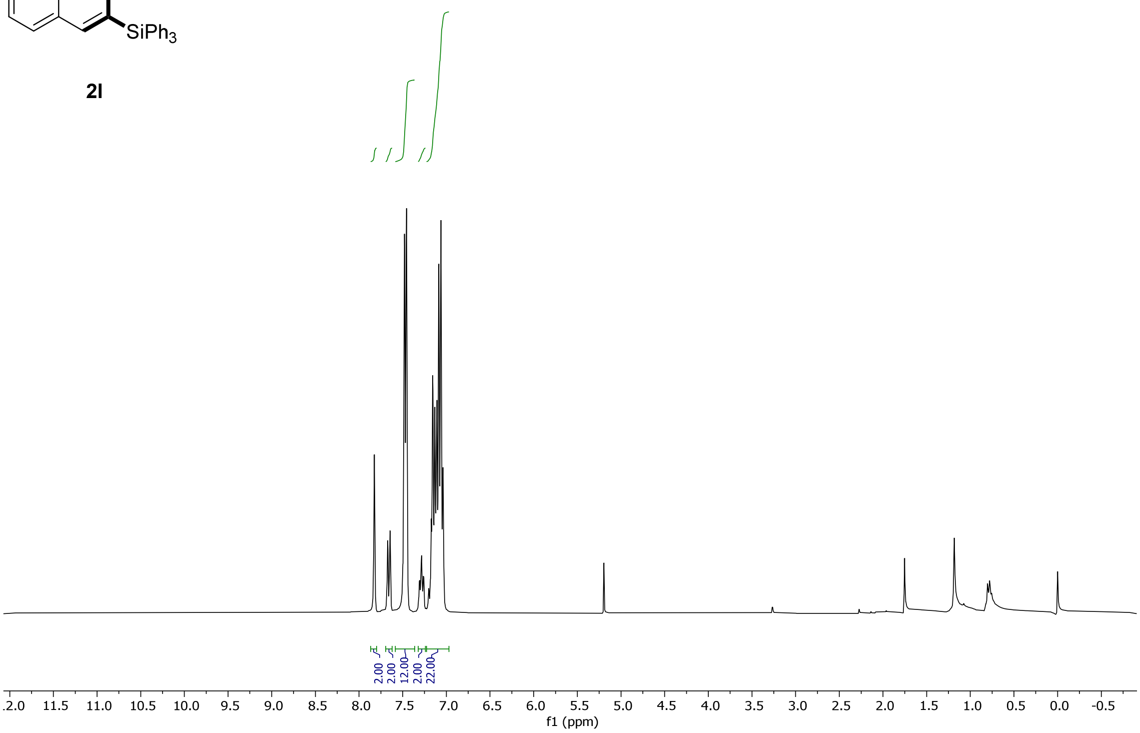

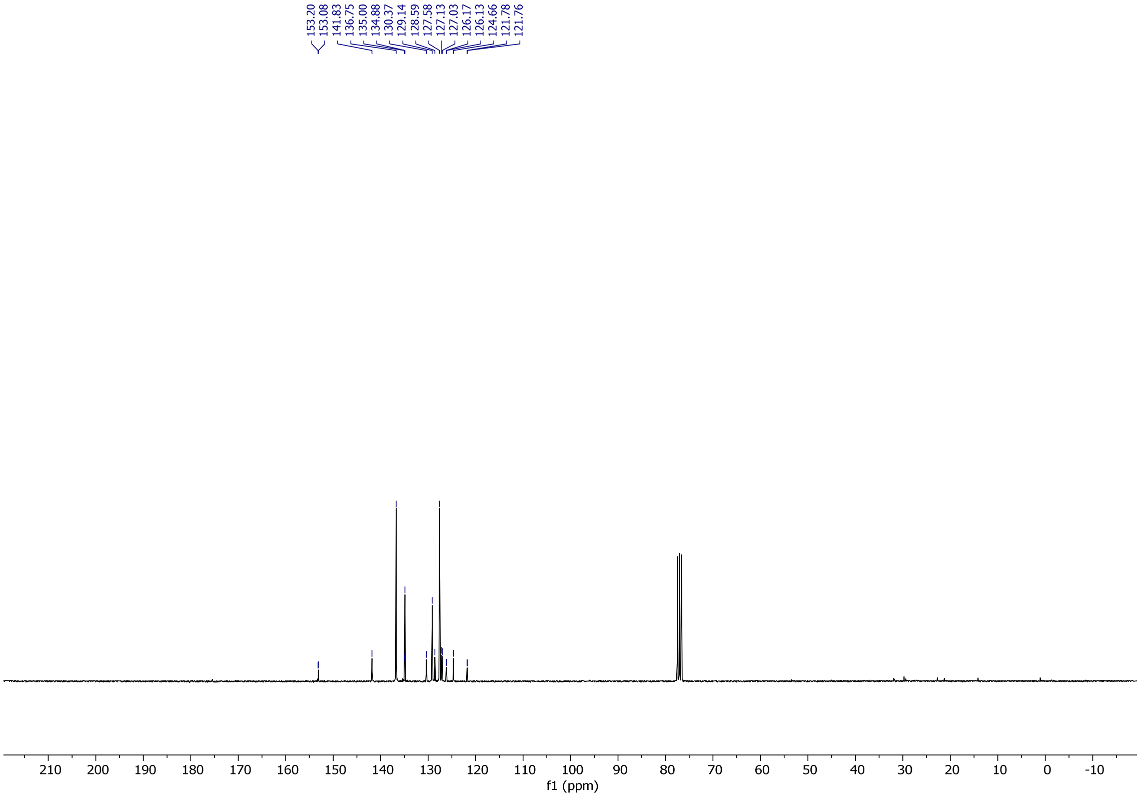

Figure S50. ^{1}H-NMR (300MHz, $CDCl_3$) and $^{13}C\{^{1}H\}$-NMR (75.5MHz, $CDCl_3$) of compound **2l**

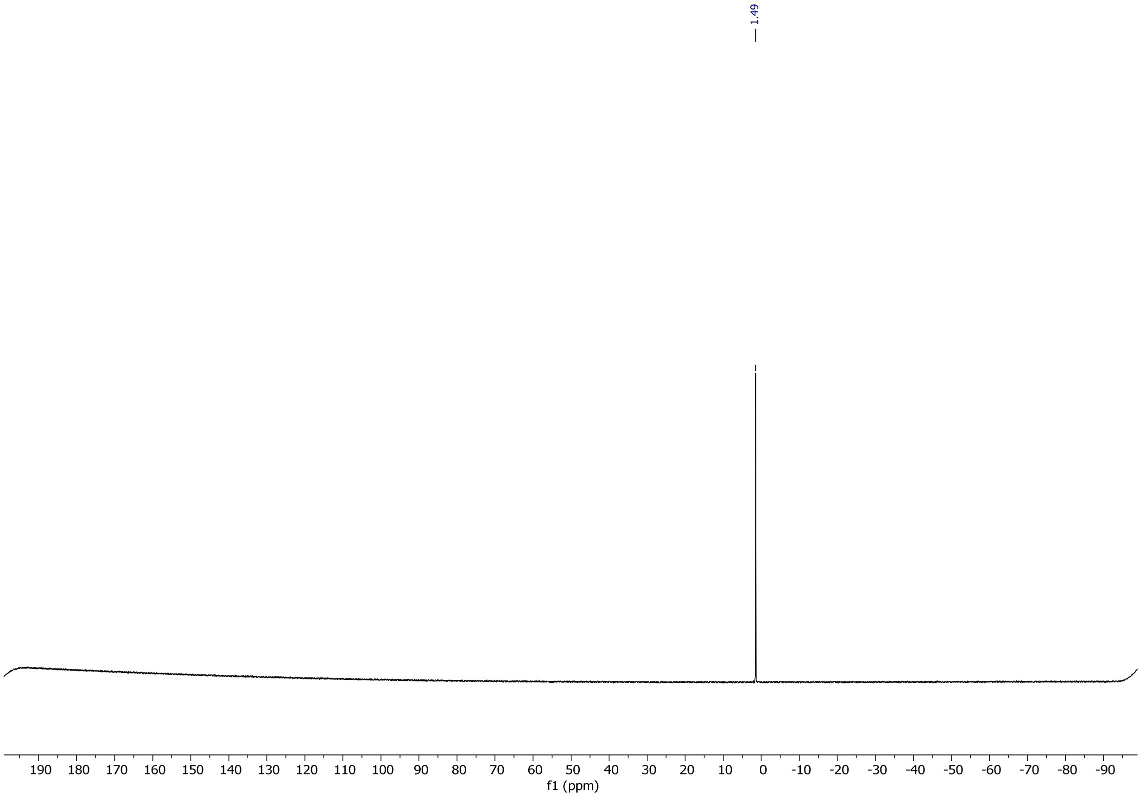

Figure S51. ^{31}P-NMR (122MHz, $CDCl_3$) of compound **2l**

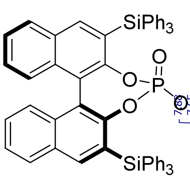

2m

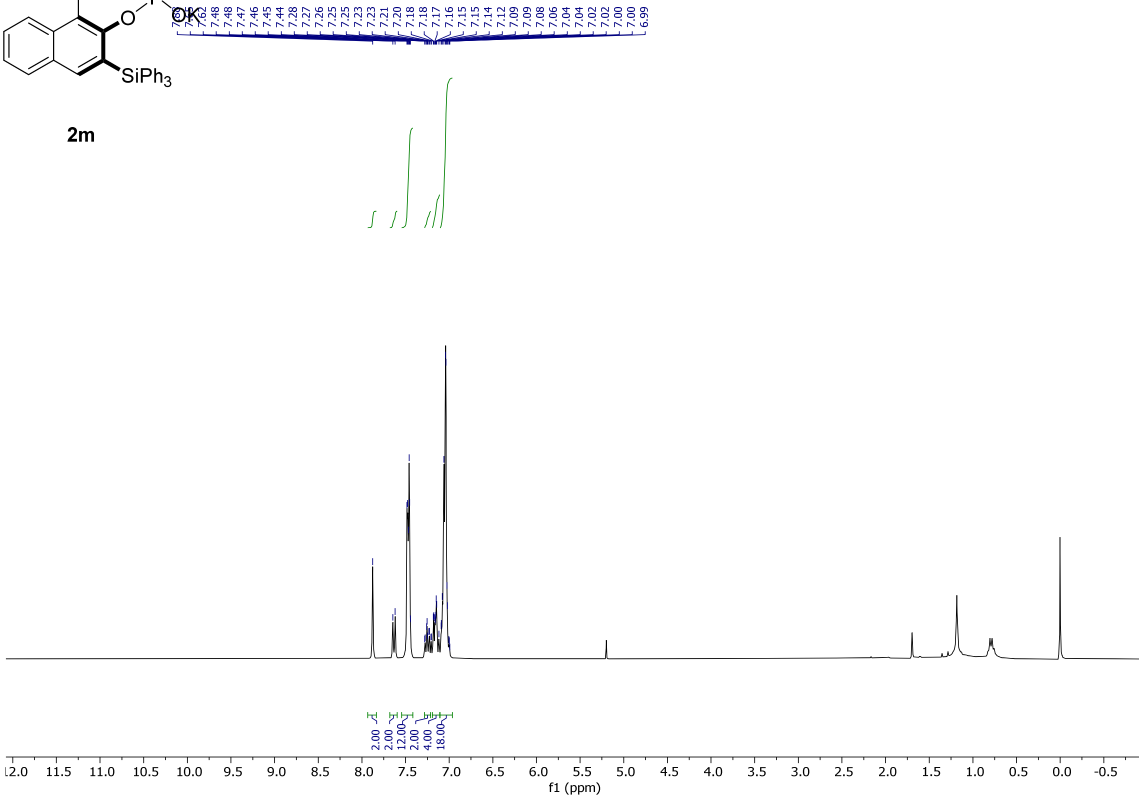

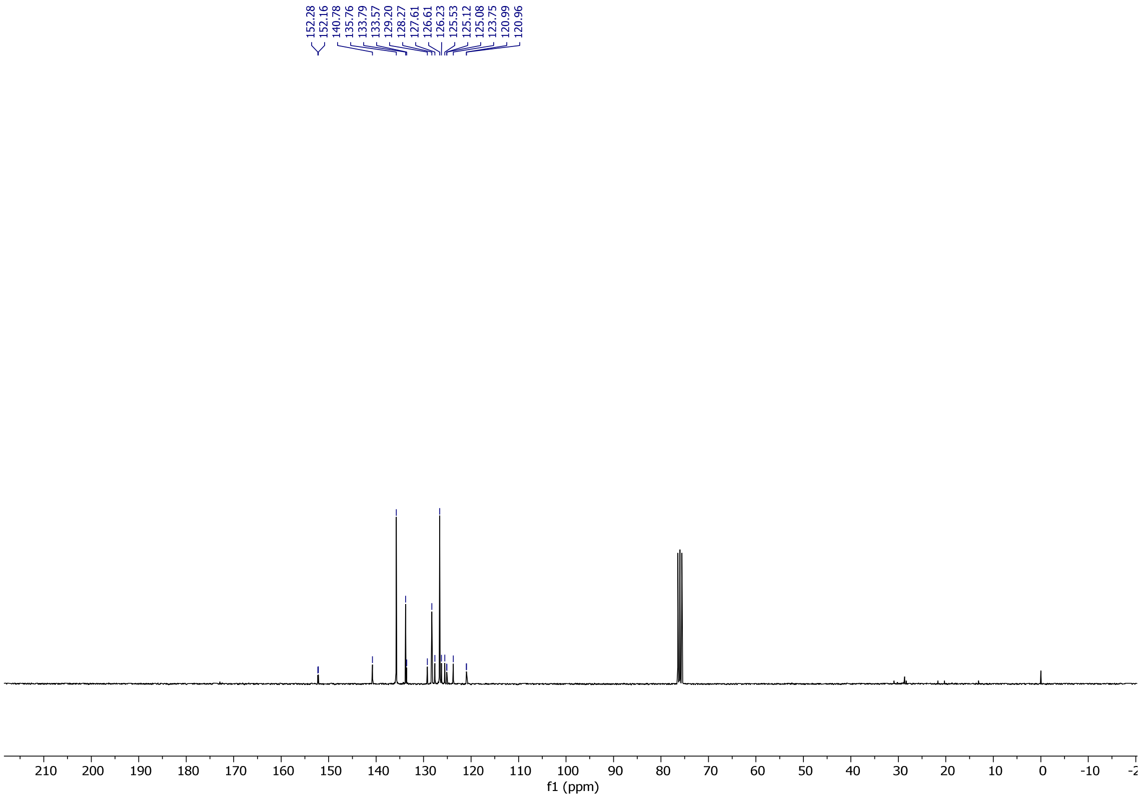

Figure S52. ^{1}H-NMR (300MHz, $CDCl_3$) and $^{13}C\{^{1}H\}$-NMR (75.5MHz, $CDCl_3$) of compound **2m**

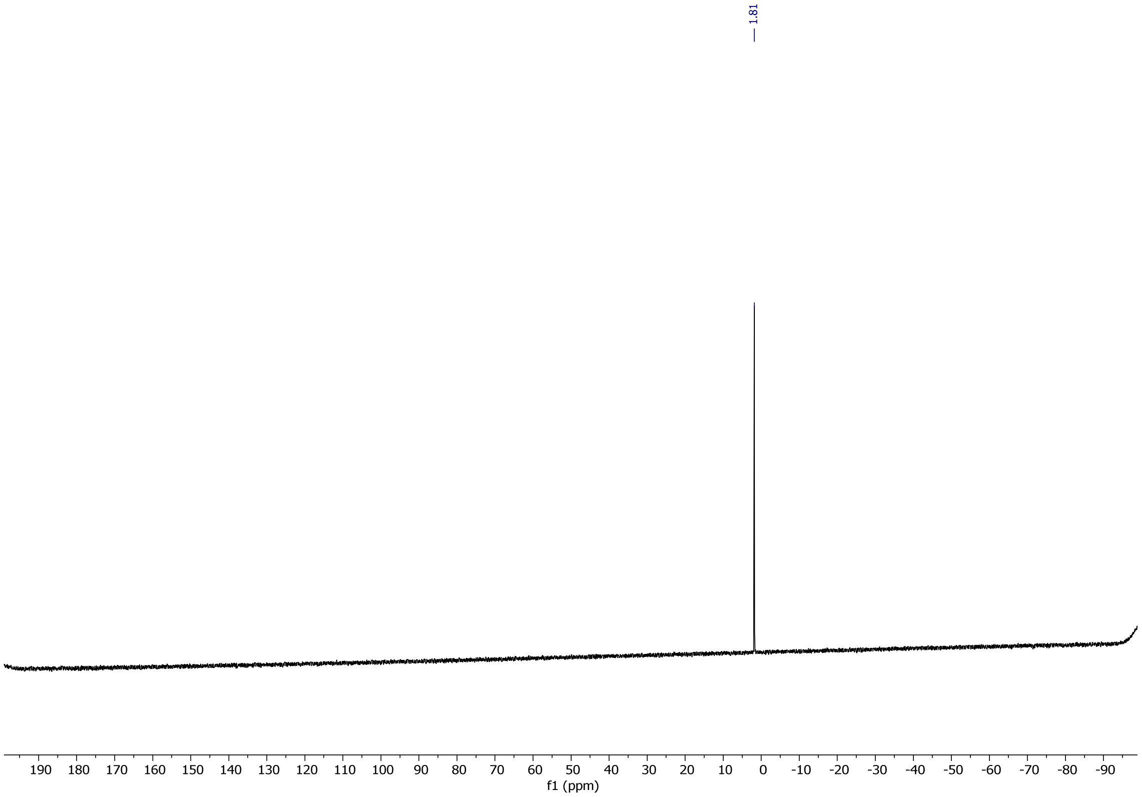

Figure S53. ^{31}P-NMR (300MHz, $CDCl_3$) of compound **2m**

^{31}P NMR (122 MHz, $CDCl_3$) spectra of catalyst mixtures for non linear effect study

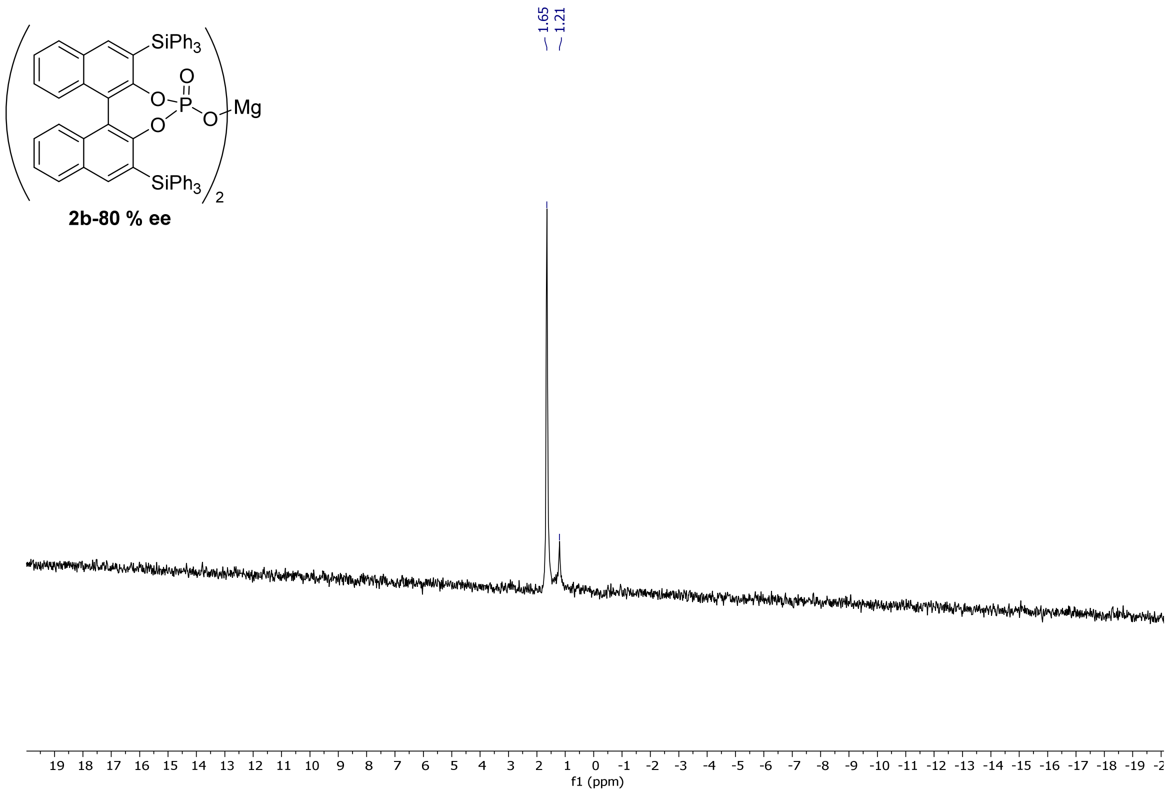

2b-80 % ee

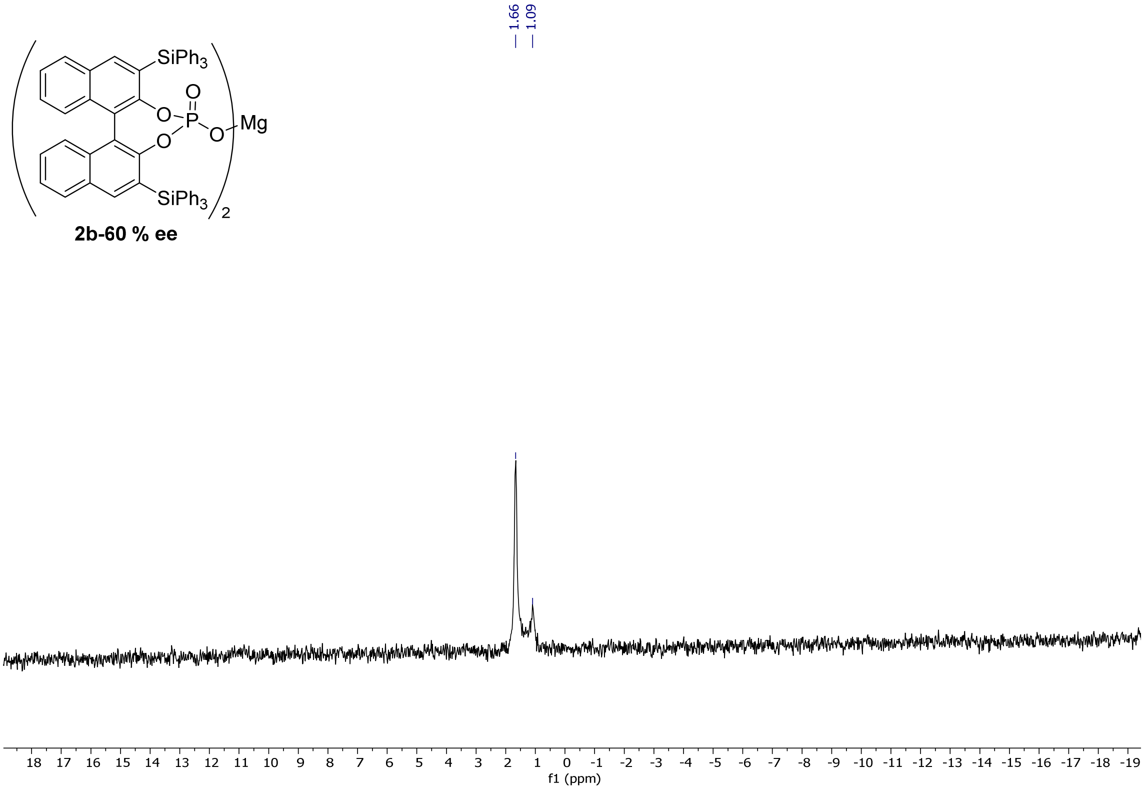

2b-60 % ee

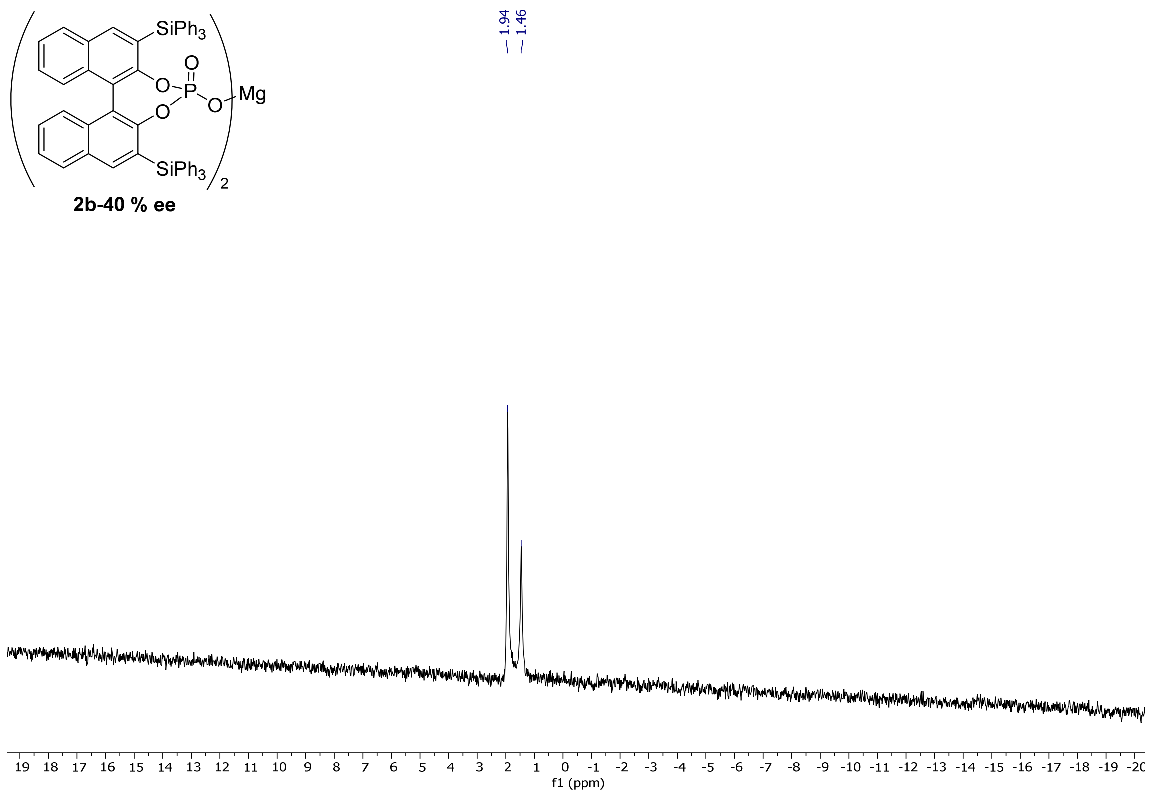

2b-40 % ee

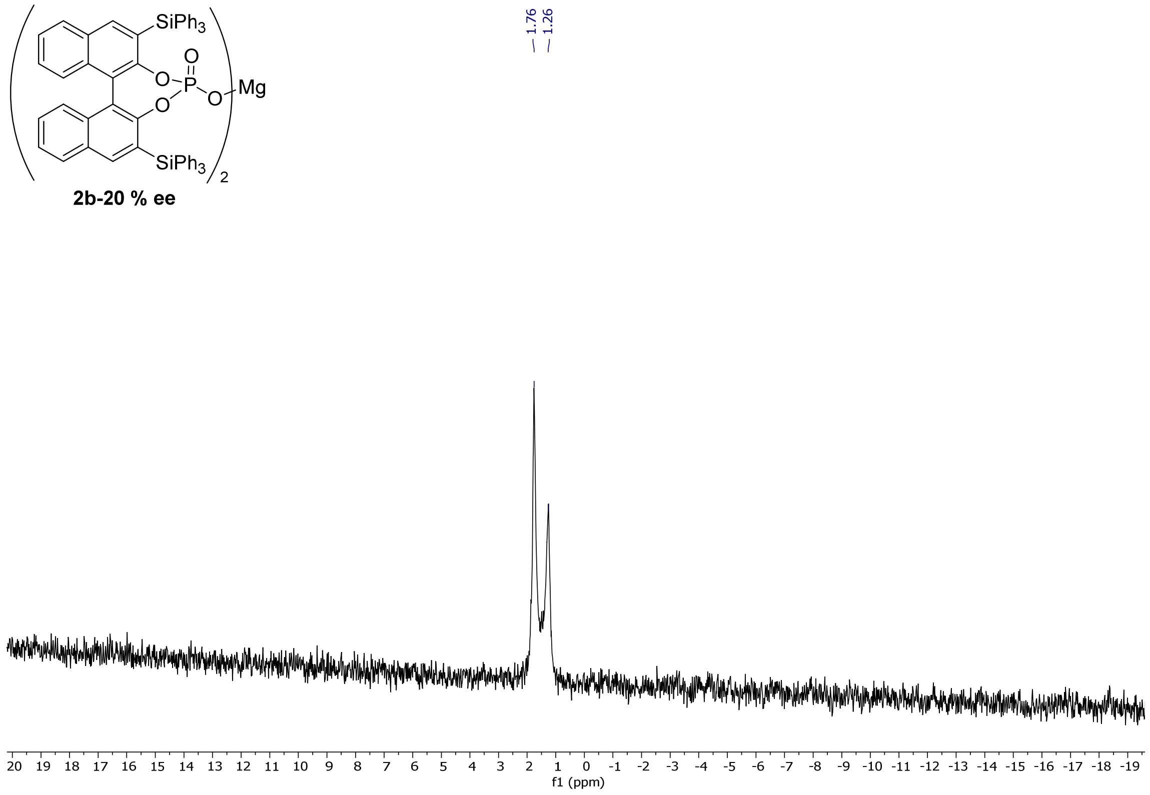

2b-20 % ee

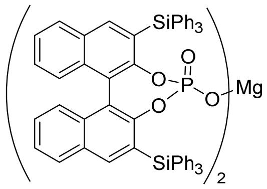
SiPh3
O
O
P
O
O
Mg
SiPh3
2

2b-0 % ee

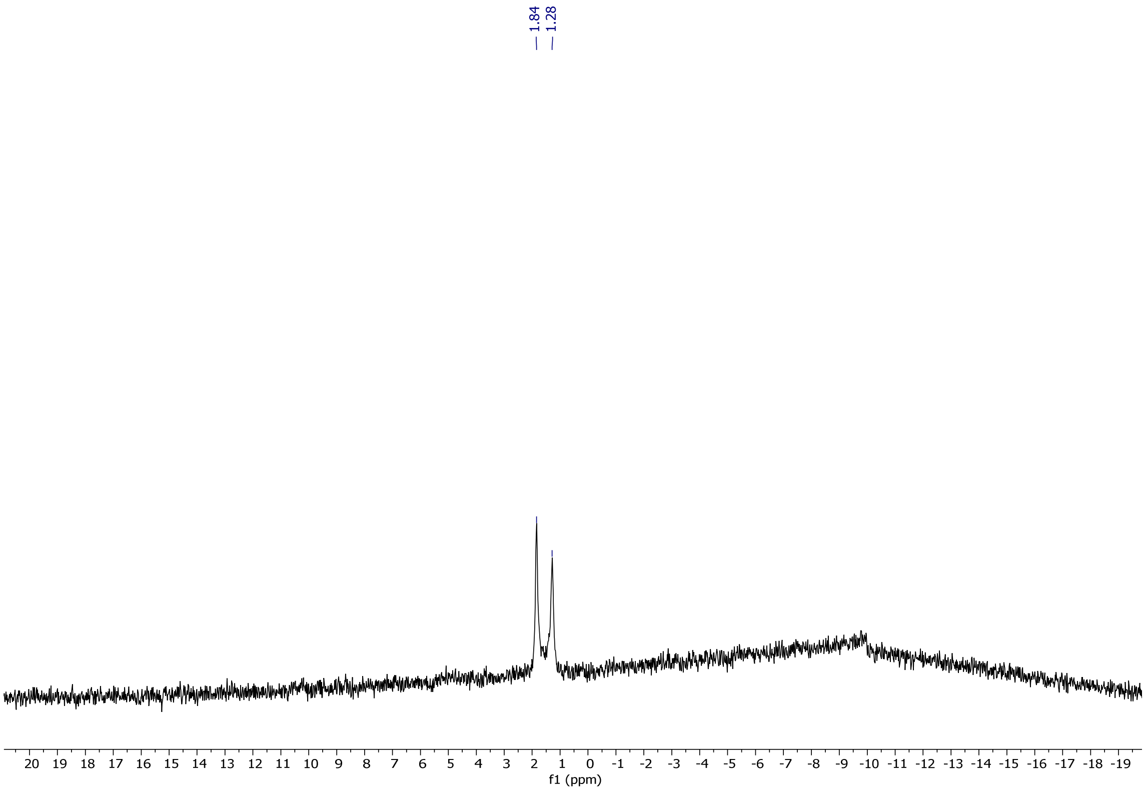
1.84
1.28
20 19 18 17 16 15 14 13 12 11 10 9 8 7 6 5 4 3 2 1 0 -1 -2 -3 -4 -5 -6 -7 -8 -9 -10 -11 -12 -13 -14 -15 -16 -17 -18 -19
f1 (ppm)

HPLC analysis

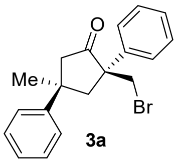

3a

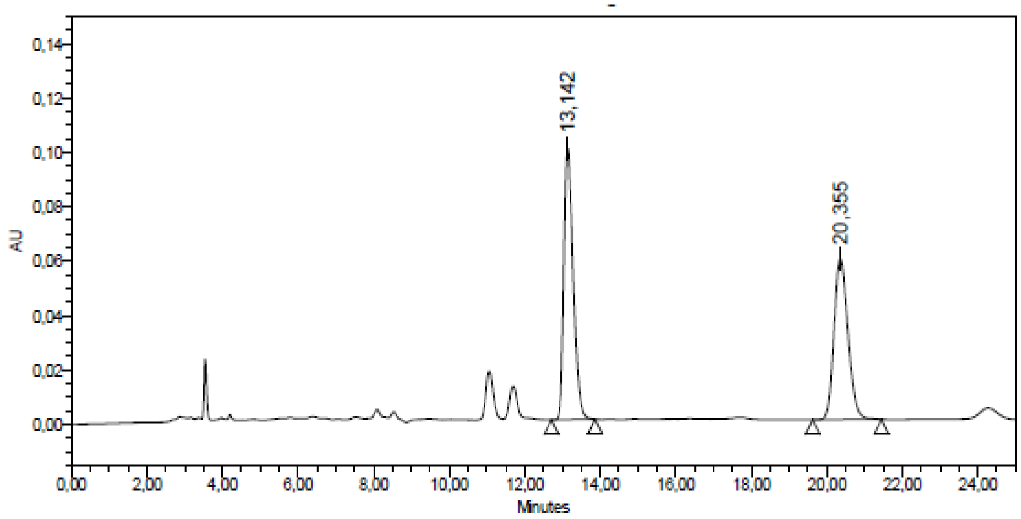

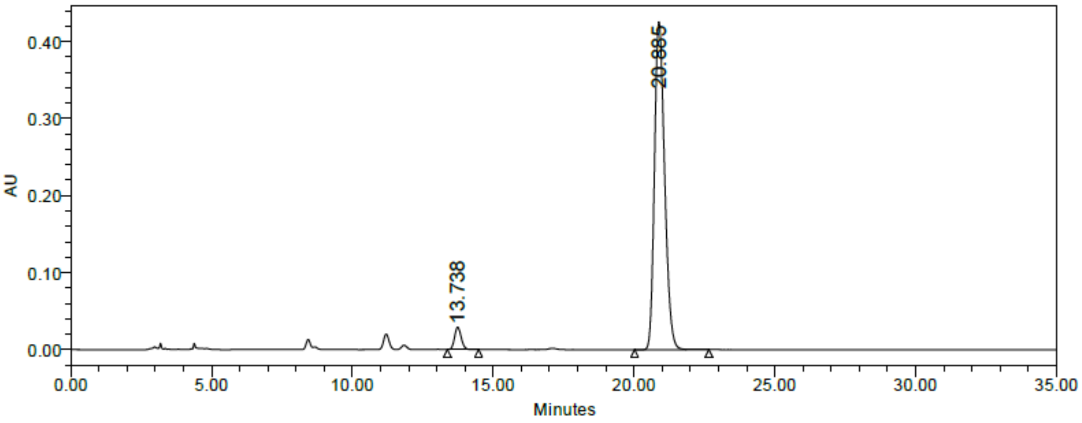

	RT	Area	% Area	Height
1	13.738	473930	4.26	29000
2	20.885	10659848	95.74	425505

Match Plot

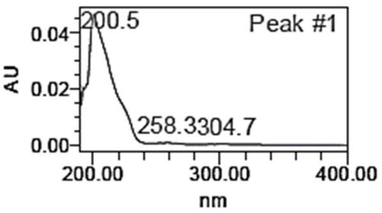

Match Plot

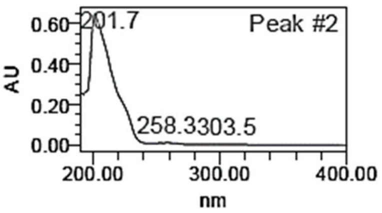

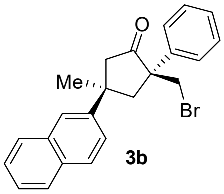

3b

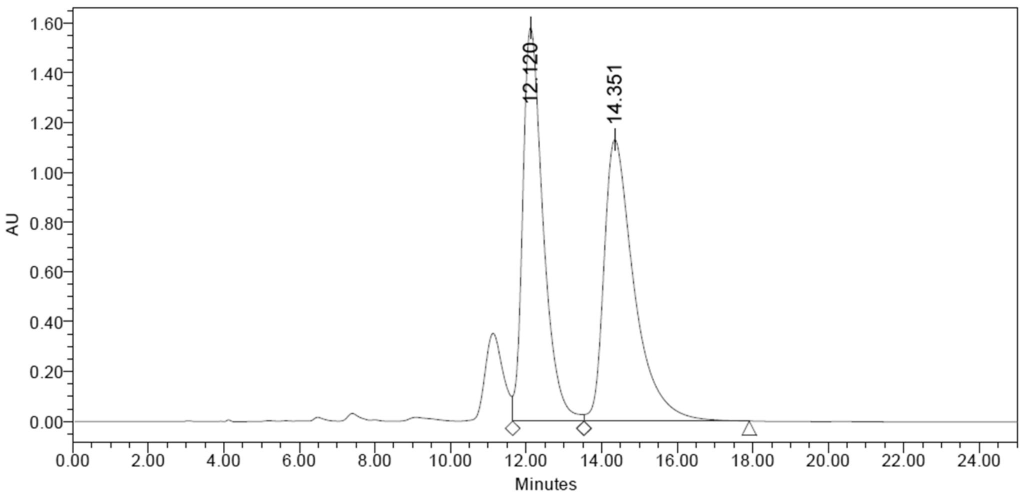

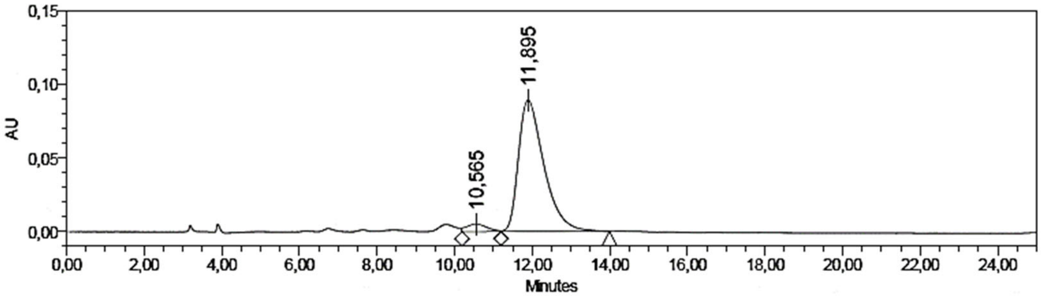

	RT	Area	Height	% Area
1	10,565	189142	5256	4,43
2	11,895	4075833	89391	95,57

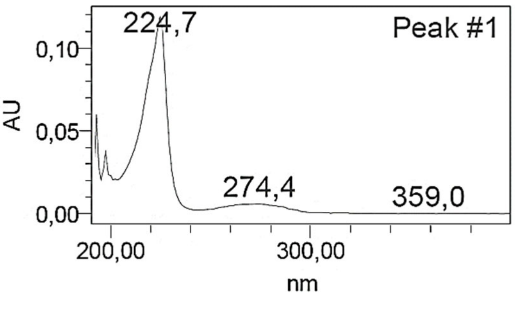

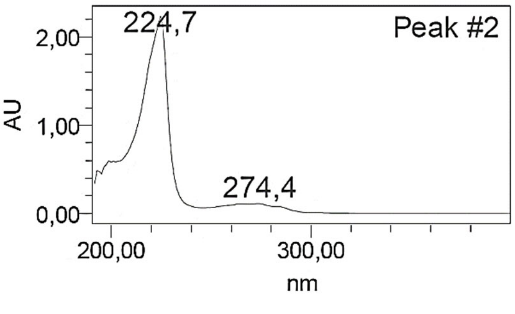

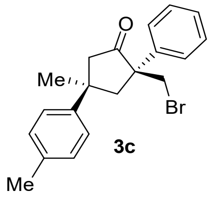

3c

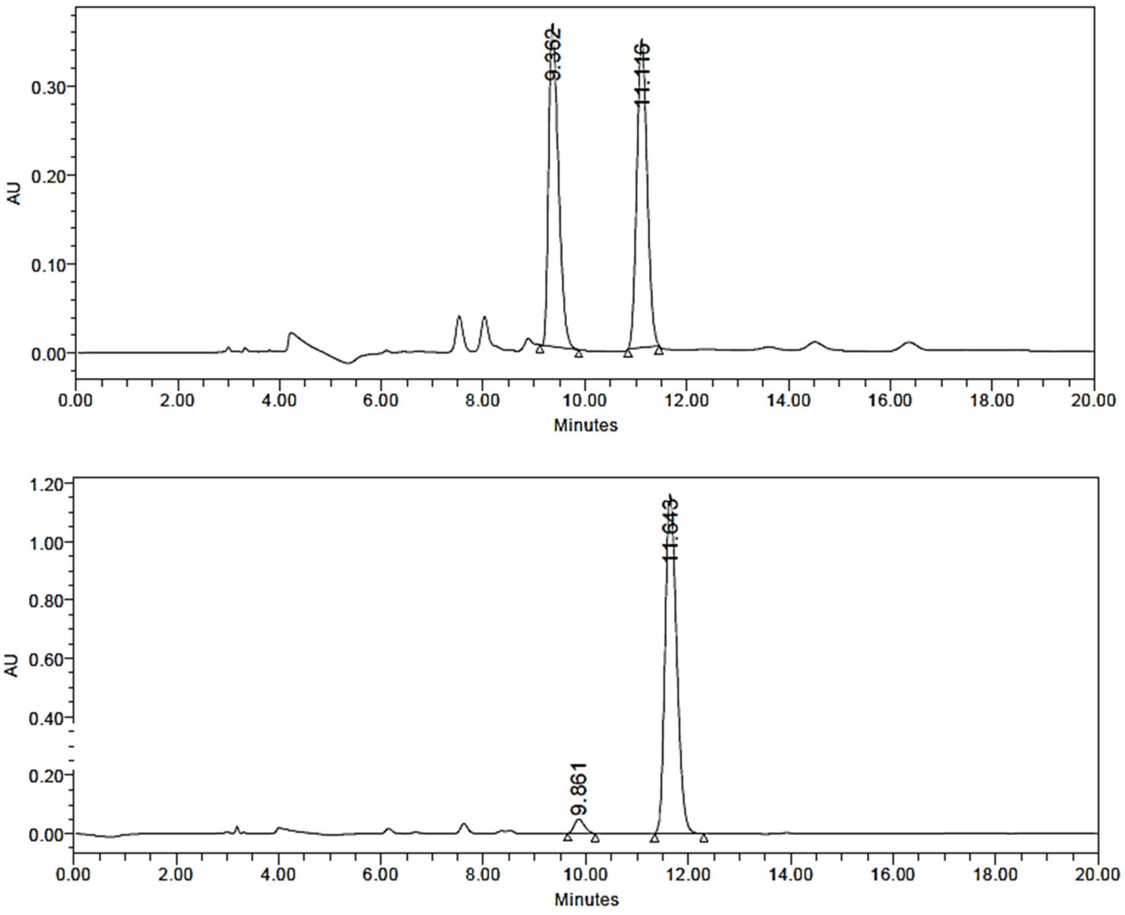

	RT	Area	% Area	Height
1	9.861	677340	3.58	49539
2	11.643	18259198	96.42	1159915

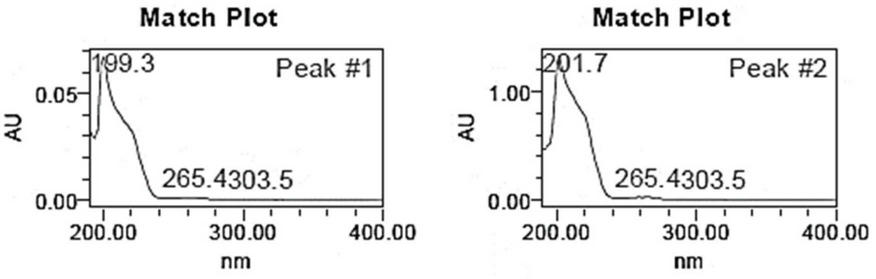

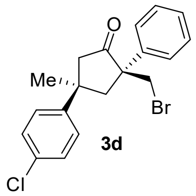

3d

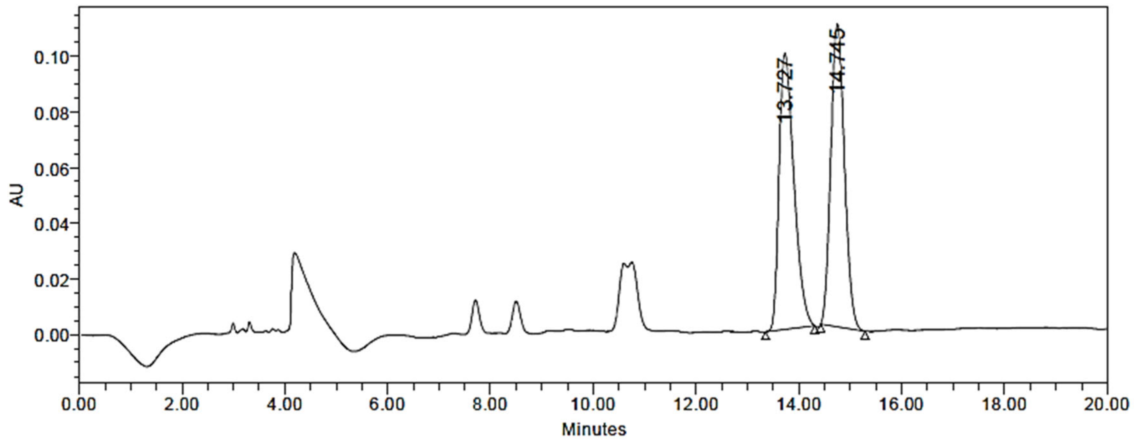

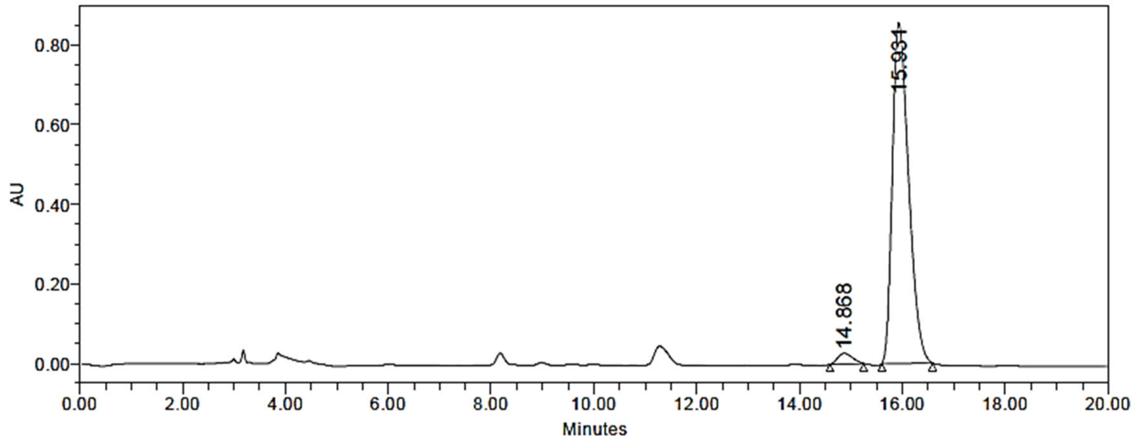

	RT	Area	% Area	Height
1	14.868	537321	2.79	26877
2	15.931	18752930	97.21	853895

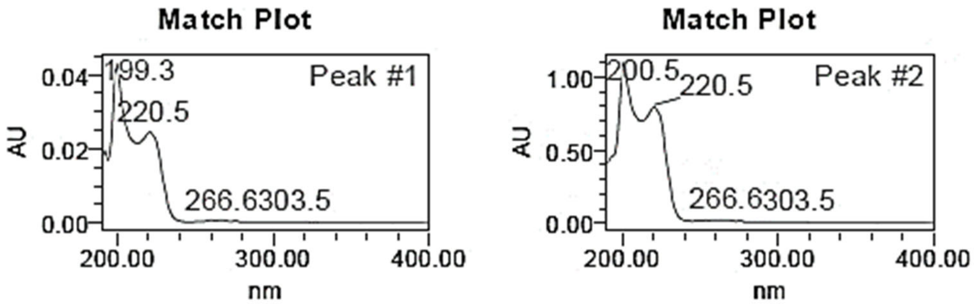

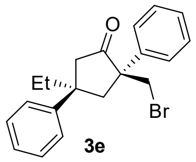

3e

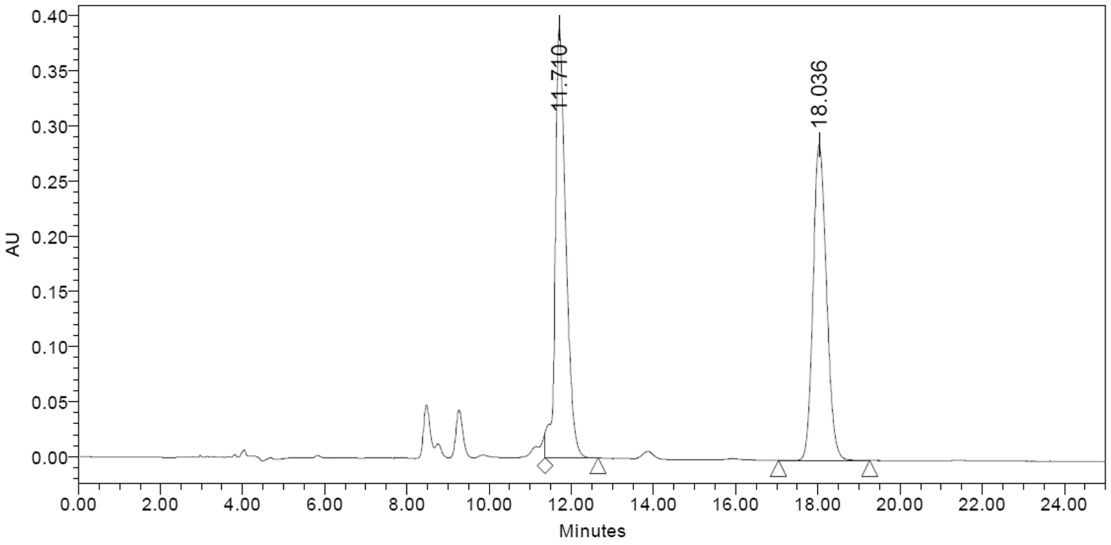

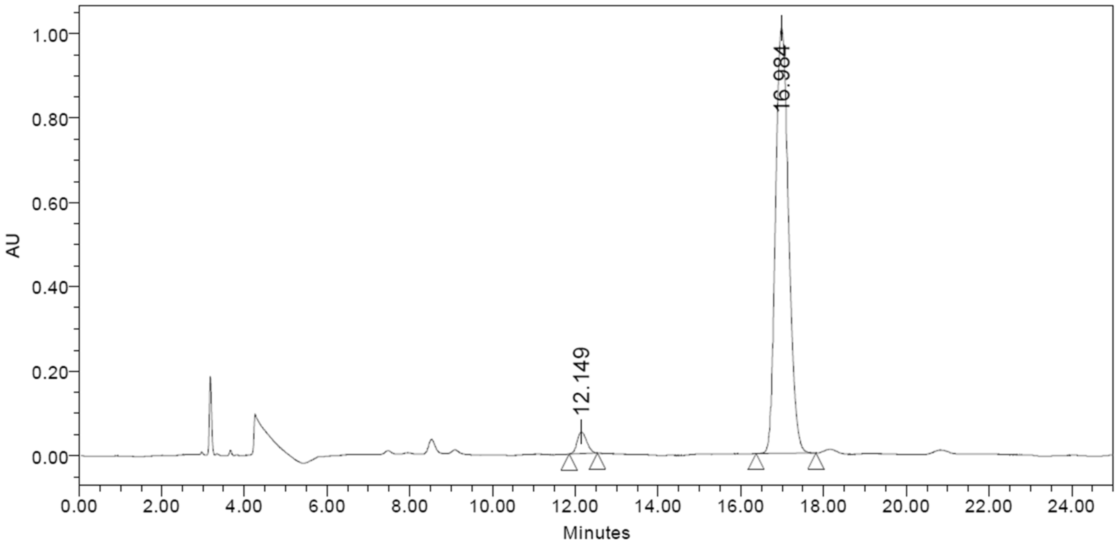

	Injection	RT	Area	% Area
1	1	12.149	814443	3.55
2	1	16.984	22135034	96.45

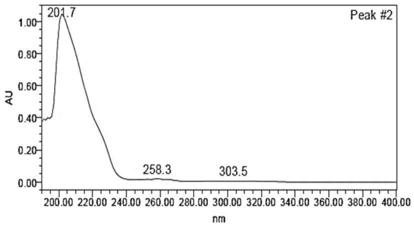

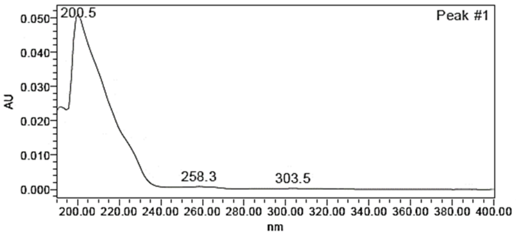

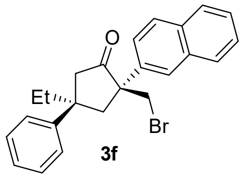

3f

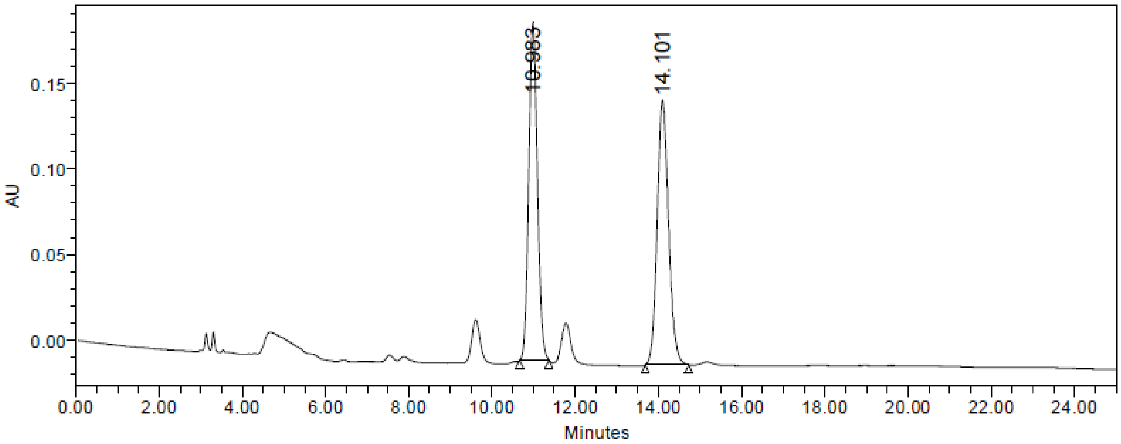

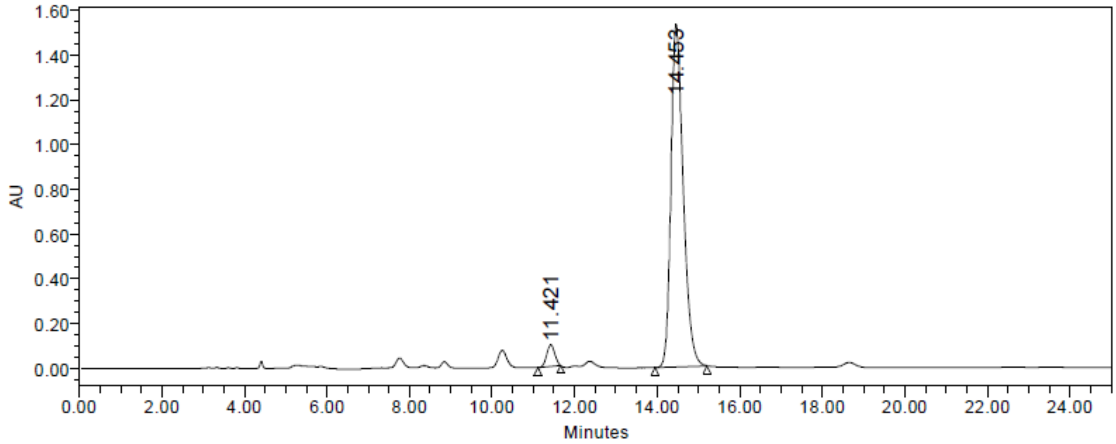

	RT	Area	% Area	Height
1	11.421	1315431	4.03	96398
2	14.453	31290341	95.97	1528391

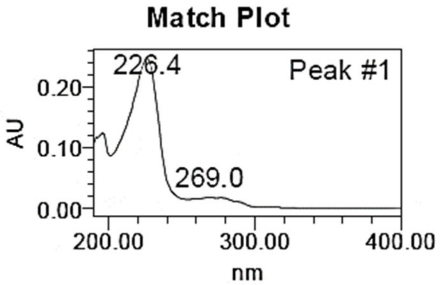

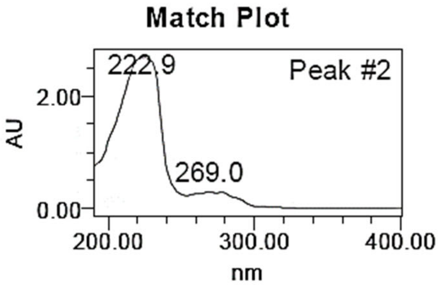

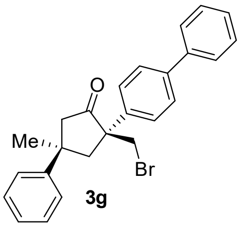

3g

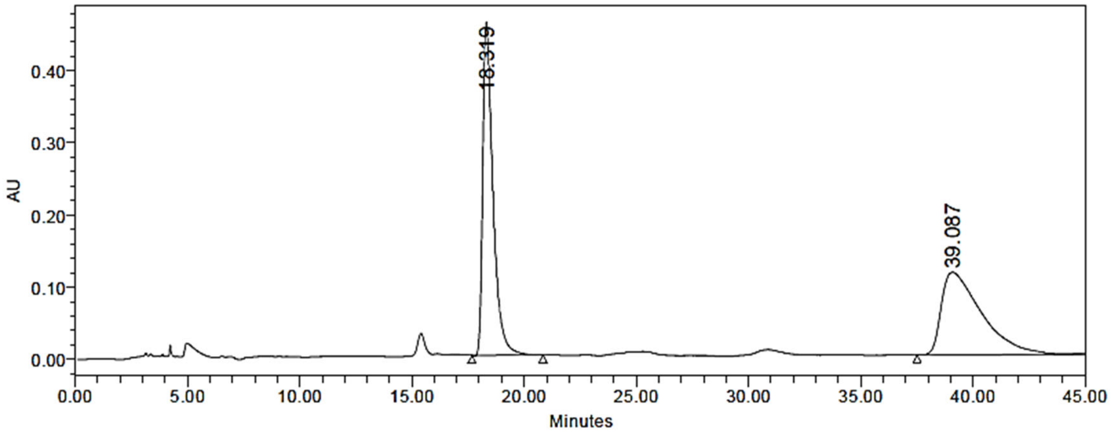

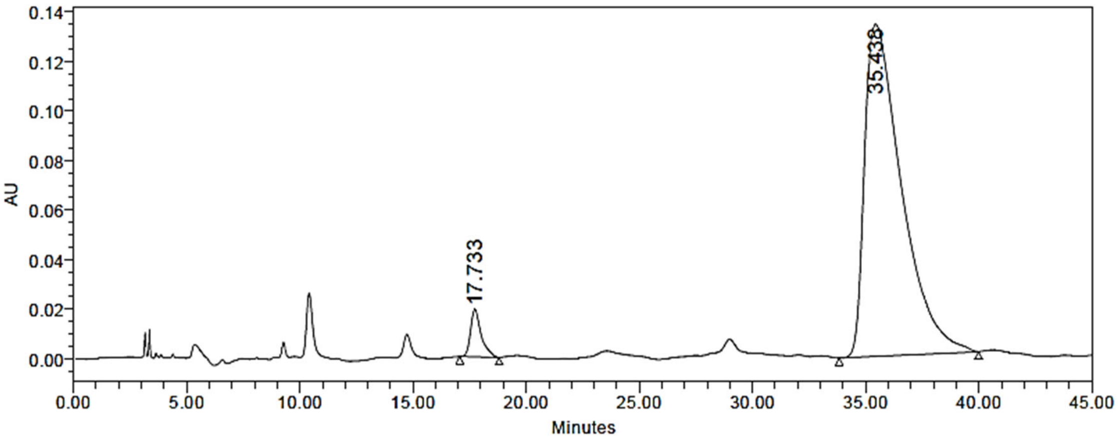

	RT	Area	% Area	Height
1	17.733	628218	3.97	19161
2	35.438	15204624	96.03	134084

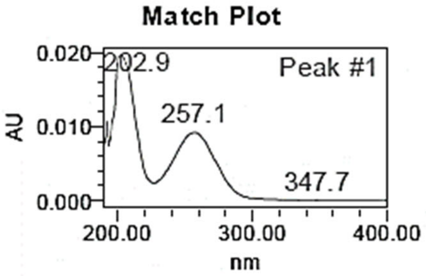

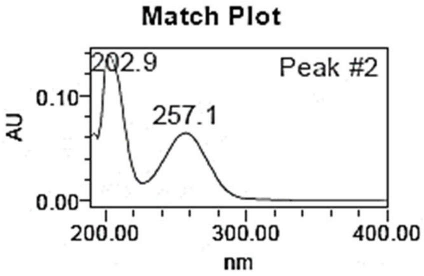

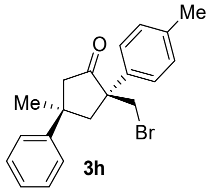

3h

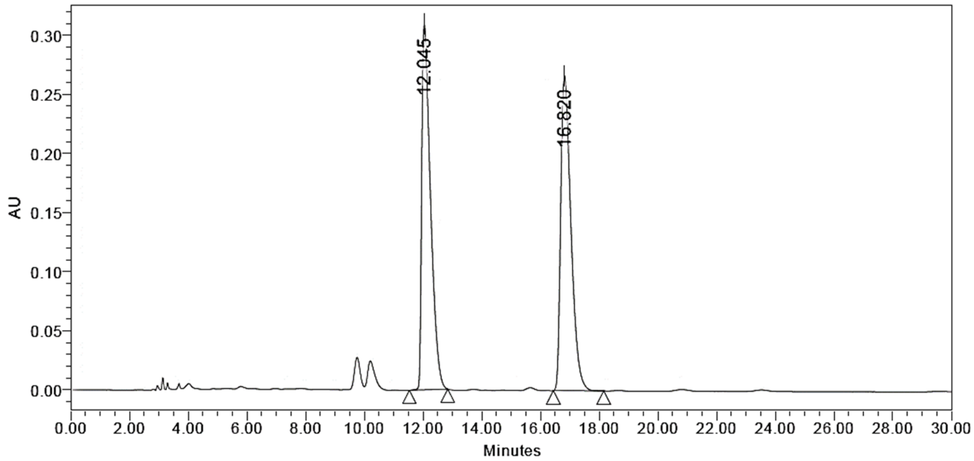

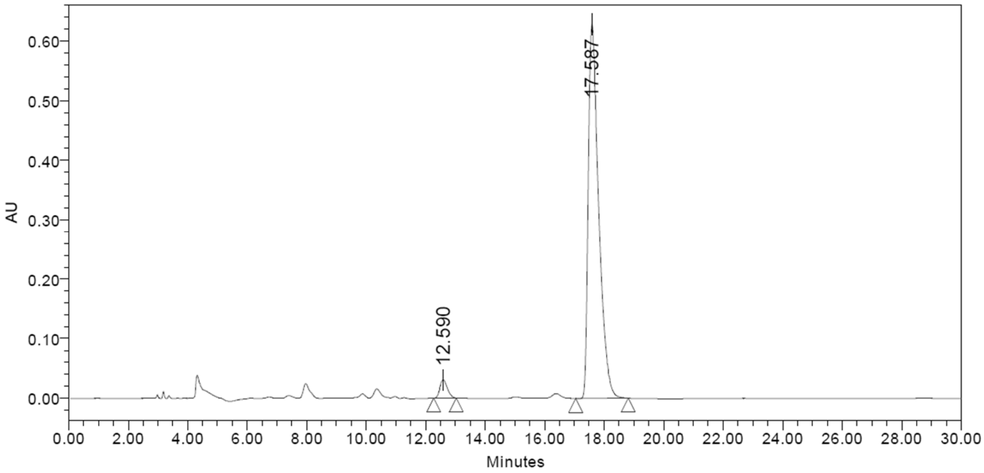

	Injection	RT	Area	% Area
1	1	12.590	528568	3.34
2	1	17.587	15307050	96.66

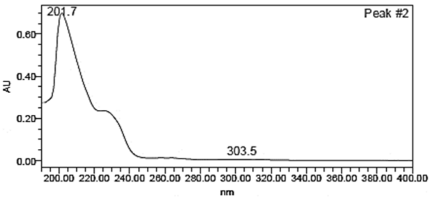

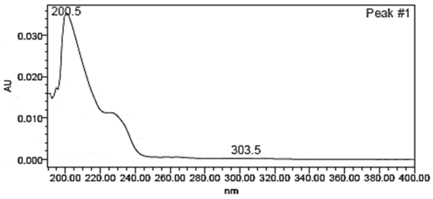

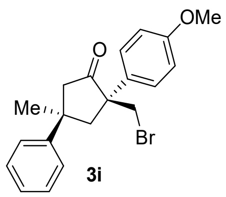

3i

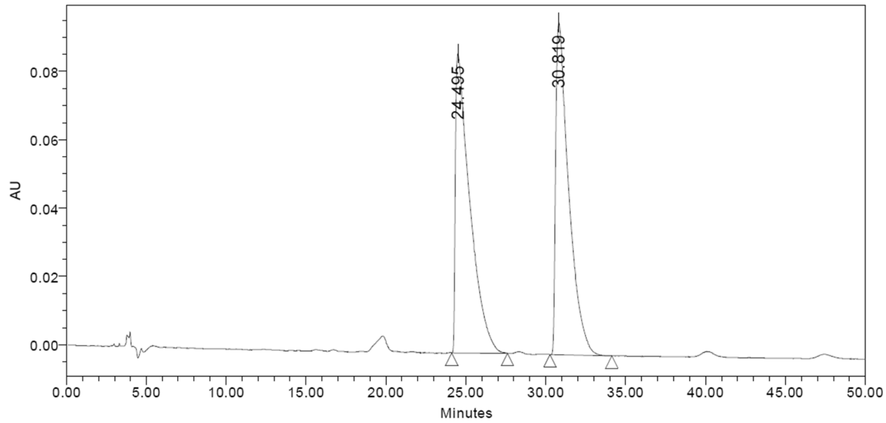

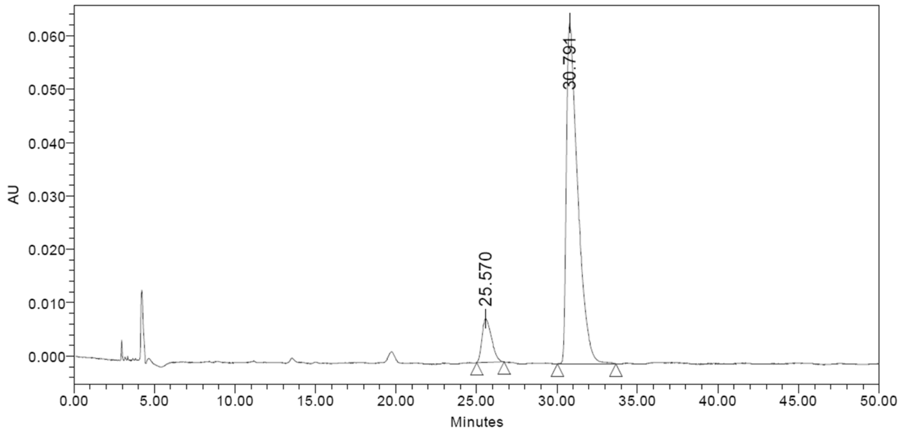

	Injection	RT	Area	% Area
1	1	30.792	1685638	90.31
2	1	25.566	180798	9.69

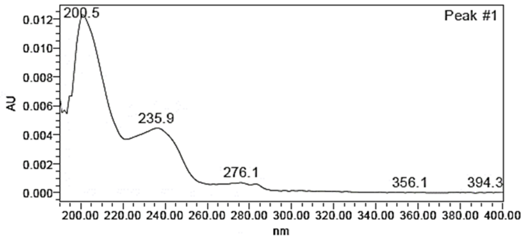

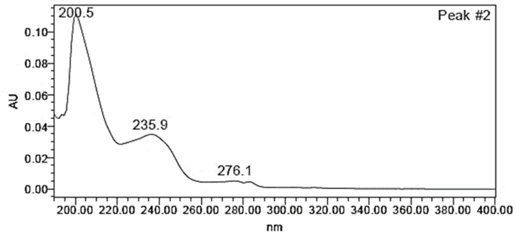

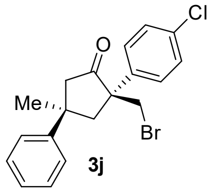

3j

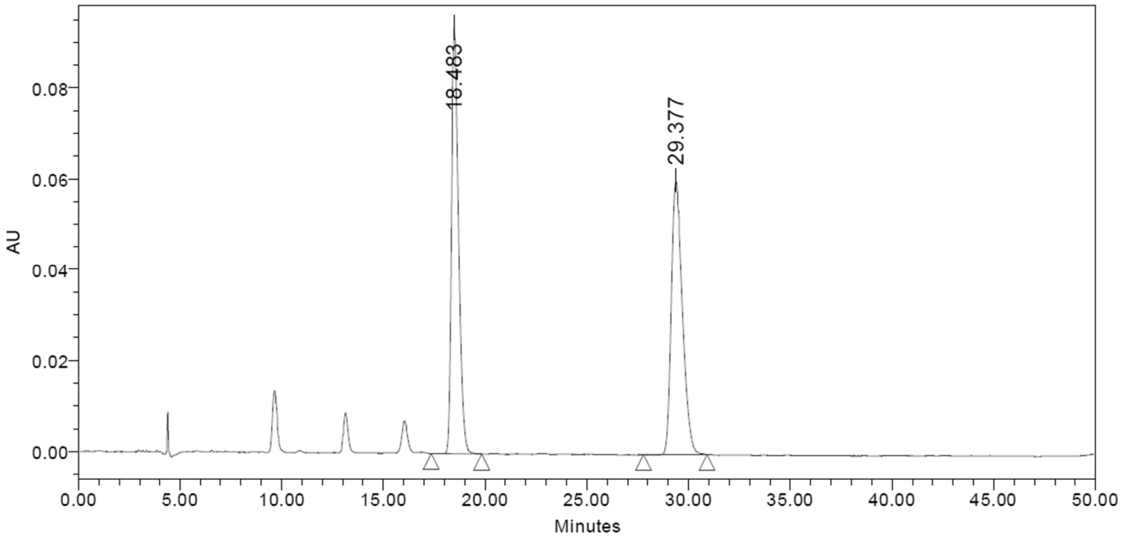

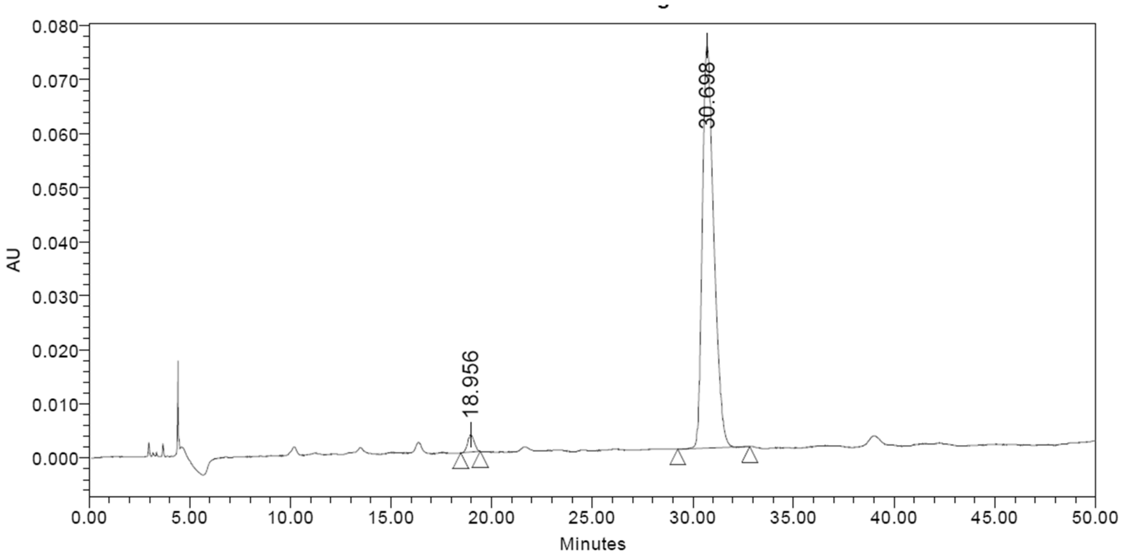

	Injection	RT	Area	% Area
1	1	18.960	46809	2.42
2	1	30.699	1887357	97.58

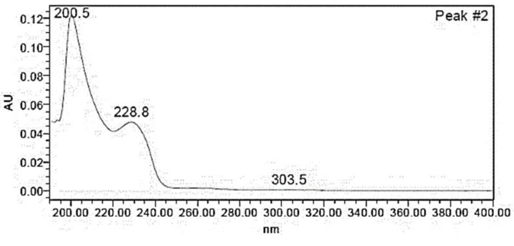

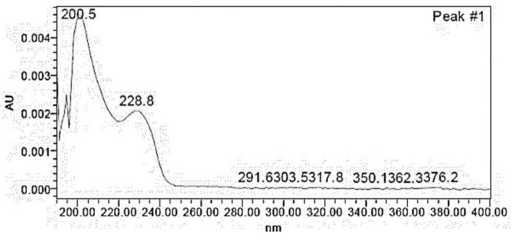

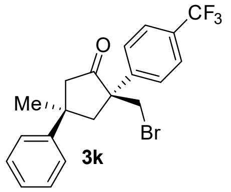

3k

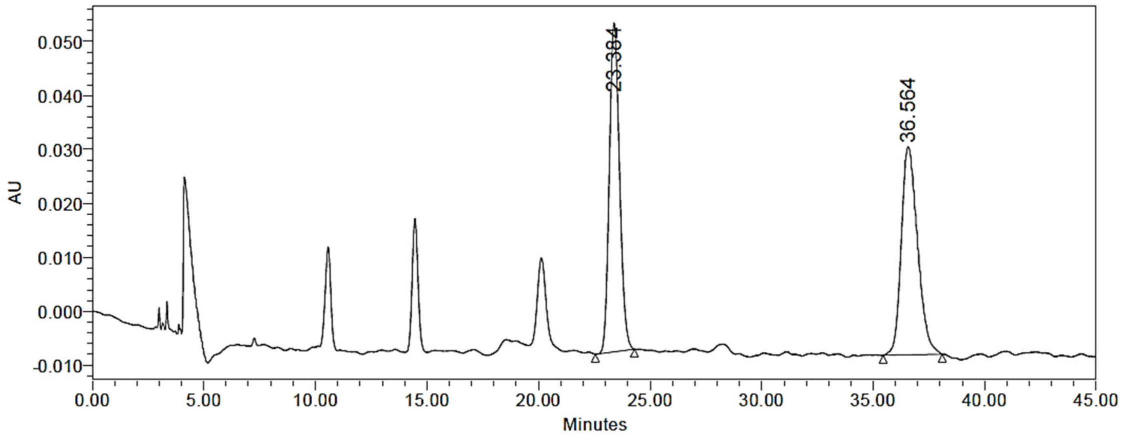

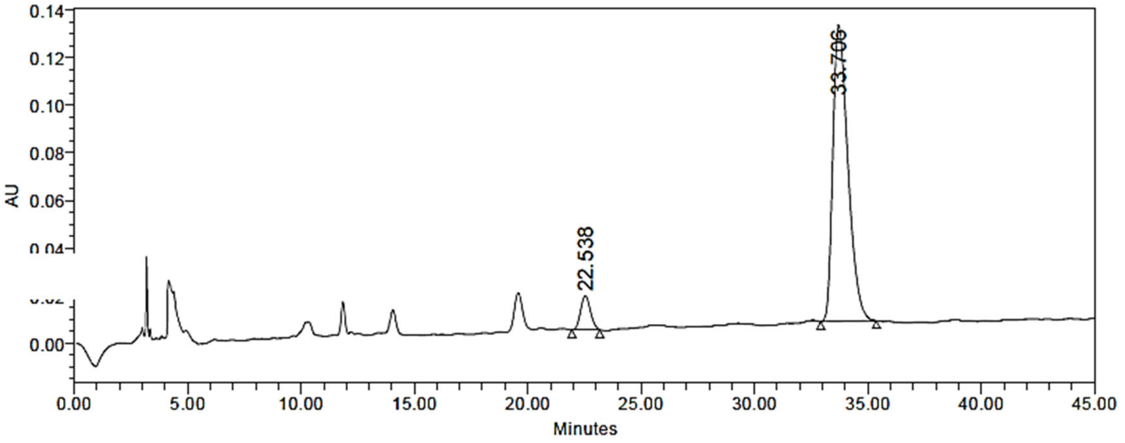

	RT	Area	% Area	Height
1	22.538	430251	6.92	14329
2	33.706	5790716	93.08	124214

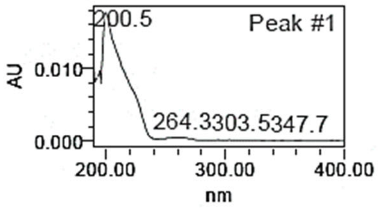

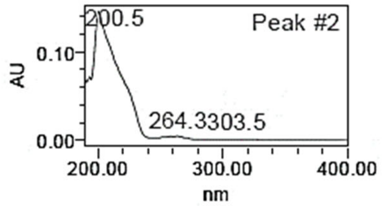

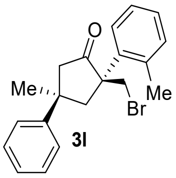

3l

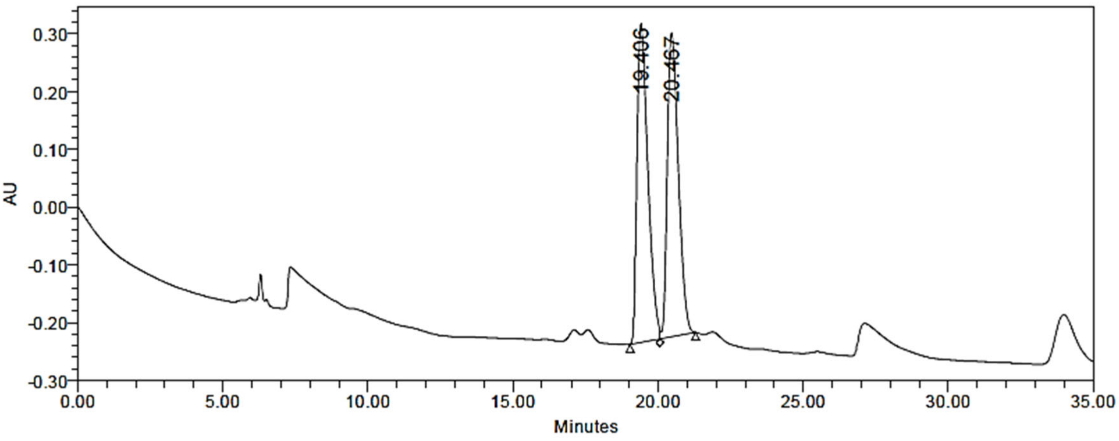

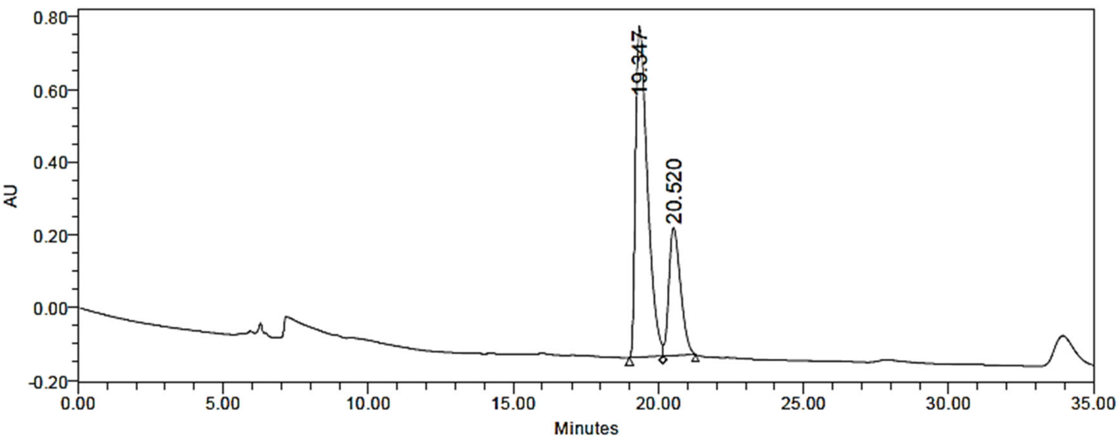

	RT	Area	% Area	Height
1	19.347	25694210	72.36	908764
2	20.520	9816905	27.64	351098

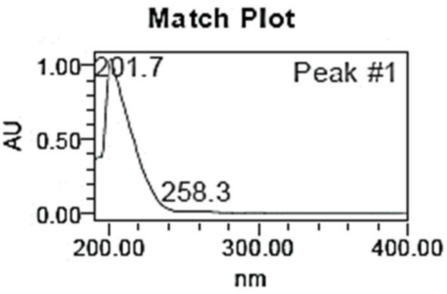

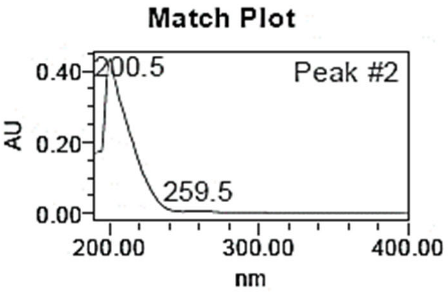

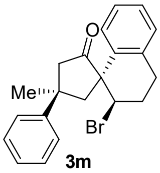

3m

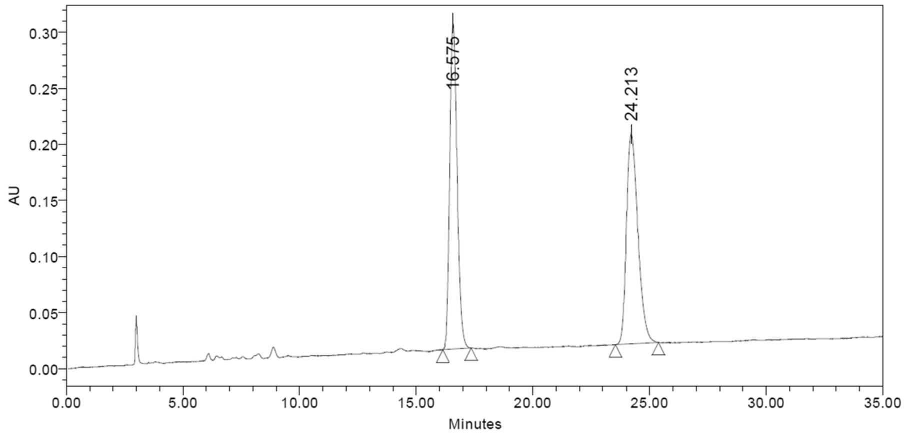

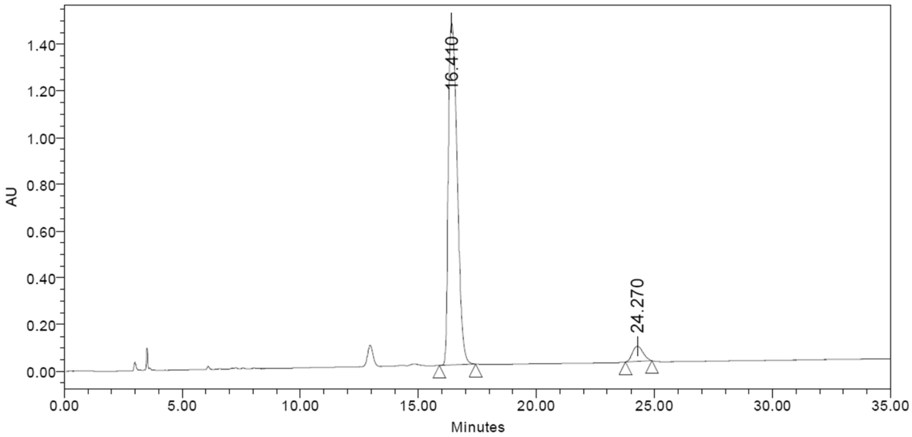

	Injection	RT	Area	% Area
1	1	24.270	2038597	5.09
2	1	16.410	38036398	94.91

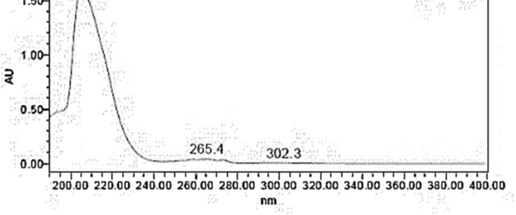

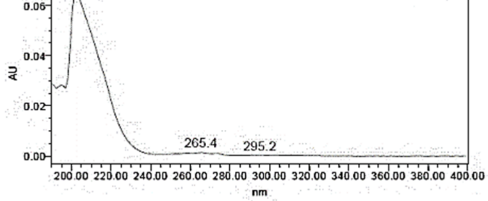

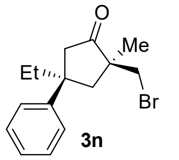

3n

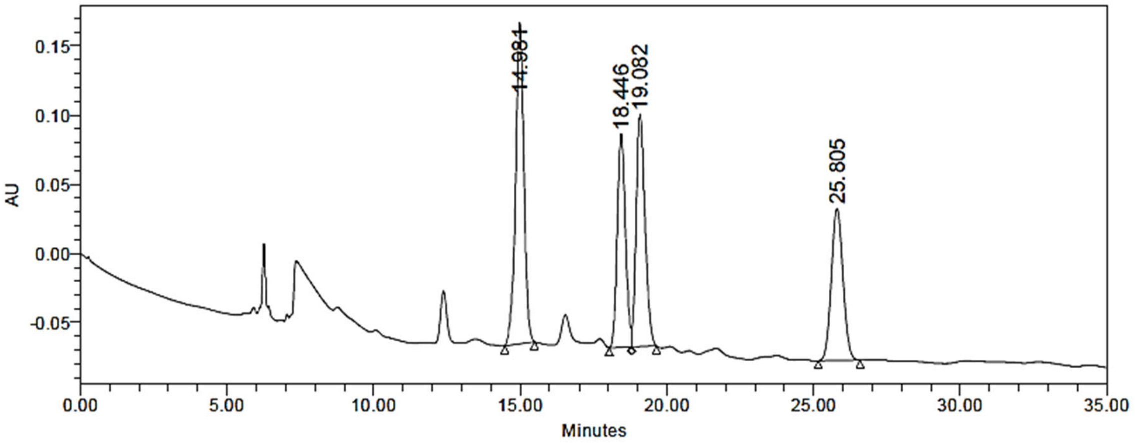

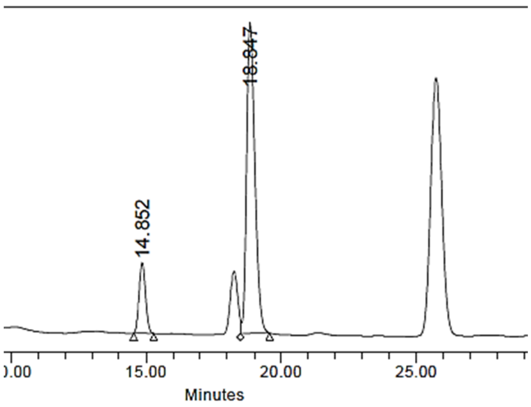

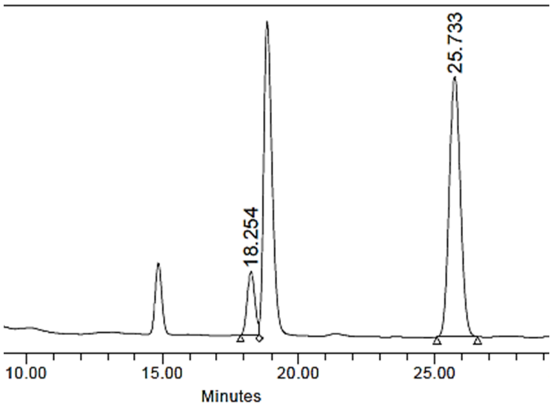

	RT	Area	% Area	Height
1	14.852	1701156	14.81	103840
2	18.847	9786127	85.19	457435

	RT	Area	% Area	Height
1	18.254	1763463	14.20	92327
2	25.733	10654502	85.80	380254

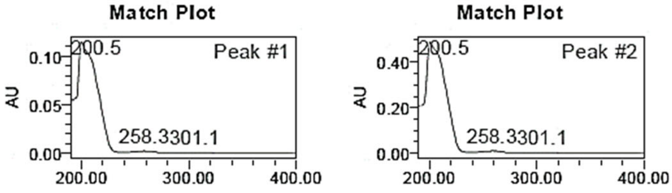

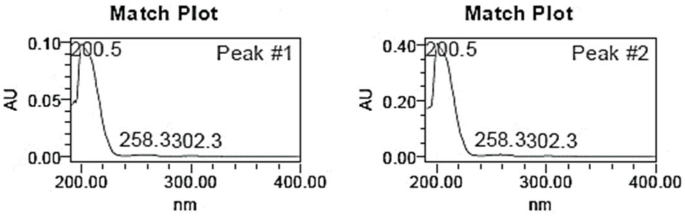

Computational Studies

The Catalyst

A close inspection of the minimized structure of the catalyst revealed a linear disposition for the coordination of the magnesium atom due to the large size of the triphenylsilyl moieties (Figure S9), in a similar way to that reported for a chiral Pd-bis-phosphate catalyst.[17] In our case, two aromatic rings from one phosphate and one from the other phosphate compose a cage in which the metal atom can interact favorably with the electron density of the aromatic rings as evidenced in the NCI analysis (Figure S10) in a similar way to that reported for cationic magnesium π-arene complexes.[18]

The structure of the catalyst does not allow the simultaneous coordination of the substrate and the NBS as required in model **A** (see main text) without any previous decoordination. Moreover, the arene stabilization might allow the formation of an ionic pair presenting η^3-arene-Mg interactions leading to model **B** (see main text) after interaction with the substrate.

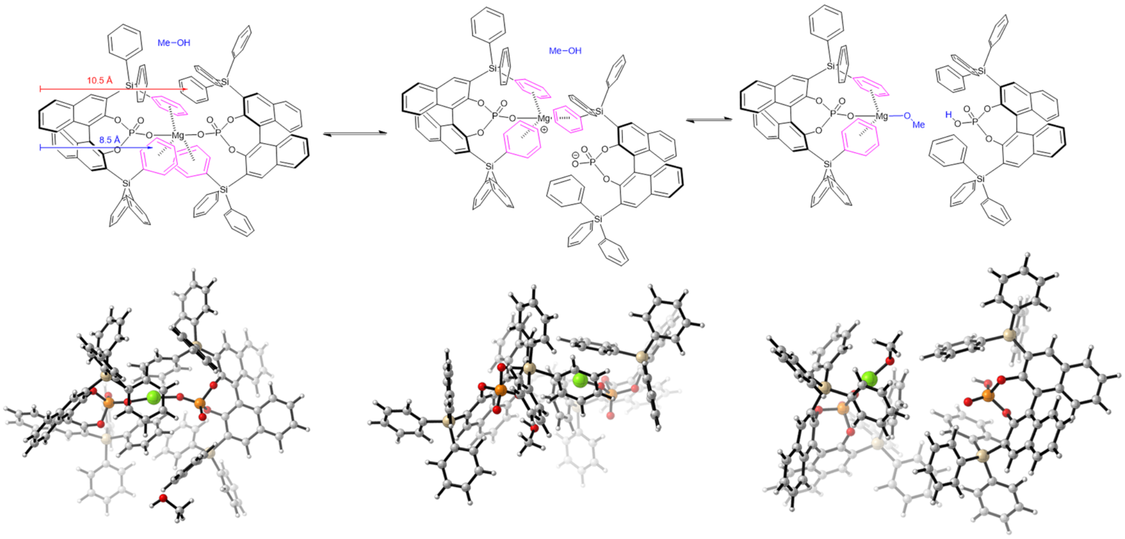

Figure S 54. Optimized structure (PM6) of the catalyst **2b** as a complex (**CAT**) and as an ionic air (**IP**) (in the presence of a molecule of MeOH), and once the alcohol is deprotonated and coordinated to form a reactive complex **RC** with an only unit of phosphate (the other is protonated in the form of phosphoric acid). Relative energies are given in kcal/mol.

---

[17] Jindal, G.; Sunoj, R. B. Deciphering the Origin of Stereoinduction in Cooperative Asymmetric Catalysis Involving Pd(II) and a Chiral Brønsted Acid. *Org. Lett.* **2015**, *17*, 2874-2877.

[18] Pahl, J.; Friedrich, A.; Elsen, H.; Harder, S. Cationic Magnesium π–Arene Complexes. *Organometallics* **2018**, *37*, 2901-2909.

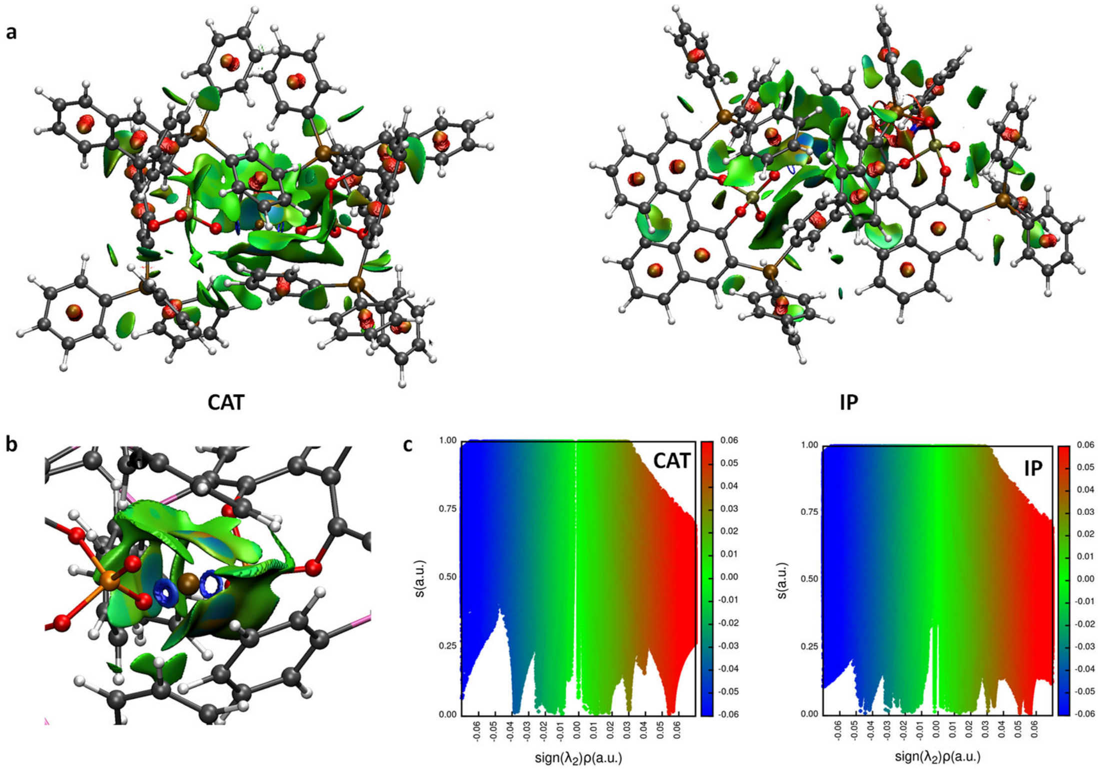

Figure S 55. a: NCI analyses for the catalyst as a complex (**CAT**) and as an ionic air (**IP**). **b**: Details of NCI interactions between the metal and the ligand. Thin, delocalized green surface indicates van der Waals interactions. Small, lenticular, bluish surfaces indicate strong interactions such as hydrogen bonding. Steric clashes are shown as red isosurfaces. **c**: s(**r**) against sign(λ2)ρ(**r**) plots for **CAT** (left) and **IP** (right). Green area corresponds to weak van der Waals interactions. Blue area corresponds to strong interactions and red area corresponds to repulsive forces.

It is plausible to hypothesize the formation of an ionic pair and further coordination of the alcohol to the magnesium atom thus leading to model **B** and liberating one unit of phosphoric acid, with a cost (in the case of MeOH) of ca. 15 kcal/mol.

Unsubstituted Model

Preliminary studies were carried out with an unsubstituted model **EC1** (Scheme S 14). These studies suggested the non-existence of an intermediate **IN** but a reaction in one kinetic step presenting a transient carbocation. Considering the whole reaction (bromination followed by rearrangement) there are two possible approaches for the formation of the epoxide and four ones for the formation of cyclopentanone. Taking into account that coordination with magnesium should be maintained, two approaches should be ruled out (Scheme S 14). In consequence, there are one approach (**TSa)** for obtaining the epoxide and four approaches (**TSb-d**) leading to the enantiomers of the cyclopentanone. The absence of cyclobutane susbituents in this model makes that

rearrangement of bonds C2-C3 and C2-C5 are equivalent leading to enantiomeric compounds.

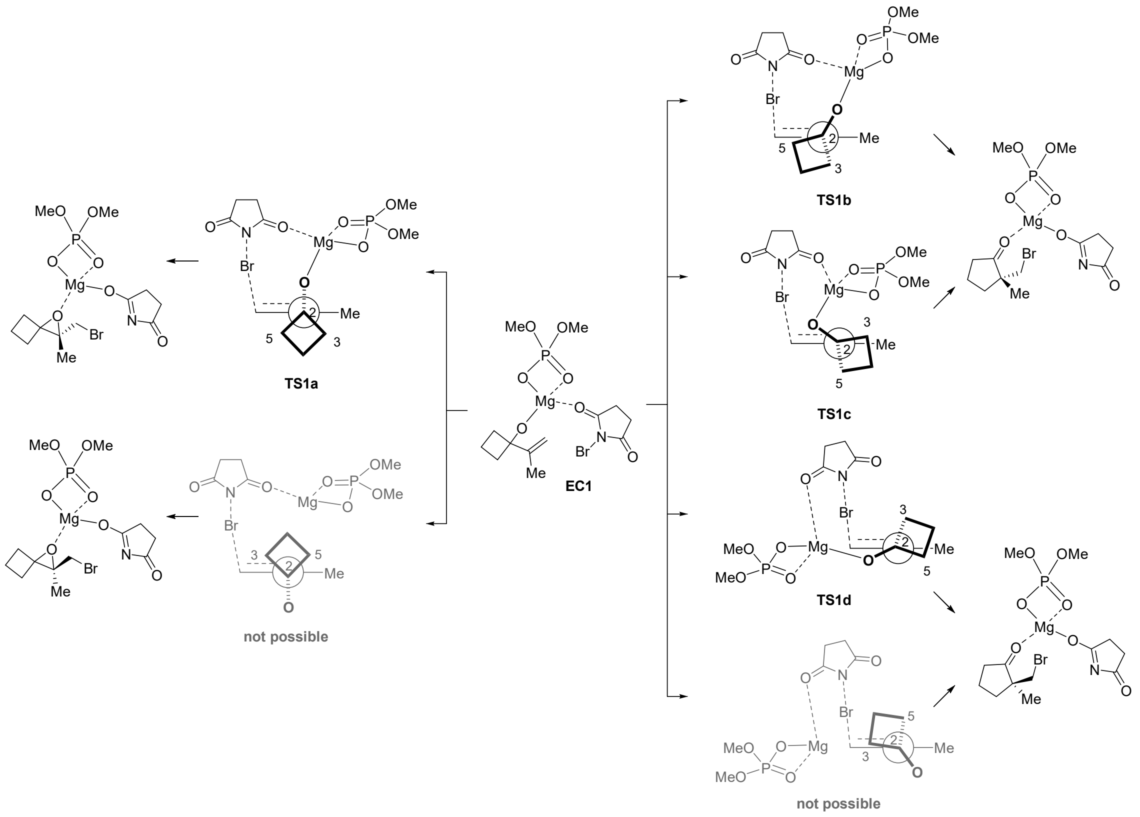

Scheme S 13. Approaches (PES) for the unsubstituted model **EC1**

The four transition structures as well as the starting minimum (**EC1**) and final products (**EP1** and **CY1**) were located. The optimized geometries are given in Figure S 56 and Figure S 57, respectively.

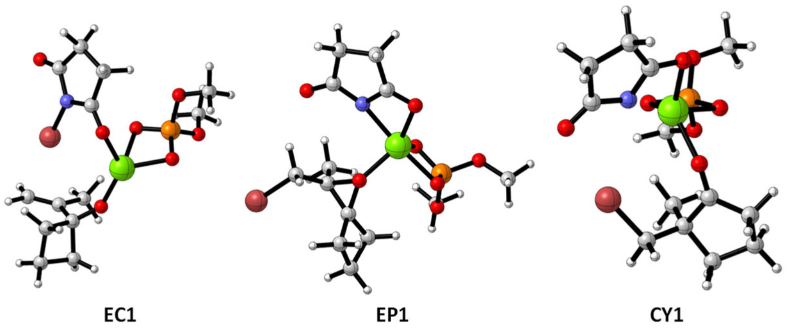

Figure S 56. Optimized (wb97xd/def2SVP) geometries of encounter complex **EC1**, epoxide **EP1** and cyclopentanone **CY1**.

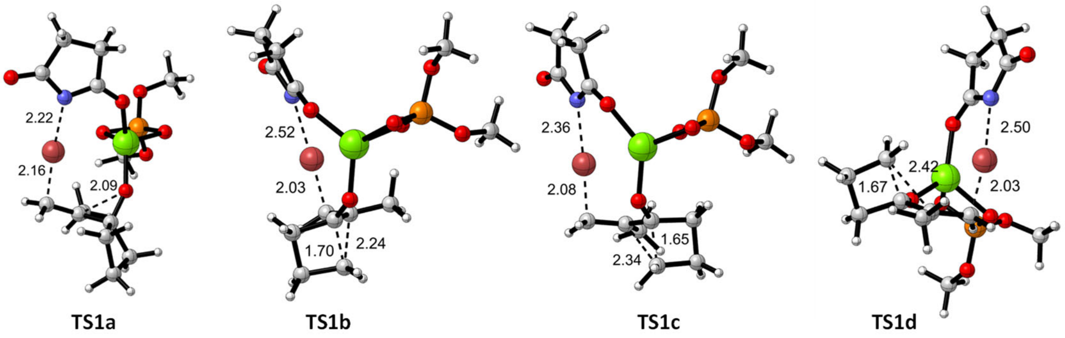

Figure S 57. Optimized (wb97xd/def2SVP) geometries of transition structures **TS1a**, **TS1b**, **TS1c** and **TS1d**.

The IRC analyses confirming that transition structures connect reagent and products are given in Figure S 58. Interestingly, the IRCs corresponding to **TS1b** and **TS1d** showed a clear shoulder typical of a “hidden intermediate”.

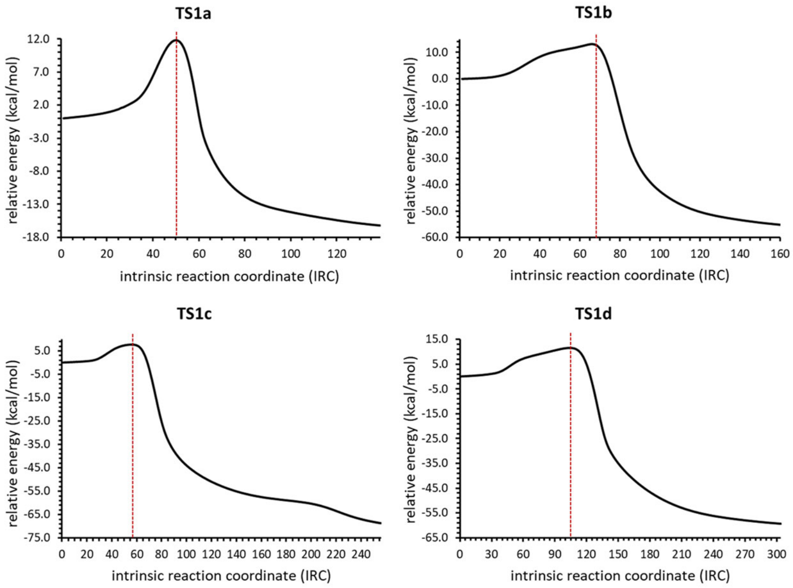

Figure S 58. IRCs (wb97xd/def2svp) corresponding to transition structures **TS1a**, **TS1b**, **TS1c** and **TS1d**. The red dotted line indicates the transition state.

Energy profiles are given in Figure S 59. Absolute and relative energies are listed in Table S 100.

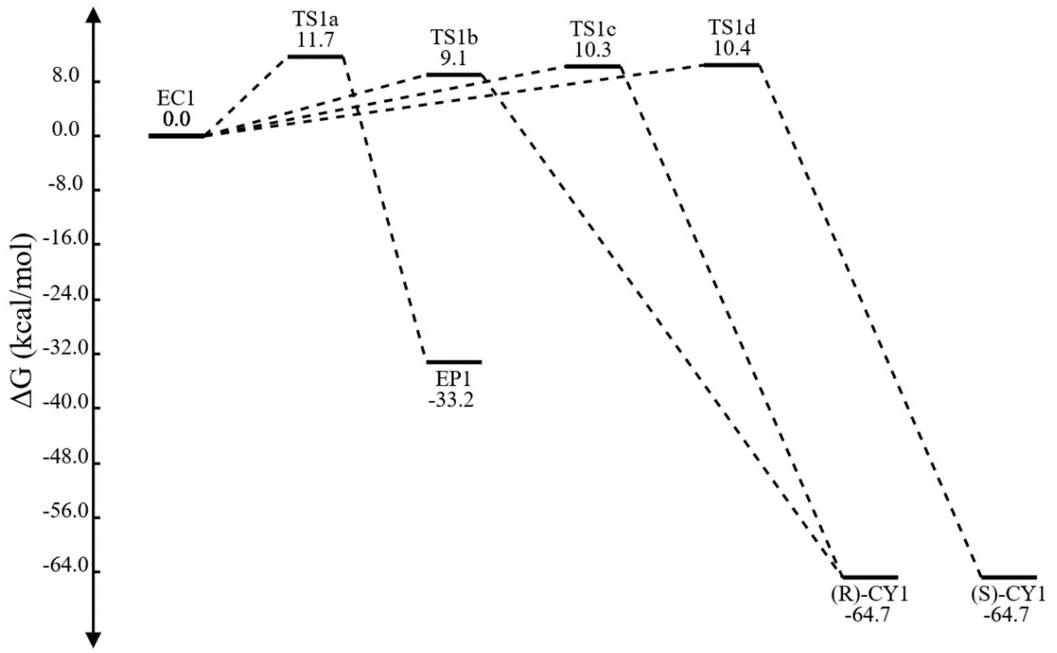

Figure S 59. Energy profiles (wb97xd/def2tzvp/pcm=toluene)//wb97xd/def2svp) for the different pathways from **EC1** to **EP1** and **CY1.** Relative free energies are given in kcal/mol.

Table S 10. Calculated (wb97xd/def2tzvp/pcm=toluene//wb97xd/def2svp) absolute (hartree) and relative (kcal/mol) energies for the transformation of **EC1** into **EP1** and **CY1**.

	E_0	ΔE_0	G	ΔG	im. freq
**EC1**	-4204.993364	0.0	-4205.074060	0.0	
**TS1a**	-4204.974328	11.9	-4205.055441	11.7	-227.6
**TS1b**	-4204.978509	9.3	-4205.059523	9.1	-285.9
**TS1c**	-4204.977618	9.9	-4205.057617	10.3	-132.3
**TS1d**	-4204.976291	10.7	-4205.057505	10.4	-125.8
**CY1**	-4205.094731	-63.6	-4205.177200	-64.7	
**EP1**	-4205.044295	-32.0	-4205.127047	-33.2	

An exploration of the potential energy surface (PES) corresponding to the second part of the reaction, i.e. formation of either the O1-C1 bond (epoxide) or C2-C3 bond (cyclopentanone) was made by carrying out relaxed scans at the different O1-C1 and C2-C3 distances. The exploration covered sampling of conformations and in all cases the most stable conformers have been considered. The resulting PES (Figure S 61) confirmed two different transition structures for the epoxide and cyclopentanone formation and the absence of a reasonable connection between the two products, in agreement with the experimental observations.

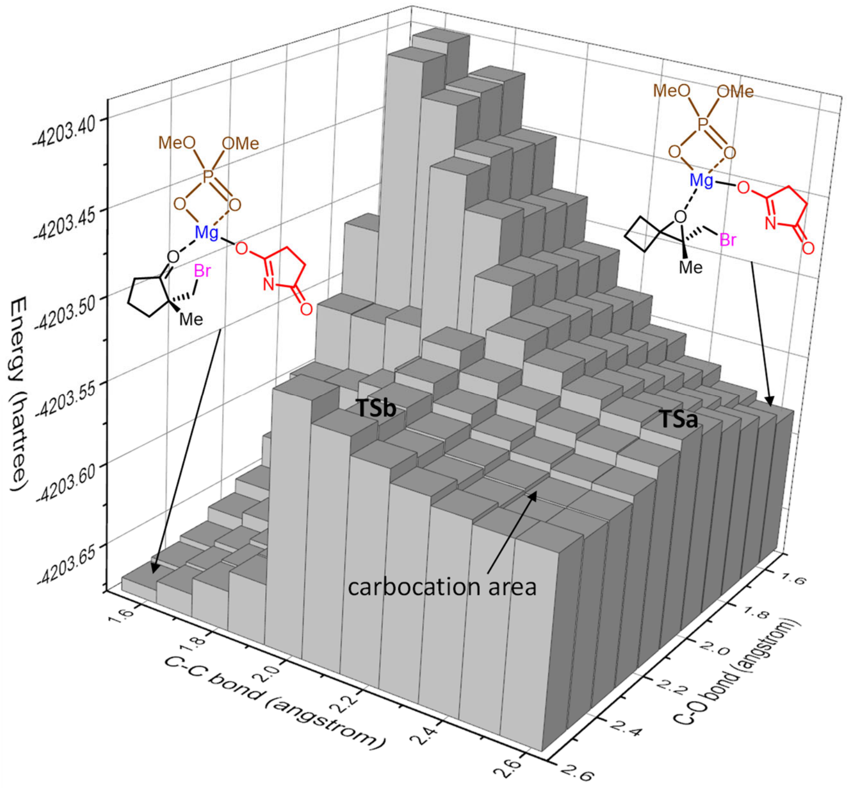

Figure S 60. Potential energy surface (wb97xd/def2svp) related to the formation of O1-C1 and C2-C3 bonds corresponding to the epoxide and the cyclopentanone, respectively.

Benchmarking

Three additional levels of theory were calculated confirming that the employed level is the closest to experimental results even though all the levels showed the same trend.

Table S 11. Calculated (m062x/cc-pvtz/pcm=toluene//m062x/cc-pvdz) absolute (hartree) and relative (kcal/mol) energies for the transformation of **EC1** into **EP1** and **CY1**.

	E_0	ΔE_0	G	ΔG	im. freq
**EC1**	-4204.873013	0.0	-4204.934009	0.0	
**TS1a**	-4204.849887	14.5	-4204.911619	14.1	-291.8
**TS1b**	-4204.852338	13.0	-4204.914097	12.5	-317.3
**TS1c**	-4204.853413	12.3	-4204.913827	12.7	-174.4
**TS1d**	-4204.850983	13.8	-4204.912320	13.6	-144.3
**CY1**	-4204.969752	-60.7	-4205.031266	-61.0	
**EP1**	-4204.924108	-32.1	-4204.986001	-32.6	

Table S 12. Calculated (b3lyp-gd3bj/6-311G(d,p)//b3lyp-gd3bj/6-31G(d,p)) absolute (hartree) and relative (kcal/mol) energies for the transformation of **EC1** into **EP1** and **CY1**.

	E_0	ΔE_0	G	ΔG	im. freq
**EC1**	-4205.215671	0.0	-4205.276833	0.0	
**TS1a**	-4205.203256	7.8	-4205.264809	7.5	-153.1
**TS1b**	-4205.200089	9.8	-4205.262251	9.2	-397.3
**TS1c**	-4205.203085	7.9	-4205.265291	7.2	-238.8
**TS1d**	-4205.199187	10.3	-4205.261305	9.7	-195.9
**CY1**	-4205.316731	-63.4	-4205.379780	-64.6	
**EP1**	-4205.257793	-26.4	-4205.320834	-27.6	

Table S 13. Calculated (m062x/6-311G(d,p)/pcm=toluene//m062x/6-31G(d,p)) absolute (hartree) and relative (kcal/mol) energies for the transformation of **EC1** into **EP1** and **CY1**.

	E_0	ΔE_0	G	ΔG	im. freq
**EC1**	-4204.631744	0.0	-4204.692064	0.0	
**TS1a**	-4204.608624	14.5	-4204.668985	14.5	-290.9
**TS1b**	-4204.610684	13.2	-4204.671587	12.8	-397.8
**TS1c**	-4204.613714	11.3	-4204.673755	11.5	-212.2
**TS1d**	-4204.609753	13.8	-4204.670650	13.4	-164.1
**CY1**	-4204.740881	-68.5	-4204.802151	-69.1	
**EP1**	-4204.686138	-34.1	-4204.747661	-34.9	

Real model with Achiral Catalyst

We studied the real model leading to desymmetrization with an achiral catalyst to evaluate the reactivity. Full studies were carried out with R = Ph and predictive studies with R = 4-$MeOC_6H_4$ and R = 4-$F_3CC_6H_4$. In a similar way to the unsubstituted model, four approaches are possible. In this case, due to the presence of substituents in the cyclobutane ring, different isomeric cyclopentanones in addition to the epoxide can be obtained (Scheme S 14).

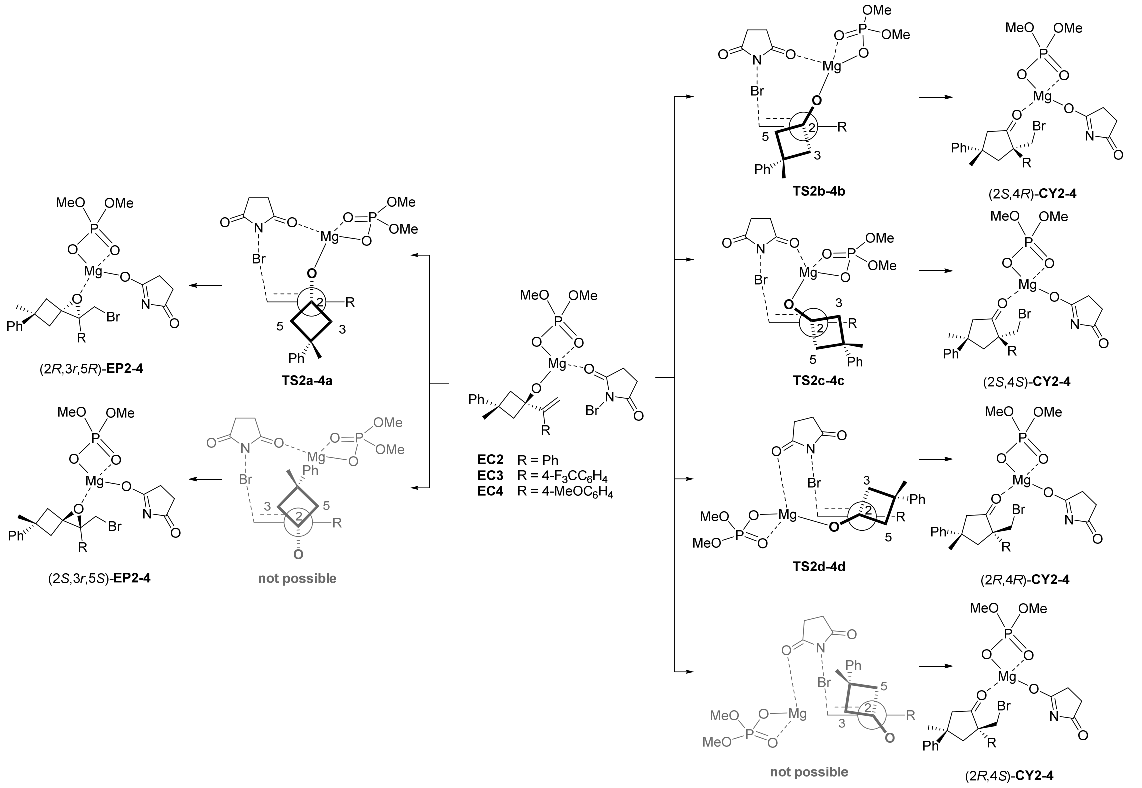

Scheme S 14. Approaches (PES) for the real models **EC2-4**

The preliminary analysis of the transition structures illustrated in Scheme S15 showed a clear competition between **TS2a** and **TS2b**, the only transition structures that show the aromatic ring planar with respect to C6-C7 bond, thus being able of contributing to the stabilization of a transient carbocation. Indeed, in both **TS2c** and **TS2d**, due to steric hindrance caused by the cyclobutane ring, the aromatic ring linked to C-6 is placed perpendicular to the double bond circumventing any conjugative effect.[19] Such a disposition facilitates π,π-interactions between the two aromatic rings linked to C4 and C6 (confirmed by NCI analysis, see below).

---

[19] This effect is also reflected in the IRCs of **TS1c** and **TS1d** that do not show the area corresponding to the transient carbocation, being more similar to that corresponding to **TS1a**.

Optimized geometries

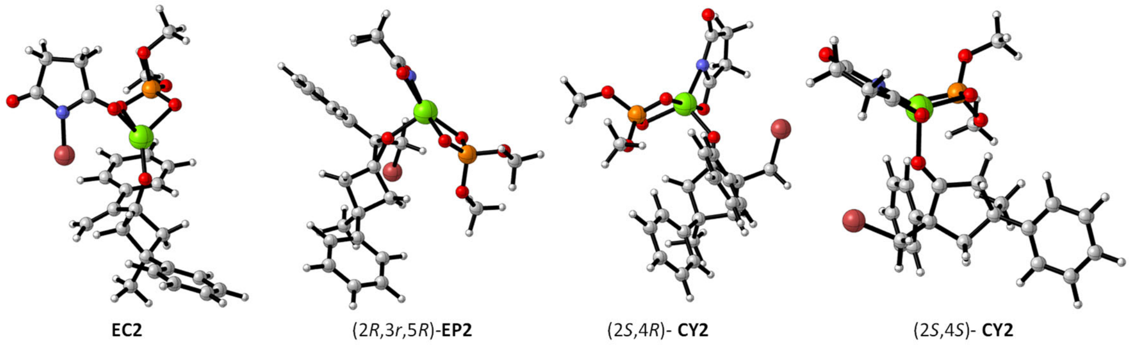

Figure S 61. Optimized (wb97xd/def2SVP) geometries of encounter complex **EC2**, epoxide (2*R*,3*r*,5*R*)-**EP2** and cyclopentanones (2*S*,4*R*)-**CY2**, (2*S*,4*S*)-**CY2** and (2*R*,4*R*)-**CY2**

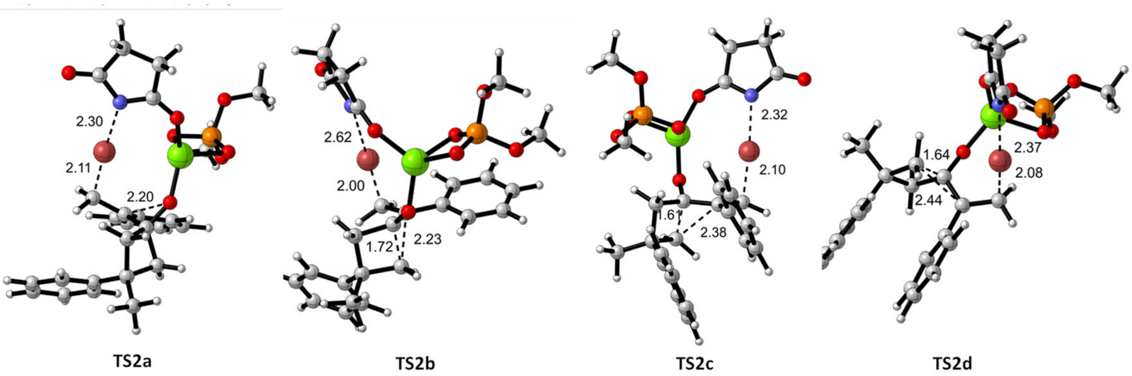

Figure S 62. Optimized (wb97xd/def2SVP) geometries of transition structures **TS2a**, **TS2b**, **TS2c** and **TS2d**.

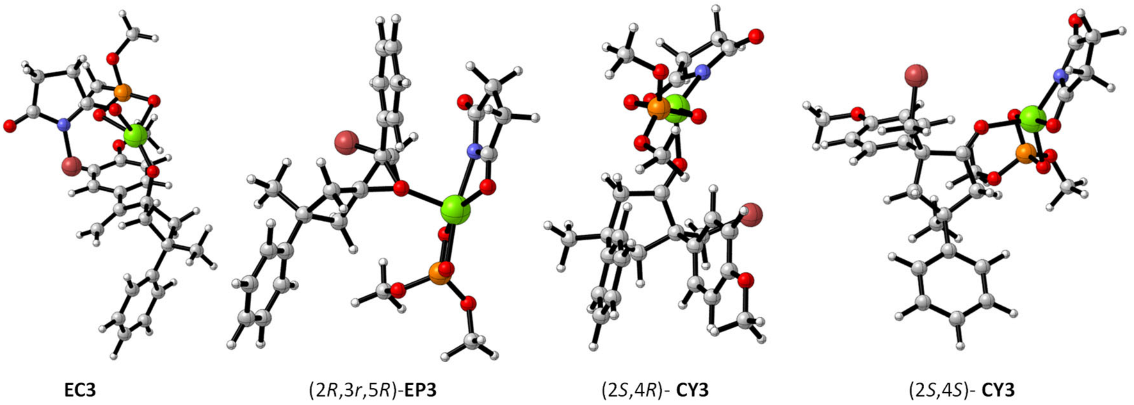

Figure S 63. Optimized (wb97xd/def2SVP) geometries of encounter complex **EC3**, epoxide (2*R*,3*r*,5*R*)-**EP3** and cyclopentanones (2*S*,4*R*)-**CY3**, (2*S*,4*S*)-**CY3** and (2*R*,4*R*)-**CY3**

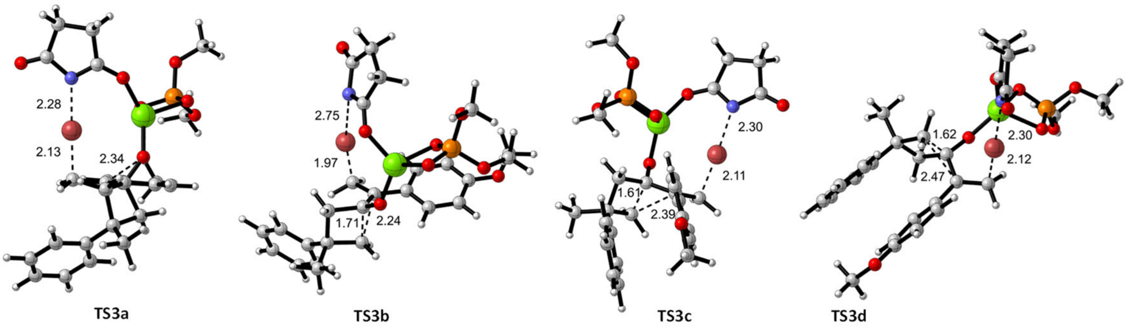

Figure S 64. Optimized (wb97xd/def2SVP) geometries of transition structures **TS3a**, **TS3b**, **TS3c** and **TS3d**.

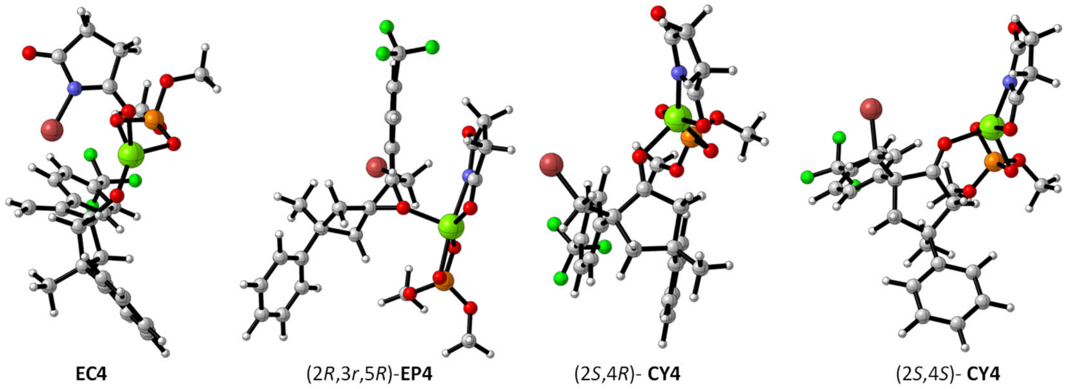

Figure S 65. Optimized (wb97xd/def2SVP) geometries of encounter complex **EC4**, epoxide (2*R*,3*r*,5*R*)-**EP4** and cyclopentanones (2*S*,4*R*)-**CY4**, (2*S*,4*S*)-**CY4** and (2*R*,4*R*)-**CY4**

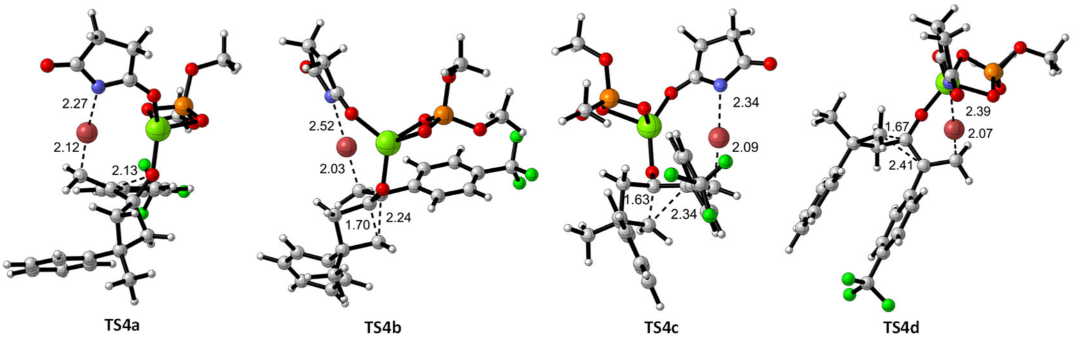

Figure S 66. Optimized (wb97xd/def2SVP) geometries of transition structures **TS4a**, **TS4b**, **TS4c** and **TS4d**.

Intrinsic reaction coordinates

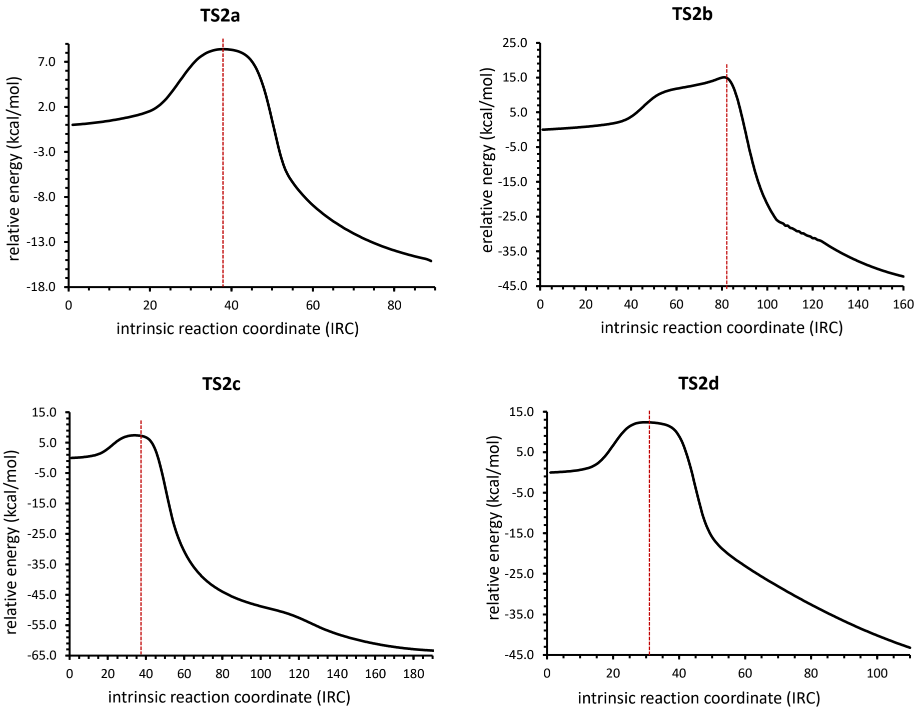

Figure S 67. IRCs (wb97xd/def2svp) corresponding to transition structures **TS2a**, **TS2b**, **TS2c** and **TS2d**. The red dashed line indicates the transition state.

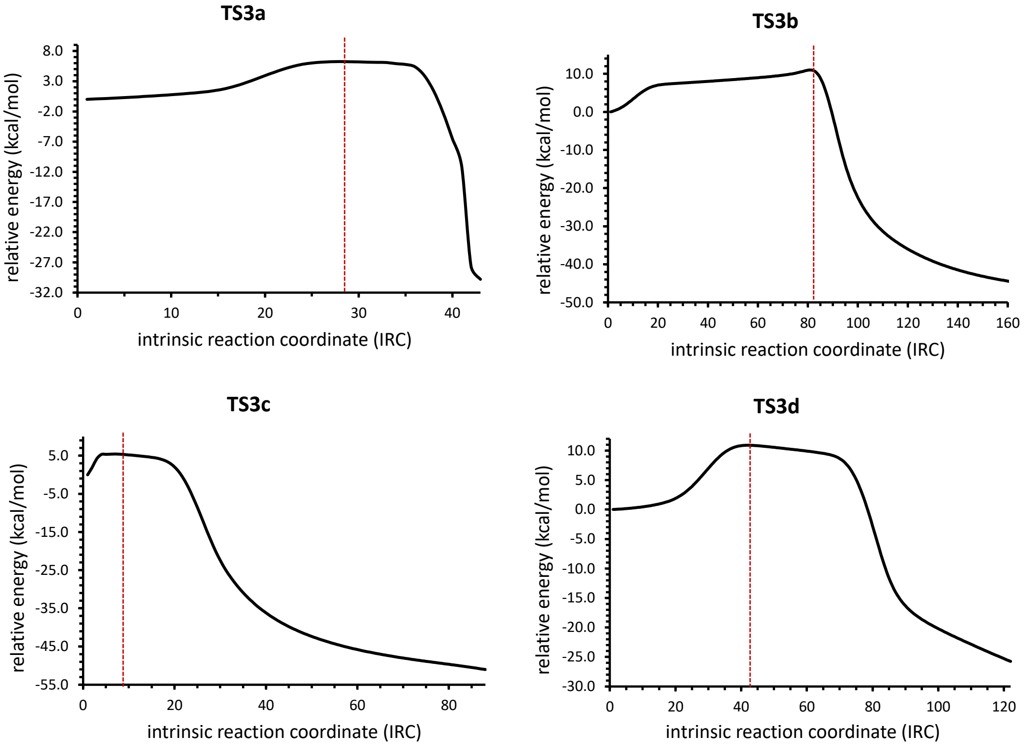

Figure S 68. IRCs (wb97xd/def2svp) corresponding to transition structures **TS3a**, **TS3b**, **TS3c** and **TS3d**.**TS3a** and **TS3c** were completed with relaxed scans after 10 points and before 8 points from the corresponding transition structure. The red dashed line indicates the transition state.

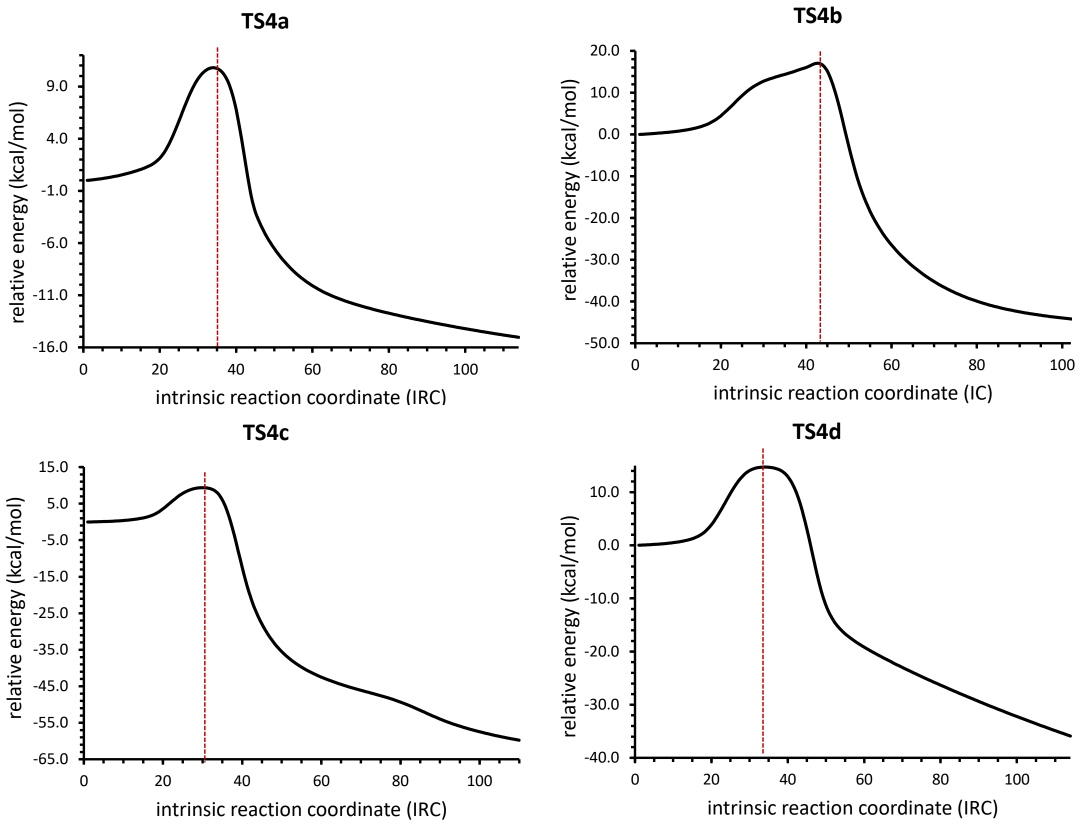

Figure S 69. IRCs (wb97xd/def2svp) corresponding to transition structures **TS4a**, **TS4b**, **TS4c** and **TS4d**. The red dashed line indicates the transition state.

Energies

Table S 14. Calculated (wb97xd/def2tzvp/pcm=toluene//wb97xd/def2svp) absolute (hartree) and relative (kcal/mol) energies for the transformation of **EC2** into **EP2** and **CY2** .

	E_0	ΔE_0	G	ΔG	im. freq
**EC2**	-4666.936063	0.0	-4667.033127	0.0	
**TS2a**	-4666.921472	9.2	-4667.016934	10.2	-102.3
**TS2b**	-4666.920881	9.5	-4667.018248	9.3	-283.1
**TS2c**	-4666.922829	8.3	-4667.016694	10.3	-100.1
**TS2d**	-4666.914449	13.6	-4667.011436	13.6	-75.2
(2*R*,4*S*)-**CY2**	-4667.035617	-62.5	-4667.136666	-65.0	
(2*S*,4*S*)-**CY2**	-4667.034869	-62.0	-4667.134881	-63.9	
(2*R*,3*r*,5*R*)-**EP2**	-4666.987781	-32.5	-4667.084815	-32.4	

Table S 15. Calculated (wb97xd/def2tzvp/pcm=toluene//wb97xd/def2svp) absolute (hartree) and relative (kcal/mol) energies for the transformation of **EC3** into **EP3** and **CY3** .

	E_0	ΔE_0	G	ΔG	im. freq
**EC3**	-4781.441079	0.0	-4781.540049	0.0	
**TS3a**	-4781.431976	5.7	-4781.532648	4.6	-83.9
**TS3b**	-4781.430028	6.9	-4781.532229	4.9	-242.3
**TS3c**	-4781.428921	7.6	-4781.526756	8.3	-87.1
**TS3d**	-4781.420430	13.0	-4781.521707	11.5	-105.4
(2*R*,4*S*)-**CY3**	-4781.538483	-61.1	-4781.642939	-64.6	
(2*S*,4*S*)-**CY3**	-4781.537674	-60.6	-4781.642778	-64.5	
(2*R*,3*r*,5*R*)-**EP3**	-4781.488421	-29.7	-4781.589374	-31.0	

Table S 16. Calculated (wb97xd/def2tzvp/pcm=toluene//wb97xd/def2svp) absolute (hartree) and relative (kcal/mol) energies for the transformation of **EC4** into **EP4** and **CY4** .

	E_0	ΔE_0	G	ΔG	im. freq
**EC4**	-5004.038543	0.0	-5004.142061	0.0	
**TS4a**	-5004.019872	11.7	-5004.122592	12.2	-169.8
**TS4b**	-5004.023355	9.5	-5004.125136	10.6	-248.5
**TS4c**	-5004.018319	12.7	-5004.122248	12.4	-120.7
**TS4d**	-5004.014970	14.8	-5004.119556	14.1	-95.3
(2*R*,4*S*)-**CY4**	-5004.137163	-61.9	-5004.241073	-62.1	
(2*S*,4*S*)-**CY4**	-5004.135087	-60.6	-5004.243853	-63.9	
(2*R*,3*r*,5*R*)-**EP4**	-5004.088621	-31.4	-5004.195912	-33.8	

Benchmarking

Three additional levels of theory were calculated confirming that the employed level is the closest to experimental results even though all the levels showed the same trend.

Level: m062x/cc-pvtz/pcm=toluene//m062x/cc-pvdz

Table S17. Calculated (m062x/cc-pvtz/pcm=toluene//m062x/cc-pvdz) absolute (hartree) and relative (kcal/mol) energies for the transformation of **EC2** into **EP2** and **CY2** .

	E_0	ΔE_0	G	ΔG	im. freq
**EC2**	-4666.784591	0.0	-4666.856119	0.0	
**TS2a**	-4666.764352	12.7	-4666.835733	12.8	-134.0
**TS2b**	-4666.761792	14.3	-4666.834845	13.3	-275.1
**TS2c**	-4666.766677	11.2	-4666.836856	12.1	-139.9
**TS2d**	-4666.758944	16.1	-4666.830760	15.9	-122.8
(2*R*,4*S*)-**CY2**	-4666.877483	-58.3	-4666.949556	-58.6	
(2*S*,4*S*)-**CY2**	-4666.876534	-57.7	-4666.952043	-60.2	
(2*R*,3*r*,5*R*)-**EP2**	-4666.834672	-31.4	-4666.907688	-32.4	

Table S18. Calculated (m062x/cc-pvtz/pcm=toluene//m062x/cc-pvdz) absolute (hartree) and relative (kcal/mol) energies for the transformation of **EC3** into **EP3** and **CY3** .

	E_0	ΔE_0	G	ΔG	im. freq
**EC3**	-4781.280628	0.0	-4781.353412	0.0	
**TS3a**	-4781.262342	11.5	-4781.336933	10.3	-80.4
**TS3b**	-4781.259134	13.5	-4781.334920	11.6	-201.3
**TS3c**	-4781.261625	11.9	-4781.334345	12.0	-97.4
**TS3d**	-4781.254139	16.6	-4781.328562	15.6	-110.2
(2*R*,4*S*)-**CY3**	-4781.369477	-55.8	-4781.444827	-57.4	
(2*S*,4*S*)-**CY3**	-4781.367670	-54.6	-4781.446764	-58.6	
(2*R*,3*r*,5*R*)-**EP3**	-4781.325350	-28.1	-4781.399192	-28.7	

Table S19. Calculated (m062x/cc-pvtz/pcm=toluene//m062x/cc-pvdz) absolute (hartree) and relative (kcal/mol) energies for the transformation of **EC4** into **EP4** and **CY4** .

	E_0	ΔE_0	G	ΔG	im. freq
**EC4**	-5003.865918	0.0	-5003.941723	0.0	
**TS4a**	-5003.841662	15.2	-5003.919115	14.2	-239.9
**TS4b**	-5003.839519	16.6	-5003.916449	15.9	-305.2
**TS4c**	-5003.845453	12.8	-5003.920524	13.3	-182.4
**TS4d**	-5003.837628	17.8	-5003.914686	17.0	-165.8
(2*R*,4*S*)-**CY4**	-5003.957344	-57.4	-5004.034098	-58.0	
(2*S*,4*S*)-**CY4**	-5003.954124	-55.4	-5004.034015	-57.9	
(2*R*,3*r*,5*R*)-**EP4**	-5003.913108	-29.6	-5003.991683	-31.3	

Level: b3lyp-gd3bj/6-311G(d,p)/pcm=toluene//b3lyp-gd3bj/6-31G(d,p)

Table S20. Calculated (b3lyp-gd3bj/6-311G(d,p)/pcm=toluene//b3lyp-gd3bj/6-31G(d,p)) absolute (hartree) and relative (kcal/mol) energies for the transformation of **EC2** into **EP2** and **CY2** .

	E_0	ΔE_0	G	ΔG	im. freq
**EC2**	-4667.321862	0.0	-4667.395918	0.0	
**TS2a**	-4667.314886	4.4	-4667.386725	5.8	-69.0
**TS2b**	-4667.304656	10.8	-4667.378087	11.2	-360.5
**TS2c**	-4667.314552	4.6	-4667.386559	5.9	-176.6
**TS2d**	-4667.304663	10.8	-4667.378478	10.9	-86.5
(2*R*,4*S*)-**CY2**	-4667.410997	-55.9	-4667.481286	-53.6	
(2*S*,4*S*)-**CY2**	-4667.410058	-55.3	-4667.483185	-54.8	
(2*R*,3*r*,5*R*)-**EP2**	-4667.362652	-25.6	-4667.433919	-23.8	

Table S21. Calculated (b3lyp-gd3bj/6-311G(d,p)/pcm=toluene//b3lyp-gd3bj/6-31G(d,p)) absolute (hartree) and relative (kcal/mol) energies for the transformation of **EC3** into **EP3** and **CY3** .

	E_0	ΔE_0	G	ΔG	im. freq
**EC3**	-4781.856839	0.0	-4781.932046	0.0	
**TS3a**	-4781.848255	5.4	-4781.923722	5.2	-61.5
**TS3b**	-4781.838406	11.6	-4781.914980	10.7	-361.4
**TS3c**	-4781.846994	6.2	-4781.922013	6.3	-145.7
**TS3d**	-4781.837596	12.1	-4781.913495	11.6	-32.0
(2*R*,4*S*)-**CY3**	-4781.939974	-52.2	-4782.017185	-53.4	
(2*S*,4*S*)-**CY3**	-4781.937686	-50.7	-4782.017172	-53.4	
(2*R*,3*r*,5*R*)-**EP3**	-4781.892203	-22.2	-4781.969625	-23.6	

Table S22. Calculated (b3lyp-gd3bj/6-311G(d,p)/pcm=toluene//b3lyp-gd3bj/6-31G(d,p)) absolute (hartree) and relative (kcal/mol) energies for the transformation of **EC4** into **EP4** and **CY4** .

	E_0	ΔE_0	G	ΔG	im. freq
**EC4**	-5004.466015	0.0	-5004.541690	0.0	
**TS4a**	-5004.455464	6.6	-5004.533307	5.3	-96.8
**TS4b**	-5004.446336	12.3	-5004.524423	10.8	-371.6
**TS4c**	-5004.456418	6.0	-5004.534177	4.7	-210.1
**TS4d**	-5004.446274	12.4	-5004.524916	10.5	-132.6
(2*R*,4*S*)-**CY4**	-5004.558047	-57.8	-5004.633597	-57.7	
(2*S*,4*S*)-**CY4**	-5004.551154	-53.4	-5004.636098	-59.2	
(2*R*,3*r*,5*R*)-**EP4**	-5004.504154	-23.9	-5004.581095	-24.7	

Level: m062x/6-311G(d,p)/pcm=toluene//m062x/6-31G(d,p)

Table S23. Calculated (b3lyp-gd3bj/6-311G(d,p)/pcm=toluene//b3lyp-gd3bj/6-31G(d,p)) absolute (hartree) and relative (kcal/mol) energies for the transformation of **EC2** into **EP2** and **CY2** .

	E_0	ΔE_0	G	ΔG	im. freq
**EC2**	-4666.497339	0.0	-4666.568052	0.0	
**TS2a**	-4666.477716	12.3	-4666.547818	12.7	-138.9
**TS2b**	-4666.472620	15.5	-4666.544921	14.5	-350.1
**TS2c**	-4666.481340	10.0	-4666.551009	10.7	-167.1
**TS2d**	-4666.471062	16.5	-4666.541926	16.4	-114.0
(2*R*,4*S*)-**CY2**	-4666.590419	-58.4	-4666.661658	-58.7	
(2*S*,4*S*)-**CY2**	-4666.591922	-59.4	-4666.668223	-62.9	
(2*R*,3*r*,5*R*)-**EP2**	-4666.550061	-33.1	-4666.621864	-33.8	

Table S24. Calculated (b3lyp-gd3bj/6-311G(d,p)/pcm=toluene//b3lyp-gd3bj/6-31G(d,p)) absolute (hartree) and relative (kcal/mol) energies for the transformation of **EC3** into **EP3** and **CY3** .

	E_0	ΔE_0	G	ΔG	im. freq
**EC3**	-4780.981389	0.0	-4781.053642	0.0	
**TS3a**	-4780.961835	12.3	-4781.035558	11.3	-36.2
**TS3b**	-4780.956007	15.9	-4781.031087	14.2	-294.6
**TS3c**	-4780.962852	11.6	-4781.035513	11.4	-109.5
**TS3d**	-4780.952437	18.2	-4781.025796	17.5	-90.9
(2*R*,4*S*)-**CY3**	-4781.069651	-55.4	-4781.147045	-58.6	
(2*S*,4*S*)-**CY3**	-4781.069510	-55.3	-4781.149609	-60.2	
(2*R*,3*r*,5*R*)-**EP3**	-4781.028720	-29.7	-4781.102358	-30.6	

Table S25. Calculated (b3lyp-gd3bj/6-311G(d,p)/pcm=toluene//b3lyp-gd3bj/6-31G(d,p)) absolute (hartree) and relative (kcal/mol) energies for the transformation of **EC4** into **EP4** and **CY4** .

	E_0	ΔE_0	G	ΔG	im. freq
**EC4**	-5003.539595	0.0	-5003.614225	0.0	
**TS4a**	-5003.516186	14.7	-5003.591986	14.0	-227.6
**TS4b**	-5003.511986	17.3	-5003.588616	16.1	-373.4
**TS4c**	-5003.521491	11.4	-5003.596650	11.0	-217.0
**TS4d**	-5003.511104	17.9	-5003.586946	17.1	-156.3
(2*R*,4*S*)-**CY4**	-5003.636309	-60.7	-5003.712570	-61.7	
(2*S*,4*S*)-**CY4**	-5003.630685	-57.2	-5003.711377	-61.0	
(2*R*,3*r*,5*R*)-**EP4**	-5003.589377	-31.2	-5003.666989	-33.1	

Energy profiles

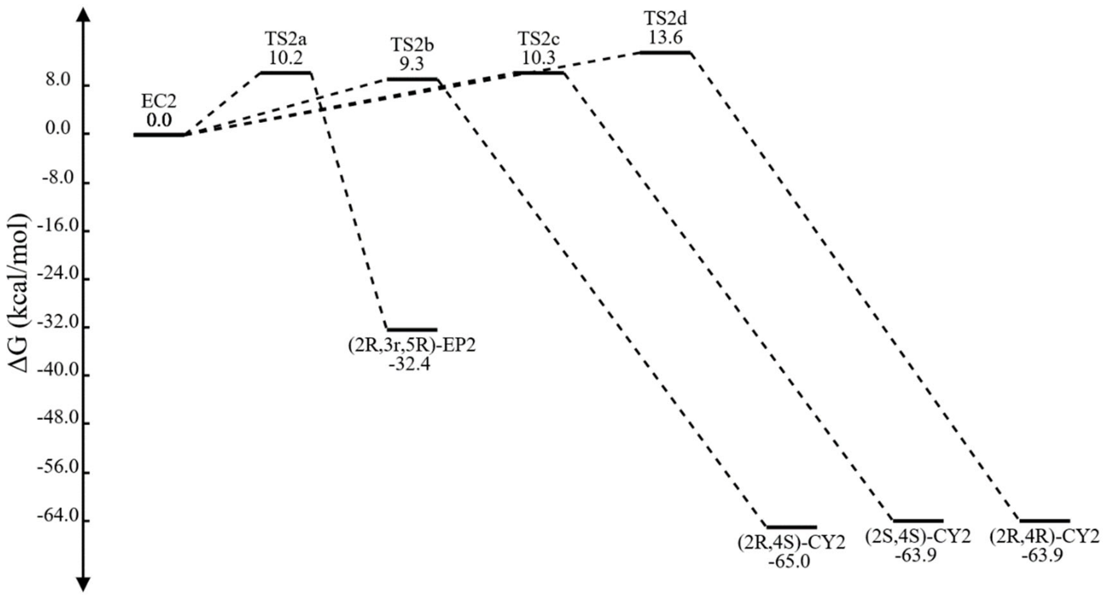

Figure S 70. Energy profiles (wb97xd/def2tzvp/pcm=toluene)//wb97xd/def2svp) for the different pathways from **EC2** to **EP2** and **CY2.** Relative free energies are given in kcal/mol.

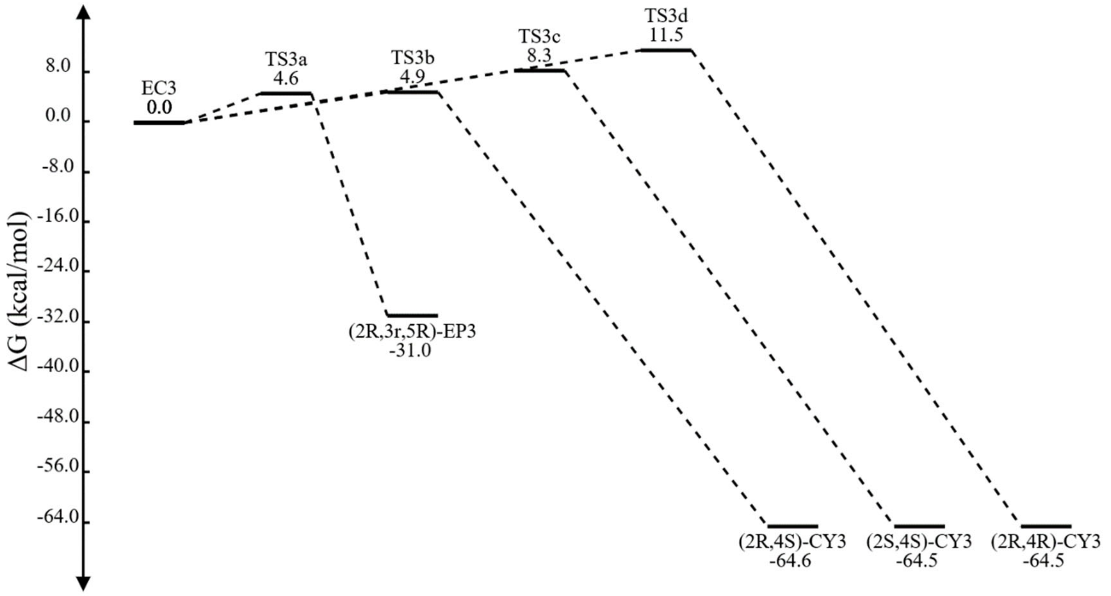

Figure S 71. Energy profiles (wb97xd/def2tzvp/pcm=toluene)//wb97xd/def2svp) for the different pathways from **EC3** to **EP3** and **CY3.** Relative free energies are given in kcal/mol.

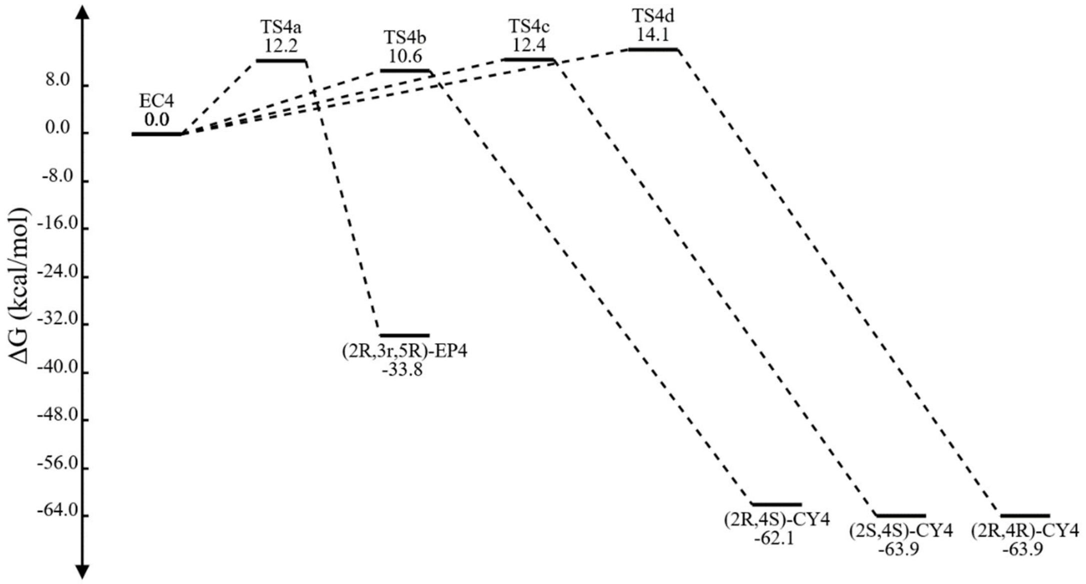

Figure S 72. Energy profiles (wb97xd/def2tzvp/pcm=toluene)//wb97xd/def2svp) for the different pathways from **EC4** to **EP4** and **CY4.** Relative free energies are given in kcal/mol.

A further exploration of the potential energy surface (PES) for the reaction of **EP3** (R = $MeOC_6H_4$) corresponding to the first part of the reaction, i.e. variation of the dihedral angle with respect to the IRC (Figure S 73, left), evidenced the planarity of the surface as it corresponds to the small difference between the two dihedral angles leading to epoxide and cyclopentanone.

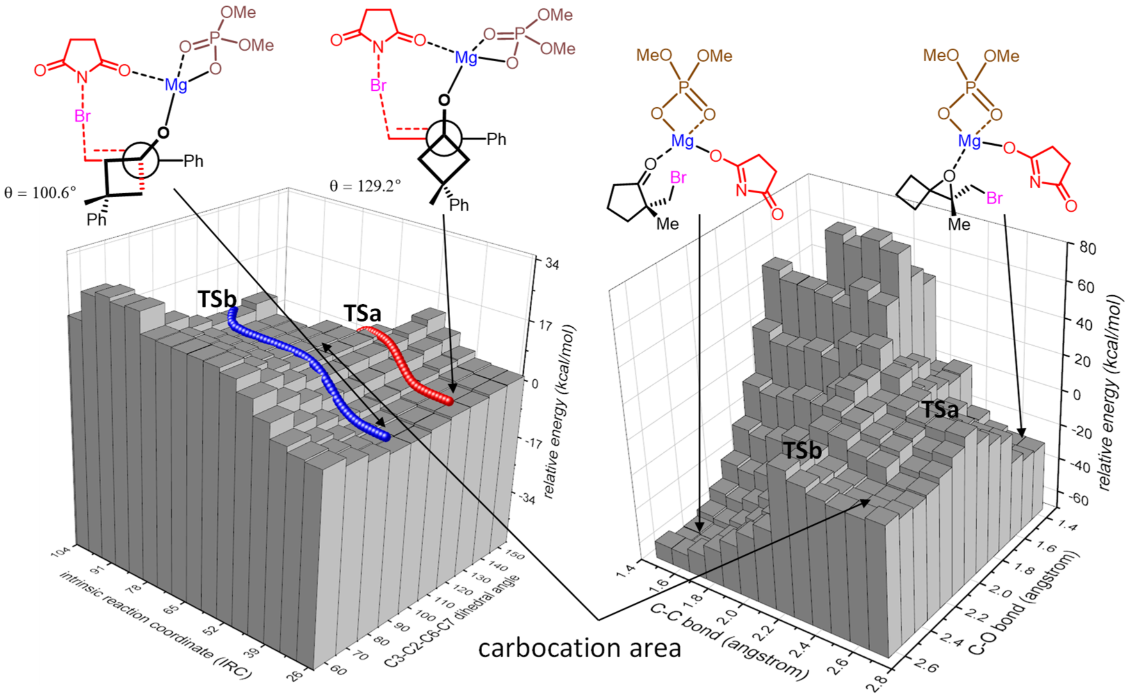

Figure S 73. Left: Potential energy surface (wb97xd/def2svp) related to the variation of the dihedral angle with respect to to the IRC (red: IRC for the formation of the epoxide; blue: IRC for the formation of the cyclopentanone). Right: Potential energy surface (wb97xd/def2svp) corresponding to the formation of O1-C1 and C2-C3 bonds leading to the epoxide and the cyclopentanone, respectively.

The exploration of the second part of the reaction, i.e. formation of either the O1-C1 bond (epoxide) or C2-C3 bond (cyclopentanone) (Figure S 73, right), was made in a similar way to the unsubstituted model; similar results were obtained confirming the absence of a direct connection between the epoxide and the cyclopentanone. For the PES exploration the most stable conformers were considered.

NCI Analysis

Non-covalent interactions are shown as isosurfaces in Figure S 74, Figure S 75 and Figure S 76. Thin, delocalized green surfaces indicates van der Waals interactions such as extended surfaces between aromatic rings illustrating π-π interactions. Small, lenticular, bluish surfaces indicate strong interactions such as hydrogen bonding. Steric clashes are shown as red isosurfaces. Forming bonds are indicated by a blue disc surrounded by a red area. s(**r**) against *sign*(λ_2)ρ(**r**) plots are also given in the bottom part. Quantitative analysis is given in Table S 26, Table S 27 and Table S 28. Blue area corresponds to strong NCI (H-bond, halogen, etc.); green area corresponds to van der Waals interactions and red area corresponds to repulsive interactions (steric clashes, etc.)

In all cases, the C-Br bond is essentially done at the transition structure but, interestingly, a strong interaction (disc blue) exists between the nitrogen and the bromine atoms indicating that the process, as a whole, has not finished, yet. This interaction demonstrates that, independently of the advance in the forming bonds like C-Br, all the process consist of a single kinetic step.

Transition structures **a** and **b**, place the aromatic ring planar with respect to the sp^2 carbon developing a positive charge. In those cases, in which the aromatic ring cannot be planar to the carbocationic sp^2 carbon for steric reasons (**c** and **d**), London interactions between the two aromatic rings are visible, compensating in part the destabilization caused by such a lack of planarity.

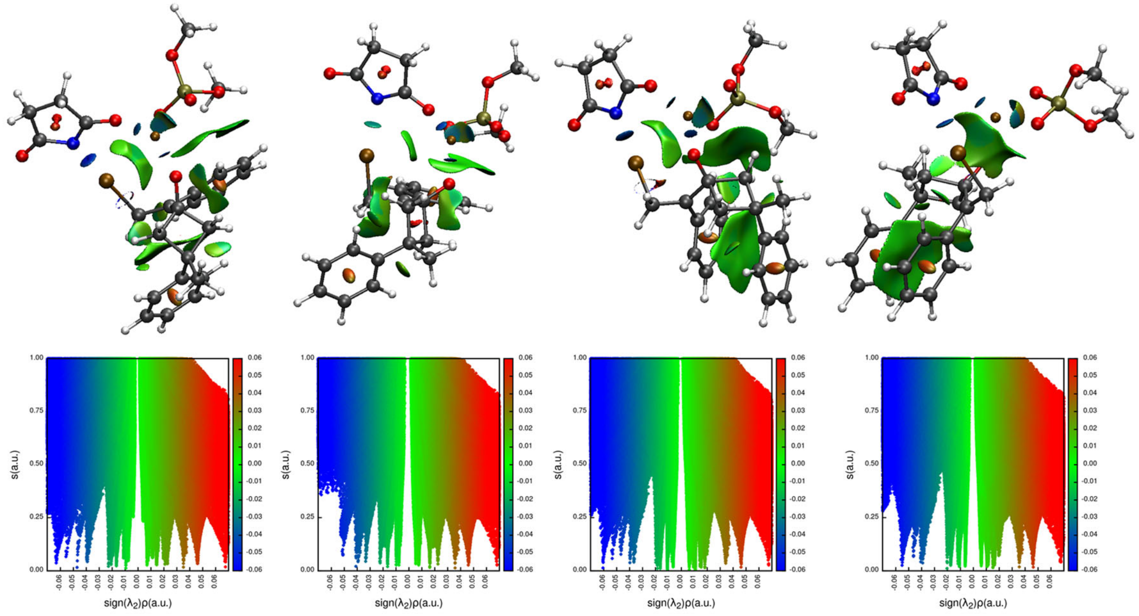

TS2a TS2b TS2c TS2d

Figure S 74. NCI analysis for transition structures **TS2a**, **TS2b**, **TS4c** and **TS2d**. Top: Isosurfaces. Bottom: s(**r**) against *sign*(λ_2)ρ(**r**) plots (see text).

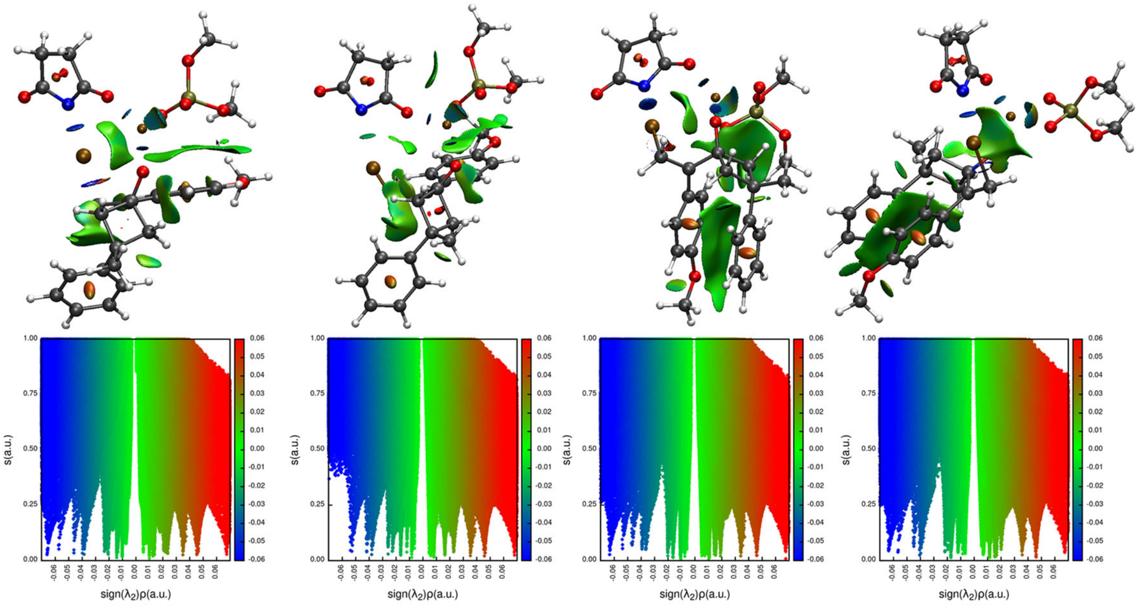

TS3a TS3b TS3c TS3d

Figure S 75. NCI analysis for transition structures **TS3a**, **TS3b**, **TS3c** and **TS3d**. Top: Isosurfaces. Bottom: s(**r**) against *sign*(λ_2)ρ(**r**) plots (see text).

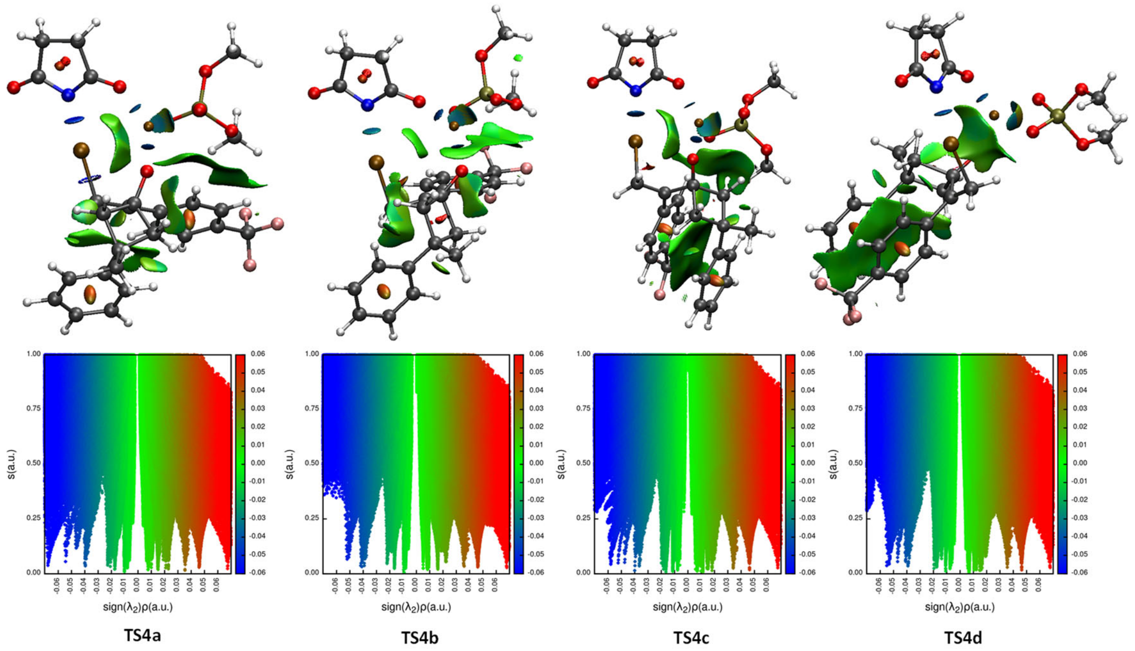

Figure S 76. NCI analysis for transition structures **TS4a**, **TS4b**, **TS4c** and **TS4d**. Top: Isosurfaces. Bottom: s(**r**) against $sign(\lambda_2)\rho(\mathbf{r})$ plots (see text).

Table S 26 Integration over the volumes of $sign(\lambda_2)\rho(\mathbf{n})$ for n=2.5 according to ref. 37 for **TS2a-d**

interval	TS2a	TS2b	TS2c	TS2d
**all**	-3.3160424	-3.30680306	-3.28056600	-3.28024501
**-1.0 to -0.02**	-0.23909918	-0.23536056	-0.23501657	-0.23027198
**-0.02 to 0.02**	0.00412829	0.00406551	0.00424492	0.00421830
**0.02 to 1.0**	0.30176314	0.30120803	0.30067717	0.29595285

[a] The interval [-0.1, -0.02] gives information about strong NCI (H-bond, halogen, etc.). The interval [-0.02, 0.02] gives information about van der Waals interactions. The interval [0.02, 1.00] gives information about steric clashes.

Table S 27 Integration over the volumes of $sign(\lambda_2)\rho(\mathbf{n})$ for n=2.5 according to ref. 37 for **TS3a-d**

interval	TS3a	TS3b	TS3c	TS3d
**all**	-3.45146392	-3.46129320	-3.43458121	-3.46034759
**-1.0 to -0.02**	-0.24680436	-0.24284827	-0.24965565	-0.24831046
**-0.02 to 0.02**	0.00424362	0.00410503	0.00446279	0.00433981
**0.02 to 1.0**	0.31623560	0.00410503	0.31787636	0.31617245

[a] The interval [-0.1, -0.02] gives information about strong NCI (H-bond, halogen, etc.). The interval [-0.02, 0.02] gives information about van der Waals interactions. The interval [0.02, 1.00] gives information about steric clashes.

Table S 28 Integration over the volumes of $sign(\lambda_2)\rho(\mathbf{n})$ for n=2.5 according to ref. 37 for **TS4a-d**

interval	TS4a	TS4b	TS4c	TS4d
**all**	-3.48364508	-3.48392618	-3.51629465	-3.48151987
**-1.0 to -0.02**	-0.23673273	-0.23556756	-0.23558497	-0.23850609
**-0.02 to 0.02**	0.00424149	0.00417054	0.00443372	0.00443898
**0.02 to 1.0**	0.31364802	0.31505788	0.31459717	0.31451545

[a] The interval [-0.1, -0.02] gives information about strong NCI (H-bond, halogen, etc.). The interval [-0.02, 0.02] gives information about van der Waals interactions. The interval [0.02, 1.00] gives information about steric clashes.

ELF Analysis

The topological analysis of the gradient field of ELF provides *basins* which are the domains in which the probability of finding an electron pair is maximal. The basins are classified as core and valence basins. The latter are characterised by the synaptic order, i.e. the number of atomic valence shells in which they participate.[20] Thus, there are monosynaptic, disynaptic, basins and so on. Monosynaptic basins, labelled V(A), correspond to lone pairs or non-bonding regions, while disynaptic basins, labelled V(A,B), connect the core of two nuclei A and B and, thus, correspond to a bonding region between A and B. This description recovers the Lewis bonding model, and the representation of the evolution of electron population of basins along the reaction coordinate provides a very intuitive graphical representation of how the molecular system evolves during the reaction. To verify the sequence of events during the course of the reaction we carried out a topological ELF analyses of the IRCs corresponding to **TS2a**, **TS2b**, **TS2c** and **TS2d**. For the purpose of comparison, we also carried out the analyses of **TS3b** and **TS4b**. Monosynaptic basins correspond to lone pairs of individual atoms. Disynaptic basins correspond to bonds. For the exact value of the basins (indicating the electron population) in each point of the IRC see the corresponding table.[21] Figures illustrate the evolution of the electronic population for the corresponding basins. Graphical representation of the attractors (maxima of electron density) allows a rapid identification of the moment in which a bond is broken or formed. Prior to a bond formation, monosynaptic basins appear in the involved atoms (or in one of them if there is a significant difference in electronegativity). When a bond is broken, monosynaptic basins are visible after breaking. The graphics illustrate how the electron density changes along the reaction resembling a classical description of the mechanism using curved arrows. The punctual changes can be immediately recognized thus identifying the events of the reaction and the order in which they take place.

---

[20] Savin, A. *J. Chem. Sci.* **2005**, *117*, 473-475

[21] Only the represntative basins that change during the reaction are collected

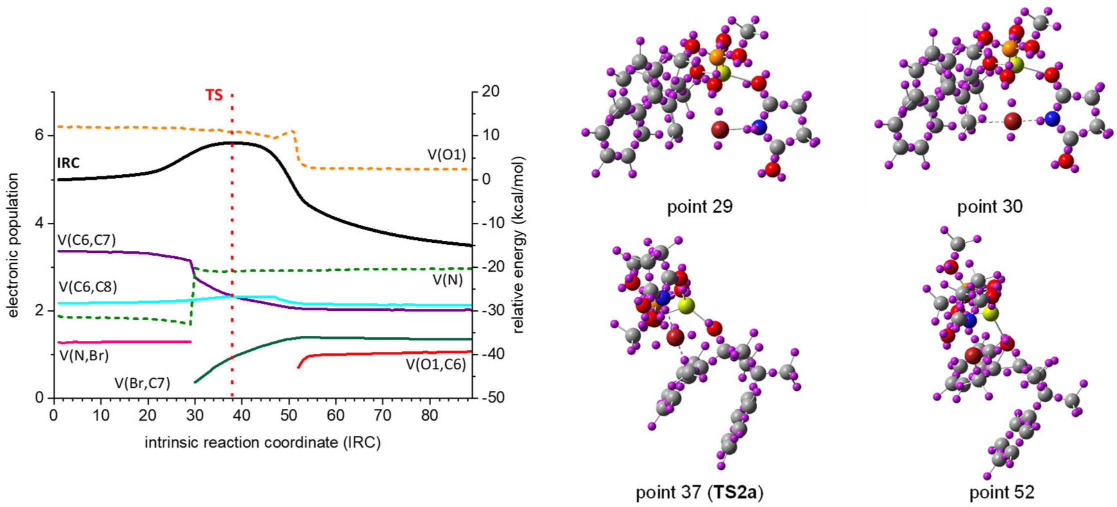

Figure S 77. Graphical representation of ELF analysis for the IRC corresponding to **TS2a**. Left: Evolution of the electron density along the IRC. Right. Position of attractors in critical and representative points.

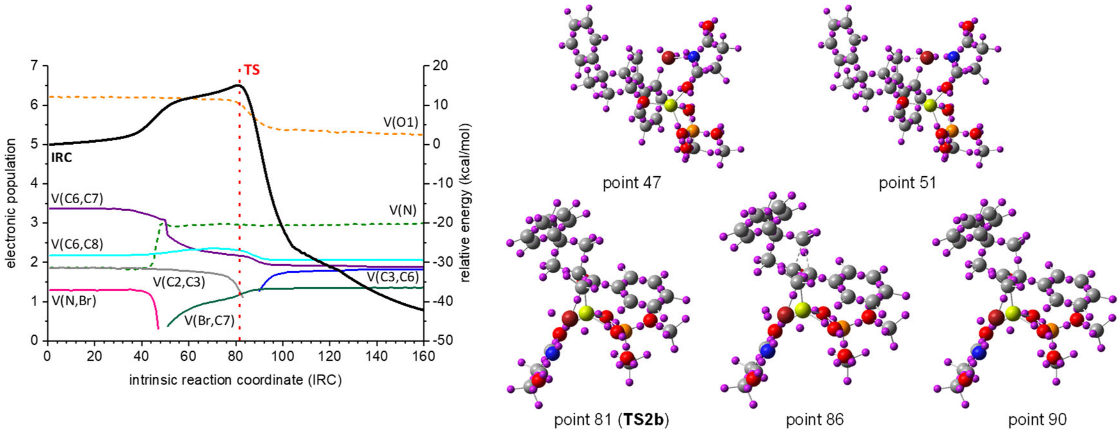

Figure S 78. Graphical representation of ELF analysis for the IRC corresponding to **TS2b**. Left: Evolution of the electron density along the IRC. Right. Position of attractors in critical and representative point

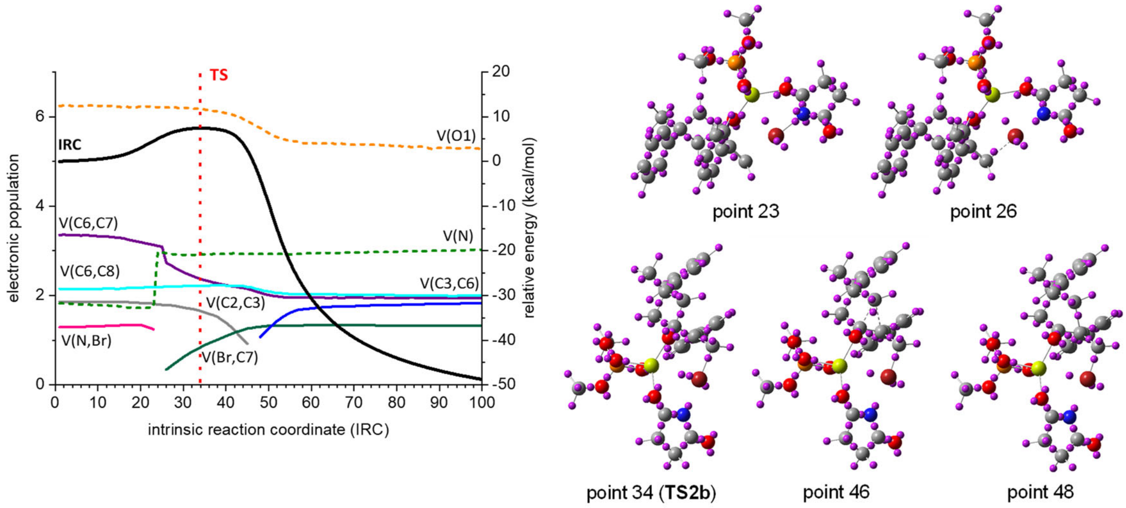

Figure S 79. Graphical representation of ELF analysis for the IRC corresponding to **TS2c**. Left: Evolution of the electron density along the IRC. Right. Position of attractors in critical and representative point

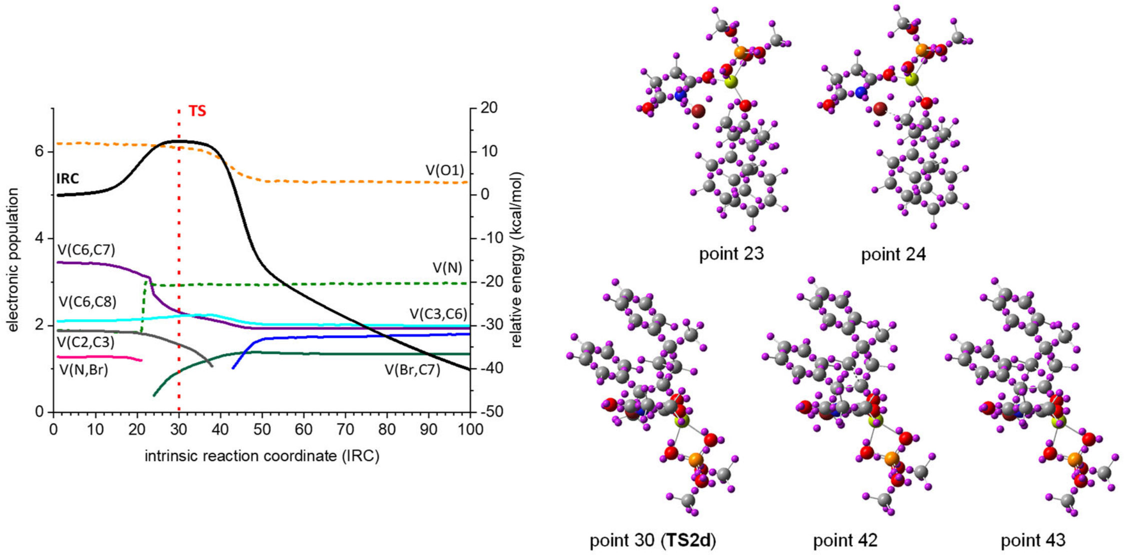

Figure S 80. Graphical representation of ELF analysis for the IRC corresponding to **TS2d**. Left: Evolution of the electron density along the IRC. Right. Position of attractors in critical and representative point

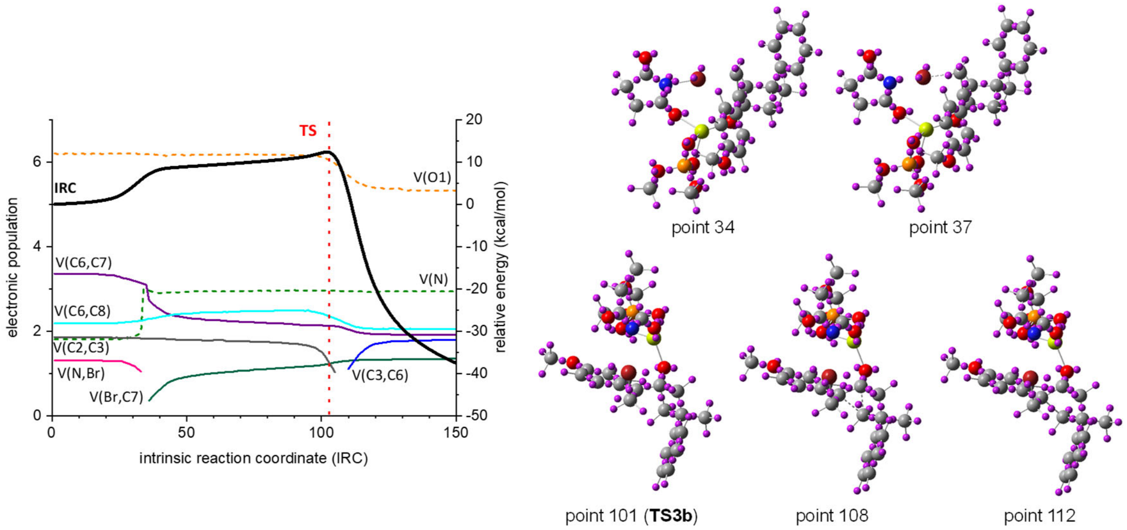

Figure S 81. Graphical representation of ELF analysis for the IRC corresponding to **TS3b**. Left: Evolution of the electron density along the IRC. Right. Position of attractors in critical and representative point

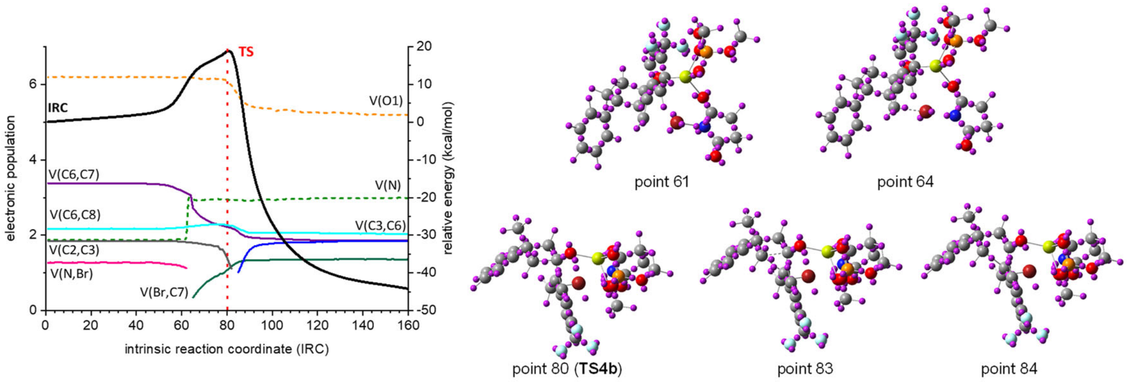

Figure S 82. Graphical representation of ELF analysis for the IRC corresponding to **TS4b**. Left: Evolution of the electron density along the IRC. Right. Position of attractors in critical and representative point

Table S 29. ELF basin populations for the IRC corresponding to **TS2a**.

	V(O1,C6)	V(C6,C8)	V(C7,Br)	V(O1)	V(C6,C7)	V(N,Br)	V(N)
1	-	2.18	-	6.21	3.37	1.28	1.88
2	-	2.18	-	6.21	3.37	1.27	1.86
3	-	2.18	-	6.20	3.37	1.28	1.86
4	-	2.18	-	6.21	3.36	1.29	1.85
5	-	2.18	-	6.21	3.36	1.29	1.86
6	-	2.18	-	6.20	3.36	1.28	1.85
7	-	2.18	-	6.20	3.36	1.28	1.85
8	-	2.19	-	6.20	3.35	1.28	1.85
9	-	2.19	-	6.20	3.35	1.28	1.84
10	-	2.19	-	6.20	3.35	1.29	1.85
11	-	2.19	-	6.19	3.35	1.29	1.85
12	-	2.19	-	6.19	3.34	1.29	1.84
13	-	2.19	-	6.19	3.35	1.29	1.84
14	-	2.19	-	6.20	3.35	1.29	1.84
15	-	2.19	-	6.19	3.34	1.29	1.84
16	-	2.19	-	6.21	3.34	1.29	1.83
17	-	2.19	-	6.20	3.34	1.29	1.82
18	-	2.20	-	6.19	3.33	1.30	1.82
19	-	2.20	-	6.20	3.32	1.30	1.82
20	-	2.20	-	6.19	3.32	1.30	1.82
21	-	2.21	-	6.19	3.30	1.30	1.81
22	-	2.21	-	6.18	3.29	1.30	1.80
23	-	2.22	-	6.18	3.27	1.30	1.80
24	-	2.22	-	6.18	3.25	1.30	1.78
25	-	2.22	-	6.19	3.24	1.30	1.77
26	-	2.23	-	6.18	3.22	1.30	1.76
27	-	2.24	-	6.18	3.2	1.30	1.74
28	-	2.24	-	6.17	3.17	1.30	1.72
29	-	2.25	-	6.16	3.15	1.30	1.69
30	-	2.26	0.37	6.15	2.77	-	2.98
31	-	2.27	0.45	6.14	2.69	-	2.95
32	-	2.28	0.53	6.14	2.63	-	2.93
33	-	2.29	0.61	6.13	2.57	-	2.91
34	-	2.30	0.70	6.14	2.51	-	2.91
35	-	2.31	0.77	6.14	2.46	-	2.90
36	-	2.32	0.83	6.12	2.41	-	2.90
37	-	2.32	0.89	6.09	2.38	-	2.91
38	-	2.33	0.94	6.11	2.34	-	2.91
39	-	2.33	0.99	6.10	2.31	-	2.90
40	-	2.33	1.03	6.08	2.28	-	2.92
41	-	2.33	1.07	6.09	2.26	-	2.92
42	-	2.33	1.11	6.06	2.24	-	2.92
43	-	2.33	1.15	6.05	2.22	-	2.92
44	-	2.32	1.19	6.02	2.20	-	2.92
45	-	2.32	1.22	6.00	2.18	-	2.92
46	-	2.32	1.25	5.99	2.16	-	2.93
47	-	2.32	1.28	5.95	2.14	-	2.92
48	-	2.27	1.31	6.00	2.12	-	2.92
49	-	2.24	1.34	6.04	2.10	-	2.92
50	-	2.21	1.36	6.09	2.08	-	2.92
51	-	2.19	1.37	6.12	2.07	-	2.92

52	0.71	2.18	1.39	5.47	2.06	-	2.92
53	0.88	2.17	1.40	5.34	2.05	-	2.93
54	0.96	2.16	1.40	5.29	2.05	-	2.92
55	0.98	2.16	1.40	5.27	2.04	-	2.92
56	0.99	2.16	1.40	5.27	2.04	-	2.93
57	1.00	2.16	1.39	5.26	2.04	-	2.93
58	1.00	2.16	1.39	5.26	2.04	-	2.93
59	1.01	2.16	1.39	5.25	2.03	-	2.94
60	1.01	2.15	1.39	5.26	2.03	-	2.94
61	1.01	2.15	1.39	5.26	2.03	-	2.94
62	1.01	2.15	1.38	5.27	2.03	-	2.94
63	1.02	2.15	1.38	5.26	2.03	-	2.95
64	1.02	2.15	1.38	5.26	2.03	-	2.95
65	1.02	2.15	1.38	5.26	2.03	-	2.95
66	1.02	2.15	1.38	5.25	2.03	-	2.95
67	1.02	2.15	1.37	5.25	2.03	-	2.96
68	1.03	2.15	1.37	5.26	2.03	-	2.96
69	1.03	2.14	1.37	5.25	2.03	-	2.96
70	1.03	2.14	1.37	5.25	2.03	-	2.96
71	1.03	2.14	1.37	5.26	2.03	-	2.96
72	1.04	2.15	1.37	5.25	2.02	-	2.96
73	1.04	2.14	1.37	5.24	2.03	-	2.96
74	1.04	2.14	1.37	5.24	2.03	-	2.96
75	1.05	2.15	1.37	5.24	2.02	-	2.96
76	1.05	2.15	1.36	5.24	2.02	-	2.96
77	1.05	2.14	1.36	5.24	2.02	-	2.96
78	1.05	2.14	1.36	5.24	2.02	-	2.96
79	1.06	2.14	1.36	5.24	2.02	-	2.96
80	1.05	2.14	1.36	5.24	2.02	-	2.97
81	1.05	2.14	1.36	5.24	2.02	-	2.96
82	1.06	2.14	1.36	5.24	2.02	-	2.96
83	1.06	2.14	1.36	5.24	2.02	-	2.96
84	1.06	2.14	1.35	5.24	2.02	-	2.97
85	1.06	2.13	1.36	5.24	2.02	-	2.97
86	1.06	2.13	1.36	5.24	2.02	-	2.97
87	1.06	2.14	1.36	5.24	2.02	-	2.97
88	1.07	2.14	1.35	5.24	2.03	-	2.96
89	1.07	2.14	1.35	5.24	2.02	-	2.97

Table S 30. ELF basin populations for the IRC corresponding to **TS2b**.

	**V(C2,C3)**	**V(C3,C6)**	**V(C6,C8)**	**V(C7,Br)**	**V(O1)**	**V(C6,C7)**	**V(N,Br)**	**VT(N)**
1	1.85	-	2.17	-	6.21	1.29	3.37	1.86
2	1.85	-	2.17	-	6.21	1.29	3.37	1.86
3	1.85	-	2.17	-	6.20	1.29	3.37	1.86
4	1.85	-	2.17	-	6.21	1.29	3.37	1.86
5	1.86	-	2.17	-	6.21	1.29	3.37	1.86
6	1.86	-	2.17	-	6.2	1.29	3.37	1.86
7	1.86	-	2.17	-	6.21	1.29	3.37	1.86
8	1.85	-	2.18	-	6.21	1.29	3.37	1.86
9	1.85	-	2.17	-	6.21	1.29	3.37	1.86
10	1.85	-	2.17	-	6.20	1.28	3.37	1.86
11	1.85	-	2.17	-	6.21	1.29	3.37	1.86

12	1.86	-	2.17	-	6.21	1.29	3.37	1.86
13	1.85	-	2.17	-	6.21	1.29	3.37	1.86
14	1.85	-	2.17	-	6.21	1.29	3.37	1.86
15	1.85	-	2.17	-	6.21	1.29	3.37	1.86
16	1.85	-	2.17	-	6.20	1.29	3.37	1.86
17	1.85	-	2.17	-	6.20	1.29	3.37	1.86
18	1.86	-	2.18	-	6.20	1.29	3.37	1.86
19	1.86	-	2.18	-	6.20	1.29	3.37	1.86
20	1.86	-	2.17	-	6.20	1.29	3.37	1.86
21	1.85	-	2.17	-	6.21	1.28	3.37	1.86
22	1.85	-	2.17	-	6.21	1.29	3.37	1.86
23	1.85	-	2.17	-	6.21	1.29	3.37	1.86
24	1.85	-	2.17	-	6.20	1.29	3.37	1.86
25	1.85	-	2.17	-	6.21	1.29	3.36	1.86
26	1.86	-	2.17	-	6.21	1.29	3.36	1.86
27	1.86	-	2.17	-	6.21	1.29	3.36	1.85
28	1.85	-	2.17	-	6.21	1.29	3.37	1.85
29	1.84	-	2.18	-	6.21	1.29	3.36	1.85
30	1.84	-	2.17	-	6.20	1.29	3.35	1.83
31	1.84	-	2.17	-	6.20	1.30	3.35	1.83
32	1.85	-	2.17	-	6.20	1.30	3.34	1.83
33	1.85	-	2.17	-	6.20	1.30	3.34	1.82
34	1.85	-	2.18	-	6.19	1.30	3.34	1.83
35	1.85	-	2.18	-	6.19	1.31	3.33	1.82
36	1.85	-	2.19	-	6.20	1.30	3.32	1.82
37	1.85	-	2.19	-	6.20	1.29	3.31	1.82
38	1.84	-	2.19	-	6.19	1.28	3.29	1.83
39	1.85	-	2.19	-	6.19	1.27	3.27	1.84
40	1.84	-	2.19	-	6.19	1.27	3.26	1.84
41	1.84	-	2.20	-	6.20	1.25	3.24	1.86
42	1.84	-	2.20	-	6.20	1.23	3.23	1.86
43	1.83	-	2.20	-	6.19	1.20	3.21	1.88
44	1.83	-	2.21	-	6.19	1.15	3.19	1.92
45	1.83	-	2.21	-	6.19	1.03	3.17	2.01
46	1.83	-	2.22	-	6.19	0.80	3.15	2.42
47	1.82	-	2.23	-	6.19	0.31	3.12	2.68
48	1.82	-	2.23	-	6.19	-	3.10	2.99
49	1.82	-	2.24	-	6.18	-	3.09	2.97
50	1.81	-	2.25	-	6.18	-	3.07	2.96
51	1.81	-	2.25	0.37	6.18	-	2.70	2.95
52	1.81	-	2.26	0.44	6.18	-	2.64	2.93
53	1.81	-	2.27	0.50	6.18	-	2.60	2.92
54	1.8	-	2.27	0.56	6.18	-	2.55	2.91
55	1.8	-	2.28	0.61	6.18	-	2.52	2.92
56	1.79	-	2.29	0.66	6.18	-	2.48	2.92
57	1.79	-	2.30	0.70	6.17	-	2.45	2.92
58	1.78	-	2.31	0.74	6.17	-	2.43	2.93
59	1.78	-	2.31	0.77	6.18	-	2.40	2.94
60	1.78	-	2.32	0.80	6.17	-	2.38	2.94
61	1.77	-	2.32	0.83	6.17	-	2.36	2.95
62	1.77	-	2.32	0.86	6.16	-	2.34	2.95
63	1.76	-	2.33	0.88	6.17	-	2.33	2.95
64	1.76	-	2.33	0.9	6.15	-	2.31	2.95
65	1.75	-	2.33	0.92	6.15	-	2.30	2.95
66	1.75	-	2.34	0.94	6.14	-	2.29	2.95
67	1.74	-	2.34	0.95	6.15	-	2.28	2.95
68	1.74	-	2.35	0.97	6.15	-	2.27	2.96
69	1.73	-	2.35	0.98	6.15	-	2.26	2.96

70	1.72	-	2.35	0.99	6.15	-	2.25	2.96
71	1.71	-	2.35	1.00	6.16	-	2.25	2.97
72	1.70	-	2.35	1.02	6.15	-	2.24	2.97
73	1.69	-	2.35	1.03	6.15	-	2.23	2.97
74	1.68	-	2.34	1.04	6.14	-	2.22	2.97
75	1.66	-	2.35	1.05	6.14	-	2.21	2.97
76	1.64	-	2.34	1.07	6.14	-	2.20	2.97
77	1.62	-	2.34	1.08	6.13	-	2.20	2.97
78	1.56	-	2.33	1.10	6.12	-	2.19	2.97
79	1.52	-	2.33	1.11	6.12	-	2.19	2.96
80	1.44	-	2.31	1.14	6.09	-	2.18	2.96
81	1.33	-	2.31	1.16	6.05	-	2.17	2.96
82	1.23	-	2.30	1.19	6.01	-	2.16	2.95
83	1.11	-	2.29	1.21	5.97	-	2.16	2.96
84	-	-	2.27	1.23	5.91	-	2.15	2.95
85	-	-	2.25	1.25	5.85	-	2.14	2.95
86	-	-	2.22	1.27	5.78	-	2.12	2.95
87	-	-	2.19	1.28	5.73	-	2.09	2.94
88	-	-	2.17	1.30	5.68	-	2.06	2.94
89	-	-	2.15	1.30	5.62	-	2.03	2.94
90	-	1.27	2.13	1.31	5.58	-	2.00	2.94
91	-	1.36	2.10	1.31	5.53	-	1.99	2.93
92	-	1.44	2.09	1.32	5.49	-	1.97	2.93
93	-	1.51	2.08	1.32	5.47	-	1.96	2.94
94	-	1.57	2.07	1.32	5.44	-	1.95	2.94
95	-	1.62	2.06	1.32	5.43	-	1.94	2.94
96	-	1.66	2.06	1.32	5.41	-	1.94	2.94
97	-	1.68	2.06	1.32	5.40	-	1.93	2.94
98	-	1.70	2.06	1.32	5.39	-	1.93	2.94
99	-	1.71	2.06	1.32	5.38	-	1.93	2.94
100	-	1.72	2.06	1.32	5.37	-	1.93	2.94
101	-	1.74	2.06	1.33	5.36	-	1.93	2.94
102	-	1.75	2.06	1.33	5.37	-	1.93	2.95
103	-	1.76	2.06	1.33	5.36	-	1.93	2.94
104	-	1.76	2.06	1.33	5.38	-	1.92	2.95
105	-	1.76	2.06	1.34	5.37	-	1.92	2.94
106	-	1.76	2.06	1.34	5.38	-	1.92	2.94
107	-	1.76	2.06	1.34	5.36	-	1.92	2.95
108	-	1.77	2.06	1.34	5.37	-	1.92	2.94
109	-	1.77	2.06	1.34	5.37	-	1.92	2.94
110	-	1.77	2.07	1.34	5.37	-	1.92	2.94
111	-	1.77	2.06	1.34	5.37	-	1.92	2.94
112	-	1.77	2.07	1.34	5.35	-	1.92	2.94
113	-	1.78	2.07	1.34	5.35	-	1.92	2.94
114	-	1.78	2.07	1.34	5.35	-	1.92	2.94
115	-	1.78	2.06	1.34	5.34	-	1.92	2.94
116	-	1.78	2.07	1.34	5.35	-	1.92	2.94
117	-	1.78	2.07	1.34	5.35	-	1.92	2.95
118	-	1.78	2.07	1.34	5.35	-	1.92	2.95
119	-	1.78	2.06	1.34	5.35	-	1.92	2.95
120	-	1.79	2.06	1.34	5.31	-	1.91	2.95
121	-	1.79	2.06	1.34	5.32	-	1.91	2.95
122	-	1.79	2.06	1.35	5.31	-	1.91	2.96
123	-	1.79	2.06	1.35	5.31	-	1.91	2.95
124	-	1.79	2.06	1.35	5.3	-	1.91	2.95
125	-	1.79	2.06	1.35	5.29	-	1.91	2.95
126	-	1.79	2.06	1.35	5.33	-	1.91	2.96
127	-	1.80	2.06	1.35	5.33	-	1.91	2.96

128	-	1.80	2.06	1.35	5.32	-	1.90	2.96
129	-	1.80	2.06	1.35	5.32	-	1.90	2.96
130	-	1.80	2.06	1.35	5.31	-	1.90	2.97
131	-	1.80	2.06	1.35	5.31	-	1.90	2.97
132	-	1.81	2.06	1.35	5.32	-	1.90	2.97
133	-	1.81	2.06	1.35	5.31	-	1.90	2.97
134	-	1.81	2.06	1.35	5.31	-	1.90	2.96
135	-	1.81	2.06	1.35	5.30	-	1.90	2.96
136	-	1.81	2.06	1.35	5.30	-	1.89	2.96
137	-	1.81	2.06	1.35	5.29	-	1.89	2.97
138	-	1.81	2.06	1.35	5.30	-	1.89	2.97
139	-	1.81	2.06	1.36	5.30	-	1.89	2.97
140	-	1.81	2.06	1.35	5.28	-	1.89	2.97
141	-	1.81	2.06	1.35	5.29	-	1.89	2.97
142	-	1.81	2.06	1.35	5.28	-	1.89	2.97
143	-	1.81	2.06	1.35	5.27	-	1.89	2.97
144	-	1.81	2.06	1.35	5.27	-	1.89	2.97
145	-	1.81	2.06	1.35	5.27	-	1.89	2.97
146	-	1.81	2.06	1.36	5.27	-	1.88	2.97
147	-	1.82	2.06	1.35	5.27	-	1.88	2.96
148	-	1.82	2.06	1.35	5.27	-	1.88	2.97
149	-	1.82	2.06	1.35	5.28	-	1.88	2.97
150	-	1.82	2.06	1.35	5.27	-	1.88	2.97
151	-	1.82	2.06	1.35	5.28	-	1.88	2.98
152	-	1.82	2.06	1.36	5.27	-	1.88	2.98
153	-	1.82	2.06	1.35	5.27	-	1.88	2.98
154	-	1.82	2.06	1.35	5.26	-	1.88	2.98
155	-	1.82	2.06	1.35	5.26	-	1.88	2.98
156	-	1.82	2.06	1.36	5.25	-	1.88	2.98
157	-	1.82	2.06	1.35	5.25	-	1.88	2.98
158	-	1.82	2.06	1.35	5.25	-	1.88	2.98
159	-	1.82	2.06	1.35	5.25	-	1.88	2.99
160	-	1.82	2.05	1.36	5.24	-	1.88	2.99

Table S 31. ELF basin populations for the IRC corresponding to **TS2c**.

	**V(C2,C3)**	**V(C3,C6)**	**V(C6,C8)**	**V(C7,Br)**	**V(O1)**	**V(C6,C7)**	**V(N,Br)**	**V(N)**
1	1.86	-	2.15	-	6.24	3.35	1.29	1.82
2	1.86	-	2.15	-	6.25	3.36	1.30	1.82
3	1.86	-	2.15	-	6.25	3.35	1.30	1.82
4	1.86	-	2.15	-	6.24	3.35	1.30	1.81
5	1.86	-	2.15	-	6.24	3.35	1.30	1.80
6	1.86	-	2.15	-	6.23	3.34	1.30	1.80
7	1.86	-	2.15	-	6.24	3.35	1.30	1.80
8	1.86	-	2.15	-	6.25	3.34	1.31	1.79
9	1.86	-	2.15	-	6.25	3.34	1.31	1.79
10	1.85	-	2.15	-	6.24	3.32	1.31	1.79
11	1.86	-	2.15	-	6.24	3.32	1.31	1.78
12	1.85	-	2.16	-	6.24	3.32	1.32	1.78
13	1.85	-	2.16	-	6.24	3.31	1.32	1.77
14	1.85	-	2.15	-	6.23	3.3	1.33	1.77

15	1.85	-	2.15	-	6.22	3.29	1.33	1.76
16	1.85	-	2.16	-	6.21	3.28	1.34	1.74
17	1.85	-	2.17	-	6.22	3.25	1.34	1.73
18	1.84	-	2.17	-	6.21	3.23	1.34	1.73
19	1.84	-	2.17	-	6.21	3.21	1.34	1.72
20	1.83	-	2.17	-	6.23	3.19	1.34	1.72
21	1.83	-	2.18	-	6.23	3.17	1.31	1.72
22	1.83	-	2.18	-	6.23	3.15	1.28	1.73
23	1.81	-	2.18	-	6.22	3.13	1.24	1.75
24	1.80	-	2.19	-	6.21	3.11	-	2.98
25	1.79	-	2.19	-	6.21	3.09	-	2.96
26	1.79	-	2.20	0.34	6.21	2.73	-	2.95
27	1.78	-	2.20	0.41	6.20	2.68	-	2.93
28	1.77	-	2.20	0.48	6.21	2.62	-	2.92
29	1.75	-	2.20	0.55	6.19	2.57	-	2.91
30	1.73	-	2.21	0.62	6.2	2.52	-	2.91
31	1.71	-	2.21	0.68	6.19	2.48	-	2.91
32	1.70	-	2.22	0.74	6.17	2.44	-	2.91
33	1.68	-	2.22	0.79	6.18	2.4	-	2.91
34	1.65	-	2.22	0.84	6.17	2.37	-	2.93
35	1.62	-	2.22	0.89	6.16	2.34	-	2.92
36	1.59	-	2.22	0.93	6.15	2.32	-	2.92
37	1.56	-	2.23	0.97	6.13	2.29	-	2.93
38	1.52	-	2.23	1.01	6.12	2.27	-	2.93
39	1.45	-	2.23	1.05	6.10	2.25	-	2.93
40	1.39	-	2.22	1.09	6.08	2.23	-	2.93
41	1.31	-	2.22	1.13	6.06	2.21	-	2.94
42	1.22	-	2.22	1.17	6.03	2.2	-	2.94
43	1.11	-	2.21	1.20	6.00	2.18	-	2.93
44	1.01	-	2.21	1.23	5.95	2.17	-	2.93
45	0.92	-	2.19	1.26	5.90	2.15	-	2.93
46	-	-	2.18	1.27	5.85	2.12	-	2.93
47	-	-	2.15	1.29	5.79	2.09	-	2.93
48	-	1.07	2.13	1.3	5.75	2.06	-	2.93
49	-	1.16	2.10	1.31	5.69	2.03	-	2.93
50	-	1.26	2.09	1.32	5.63	2.01	-	2.93
51	-	1.35	2.07	1.33	5.58	1.99	-	2.93
52	-	1.43	2.05	1.33	5.54	1.98	-	2.93
53	-	1.50	2.05	1.33	5.51	1.97	-	2.93
54	-	1.56	2.04	1.33	5.48	1.95	-	2.92
55	-	1.61	2.03	1.33	5.46	1.95	-	2.92
56	-	1.65	2.03	1.33	5.45	1.95	-	2.92
57	-	1.68	2.03	1.33	5.44	1.95	-	2.93
58	-	1.69	2.02	1.33	5.41	1.95	-	2.93
59	-	1.70	2.02	1.34	5.40	1.95	-	2.94
60	-	1.71	2.02	1.34	5.41	1.95	-	2.94
61	-	1.72	2.02	1.34	5.40	1.95	-	2.94
62	-	1.73	2.02	1.34	5.40	1.95	-	2.95
63	-	1.74	2.02	1.34	5.40	1.95	-	2.95
64	-	1.74	2.02	1.34	5.40	1.94	-	2.95
65	-	1.75	2.02	1.34	5.40	1.95	-	2.95
66	-	1.75	2.02	1.34	5.39	1.95	-	2.95
67	-	1.76	2.01	1.34	5.40	1.94	-	2.95
68	-	1.76	2.01	1.34	5.38	1.95	-	2.96
69	-	1.76	2.01	1.34	5.38	1.95	-	2.96
70	-	1.76	2.01	1.34	5.37	1.95	-	2.96
71	-	1.77	2.01	1.34	5.38	1.95	-	2.96
72	-	1.77	2.01	1.34	5.38	1.95	-	2.96

73	-	1.77	2.01	1.34	5.38	1.94	-	2.97
74	-	1.78	2.01	1.34	5.37	1.94	-	2.97
75	-	1.78	2.01	1.33	5.37	1.95	-	2.97
76	-	1.78	2.01	1.33	5.37	1.95	-	2.97
77	-	1.79	2.01	1.33	5.36	1.95	-	2.97
78	-	1.79	2.01	1.33	5.36	1.94	-	2.97
79	-	1.79	2.01	1.33	5.35	1.94	-	2.97
80	-	1.79	2.00	1.33	5.35	1.94	-	2.98
81	-	1.79	2.00	1.33	5.34	1.94	-	2.98
82	-	1.80	2.00	1.33	5.33	1.94	-	2.99
83	-	1.80	2.00	1.33	5.33	1.95	-	2.99
84	-	1.80	2.00	1.33	5.31	1.95	-	2.99
85	-	1.80	2.00	1.33	5.33	1.94	-	3.00
86	-	1.80	2.00	1.33	5.33	1.94	-	2.99
87	-	1.81	2.00	1.33	5.31	1.94	-	2.99
88	-	1.81	2.00	1.33	5.28	1.94	-	3.00
89	-	1.81	2.00	1.33	5.29	1.94	-	3.00
90	-	1.81	2.00	1.33	5.31	1.94	-	3.00
91	-	1.81	2.00	1.32	5.31	1.94	-	3.00
92	-	1.81	1.99	1.32	5.30	1.94	-	3.00
93	-	1.81	1.99	1.32	5.30	1.94	-	3.00
94	-	1.81	1.99	1.32	5.29	1.94	-	3.01
95	-	1.82	2.00	1.33	5.29	1.94	-	3.01
96	-	1.82	2.00	1.32	5.30	1.94	-	3.01
97	-	1.82	2.00	1.32	5.31	1.94	-	3.02
98	-	1.82	2.00	1.32	5.30	1.94	-	3.02
99	-	1.82	2.00	1.33	5.28	1.94	-	3.03
100	-	1.82	2.00	1.32	5.29	1.94	-	3.02

Table S 32. ELF basin populations for the IRC corresponding to **TS2d**.

	**V(C2,C3)**	**V(C3,C6)**	**V(C6,C8)**	**V(C7,Br)**	**V(O1)**	**V(C6,C7)**	**V(N,Br)**	**V(N)**
1	1.88	-	2.10	-	6.19	3.45	1.28	1.89
2	1.88	-	2.10	-	6.19	3.45	1.27	1.89
3	1.88	-	2.11	-	6.18	3.44	1.27	1.89
4	1.88	-	2.11	-	6.18	3.44	1.27	1.88
5	1.87	-	2.11	-	6.19	3.44	1.27	1.88
6	1.87	-	2.11	-	6.2	3.44	1.27	1.88
7	1.87	-	2.11	-	6.2	3.43	1.27	1.89
8	1.87	-	2.11	-	6.2	3.43	1.28	1.88
9	1.87	-	2.11	-	6.18	3.44	1.28	1.87
10	1.87	-	2.11	-	6.18	3.42	1.28	1.88
11	1.87	-	2.11	-	6.19	3.42	1.28	1.87
12	1.87	-	2.11	-	6.17	3.41	1.28	1.87
13	1.86	-	2.11	-	6.16	3.4	1.28	1.86
14	1.86	-	2.11	-	6.18	3.38	1.28	1.86
15	1.86	-	2.12	-	6.18	3.36	1.27	1.85
16	1.85	-	2.12	-	6.17	3.34	1.27	1.85
17	1.85	-	2.13	-	6.16	3.31	1.27	1.85
18	1.84	-	2.13	-	6.17	3.28	1.26	1.85
19	1.84	-	2.14	-	6.16	3.24	1.24	1.85
20	1.83	-	2.14	-	6.16	3.21	1.21	1.85
21	1.81	-	2.15	-	6.16	3.16	1.20	1.85

22	1.80	-	2.15	-	6.16	3.13	-	3.01
23	1.78	-	2.16	-	6.15	3.10	-	2.97
24	1.76	-	2.17	0.39	6.14	2.71	-	2.94
25	1.73	-	2.18	0.51	6.14	2.61	-	2.92
26	1.70	-	2.18	0.63	6.13	2.52	-	2.92
27	1.67	-	2.19	0.73	6.12	2.45	-	2.92
28	1.63	-	2.20	0.82	6.11	2.39	-	2.92
29	1.60	-	2.21	0.88	6.10	2.35	-	2.93
30	1.56	-	2.22	0.93	6.10	2.31	-	2.93
31	1.53	-	2.23	0.99	6.09	2.28	-	2.93
32	1.49	-	2.23	1.03	6.08	2.25	-	2.93
33	1.45	-	2.23	1.07	6.07	2.23	-	2.94
34	1.40	-	2.24	1.11	6.05	2.20	-	2.94
35	1.33	-	2.24	1.14	6.04	2.18	-	2.95
36	1.26	-	2.23	1.17	6.02	2.17	-	2.94
37	1.17	-	2.24	1.20	5.98	2.15	-	2.95
38	1.06	-	2.24	1.23	5.94	2.13	-	2.95
39	-	-	2.23	1.26	5.89	2.11	-	2.94
40	-	-	2.2	1.29	5.84	2.09	-	2.94
41	-	-	2.18	1.32	5.75	2.07	-	2.94
42	-	-	2.16	1.33	5.68	2.04	-	2.94
43	-	1.02	2.14	1.35	5.62	2.01	-	2.94
44	-	1.15	2.11	1.36	5.54	1.99	-	2.94
45	-	1.28	2.09	1.37	5.47	1.97	-	2.94
46	-	1.41	2.07	1.38	5.44	1.95	-	2.94
47	-	1.51	2.06	1.38	5.40	1.95	-	2.94
48	-	1.60	2.05	1.39	5.39	1.93	-	2.94
49	-	1.64	2.04	1.39	5.36	1.94	-	2.93
50	-	1.68	2.03	1.38	5.34	1.94	-	2.94
51	-	1.70	2.03	1.38	5.32	1.94	-	2.94
52	-	1.70	2.02	1.37	5.31	1.94	-	2.94
53	-	1.71	2.02	1.37	5.32	1.94	-	2.94
54	-	1.71	2.02	1.37	5.33	1.94	-	2.94
55	-	1.72	2.02	1.36	5.33	1.94	-	2.94
56	-	1.72	2.02	1.36	5.32	1.94	-	2.94
57	-	1.73	2.02	1.36	5.32	1.94	-	2.95
58	-	1.72	2.02	1.36	5.32	1.94	-	2.95
59	-	1.73	2.02	1.36	5.31	1.94	-	2.95
60	-	1.73	2.02	1.35	5.31	1.94	-	2.95
61	-	1.74	2.02	1.35	5.3	1.94	-	2.95
62	-	1.74	2.02	1.36	5.29	1.94	-	2.96
63	-	1.74	2.02	1.36	5.31	1.94	-	2.95
64	-	1.73	2.01	1.35	5.31	1.94	-	2.95
65	-	1.74	2.02	1.35	5.31	1.94	-	2.96
66	-	1.74	2.02	1.35	5.31	1.94	-	2.96
67	-	1.74	2.02	1.35	5.30	1.94	-	2.97
68	-	1.74	2.01	1.35	5.30	1.94	-	2.96
69	-	1.75	2.02	1.35	5.32	1.94	-	2.96
70	-	1.75	2.02	1.35	5.31	1.94	-	2.97
71	-	1.75	2.02	1.35	5.31	1.94	-	2.96
72	-	1.75	2.01	1.35	5.31	1.94	-	2.96
73	-	1.75	2.01	1.35	5.31	1.94	-	2.95
74	-	1.75	2.01	1.35	5.31	1.94	-	2.96
75	-	1.75	2.01	1.35	5.31	1.94	-	2.96
76	-	1.76	2.01	1.35	5.32	1.94	-	2.96
77	-	1.76	2.01	1.35	5.30	1.94	-	2.97
78	-	1.76	2.01	1.35	5.30	1.94	-	2.97
79	-	1.76	2.01	1.35	5.31	1.94	-	2.97

80	-	1.76	2.01	1.35	5.31	1.94	-	2.97
81	-	1.77	2.01	1.35	5.31	1.94	-	2.97
82	-	1.77	2.01	1.35	5.30	1.94	-	2.97
83	-	1.77	2.01	1.35	5.31	1.94	-	2.97
84	-	1.77	2.00	1.35	5.30	1.94	-	2.97
85	-	1.77	2.00	1.35	5.30	1.94	-	2.97
86	-	1.78	2.00	1.35	5.30	1.94	-	2.97
87	-	1.78	2.00	1.35	5.31	1.94	-	2.97
88	-	1.78	2.00	1.35	5.30	1.94	-	2.97
89	-	1.78	2.00	1.35	5.30	1.94	-	2.97
90	-	1.78	2.00	1.35	5.30	1.94	-	2.97
91	-	1.79	2.00	1.34	5.30	1.94	-	2.97
92	-	1.79	2.00	1.34	5.29	1.94	-	2.97
93	-	1.79	2.00	1.34	5.30	1.94	-	2.97
94	-	1.79	2.00	1.34	5.29	1.94	-	2.98
95	-	1.80	2.00	1.34	5.29	1.94	-	2.97
96	-	1.79	2.00	1.34	5.29	1.94	-	2.97
97	-	1.80	2.00	1.34	5.29	1.93	-	2.97
98	-	1.80	2.00	1.34	5.29	1.93	-	2.97
99	-	1.80	2.00	1.34	5.30	1.93	-	2.97
100	-	1.80	2.00	1.34	5.30	1.93	-	2.97
101	-	1.81	2.00	1.34	5.30	1.93	-	2.97
102	-	1.81	2.00	1.34	5.30	1.93	-	2.98
103	-	1.8	2.00	1.35	5.31	1.94	-	2.98
104	-	1.81	2.00	1.35	5.3	1.94	-	2.98
105	-	1.81	1.99	1.34	5.29	1.93	-	2.98
106	-	1.81	1.99	1.35	5.31	1.93	-	2.98
107	-	1.81	1.99	1.34	5.3	1.94	-	2.98
108	-	1.81	1.99	1.34	5.29	1.93	-	2.97
109	-	1.81	1.99	1.34	5.29	1.93	-	2.98
110	-	1.82	1.99	1.34	5.29	1.93	-	2.98

Table S 33. ELF basin populations for the IRC corresponding to **TS3b**.

	**V(C2,C3)**	**V(C3,C6)**	**V(C6,C8)**	**V(C7,Br)**	**V(O1)**	**V(C6,C7)**	**V(N,Br)**	**V(N)**
1	1.86	--	2.18	--	6.20	3.36	1.31	1.81
2	1.86	--	2.18	--	6.19	3.36	1.31	1.81
3	1.86	--	2.18	--	6.20	3.36	1.31	1.81
4	1.86	--	2.18	--	6.19	3.36	1.31	1.81
5	1.86	--	2.18	--	6.2	3.36	1.31	1.81
6	1.86	--	2.18	--	6.20	3.36	1.31	1.81
7	1.86	--	2.18	--	6.19	3.36	1.31	1.81
8	1.86	--	2.18	--	6.20	3.36	1.31	1.81
9	1.86	--	2.18	--	6.19	3.36	1.31	1.81
10	1.86	--	2.18	--	6.20	3.36	1.31	1.81
11	1.85	--	2.19	--	6.20	3.36	1.31	1.81
12	1.85	--	2.19	--	6.19	3.36	1.31	1.81
13	1.85	--	2.19	--	6.20	3.36	1.31	1.81
14	1.85	--	2.19	--	6.19	3.35	1.31	1.81
15	1.85	--	2.19	--	6.20	3.35	1.31	1.81
16	1.85	--	2.19	--	6.20	3.35	1.31	1.81
17	1.85	--	2.19	--	6.19	3.35	1.31	1.81
18	1.85	--	2.19	--	6.20	3.34	1.31	1.81

19	1.85	--	2.19	--	6.19	3.34	1.30	1.81
20	1.85	--	2.19	--	6.20	3.33	1.30	1.81
21	1.85	--	2.19	--	6.20	3.33	1.30	1.81
22	1.85	-	2.19	-	6.20	3.33	1.30	1.81
23	1.84	-	2.19	-	6.19	3.32	1.30	1.81
24	1.85	-	2.19	-	6.20	3.30	1.30	1.81
25	1.84	-	2.20	-	6.19	3.29	1.29	1.81
26	1.84	-	2.20	-	6.20	3.26	1.29	1.82
27	1.84	-	2.21	-	6.21	3.25	1.26	1.82
28	1.85	-	2.21	-	6.20	3.24	1.24	1.83
29	1.84	-	2.22	-	6.20	3.22	1.22	1.85
30	1.84	-	2.23	-	6.20	3.20	1.19	1.87
31	1.84	-	2.23	-	6.20	3.18	1.16	1.89
32	1.84	-	2.24	-	6.19	3.16	1.10	1.92
33	1.83	-	2.25	-	6.19	3.14	1.05	1.96
34	1.83	-	2.26	-	6.19	3.12	-	2.99
35	1.83	-	2.28	-	6.18	3.1	-	2.97
36	1.83	-	2.30	0.36	6.18	2.73	-	2.96
37	1.83	-	2.30	0.43	6.17	2.67	-	2.94
38	1.83	-	2.31	0.5	6.18	2.61	-	2.92
39	1.83	-	2.32	0.56	6.17	2.56	-	2.91
40	1.82	-	2.34	0.62	6.17	2.52	-	2.90
41	1.82	-	2.35	0.68	6.16	2.48	-	2.91
42	1.82	-	2.36	0.73	6.15	2.44	-	2.91
43	1.81	-	2.37	0.78	6.14	2.41	-	2.92
44	1.81	-	2.38	0.81	6.16	2.39	-	2.92
45	1.81	-	2.39	0.84	6.17	2.37	-	2.92
46	1.80	-	2.40	0.86	6.18	2.36	-	2.92
47	1.80	-	2.40	0.88	6.17	2.35	-	2.92
48	1.80	-	2.41	0.89	6.17	2.34	-	2.92
49	1.80	-	2.42	0.91	6.16	2.33	-	2.93
50	1.80	-	2.42	0.92	6.16	2.32	-	2.93
51	1.80	-	2.42	0.93	6.16	2.31	-	2.93
52	1.80	-	2.42	0.95	6.17	2.30	-	2.93
53	1.80	-	2.43	0.95	6.17	2.30	-	2.94
54	1.80	-	2.43	0.96	6.18	2.29	-	2.94
55	1.80	-	2.44	0.97	6.17	2.29	-	2.94
56	1.78	-	2.44	0.98	6.17	2.28	-	2.94
57	1.78	-	2.44	0.99	6.16	2.27	-	2.94
58	1.78	-	2.44	0.99	6.17	2.27	-	2.94
59	1.78	-	2.45	1.00	6.18	2.27	-	2.95
60	1.79	-	2.45	1.00	6.18	2.26	-	2.95
61	1.79	-	2.45	1.01	6.17	2.26	-	2.94
62	1.80	-	2.45	1.02	6.17	2.26	-	2.94
63	1.80	-	2.45	1.02	6.17	2.25	-	2.94
64	1.78	-	2.46	1.03	6.17	2.25	-	2.94
65	1.76	-	2.45	1.03	6.17	2.24	-	2.94
66	1.76	-	2.46	1.04	6.18	2.24	-	2.94
67	1.76	-	2.46	1.04	6.18	2.23	-	2.95
68	1.77	-	2.46	1.05	6.17	2.23	-	2.95
69	1.77	-	2.46	1.06	6.17	2.23	-	2.95
70	1.77	-	2.47	1.06	6.18	2.22	-	2.95
71	1.77	-	2.47	1.07	6.18	2.22	-	2.95
72	1.77	-	2.47	1.07	6.18	2.22	-	2.95
73	1.76	-	2.47	1.07	6.18	2.21	-	2.95
74	1.75	-	2.47	1.08	6.18	2.21	-	2.95
75	1.75	-	2.47	1.09	6.18	2.21	-	2.95
76	1.75	-	2.48	1.09	6.18	2.20	-	2.96

77	1.75	-	2.48	1.09	6.17	2.20	-	2.96
78	1.75	-	2.48	1.10	6.18	2.20	-	2.95
79	1.75	-	2.48	1.10	6.17	2.20	-	2.96
80	1.75	-	2.48	1.11	6.17	2.19	-	2.96
81	1.74	-	2.48	1.11	6.17	2.19	-	2.95
82	1.74	-	2.48	1.11	6.17	2.19	-	2.96
83	1.74	-	2.49	1.12	6.17	2.18	-	2.96
84	1.73	-	2.49	1.12	6.17	2.18	-	2.96
85	1.73	-	2.49	1.13	6.17	2.18	-	2.96
86	1.72	-	2.49	1.13	6.18	2.18	-	2.96
87	1.72	-	2.49	1.14	6.17	2.17	-	2.96
88	1.71	-	2.49	1.14	6.17	2.17	-	2.96
89	1.71	-	2.49	1.15	6.16	2.17	-	2.96
90	1.71	-	2.49	1.15	6.16	2.16	-	2.97
91	1.7	-	2.49	1.16	6.16	2.16	-	2.96
92	1.69	-	2.49	1.16	6.16	2.15	-	2.96
93	1.68	-	2.49	1.16	6.16	2.15	-	2.96
94	1.67	-	2.48	1.17	6.15	2.15	-	2.96
95	1.66	-	2.50	1.17	6.15	2.15	-	2.96
96	1.64	-	2.47	1.18	6.15	2.14	-	2.96
97	1.62	-	2.46	1.18	6.15	2.14	-	2.96
98	1.60	-	2.45	1.19	6.14	2.14	-	2.96
99	1.55	-	2.43	1.19	6.14	2.14	-	2.96
100	1.51	-	2.42	1.20	6.12	2.14	-	2.96
101	1.44	-	2.40	1.21	6.11	2.13	-	2.96
102	1.34	-	2.37	1.22	6.07	2.13	-	2.96
103	1.24	-	2.35	1.23	6.05	2.13	-	2.96
104	1.14	-	2.33	1.25	6.01	2.13	-	2.96
105	1.03	-	2.30	1.26	5.97	2.12	-	2.96
106	-	-	2.28	1.27	5.91	2.12	-	2.96
107	-	-	2.25	1.28	5.84	2.10	-	2.95
108	-	-	2.22	1.29	5.78	2.08	-	2.95
109	-	-	2.19	1.30	5.73	2.06	-	2.95
110	-	1.11	2.17	1.30	5.68	2.04	-	2.95
111	-	1.20	2.14	1.31	5.63	2.02	-	2.94
112	-	1.29	2.12	1.31	5.57	2.00	-	2.94
113	-	1.37	2.10	1.31	5.54	1.98	-	2.94
114	-	1.45	2.09	1.32	5.49	1.97	-	2.94
115	-	1.51	2.08	1.32	5.49	1.96	-	2.94
116	-	1.57	2.06	1.32	5.45	1.95	-	2.93
117	-	1.61	2.06	1.32	5.42	1.94	-	2.93
118	-	1.65	2.06	1.32	5.42	1.94	-	2.93
119	-	1.68	2.06	1.32	5.4	1.93	-	2.93
120	-	1.70	2.05	1.32	5.39	1.93	-	2.93
121	-	1.71	2.05	1.32	5.39	1.93	-	2.93
122	-	1.72	2.05	1.33	5.39	1.93	-	2.94
123	-	1.73	2.06	1.33	5.37	1.93	-	2.94
124	-	1.74	2.05	1.33	5.37	1.93	-	2.94
125	-	1.75	2.06	1.33	5.37	1.93	-	2.94
126	-	1.76	2.06	1.33	5.37	1.92	-	2.94
127	-	1.76	2.06	1.34	5.38	1.92	-	2.94
128	-	1.76	2.05	1.34	5.38	1.92	-	2.94
129	-	1.76	2.05	1.34	5.38	1.92	-	2.95
130	-	1.77	2.05	1.34	5.35	1.92	-	2.94
131	-	1.77	2.05	1.34	5.34	1.92	-	2.94
132	-	1.77	2.05	1.34	5.32	1.92	-	2.94
133	-	1.78	2.06	1.34	5.34	1.91	-	2.94
134	-	1.78	2.05	1.34	5.33	1.91	-	2.95

135	--	1.78	2.05	1.34	5.33	1.91	--	2.94
136	--	1.78	2.05	1.34	5.33	1.91	--	2.94
137	--	1.78	2.05	1.34	5.33	1.91	--	2.94
138	--	1.78	2.05	1.34	5.33	1.91	--	2.94
139	--	1.78	2.05	1.34	5.33	1.91	--	2.94
140	--	1.78	2.05	1.34	5.33	1.91	--	2.94
141	--	1.79	2.05	1.34	5.33	1.91	--	2.94
142	--	1.79	2.05	1.34	5.33	1.91	--	2.94
143	--	1.79	2.05	1.34	5.33	1.91	--	2.94
144	--	1.79	2.05	1.34	5.33	1.91	--	2.94
145	--	1.79	2.05	1.34	5.33	1.91	--	2.94
146	--	1.79	2.05	1.35	5.33	1.91	--	2.94
147	--	1.79	2.05	1.35	5.33	1.91	--	2.94
148	--	1.79	2.05	1.35	5.33	1.91	--	2.94
149	--	1.79	2.05	1.35	5.33	1.91	--	2.94
150	--	1.79	2.05	1.35	5.33	1.91	--	2.94

Table S 34. ELF basin populations for the IRC corresponding to **TS4b**.

	**V(C2,C3)**	**V(C3,C6)**	**V(C6,C8)**	**V(C7,Br)**	**V(O1)**	**V(C6,C7)**	**V(N,Br)**	**V(N)**
1	1.85	-	2.17	-	6.19	3.38	1.27	1.88
2	1.85	-	2.17	-	6.20	3.38	1.28	1.89
3	1.85	-	2.17	-	6.20	3.38	1.27	1.88
4	1.85	-	2.17	-	6.20	3.38	1.28	1.88
5	1.85	-	2.17	-	6.19	3.38	1.28	1.88
6	1.85	-	2.17	-	6.19	3.38	1.28	1.87
7	1.85	-	2.17	-	6.21	3.38	1.28	1.87
8	1.85	-	2.17	-	6.19	3.38	1.27	1.87
9	1.85	-	2.18	-	6.19	3.38	1.27	1.88
10	1.85	-	2.17	-	6.19	3.38	1.27	1.88
11	1.85	-	2.17	-	6.19	3.38	1.27	1.88
12	1.85	-	2.17	-	6.20	3.38	1.28	1.89
13	1.85	-	2.17	-	6.20	3.38	1.27	1.88
14	1.85	-	2.17	-	6.20	3.38	1.28	1.88
15	1.85	-	2.17	-	6.19	3.38	1.28	1.88
16	1.85	-	2.17	-	6.19	3.38	1.28	1.87
17	1.85	-	2.17	-	6.21	3.38	1.28	1.87
18	1.85	-	2.17	-	6.19	3.38	1.27	1.87
19	1.85	-	2.18	-	6.19	3.38	1.27	1.88
20	1.85	-	2.17	-	6.19	3.38	1.27	1.88
21	1.85	-	2.17	-	6.19	3.38	1.27	1.88
22	1.85	-	2.17	-	6.20	3.38	1.28	1.89
23	1.85	-	2.17	-	6.20	3.38	1.27	1.88
24	1.85	-	2.17	-	6.20	3.38	1.28	1.88
25	1.85	-	2.17	-	6.19	3.38	1.28	1.88
26	1.85	-	2.17	-	6.19	3.38	1.28	1.87
27	1.85	-	2.17	-	6.21	3.38	1.28	1.87
28	1.85	-	2.17	-	6.19	3.38	1.27	1.87
29	1.85	-	2.18	-	6.19	3.38	1.27	1.88
30	1.85	-	2.17	-	6.19	3.38	1.27	1.88
31	1.85	-	2.17	-	6.19	3.38	1.27	1.88
32	1.85	-	2.17	-	6.20	3.38	1.28	1.89
33	1.85	-	2.17	-	6.20	3.38	1.27	1.88

34	1.85	-	2.17	-	6.20	3.38	1.28	1.88
35	1.85	-	2.17	-	6.19	3.38	1.28	1.88
36	1.85	-	2.17	-	6.19	3.38	1.28	1.87
37	1.85	-	2.17	-	6.21	3.38	1.28	1.87
38	1.85	-	2.17	-	6.19	3.38	1.27	1.87
39	1.85	-	2.18	-	6.19	3.38	1.27	1.88
40	1.85	-	2.17	-	6.19	3.38	1.27	1.88
41	1.85	-	2.17	-	6.19	3.38	1.27	1.88
42	1.85	-	2.17	-	6.20	3.37	1.28	1.89
43	1.85	-	2.17	-	6.20	3.37	1.27	1.88
44	1.85	-	2.17	-	6.20	3.38	1.28	1.88
45	1.85	-	2.17	-	6.19	3.36	1.28	1.88
46	1.85	-	2.17	-	6.19	3.36	1.28	1.87
47	1.85	-	2.17	-	6.21	3.35	1.28	1.87
48	1.85	-	2.17	-	6.19	3.36	1.27	1.87
49	1.85	-	2.18	-	6.19	3.35	1.27	1.88
50	1.85	-	2.17	-	6.19	3.35	1.27	1.88
51	1.85	-	2.17	-	6.20	3.34	1.25	1.88
52	1.85	-	2.17	-	6.20	3.34	1.25	1.88
53	1.85	-	2.18	-	6.19	3.33	1.24	1.88
54	1.84	-	2.18	-	6.20	3.32	1.24	1.88
55	1.84	-	2.18	-	6.18	3.3	1.24	1.88
56	1.84	-	2.19	-	6.18	3.29	1.22	1.88
57	1.84	-	2.19	-	6.18	3.26	1.22	1.89
58	1.84	-	2.20	-	6.18	3.23	1.21	1.89
59	1.83	-	2.20	-	6.18	3.21	1.20	1.89
60	1.83	-	2.20	-	6.19	3.18	1.17	1.89
61	1.83	-	2.21	-	6.19	3.15	1.16	1.89
62	1.82	-	2.22	-	6.19	3.12	1.13	1.89
63	1.82	-	2.23	-	6.18	3.09	-	3.00
64	1.81	-	2.23	-	6.18	3.07	-	2.98
65	1.80	-	2.24	0.35	6.18	2.71	-	2.95
66	1.80	-	2.25	0.43	6.17	2.65	-	2.93
67	1.79	-	2.25	0.51	6.16	2.58	-	2.92
68	1.78	-	2.26	0.59	6.15	2.52	-	2.93
69	1.77	-	2.27	0.65	6.14	2.47	-	2.93
70	1.76	-	2.27	0.71	6.14	2.44	-	2.94
71	1.75	-	2.27	0.76	6.14	2.4	-	2.95
72	1.74	-	2.28	0.8	6.15	2.37	-	2.94
73	1.74	-	2.29	0.84	6.15	2.34	-	2.95
74	1.72	-	2.29	0.88	6.15	2.32	-	2.95
75	1.71	-	2.29	0.91	6.15	2.30	-	2.95
76	1.72	-	2.29	0.93	6.15	2.29	-	2.96
77	1.62	-	2.29	0.98	6.12	2.27	-	2.95
78	1.63	-	2.29	0.98	6.14	2.26	-	2.96
79	1.54	-	2.28	1.02	6.13	2.24	-	2.95
80	1.40	-	2.27	1.08	6.09	2.22	-	2.95
81	1.25	-	2.26	1.12	6.03	2.21	-	2.94
82	1.11	-	2.26	1.18	5.97	2.19	-	2.94
83	-	-	2.24	1.22	5.89	2.17	-	2.94
84	-	-	2.21	1.26	5.79	2.14	-	2.94
85	-	1.02	2.18	1.29	5.71	2.09	-	2.93
86	-	1.16	2.15	1.31	5.61	2.04	-	2.93
87	-	1.30	2.13	1.32	5.56	2.01	-	2.93
88	-	1.43	2.10	1.33	5.50	1.97	-	2.92
89	-	1.53	2.08	1.33	5.46	1.96	-	2.92
90	-	1.61	2.07	1.34	5.42	1.95	-	2.92
91	-	1.66	2.06	1.33	5.41	1.94	-	2.92

92	-	1.70	2.06	1.33	5.40	1.94	-	2.93
93	-	1.72	2.06	1.34	5.39	1.93	-	2.93
94	-	1.74	2.06	1.34	5.38	1.93	-	2.94
95	-	1.75	2.07	1.34	5.37	1.92	-	2.94
96	-	1.76	2.07	1.34	5.37	1.92	-	2.94
97	-	1.76	2.07	1.34	5.36	1.92	-	2.94
98	-	1.77	2.06	1.34	5.36	1.92	-	2.95
99	-	1.78	2.06	1.35	5.36	1.91	-	2.94
100	-	1.78	2.06	1.35	5.35	1.91	-	2.95
101	-	1.79	2.06	1.35	5.31	1.91	-	2.94
102	-	1.79	2.06	1.35	5.29	1.90	-	2.94
103	-	1.80	2.07	1.35	5.31	1.90	-	2.95
104	-	1.80	2.07	1.35	5.32	1.90	-	2.96
105	-	1.80	2.07	1.35	5.32	1.90	-	2.95
106	-	1.80	2.06	1.36	5.33	1.90	-	2.96
107	-	1.81	2.06	1.36	5.32	1.90	-	2.95
108	-	1.81	2.06	1.36	5.31	1.89	-	2.96
109	-	1.81	2.06	1.36	5.30	1.89	-	2.96
110	-	1.81	2.06	1.36	5.29	1.88	-	2.97
111	-	1.81	2.06	1.36	5.29	1.88	-	2.96
112	-	1.81	2.06	1.36	5.28	1.88	-	2.97
113	-	1.81	2.06	1.36	5.26	1.88	-	2.96
114	-	1.82	2.06	1.35	5.25	1.88	-	2.97
115	-	1.82	2.06	1.36	5.26	1.88	-	2.96
116	-	1.82	2.06	1.36	5.26	1.88	-	2.97
117	-	1.82	2.06	1.36	5.25	1.87	-	2.98
118	-	1.82	2.06	1.36	5.26	1.87	-	2.98
119	-	1.82	2.06	1.36	5.27	1.87	-	2.98
120	-	1.83	2.06	1.36	5.26	1.87	-	2.98
121	-	1.83	2.06	1.36	5.24	1.87	-	2.97
122	-	1.82	2.05	1.36	5.24	1.87	-	2.97
123	-	1.83	2.05	1.36	5.25	1.87	-	2.98
124	-	1.83	2.05	1.36	5.24	1.87	-	2.98
125	-	1.83	2.06	1.36	5.25	1.87	-	2.99
126	-	1.83	2.05	1.36	5.25	1.87	-	2.99
127	-	1.83	2.05	1.36	5.25	1.86	-	2.99
128	-	1.83	2.05	1.37	5.25	1.86	-	2.99
129	-	1.84	2.05	1.36	5.24	1.87	-	2.99
130	-	1.84	2.05	1.37	5.25	1.86	-	2.98
131	-	1.84	2.05	1.37	5.24	1.86	-	2.98
132	-	1.84	2.05	1.37	5.24	1.86	-	2.98
133	-	1.84	2.05	1.37	5.24	1.86	-	2.99
134	-	1.84	2.05	1.37	5.23	1.86	-	2.99
135	-	1.84	2.05	1.37	5.23	1.86	-	2.99
136	-	1.84	2.05	1.37	5.23	1.86	-	2.99
137	-	1.84	2.05	1.37	5.23	1.86	-	2.99
138	-	1.84	2.05	1.37	5.21	1.86	-	2.98
139	-	1.84	2.04	1.37	5.21	1.86	-	2.99
140	-	1.84	2.05	1.37	5.21	1.87	-	2.99
141	-	1.84	2.05	1.36	5.20	1.86	-	2.99
142	-	1.84	2.05	1.37	5.20	1.86	-	2.99
143	-	1.84	2.05	1.37	5.21	1.87	-	2.99
144	-	1.84	2.05	1.37	5.20	1.87	-	2.99
145	-	1.84	2.05	1.37	5.21	1.86	-	2.99
146	-	1.84	2.04	1.37	5.20	1.86	-	2.99
147	-	1.84	2.04	1.37	5.19	1.86	-	2.99
148	-	1.84	2.04	1.37	5.19	1.86	-	2.99
149	-	1.84	2.05	1.37	5.19	1.86	-	2.99

150	-	1.85	2.04	1.37	5.19	1.86	-	2.99
151	-	1.84	2.04	1.36	5.19	1.86	-	2.99
152	-	1.84	2.04	1.36	5.19	1.86	-	2.99
153	-	1.84	2.04	1.37	5.20	1.87	-	2.99
154	-	1.84	2.04	1.36	5.20	1.87	-	2.99
155	-	1.85	2.04	1.36	5.19	1.86	-	2.99
156	-	1.85	2.04	1.36	5.20	1.87	-	3.00
157	-	1.85	2.04	1.36	5.20	1.87	-	2.99
158	-	1.85	2.04	1.36	5.20	1.87	-	2.99
159	-	1.84	2.04	1.36	5.20	1.87	-	2.99
160	-	1.84	2.04	1.37	5.20	1.87	-	2.99

Stabilization of the intermediate

A stepwise processed was achieved by placing a dimetylamino group instead the aromatic ring. In this case a stable intermediate iminium ion was located. From this intermediate, paths towards an epoxide and a cyclopentanone were calculated (Scheme S 15).

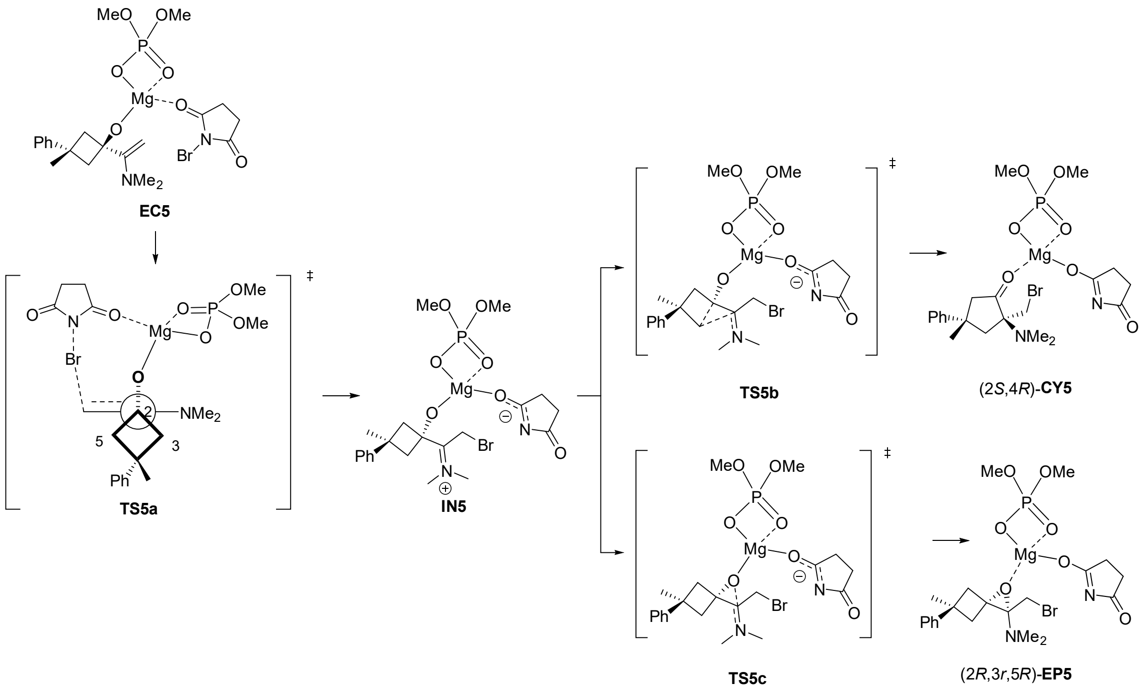

Scheme S 15. Stabilization of intermediate as iminium ion. Only the attack by one face of the iminium ion has been calculated.

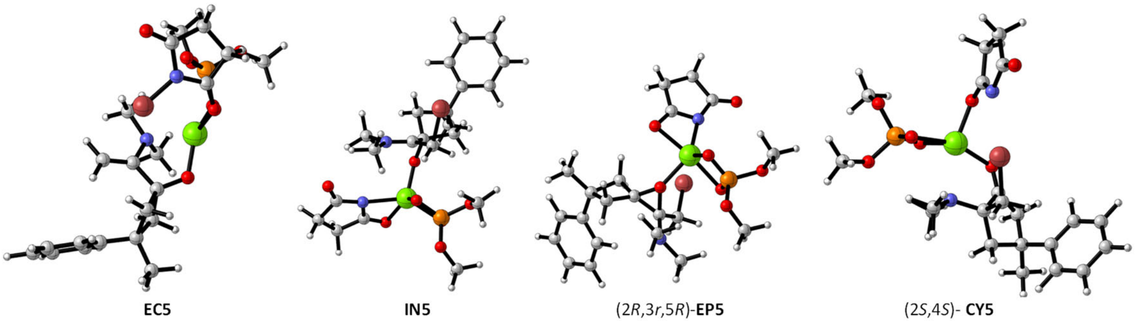

Figure S 83. Optimized (wb97xd/def2SVP) geometries of encounter complex **EC5**, intermediate **IN5**, epoxide (2*R*,3*r*,5*R*)-**EP5** and cyclopentanone (2*S*,4*R*)-**CY5**

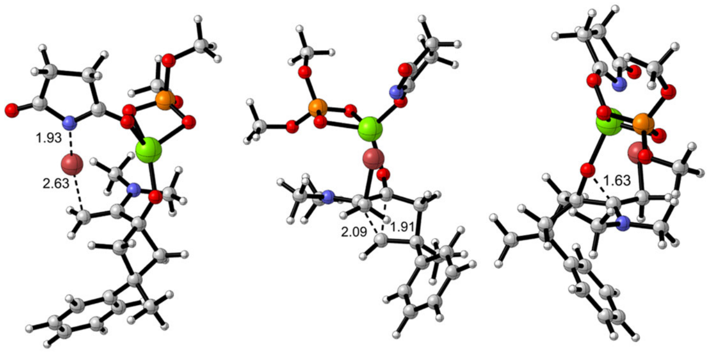

TS5a **TS5b** **TS5c**

Figure S 84. Optimized (wb97xd/def2SVP) geometries of transition structures **TS5a**, **TS5b** and **TS5c**.

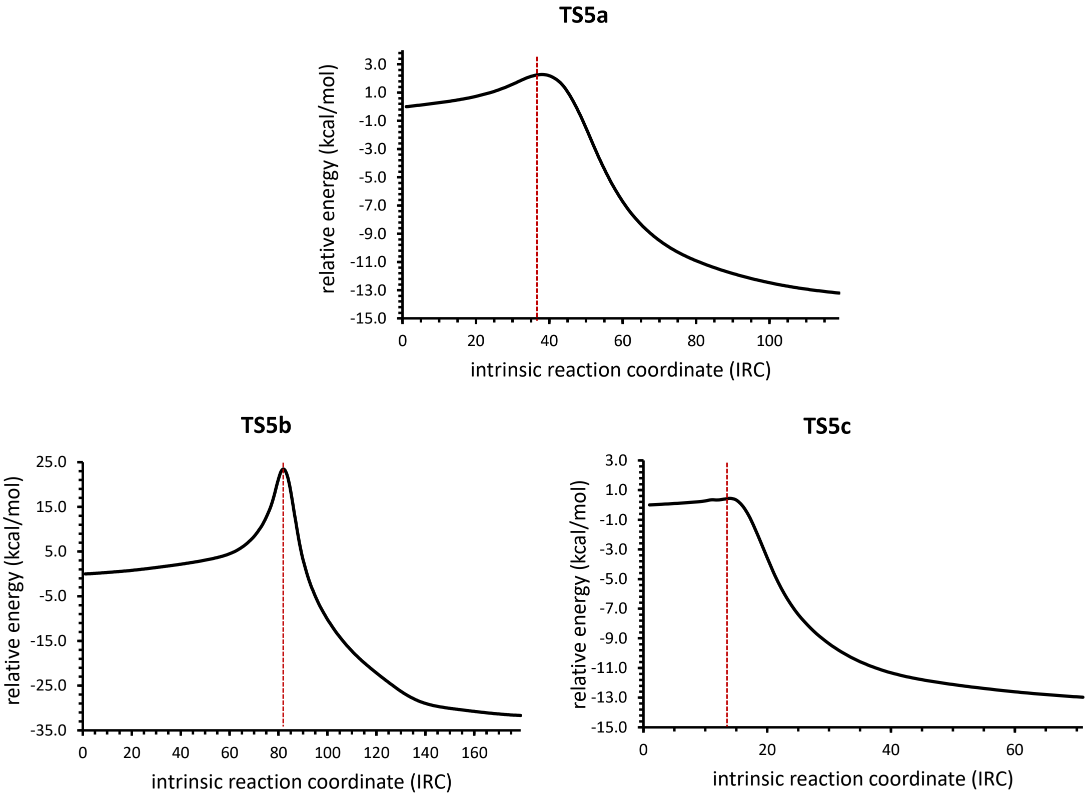

Figure S 85. IRCs (wb97xd/def2svp) corresponding to transition structures **TS5a**, **TS5b** and **TS5c**.

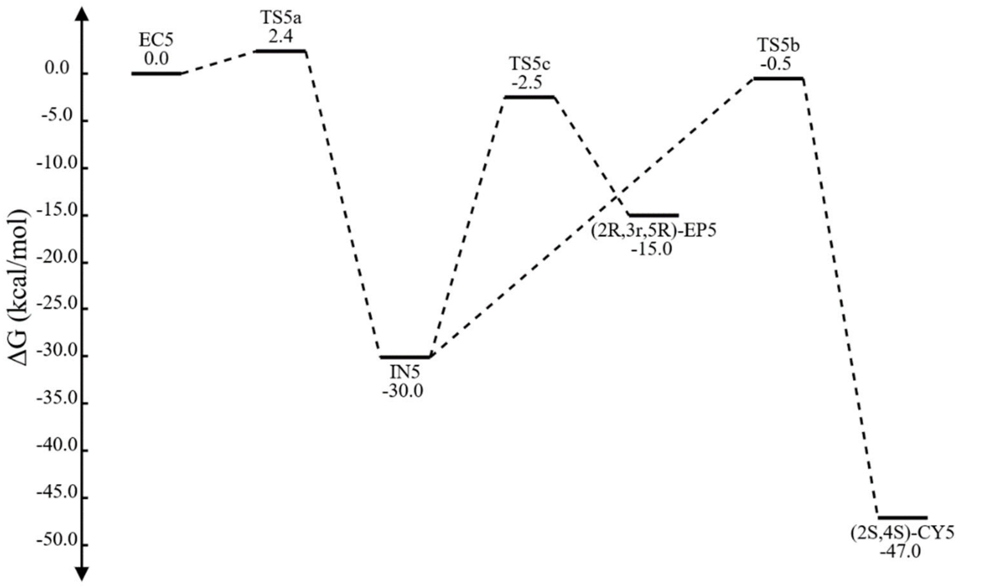

Figure S 86. Energy profiles (wb97xd/def2tzvp/pcm=toluene)//wb97xd/def2svp) for the different pathways from **EC5** to **EP5** and **CY5.** Relative free energies are given in kcal/mol.

Table S 35. Calculated (wb97xd/def2tzvp/pcm=toluene//wb97xd/def2svp) absolute (hartree) and relative (kcal/mol) energies for the transformation of **EC5** into **EP5** and **CY5** .

	E_0	ΔE_0	G	ΔG	im. freq
**EC5**	-4569.880067	0.0	-4569.974554	0.0	
**TS5a**	-4569.876584	2.2	-4569.970712	2.4	-118.0
**IN5**	-4569.925222	-28.3	-4570.022437	-30.0	
**TS5b**	-4569.875033	3.2	-4569.974764	-0.1	-485.1
**TS5c**	-4569.884170	-2.6	-4569.978612	-2.5	-225.6
(2*S*,4*S*)-**CY5**	-4569.955115	-47.1	-4570.049506	-47.0	
(2*R*,3*r*,5*R*)-**EP5**	-4569.900108	-12.6	-4569.998385	-15.0	

Real Model with Real Catalyst

Consideration of the chiral catalyst duplicates the approaches illustrated in Scheme S 14 We located 8 transition structures (**TS2a1** and **TS2a2** leading to isomeric epoxides and **TS2b1**, **TS2b2**, **TS2c1**, **TS2c2**, **TS2d1** and **TS2d2** leading to isomeric cyclopentanones) (Scheme S 16). The optimized structures are given in Figure S 87 and their absolute and relative energies in Table S 36.

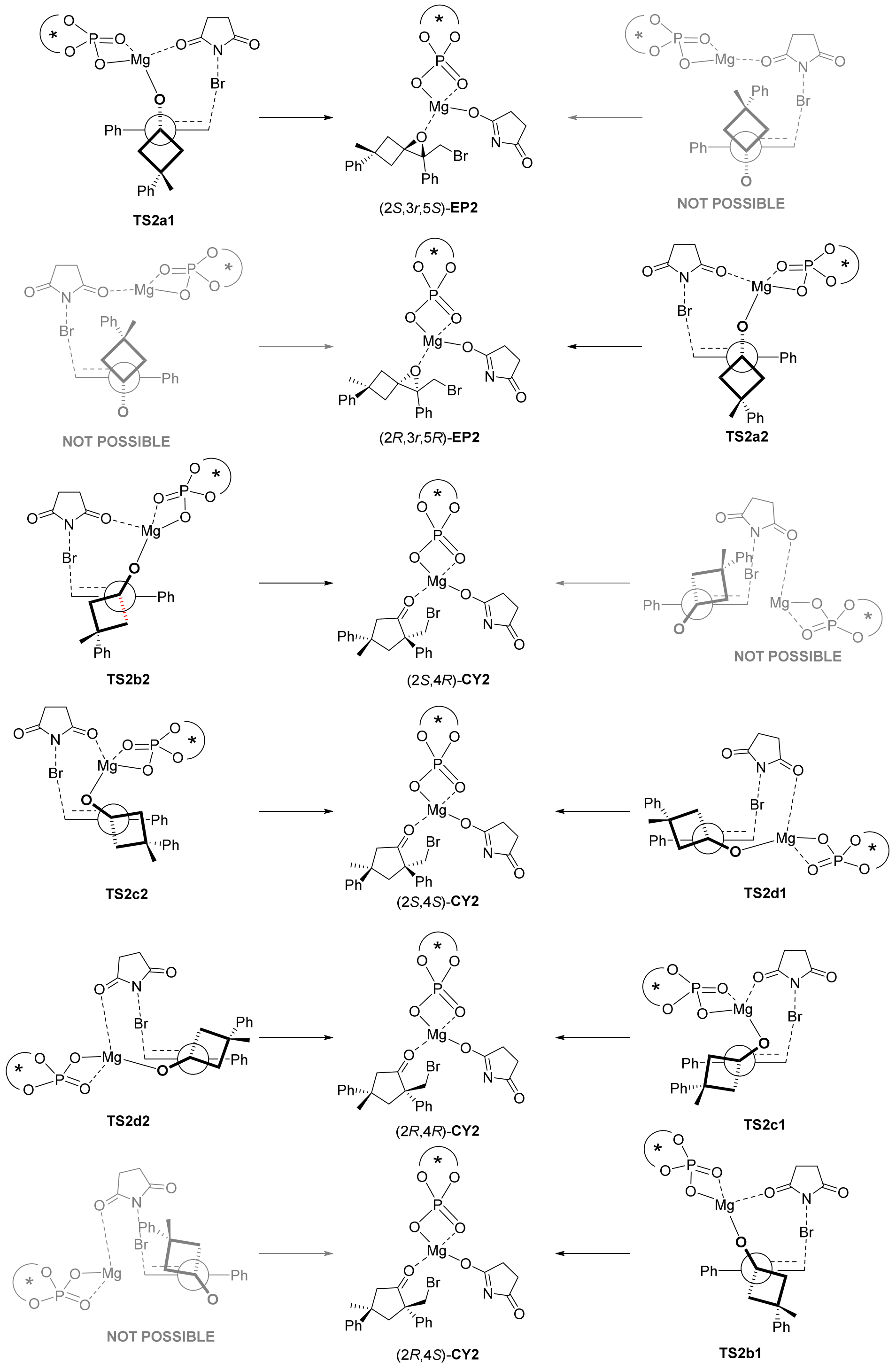

Scheme S 16. Approaches for the real model with the real catalyst.

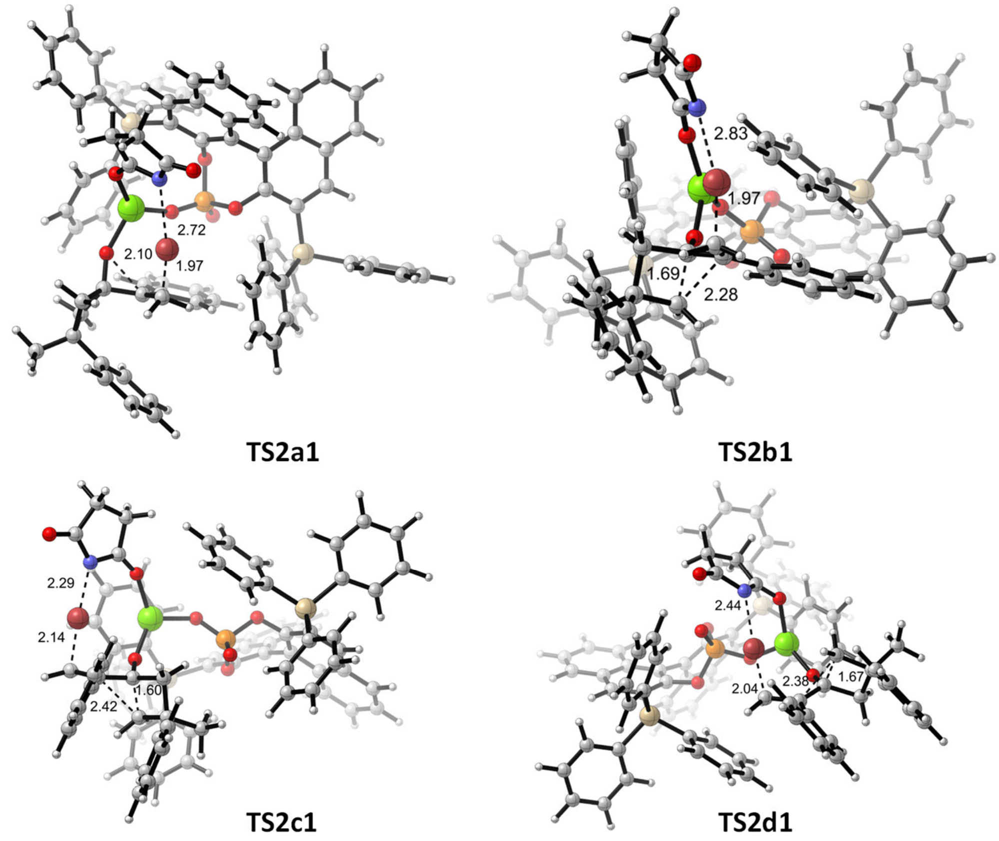

Figure S 87. Optimized (wb97xd/def2SVP:PM6) geometries of transition structures **TS2a1**, **TS2b1**, **TS2c1** and **TS2d1**.

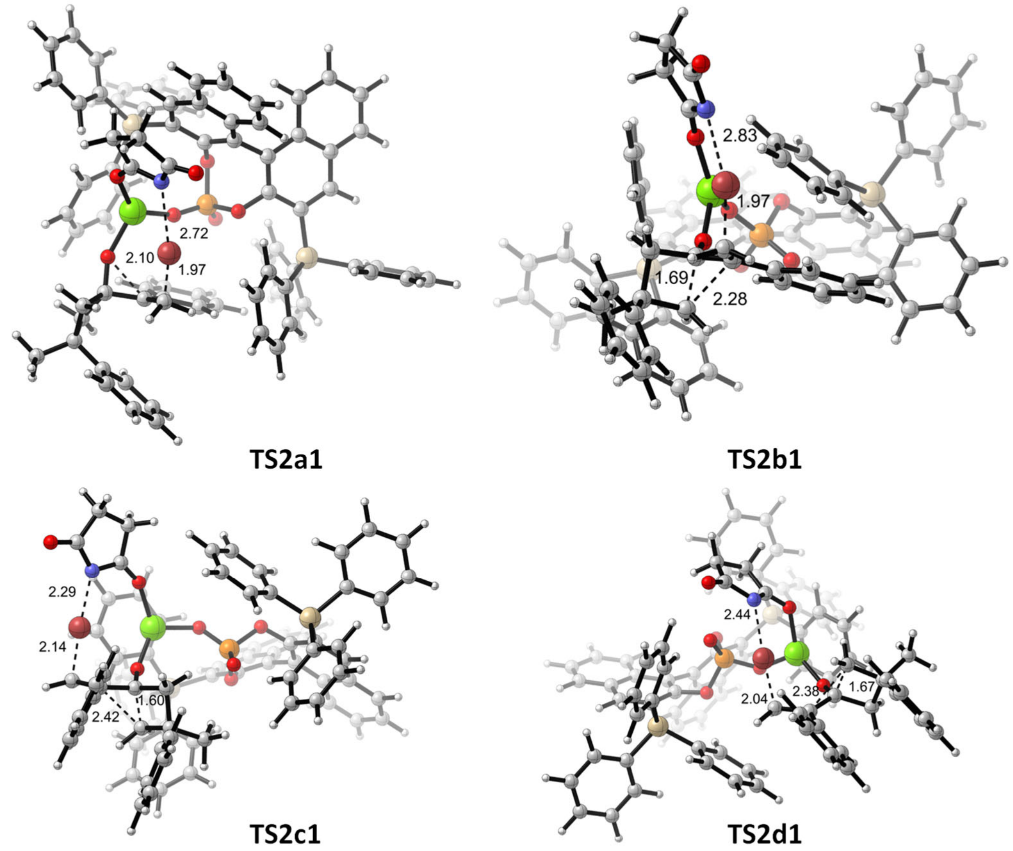

Figure S 88. Optimized (wb97xd/def2SVP:PM6) geometries of transition structures **TS2a2**, **TS2b2**, **TS2c2** and **TS2d2**.

Table S 36. Calculated (wb97xd/def2tzvp/pcm=toluene:PM6//wb97xd/def2svp:PM6) absolute (hartree) and relative (kcal/mol) energies for the of transition structures illustrated in Scheme S 16.

	E_0	ΔE_0[a]	$\Delta\Delta E_0$[b]	G	ΔG[a]	$\Delta\Delta G$[b]	im. freq
**ECr**	-5355.664952	0.0		-5355.843317	0.0		
**TS2a1**	-5355.658142	4.3	0.0	-5355.831175	7.6	2.3	-117.4
**TS2b1**	-5355.655543	5.9	1.6	-5355.834840	5.3	0.0	-194.8
**TS2c1**	-5355.650568	9.0	4.8	-5355.827010	10.2	4.9	-88.9
**TS2d1**	-5355.643397	13.5	9.3	-5355.821245	13.9	8.5	-88.1
**TS2a2**	-5355.652947	7.5	3.3	-5355.827309	10.0	4.7	-106.9
**TS2b2**	-5355.653358	7.3	3.0	-5355.829366	8.8	3.4	-118.3
**TS2c2**	-5355.651228	8.6	4.3	-5355.827379	10.0	4.7	-152.5
**TS2d2**	-5355.635444	18.5	14.2	-5355.809567	21.2	15.9	-119.2
**(2*R*,3*r*,5*R*)-EPr**	-5355.715864	-31.9		-5355.888529	-28.4		
**(2*S*,3*r*,5*S*)-EPr**	-5355.675336	-6.5		-5355.849791	-4.1		
**(2*R*,4*R*)-CYr**	-5355.769241	-65.4		-5355.942932	-62.5		
**(2*R*,4*S*)-CYr**	-5355.768198	-64.8		-5355.942795	-62.4		
**(2*S*,4*R*)-CYr**	-5355.759003	-59.0		-5355.934484	-57.2		
**(2*S*,4*S*)-CYr**	-5355.753488	-55.6		-5355.930652	-54.8		

[a] Referred to **ECr**. [b] Referred to the minimum value of the transition structures

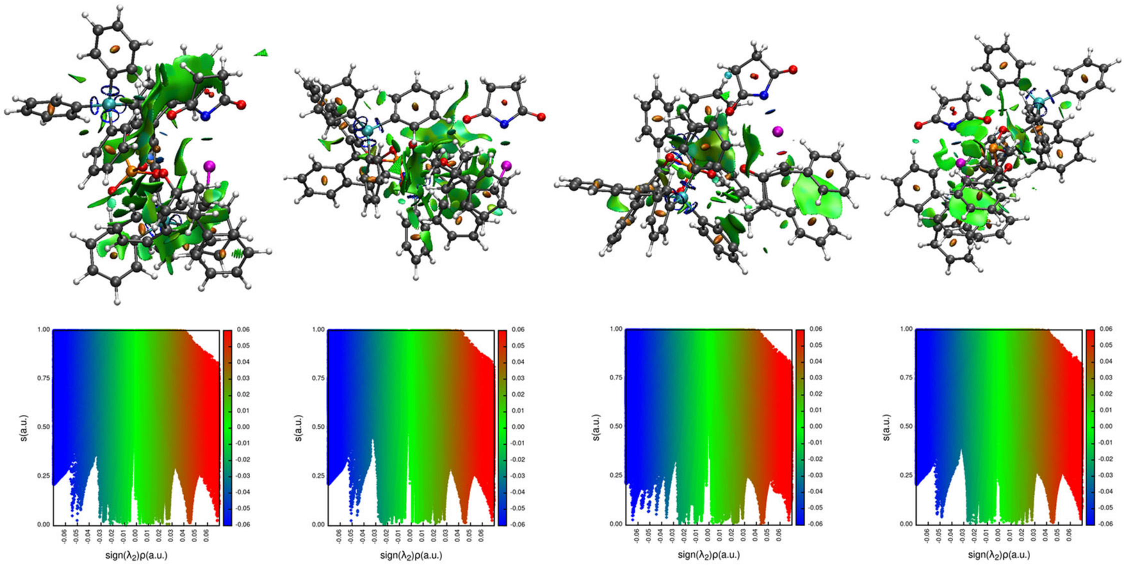

TS2a1 **TS2b1** **TS2c1** **TS2d1**

Figure S 89. NCI analysis for transition structures **TS2a1**, **TS2b1**, **TS2c1** and **TS2d1**. Top: Isosurfaces. Bottom: s(**r**) against *sign*(λ_2)ρ(**r**) plots (see text).

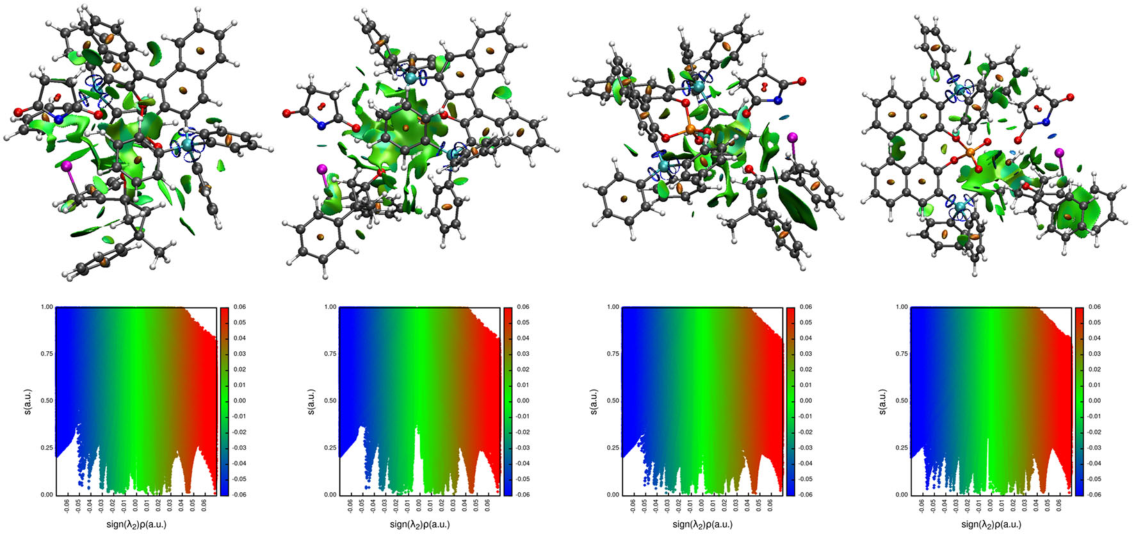

TS2a2 **TS2b2** **TS2c2** **TS2d2**

Figure S 90. NCI analysis for transition structures **TS2a2**, **TS2b2**, **TS2c2** and **TS2d2**. Top: Isosurfaces. Bottom: s(**r**) against *sign*(λ_2)ρ(**r**) plots (see text).

Table S 37 Integration over the volumes of *sign*(λ_2)ρ(**n**) for n=2.5 according to ref. 37 for **TS2a1**, **TS2b1**, **TS2c1** and **TS2d1**

interval	TS2a1	TS2b1	TS2c1	TS2d1
**all**	-8.22861886	-8.24809882	-8.23758177	-8.24182234
**-1.0 to -0.02**	-0.64825856	-0.65183371	-0.65442657	-0.65456785
**-0.02 to 0.02**	0.01078311	0.01062379	0.01121693	0.01112437
**0.02 to 1.0**	0.80472338	0.80664636	0.80362521	0.80557497

[a] The interval [-0.1, -0.02] gives information about strong NCI (H-bond, halogen, etc.). The interval [-0.02, 0.02] gives information about van der Waals interactions. The interval [0.02, 1.00] gives information about steric clashes.

Table S 38 Integration over the volumes of *sign*(λ_2)ρ(**n**) for n=2.5 according to ref. 37 for **TS2a2**, **TS2b2**, **TS2c2** and **TS2d2**.

interval	TS2a2	TS2b2	TS2c2	TS2d2
**all**	-8.24209417	-8.23800821	-8.24454098	-8.23658102
**-1.0 to -0.02**	-0.65124256	-0.65469843	-0.65292160	-0.65315603
**-0.02 to 0.02**	0.01049493	0.01117797	0.01144069	0.01108816
**0.02 to 1.0**	0.80401630	0.80813914	0.80717058	0.80439725

[a] The interval [-0.1, -0.02] gives information about strong NCI (H-bond, halogen, etc.). The interval [-0.02, 0.02] gives information about van der Waals interactions. The interval [0.02, 1.00] gives information about steric clashes.

Molecular Dynamic Simulations

The following file corresponds to the "progdyn.conf" file used in the Progdyn script package. ¡Error! Marcador no definido. It contains information about the level of theory, charge, multiplicity, and all other inputs for a Gaussian electronic structure calculation. Transition structure **TS2b**, calculated at the ωB97XD/def2svp level of theory was used as the input structure for the molecular dynamics simulations.

--- Start of file

```
#This is the configuration file for PROGDYN.  This file is read by progdynstarterHP and
# the awk programs proggenHP, prog1stpoint, prog2ndpoint, and progdynb.
#The programs won't read anything past the first blank line,
#and this file must end with a blank line.
#The program has a number of default values but they are unlikely to be what you want.
#Do not delete lines - rather, comment out lines for unwanted options.
#The values here are read repeatedly and most can be changed in the middle of running jobs
#***The keywords are case sensitive.  The following keywords should always be defined:***
#***method, charge, multiplicity, memory, processors, title
#*** method --The following word is copied exactly to the gaussian input file.
method wb97xd/def2svp
#To do a nonstandard route, make nonstandard 1.  For normal calcs, use nonstandard 0 or else leave it out.
#Then make a file called "nonstandard" containing the nonstandard route with no extra lines.
#nonstandard 0
# NMRoptions As is NMRtype=1 will add a section for an NMR calc at every NMRevery intervals. If you want to combine the two use nonstandard
#NMRtype 1
#NMRmethod2 B97D/6-31G*
#NMRmethod LC-wPBE/6-31G*
#NMRmethod3 B3LYP/cc-pvtz
#NMRevery 4
#NMRrand 1
#NMRcc 1
#loadlimit 10.0
#geometry linear
rotationmode 1
#*** method2 --The options here are restricted, unrestricted, and read. restricted is the default
#If the method is U..., put unrestricted here and the .com files will have in them guess=mix.
#If you put read here, the .com files will contain guess=tcheck, which sometimes makes things faster, sometimes not.
#The use of read requires a specifically defined checkpoint file name using the keyword checkpoint.
method2 restricted
charge 0
multiplicity 1
#oniomchargemult 1 1
processors 16
#*** memory --The following "word" is copied exactly to the gaussian input file after %mem=.
```

memory 8gb
#*** killcheck and checkpoint -- You can use a specifically defined checkpoint file name by putting
#the name after the keyword checkpoint. This is necessary if you use the read option with method2.
#Defined checkpoint names are an unnecessary modest hastle and if you do not want to bother, use killcheck 1
killcheck 1
#checkpoint g09.chk
#*** diagnostics -- 0 prints out nothing extra, 1 (default) prints out extra stuff to a
#file "diagnostics", 2 adds more stuff, 3 adds velocities to a file "vellist"
#4 adds the apparent temperature to vellist, but this is meaningless with quasiclassical calculations
diagnostics 4
#*** title -- the title keyword must be followed by exactly four words (the first after title is loaded by proganal)
title title CB08 TS1b model
#*** initialdis -- 0 (default) turns off displacement of the normal modes, so that all trajectories start from the same place
and only the energies and signs of the motion in the modes are randomized
1 gives a flat distribution of displacements where all of the possible values are equally likely
2 (recommended) gives a QM-like gaussian distribution of displacements, so that displacements in the middle are more likely that
those at the end by 1/e
initialdis 0
#*** timestep -- this is the time between points in the trajectory. Typical values would be 1E-15 or 0.5E-15 or 0.25E-15
timestep 1E-15
#*** scaling -- this lets you scale the gaussian frequencies by a constant
scaling 1.0
temperature 298.15
#*** thermostat 1 puts in a damping factor so as to bring the classical temperature toward the desired temperature.
#*** use a thermostatmult between 0.95 and 1, typically 0.995, so the damping happens slowly - otherwise there will be
#*** overadjustment in response to random variation
#*** the thermostat is not exact. The second traj point ignores this, so it only applies to later points handled by progdynb.
thermostat 1
thermostatmult 0.999
#*** method3, method4, method5, and method6 -- These keywords let you add extra lines to the gaussian input file.
#method3 and method4 add lines at the top of the input after the lines defining the method, and
#this is useful to implement things like the iop for mPW1k
#method5 and method6 add lines after the geometry, after a blank line of course
#only a single term with no spaces can be added, one per method line. Here are some examples to uncomment if needed
#method3 IOp(3/76=0572004280)
#method3 scrf=(pcm,solvent=ethanol)
#method3 scrf=(pcm,Solvent=dichloromethane)

#add the line below with big structures (more than 50 atoms) to get it to put out the distance matrix and the input orientation
method3 iop(2/9=2000)
#other possibilities
#method4 emp=gd3bj
method4 int=ultrafine
#method4 scf=(conver=5)
#method4 iop(3/124=3)
#method4 scrf=(pcm,solvent=dmso,read)
#method5 radii=bondi
#method6
#*** methodfile -- This keyword lets you add more complicated endings to gaussian input files
#such as a gen basis set. Put after the keyword the number of lines in a file you create called
#methodfile that contains the test you want to add to the end of the gaussian input
methodfile 0
#*** numimag --This tells the program the number of imaginary frequencies in the starting structure.
#if 0, treats as ground state and direction of all modes is random
#if 1, motion along the reaction coordinate will start out in the direction defined by searchdir
#if 2, only lowest freq will go direction of searchdir and other imag mode will go in random direction
numimag 1
#*** searchdir -- This keyword says what direction to follow the mode associated with the imaginary frequency.
#The choices are "negative" and "positive". Positive moves in the direction defined in the gaussian frequency calculation
#for the imaginary frequency, while negative moves in the opposite direction. The correct choice can be made either
#by a careful inspection of the normal modes and standard orientation geometry, or by trial and error.
searchdir negative
#*** classical -- for quassiclassical dynamics, the default, use 0. for classical dynamics, use 1
#if there are no normal modes and the velocities are to be generated from scratch, use classical 2
classical 0
#*** DRP, saddlepoint, and maxAtomMove --to run a DRP use 'DRP 1' in the line below, otherwise leave it at 0 or comment it out
#the treatment of starting saddlepoints is not yet implemented so use saddlepoint no
#if DRP shows oscillations then decrease maxAtomMove
#DRP 1
#saddlepoint no
#maxAtomMove 0.01
#*** cannonball -- The program can "fire" a trajectory from a starting position toward a particular target, such as toward
#a ts. To use this, make a file cannontraj with numAtom lines and three numbers per line that defines the vector
#for firing the trajectory, relative to the starting geometry's standard orientation. The number following cannonball sets
#the extra energy being put into the structure in kcal/mol
#cannonball 10
#*** keepevery --This tells the program how often to write the gaussian output file to file dyn, after the first two points.

#Use 1 for most dynamics to start with, but use a higher number to save on disk space or molden loading time.
keepevery 1
#*** highlevel --For ONIOM jobs, the following line states the number of highlevel atoms,
#which must come before the medium level atoms. Use some high value such as 999 if not using ONIOM
highlevel 9999
#*** fixedatom1, fixedatom2, fixedatom3, and fixedatom4 - These fix atoms in space.
#Fixing one atom serves no useful purpose and messes things up, while fixing two atoms
#fixes one distance and fixing three has the effect of fixing three distances, not just two
#in current form fixed atoms only are meant to work with no displacements, that is, initialdis=0
#fixedatom1 16
#fixedatom2 1
#fixedatom3 4
#fixedatom4 20
#applyforce 1 lets one push atoms together or appart - a positive force pushes them together
#format is applyforce force - with the units on force the same as in the Gaussian output file
#applyforce 2 or 3 or 4 applys a polynomical force centered at dist0. 2 is just harmonic, 3 is second order, 4 is third order
#format is applyforce 4 forcecoefficient dist0 forcecoefficient2 forcecoefficient3
#then use afatoms to chose the atoms with format afatoms firstatom secondatom [additional atoms]
#applyforce 2 0.1 2.1
#afatoms 16 1 2 3 4 5 6
#applyforceB 2 0.01 5.2
#afatomsB 8 15
#applyforceC 2 0.01 5.2
#afatomsC 8 15
#zeroatom pushes the numbered atom toward the origin with a small harmonic potential - good with boxon when you want to keep the reaction in the center
#zeroatom 16
#*** boxon and boxsize - With boxon 1, a cubic box is set such that atoms that reach the edge
#are reflected back toward the middle. Useful for dynamics with solvent molecules. This is a crude
#implementation that is ok for a few thousand femtoseconds but will not conserve energy long term.
#Set the box size so as to fit the entire initial molecule but not have too much extra room.
#The dimensions of the box are two times the boxsize, e.g. boxsize 7.5 leads to a box that is 15 x 15 x 15 angstroms
boxon 0
boxsize 8
#*** sphereon and spheresize and sphereforce - uses a force to push atoms within a sphere. notice that if the atom is far outside of
#the sphere then the force is large unless sphereforce is set small
#sphereon 1
#spheresize 12.9
#sphereforce .01
#setting a value for empiricaldispersion sets its s6 value with the Grimme 2006 algorithm. Default is 0, with no empiricaldispersion; 1.0 for correction
empiricaldispersion 0.0
#*** displacements -- This keyword lets you set the initialdis of particular modes by using a series of lines of the format

displacements NumberOfMode InitialDisForThatMode, as in the example below. You should be able to do as many of these as you like
you might consider this for rotations where a straight-line displacement goes wrong at large displacements
The choices for InitialDisForThatMode are 0, 1, 2, and 10, where 10 does the same thing as 0 but is maintained for now because
a previous version of the program had a bug that made 0 not work.
#displacements 2 0
#displacements 3 0
#displacements 4 0
#displacements 5 0
#displacements 6 0
#displacements 7 0
#displacements 8 0
#displacements 9 0
#displacements 10 0
#*** etolerance --This sets the allowable difference between the desired energy in a trajectory and the actual
#energy, known after point 1 from the potential energy + the kinetic energy in the initial velocities.
#The unit is kcal/mol and 1 is a normal value for mid-sized organic systems. For very large and floppy molecules, a larger value
#may be needed, but the value must stay way below the average thermal energy in the molecule (not counting zpe).
#If initialdis is not 0 and few trajectories are being rejected, decrease the value.
etolerance 1
#*** controlphase --It is sometimes useful to set the phase of particular modes in the initialization of trajectories.
#The format is controlphase numberOfModeToControl positive or controlphase numberOfModeToControl negative.
#controlphase 2 positive
#*** damping -- The damping keyword lets you add or subtract energy from the system at each point, by multiplying the velocities
#by the damping factor. A damping of 1 has no effect, and since you mostly want to change the energy slowly, normal values range
#from 0.95 to 1.05. The use of damping lets one do simulated annealing - you add energy until the structure is moving enough
#to sample the kinds of possibilities you are interested in, then you take away the energy slowly.
damping 1.00
#at a damping of .9995, the energy is cut in half in 693 points
#*** reversetraj --This keyword sets the trajectories so that both directions from a transition state are explored.
reversetraj true
#updated Aug 9, 2007 to include the possibility of classical dynamics by the keyword classical
#updated Jan 2008 to include fixed atoms, ONIOM jobs, keepevery, and box size
#update Feb 2008 to include methodfile parameter
updated Nov 2008 to allow for start without an initial freq calc using classical = 2
update Aug 2010 to include etolerance, damping controlphase and reversetraj
--- End of file

Cartesian Coordinates

Catalyst

CATa-MeOH
0 1

Mg	-0.1264955464	0.7810007121	0.2231375775
O	-1.9720070365	0.4044166471	0.1335976432
O	1.7562687404	0.9775358192	0.3116246580
O	-2.7616162815	-0.3084572562	-2.2426475454
O	2.7993886856	0.5170256460	2.6434769647
P	2.9822303264	0.7832895982	1.1944727179
O	3.8326328148	-0.3754107527	0.3771430088
O	3.9333673052	2.1085790058	1.0161087291
C	5.1393146020	-0.6495026190	0.7763753446
C	4.7641234238	2.3382869339	-0.0694447135
C	6.1081650280	0.3307794808	0.6095994329
C	5.4306905300	-1.9587331860	1.2522142146
C	5.8560475959	1.5112659659	-0.2600582744
C	4.4723798523	3.4679638397	-0.8852767433
C	7.3896450435	0.1234531209	1.2210583231
C	6.7086993637	-2.1762432415	1.7378926902
Si	4.2742769798	-3.4525040873	1.1045631712
C	6.7139709662	1.7679044844	-1.3882981532
C	5.3732748607	3.7667949208	-1.8903364907
Si	2.7861894120	4.3119060819	-0.9492172289
C	8.3755556772	1.1427194081	1.2950896588
C	7.6823354226	-1.1485614319	1.8027150916
H	6.9896270501	-3.1648122870	2.0873272189
C	3.4237228832	-3.5012385853	-0.5788254894
C	3.0597279513	-3.5527927256	2.5310778158
C	5.3576942556	-4.9926359189	1.1960545662
C	7.7596765637	0.8915637795	-1.7857637227
C	6.4929967353	2.9442114287	-2.1717523829
H	5.1979870180	4.6241727234	-2.5330941715
C	1.6495066575	3.0341593742	-1.7500700196
C	2.9036329647	5.7792557230	-2.1230017553
C	2.0791336848	4.9756325750	0.6595892992
C	9.5897683806	0.9060428385	1.8986025593
H	8.1541705974	2.1194866313	0.8823848830
C	8.9503969563	-1.3662915398	2.4022622523
C	2.4090372342	-4.4465216467	-0.8144560479
C	3.7885565282	-2.6615706333	-1.6438078879
C	2.8723665305	-2.4833322162	3.4196439088
C	2.2927257229	-4.7173535083	2.7273577479
C	5.9290548375	-5.5376365382	0.0339329561
C	5.6522201576	-5.6057780527	2.4258528342
C	8.5672769358	1.1922044456	-2.8597142030
H	7.9170616698	-0.0287268602	-1.2394636769
C	7.3536278749	3.2346377382	-3.2625784823
C	0.2487527510	3.1124286825	-1.6419496276
C	2.1948421979	1.9344828606	-2.4337990873
C	3.3279592950	7.0368723941	-1.6602520070
C	2.5899183232	5.6487452121	-3.4860377219
C	2.6080887322	4.7117345515	1.9341418606
C	0.9537612392	5.8189448473	0.5763905416
C	9.8882873065	-0.3619903775	2.4502190657
H	10.3251576072	1.7026440296	1.9559443242
H	9.1594302418	-2.3427207805	2.8299775868
C	1.7678819408	-4.5360418914	-2.0478701917

H	2.0925201950	-5.1078525905	-0.0142332032
C	3.1430239510	-2.7402990931	-2.8803373636
H	4.5721330409	-1.9241190557	-1.5088301863
C	1.9170528418	-2.5540317908	4.4367363711
H	3.4374861563	-1.5664589240	3.3012652411
C	1.3385958419	-4.7911925106	3.7391387498
H	2.4525300919	-5.5841887227	2.0917773325
C	6.7704272798	-6.6478395493	0.0964856294
H	5.7045821227	-5.0918239516	-0.9311488522
C	6.4945581663	-6.7160304500	2.4947223663
H	5.2096716884	-5.2149781627	3.3378422212
C	8.3780388955	2.3820202859	-3.5991519300
H	9.3555992450	0.5024031776	-3.1449954176
H	7.1722946401	4.1395091830	-3.8357641190
C	-0.5682243885	2.1006670400	-2.1615345345
H	-0.2218572817	3.9586213274	-1.1516401594
C	1.3876594973	0.9150367387	-2.9405434547
H	3.2717107283	1.8416955354	-2.5252568949
C	3.4424949074	8.1225134340	-2.5273875530
H	3.5592815534	7.1706416915	-0.6070027281
C	2.7010668947	6.7322216080	-4.3581468373
H	2.2493901487	4.6898866977	-3.8665342108
C	2.0201233217	5.2489416484	3.0809095547
H	3.4697657336	4.0643185824	2.0352051188
C	0.3615615461	6.3515108415	1.7203279504
H	0.5372801855	6.0722761396	-0.3938531965
H	10.8531357965	-0.5347382056	2.9164599870
C	2.1250633643	-3.6729225166	-3.0848042709
H	0.9715758463	-5.2576287494	-2.2006787316
H	3.4398675098	-2.0729016501	-3.6848498501
C	1.1384166246	-3.7003704714	4.5896968800
H	1.7791398541	-1.7036612483	5.0980062826
H	0.7395719421	-5.6869585380	3.8684906233
C	7.0560878699	-7.2380105799	1.3287655906
H	7.2000194472	-7.0553448930	-0.8141529926
H	6.7085120472	-7.1760076721	3.4552132287
H	9.0284738315	2.6083746521	-4.4380699333
C	0.0020804686	0.9821982115	-2.7911478069
H	-1.6450303454	2.1860004393	-2.0968307786
H	1.8407487153	0.0530713513	-3.4205649712
C	3.1289554146	7.9709173270	-3.8793996093
H	3.7713968330	9.0863584746	-2.1499702084
H	2.4507990178	6.6113775331	-5.4081560571
C	0.8917172646	6.0619451154	2.9783655268
H	2.4415439589	5.0226567784	4.0556168385
H	-0.5094695191	6.9935918551	1.6306867044
H	1.6046024766	-3.7275411668	-4.0361940734
H	0.3733950818	-3.7539823252	5.3574982646
H	7.7095828787	-8.1040840465	1.3794550120
H	-0.6533959654	0.1947148171	-3.1444320262
H	3.2142234080	8.8163347975	-4.5558333519
H	0.4299779430	6.4716132816	3.8716070932
P	-3.0762213114	-0.0215385240	-0.8190035423
O	-3.8305697843	-1.2417743735	-0.0281608854
O	-4.2621487777	1.1267547335	-0.7673645771
C	-4.8519779253	-1.7935451560	-0.7927440083
C	-5.3409651206	1.1038397096	0.1013989374
C	-6.0595543736	-1.1199215847	-0.8209257172
C	-4.5515394529	-2.9626614341	-1.5339330686
C	-6.2527332637	0.0557804515	0.0759703143

C	-5.4653955217	2.2417638240	0.9465261337
C	-7.0601552783	-1.5904599890	-1.7342414682
C	-5.5491790632	-3.4383364745	-2.3642096207
Si	-2.8662907528	-3.8210749533	-1.4680392136
C	-7.3454790337	0.0957001225	1.0107546130
C	-6.5753937802	2.3064519959	1.7669986433
Si	-4.0803907485	3.5088921269	1.1623365848
C	-8.2834257521	-0.9042279697	-1.9571368523
C	-6.7975305591	-2.7753728283	-2.4977958965
H	-5.3756163195	-4.3207743258	-2.9757522345
C	-1.5491040229	-2.9553924991	-0.4405676545
C	-2.1921910396	-4.0038948318	-3.2110238736
C	-3.0549584252	-5.5176998868	-0.6667176789
C	-8.2463196478	-0.9889168646	1.1896075463
C	-7.5257114939	1.2564995769	1.8284235202
H	-6.7154029704	3.1617321516	2.4208581100
C	-2.6216620880	2.6267825835	1.9719071193
C	-4.7069623456	4.8103383993	2.3707981337
C	-3.4713511629	4.4305961001	-0.3626597891
C	-9.2112319745	-1.3841481197	-2.8532928421
H	-8.4764801700	0.0135358015	-1.4158439690
C	-7.7821649657	-3.2483662955	-3.4050234195
C	-0.4316360094	-2.3578887174	-1.0314502927
C	-1.6344200320	-2.9619894941	0.9645911518
C	-2.1578589698	-2.8862920094	-4.0665544254
C	-1.5967435592	-5.1980918182	-3.6484153932
C	-1.8937835688	-6.2016247728	-0.2561546759
C	-4.2920855076	-6.1472668865	-0.4642620063
C	-9.2902946462	-0.9054020020	2.0831917056
H	-8.1007794219	-1.8960232493	0.6179520614
C	-8.6201001653	1.3170023764	2.7304963801
C	-1.3070875796	3.0851774205	1.7810210856
C	-2.8097450458	1.4794028946	2.7596043870
C	-5.5221325634	5.8660374199	1.9255733308
C	-4.4095986406	4.7399773500	3.7414301942
C	-3.6079805312	3.9818936294	-1.6871510636
C	-2.7647744440	5.6303539632	-0.1479538725
C	-8.9676920058	-2.5741849453	-3.5777849723
H	-10.1372319346	-0.8397338669	-3.0107196902
H	-7.5714822322	-4.1514964587	-3.9710043334
C	0.5834245668	-1.7939379149	-0.2476920892
H	-0.3314641490	-2.3437593905	-2.1093851724
C	-0.6440948987	-2.3894230149	1.7539466644
H	-2.4953913209	-3.4069080146	1.4518332951
C	-1.5301211319	-2.9629163596	-5.3099753759
H	-2.6011899154	-1.9496727380	-3.7412832093
C	-0.9708485277	-5.2759503241	-4.8937622013
H	-1.6192893675	-6.0764775624	-3.0097693695
C	-1.9650921499	-7.4725805795	0.3114664082
H	-0.9230489916	-5.7269525580	-0.3754573908
C	-4.3705943667	-7.4157904498	0.1147664651
H	-5.2063835913	-5.6376056803	-0.7532620566
C	-9.4934422123	0.2626020699	2.8534353457
H	-9.9611513535	-1.7506747425	2.2025946757
H	-8.7423457087	2.2126948990	3.3330582089
C	-0.2154269195	2.4027893202	2.3343210778
H	-1.1175240356	3.9770368808	1.1953435279
C	-1.7281581555	0.7866556187	3.3071542883
H	-3.8131402596	1.0908148961	2.9078736411
C	-6.0271594771	6.8121412674	2.8162417307

H	-5.7556530587	5.9546495652	0.8678295199
C	-4.9113831268	5.6847591094	4.6375011898
H	-3.7745059781	3.9398199337	4.1106645723
C	-3.0373002421	4.6876127736	-2.7480961952
H	-4.1439646988	3.0628813846	-1.8895744497
C	-2.1889280130	6.3347973455	-1.2050059463
H	-2.6661236538	6.0214693386	0.8603278314
H	-9.7108152242	-2.9429277139	-4.2777481544
C	0.4770826596	-1.8084096930	1.1490072890
H	1.4736922575	-1.3933703803	-0.7183431140
H	-0.7278999408	-2.4147758103	2.8338446664
C	-0.9328781532	-4.1553719729	-5.7249197758
H	-1.5063247696	-2.0899427532	-5.9560369330
H	-0.5156509598	-6.2084375274	-5.2149115477
C	-3.2074195575	-8.0830858460	0.4975071384
H	-1.0560568607	-7.9840779530	0.6146748731
H	-5.3396001405	-7.8820812017	0.2671160046
H	-10.3248623314	0.3156217071	3.5491348041
C	-0.4229493566	1.2345156246	3.0875649246
H	0.7873016951	2.7906858037	2.2026141338
H	-1.9013605741	-0.1174638055	3.8819979518
C	-5.7222266447	6.7217658364	4.1758235657
H	-6.6544090016	7.6204642341	2.4518031420
H	-4.6673996973	5.6135564670	5.6934679921
C	-2.3169983155	5.8583161146	-2.5104952639
H	-3.1482950165	4.3140261513	-3.7615930031
H	-1.6459443323	7.2555128891	-1.0114721252
H	1.2652168120	-1.3994345088	1.7679353381
H	-0.4451355156	-4.2129207415	-6.6938558204
H	-3.2674064396	-9.0706803097	0.9450652135
H	0.4381421155	0.7054651023	3.4844131774
H	-6.1125003215	7.4590906701	4.8712002494
H	-1.8622834271	6.3984726969	-3.3353118488
C	-2.9286146235	-5.6872461400	3.2852119384
H	-3.4507596341	-5.4137825591	2.3592132050
H	-2.6185655520	-6.7368624370	3.1994263371
H	-3.6321225793	-5.6016887472	4.1174394740
O	-1.8392244960	-4.8209331361	3.5754890712
H	-1.2402777134	-4.8231104989	2.8172439081

CATb-MeOH
0 1

Mg	0.9174894099	1.1856007231	-0.3728477255
O	2.6446981626	0.6102572757	0.0612078671
O	-5.3894212357	2.7298724718	0.6982726726
O	2.8176336624	-0.8734140138	2.1910325344
O	-4.7599524838	0.3273951627	1.6598542680
P	-4.8616363746	1.3582729436	0.5659078317
O	-5.5353918499	0.7073144658	-0.8131302552
O	-3.2301609751	1.3623706740	-0.0109077862
C	-5.1103901228	-0.5495027692	-1.1563857320
C	-2.8578338340	1.5211512699	-1.3044506047
C	-3.9577515519	-0.6561882146	-1.9239537858
C	-5.8693973812	-1.6554571512	-0.6921394092
C	-3.2176799670	0.5901232381	-2.2873371214
C	-1.9486605194	2.6026825559	-1.5476269173
C	-3.4764983211	-1.9789489296	-2.2406667643
C	-5.3786823617	-2.9129048826	-0.9755712859
Si	-7.4896783778	-1.5342668244	0.2873964733
C	-2.8159786663	0.8608747284	-3.6378204151

C	-1.4455743951	2.7372081674	-2.8322376797
Si	-1.4544760112	3.7563828753	-0.1084493646
C	-2.3010135790	-2.2472347357	-2.9929900302
C	-4.1941691774	-3.1109546910	-1.7286701289
H	-5.8943436572	-3.7940558347	-0.6008128664
C	-8.0377023103	0.1759016975	0.8157431813
C	-7.2657749831	-2.6666370705	1.7719326597
C	-8.8563636189	-2.2272503566	-0.8176965718
C	-3.3041807030	0.1134465151	-4.7440765165
C	-1.8847278511	1.9172591440	-3.9020973315
H	-0.7203296532	3.5075301836	-3.0646838451
C	-0.6738085179	5.3364811264	-0.8047516807
C	-0.2124287659	2.7977953379	0.9981894966
C	-2.8127362598	4.3553111527	1.0196212645
C	-1.8477975816	-3.5310932709	-3.1996348187
H	-1.7457099950	-1.4274124426	-3.4175270762
C	-3.7081095553	-4.4232103943	-1.9639934816
C	-7.9213331495	0.6251038039	2.1393150841
C	-8.5836537974	1.0539750843	-0.1363472729
C	-6.2645795088	-2.3826492400	2.7207753271
C	-8.0540323694	-3.8129471830	1.9588846712
C	-10.1933000790	-2.0768956630	-0.4043325285
C	-8.6189339228	-2.9065909025	-2.0226228904
C	-2.8573014170	0.3610457087	-6.0217273362
H	-4.0434438630	-0.6572362242	-4.5641928692
C	-1.4262950975	2.1340104945	-5.2295864756
C	-0.9737998489	5.8435612583	-2.0826631380
C	0.1191846412	6.1485587819	0.0310005758
C	-0.6653800859	1.5767986734	1.5800741176
C	1.0119855604	3.3141852571	1.4931720389
C	-2.9761157957	3.8957739314	2.3340828958
C	-3.6809976249	5.3536536795	0.5491620352
C	-2.5551948935	-4.6380566800	-2.6806237882
H	-0.9341061328	-3.6909366297	-3.7639391727
H	-4.2719617065	-5.2582234479	-1.5576834831
C	-8.3163425104	1.9137613148	2.4958138703
H	-7.4992382856	-0.0273237005	2.8946850214
C	-8.9776637355	2.3411236221	0.2155515390
H	-8.6901980475	0.7306966217	-1.1680546528
C	-6.0701164803	-3.2164048515	3.8212466262
H	-5.6417266184	-1.5023162046	2.5897548676
C	-7.8542351576	-4.6523115292	3.0567694752
H	-8.8322769744	-4.0521988135	1.2398616661
C	-11.2468919670	-2.5972908245	-1.1539732624
H	-10.4111963917	-1.5347058600	0.5118838533
C	-9.6697287137	-3.4246878548	-2.7815120760
H	-7.6006864625	-3.0274084924	-2.3788116756
C	-1.8949950908	1.3688465708	-6.2708589314
H	-3.2520651663	-0.2192023560	-6.8498672118
H	-0.7132019007	2.9352974476	-5.4068394153
C	-0.4488612437	7.0569237623	-2.5308935361
H	-1.6453600248	5.3004692157	-2.7371064032
C	0.6552708857	7.3558159160	-0.4130807077
H	0.2923197125	5.8581374901	1.0610453499
C	0.0632545915	0.9155100683	2.5722622844
H	-1.6159272127	1.1600582505	1.2752912457
C	1.7242684622	2.6664912569	2.5020371705
H	1.4184168516	4.2273435220	1.0831227687
C	-3.9859266468	4.4036699749	3.1472388525
H	-2.3273740372	3.1209668773	2.7281548621

C	-4.6962324031	5.8560395309	1.3563548063
H	-3.5682637331	5.7401314297	-0.4602700797
H	-2.1954294837	-5.6474342037	-2.8521505895
C	-8.8395279093	2.7749295651	1.5345382451
H	-8.2005159665	2.2484040065	3.5224011472
H	-9.3814369551	3.0099894297	-0.5389089391
C	-6.8620120688	-4.3542065796	3.9904178187
H	-5.2973111546	-2.9787189649	4.5471343225
H	-8.4732380562	-5.5362866449	3.1831524244
C	-10.9861049327	-3.2744212562	-2.3467264877
H	-12.2702723395	-2.4697429211	-0.8127113323
H	-9.4604013991	-3.9427666759	-3.7132901592
H	-1.5474483124	1.5472052785	-7.2834399685
C	0.3863703051	7.8071201845	-1.7055498300
H	-0.7007748877	7.4171748856	-3.5238438672
H	1.2687271464	7.9520023685	0.2560126649
C	1.2539704105	1.4684557249	3.0442361065
H	-0.3251476969	-0.0236449586	2.9498870179
H	2.6598595737	3.0866362909	2.8460154855
C	-4.8507874826	5.3795994763	2.6574618688
H	-4.1067902737	4.0237984061	4.1570310212
H	-5.3709675281	6.6133944614	0.9689359922
H	-9.1329834179	3.7841281522	1.8087292378
H	-6.7069801657	-5.0045400928	4.8469134933
H	-11.8051746969	-3.6771115826	-2.9357433080
H	0.8028113079	8.7466854360	-2.0554431567
H	1.8346751163	0.9475522767	3.7946735506
H	-5.6476268225	5.7652368394	3.2863928594
P	3.4306608167	-0.3505346354	0.9452963137
O	3.9301905631	-1.5232293664	-0.0925964142
O	4.8758775499	0.3670042388	1.2791198531
C	4.5932229777	-2.5405932941	0.5904095474
C	6.0465363742	0.1642657375	0.5611847449
C	5.9095757228	-2.3144004857	0.9481216290
C	3.8448079807	-3.6919539519	0.9304012971
C	6.5997259098	-1.1022528751	0.4248658295
C	6.6493028781	1.3455118340	0.0444771995
C	6.5373274987	-3.2696244475	1.8138361056
C	4.4838523011	-4.6339709306	1.7149923294
Si	2.0566524225	-3.9792295751	0.3911775814
C	7.8173786613	-1.2252665109	-0.3332893810
C	7.8675336270	1.2187687281	-0.5940309446
Si	5.7312211576	2.9888799297	-0.0172735569
C	7.8317192842	-3.0756040920	2.3646381966
C	5.8099593888	-4.4474924212	2.1855914029
H	3.9562073286	-5.5325269846	2.0259599139
C	1.2491876278	-2.5194138170	-0.5124942782
C	1.0023984201	-4.3502446564	1.8921820556
C	2.0165954418	-5.3875667171	-0.8545656794
C	8.3873676156	-2.4816512805	-0.6751886083
C	8.4760695668	-0.0439924436	-0.8016560517
H	8.3621208344	2.0990913331	-0.9934976241
C	4.2717163546	2.7368766386	-1.1951015370
C	6.8713537055	4.2654619920	-0.7961049771
C	5.1852773319	3.6648020660	1.6462411129
C	8.3899532550	-4.0115524420	3.2052522231
H	8.3759616425	-2.1714375586	2.1215881066
C	6.4210776968	-5.3978063873	3.0456360788
C	0.1665062671	-1.8365695803	0.0491591522
C	1.6703611876	-2.1473159752	-1.8052880786

C	1.0583288599	-3.4930401577	3.0086381784
C	0.0692929985	-5.3996443164	1.8971666911
C	0.8379198054	-5.6129030639	-1.5913867243
C	3.1157250537	-6.2262891258	-1.0958137023
C	9.5636075226	-2.5583178963	-1.3865151830
H	7.8824542604	-3.3904150322	-0.3758662767
C	9.6949397069	-0.1560907973	-1.5206424897
C	3.1096092349	3.5148321776	-1.1233255640
C	4.3228976648	1.7285352132	-2.1745373235
C	7.7233929785	5.0470200482	0.0031054162
C	6.9344455521	4.4336202253	-2.1897775498
C	5.3533634411	2.9586247429	2.8484989496
C	4.6227161016	4.9538696663	1.7129101537
C	7.6860812540	-5.1911194902	3.5424862271
H	9.3786832991	-3.8396553966	3.6192690894
H	5.8599670256	-6.2887428916	3.3129646299
C	-0.5007866655	-0.8236793622	-0.6552921905
H	-0.1975864477	-2.0921606273	1.0347825681
C	1.0683959650	-1.1001792027	-2.4879636673
H	2.4960620956	-2.6758880447	-2.2705522021
C	0.1943178471	-3.6794278769	4.0870337913
H	1.7640241797	-2.6670468949	3.0175374160
C	-0.7909115677	-5.5875598981	2.9800052455
H	0.0093759470	-6.0721616554	1.0464185967
C	0.7604179360	-6.6449520190	-2.5247123147
H	-0.0270235939	-4.9728503824	-1.4416313751
C	3.0423411789	-7.2590387685	-2.0317261249
H	4.0424306746	-6.0666933363	-0.5531315104
C	10.2372837278	-1.3871512464	-1.8027722547
H	9.9747193404	-3.5315341325	-1.6364465918
H	10.1818588702	0.7555562487	-1.8553191641
C	2.0127554347	3.2717619931	-1.9608726689
H	3.0385198983	4.3181237172	-0.3997147923
C	3.2487691937	1.4867018501	-3.0271476056
H	5.2045552113	1.0993264187	-2.2487817842
C	8.6114683273	5.9577626343	-0.5683297331
H	7.6852673406	4.9476146164	1.0842961002
C	7.8207928784	5.3429891864	-2.7664042260
H	6.2791249112	3.8507808369	-2.8312016963
C	4.9616832452	3.5123068917	4.0687247202
H	5.7759952716	1.9615917625	2.8315937974
C	4.2108136783	5.5028276195	2.9269308214
H	4.5231746465	5.5487495457	0.8085527735
H	8.1412940913	-5.9220633906	4.2031328873
C	-0.0260696722	-0.4286001565	-1.9172451034
H	-1.4163199403	-0.4273275771	-0.2298881595
H	1.4147619214	-0.8171395249	-3.4756055159
C	-0.7316134779	-4.7245016919	4.0741111677
H	0.2397490087	-3.0050829750	4.9371418064
H	-1.5101452028	-6.4010404629	2.9664295443
C	1.8639122572	-7.4710332925	-2.7467127122
H	-0.1570490660	-6.8036103207	-3.0838214032
H	3.9054946016	-7.8951417705	-2.2047504916
H	11.1675698173	-1.4644176427	-2.3564080640
C	2.0784740471	2.2440080130	-2.9144035621
H	1.1457887483	3.9216479832	-1.9044551348
H	3.3159710201	0.6975585901	-3.7685285930
C	8.6617603892	6.1060777269	-1.9551905698
H	9.2610606434	6.5531492359	0.0664256247
H	7.8526779074	5.4599444566	-3.8456556358

C	4.3808018847	4.7805389187	4.1098713805
H	5.1016882894	2.9482103269	4.9858040230
H	3.7703509173	6.4950773182	2.9527330889
H	-0.5645078098	0.3143621514	-2.4933287101
H	-1.4080392018	-4.8628300575	4.9123023575
H	1.8058456025	-8.2732439336	-3.4763807404
H	1.2403266260	2.0637266250	-3.5807074062
H	9.3508845488	6.8166344151	-2.4018462585
H	4.0655872926	5.2060283369	5.0576608441
O	-2.4213853577	-1.0571011398	1.8069710158
H	-3.2465488332	-0.5128698725	1.7869989048
C	-2.8243242955	-2.4135587521	1.9024528233
H	-3.5756810349	-2.6730904269	1.1475402776
H	-1.9484298780	-3.0497926393	1.7501703429
H	-3.2395440347	-2.6550274361	2.8908222078

Unsubstituted Model

CY1
0 1

O	0.0446115596	0.5527046608	0.9338260313
C	-0.9578399103	-0.0410394269	0.5895812356
C	-2.5925680154	-1.2825521165	-0.6701385263
C	-2.0295564588	-0.5062638119	1.5350322730
C	-2.6360775559	-1.7052446628	0.8048460470
H	-2.6475813355	-2.1339380315	-1.3626896713
H	-3.4562668899	-0.6326132676	-0.8885626406
H	-1.6191664279	-0.6937172885	2.5353969962
H	-2.7655107201	0.3147414971	1.6186345189
C	-1.2726893356	-0.4852527091	-0.8425710905
C	-1.5200599667	0.7127507710	-1.7753545805
H	-2.3512100226	1.3261663231	-1.4012153553
H	-1.8009873635	0.3286756046	-2.7659889317
Mg	2.0892998865	0.6968466717	0.7889819695
O	3.2244853882	-0.4846160375	-0.4925052010
O	2.1333971165	-1.3134427921	1.5635188743
P	3.0749900445	-1.6873422063	0.4315735196
O	2.5046511021	-2.9907507104	-0.3302186446
O	4.5303004657	-2.1473973570	0.9338927396
C	4.6444733685	-3.0971078046	1.9796300917
H	4.2529378041	-4.0787252973	1.6662515868
H	5.7112713547	-3.1932295195	2.2161779048
H	4.0980145107	-2.7611357710	2.8738207485
C	3.1249989794	-3.4144005907	-1.5328558605
H	2.5645788036	-4.2831276446	-1.9003993040
H	3.1029348899	-2.6132612934	-2.2876574261
H	4.1720762847	-3.7070349925	-1.3545387022
O	3.0029330911	1.8941355278	2.2892940643
C	2.9768096562	2.8693411214	1.5077195865
C	3.5206475739	4.2579051858	1.7538633014
N	2.4233590512	2.7247375508	0.3010247095
C	3.2193525304	4.9624351481	0.4296637433
H	4.5895914505	4.1915763976	2.0025209712
H	3.0144541221	4.6971530493	2.6259035284
C	2.5051148147	3.8981521147	-0.4229100829
Br	-0.0270173622	1.9212432585	-2.0234775954
H	2.5625903751	5.8382729335	0.5296056309
H	4.1193661551	5.2968542387	-0.1060406544
O	2.0864808870	4.0742142215	-1.5411657709
C	-0.1333139546	-1.3793466184	-1.3479323934

H	-0.3878488632	-1.7632224017	-2.3472211205
H	0.8110988993	-0.8228821559	-1.4310345682
H	0.0410822949	-2.2341214495	-0.6792780685
H	-2.0077675731	-2.5945438130	0.9695855434
H	-3.6501205051	-1.9510065057	1.1467601030

EC1
0 1

O	-0.7402626821	-0.4961827432	1.4349349416
C	-2.0003602637	-0.2935271753	0.9604826101
C	-3.1119497957	-1.2667742546	1.4890166028
C	-2.8156352028	0.8620447432	1.6093312018
C	-4.0483143193	-0.0590564019	1.6836055788
H	-2.7680000937	-1.6864885641	2.4452342019
H	-3.4456435380	-2.0851821714	0.8321419272
H	-2.3927547140	1.0455999481	2.6072772695
H	-2.8960962695	1.8257948983	1.0825300381
C	-2.0187686956	-0.3125649942	-0.5713598709
C	-2.6849513700	0.5706158209	-1.3402883771
H	-3.2845121039	1.3786039551	-0.9147830557
H	-2.7057818540	0.4631785036	-2.4290924598
Mg	1.0105095445	0.0431512861	1.4154034795
O	2.4977019439	-0.1712216394	-0.1102178323
O	2.6799518891	-0.8386196872	2.2686112126
P	3.4301817318	-0.7448060405	0.9476239152
O	4.0396610176	-2.1756344442	0.5662446032
O	4.7267900998	0.2203816485	1.0512112812
C	5.6765786787	-0.0140540490	2.0808917426
H	6.1629045506	-0.9935778558	1.9501200905
H	6.4341717998	0.7765427770	2.0124234426
H	5.1952927892	0.0181475772	3.0699791110
C	4.6067882390	-2.3868204009	-0.7173522473
H	4.8808132106	-3.4468406944	-0.7787081835
H	3.8811355478	-2.1469356615	-1.5088577221
H	5.5089971391	-1.7694593901	-0.8557137231
O	1.6016909801	2.0267066303	1.5546052300
C	2.0589877386	2.6933232201	0.6338921090
C	3.3976374156	3.3784946658	0.6151925656
N	1.4446514102	2.8933705909	-0.5623659412
C	3.4518223417	4.1002742867	-0.7315882089
H	4.1426613020	2.5719829927	0.7102209282
H	3.4934518886	4.0292493171	1.4949041724
C	2.1948912946	3.6742960543	-1.4722722629
Br	-0.1561885533	2.0356781704	-0.9738581788
H	3.4380631085	5.1961737535	-0.6407910513
H	4.3249052981	3.8353490311	-1.3428793754
O	1.8567744019	3.9292719591	-2.5865383764
C	-1.2314512847	-1.4363759582	-1.1869397873
H	-0.1514047035	-1.2829776538	-1.0372780554
H	-1.4798055839	-2.3908843333	-0.6983478736
H	-1.4227494196	-1.5239456164	-2.2653990168
H	-4.6425510157	-0.0287941634	2.6079799285
H	-4.7274001383	0.0809523428	0.8294090260

EP1
0 1

O	0.0441686384	0.4705090044	0.8052623417
C	-1.1285205353	-0.3435196859	0.8151989752
C	-1.1475243101	-1.8605311640	0.8010809982
C	-2.1790524229	-0.2864596543	1.9095535080

C	-2.4822297851	-1.7710738368	1.5840941629
H	-0.3246365767	-2.2697046287	1.4025305481
H	-1.1518076620	-2.3526328522	-0.1811799058
H	-1.7054051678	-0.1615367299	2.8939260866
H	-2.9917735730	0.4441140306	1.7937159872
C	-0.8940379605	0.5884322925	-0.2978724232
C	-1.5953357253	1.9242397323	-0.2985409808
H	-1.0454912220	2.6441795992	-0.9191490611
H	-1.7111006342	2.3069591036	0.7214217170
Mg	2.0821678295	0.3101942372	0.6565635154
O	2.6901339133	-1.2409823211	-0.5810304729
O	2.1931966042	-1.4427129812	1.8308218449
P	2.6769079950	-2.2020486861	0.6029056989
O	1.7160319497	-3.4714804692	0.3589910358
O	4.1355510921	-2.8448907019	0.7816102095
C	4.4342061519	-3.6066131691	1.9406412285
H	3.8331142416	-4.5297616036	1.9715225465
H	5.4978591900	-3.8690612474	1.8901761525
H	4.2417932339	-3.0213123111	2.8522941817
C	1.8696779453	-4.2549547691	-0.8129377781
H	1.0874468581	-5.0237651444	-0.7943800347
H	1.7570883742	-3.6357006242	-1.7160839605
H	2.8575878162	-4.7418167879	-0.8350695963
O	3.5014605008	1.6985564168	1.3758715633
C	3.1386343917	2.5291257645	0.5160660085
C	3.6920670387	3.9163169235	0.2896468307
N	2.1433821744	2.2132803330	-0.3201045741
C	2.8470600165	4.4140967625	-0.8846762264
H	4.7698695522	3.8505250322	0.0833147240
H	3.5770407401	4.5070733348	1.2101557433
C	1.8545579413	3.2722184860	-1.1526724518
Br	-3.3824984929	1.7523068360	-1.0497618595
H	2.2879648737	5.3371994065	-0.6774095354
H	3.4301857414	4.5923404732	-1.7999123609
O	0.9538985341	3.3101048213	-1.9609201632
C	-0.3842000913	0.0987701975	-1.6261544780
H	0.2219380532	0.8875287638	-2.0952000297
H	0.2300626727	-0.8040796495	-1.5072545592
H	-1.2348473686	-0.1274119204	-2.2850292794
H	-2.5935756734	-2.4483313964	2.4403779783
H	-3.3574272230	-1.8744169370	0.9279913747

TS1a
0 1

O	0.2893424870	-0.3592614767	1.5686389278
C	1.5936617061	-0.0630693240	1.3079029639
C	2.3659538441	0.9542647616	2.1819100752
C	2.7049479128	-1.0926698175	1.6063501713
C	3.6232891936	0.0733456596	2.0241376846
H	1.9553820780	0.9129022570	3.2012621624
H	2.4077745506	2.0043448431	1.8554588012
H	2.3868023716	-1.6878749765	2.4739056770
H	3.0316749436	-1.7822870288	0.8147007544
C	1.3283291017	0.3283441382	-0.1152440817
C	1.6224053193	-0.5204683858	-1.2155255721
H	2.5331158903	-1.1198630970	-1.1243122995
H	1.4711013107	-0.0716259687	-2.2023318265
Mg	-1.4735432890	-0.9026253908	1.1206636542
O	-2.3904845287	0.3903057659	-0.2701576866
O	-2.8714038275	0.2479332986	2.1541441251

P	-3.2149438018	0.9823204358	0.8676546406
O	-2.9429168162	2.5575745691	1.0694573527
O	-4.7750708682	0.9027167712	0.4906000098
C	-5.7480169215	1.1895704713	1.4815071366
H	-5.7030300079	2.2480650437	1.7854188601
H	-6.7311271630	0.9821777955	1.0412101094
H	-5.5996056887	0.5564479580	2.3692686468
C	-3.1204212157	3.4452954644	-0.0184857264
H	-2.8747798125	4.4531932087	0.3387603839
H	-2.4554508433	3.1823819180	-0.8563076761
H	-4.1621553986	3.4317432603	-0.3775480047
O	-2.2034685589	-2.7412665994	0.9957540031
C	-2.2074154412	-3.6264709655	0.1153133258
C	-3.1458098431	-4.8155187682	0.1504603956
N	-1.4128176973	-3.6578414476	-0.9483412883
C	-2.7907635766	-5.5841305014	-1.1195729500
H	-4.1816234270	-4.4483698136	0.1864642632
H	-2.9715979828	-5.3752298145	1.0811719441
C	-1.6503457216	-4.7767767008	-1.7491321865
Br	0.1335101228	-2.0832552145	-1.1651324641
H	-2.4410342731	-6.6103689157	-0.9396935301
H	-3.6157747575	-5.6464011459	-1.8434672836
O	-1.0558795184	-5.0489812851	-2.7550236816
C	0.5880055992	1.5966282765	-0.3542607378
H	-0.3471601304	1.3868697193	-0.8998133120
H	0.3362434652	2.1155816292	0.5776436193
H	1.2121823574	2.2452123847	-0.9915847866
H	4.2655755551	0.4128635178	1.1972041279
H	4.2515778317	-0.0731077596	2.9129063984

TS1b

0 1

O	-0.1238087984	-1.9919445122	-1.2376164380
C	0.9979617128	-2.4964451731	-0.7615977359
C	1.1803462858	-4.1841857844	-0.7236988090
C	2.2478864506	-2.5123833991	-1.6712615045
C	2.5268783422	-4.0057195627	-1.4252063166
H	0.3498217661	-4.4866408124	-1.3755263621
H	1.1430306366	-4.7690881390	0.2071278238
H	1.9041893924	-2.3147928620	-2.6937298258
H	3.0360321522	-1.7864731336	-1.4238240884
C	1.1789561534	-2.4330439044	0.6798095729
C	2.4720841413	-2.1677384603	1.2973727250
H	3.3598370038	-2.4611939293	0.7310689117
H	2.5492734933	-2.4022571006	2.3638941772
Mg	-0.8034886542	-0.2033428339	-1.0356042242
O	-1.8080464381	0.0168492960	0.8168409433
O	-2.8128030293	0.0559189244	-1.4450372038
P	-3.1129245920	0.1696396615	0.0438467457
O	-4.2104065434	-0.9348232983	0.4462571811
O	-3.7995428013	1.5666958815	0.4396313901
C	-4.9154295813	2.0333908192	-0.3006073177
H	-5.7832181118	1.3665584971	-0.1705585664
H	-5.1652915610	3.0302021366	0.0830165411
H	-4.6731560227	2.1000011363	-1.3720039844
C	-4.5786563768	-1.0961583925	1.8039664616
H	-5.3123007324	-1.9106186389	1.8482467153
H	-3.7043635072	-1.3563875049	2.4208544004
H	-5.0333933005	-0.1757333323	2.2044641265
O	0.2612645096	1.3735011489	-1.3820516747

C	0.9488717434	2.2909703059	-0.8554180338
C	0.9058054317	3.7085961550	-1.4017244929
N	1.7592228303	2.1534150959	0.1735113810
C	1.8479578916	4.4591819468	-0.4696240902
H	-0.1340606718	4.0667863801	-1.3882033530
H	1.2258416189	3.6950987792	-2.4544345440
C	2.3690430865	3.3674965815	0.4776450385
Br	2.2434740234	-0.1701213424	1.0108244242
H	2.7010804468	4.9346450389	-0.9738974778
H	1.3523299117	5.2373273378	0.1287701565
O	3.1823918391	3.5353126540	1.3492813700
C	-0.0388314659	-2.5183631891	1.5264396741
H	-0.5192021978	-1.5236092924	1.5864155304
H	-0.7875278561	-3.1607798478	1.0444420082
H	0.1927202160	-2.8662257362	2.5414879131
H	3.3817533158	-4.1968442450	-0.7606702036
H	2.6623748468	-4.6184093498	-2.3266059643

TS1c
0 1

O	-1.2630736309	1.2063246278	1.4524209019
C	-1.9214903129	1.8039551615	0.4610498993
C	-3.0274852503	1.0040828572	-0.2928958692
C	-3.1314455328	2.8308403485	0.9104807528
C	-4.0960833332	2.0761379747	-0.0106111457
H	-3.2107860822	0.0684337226	0.2482842048
H	-2.8167025573	0.7563135685	-1.3421881623
H	-3.2454294555	2.6066144335	1.9784460400
H	-3.0227949218	3.9177181129	0.7762320511
C	-1.1405490651	2.7791033833	-0.3266690259
C	-0.0282667438	3.4061759113	0.3163788276
H	-0.0491968549	3.3550165610	1.4085360160
H	0.3169472951	4.3530933551	-0.1097376261
Mg	-0.1777075717	-0.3557400461	1.1259609366
O	-0.6595201185	-1.0985671125	-0.8000527680
O	-1.2443930234	-2.1357154510	1.3768849543
P	-1.3279072285	-2.2927539256	-0.1337721239
O	-2.8771497694	-2.4537273221	-0.5566858090
O	-0.6441972825	-3.6486373478	-0.6641296291
C	-0.9481700429	-4.8777091145	-0.0269023214
H	-2.0071743123	-5.1472818542	-0.1713144983
H	-0.3148712842	-5.6473647162	-0.4853872406
H	-0.7383795817	-4.8210630108	1.0519258143
C	-3.2196236095	-2.5147196967	-1.9283429639
H	-4.3138253225	-2.5680150485	-1.9907743252
H	-2.8642259069	-1.6199578606	-2.4634389253
H	-2.7849223916	-3.4074444225	-2.4063760878
O	1.7035366972	-0.8610472858	1.4405925686
C	2.7455749365	-0.7443316690	0.7506434126
C	3.9017238871	-1.7191757225	0.8698652871
N	2.9739583310	0.2082029738	-0.1401671577
C	4.8992012458	-1.1980636497	-0.1591506790
H	3.5306467882	-2.7355450235	0.6737886981
H	4.2721136714	-1.7066099946	1.9057367984
C	4.2303213950	0.0634280418	-0.7235169367
Br	1.3839998987	1.9554154247	-0.1529733643
H	5.8789754933	-0.9265134903	0.2582435286
H	5.0834311619	-1.8968500249	-0.9878839934
O	4.7103977587	0.8081739319	-1.5362391632
C	-1.4806400840	3.1088269935	-1.7336637386

H	-1.2097391456	2.2382809880	-2.3551431639
H	-2.5634101074	3.2488193029	-1.8570233963
H	-0.9471027752	3.9935379199	-2.1016058417
H	-4.4035456752	2.6538415848	-0.8947947892
H	-5.0095805863	1.7143006103	0.4802960533

TS1d
0 1

C	0.0582300124	2.7834202524	0.5573933060
C	0.3065787534	4.0702992178	1.4267258087
C	-1.1672450123	4.2037517358	1.8411485951
H	0.7531956407	4.9268026905	0.9023383714
H	0.9702226632	3.7624604459	2.2422972220
C	-0.1413426146	3.0041362949	-0.8756299384
C	0.1856524562	1.9450254284	-1.8084385381
H	1.1091188393	1.4003817392	-1.5577395115
H	0.1143710729	2.2296276107	-2.8635580433
Mg	0.6638914583	-0.2135340401	0.7875137553
O	2.4155637622	-1.0539335015	1.5313773308
O	1.9024128558	-0.6933004191	-0.8658194620
P	2.9544129659	-1.1956411199	0.1155346732
O	4.3209499855	-0.3786086524	-0.1217269950
O	3.3685232748	-2.7276743693	-0.1420109728
C	3.7311198814	-3.1445745436	-1.4463615570
H	4.6516739342	-2.6394806224	-1.7817934492
H	3.9079979506	-4.2265087690	-1.4050372867
H	2.9243837049	-2.9312455024	-2.1642257977
C	5.3908770532	-0.5342043116	0.7940945972
H	6.1840235750	0.1604963938	0.4904823234
H	5.0663399820	-0.2974114505	1.8187969441
H	5.7835878070	-1.5638683807	0.7726044509
O	-0.8883176150	-1.3044856921	1.2759526429
C	-1.9714413778	-1.7199007487	0.7847412909
C	-2.7818291519	-2.8036219562	1.4758559575
N	-2.5257432000	-1.2923803263	-0.3343899909
C	-3.9705498977	-2.9866439867	0.5405679483
H	-3.0477521510	-2.4620095882	2.4873334928
H	-2.1525006611	-3.6974889180	1.5971252102
C	-3.7236225059	-1.9578737319	-0.5747508581
Br	-1.2383390935	0.5582364210	-1.4034639190
H	-4.0316559175	-3.9875497871	0.0894427707
H	-4.9458651802	-2.7832491143	1.0051527161
O	-4.4563321174	-1.7585366916	-1.5088498506
C	-0.7328151661	4.2485808368	-1.4268179267
H	-1.5311630715	4.0019274964	-2.1441553611
H	-1.1131542454	4.9458518808	-0.6735332343
H	0.0598608747	4.7550863219	-2.0051746780
H	-1.3525642918	4.1790643883	2.9231253773
H	-1.6722636255	5.0929075392	1.4369448446
O	0.6965084699	1.6939656294	0.9265156896
C	-1.5185728120	2.9090271817	1.1061022999
H	-1.6206598826	2.0201147861	1.7392636938
H	-2.3353693830	2.9306409324	0.3740350580

Real model with achiral catalyst

(2S,4R)-CY2
0 1

O	0.7332191310	-0.5871603570	-0.4627994537
C	-0.4278912248	-0.5363009141	-0.8216872406

C	-1.0803804729	0.6505598126	-1.4706462951
C	-2.3162503686	-1.3523025886	-1.9337187817
C	-2.4856330309	0.1943756525	-1.9221901268
H	-0.4477028370	0.9029806257	-2.3388443613
H	-1.0212081463	1.5347085442	-0.8200373939
H	-1.8038870873	-1.6450993650	-2.8664951942
H	-3.2733468691	-1.8877682336	-1.9175370146
C	-2.7911705828	0.7319215421	-3.3276185917
H	-3.7345599240	0.3281201278	-3.7214945467
H	-2.8775991784	1.8284090493	-3.3087324621
H	-1.9898539500	0.4609468909	-4.0326747265
C	-3.6237839846	0.5818629848	-0.9656796359
C	-3.4305013907	1.3062395734	0.2122231811
C	-4.9251735639	0.1495161804	-1.2596033589
C	-4.4926384889	1.5632552672	1.0795712144
H	-2.4386187587	1.6628800590	0.4861443965
C	-5.9891677269	0.4110719128	-0.4026073484
H	-5.1178030803	-0.4215965530	-2.1715968793
C	-5.7755043261	1.1164070727	0.7807688348
H	-4.3075551726	2.1130653721	2.0047900629
H	-6.9901422668	0.0562957830	-0.6581359360
C	-1.3929737374	-1.7073609231	-0.7432658511
C	-0.7303204161	-3.0696999517	-0.9903989315
H	-0.1124652523	-3.0369197653	-1.8975188149
H	-1.5142497601	-3.8261143065	-1.1243254600
C	-2.0998080592	-1.6455782139	0.6215286003
C	-1.4701339168	-1.0917943975	1.7424094937
C	-3.3842244466	-2.1789887408	0.7690502587
C	-2.1235673394	-1.0607589116	2.9742054622
H	-0.4643659466	-0.6701798330	1.6827311490
C	-4.0383130341	-2.1376614891	1.9958225869
H	-3.8992126161	-2.6218311481	-0.0851818055
C	-3.4107176439	-1.5727151481	3.1051257221
H	-1.6082039936	-0.6357175022	3.8384595276
H	-5.0465817144	-2.5472687215	2.0833412642
Mg	2.2663187273	0.6986676796	-0.0371358558
O	1.3767280482	1.0799713437	1.8778902154
O	1.4115824452	2.5879538172	-0.0811705220
P	0.9841341964	2.4577262220	1.3785033495
O	-0.6050574738	2.7448902153	1.4566289936
O	1.6196575942	3.5869990576	2.3235723419
C	1.5783098748	4.9495248712	1.9304347998
H	0.5448343042	5.3321668987	1.9411423114
H	2.1802012326	5.5127737699	2.6537167340
H	1.9981382367	5.0776255233	0.9215397158
C	-1.2764613202	2.7138507804	2.7073694169
H	-2.1759800976	3.3360289203	2.6114735192
H	-1.5696619272	1.6840076065	2.9594102967
H	-0.6414868908	3.1207909319	3.5093756897
O	3.1624688980	0.3146276339	-1.9660696374
C	4.1945530633	-0.0433167760	-1.3638729554
C	5.4749431614	-0.5675940533	-1.9721841687
N	4.2319538342	0.0174702633	-0.0265094607
C	6.3384273745	-0.8337505107	-0.7388231443
H	5.8845416039	0.1867100560	-2.6597013136
H	5.2514825414	-1.4615195292	-2.5724694468
C	5.4566901367	-0.4245866897	0.4563307170
Br	0.4143913113	-3.7378658771	0.4229083611
H	6.6252233062	-1.8882695593	-0.6190429816
H	7.2663527842	-0.2449759484	-0.7113395447

O	5.7861679121	-0.4823274029	1.6109543239
H	-6.6039801779	1.3152147581	1.4637101570
H	-3.9227165221	-1.5399613892	4.0690975442

(2S,4R)-CY3
0 1

O	0.8853328265	-0.6398495459	-0.4908719827
C	-0.2860559548	-0.6100249873	-0.8200283165
C	-0.9924106303	0.5882074880	-1.3864669778
C	-2.1810898664	-1.4239609386	-1.9219242056
C	-2.3964647435	0.1141565223	-1.8218825586
H	-0.3929805287	0.9034844442	-2.2578355486
H	-0.9388759136	1.4402434160	-0.6941781637
H	-1.6893704539	-1.6489939026	-2.8841638766
H	-3.1206715843	-1.9899331419	-1.9084316450
C	-2.7574250330	0.7148274803	-3.1883532809
H	-3.6982939192	0.3022566040	-3.5792220098
H	-2.8766196712	1.8055205947	-3.1089366393
H	-1.9678359609	0.5071242263	-3.9272554048
C	-3.5175498196	0.4214448915	-0.8161802298
C	-3.3095608968	1.0896174367	0.3924577809
C	-4.8160338480	-0.0276942929	-1.0975569285
C	-4.3534858364	1.2767237836	1.2989615227
H	-2.3194100593	1.4559348710	0.6600369127
C	-5.8626530048	0.1647687248	-0.2012370850
H	-5.0200203033	-0.5573860844	-2.0318011307
C	-5.6339392761	0.8154707696	1.0102315673
H	-4.1553057286	1.7825992689	2.2463358510
H	-6.8618744688	-0.2000353117	-0.4497226393
C	-1.2115813466	-1.8140738794	-0.7793388733
C	-0.5178806695	-3.1403551200	-1.1168702872
H	0.0752898908	-3.0434313937	-2.0359558853
H	-1.2835402177	-3.9116609225	-1.2705075331
C	-1.8806904563	-1.8278361286	0.6036907433
C	-1.2347705599	-1.3168443501	1.7399043153
C	-3.1589871644	-2.3602808824	0.7657257377
C	-1.8658663801	-1.3107253876	2.9752624153
H	-0.2270515544	-0.8996656010	1.6829251854
C	-3.8076898894	-2.3528753870	1.9974069942
H	-3.6953863907	-2.7781908219	-0.0877685080
C	-3.1662271830	-1.8081361946	3.1146428776
H	-1.3586843026	-0.9196584059	3.8588560203
H	-4.8157381408	-2.7605687471	2.0652664354
Mg	2.3663193100	0.7107627247	-0.0854699097
O	1.5773594886	0.9881447374	1.8847635084
O	1.4228024421	2.5609083215	-0.0150654471
P	1.0851731001	2.3589132426	1.4596178901
O	-0.5095265943	2.5578767856	1.6324681302
O	1.7131597453	3.4874279368	2.4107829846
C	1.5726172355	4.8583908455	2.0746738470
H	0.5214476957	5.1792138862	2.1567553708
H	2.1817154249	5.4295368343	2.7856567389
H	1.9265231223	5.0474482647	1.0501521173
C	-1.1126724796	2.4466902550	2.9134592384
H	-2.0391808908	3.0354371732	2.8911881353
H	-1.3523780218	1.3962954859	3.1334878933
H	-0.4527071115	2.8449787111	3.6994306118
O	3.1433365526	0.5098207008	-2.0996632822
C	4.2264506860	0.1571882974	-1.5926967212
C	5.4856357201	-0.2580337986	-2.3187283083

N	4.3505055545	0.1192376994	-0.2591032998
C	6.4406761282	-0.5709770877	-1.1666003589
H	5.8145204987	0.5634768233	-2.9716747982
H	5.2622901422	-1.1158037390	-2.9697079793
C	5.6226561341	-0.2973338637	0.1097740682
Br	0.6804488699	-3.8460988491	0.2323005455
H	6.7835934189	-1.6152958506	-1.1472007118
H	7.3399271199	0.0611923839	-1.1518613540
O	6.0302385285	-0.4259567206	1.2334076602
H	-6.4490325509	0.9629181556	1.7220702467
O	-3.7150997738	-1.7234443626	4.3426297565
C	-5.0277381774	-2.1763490604	4.5328081942
H	-5.7443956418	-1.6271919216	3.8972081474
H	-5.1231770107	-3.2567406364	4.3270237477
H	-5.2739994156	-1.9947740992	5.5859712887

(2S,4R)-CY4
0 1

O	0.8622306163	-0.8010098850	0.0709588775
C	-0.1216616186	-0.9753506877	-0.6293125837
C	-0.3965905547	-0.2753830080	-1.9236382123
C	-1.9543716073	-2.0497562046	-1.6708522857
C	-1.8364495033	-0.6414541811	-2.3140233112
H	0.3252204908	-0.7128651670	-2.6384919108
H	-0.1396119444	0.7919538597	-1.8688935718
H	-1.4134301891	-2.7675984670	-2.3107701948
H	-2.9888206169	-2.4074089937	-1.6076994715
C	-1.9933079995	-0.6981821573	-3.8372930860
H	-2.9848451035	-1.0692138963	-4.1328462505
H	-1.8658605306	0.3035807620	-4.2730078411
H	-1.2390772270	-1.3655458240	-4.2826714931
C	-2.8829285310	0.2963512499	-1.6959443092
C	-2.5485119917	1.3303035354	-0.8174815987
C	-4.2429154706	0.0647510267	-1.9478238165
C	-3.5379791579	2.0786250115	-0.1793556868
H	-1.5109261697	1.5662981100	-0.5843158859
C	-5.2328105794	0.8172007159	-1.3233129009
H	-4.5441013480	-0.7366609263	-2.6277998941
C	-4.8835063329	1.8241293538	-0.4229354841
H	-3.2360625940	2.8608076888	0.5194474521
H	-6.2844983366	0.6092884630	-1.5333706306
C	-1.2298032191	-1.9799915264	-0.3015109600
C	-0.6954656036	-3.3839282192	0.0388619114
H	-0.0005430042	-3.7242929053	-0.7399845433
H	-1.5450525916	-4.0779434449	0.0771843981
C	-2.0897947584	-1.3984183388	0.8371028112
C	-1.5052721591	-0.6741311201	1.8841092706
C	-3.4744712696	-1.5847474940	0.8542530093
C	-2.2912595221	-0.1079596624	2.8791011004
H	-0.4269496044	-0.5270747514	1.9263581450
C	-4.2679547732	-0.9976395975	1.8333677372
H	-3.9681686994	-2.1616538935	0.0733832109
C	-3.6773055491	-0.2367942485	2.8380141141
H	-1.8169881260	0.4510241560	3.6864283896
H	-5.3508237955	-1.1206295955	1.8016695929
Mg	2.2711604181	0.6998224560	-0.0568500288
O	1.7774418243	1.7064041481	1.7191419369
O	0.9785542964	2.2910895229	-0.5399987913
P	0.9338736979	2.6961034020	0.9330670406
O	-0.6068861752	2.7391636695	1.4008566801

O	1.4623055136	4.1877547204	1.1871019164
C	0.9520774971	5.2605360270	0.4109110660
H	-0.1161495424	5.4287351150	0.6231265652
H	1.5208202520	6.1571225963	0.6850859237
H	1.0764267035	5.0585297079	-0.6636230040
C	-0.9324320182	3.0582646059	2.7451403450
H	-0.7300826319	4.1200070002	2.9562729830
H	-2.0024492911	2.8558406416	2.8763527013
H	-0.3513174154	2.4379436276	3.4449645364
O	2.8054781233	-0.0529950874	-2.0563096355
C	3.9376100056	-0.2947367901	-1.5921343493
C	5.1047601621	-0.9342188721	-2.3091393567
N	4.2116704086	0.0224265658	-0.3203723204
C	6.1859197710	-0.9511152869	-1.2281236013
H	5.3573374436	-0.3354217886	-3.1962543337
H	4.8077030252	-1.9302677533	-2.6687809013
C	5.5167180779	-0.3249292276	0.0092339472
Br	0.2405526248	-3.5970670175	1.7205770857
H	6.5366766505	-1.9596313238	-0.9669567510
H	7.0764484681	-0.3596188304	-1.4843460289
O	6.0432070650	-0.1673358547	1.0776543879
H	-5.6564195823	2.4039509283	0.0858077302
C	-4.5140186276	0.5100222577	3.8404183323
F	-5.7767154326	0.0801364796	3.8733436863
F	-4.5537759279	1.8226283997	3.5460904510
F	-4.0189790493	0.4113693633	5.0776074895

(2S,4S)-CY2
0 1

O	0.9660724224	-0.7346463463	-0.4679436671
C	-0.1157630791	-0.7662475797	-1.0249604238
C	-0.5185325257	0.1153065609	-2.1646722310
C	-2.2009473562	-1.5607546202	-1.8591937855
C	-2.0291996214	-0.1037764308	-2.3578359359
H	0.0547510880	-0.2569135707	-3.0344306526
H	-0.1901703762	1.1512468731	-1.9961169359
H	-1.9234766976	-2.2478198760	-2.6758426810
H	-3.2373277127	-1.7902424002	-1.5812047050
C	-2.8288027681	0.8803692952	-1.4841342056
H	-2.6373904268	1.9132381412	-1.8088266694
H	-3.9066881888	0.6801793155	-1.5755507224
H	-2.5535236136	0.8023795083	-0.4231944057
C	-2.4695844661	0.0780252816	-3.8051689053
C	-3.5388329797	-0.6537126094	-4.3350499197
C	-1.8558625968	1.0358058171	-4.6220670736
C	-3.9733241733	-0.4460350285	-5.6432216828
H	-4.0496868338	-1.3990731571	-3.7215656573
C	-2.2874592980	1.2459319044	-5.9303535944
H	-1.0280249206	1.6342168488	-4.2345778912
C	-3.3473974139	0.5037452354	-6.4477779216
H	-4.8071443449	-1.0326009090	-6.0353884591
H	-1.7893534312	1.9955666714	-6.5491019537
C	-1.2190162361	-1.7610830804	-0.6737297397
C	-0.6923124667	-3.2097378771	-0.7047385359
H	-0.1339741670	-3.3979072389	-1.6315959915
H	-1.5461566319	-3.8985061319	-0.6675821682
C	-1.8462397603	-1.4361423892	0.6911832076
C	-1.2387509411	-0.5915832865	1.6263491070
C	-3.0566684034	-2.0517140610	1.0340325203
C	-1.8411854262	-0.3600273019	2.8636622000

H	-0.2916931116	-0.0924373669	1.4171805421
C	-3.6541274821	-1.8225608967	2.2698012011
H	-3.5455150186	-2.7324905565	0.3326402735
C	-3.0487334057	-0.9690583869	3.1911749785
H	-1.3434198988	0.2997049470	3.5779142764
H	-4.5964723728	-2.3172579273	2.5144602609
Mg	2.5085930904	0.6103615208	-0.2722556875
O	1.4020403627	1.6150615531	1.2623619643
O	1.7042485281	2.3567219985	-1.0753744168
P	1.0460376407	2.6979280709	0.2596753524
O	-0.5428197039	2.8215138048	-0.0026715210
O	1.4634299119	4.1378340689	0.8248242633
C	1.4296679618	5.2721707206	-0.0270329735
H	0.3950311744	5.5174704735	-0.3165533876
H	1.8566867199	6.1112245306	0.5353150320
H	2.0246978400	5.0956648169	-0.9355387420
C	-1.4255021985	3.1076351023	1.0702146056
H	-2.4291737097	3.2221598818	0.6423419341
H	-1.4352825730	2.2839342778	1.7993896723
H	-1.1374436019	4.0402292941	1.5804822287
O	3.6400456790	-0.3143333476	-1.8579791620
C	4.5344294034	-0.5494925057	-1.0209836375
C	5.8327393285	-1.2882625286	-1.2494932561
N	4.3821171055	-0.1284074280	0.2416822939
C	6.4777629173	-1.2532925773	0.1363543642
H	6.4090530720	-0.7745586789	-2.0326600511
H	5.6129084447	-2.2988682080	-1.6232625756
C	5.4735437894	-0.4899891550	1.0205006094
Br	0.4728565149	-3.7218509750	0.7542184751
H	6.6439744016	-2.2487974526	0.5721208903
H	7.4427693818	-0.7273902869	0.1632250665
O	5.6215983758	-0.2524655052	2.1896081478
H	-3.6848170340	0.6656468990	-7.4736143064
H	-3.5137894763	-0.7876153256	4.1623325846

(2S,4S)-CY3

0 1

O	1.2024152176	-0.6889794397	-0.3519494433
C	0.2543374366	-0.7167935967	-1.1163903187
C	0.1265643314	0.1160232636	-2.3522952462
C	-1.6551927829	-1.4802861092	-2.3160382486
C	-1.3258050345	-0.0557091487	-2.8282649374
H	0.8419657649	-0.3243816053	-3.0722327383
H	0.4666995283	1.1457483090	-2.1666340979
H	-1.2513483170	-2.2135853980	-3.0338422057
H	-2.7347893149	-1.6612451467	-2.2431476990
C	-2.2304356835	0.9987721114	-2.1640941763
H	-1.9444617060	2.0062744716	-2.4983589771
H	-3.2818784624	0.8261890902	-2.4383043553
H	-2.1481805004	0.9734932966	-1.0688461248
C	-1.4709639106	0.0730927618	-4.3393857656
C	-2.4564530541	-0.6363041417	-5.0359397302
C	-0.6623384539	0.9602572676	-5.0607577102
C	-2.6214369774	-0.4755430116	-6.4107630082
H	-3.1134403236	-1.3268277797	-4.5025907676
C	-0.8242680142	1.1234145621	-6.4352387167
H	0.1071897384	1.5394136343	-4.5451218628
C	-1.8038311861	0.4040561760	-7.1172068663
H	-3.3948671799	-1.0435845524	-6.9326108284
H	-0.1778404270	1.8181207473	-6.9760413554

C	-0.9367455253	-1.6574366527	-0.9515511041
C	-0.4746127367	-3.1233078021	-0.8255377260
H	0.2438810848	-3.3735420958	-1.6177473593
H	-1.3491981278	-3.7786641673	-0.9290071013
C	-1.8030874488	-1.2474616174	0.2484041831
C	-1.3375057350	-0.4289409935	1.2884318706
C	-3.0983167528	-1.7562700410	0.3544551003
C	-2.1492985504	-0.1222212582	2.3708969776
H	-0.3304769013	-0.0100123402	1.2749541112
C	-3.9233567390	-1.4599640531	1.4384191808
H	-3.4951305583	-2.4168663515	-0.4203180002
C	-3.4505637931	-0.6297925214	2.4600447055
H	-1.7808076646	0.5096535385	3.1806909215
H	-4.9258562857	-1.8861959131	1.4725938411
Mg	2.7552614564	0.6284472506	-0.0377285514
O	1.5758859982	1.6804508620	1.3921170121
O	2.0078056335	2.3570584972	-0.9452816732
P	1.2509505546	2.7172072739	0.3314486385
O	-0.3215427452	2.7640325794	-0.0331010302
O	1.5768487837	4.1891678083	0.8725858171
C	1.5556971860	5.2920237759	-0.0193686422
H	0.5407406783	5.4625270539	-0.4138813489
H	1.8754058677	6.1736670174	0.5490353312
H	2.2439629671	5.1259597494	-0.8616198542
C	-1.2668473282	3.0914870910	0.9736825317
H	-2.2429886878	2.7143175061	0.6430609606
H	-1.0077782253	2.6144238006	1.9306321035
H	-1.3229323017	4.1821947535	1.1147046049
O	3.9426624557	-0.3230263124	-1.5776691649
C	4.7954577378	-0.5660264165	-0.7010938393
C	6.0946281491	-1.3196546250	-0.8698430531
N	4.5925675902	-0.1398002180	0.5531359491
C	6.6791603508	-1.2864684905	0.5425355678
H	6.7100410726	-0.8151447733	-1.6288623395
H	5.8805495149	-2.3293365345	-1.2494783487
C	5.6454490422	-0.5099208758	1.3798440994
Br	0.3634948348	-3.6110777831	0.8503254398
H	6.8161541587	-2.2819982917	0.9882739664
H	7.6473785493	-0.7701581696	0.6096449082
O	5.7449442735	-0.2697894388	2.5534686577
H	-1.9299561852	0.5290980651	-8.1946674894
O	-4.1626612100	-0.2738386838	3.5472113046
C	-5.4610661419	-0.7765956126	3.7069640476
H	-6.1286604956	-0.4566215036	2.8876485310
H	-5.4690425349	-1.8790597772	3.7639029493
H	-5.8415232544	-0.3707783512	4.6520119338

(2S,4S)-CY4
0 1

O	-1.6197501616	-0.8114392273	0.0502629686
C	-0.8228105379	-1.1789880455	0.8947678362
C	-0.8427027160	-0.7630281152	2.3308660815
C	0.7927667248	-2.4866238819	2.0548681328
C	0.4911941822	-1.2431327953	2.9274165579
H	-1.7033586325	-1.2963757928	2.7766444476
H	-1.0624316731	0.3120998179	2.4188363974
H	0.2123719794	-3.3388354607	2.4452451378
H	1.8495800505	-2.7774425406	2.0890957734
C	1.5707150496	-0.1597348711	2.7476695873
H	1.3195019348	0.7254987441	3.3489902377

H	2.5502414197	-0.5365539295	3.0777752643
H	1.6540874118	0.1670510152	1.7018314710
C	0.3893834885	-1.5794325194	4.4098956321
C	1.1875138549	-2.5757544049	4.9841330043
C	-0.4610073614	-0.8490346580	5.2493080590
C	1.1293946818	-2.8439421751	6.3508812709
H	1.8725824789	-3.1559725789	4.3621110071
C	-0.5217342695	-1.1144997310	6.6159389130
H	-1.0868573215	-0.0550196333	4.8353303544
C	0.2721803085	-2.1154184119	7.1727119134
H	1.7592202404	-3.6291526707	6.7749373227
H	-1.1963111687	-0.5339172093	7.2489529907
C	0.3088837745	-2.1685840483	0.6157156551
C	-0.2202475242	-3.4677764404	-0.0255897027
H	-1.0743992187	-3.8564227294	0.5446820322
H	0.5799502598	-4.2192287924	-0.0102294240
C	1.3917198149	-1.5305256725	-0.2687443244
C	1.1307851512	-0.4442841943	-1.1125817746
C	2.6744108713	-2.0897092777	-0.2818587523
C	2.1362109377	0.0810430880	-1.9175668467
H	0.1458033202	0.0199735844	-1.1575487700
C	3.6839047856	-1.5615820386	-1.0793178565
H	2.9054787426	-2.9584652193	0.3372646371
C	3.4182707292	-0.4628233916	-1.8934134544
H	1.9093351218	0.9230478894	-2.5741973135
H	4.6788938650	-2.0081181894	-1.0689317878
Mg	-2.9388539175	0.7778325380	-0.0286424766
O	-1.4464608845	1.9529423052	-0.9967328434
O	-2.0879806527	2.0577141715	1.3857418563
P	-1.1356044687	2.6204928137	0.3320342620
O	0.3723448996	2.3396145175	0.8349138098
O	-1.2165143157	4.2128002274	0.1861533978
C	-1.1965677692	5.0343104084	1.3431662046
H	-0.2353988167	4.9458836300	1.8752026232
H	-1.3291544247	6.0693567017	1.0063521620
H	-2.0121201642	4.7612231240	2.0292550403
C	1.4718194861	2.8330125653	0.0838319127
H	1.6945913845	3.8746437425	0.3617610556
H	2.3399061925	2.2008746306	0.3113881207
H	1.2714939132	2.7827078556	-0.9969337663
O	-4.4177765055	-0.3386243804	1.0903578365
C	-5.1808700389	-0.2097964414	0.1122230142
C	-6.5796894358	-0.7610636614	-0.0393094842
N	-4.7691250923	0.4835374202	-0.9580880218
C	-6.9755475200	-0.2738953844	-1.4335789791
H	-7.2112358078	-0.3746786599	0.7738389242
H	-6.5515861982	-1.8551232125	0.0687528484
C	-5.7516903629	0.5117088727	-1.9401977035
Br	-0.8014961143	-3.3393337638	-1.8673777124
H	-7.1900616148	-1.0865508726	-2.1421398597
H	-7.8494979692	0.3929568974	-1.4382619122
O	-5.6702835554	1.0552991545	-3.0089258039
H	0.2236775821	-2.3260248812	8.2430638637
C	4.5085459385	0.1795198612	-2.7109365011
F	5.5767725033	-0.6115687594	-2.8381001024
F	4.9258078443	1.3193870033	-2.1402326628
F	4.0872184707	0.4925428434	-3.9386717407

(2S,4S)-CY5
0 1

O	-0.3794638183	-0.1406105639	1.7507714701
C	-1.3335896929	-0.7839061942	1.3673401025
C	-2.5609539145	-2.3942328240	0.0816548469
C	-2.4684981124	-1.2776909139	2.2098104704
C	-3.4873166130	-1.9127944385	1.2436967705
H	-2.1271530811	-3.3616298748	0.3698196625
H	-3.1098418315	-2.5541564692	-0.8559461376
H	-2.0123514257	-2.0744338275	2.8260183392
H	-2.8395838273	-0.5299176280	2.9203662481
C	-4.1991403029	-3.0987271908	1.9105388046
H	-4.8189440702	-2.7538131615	2.7514428970
H	-4.8582637216	-3.6207183956	1.2024059398
H	-3.4698296912	-3.8293430459	2.2942393061
C	-4.5459382160	-0.9197202108	0.7405445959
C	-5.4409793752	-1.3338165694	-0.2577564547
C	-4.6728838862	0.3870060008	1.2234278324
C	-6.4136105257	-0.4766204831	-0.7616121972
H	-5.3768093950	-2.3480436898	-0.6602160497
C	-5.6456125763	1.2525383370	0.7187863928
H	-3.9994745727	0.7700199710	1.9906100725
C	-6.5191654749	0.8267651763	-0.2759072008
H	-7.0922813991	-0.8279037003	-1.5417521279
H	-5.7102529041	2.2707367566	1.1076116278
C	-1.4403645399	-1.3340042064	-0.0639348490
C	-1.9424915546	-0.2288997373	-1.0164625308
H	-2.9103263247	0.1530207952	-0.6789914090
H	-2.0849221538	-0.6534415100	-2.0180311994
Mg	1.2993056643	0.0854549218	0.3845070992
O	2.7790244490	-0.4651438966	-1.0459337972
O	2.6885975881	-1.1975371977	1.3243097224
P	3.5147504598	-1.2201876324	0.0471485682
O	3.7723456270	-2.7690687342	-0.3593286416
O	4.9939096084	-0.6254678217	0.2302463234
C	5.7592740253	-0.9781245971	1.3700142558
H	6.0064417118	-2.0524635273	1.3633880572
H	6.6872328309	-0.3945166324	1.3302299340
H	5.2132107612	-0.7409645636	2.2951193904
C	4.4769965234	-3.0601014078	-1.5534513421
H	4.4857551713	-4.1509949211	-1.6719630241
H	3.9855177632	-2.5982130215	-2.4244263020
H	5.5141004385	-2.6929195576	-1.4984360585
O	1.8947041672	1.8633304221	0.9459264607
C	1.8923057914	2.9632463691	0.3242495332
C	2.7405152508	4.1312222622	0.8027898149
N	1.1869894772	3.2193174075	-0.7565958306
C	2.4417255072	5.2004948385	-0.2389590855
H	3.7933512730	3.8158376357	0.8482105700
H	2.4412564631	4.3985546203	1.8275043709
C	1.4131697054	4.5243209651	-1.1669528134
Br	-0.7508502094	1.3023976264	-1.1549537698
H	2.0055366234	6.1233024795	0.1696978926
H	3.3165023472	5.4949904834	-0.8370857714
O	0.8880005700	5.0668214246	-2.1084371186
H	-7.2762035088	1.5052373087	-0.6737265190
N	-0.0807372121	-1.7817734017	-0.4247873461
C	0.3559443910	-2.9519977533	0.3460767536
H	0.2307733820	-2.7842272870	1.4246893825
H	-0.1879783955	-3.8687775733	0.0552345975
H	1.4248298475	-3.1202664629	0.1754815863
C	0.1372118752	-2.0358346427	-1.8505272796

H	0.0235929785	-1.1185846637	-2.4399029772
H	1.1774778754	-2.3578999937	-1.9763935703
H	-0.5372258210	-2.8215538775	-2.2410212890

EC2
0 1

O	1.1551415629	-0.4600184195	-0.6175353191
C	1.9685576686	-0.9709461449	0.3509686476
C	2.7650276986	-2.2626838849	-0.0650307196
C	3.3386080162	-0.2415792249	0.4642129439
C	4.1064021037	-1.5797780435	0.3045486529
H	2.6428275164	-2.4138292794	-1.1464722211
H	2.5415250497	-3.1988677642	0.4701446126
H	3.4371344494	0.3990838318	-0.4210249251
H	3.5603015201	0.3588849034	1.3594743983
C	4.6939354920	-2.1085538321	1.6178782177
H	5.0919654962	-3.1258222202	1.4778415374
H	5.5212366018	-1.4667185887	1.9601224811
H	3.9323572200	-2.1480710180	2.4102718500
C	5.1892573437	-1.6031777882	-0.7553239292
C	6.0379922883	-0.5058456580	-0.9388187593
C	5.4106843445	-2.7469742712	-1.5306158564
C	7.0723748044	-0.5457011871	-1.8724080398
H	5.8841528999	0.3975225594	-0.3424293182
C	6.4428844428	-2.7921618171	-2.4661978164
H	4.7610789460	-3.6169240636	-1.4015068307
C	7.2784042534	-1.6899182423	-2.6417648119
H	7.7203556490	0.3245420665	-2.0017050588
H	6.5939658613	-3.6940786563	-3.0641775440
C	1.1871415332	-1.1656312600	1.6594234757
C	1.6250728565	-0.7516828852	2.8621911062
H	2.5884233062	-0.2548680280	2.9825148227
H	1.0641813544	-0.9477048011	3.7789341515
C	-0.1522544188	-1.8224262834	1.5268673489
C	-0.4427596212	-2.6885181715	0.4554282500
C	-1.1778156650	-1.5716668773	2.4516936921
C	-1.6933785771	-3.2889611370	0.3322147499
H	0.3160688821	-2.8914246406	-0.2990524143
C	-2.4237661445	-2.1805133867	2.3366272717
H	-1.0121420177	-0.8653697541	3.2668704307
C	-2.6875933192	-3.0437098546	1.2764922603
H	-1.8921476849	-3.9462547631	-0.5162611616
H	-3.1993708845	-1.9606020483	3.0737645818
H	-3.6674953831	-3.5154327701	1.1772813739
Mg	-0.5618462741	0.1266241093	-0.9304153022
O	-2.5210623056	0.3883663373	-0.0525369095
O	-1.9800904290	-0.4842394949	-2.3088585808
P	-3.1107437787	-0.0870004200	-1.3704310176
O	-4.1554363330	-1.2941421612	-1.2299385458
O	-4.0044443990	1.1226149149	-1.9775785055
C	-4.5195502508	1.0025257640	-3.2951172917
H	-5.2370220681	0.1692779073	-3.3618648191
H	-5.0352044105	1.9416538636	-3.5315619603
H	-3.7068619707	0.8342877545	-4.0176661990
C	-5.2054492232	-1.2011245091	-0.2813942330
H	-5.7432526489	-2.1569714365	-0.2991522317
H	-4.8029541982	-1.0233570886	0.7273127937
H	-5.9017105907	-0.3881146775	-0.5436783339
O	-0.6570582331	2.2063117640	-1.1748797636
C	-1.2628542393	2.9621512494	-0.4323728435

C	-2.3551355668	3.9166639670	-0.8278163359
N	-1.0830535505	3.0449402513	0.9195751425
C	-2.7423803732	4.6386546356	0.4630848637
H	-3.1607722329	3.2855298586	-1.2366315372
H	-1.9992381558	4.5708192140	-1.6352381683
C	-1.9453056872	3.9628948953	1.5647865165
Br	0.0316204245	1.8842570431	1.8177294314
H	-2.4817870200	5.7070914818	0.4616796909
H	-3.8099311565	4.5643597020	0.7098977706
O	-1.9882015383	4.1389875294	2.7427644838
H	8.0857187643	-1.7218250506	-3.3768292459

EC3

0 1

O	1.1150658508	-1.0244704948	-0.7587422367
C	1.9220155910	-1.4737967281	0.2443870991
C	2.6645869831	-2.8319347250	-0.0231338426
C	3.3196894747	-0.8006993474	0.2931778050
C	4.0232163110	-2.1858146096	0.3555549131
H	2.6092692267	-3.0570535076	-1.0976439844
H	2.3344712720	-3.7116148999	0.5473798400
H	3.4826413531	-0.3153361102	-0.6779445695
H	3.5308356998	-0.0698617746	1.0876722344
C	5.1335065747	-2.3903864532	-0.6783155261
H	6.0091396658	-1.7636097803	-0.4463623716
H	5.4661384336	-3.4404116448	-0.6918043252
H	4.7787512663	-2.1266481494	-1.6865998095
C	4.5681896790	-2.5450986770	1.7310578977
C	4.3588008291	-3.7967666656	2.3172325934
C	5.3699313964	-1.6257532323	2.4217180065
C	4.9105632858	-4.1136264895	3.5587087894
H	3.7507260486	-4.5425396070	1.8018333342
C	5.9228550716	-1.9349900927	3.6618515430
H	5.5622920883	-0.6440909313	1.9800628279
C	5.6907082283	-3.1827586653	4.2402464340
H	4.7246343444	-5.0971363181	3.9967527095
H	6.5384772130	-1.1963972253	4.1808781500
C	1.1726397361	-1.5336480644	1.5874876910
C	1.6983053954	-1.1267677862	2.7578997118
H	2.7140545629	-0.7370004089	2.8312284777
H	1.1607428339	-1.2524946602	3.7007175106
C	-0.2177009922	-2.0776454099	1.5256998687
C	-0.6166705525	-2.9653776876	0.5136561791
C	-1.1993593023	-1.7030622715	2.4616720289
C	-1.9182846699	-3.4575297669	0.4303303257
H	0.0994053977	-3.2761927490	-0.2462340971
C	-2.4924078589	-2.1943433698	2.4014766909
H	-0.9544540716	-0.9811004159	3.2428310089
C	-2.8696242665	-3.0728088338	1.3780891633
H	-2.1743116209	-4.1258905136	-0.3906351128
H	-3.2451622362	-1.8862722207	3.1290716600
Mg	-0.5811915618	-0.3037636134	-0.9332022445
O	-2.4258192495	0.3010973015	0.0378449509
O	-2.1819880255	-0.8709176566	-2.1347369105
P	-3.1700253579	-0.2148466457	-1.1809297525
O	-4.3680614196	-1.2279343238	-0.8387763224
O	-3.9279182987	1.0436447557	-1.8683274783
C	-4.5508974960	0.8670904515	-3.1310731403
H	-5.3923572872	0.1596347428	-3.0585650742
H	-4.9305886853	1.8462646065	-3.4486611927

H	-3.8297861602	0.4921795507	-3.8729863234
C	-5.3160438537	-0.8581022373	0.1542777514
H	-6.0444075755	-1.6737729483	0.2257341052
H	-4.8226916657	-0.7256178345	1.1279303849
H	-5.8319730727	0.0734069583	-0.1287020607
O	-0.4643033066	1.7466422870	-1.3800254394
C	-0.9001613464	2.6351397112	-0.6657145952
C	-1.8736391348	3.7072188455	-1.0718384557
N	-0.6127922663	2.7899424760	0.6611485986
C	-2.0547864931	4.5749838043	0.1736541541
H	-2.7888671348	3.1708949285	-1.3703784316
H	-1.4927725658	4.2377427543	-1.9549525267
C	-1.2855229097	3.8700747087	1.2774248828
Br	0.3814507630	1.5375679969	1.5861109455
H	-1.6404256855	5.5878520359	0.0643963128
H	-3.1002958392	4.6836053772	0.4916418933
O	-1.2185465269	4.1382849687	2.4370842066
H	6.1181107838	-3.4278184246	5.2150399748
O	-4.1552872422	-3.4824009829	1.3766603156
C	-4.5983763352	-4.3042186901	0.3278071226
H	-5.6616763418	-4.5018977847	0.5133848068
H	-4.4849887094	-3.8006970431	-0.6470284318
H	-4.0598146820	-5.2674194490	0.3085545454

EC4
0 1

O	-1.1905436972	-0.3311755444	0.5427259828
C	-2.0693949327	-0.2040920775	-0.4919184498
C	-3.2773048384	-1.2113940051	-0.4596037481
C	-3.1034197311	0.9428189795	-0.3015297678
C	-4.2860257629	-0.0362646568	-0.5238674299
H	-3.2758230256	-1.7253112022	0.5115084320
H	-3.3515702354	-1.9505606471	-1.2725633519
H	-3.0322023442	1.2742772650	0.7419451514
H	-3.0564509241	1.8273161097	-0.9547941346
C	-4.9439388684	0.1003637628	-1.9014908064
H	-5.6691302909	-0.7126519749	-2.0620004142
H	-5.4857049482	1.0561380799	-1.9807371802
H	-4.1985224750	0.0538473722	-2.7088413466
C	-5.3693045103	-0.0204157581	0.5358958272
C	-5.8068230774	1.1847250092	1.0963983364
C	-6.0047073538	-1.2028177006	0.9312815789
C	-6.8423511849	1.2090373244	2.0292188514
H	-5.3263498632	2.1210653346	0.7999544051
C	-7.0401445044	-1.1845040131	1.8642099177
H	-5.6812938369	-2.1556392857	0.5034875711
C	-7.4628639007	0.0228903112	2.4184501633
H	-7.1648803282	2.1612532949	2.4571145183
H	-7.5183169587	-2.1206227011	2.1621740879
C	-1.3226992657	-0.2353484131	-1.8328218057
C	-1.5300041143	0.6445235678	-2.8291589401
H	-2.2655075107	1.4450963292	-2.7433798072
H	-1.0171051629	0.5605268324	-3.7900541098
C	-0.2964519336	-1.3146001250	-1.9862458990
C	-0.3804682562	-2.5165106151	-1.2599793599
C	0.7972633817	-1.1561658125	-2.8498874776
C	0.5576476270	-3.5283432633	-1.4288184902
H	-1.1888626532	-2.6647424534	-0.5461151087
C	1.7330430218	-2.1672717731	-3.0317882831
H	0.9382801301	-0.2149245118	-3.3827513993

C	1.6092063523	-3.3643897894	-2.3302113230
H	0.4696795588	-4.4503577356	-0.8522929646
H	2.5690734801	-2.0172974272	-3.7165158256
Mg	0.5778008150	-0.3342484064	1.0362540487
O	2.5225252389	-0.5406736199	0.1471959794
O	1.6416848934	-1.7098879627	2.1439388518
P	2.8751502343	-1.5000442926	1.2751038584
O	3.4488750310	-2.9105434977	0.7909242963
O	4.0992413613	-0.8462451490	2.1138773655
C	4.5060841370	-1.4628674077	3.3270724074
H	4.9106887271	-2.4699596509	3.1392495696
H	5.2917495375	-0.8349043583	3.7653454863
H	3.6605909454	-1.5409783618	4.0269864010
C	4.5421572675	-2.9506605665	-0.1156439250
H	4.7563565716	-4.0058254370	-0.3150164706
H	4.2804062938	-2.4495573914	-1.0588521300
H	5.4307536655	-2.4665392456	0.3200770455
O	1.3256609622	1.4561128799	1.8029550585
C	2.1910763236	2.1307477977	1.2657390311
C	3.5135583971	2.5259351499	1.8608582051
N	2.1264642859	2.6152391082	-0.0086263107
C	4.1881424725	3.3920263095	0.7964086782
H	4.0413944238	1.5773457638	2.0520328846
H	3.3516661285	3.0210576471	2.8278267675
C	3.2794780742	3.3287730765	-0.4181560206
Br	0.7471852190	2.1610854748	-1.1435444601
H	4.2882118404	4.4469464515	1.0908690396
H	5.1863219176	3.0394753136	0.5042587142
O	3.4441170116	3.7779285797	-1.5093367357
H	-8.2715691227	0.0390580658	3.1524334725
C	2.5542285883	-4.5045264436	-2.5950728844
F	2.7892189805	-5.2313223233	-1.4957998178
F	3.7419174340	-4.0811011081	-3.0522551358
F	2.0640994518	-5.3434989719	-3.5152394506

EC5
0 1

O	-0.8878564081	-0.6009694701	1.9509394368
C	-2.0094976799	-0.5485706783	1.1721795554
C	-3.1255272467	-1.6021223533	1.5043114982
C	-3.0239777245	0.5567758901	1.5740684017
C	-4.1845041036	-0.4755481938	1.6390517504
H	-2.8940675400	-2.0690556682	2.4717928885
H	-3.3146849907	-2.3872265567	0.7589590252
H	-2.7428313090	0.9121568623	2.5739093645
H	-3.1382664134	1.4260152716	0.9104231684
C	-4.9527157177	-0.4867111278	2.9633360796
H	-5.5526425694	0.4300058218	3.0778589888
H	-5.6397496446	-1.3464573019	3.0099852638
H	-4.2573268635	-0.5522718676	3.8142086119
C	-5.1865030546	-0.3707702965	0.4981664224
C	-5.6736397580	-1.4988759275	-0.1694529351
C	-5.7084669553	0.8786561067	0.1367587003
C	-6.6306477292	-1.3839893842	-1.1775868696
H	-5.3017093079	-2.4898272721	0.0991585852
C	-6.6632368647	1.0007615776	-0.8702432120
H	-5.3588964454	1.7763582356	0.6537520407
C	-7.1260683921	-0.1328272936	-1.5376012308
H	-6.9898611032	-2.2816708180	-1.6864340025
H	-7.0485233834	1.9881849689	-1.1354788115

C	-1.6411139625	-0.5594157471	-0.3288788732
C	-2.2962656925	0.1005135119	-1.2935984801
H	-3.1779158344	0.6958220482	-1.0602055503
H	-2.0140035310	0.0370466170	-2.3449817262
Mg	0.8095354793	-0.5953518361	1.1483609643
O	3.2465610391	-0.0244312646	-0.8868114351
O	2.3447935372	-1.7558797636	0.8246815915
P	3.4190638524	-1.3352715103	-0.1832029118
O	3.5224533466	-2.5817804574	-1.2127105023
O	4.8573485720	-1.3210340233	0.5656085372
C	5.2259321855	-2.3995793702	1.4055482132
H	5.2302567036	-3.3502315885	0.8482502154
H	6.2386340556	-2.1955182124	1.7760318285
H	4.5336400857	-2.4917114754	2.2561564572
C	4.3619524702	-2.4648498463	-2.3467463738
H	4.1931401161	-3.3500122338	-2.9729796450
H	4.1237735682	-1.5552368645	-2.9193186876
H	5.4231657328	-2.4308833701	-2.0501222337
O	1.9362944361	1.0673045461	1.6066597177
C	2.5800494095	1.8179157829	0.8871136721
C	4.0404941502	2.1539661411	0.9928622532
N	2.0489664109	2.5396551227	-0.1424982393
C	4.3631252323	2.8223211240	-0.3438720131
H	4.6019661559	1.2301027190	1.1804711559
H	4.1716737296	2.8268442044	1.8551472164
C	3.0249433626	3.1547496648	-0.9698221422
Br	0.2946926003	2.3317925809	-0.6356124856
H	4.9876397461	3.7222957939	-0.2821880078
H	4.8395570629	2.1012927137	-1.0242839584
O	2.7752735287	3.7735114924	-1.9568761917
H	-7.8713912397	-0.0409696451	-2.3306620215
N	-0.4432635641	-1.3139329490	-0.5964290842
C	-0.5716676497	-2.7388705812	-0.2316526796
H	-0.8923511604	-2.8385304835	0.8121724438
H	-1.3053082720	-3.2433988596	-0.8853026043
H	0.4098782694	-3.2192403178	-0.3437276448
C	0.1059957231	-1.2045202716	-1.9449675287
H	0.3065100339	-0.1556745836	-2.1950869317
H	1.0564708688	-1.7526368428	-1.9795727453
H	-0.5753183534	-1.6349724901	-2.7020292894

EP2
0 1

O	-0.4468264800	0.3314510940	-0.5627767075
C	0.8161418656	0.5711350691	0.0308177516
C	1.7481340124	-0.5257045336	0.5014833523
C	1.9411516581	1.3124840991	-0.6608533190
C	2.9330572315	0.4280710868	0.1591141647
H	1.6880930351	-1.3895751465	-0.1757870592
H	1.6519288516	-0.8616502776	1.5432919435
H	1.9596338189	1.0765201317	-1.7345742871
H	1.9881800757	2.4014015612	-0.5170636755
C	3.4431919312	1.1652435688	1.4003404790
H	3.9778840380	0.4713789390	2.0663807521
H	4.1391092324	1.9679933996	1.1131467915
H	2.6108459950	1.6096580600	1.9682480583
C	4.0869709218	-0.1774248478	-0.6079048229
C	4.7997674535	0.5861883162	-1.5387431251
C	4.5021445826	-1.4903087477	-0.3645649454
C	5.8952575185	0.0511879406	-2.2134369619

H	4.4919500088	1.6158645597	-1.7413483788
C	5.5981894255	-2.0292139918	-1.0364388985
H	3.9579621717	-2.1040479465	0.3580145386
C	6.2982779648	-1.2601626077	-1.9643063225
H	6.4360123419	0.6614182286	-2.9403292799
H	5.9046145892	-3.0583842604	-0.8362429872
C	-0.4155502738	1.1069310056	0.6579778220
C	-0.9921012668	0.3538484762	1.8393980308
H	-0.9457483226	-0.7257867849	1.6510232282
H	-2.0352019189	0.6375347188	2.0359850463
C	-0.8247800408	2.5367462106	0.4365738821
C	-0.8152358934	3.0685895573	-0.8600522280
C	-1.2596410189	3.3422164898	1.4933235792
C	-1.2175553676	4.3813495990	-1.0913468851
H	-0.5159271592	2.4380882039	-1.6989012752
C	-1.6598225566	4.6568649498	1.2597237778
H	-1.2992214835	2.9472980647	2.5085624449
C	-1.6387238053	5.1825739970	-0.0302659955
H	-1.2060654042	4.7776882569	-2.1090645683
H	-2.0002291273	5.2694394270	2.0967838229
H	-1.9561083856	6.2118967672	-0.2101168518
Mg	-1.9481578780	-0.9257316910	-1.1793968216
O	-1.7501681477	-2.4815709978	0.2195295427
O	-0.8391596160	-2.4435951172	-2.0716585567
P	-0.9157836808	-3.2619382643	-0.7893120650
O	0.5800134738	-3.5516081781	-0.2619125841
O	-1.5470762466	-4.7197537495	-0.9994148019
C	-1.0549034493	-5.5604798942	-2.0320974746
H	-0.0083226533	-5.8467127268	-1.8410100283
H	-1.6811505803	-6.4606520186	-2.0416851179
H	-1.1174278139	-5.0561677239	-3.0080527544
C	0.7755170185	-4.1067702815	1.0283445850
H	1.8583586160	-4.1884161737	1.1852334261
H	0.3382349002	-3.4604137994	1.8049375819
H	0.3221750836	-5.1081503471	1.1001140057
O	-2.8533258168	0.2549613796	-2.6679681808
C	-3.4349393436	0.9275216053	-1.7883088211
C	-4.2228030285	2.1996158072	-1.9835374989
N	-3.3562368381	0.5403719977	-0.5105722392
C	-4.5478688256	2.6012307886	-0.5452988093
H	-5.1048179300	1.9886611006	-2.6061805472
H	-3.6053233039	2.9282841681	-2.5279726764
C	-3.9777960062	1.4635031514	0.3108344252
Br	0.0154390356	0.6746256291	3.4795251082
H	-4.0473951449	3.5312101276	-0.2358373016
H	-5.6200125540	2.7203228947	-0.3362001149
O	-4.0282640601	1.4135327511	1.5175679438
H	7.1543455708	-1.6825170717	-2.4947021160

EP3
0 1

O	-2.0049647340	-0.9651705384	-2.7461223143
C	-1.4991322749	-0.2082587416	-1.6521221536
C	-1.6832604647	1.2911596544	-1.5742968415
C	-1.8690407907	-0.3908706016	-0.1943060186
C	-1.7362568479	1.1568651464	-0.0200551026
H	-2.6611293530	1.5698350036	-1.9907365593
H	-0.8961846110	1.9135658280	-2.0188970813
H	-2.9031961357	-0.7468314475	-0.0904014338
H	-1.1937616171	-1.0248743685	0.3947087282

C	-2.9483046567	1.8238410565	0.6297341420
H	-3.0087642585	1.5604897671	1.6969345718
H	-2.8713125185	2.9200961713	0.5582116785
H	-3.8752982335	1.4956161640	0.1357010948
C	-0.4600590137	1.5871741200	0.6890593554
C	0.2745832358	2.6981351662	0.2615142299
C	-0.0352780261	0.9246718362	1.8471823123
C	1.4074957754	3.1221683473	0.9542222374
H	-0.0364160159	3.2481790469	-0.6291732262
C	1.0955798478	1.3443512156	2.5441941914
H	-0.5985632292	0.0641822357	2.2170593001
C	1.8258850694	2.4440951242	2.0968746493
H	1.9679185080	3.9879737651	0.5944094550
H	1.4088284823	0.8063645407	3.4417824410
C	-0.5798255380	-0.9944403924	-2.5030171562
C	0.1768167205	-0.2547340649	-3.5799071786
H	-0.4571648848	0.5403681570	-3.9855932130
H	0.4726651868	-0.9242680134	-4.3978349204
C	-0.0951288521	-2.3514300413	-2.0780443053
C	-0.9324497318	-3.1995809828	-1.3314674997
C	1.1215789108	-2.8656573435	-2.5223791250
C	-0.5853326344	-4.5158511669	-1.0808391813
H	-1.8999743714	-2.8498548428	-0.9612279444
C	1.4896778566	-4.1876925775	-2.2690962283
H	1.8047184589	-2.2470479306	-3.1039987687
C	0.6253749352	-5.0300312833	-1.5650252669
H	-1.2568173202	-5.1778629252	-0.5328408154
H	2.4449088862	-4.5475760837	-2.6506962773
Mg	-3.3304478440	-2.4836747419	-3.2527361047
O	-4.9514809799	-1.3035731982	-3.7324822008
O	-4.3872707423	-2.0490195969	-1.4372451950
P	-5.3138509193	-1.1660652655	-2.2587760926
O	-5.1500691564	0.3560646033	-1.7357383134
O	-6.8697021763	-1.4856086891	-2.0518289838
C	-7.3950052038	-1.6208386702	-0.7407941056
H	-7.3442271998	-0.6645719506	-0.1955348239
H	-8.4440292419	-1.9238829664	-0.8426780205
H	-6.8428878900	-2.3874314998	-0.1767580188
C	-5.7683655170	1.4141515734	-2.4503399131
H	-5.4706064494	2.3510398192	-1.9622964680
H	-5.4374750663	1.4217196792	-3.4998770604
H	-6.8656869451	1.3222779313	-2.4197561088
O	-3.3547902858	-4.6018947285	-3.3052207680
C	-2.3496686846	-4.5934000342	-4.0454828717
C	-1.5042186069	-5.7748578900	-4.4542722844
N	-1.8925655128	-3.4240946610	-4.5121698240
C	-0.4075087222	-5.1128385820	-5.2884397219
H	-2.1212083622	-6.5044089921	-4.9981445112
H	-1.1332352636	-6.2724113432	-3.5456631370
C	-0.7123213491	-3.6095962005	-5.2024736903
Br	1.7640706731	0.6119100086	-2.8590410703
H	0.6070871247	-5.2835525613	-4.9024348755
H	-0.4105903430	-5.4158431344	-6.3456502405
O	-0.0065213385	-2.7367151193	-5.6525971370
H	2.7163951510	2.7715951938	2.6376204259
O	0.8557727078	-6.3371349537	-1.3276335108
C	2.0462872360	-6.9144056510	-1.7937211932
H	2.0229838832	-7.9685747517	-1.4930018881
H	2.1243283845	-6.8585941304	-2.8936596056
H	2.9347900201	-6.4350131770	-1.3482103819

EP4
0 1

O	-0.2694501292	0.3200916031	-0.4736867824
C	-0.7452519372	-0.9144224632	0.0317907556
C	-2.1434565354	-1.1218903760	0.5736986006
C	-0.8069342348	-2.1878424681	-0.7855450915
C	-2.0438485919	-2.5963254842	0.0760278662
H	-2.8639988804	-0.5238638856	-0.0021013359
H	-2.3002162254	-0.9587196963	1.6489951786
H	-1.0782133362	-1.9677028452	-1.8280387481
H	0.0734826448	-2.8458908751	-0.7598405140
C	-1.6474516732	-3.5496535363	1.2066218923
H	-2.4804660669	-3.6718446599	1.9151087327
H	-1.3935721067	-4.5416712185	0.8034869913
H	-0.7790984215	-3.1656026838	1.7647194146
C	-3.2382759919	-3.1364761783	-0.6778516803
C	-3.0647823616	-4.0608373905	-1.7137514817
C	-4.5396870962	-2.7699269452	-0.3215201638
C	-4.1623166060	-4.6011791735	-2.3807329510
H	-2.0547673925	-4.3621617819	-2.0056005614
C	-5.6405619397	-3.3089796012	-0.9853953923
H	-4.6971296527	-2.0481256213	0.4841891177
C	-5.4554175084	-4.2261284260	-2.0183200085
H	-4.0066021163	-5.3178089807	-3.1902282655
H	-6.6495965627	-3.0070525311	-0.6959379187
C	0.4262757136	-0.2535524234	0.6543686508
C	0.2098894905	0.5292006454	1.9329209902
H	-0.7164165163	1.1119934194	1.8589653304
H	1.0473716567	1.2113646828	2.1363183358
C	1.8237902452	-0.6776328636	0.2981565302
C	2.1780876540	-0.8277525982	-1.0491008921
C	2.8025520995	-0.8581042936	1.2781826210
C	3.4829564715	-1.1400737069	-1.4089310998
H	1.4312308132	-0.6619464359	-1.8265021512
C	4.1110713485	-1.1703839380	0.9209529207
H	2.5593344306	-0.7345305919	2.3331449329
C	4.4557020958	-1.3038119513	-0.4218015157
H	3.7480788060	-1.2463531165	-2.4622917790
H	4.8688836178	-1.2935454646	1.6956014192
Mg	-0.4566979329	2.3251089254	-0.8956719260
O	-1.7377125143	2.9356159808	0.6483073719
O	-2.3991326549	2.3147407790	-1.6446114137
P	-2.9295064382	2.7484191133	-0.2843150086
O	-3.9640977271	1.6352035874	0.2535155928
O	-3.7922683971	4.0970443361	-0.3338380373
C	-4.8424383685	4.2310327500	-1.2802502166
H	-5.6515440159	3.5123820169	-1.0740122782
H	-5.2317037181	5.2520012189	-1.1878210681
H	-4.4685277582	4.0688624962	-2.3022626961
C	-4.4331731482	1.6964366526	1.5906480979
H	-5.1037044552	0.8406778857	1.7382280281
H	-3.5967643851	1.6363836598	2.3040427937
H	-4.9904323791	2.6292112217	1.7713658032
O	0.9208471667	2.5909106793	-2.4599228398
C	1.8672272316	2.5949458295	-1.6413006587
C	3.3439964307	2.5937290987	-1.9508612026
N	1.5973731938	2.5658681691	-0.3320752407
C	3.9678251859	2.5119773617	-0.5576926280
H	3.6016035247	3.5015994934	-2.5154266514

H	3.5761838756	1.7344798543	-2.5968767123
C	2.7692634718	2.4900504246	0.3990953559
Br	0.0162740365	-0.6373997322	3.4839738942
H	4.5597338527	1.5990966464	-0.3993404809
H	4.6161889518	3.3631957927	-0.3062675793
O	2.8430165629	2.3851071991	1.6013741164
H	-6.3167822995	-4.6458138088	-2.5421756761
C	5.8905544924	-1.5297900466	-0.8204155425
F	6.5875251239	-2.1476198422	0.1344707949
F	6.5069889029	-0.3623888213	-1.0660513017
F	5.9888959838	-2.2613520669	-1.9339486380

EP5
0 1

O	-0.2105845477	-0.6185287972	0.6266098791
C	-1.5658562225	-0.1528154423	0.6921164271
C	-2.4842498271	-0.4192217147	1.8696388400
C	-2.0062942061	1.2987898677	0.6271686198
C	-3.2237795603	0.9116296137	1.5329514152
H	-1.9312848881	-0.3419951935	2.8170013972
H	-3.0723433397	-1.3464463057	1.8600871919
H	-1.2794585140	1.9337541770	1.1556085703
H	-2.2361780030	1.7285763031	-0.3548950741
C	-3.4393682836	1.8352624815	2.7245507994
H	-3.6755409044	2.8615583844	2.4063761316
H	-4.2577014984	1.4773991229	3.3682620958
H	-2.5219732570	1.8883428526	3.3287458287
C	-4.4720242800	0.6956117306	0.6810335478
C	-4.5907178372	-0.4174810861	-0.1685693236
C	-5.5143285800	1.6309652847	0.6707023780
C	-5.7004384739	-0.5792514751	-0.9953040207
H	-3.8162230419	-1.1885152963	-0.1906611138
C	-6.6269796898	1.4694897480	-0.1547503570
H	-5.4689340180	2.5089070990	1.3161060293
C	-6.7261064466	0.3641379123	-0.9949866247
H	-5.7648948185	-1.4579625005	-1.6414278039
H	-7.4231022575	2.2172595919	-0.1362698293
C	-1.1465336538	-1.1050801483	-0.3517461110
C	-0.8877341145	-0.6558801546	-1.7857112707
H	-1.8337789790	-0.5890534413	-2.3374833455
H	-0.2024860057	-1.3570429311	-2.2702992977
Mg	1.7319550011	0.0467164969	0.4740236054
O	1.9902607125	-1.5522091060	-0.9649242022
O	2.8729805779	-1.4407828138	1.3472289314
P	2.8676100774	-2.2543226006	0.0603651001
O	2.3656822369	-3.7551122857	0.3884293520
O	4.3361437373	-2.4738387989	-0.5491625651
C	5.4172973762	-2.8061243181	0.3061223445
H	5.2792967212	-3.8064405223	0.7486484103
H	6.3267658781	-2.8041890179	-0.3068872296
H	5.5160697337	-2.0676431469	1.1153527098
C	2.3494249065	-4.7239246833	-0.6433884813
H	1.8032447105	-5.5971882747	-0.2643815423
H	1.8421305291	-4.3403373147	-1.5432989742
H	3.3722287913	-5.0259485922	-0.9184380342
O	1.1654966498	1.6831747599	1.7850266935
C	2.0094803414	2.3951643846	1.1988281820
C	2.3260781816	3.8470486960	1.4752370346
N	2.7349478209	1.8746133493	0.2060463156
C	3.4302167325	4.1384356098	0.4587590053

H	2.6308951631	3.9603628936	2.5256455873
H	1.4152785523	4.4486050400	1.3383156283
C	3.6077070990	2.8186806827	-0.3162428709
Br	0.0049105214	1.0515241505	-1.9722217815
H	3.1811034873	4.9379873307	-0.2532845024
H	4.3920587848	4.4079499541	0.9181245990
O	4.3782327346	2.6475037061	-1.2236889295
H	-7.5972127683	0.2357126724	-1.6408585598
N	-1.5167814623	-2.4763506655	-0.2365778989
C	-1.0738242184	-3.1705231173	0.9671402587
H	-1.1601151218	-2.5388462008	1.8556921322
H	-1.7088891203	-4.0557513553	1.1188302362
H	-0.0191103669	-3.4911082111	0.8943728464
C	-1.4151491088	-3.3313531883	-1.4038594494
H	-0.3708417270	-3.5520124486	-1.6993233724
H	-1.9062699210	-4.2863916720	-1.1696600842
H	-1.9464299950	-2.9090880752	-2.2669264743

IN5
0 1

O	0.2202392161	-0.9862786520	-0.9660013293
C	-0.8864770761	-0.6867166568	-0.2384493168
C	-1.8755325033	0.2784255371	-0.9505406125
C	-2.0122889416	-1.7686747322	-0.2699387051
C	-2.7431380023	-0.9420424638	-1.3591964580
H	-1.4157915184	0.8730080927	-1.7529607371
H	-2.4324386607	0.9340618375	-0.2659636512
H	-1.6710020058	-2.7742209152	-0.5543338903
H	-2.5860840818	-1.8174756309	0.6690755669
C	-2.4135071679	-1.4397877816	-2.7699146596
H	-2.8967698858	-2.4114140020	-2.9574503055
H	-2.7810159480	-0.7257147703	-3.5229525865
H	-1.3264812060	-1.5519706298	-2.8878523065
C	-4.2393731582	-0.7846735441	-1.1985468213
C	-4.8726538899	0.4189761521	-1.5270349727
C	-5.0312426853	-1.8564888825	-0.7722065229
C	-6.2563126729	0.5520482240	-1.4244231742
H	-4.2727153320	1.2667645904	-1.8690435494
C	-6.4153498323	-1.7285257926	-0.6676483274
H	-4.5567497247	-2.8083787153	-0.5179402159
C	-7.0333991884	-0.5217378321	-0.9921281473
H	-6.7306534231	1.5016506332	-1.6829477078
H	-7.0150020032	-2.5769344968	-0.3296246676
C	-0.3824710735	-0.2528646870	1.1440217505
C	-0.5538879287	1.1700598485	1.5690990200
H	-0.6735760138	1.8194778148	0.6974949752
H	0.2615530303	1.5397569705	2.1912379606
Mg	2.0361882813	-0.3891016754	-1.0009748754
O	2.0765346972	1.2147607587	0.5857009972
O	2.2316005550	1.4910763030	-1.8790527365
P	2.2120393732	2.2184220362	-0.5478503259
O	1.0029990010	3.2965156082	-0.5533588850
O	3.5216279085	3.1218714094	-0.3105057222
C	4.0518930714	3.8847988389	-1.3827852493
H	3.3681812385	4.7025665174	-1.6645551085
H	5.0013039000	4.3127241812	-1.0379204527
H	4.2283538839	3.2502489872	-2.2638149938
C	0.9153225611	4.2786268589	0.4606267727
H	-0.0526431657	4.7828639532	0.3454786564
H	0.9698863107	3.8306634013	1.4665775566

H	1.7229718505	5.0214193129	0.3678087016
O	3.4811118758	-1.5167885856	-2.0877648452
C	3.8676644516	-2.0795938387	-1.0469713137
C	5.0064221319	-3.0666575055	-0.9173817614
N	3.2551664943	-1.8207311379	0.1179711658
C	5.0391553860	-3.3249884926	0.5885133531
H	5.9244532105	-2.6260609405	-1.3310093759
H	4.7784003639	-3.9587054627	-1.5192590905
C	3.8393661889	-2.5352337981	1.1340126688
Br	-2.2010572279	1.2709516916	2.6147126546
H	4.9349544411	-4.3820778028	0.8702712704
H	5.9524370351	-2.9526997720	1.0757324500
O	3.4685676791	-2.5670325677	2.2905345232
H	-8.1174483461	-0.4183795564	-0.9083499577
N	0.2618347188	-1.0781664811	1.9073751611
C	0.3579348289	-2.5092217478	1.5947857431
H	0.3571130977	-2.6396804388	0.5088073909
H	1.3037560685	-2.8787273176	2.0039261302
H	-0.4936612017	-3.0295562669	2.0572557857
C	0.9666762101	-0.6280683884	3.1142937477
H	1.7056205833	0.1303093662	2.8208657129
H	0.2514659883	-0.2214050603	3.8430413855
H	1.5087182327	-1.4774009033	3.5374542575

TS2a
0 1

O	0.4954553344	-1.0559903412	1.6688631060
C	0.8631483624	-2.3348521328	1.4025669843
C	2.0053744991	-3.0033600649	2.2108052070
C	-0.0329783483	-3.5074830766	1.8611634482
C	1.2500334973	-4.3519952353	2.1099102150
H	1.9510201940	-2.6123550963	3.2371681725
H	3.0425764041	-2.9210439717	1.8599968029
H	-0.4947197010	-3.2095570924	2.8132285762
H	-0.8195603050	-3.8946460931	1.1985135403
C	1.1320579985	-2.2217293287	-0.0901376169
C	0.1778743940	-2.7025982793	-1.0426459267
H	-0.2434389320	-3.6823655533	-0.7989971417
H	0.4535015937	-2.6002043303	-2.0942306437
Mg	-0.3759346331	0.5291424591	1.0818056673
O	0.4474959208	1.5121834787	-0.5780800212
O	0.7202618803	2.1811741311	1.7904889088
P	1.0376931935	2.5615342562	0.3524991555
O	2.6330764139	2.7249906202	0.1753511708
O	0.4649522090	4.0099496218	-0.0532496118
C	0.7118565128	5.1188823578	0.7929993774
H	1.7864602044	5.3629921372	0.8222462244
H	0.1597812356	5.9728222884	0.3809783698
H	0.3641656292	4.9148557036	1.8171730598
C	3.1524973933	2.9257337211	-1.1274263487
H	4.2436300778	3.0027838674	-1.0363872025
H	2.8986505192	2.0789470695	-1.7839326872
H	2.7596985001	3.8551674532	-1.5707387493
O	-2.2978452795	0.9655065981	1.2381713840
C	-3.2922849367	0.7568686579	0.5078327255
C	-4.5985630882	1.5075277487	0.6808267535
N	-3.3473790764	-0.1209062648	-0.4836413727
C	-5.4796315745	0.9333584700	-0.4243777495
H	-4.4023401118	2.5860053768	0.5941145238
H	-4.9764953541	1.3310184122	1.6986837031

C	-4.5963452390	-0.1230047457	-1.1014929378
Br	-1.5232121760	-1.4733390701	-0.8473747169
H	-6.3976448778	0.4497217671	-0.0619422261
H	-5.7810253751	1.6735754502	-1.1793463519
O	-4.9221467335	-0.8365740849	-2.0113829198
C	2.3383398349	-1.5208323548	-0.5237808834
C	2.8948915115	-1.8198179671	-1.7842067433
C	3.0334608421	-0.6287883177	0.3212553907
C	4.1053429418	-1.2624221811	-2.1782273205
H	2.4055261379	-2.5365232820	-2.4439199921
C	4.2346115374	-0.0662162782	-0.0828058871
H	2.5908381780	-0.3536848012	1.2786432612
C	4.7762998770	-0.3827267083	-1.3295905357
H	4.5284366924	-1.5201749834	-3.1508380069
H	4.7383711891	0.6429615560	0.5755630058
H	5.7245087712	0.0604341264	-1.6425745294
C	1.2721343357	-5.2355216195	3.3535888574
H	0.5723127573	-6.0798272336	3.2579739435
H	2.2783414573	-5.6514686825	3.5171381656
H	0.9884509438	-4.6566990726	4.2457690089
C	1.6024076325	-5.1639527274	0.8659133266
C	2.8070828485	-5.0194946211	0.1713522208
C	0.6702743935	-6.0902874030	0.3732933597
C	3.0636718837	-5.7538053339	-0.9888983649
H	3.5637611752	-4.3175505342	0.5242697075
C	0.9211902631	-6.8265126551	-0.7801237793
H	-0.2795820470	-6.2265408317	0.8976835470
C	2.1210788940	-6.6551178582	-1.4733819309
H	4.0106310780	-5.6137930442	-1.5153598344
H	0.1741809750	-7.5350745736	-1.1448731705
H	2.3182718807	-7.2258603229	-2.3832762281

TS2b
0 1

O	-0.1706470726	-0.8195503489	1.6358279802
C	-1.2934151926	-0.6299161314	0.9782612371
C	-2.5287945645	-1.8195930281	1.1001577140
C	-2.3252677616	0.3217953304	1.6133013188
C	-3.4647204362	-0.7312971705	1.6591153418
H	-2.0984956870	-2.4954753351	1.8504377610
H	-2.8653299796	-2.4020557483	0.2329087858
H	-1.9574324012	0.5850589628	2.6115460969
H	-2.5110489536	1.2518105125	1.0602661859
C	-1.2893710471	-0.8174479381	-0.4662537547
C	-2.0787964419	0.0861335688	-1.3224536745
H	-3.0060153383	0.4553478617	-0.8779896954
H	-2.2559344960	-0.2632465968	-2.3430835065
Mg	1.5149298052	0.0465615997	1.2372130781
O	2.6400061419	-0.6445109538	-0.4034806663
O	3.1364018404	-1.0135934981	1.9931582631
P	3.6434064569	-1.2360572663	0.5739242318
O	3.9050009497	-2.8098432586	0.3378210767
O	5.0878572418	-0.5728762528	0.3214651283
C	6.1265533847	-0.7842352613	1.2615567349
H	6.4274451924	-1.8446373430	1.2895897335
H	6.9823806884	-0.1757969118	0.9437380452
H	5.8100963824	-0.4779227764	2.2701034654
C	4.4081044406	-3.2323366482	-0.9165000769
H	4.3182509452	-4.3259040935	-0.9547344999
H	3.8290153589	-2.7894919027	-1.7420730536

H	5.4676732719	-2.9531125096	-1.0322664668
O	1.6607137306	1.9685792036	1.3222760284
C	1.7431274182	3.0233752508	0.6304617348
C	2.6248098569	4.1763417961	1.0858252770
N	1.1102026808	3.2567067599	-0.4982719842
C	2.4381054697	5.1993937471	-0.0259693362
H	3.6562606091	3.8134367815	1.2063148915
H	2.2872799550	4.5160705366	2.0766040364
C	1.4265117205	4.5261384825	-0.9690270224
Br	-0.7409634926	1.5690279267	-1.2612694939
H	2.0311718791	6.1641979301	0.3087336822
H	3.3571111981	5.4151896234	-0.5900918637
O	0.9756621777	5.0297133828	-1.9668895922
C	-3.9342272443	-1.0441523405	3.0843185783
H	-4.4685560233	-0.1798585863	3.5070692920
H	-4.6206710635	-1.9046512690	3.0903620875
H	-3.0770472352	-1.2736842748	3.7353746004
C	-4.6775628443	-0.4200624311	0.7930952135
C	-5.3647417729	-1.4307837184	0.1119878864
C	-5.1775791637	0.8858370869	0.7168676545
C	-6.5009715525	-1.1437773305	-0.6434406115
H	-5.0160762625	-2.4648571156	0.1715906378
C	-6.3124103723	1.1775017934	-0.0375308027
H	-4.6727504438	1.6931921551	1.2531979750
C	-6.9760077044	0.1634318586	-0.7263320622
H	-7.0175533823	-1.9489219262	-1.1705623255
H	-6.6786147909	2.2051274840	-0.0878409437
H	-7.8620580230	0.3910840490	-1.3223723880
C	-0.4020779157	-1.8129378864	-1.0828703403
C	-0.0947213701	-1.7410099968	-2.4544801018
C	0.1530392872	-2.8658405527	-0.3274421608
C	0.7216550045	-2.6925076445	-3.0509257143
H	-0.4536840061	-0.9091531401	-3.0605379115
C	0.9460605026	-3.8290237865	-0.9335450407
H	-0.0292014523	-2.9179315655	0.7440245113
C	1.2303613860	-3.7476813435	-2.2960290573
H	0.9634833156	-2.6063278182	-4.1115400382
H	1.3632686573	-4.6362329414	-0.3297317126
H	1.8635045384	-4.5016590427	-2.7691303674

TS2c

0 1

O	0.7089150540	0.5244115239	-2.3122787702
C	-0.5044228991	0.3197755535	-1.7764546949
C	-1.1940474632	1.5219117644	-1.0671216425
C	-1.7427150990	0.3456109152	-2.8114410145
C	-2.4642465205	1.4058328663	-1.9461496195
H	-0.6191464043	2.4273541198	-1.2963867696
H	-1.3198869682	1.4631596815	0.0169348152
H	-1.3131704044	0.7422828665	-3.7409050615
H	-2.2821050825	-0.5876768286	-3.0307343947
C	-2.8158093730	2.6904226991	-2.7008571614
H	-3.6157907081	2.5107148346	-3.4358838724
H	-3.1677044966	3.4629698266	-1.9998249958
H	-1.9349413374	3.0822721571	-3.2316481466
C	-3.7108801767	0.8888503317	-1.2401562051
C	-3.9899120191	1.2150644413	0.0903354636
C	-4.6543942268	0.1273475422	-1.9408689606
C	-5.1577044336	0.7731428162	0.7096671328
H	-3.2795593443	1.8162602441	0.6616500185

C	-5.8247069229	-0.3152636216	-1.3283806802
H	-4.4744431531	-0.1250108269	-2.9894158139
C	-6.0789828399	0.0015944071	0.0049988625
H	-5.3451819217	1.0314272109	1.7543683875
H	-6.5429068197	-0.9103138721	-1.8973107916
C	-0.6989772755	-1.0504413665	-1.1928297928
C	-0.0646070113	-2.1369654234	-1.8581944899
H	0.2880869350	-1.9305579727	-2.8723100761
H	-0.4695705293	-3.1366377841	-1.6730899749
C	-1.5558486488	-1.3133897144	-0.0313590765
C	-1.2462220264	-0.7252497519	1.2060355892
C	-2.6620203008	-2.1667832792	-0.1458501294
C	-2.0447160899	-0.9988698050	2.3110072629
H	-0.3574133854	-0.0916243795	1.2940298946
C	-3.4830476849	-2.3913039764	0.9537345007
H	-2.9037002851	-2.6189992130	-1.1102977233
C	-3.1699541545	-1.8139016263	2.1833637450
H	-1.7873749624	-0.5678933757	3.2806632245
H	-4.3676765085	-3.0218583704	0.8508978891
Mg	2.1823787090	0.9312845076	-1.1486169413
O	1.3869014743	1.1609074183	0.8273864491
O	2.3086888778	2.9525705806	-0.6175778469
P	1.7183030084	2.6458444963	0.7492866618
O	0.4306664982	3.5851500486	0.9931789686
O	2.7034659956	3.0261402756	1.9617462855
C	3.3253772075	4.3002144275	1.9841506027
H	2.5797519375	5.1019648743	2.1102274053
H	4.0140077450	4.3105950801	2.8380207905
H	3.8870691342	4.4770048583	1.0545147138
C	-0.3786146664	3.3870154423	2.1356982359
H	-1.1515543832	4.1657944975	2.1279801000
H	-0.8618756976	2.3963393723	2.1120749409
H	0.2116748903	3.4699982133	3.0625741544
O	4.0268167392	0.2506155745	-0.9534787081
C	4.5287319326	-0.7071364982	-0.3192193945
C	5.9875830133	-0.7255706975	0.0946677172
N	3.8826621939	-1.7987745629	0.0637497361
C	6.1177448467	-2.0450657403	0.8485522182
H	6.1975567357	0.1692136563	0.6982305781
H	6.6126737177	-0.6576709253	-0.8080903466
C	4.7253611866	-2.6807816105	0.7383244363
Br	1.7198010953	-2.0413443500	-0.7461013835
H	6.8597843294	-2.7359520921	0.4245019009
H	6.3623400561	-1.9220904139	1.9134747884
O	4.4084308819	-3.7598675273	1.1608210467
H	-6.9927089034	-0.3475078841	0.4908053731
H	-3.8068778276	-2.0038368856	3.0500216295

TS2d
0 1

C	0.9003732528	-1.0497363942	0.4645477108
C	1.9118560997	-1.8536625131	1.3516646645
C	2.5930918332	-0.5793272376	1.9111075220
H	2.5584135290	-2.5849801315	0.8566190064
H	1.2983786243	-2.3723510412	2.0972656588
C	1.1929505957	-0.8526312436	-0.9815405085
C	0.0777469223	-0.6750229182	-1.8691189010
H	-0.7863329698	-1.3090973496	-1.6236823476
H	0.3269486124	-0.6323073373	-2.9328316569
Mg	-2.0884183874	-0.4233791159	0.7850393857

O	-3.5716712987	-1.6629406555	1.5591935261
O	-3.2094786968	-1.1590324256	-0.8391525905
P	-4.0615208910	-1.9483847225	0.1466285001
O	-3.9972443239	-3.5102332124	-0.2365487075
O	-5.6264827543	-1.5896678866	0.0472794350
C	-6.2682388438	-1.6110988265	-1.2153054523
H	-6.2708014306	-2.6278179086	-1.6410869812
H	-7.3035118335	-1.2825952648	-1.0602530469
H	-5.7681350088	-0.9300781563	-1.9208914509
C	-4.5535801895	-4.4664716135	0.6483125226
H	-4.3363311504	-5.4591166198	0.2344159106
H	-4.1063356637	-4.3793365401	1.6502263556
H	-5.6453773875	-4.3401131864	0.7321968280
O	-2.4326606756	1.4297612080	1.3924376885
C	-2.3939491314	2.5937368722	0.9266624865
C	-3.0376381216	3.7656099781	1.6460746927
N	-1.8075946395	2.9554204904	-0.2047843078
C	-2.7507412344	4.9409505328	0.7181016990
H	-2.5897904404	3.8585199134	2.6465890581
H	-4.1056592078	3.5491267723	1.7938932402
C	-1.9318019374	4.3242545637	-0.4251916793
Br	-0.6820495891	1.1768446710	-1.2965653681
H	-3.6546026220	5.4008423720	0.2932309103
H	-2.1658605900	5.7484127873	1.1807746427
O	-1.4688274172	4.9246628264	-1.3586070295
O	-0.3738140577	-1.2450027923	0.7697310010
C	1.5798768323	0.2880595707	1.1357109097
H	0.7903624134	0.7255103551	1.7586402165
H	1.9714987996	1.0542331610	0.4582971233
C	2.4640802661	-0.4290684194	3.4294037653
H	3.0740040075	-1.1836477758	3.9496979027
H	2.8095242264	0.5655277757	3.7512880589
H	1.4168047813	-0.5540639682	3.7437434695
C	4.0544867438	-0.4228196448	1.5151910838
C	4.9354427755	-1.4946975470	1.7112261333
C	4.5750709769	0.7725398495	1.0135367479
C	6.2859916748	-1.3859009393	1.3925295395
H	4.5562023459	-2.4364868997	2.1180983718
C	5.9270377884	0.8863883303	0.6895407965
H	3.9219364202	1.6341263840	0.8613573468
C	6.7875963849	-0.1928246293	0.8723516059
H	6.9514753681	-2.2376138212	1.5513980415
H	6.3070328561	1.8286341582	0.2884420674
H	7.8456474294	-0.1042406723	0.6166114578
C	2.5445752910	-0.6442273414	-1.5318822941
C	3.5535270148	-1.6108237263	-1.4183277360
C	2.8206453511	0.5491315792	-2.2249697971
C	4.8109385994	-1.3838118526	-1.9689272621
H	3.3521934296	-2.5610789715	-0.9269363952
C	4.0889141726	0.7839266680	-2.7412821935
H	2.0438260118	1.3105991667	-2.3229008087
C	5.0874171746	-0.1817419129	-2.6140722409
H	5.5838797311	-2.1481573114	-1.8747802700
H	4.2968010351	1.7276000791	-3.2490949727
H	6.0832571228	0.0031294617	-3.0216100848

TS3a
0 1

O	0.4144706507	-0.1747326069	1.8827967482
C	1.6660818539	-0.0599354598	1.3790579178

C	2.6789883177	0.8835893258	2.1205977157
C	2.6589568844	-1.2094197666	1.6985549656
C	3.7920025658	-0.1789187709	1.9658619775
H	2.3394478434	0.9427033168	3.1647868242
H	2.8465909552	1.9026700606	1.7466314721
H	2.3136564510	-1.6583332942	2.6395814981
H	2.8239303206	-2.0185630603	0.9729251968
C	4.6488245431	-0.4211434448	3.2076553889
H	5.2919555738	-1.3059909474	3.0834432922
H	5.3024918348	0.4429097549	3.4044970398
H	4.0133363091	-0.5826302303	4.0917421601
C	4.6883697798	0.0081284841	0.7463060164
C	4.8843101048	1.2416821926	0.1193016461
C	5.3577081422	-1.1071796125	0.2198539054
C	5.7030807939	1.3565745595	-1.0063110580
H	4.3906583495	2.1358919057	0.5026726404
C	6.1748432449	-0.9991275199	-0.9010415198
H	5.2243935573	-2.0845524136	0.6919856977
C	6.3473228063	0.2376192680	-1.5250294562
H	5.8340134403	2.3324264019	-1.4796850028
H	6.6777264152	-1.8858686741	-1.2935670008
C	1.5442826791	0.3401058946	-0.0997268656
C	2.0015321299	-0.5408775627	-1.1201970606
H	2.8390474072	-1.1893901270	-0.8617745602
H	2.0545890248	-0.1729438017	-2.1457637998
C	0.8291943313	1.5584619649	-0.4141606341
C	0.3231185146	2.4070410387	0.6074194750
C	0.6092551210	1.9606911737	-1.7475601289
C	-0.3179204514	3.5862010518	0.3087363403
H	0.4075871441	2.0992686862	1.6481680323
C	-0.0465717272	3.1383961032	-2.0630403853
H	0.9471669657	1.3368197016	-2.5741359554
C	-0.5092069586	3.9692121603	-1.0305105955
H	-0.7181938241	4.2243622973	1.0965688712
H	-0.1969462348	3.3992330216	-3.1097864144
Mg	-1.3231503623	-0.6649390465	1.3288125119
O	-2.3449596391	0.4193188288	-0.1756479703
O	-2.8339632790	0.4561507093	2.2546951563
P	-3.2887997510	0.9520282365	0.8911156063
O	-3.3764706213	2.5631441327	0.9026104316
O	-4.7942534787	0.4989867185	0.5358527339
C	-5.8187088965	0.6918653086	1.4944522742
H	-5.9897206299	1.7647814471	1.6826043357
H	-6.7357736517	0.2480069794	1.0868847753
H	-5.5626211311	0.2010910447	2.4457809569
C	-3.7144791458	3.2303409140	-0.2991718814
H	-3.4633074102	4.2905197784	-0.1700216098
H	-3.1384714959	2.8239098780	-1.1466255665
H	-4.7899463766	3.1291825537	-0.5178945991
O	-2.0498694332	-2.5017252443	1.1388950033
C	-2.1660026565	-3.3061773479	0.1907579340
C	-3.2282675321	-4.3889433382	0.1684353443
N	-1.4051209814	-3.3354127026	-0.8968272962
C	-2.9888658333	-5.0886762903	-1.1657996267
H	-4.2156954201	-3.9143567926	0.2629725191
H	-3.0958710695	-5.0345533991	1.0490653585
C	-1.7848149071	-4.3531411838	-1.7677243999
Br	0.3371904815	-1.8764833371	-1.0614560669
H	-2.7436040783	-6.1563319002	-1.0757966191
H	-3.8332857240	-5.0089869757	-1.8652725235

O	-1.2528725261	-4.6084346329	-2.8146906930
O	-1.1504993432	5.1244535817	-1.2278635966
C	-1.4406970844	5.5471701730	-2.5387504897
H	-2.0812872911	4.8184855887	-3.0629606035
H	-1.9782617903	6.4977076264	-2.4463903227
H	-0.5207777393	5.7101598077	-3.1238643684
H	6.9832969380	0.3257148128	-2.4084130924

TS3b
0 1

O	-0.4515803986	-0.5738030990	1.8910380125
C	-1.5572580918	-0.5552752112	1.1728129996
C	-2.6201170956	-1.8861916145	1.3435976283
C	-2.7241583650	0.2976387370	1.7072561096
C	-3.6941507077	-0.9046241547	1.8573782140
H	-2.1172522586	-2.4834916480	2.1145577246
H	-2.8791953376	-2.5265137588	0.4900613893
H	-2.4181087731	0.7218067453	2.6700555232
H	-3.0369272513	1.1213687614	1.0531548446
C	-1.4661577301	-0.8201478657	-0.2582157970
C	-2.4136869235	-0.1678153891	-1.1968844432
H	-3.4112645200	-0.0036628697	-0.7794505898
H	-2.4968014759	-0.6624196891	-2.1694223471
Mg	1.1059167425	0.5026059945	1.5295882083
O	2.5704917678	-0.1597750398	0.1369163654
O	2.8381321379	0.1119223482	2.5841052983
P	3.5608825334	-0.2359006038	1.2872789303
O	4.2605609186	-1.6742568818	1.4515977654
O	4.7911917618	0.7602583749	0.9817905454
C	5.7159751552	1.0678737221	2.0116977017
H	6.2948122779	0.1761120932	2.3023595982
H	6.3998533067	1.8294989929	1.6172380596
H	5.1963926752	1.4611803225	2.8983024081
C	4.9993691448	-2.2375150818	0.3819191023
H	5.5526540894	-3.0951075906	0.7855067763
H	4.3318221969	-2.5852409331	-0.4204220983
H	5.7153870224	-1.5097823066	-0.0328020595
O	0.7465083992	2.3147956785	1.0248110021
C	1.0932403512	3.1528838815	0.1373029899
C	2.4784070167	3.7833342001	0.1629112591
N	0.3390843479	3.5448716476	-0.8620362760
C	2.4425580429	4.6997670918	-1.0510967070
H	3.2320796598	2.9817486083	0.1065676406
H	2.6265406082	4.2995745093	1.1234391474
C	1.0459930976	4.4462386506	-1.6503760543
Br	-1.5130081115	1.5786165501	-1.3685723627
H	2.5370471349	5.7684383434	-0.8088650981
H	3.2039458888	4.4718069298	-1.8106711405
O	0.6374947867	4.9566169411	-2.6638853038
C	-4.0709324542	-1.1807208413	3.3171014942
H	-4.7161328913	-0.3758238682	3.7006227260
H	-4.6198635056	-2.1306145574	3.4079186379
H	-3.1698224075	-1.2327586242	3.9467979601
C	-4.9601831889	-0.8408387469	1.0158646972
C	-5.5266336420	-2.0011777014	0.4756143906
C	-5.6283476864	0.3739026012	0.8215970001
C	-6.7116591264	-1.9482322081	-0.2563238011
H	-5.0406823057	-2.9681535315	0.6305463534
C	-6.8132589195	0.4315110389	0.0895977577
H	-5.2183282026	1.2938833635	1.2452764594

C	-7.3571884864	-0.7294033885	-0.4569780669
H	-7.1326716603	-2.8662643147	-0.6722274612
H	-7.3125288920	1.3919326086	-0.0551366573
H	-8.2825212623	-0.6847601327	-1.0349137331
C	-0.3789726027	-1.6292134520	-0.7786819851
C	0.0227090480	-1.4993289933	-2.1227486757
C	0.3521773966	-2.5266649082	0.0401256964
C	1.1237176636	-2.1750853623	-2.6217172838
H	-0.4849262269	-0.7978413626	-2.7852562591
C	1.4192874460	-3.2367128603	-0.4590373546
H	0.0765021578	-2.6506566268	1.0851287136
C	1.8385726102	-3.0441242064	-1.7863638636
H	1.4290741243	-2.0000134841	-3.6521955841
H	1.9815130267	-3.9245757411	0.1732457467
O	2.9311263227	-3.7177462769	-2.1623239269
C	3.4441488974	-3.5448888885	-3.4628518837
H	4.3377277553	-4.1758630397	-3.5294369669
H	2.7188305322	-3.8681110154	-4.2273912393
H	3.7263614557	-2.4952948667	-3.6463848579

TS3c
0 1

O	-0.7232621025	0.5335623343	2.2923468531
C	0.4931036775	0.3172438240	1.7644149868
C	1.1942921686	1.5173667060	1.0582824919
C	1.7219793463	0.3371902117	2.8028019025
C	2.4637311694	1.3819953936	1.9354035416
H	0.6285851809	2.4257014804	1.2995277141
H	1.3197524596	1.4743555169	-0.0262071511
H	1.3007704529	0.7493801908	3.7294234712
H	2.2464162507	-0.6027576423	3.0304781671
C	2.8393702367	2.6623653182	2.6866415894
H	3.6368474161	2.4703212509	3.4213970521
H	3.2038564087	3.4278281578	1.9841514167
H	1.9660251114	3.0701951415	3.2177517723
C	3.7047796164	0.8502384117	1.2283626619
C	4.0013141224	1.1994395051	-0.0928031999
C	4.6346392131	0.0646980716	1.9218102954
C	5.1746889796	0.7654706111	-0.7084944992
H	3.3010068280	1.8165337510	-0.6594784047
C	5.8105551687	-0.3699578283	1.3143888268
H	4.4409400095	-0.2087358801	2.9624531572
C	6.0850375856	-0.0226594283	-0.0077760704
H	5.3728549125	1.0416222647	-1.7467448667
H	6.5191194759	-0.9797488252	1.8798736376
C	0.6734302543	-1.0586312833	1.1717713699
C	0.0478683100	-2.1406433000	1.8547929732
H	-0.2811738203	-1.9333641306	2.8766643649
H	0.4434581652	-3.1431814170	1.6638486694
C	1.5188948261	-1.3122278856	0.0145819068
C	1.2796697171	-0.6542248803	-1.2102789297
C	2.6015343722	-2.1945551745	0.1128676446
C	2.1259934637	-0.8583652624	-2.2806880874
H	0.3956277809	-0.0159531510	-1.3106888980
C	3.4904185718	-2.3568967707	-0.9422353134
H	2.7951247991	-2.7143505830	1.0539036642
C	3.2639452879	-1.6729835733	-2.1432758306
H	1.9467854129	-0.3775631304	-3.2434910776
H	4.3613446191	-2.9970044177	-0.8106041530
Mg	-2.1899142927	0.9321240207	1.1225215593

O	-1.3874460117	1.1587196205	-0.8530150642
O	-2.3152492886	2.9541036627	0.5845266966
P	-1.7166304423	2.6441754804	-0.7775437757
O	-0.4258871724	3.5814499657	-1.0154693840
O	-2.6931371315	3.0253652528	-1.9972370864
C	-3.3134989796	4.2998444252	-2.0233490669
H	-2.5663003792	5.1010510567	-2.1439011859
H	-3.9962912836	4.3115160842	-2.8819386442
H	-3.8813070206	4.4768620259	-1.0974561720
C	0.3860172307	3.3856781759	-2.1563784655
H	1.1418605373	4.1811272686	-2.1605086778
H	0.8917082772	2.4066496535	-2.1201028314
H	-0.2066355402	3.4429097231	-3.0836864197
O	-4.0395786581	0.2610200879	0.9296073776
C	-4.5471448844	-0.7151304596	0.3299857465
C	-6.0079801051	-0.7432433178	-0.0761724215
N	-3.9059206588	-1.8213845208	-0.0192450842
C	-6.1453170197	-2.0863470617	-0.7857394094
H	-6.2190003325	0.1319686205	-0.7074111722
H	-6.6281755862	-0.6444639985	0.8271225367
C	-4.7546623633	-2.7227755201	-0.6597523935
Br	-1.7593867280	-2.0513329129	0.7738064595
H	-6.8881492095	-2.7603885525	-0.3366914461
H	-6.3932614764	-1.9974705366	-1.8532861428
O	-4.4432803126	-3.8168704386	-1.0464266915
H	7.0086573776	-0.3577734065	-0.4858460933
O	4.0774801698	-1.7320960487	-3.2068739582
C	5.2539244238	-2.4971134720	-3.1321962633
H	5.9116377264	-2.1436965293	-2.3202616431
H	5.0336704424	-3.5673334815	-2.9806122648
H	5.7683002439	-2.3727434445	-4.0922492679

TS3d

0 1

C	0.5605027946	-1.0457066069	0.6668857392
C	1.4974962128	-1.7968894951	1.6765520045
C	2.0732042133	-0.4761779602	2.2530615620
H	2.2123267401	-2.5291900044	1.2871439288
H	0.8256358568	-2.3065615049	2.3770318205
C	0.9794374547	-0.9327470554	-0.7758249492
C	-0.0552118171	-0.8740835793	-1.7541122655
H	-0.9285910886	-1.4958123540	-1.5178062863
H	0.2645469313	-0.8888071693	-2.7992915224
Mg	-2.4466711407	-0.4823762346	0.7313826906
O	-3.9860886660	-1.6225763456	1.5550014757
O	-3.5701712267	-1.2634328733	-0.8608202254
P	-4.4699825556	-1.9596346044	0.1518363038
O	-4.4756341819	-3.5411377617	-0.1471871256
O	-6.0157167940	-1.5349925298	0.0095280777
C	-6.6417628293	-1.6011044740	-1.2591165689
H	-6.6818913463	-2.6392207135	-1.6275736983
H	-7.6642549874	-1.2218076924	-1.1384617091
H	-6.1044943193	-0.9826992631	-1.9945082148
C	-5.0814693754	-4.4219298252	0.7820328457
H	-4.9046916680	-5.4444028291	0.4253736672
H	-4.6397161835	-4.2994825743	1.7826970504
H	-6.1673763399	-4.2436377114	0.8466867985
O	-2.8493450657	1.4229207985	1.1575578355
C	-2.8046856430	2.5302535366	0.5782407261
C	-3.5247082053	3.7541808101	1.1133206645

N	-2.1425210083	2.7932012864	-0.5424498924
C	-3.1951249565	4.8320728914	0.0853715694
H	-3.1552226709	3.9679354911	2.1271424226
H	-4.5957402276	3.5230410332	1.2062509813
C	-2.2779649987	4.1262104536	-0.9223890721
Br	-0.9254686911	1.0202757652	-1.3555148310
H	-4.0749085391	5.2151103631	-0.4514280256
H	-2.6662052349	5.7021405875	0.4991574729
O	-1.7582441264	4.6344032665	-1.8792662506
O	-0.7365128966	-1.2884363115	0.8478420878
C	1.1085656340	0.3185807278	1.3451527925
H	0.2619214104	0.7668378482	1.8801572815
H	1.5441065698	1.0688915037	0.6757384484
C	1.7858617122	-0.2814306948	3.7446504538
H	2.3662299267	-0.9921047185	4.3533463643
H	2.0577728192	0.7371620079	4.0624091752
H	0.7172893405	-0.4388676690	3.9555985201
C	3.5605177290	-0.2680157131	2.0040767217
C	4.4612514789	-1.2825848777	2.3561449714
C	4.0816511033	0.9227112815	1.4904684512
C	5.8334750330	-1.1210189065	2.1873247286
H	4.0795332387	-2.2199889382	2.7708120000
C	5.4552244414	1.0892014167	1.3131807515
H	3.4120931386	1.7388091709	1.2127872584
C	6.3373531738	0.0689870635	1.6610875742
H	6.5141294726	-1.9265956637	2.4728707193
H	5.8358567637	2.0255413790	0.8991594566
H	7.4142329714	0.2043094052	1.5350376341
C	2.3579266994	-0.6735804534	-1.2026868470
C	3.4285329771	-1.5119594892	-0.8660645085
C	2.6346682365	0.4597419098	-1.9997612189
C	4.7261002462	-1.2375543313	-1.2796576549
H	3.2541705394	-2.4244023064	-0.3004984028
C	3.9281265913	0.7702946099	-2.3695984366
H	1.8234628007	1.1340416778	-2.2820861087
C	4.9925679713	-0.0707938331	-2.0035150404
H	5.5233675103	-1.9225695969	-0.9954906605
H	4.1494912402	1.6707389913	-2.9440242789
O	6.2150499308	0.3185675342	-2.3886493501
C	7.3272724053	-0.4703702884	-2.0466595619
H	8.2103218996	0.0523389159	-2.4322136029
H	7.4193145921	-0.5822094922	-0.9537762586
H	7.2722069825	-1.4699202811	-2.5104034599

TS4a

0 1

O	-0.4918203398	-0.5483818971	1.6469778763
C	0.3473209458	-1.5969135236	1.4306006901
C	1.5398198150	-1.8791151918	2.3727405906
C	-0.1086160128	-3.0450813451	1.6962410020
C	1.3588038932	-3.3913236047	2.0830910507
H	1.2192219871	-1.6471537546	3.3987774578
H	2.5062750967	-1.3916271812	2.1892731091
H	-0.7545119994	-3.0311916401	2.5858404397
H	-0.6139569873	-3.6166516643	0.9055641109
C	0.6980233961	-1.2246602300	0.0084464749
C	0.0727172881	-1.8782835335	-1.0980812399
H	0.0197740076	-2.9667998536	-0.9952085765
H	0.3917840985	-1.5484619443	-2.0889795814
Mg	-1.7824431815	0.7125777734	1.0182739280

O	-1.1458343844	2.0378995589	-0.4736722518
O	-1.3940164844	2.5632501642	1.9299535070
P	-1.0720600528	3.1508105145	0.5630522748
O	0.3674786966	3.8734336960	0.6139236957
O	-2.0744954103	4.3340080647	0.1369689630
C	-2.3264940516	5.3956638170	1.0414462882
H	-1.4167818254	5.9940358354	1.2124561469
H	-3.0969073668	6.0325284101	0.5893888469
H	-2.6872831416	5.0095999374	2.0069226609
C	0.9312572552	4.3760485595	-0.5867590109
H	1.9051150823	4.8123878560	-0.3327162830
H	1.0730648816	3.5680625333	-1.3211632698
H	0.2886360841	5.1541222482	-1.0288662513
O	-3.7460845368	0.4681592122	0.9721376202
C	-4.5300322153	-0.0376081408	0.1394297172
C	-6.0272806325	0.1946576361	0.1814696832
N	-4.1777245115	-0.8271658242	-0.8663131491
C	-6.5386953050	-0.5966454995	-1.0189166983
H	-6.2197800961	1.2760533592	0.1301024859
H	-6.4126884569	-0.1539736545	1.1509117512
C	-5.2799193571	-1.2375260150	-1.6162401075
Br	-1.9928450888	-1.3953880493	-1.0674310646
H	-7.2534265714	-1.3909204039	-0.7615709449
H	-7.0130628826	0.0270051707	-1.7900580046
O	-5.2404791093	-1.9703628057	-2.5663601787
C	1.6277007165	-0.1061451463	-0.2060826417
C	2.3898509279	-0.0627313115	-1.3892867418
C	1.8480398387	0.8836348492	0.7681505620
C	3.3283020181	0.9379655055	-1.5942681521
H	2.2798291870	-0.8415709744	-2.1437065038
C	2.7816313979	1.8887848690	0.5598299195
H	1.2395130675	0.8882512922	1.6719055267
C	3.5169049207	1.9223689232	-0.6223758730
H	3.9143040863	0.9539095524	-2.5149056647
H	2.9089440646	2.6669885075	1.3116169562
C	1.5661716829	-4.3393772677	3.2597674628
H	1.2491932494	-5.3631452614	3.0087513166
H	2.6288598678	-4.3782840158	3.5446264270
H	0.9859422327	-4.0068253024	4.1339929071
C	2.1294091626	-3.8738571439	0.8560297547
C	3.2739157113	-3.2341921561	0.3705612150
C	1.6670609493	-5.0030344971	0.1630803285
C	3.9208500870	-3.6884371436	-0.7809815155
H	3.6743329995	-2.3595159436	0.8849242536
C	2.3085336934	-5.4617346391	-0.9831371155
H	0.7768981656	-5.5266992316	0.5224613073
C	3.4384162457	-4.7990095919	-1.4663558549
H	4.8091272092	-3.1642206518	-1.1410036590
H	1.9223459137	-6.3391864886	-1.5066028394
H	3.9402834295	-5.1522374763	-2.3695875826
C	4.4984052983	3.0348760861	-0.8966083946
F	4.1049741027	3.7699033025	-1.9450520531
F	4.6275494064	3.8628237206	0.1416598327
F	5.7110878412	2.5566430451	-1.1877749368

TS4b

0 1

O	-0.5281150000	0.0597230000	1.9778500000
C	-1.5838540000	-0.2876470000	1.2672950000
C	-2.3886300000	-1.7339900000	1.6462330000

C	-2.9190910000	0.4045550000	1.6034770000
C	-3.6845730000	-0.9238740000	1.8503160000
H	-1.8896620000	-2.0420230000	2.5747850000
H	-2.4084130000	-2.5805420000	0.9475790000
H	-2.7674130000	0.9754670000	2.5267550000
H	-3.3079400000	1.0883340000	0.8370160000
C	-1.3581390000	-0.7626230000	-0.0947070000
C	-2.2649730000	-0.3848700000	-1.1761400000
H	-3.2992510000	-0.1857350000	-0.8893090000
H	-2.2112150000	-0.9837290000	-2.0890070000
Mg	0.8779720000	1.2708240000	1.4475200000
O	2.3313270000	0.5649640000	0.0934980000
O	2.6343530000	1.0003080000	2.5103870000
P	3.3476400000	0.5941010000	1.2258880000
O	4.0899260000	-0.8168760000	1.4484320000
O	4.5427830000	1.5995870000	0.8394590000
C	5.4838390000	1.9780680000	1.8292000000
H	6.0789380000	1.1123640000	2.1638080000
H	6.1525580000	2.7201350000	1.3756470000
H	4.9782830000	2.4201180000	2.7010370000
C	4.8765040000	-1.3476830000	0.3943770000
H	5.1096140000	-2.3880090000	0.6496500000
H	4.3277730000	-1.3222900000	-0.5594480000
H	5.8145780000	-0.7822420000	0.2783340000
O	0.5092090000	3.1138150000	0.9750580000
C	0.3963760000	3.9049120000	-0.0003870000
C	0.9456690000	5.3200850000	0.0741370000
N	-0.1876900000	3.6335740000	-1.1498120000
C	0.6032880000	5.8890890000	-1.2966860000
H	2.0225150000	5.2731590000	0.2935830000
H	0.4695950000	5.8452130000	0.9156950000
C	-0.1439700000	4.7403840000	-1.9918750000
Br	-1.3542800000	1.4170930000	-1.3961710000
H	-0.0467040000	6.7751720000	-1.2693680000
H	1.4836110000	6.1532780000	-1.9002700000
O	-0.6206530000	4.7907870000	-3.0965900000
C	-4.2372600000	-1.0472090000	3.2743150000
H	-5.0623690000	-0.3348610000	3.4261910000
H	-4.6259060000	-2.0608650000	3.4563400000
H	-3.4540180000	-0.8330210000	4.0172710000
C	-4.7984780000	-1.2275620000	0.8581350000
C	-5.0324540000	-2.5276030000	0.3974580000
C	-5.6648490000	-0.2111930000	0.4361320000
C	-6.0822990000	-2.8012780000	-0.4786150000
H	-4.3915020000	-3.3486450000	0.7280410000
C	-6.7147620000	-0.4797540000	-0.4398120000
H	-5.5182480000	0.8107840000	0.7948310000
C	-6.9241060000	-1.7767780000	-0.9061400000
H	-6.2419650000	-3.8235280000	-0.8285660000
H	-7.3724410000	0.3310170000	-0.7604140000
H	-7.7428720000	-1.9883170000	-1.5968010000
C	-0.1424870000	-1.5419710000	-0.4053090000
C	0.3077280000	-1.6644020000	-1.7318480000
C	0.5814020000	-2.1935230000	0.6079500000
C	1.4199110000	-2.4323180000	-2.0368830000
H	-0.1843180000	-1.1212060000	-2.5384090000
C	1.6800120000	-2.9846220000	0.3004470000
H	0.2904330000	-2.0712300000	1.6489630000
C	2.0942720000	-3.1129220000	-1.0222840000
H	1.7635530000	-2.5027090000	-3.0700460000

H	2.2241770000	-3.4878880000	1.0993080000
C	3.2523290000	-4.0066120000	-1.3942390000
F	3.9071550000	-4.4603880000	-0.3221790000
F	4.1380480000	-3.3660070000	-2.1654510000
F	2.8317840000	-5.0706390000	-2.0844950000

TS4c

0 1

O	0.2099257810	0.0504322824	-2.2408620742
C	-0.8904252352	-0.1597966344	-1.5111186238
C	-1.6240645148	1.0829882365	-0.9322659241
C	-2.2635584952	-0.4475228671	-2.3471211447
C	-2.9675343855	0.7141784427	-1.6080076171
H	-1.1730964501	1.9811488288	-1.3705895361
H	-1.6213376222	1.1953361918	0.1545815491
H	-1.9841934674	-0.2248441589	-3.3852141699
H	-2.7349120510	-1.4401867260	-2.3028290787
C	-3.5020153865	1.8006643154	-2.5450809415
H	-4.3599856756	1.4322909676	-3.1285698719
H	-3.8366968592	2.6742336706	-1.9649062082
H	-2.7186205671	2.1305491947	-3.2440777396
C	-4.0823399031	0.2767530321	-0.6662758462
C	-4.2285704179	0.8376534740	0.6059577972
C	-5.0393276856	-0.6556852486	-1.0863422280
C	-5.2747542242	0.4573819800	1.4453385706
H	-3.5068700555	1.5774756404	0.9588190409
C	-6.0882279919	-1.0385684370	-0.2531902121
H	-4.9657794078	-1.0941402125	-2.0854977125
C	-6.2056578153	-0.4886493877	1.0226866391
H	-5.3525125950	0.8930760228	2.4434047242
H	-6.8183773844	-1.7720325752	-0.6031095474
C	-0.8882335079	-1.4205025658	-0.7171844110
C	-0.2000285529	-2.5458172944	-1.2605997127
H	0.0249496291	-2.4818879312	-2.3288189261
H	-0.4934450825	-3.5305463694	-0.8837398661
C	-1.6205347259	-1.5539898145	0.5514979146
C	-1.2503095428	-0.7621366396	1.6501509687
C	-2.6874485596	-2.4506549412	0.6590322468
C	-1.9701790676	-0.8580995693	2.8321917360
H	-0.3924627297	-0.0866741254	1.5633310480
C	-3.4336406025	-2.5079702772	1.8309628781
H	-2.9730596086	-3.0679188467	-0.1950662420
C	-3.0782291864	-1.7058315337	2.9111258572
H	-1.6840946644	-0.2495171891	3.6920455523
H	-4.3022030783	-3.1628295876	1.8958928732
Mg	1.7568037098	0.7640508866	-1.3336183997
O	1.1325453872	1.2186922455	0.6704546589
O	1.7066060695	2.8430795966	-1.1112363719
P	1.3024614584	2.6983493437	0.3472702518
O	-0.0389076722	3.5527086532	0.6118200867
O	2.3697755410	3.3357246995	1.3654426945
C	2.8626561874	4.6457548378	1.1356049307
H	2.0586061171	5.3931233656	1.2326178760
H	3.6304146879	4.8411749337	1.8942647205
H	3.3062699562	4.7251038015	0.1317508389
C	-0.6892119258	3.4748258803	1.8653245491
H	-1.5214243234	4.1896236194	1.8473166558
H	-1.0862768897	2.4616723221	2.0434556097
H	-0.0042387029	3.7350172309	2.6882464764
O	3.6713984905	0.3004335690	-1.2668120476

C	4.3435846322	-0.4771295764	-0.5464722509
C	5.8332455531	-0.2905648545	-0.3320192629
N	3.8669546704	-1.5293100096	0.1003387713
C	6.1914728673	-1.4306534787	0.6156112199
H	6.0116989500	0.7156605373	0.0738480476
H	6.3403342622	-0.3376294594	-1.3072308978
C	4.8731348584	-2.1945303428	0.7995907101
Br	1.6543434458	-2.1158050339	-0.3944787468
H	6.9523740698	-2.1205987520	0.2247645222
H	6.5393958498	-1.0912773468	1.6019102359
O	4.7262082215	-3.2011129119	1.4388701832
H	-7.0173112407	-0.7958396416	1.6852787060
C	-3.8863196928	-1.7320692308	4.1847178741
F	-4.1785968254	-0.4901935637	4.5947921562
F	-3.2166720003	-2.3216943698	5.1785573802
F	-5.0411092730	-2.3843141075	4.0352366296

TS4d
0 1

C	0.8434333826	-1.0713207222	0.3752853772
C	1.8592044127	-1.9471574286	1.1819654839
C	2.5848880085	-0.7350056289	1.8164647665
H	2.4776622076	-2.6509692570	0.6162750505
H	1.2538844348	-2.5082406439	1.9029340708
C	1.1210949015	-0.7347930753	-1.0378812556
C	0.0072399416	-0.4144952471	-1.8913704485
H	-0.8746923067	-1.0504492125	-1.7207601620
H	0.2594792441	-0.2645599230	-2.9449361074
Mg	-2.1369072550	-0.4189113606	0.7690433661
O	-3.6258594582	-1.7432656347	1.3599810740
O	-3.1898593119	-1.0024355037	-0.9628010660
P	-4.0595696067	-1.9001092217	-0.0908468250
O	-3.9439869168	-3.4150032106	-0.6207866761
O	-5.6268127527	-1.5633703652	-0.2134691811
C	-6.2253295152	-1.4777731794	-1.4948379073
H	-6.1915195837	-2.4499190358	-2.0131562950
H	-7.2719277084	-1.1843179425	-1.3463075924
H	-5.7166513506	-0.7241992964	-2.1153348994
C	-4.5131508695	-4.4645552278	0.1424069082
H	-4.2562618444	-5.4070896130	-0.3569580212
H	-4.1085395498	-4.4678246335	1.1659590458
H	-5.6099983277	-4.3680185312	0.1918697452
O	-2.4658030791	1.3529246128	1.5757390671
C	-2.3972160949	2.5652611587	1.2564087291
C	-3.0276430804	3.6522464722	2.1083922641
N	-1.7888857073	3.0491513080	0.1850785924
C	-2.7052099449	4.9265964517	1.3358110266
H	-2.5918881463	3.6117045472	3.1177199042
H	-4.1016414339	3.4418456081	2.2165333181
C	-1.8840117886	4.4374866926	0.1339922463
Br	-0.7021841013	1.3766328390	-1.1248754348
H	-3.5939965758	5.4547603051	0.9615349854
H	-2.1104391409	5.6581216278	1.9008229819
O	-1.3972376221	5.1376551006	-0.7137819279
O	-0.4233306608	-1.2518262884	0.6959145433
C	1.5818171317	0.2156663896	1.1334620419
H	0.8033113535	0.6090954721	1.7976065207
H	1.9756702315	1.0277262879	0.5139595852
C	2.4900198629	-0.6938002154	3.3443326235
H	3.0910044603	-1.5000582973	3.7922518039

H	2.8688830771	0.2648919092	3.7306868006
H	1.4467017753	-0.8160092576	3.6726084927
C	4.0426578548	-0.5848866855	1.4018491810
C	4.8951442437	-1.6948878372	1.4714226842
C	4.5891207606	0.6388258152	1.0074588444
C	6.2392386183	-1.5944947886	1.1233061309
H	4.4987362419	-2.6605789645	1.7979229745
C	5.9350684200	0.7448176038	0.6554876170
H	3.9602634759	1.5305587826	0.9608255548
C	6.7641725785	-0.3726207165	0.7025279581
H	6.8811493657	-2.4767307517	1.1777980233
H	6.3339925547	1.7075204634	0.3301070290
H	7.8132336297	-0.2920205363	0.4115378824
C	2.4788868996	-0.5305228854	-1.5821109429
C	3.4297362859	-1.5551633595	-1.6020585689
C	2.8200578455	0.7283765968	-2.1089428444
C	4.7101681803	-1.3201145693	-2.0926774927
H	3.1743045537	-2.5516963925	-1.2453862182
C	4.1046769788	0.9691434893	-2.5707190665
H	2.0830978638	1.5343793941	-2.1110355353
C	5.0559783977	-0.0534965634	-2.5489067252
H	5.4492598714	-2.1209544284	-2.0867803743
H	4.3727308963	1.9617101950	-2.9368816940
C	6.4547703968	0.2378113747	-3.0338453940
F	7.3043934108	-0.7476985068	-2.7388586475
F	6.9329551278	1.3599505679	-2.4836219633
F	6.4835601861	0.4104625934	-4.3588175476

TS5a
0 1

O	-0.4964995793	-0.6752142194	1.7456686448
C	-1.6989367148	-0.5689698650	1.1157137117
C	-2.8276876109	-1.5754442326	1.5378268646
C	-2.6245757786	0.5601216200	1.6411491840
C	-3.8373761907	-0.4084887930	1.6838133872
H	-2.5547248289	-1.9975840596	2.5153703473
H	-3.0799766872	-2.3977359729	0.8539771196
H	-2.2771870515	0.7941054168	2.6561735173
H	-2.7110060643	1.5014879324	1.0808644740
C	-4.6343970764	-0.4026623786	2.9900146545
H	-5.1926762413	0.5395123667	3.1070388006
H	-5.3631565981	-1.2283646097	3.0070632224
H	-3.9634429514	-0.5150971699	3.8555787123
C	-4.8040426539	-0.2347218799	0.5192796227
C	-5.2999114470	-1.3190058496	-0.2106291075
C	-5.2804165688	1.0431180875	0.1958653538
C	-6.2201126621	-1.1341775047	-1.2429010132
H	-4.9663723800	-2.3313717959	0.0256283468
C	-6.1978568900	1.2350887372	-0.8337524498
H	-4.9218628664	1.9083282842	0.7600865793
C	-6.6691915027	0.1442440570	-1.5640275076
H	-6.5868864172	-1.9991236430	-1.8007342211
H	-6.5463964663	2.2435716180	-1.0686442864
C	-1.4795719318	-0.5867983799	-0.4122454571
C	-1.9469682239	0.3953596168	-1.2495135114
H	-2.6694090279	1.1176027236	-0.8766313886
H	-1.8579976546	0.3163214906	-2.3333570301
Mg	1.2290905772	-0.5142023852	1.0102604900
O	2.5182510991	-0.5902841693	-0.7547374916
O	2.5299796272	-2.1161284376	1.2009750876

P	3.1872001336	-1.8038283785	-0.1359598248
O	3.0902158520	-3.1138506243	-1.0757746165
O	4.7693086316	-1.5358881729	-0.0057634154
C	5.5711720135	-2.4478157703	0.7275681838
H	5.5938386710	-3.4355794851	0.2393360487
H	6.5880447659	-2.0371112190	0.7566655561
H	5.1902577587	-2.5655075593	1.7532187855
C	3.5737543761	-3.0493147789	-2.4062214907
H	3.3400714780	-4.0079942301	-2.8857171975
H	3.0886615912	-2.2313518971	-2.9620418332
H	4.6640869819	-2.8914915178	-2.4231931730
O	2.2817986531	1.1720108359	1.5603052284
C	2.5725374509	2.1567086442	0.8894684691
C	3.8375184189	2.9668231735	1.0364286020
N	1.8180560897	2.6817966274	-0.1063129499
C	3.7071466303	4.0802226995	-0.0031765697
H	4.6835062507	2.2875662737	0.8554732317
H	3.9191350938	3.3192710236	2.0741783733
C	2.3940756080	3.8057510733	-0.7248859228
Br	0.1732199551	1.8373261280	-0.6487097972
H	3.6558909147	5.0872803046	0.4346079681
H	4.5171597847	4.0906950879	-0.7453347879
O	1.9113298222	4.4082146978	-1.6364198593
H	-7.3852245715	0.2908984552	-2.3756011061
N	-0.6111266955	-1.5614787880	-0.8531698267
C	-0.5545883683	-2.8787950959	-0.2201131566
H	-0.6529222849	-2.7901737201	0.8657572232
H	-1.3450784479	-3.5428223828	-0.6139202825
H	0.4235786935	-3.3325730019	-0.4321789659
C	-0.2076786362	-1.5619262545	-2.2448536455
H	0.1834820845	-0.5756568700	-2.5288162424
H	0.6036665408	-2.2890557463	-2.3706392383
H	-1.0390594777	-1.8324571367	-2.9224274241

TS5b
0 1

O	0.0182896017	-1.0374450131	1.4931805717
C	-1.0783888211	-0.8039737271	0.8682835491
C	-2.5169674894	-2.0636595714	0.7703726606
C	-2.1547504347	0.0399104587	1.5471769044
C	-3.3346019690	-0.9615540291	1.4651082014
H	-2.0936611987	-2.8117583534	1.4514175740
H	-2.9384154358	-2.5593749440	-0.1125984072
H	-1.8264239879	0.2171176719	2.5781161683
H	-2.2899690849	1.0189582387	1.0665665772
C	-3.8052392806	-1.4081848197	2.8568704032
H	-4.2826200590	-0.5695087381	3.3859557987
H	-4.5400026413	-2.2237651744	2.7770903801
H	-2.9550339755	-1.7604398959	3.4610094156
C	-4.5486061359	-0.4784732673	0.6787437309
C	-5.3469970653	-1.3771007973	-0.0386521972
C	-4.9415516931	0.8646421056	0.7195446919
C	-6.4848518134	-0.9447132592	-0.7175230555
H	-5.0849969478	-2.4377708696	-0.0670250334
C	-6.0781037505	1.3018072288	0.0412139918
H	-4.3523568132	1.5886850670	1.2870935335
C	-6.8517833944	0.3993233458	-0.6857507818
H	-7.0885783867	-1.6644706264	-1.2747126535
H	-6.3579557715	2.3565999879	0.0810770066
C	-1.1941995939	-1.1058394891	-0.5369658063

C	-1.9369014341	-0.1284808010	-1.4000741314
H	-2.8760386285	0.1979617919	-0.9467922635
H	-2.1487979256	-0.4983933474	-2.4073233707
Mg	1.7220101010	-0.1761588026	0.9889530766
O	2.6531903759	-0.9785671368	-0.7207800119
O	3.3696891270	-1.2773214108	1.6259246720
P	3.7293133815	-1.5756983302	0.1772531370
O	3.8761419405	-3.1707552664	-0.0184506926
O	5.1671723405	-1.0021874338	-0.2467121158
C	6.2813231054	-1.1851902394	0.6124982497
H	6.5649191381	-2.2487276312	0.6711909492
H	7.1146529451	-0.6121175561	0.1881279600
H	6.0572822892	-0.8179684709	1.6250622731
C	4.2546932737	-3.6901568338	-1.2803503638
H	4.2635936293	-4.7838425398	-1.1941646883
H	3.5387660777	-3.3903092210	-2.0628798838
H	5.2577274242	-3.3393736675	-1.5702133306
O	1.8366425866	1.7048968800	1.3010927243
C	1.8185775954	2.8501080238	0.7519024928
C	2.4296181434	4.0441205942	1.4708411491
N	1.3001127724	3.1291946923	-0.4174659117
C	2.2187715631	5.1703000471	0.4695965230
H	3.4823720869	3.8275656254	1.7056580676
H	1.9122076993	4.1890980276	2.4314646713
C	1.4512510311	4.4868613299	-0.6779271301
Br	-0.7263124728	1.4084514141	-1.4599735766
H	1.6246583011	6.0132189329	0.8504004706
H	3.1535374718	5.5896785748	0.0694384970
O	1.0406383891	5.0578810432	-1.6574600634
H	-7.7390933682	0.7413972922	-1.2221976628
N	-0.3687858482	-1.9911352217	-1.1352791473
C	0.2431373258	-3.1129329553	-0.4367050519
H	0.0963467262	-3.0421247638	0.6424017639
H	-0.1982824213	-4.0527201023	-0.8099383018
H	1.3249095648	-3.1215489231	-0.6224240639
C	-0.2053507261	-2.0429893540	-2.5782132621
H	-0.1610956708	-1.0368900391	-3.0121698041
H	0.7636023183	-2.5140344733	-2.7843704964
H	-0.9961150869	-2.6347352773	-3.0725185767

TS5c

0 1

O	-0.1445715782	-0.3905741650	1.0725377774
C	-1.5210616503	-0.0942567145	1.1105103210
C	-2.5115344629	-0.4984317494	2.2016887680
C	-2.1071059778	1.3142336488	1.1360309816
C	-3.4024928910	0.6759314634	1.7166950365
H	-2.0849660328	-0.2704290533	3.1898119582
H	-2.9240154289	-1.5119931711	2.2189003638
H	-1.5768551843	1.9127292822	1.8926298227
H	-2.1729776620	1.8917719292	0.2068404793
C	-4.1457615230	1.4535889717	2.7967158628
H	-4.6087915743	2.3625997240	2.3845895663
H	-4.9466084865	0.8389210709	3.2353635376
H	-3.4593290261	1.7556595476	3.6023426735
C	-4.3347434982	0.2922376293	0.5676048132
C	-4.7492172187	-1.0185087041	0.3145841668
C	-4.7990942150	1.3029093930	-0.2880066157
C	-5.5915901615	-1.3128558337	-0.7594211472
H	-4.4141318785	-1.8363343379	0.9532609303

C	-5.6400110823	1.0155539941	-1.3587768234
H	-4.4907080135	2.3379180344	-0.1168279245
C	-6.0386433956	-0.2995349410	-1.6016875591
H	-5.8976824720	-2.3465272804	-0.9356445827
H	-5.9832635357	1.8225032132	-2.0098252356
C	-1.1856997682	-0.8594718474	-0.0914332335
C	-1.0586008137	-0.1009356876	-1.4062225331
H	-2.0606798230	0.2518687154	-1.6922219008
H	-0.6570463405	-0.7332077555	-2.1985676578
Mg	1.7496978830	0.0079705573	0.5744434978
O	2.0176117034	-1.4447359885	-0.9503900383
O	2.6832688312	-1.6744225076	1.4187407012
P	2.7155430688	-2.3499465386	0.0590385310
O	2.0303666860	-3.8105441976	0.1613911239
O	4.2040810947	-2.6694700206	-0.4532593553
C	5.1656461253	-3.1910621534	0.4506633961
H	4.9072360895	-4.2186953922	0.7552388753
H	6.1292758032	-3.2022652124	-0.0729921999
H	5.2386496284	-2.5604033501	1.3489375064
C	2.1379073136	-4.7259501802	-0.9141421680
H	1.4464626201	-5.5530578932	-0.7078065490
H	1.8647191823	-4.2579149946	-1.8732755102
H	3.1615619082	-5.1227901535	-0.9953882014
O	2.7076565366	1.6006106189	1.0731352451
C	2.8012594507	2.7714296261	0.5864087023
C	3.7265248740	3.7955305571	1.2223638309
N	2.1477020630	3.2069278499	-0.4617489131
C	3.5343586121	5.0087412268	0.3229833087
H	4.7512300871	3.3956330945	1.2413723977
H	3.4268387650	3.9528815463	2.2695001496
C	2.4877074041	4.5342312902	-0.7032939988
Br	0.1386156221	1.4278667511	-1.3368120016
H	3.1521060740	5.8999953273	0.8408670373
H	4.4441100154	5.3142686457	-0.2140478583
O	2.0342626218	5.2221249343	-1.5828196665
H	-6.6948211060	-0.5302766824	-2.4434845539
N	-1.3325665309	-2.2196714219	-0.1893538712
C	-1.2060494437	-3.0702538754	0.9878818134
H	-1.0375456429	-2.4784191820	1.8898599168
H	-2.0996241314	-3.7014653347	1.1231752166
H	-0.3182032697	-3.7119941213	0.8788958555
C	-1.0290079387	-2.9109500805	-1.4353489066
H	0.0273926406	-2.7922498940	-1.7280576964
H	-1.2341647375	-3.9781469513	-1.2839611388
H	-1.6817402092	-2.5786412756	-2.2544873239

Real Model with Real Catalyst

(2R,4R)-CYr
0 1

O	-2.3331166078	-1.7724600244	0.2418225517
C	-3.2453540315	-1.0327399451	-0.0883920137
C	-5.2470441729	-0.1929619605	-1.1029416603
C	-3.3285233808	0.4396268306	0.1445231470
C	-4.8219556901	0.7947334979	0.0120743560
H	-4.9283433348	0.2236748345	-2.0699161225
H	-6.3314652331	-0.3521004334	-1.1698298658
H	-2.7725692734	0.8565647298	-0.7226376242
H	-2.7820122360	0.7945310375	1.0290062958
C	-4.4728057947	-1.5201570907	-0.8700503676

C	-5.3149024536	-2.4344065369	0.0400619760
H	-5.5183041504	-1.9465561961	0.9998208178
H	-6.2792136947	-2.6455845958	-0.4370319892
O	0.4815640634	-2.1791079690	2.4736988557
C	0.0565638321	-1.1680594157	3.0655668632
C	0.1835495618	-0.8549518668	4.5381905823
N	-0.6104564345	-0.2281554081	2.3807924890
C	-0.6051124735	0.4472449820	4.6610698607
H	-0.2147025938	-1.6923953851	5.1278794778
H	1.2487496721	-0.7521838340	4.7894501857
C	-1.0880827636	0.7341483911	3.2357414786
Br	-4.5339236796	-4.1596677568	0.4781426530
H	0.0017094816	1.2963007391	5.0030254943
H	-1.4779087102	0.3798840651	5.3254786135
O	-1.8187890251	1.6538649597	2.9246114696
C	-5.0370762123	2.2467481168	-0.4261599453
H	-4.7564127423	2.9413780970	0.3798319248
H	-6.0922811749	2.4314365050	-0.6749558268
H	-4.4217483767	2.4904799782	-1.3127775413
C	-5.6043789684	0.5556558850	1.3119638735
C	-7.0047175123	0.4717565777	1.2725776714
C	-4.9801732147	0.4613286785	2.5605705456
C	-7.7517489556	0.2652485716	2.4281753194
H	-7.5304390926	0.5686845114	0.3194982503
C	-5.7272411478	0.2526567585	3.7215282499
H	-3.9000161976	0.5752330523	2.6514564194
C	-7.1129453745	0.1444852257	3.6623159192
H	-8.8400170644	0.1984316091	2.3643230171
H	-5.2118895624	0.1801926308	4.6817257686
H	-7.6946805459	-0.0234458032	4.5708956019
C	-4.0852961411	-2.1378638892	-2.2111015400
C	-4.8864950367	-3.1002704416	-2.8329083026
C	-2.9710377179	-1.6465356693	-2.9060286954
C	-4.5876739885	-3.5568794811	-4.1158158811
H	-5.7582246083	-3.5122338741	-2.3219245182
C	-2.6747452536	-2.1004786775	-4.1891776272
H	-2.3009886370	-0.9044019046	-2.4549851288
C	-3.4827453066	-3.0573829504	-4.8006415498
H	-5.2272540472	-4.3102723305	-4.5805599803
H	-1.7989876637	-1.7000746661	-4.7045288326
H	-3.2511709105	-3.4140647991	-5.8062666989
Mg	-0.3060024274	-1.3814475706	0.5943884627
O	-0.3038310612	-0.2680384286	-1.0892696431
P	1.1647908468	-0.0721010583	-1.4542374876
O	1.7796482750	-0.5023330634	-2.7220550096
O	1.6747460503	1.4376872777	-1.0833401007
O	1.7694405560	-0.8839159589	-0.0851327176
C	1.5588696830	1.9673641774	0.1787625982
C	3.0998074666	-0.7098157536	0.2535387154
C	2.3866391058	1.4804500155	1.1803663057
C	0.6492089478	3.0404084671	0.4039870063
C	3.4431996085	0.4847338798	0.8552914347
C	4.0468988636	-1.7150198258	-0.0677232580
C	2.2272380080	1.9745479817	2.5161114475
C	0.5838251322	3.5824110367	1.6666812097
Si	-0.3407912351	3.9264025365	-0.9417830000
C	4.8318687812	0.7591087121	1.0936085627
C	5.3697377648	-1.4749463497	0.2201926077
Si	3.6165348462	-3.3451751027	-0.9090694531
C	2.9269418027	1.4250713774	3.6283813594

C	1.3385684579	3.0591310345	2.7499441635
H	-0.0675274830	4.4377477806	1.8833803506
C	-0.8438411656	2.8551782570	-2.3906437097
C	-1.8683936578	4.6930315734	-0.1616614665
C	0.7347700087	5.3215382709	-1.5965129518
C	5.2973518699	2.0127583108	1.5821468297
C	5.7961118198	-0.2447523271	0.7911209232
H	6.1385921751	-2.2210654108	-0.0153945802
C	4.4904064685	-3.3860250642	-2.5592302782
C	4.1890110743	-4.7762816145	0.1654659136
C	1.7625767290	-3.5196359893	-1.0700187606
C	2.7886206825	1.9625507127	4.8855364502
H	3.5828046180	0.5672493886	3.4737002828
C	1.2222722254	3.5996326140	4.0592302244
C	-2.1741460704	2.4879908835	-2.6398976092
C	0.1572696168	2.4267721763	-3.2814645217
C	-2.3871156841	5.8825825029	-0.7023356906
C	-2.4896603622	4.1390195135	0.9691657831
C	1.7332817293	5.9090879072	-0.8037272835
C	0.5261860580	5.8156968137	-2.8961724598
C	6.6354667949	2.2357976414	1.7954382425
H	4.5798674381	2.8096705393	1.7771256592
C	7.1732894383	0.0133370633	1.0333998975
C	5.4523103615	-4.3565602787	-2.8833627344
C	4.1943552637	-2.3824331895	-3.4988328679
C	3.9771202217	-6.0822990958	-0.3138709586
C	4.7762026262	-4.6082258788	1.4278017940
C	1.0091188699	-3.6537059635	0.1155552248
C	1.1019543899	-3.5045246599	-2.3028798928
C	1.9394288828	3.0739835483	5.1055670367
H	3.3377123385	1.5269887489	5.7230871102
H	0.5390458773	4.4371648396	4.2189811740
C	-2.4995084934	1.6960101811	-3.7472944216
H	-2.9817220540	2.8197728969	-1.9729228011
C	-0.1646402475	1.6216033972	-4.3787082835
H	1.2065700234	2.7077538667	-3.1192873503
C	-3.4902005363	6.5125543834	-0.1154637795
H	-1.9318594759	6.3388185070	-1.5857160204
C	-3.5815332283	4.7790783376	1.5677146292
H	-2.1301970794	3.1972411651	1.4113208993
C	2.5042917888	6.9689706016	-1.2963286024
H	1.9284189151	5.5458292406	0.2077595474
C	1.2957439067	6.8746530800	-3.3890426856
H	-0.2362134029	5.3741503698	-3.5436313954
C	7.5873608751	1.2227653776	1.5314189978
H	6.9684763321	3.2078536310	2.1648926581
H	7.8997001837	-0.7699770260	0.8027645201
C	6.0985116738	-4.3330317054	-4.1244773517
H	5.7144246024	-5.1425923516	-2.1718243042
C	4.8411398970	-2.3599832321	-4.7393687351
H	3.4513967814	-1.5965140110	-3.2775537176
C	4.3594990281	-7.1916005956	0.4467042329
H	3.5052249670	-6.2479740394	-1.2868965811
C	5.1595789892	-5.7187977202	2.1904043229
H	4.9372518629	-3.6097257558	1.8398712005
C	-0.3903534296	-3.7647524872	0.0599986820
H	1.5095012992	-3.7090413056	1.0957210544
C	-0.2956653612	-3.6130068430	-2.3600996420
H	1.6607335660	-3.3910989799	-3.2379787050
H	1.8441466163	3.4968683337	6.1074642142

C	-1.4942671872	1.2588613639	-4.6147199951
H	-3.5341041640	1.4204391421	-3.9359650571
H	0.6271243864	1.2646011590	-5.0371621952
C	-4.0835505428	5.9656288773	1.0262788068
H	-3.8805296270	7.4329918615	-0.5435099454
H	-4.0350072829	4.3482704036	2.4594090276
C	2.2859132415	7.4532472729	-2.5887614092
H	3.2763610293	7.4134366357	-0.6722144949
H	1.1262935708	7.2459895780	-4.3972614468
H	8.6476587838	1.4119568410	1.7093323014
C	5.7921602738	-3.3348373580	-5.0542316267
H	6.8402316202	-5.0906649576	-4.3647375983
H	4.6018038956	-1.5773494958	-5.4568240108
C	4.9541788273	-7.0106125655	1.6999142408
H	4.1916526957	-8.1964044181	0.0651117735
H	5.6154651856	-5.5738724391	3.1673214003
C	-1.0394761813	-3.7325510074	-1.1888611293
H	-0.9710499085	-3.9737656043	0.9684973152
H	-0.7976416283	-3.6014217358	-3.3264893031
H	-1.7456177914	0.6389474487	-5.4727823363
H	-4.9315353562	6.4630796710	1.4922777492
H	2.8858260727	8.2757933169	-2.9724704388
H	6.2944064197	-3.3157315377	-6.0191659061
H	5.2523484329	-7.8737258720	2.2914844174
H	-2.1290833165	-3.8108442955	-1.2317149835

(2R,4S)-CYr
0 1

C	-3.4538414764	-1.2797112173	-0.4637782937
C	-2.9509993807	-2.1282843069	0.6558475463
C	-4.1873956866	-2.5973726496	1.4390883527
H	-2.1340630702	-1.6490233726	1.2157901931
H	-2.4658964031	-2.9796813549	0.1424070389
C	-4.9782644023	-1.3631263950	-0.5594253258
C	-5.4316221814	-1.4717377190	-2.0197926693
H	-5.0964571755	-0.5957493596	-2.5862697200
H	-6.5241828960	-1.5471724811	-2.0758070318
Mg	-0.7150969929	-0.6374431613	-1.7793407749
O	-0.2772897818	0.6786696486	-0.0713290851
O	1.3320614154	-0.2608158208	-1.7033141360
O	-0.7159481546	-2.7752768773	-1.3432748941
C	-0.8872274076	-3.0063179571	-2.5588662211
C	-0.9993665041	-4.3650539117	-3.2122207853
N	-0.9952474987	-1.9951182164	-3.4263282524
C	-1.2684321760	-4.0042182379	-4.6729488824
H	-1.7963847323	-4.9449337412	-2.7259049363
H	-0.0538615249	-4.9084114249	-3.0529918365
C	-1.1984917966	-2.4687714449	-4.7065731013
Br	-4.7232299607	-3.0208519816	-2.9750314391
H	-0.5334140810	-4.4153125328	-5.3781569033
H	-2.2636116245	-4.3134630632	-5.0239695707
O	-1.3110707311	-1.7871583154	-5.6973524919
O	-2.7715113681	-0.6548101111	-1.2586659940
C	-5.2585699866	-2.6023594256	0.3133365753
H	-5.1083458333	-3.5008980121	-0.3044296129
H	-6.2822271346	-2.6507201363	0.7021352646
C	-3.9720245340	-4.0048148842	2.0087068969
H	-3.1968431757	-3.9922963348	2.7892309524
H	-4.8918367641	-4.4085644875	2.4545508094
H	-3.6548315556	-4.6977381860	1.2143244584

C	-4.6075197346	-1.6282287106	2.5518867748
C	-3.9655155323	-0.4097523364	2.7870647184
C	-5.7294382151	-1.9350744643	3.3358434394
C	-4.4384762593	0.4786080546	3.7533687547
H	-3.0942122274	-0.1132506142	2.2007896495
C	-6.2004272334	-1.0560070349	4.3053605884
H	-6.2645893080	-2.8746276633	3.1764626282
C	-5.5575944200	0.1637919337	4.5158759614
H	-3.9222325862	1.4354592953	3.8911093932
H	-7.0794751520	-1.3225527715	4.8964765314
H	-5.9291230520	0.8622596242	5.2683718711
C	-5.5935014874	-0.0524050400	-0.0179794112
C	-4.9614467316	1.1808818474	-0.2182943377
C	-6.8235553136	-0.0656691049	0.6485408901
C	-5.5240811486	2.3588672840	0.2695076863
H	-4.0060972530	1.2354166108	-0.7431784905
C	-7.3868363038	1.1104849258	1.1345769322
H	-7.3524323872	-1.0037169081	0.8158248666
C	-6.7348205247	2.3285288689	0.9552737781
H	-4.9966267054	3.3059569910	0.1234432653
H	-8.3396198418	1.0694843199	1.6658463168
H	-7.1692430596	3.2499719330	1.3477606154
P	1.1804042629	0.6323903140	-0.4884261095
O	2.0892373117	0.2216786642	0.7858872988
O	1.8526199770	2.0623996747	-0.8655593749
C	3.4629396371	0.3043872713	0.6322180098
C	2.1016834671	2.9685855128	0.1422486270
C	4.0505727190	1.5552406603	0.7019191530
C	4.1901407453	-0.8889154505	0.3862211360
C	3.1916583245	2.7435757945	0.9641083101
C	1.2226019213	4.0695615772	0.2953946442
C	5.4571273762	1.6628254392	0.4447527166
C	5.5496959000	-0.7852432123	0.2060012908
Si	3.3819147401	-2.6001993101	0.3615413190
C	3.4084234624	3.6334582211	2.0663581602
C	1.4687840848	4.9536684318	1.3202769271
Si	-0.3103841366	4.3299606926	-0.7816476436
C	6.1310465649	2.9140146613	0.3674021597
C	6.2098855247	0.4752179193	0.2157310211
H	6.1640471487	-1.6771411823	0.0331704074
C	1.8411462203	-2.6568732896	1.4214615178
C	3.0477393665	-3.2363570626	-1.3663008563
C	4.6504145964	-3.7515509203	1.1488131887
C	4.4264431966	3.4185464718	3.0378475032
C	2.5491363280	4.7598085227	2.2256876144
H	0.8136099109	5.8178319354	1.4856973053
C	-1.7454167131	4.4041981144	0.4180084089
C	-0.1461731743	5.9608381154	-1.6951767287
C	-0.5648434325	3.0016104682	-2.0694671228
C	7.4806433677	2.9754459400	0.1201760947
H	5.5622651253	3.8346077720	0.4998978900
C	7.6076450441	0.5724643497	-0.0260513974
C	0.7753568820	-3.5111683130	1.0987246401
C	1.7747489867	-1.9130480822	2.6134915946
C	2.9114948129	-2.3962682754	-2.4794935713
C	2.9029209356	-4.6266843893	-1.5452030637
C	4.6474455705	-3.9918469567	2.5317614772
C	5.6393702563	-4.3647044559	0.3599881961
C	4.6035134642	4.2936447694	4.0811695807
H	5.0678684925	2.5409981571	2.9509354129

C	2.7657424517	5.6519306410	3.3119177772
C	-2.5339531363	5.5518346974	0.5873305503
C	-1.9798057095	3.2840199110	1.2362691474
C	-1.1882724710	6.3389845134	-2.5617967083
C	0.9689440705	6.8023243398	-1.5714987891
C	0.4370040008	2.8504986174	-3.0504614386
C	-1.6711235680	2.1421708310	-2.0934843229
C	8.2341513419	1.7926453332	-0.0673081406
H	7.9756915292	3.9470114654	0.0623936903
H	8.1747061220	-0.3479824887	-0.1874202570
C	-0.3024804245	-3.6675148143	1.9794684255
H	0.7558789522	-4.0469245787	0.1437734240
C	0.6877075158	-2.0527180322	3.4826136753
H	2.5624790818	-1.1973810853	2.8657016287
C	2.6351559802	-2.9312723528	-3.7465780350
H	2.9984681352	-1.3055056277	-2.3806823381
C	2.6357771652	-5.1603297456	-2.8083841186
H	3.0042459911	-5.3097962990	-0.6969955227
C	5.6119737572	-4.8230127123	3.1145882347
H	3.8894334464	-3.5375488692	3.1749937199
C	6.6034471462	-5.1951075705	0.9407497318
H	5.6642806748	-4.2069279214	-0.7216105680
C	3.7726529032	5.4311771088	4.2175282453
H	5.3891013153	4.1064336711	4.8161731049
H	2.1041333073	6.5155597855	3.4180991682
C	-3.5359485212	5.5891690199	1.5647431751
H	-2.3749139069	6.4342297742	-0.0372727635
C	-2.9707058923	3.3302992154	2.2213197408
H	-1.3884449788	2.3628816215	1.1107314237
C	-1.1192398441	7.5381732466	-3.2776409998
H	-2.0633989425	5.6961363358	-2.6929894381
C	1.0388993323	8.0033202828	-2.2887426853
H	1.8025847469	6.5317368396	-0.9197145727
C	0.3402324235	1.8489979868	-4.0191616822
H	1.3180256483	3.5018384699	-3.0491608408
C	-1.7904870683	1.1563613062	-3.0913671146
H	-2.4636826872	2.2237756118	-1.3412973076
H	9.3074411869	1.8549614922	-0.2567814348
C	-0.3459386722	-2.9420575202	3.1726529213
H	-1.1051201265	-4.3557838763	1.7285527542
H	0.6452630825	-1.4659984594	4.3971127201
C	2.5024061559	-4.3099679419	-3.9132096315
H	2.5178756338	-2.2611583359	-4.5967649471
H	2.5405906080	-6.2353495571	-2.9357952463
C	6.5914051426	-5.4247241549	2.3202552769
H	5.5969368059	-5.0006533289	4.1876617154
H	7.3619017615	-5.6647919625	0.3185258391
H	3.9266391111	6.1212108503	5.0493754860
C	-3.7490302730	4.4805283413	2.3877655923
H	-4.1411206523	6.4842024565	1.6878244959
H	-3.1396120026	2.4651425436	2.8677941587
C	-0.0051655665	8.3730374977	-3.1402041784
H	-1.9300877513	7.8201212778	-3.9459188503
H	1.9097406292	8.6469459444	-2.1843623059
C	-0.7742317447	1.0024013268	-4.0465990527
H	1.1342952248	1.7199098609	-4.7530039958
H	-2.7110198457	0.5625544971	-3.1730376568
H	-1.1826380992	-3.0615562252	3.8575131860
H	2.2996610220	-4.7244963286	-4.8982492788
H	7.3409148004	-6.0705954393	2.7724703416

H	-4.5175876184	4.5112705872	3.1571839196
H	0.0490546971	9.3056764666	-3.6982586279
H	-0.8688498223	0.2396962607	-4.8343517794

(2S,4R)-CYr
0 1

O	-1.0565345481	-3.3012194389	-1.1380848672
C	-2.2327304779	-2.9562588158	-1.1790701662
C	-2.7727536428	-1.6344376405	-0.7508744044
C	-4.0900131328	-2.6504966083	-2.5105370470
C	-4.1114158774	-1.4477442890	-1.5151693843
H	-2.0317052697	-0.8484538469	-0.9692301226
H	-2.8456490223	-1.6584995558	0.3525357952
H	-3.5109934242	-2.3660851842	-3.4060594010
H	-5.0892155408	-2.9458536526	-2.8517726893
C	-4.0868154037	-0.1140597579	-2.2729769989
H	-4.9670643652	0.0078199072	-2.9191344557
H	-4.0686646981	0.7296337904	-1.5649025065
H	-3.1884045340	-0.0326224598	-2.9064228037
C	-5.3801148088	-1.5142729970	-0.6470809736
C	-5.3697871716	-1.6350702929	0.7444102816
C	-6.6270645118	-1.4688962878	-1.2900207338
C	-6.5635861471	-1.7347867469	1.4614517913
H	-4.4363441662	-1.6787408656	1.3069633636
C	-7.8180827044	-1.5637947282	-0.5779463822
H	-6.6774647123	-1.3669415414	-2.3776334785
C	-7.7913384251	-1.7046847024	0.8090245613
H	-6.5191828665	-1.8459998250	2.5465730878
H	-8.7712556030	-1.5300987363	-1.1102063585
C	-3.3420744596	-3.7959159167	-1.7847531799
C	-2.8607427967	-4.8468408301	-2.7851034301
H	-2.1424543982	-4.4134928932	-3.4925498177
H	-3.7200122637	-5.2369351914	-3.3444635737
C	-4.1801539852	-4.3998524872	-0.6517900799
C	-3.6619698342	-4.5415361036	0.6373486638
C	-5.4942647130	-4.8088237872	-0.8995951155
C	-4.4528376236	-5.0490393075	1.6663705873
H	-2.6444611980	-4.2217971355	0.8743519845
C	-6.2813722961	-5.3235134799	0.1241560010
H	-5.9250379979	-4.7041488095	-1.8977292881
C	-5.7640060269	-5.4385541429	1.4145258714
H	-4.0374890743	-5.1201372469	2.6729424062
H	-7.3104601090	-5.6222234295	-0.0838036434
Mg	0.5586202698	-2.5590476036	-0.0824621102
O	0.3067348791	0.6320085834	1.5516027175
O	0.8287489590	-0.7151058251	-0.6307099202
O	1.7213632973	-2.7535538601	1.6813168317
C	0.7261763366	-2.4130700784	2.3587220347
C	0.7365582893	-1.8295141986	3.7456426870
N	-0.4950693464	-2.4971800347	1.8147117601
C	-0.7303002996	-1.4561596902	3.9440570563
H	1.4246899568	-0.9738495156	3.7465283563
H	1.1199847599	-2.5744096556	4.4578687757
C	-1.4268874811	-1.9876694249	2.6904626180
Br	-1.9965320559	-6.4030694268	-2.0040755122
H	-1.1993019405	-1.8751890919	4.8437639253
H	-0.8839245849	-0.3653193481	3.9464966457
O	-2.6247698038	-1.9591211915	2.4983552360
H	-8.7218623369	-1.7886713253	1.3741454979
H	-6.3878228521	-5.8258394657	2.2224324545

P	0.9771370486	0.5404632645	0.2347085771
O	2.5771395519	0.8249274606	0.4764430196
O	0.6097300197	1.7416146196	-0.8254373878
C	3.4213285874	1.1484776935	-0.5522159532
C	0.9658700284	3.0386840231	-0.5323282596
C	3.2986677867	2.4002565669	-1.1277151616
C	4.4118713022	0.2120066892	-0.9613501888
C	2.3091011209	3.3734763749	-0.5891208056
C	-0.0448958497	3.9937652750	-0.2202881493
C	4.1194436689	2.7268158748	-2.2583192537
C	5.2492189561	0.5610953192	-1.9966617895
Si	4.6014808091	-1.5067341576	-0.2048754133
C	2.7217730662	4.6677620512	-0.1287815289
C	0.3561514355	5.2583320994	0.1471087218
Si	-1.9044712455	3.7091279139	-0.4243502497
C	3.9630486297	3.9302339995	-3.0031017478
C	5.1169271061	1.8017405689	-2.6784320445
H	6.0355500056	-0.1214781866	-2.3447528360
C	4.7002940020	-1.5568797741	1.6610836340
C	3.2189065484	-2.5920489055	-0.8736200993
C	6.2155036251	-2.2243361182	-0.8556490363
C	4.0900266957	5.0358384305	0.0055262799
C	1.7293555661	5.6155557124	0.2438058191
H	-0.3776508162	6.0381385672	0.3903721382
C	-2.6329504080	2.5828275300	0.8651130822
C	-2.7293088307	5.3940980311	-0.2296138560
C	-2.3337108639	3.1078763199	-2.1540937210
C	4.7739504827	4.2083373893	-4.0761484839
H	3.1808951278	4.6350927906	-2.7193963457
C	5.9489354855	2.1242424411	-3.7855829804
C	5.2415069732	-2.7118445080	2.2599007133
C	4.2528768241	-0.5178617484	2.4898282850
C	2.4721078345	-2.1898382826	-1.9973709829
C	2.9464820785	-3.8421922800	-0.2872889141
C	7.4281476448	-1.8036812067	-0.2817944061
C	6.2502325172	-3.1520964664	-1.9076768343
C	4.4441668751	6.2833165846	0.4586353735
H	4.8643565957	4.3100415110	-0.2444845778
C	2.1267879913	6.9024444598	0.6980911686
C	-3.9732847859	2.1717714776	0.7293994523
C	-1.9122337361	2.1841719172	1.9998604967
C	-2.6572035305	6.3388052185	-1.2684497111
C	-3.4098274911	5.7357374994	0.9488443479
C	-1.4562083337	2.3510747544	-2.9437046188
C	-3.5974521554	3.4355052851	-2.6811813915
C	5.7882797869	3.3030552469	-4.4692326476
H	4.6308974463	5.1361130809	-4.6345752257
H	6.7177271904	1.4077447810	-4.0875184563
C	5.3240115320	-2.8238370067	3.6507631443
H	5.6130556595	-3.5370164264	1.6464097911
C	4.3347661079	-0.6299235999	3.8842118063
H	3.8287467292	0.4002595214	2.0643037725
C	1.4812604195	-3.0293682020	-2.5321783670
H	2.6432860465	-1.2137860520	-2.4665280337
C	1.9469972666	-4.6785284224	-0.8127542327
H	3.4963837420	-4.1716646361	0.6026483177
C	8.6481384308	-2.3008829816	-0.7516627875
H	7.4326271211	-1.0845990710	0.5427615604
C	7.4711046212	-3.6511364918	-2.3777142742
H	5.3256338215	-3.5002241380	-2.3738852367

C	3.4544953979	7.2348611892	0.8009576891
H	5.5005130816	6.5411280793	0.5607694295
H	1.3509570266	7.6229440815	0.9712409586
C	-4.5750193848	1.3732165984	1.7042172035
H	-4.5631570405	2.4717684236	-0.1399707556
C	-2.5172770794	1.3797321534	2.9751718756
H	-0.8623062177	2.4688213141	2.1366006400
C	-3.2530645240	7.5963011156	-1.1308040810
H	-2.1357904235	6.0999234589	-2.1992534980
C	-4.0070633502	6.9949849131	1.0876442461
H	-3.4861599910	5.0220452789	1.7744291452
C	-1.8235454944	1.9405527993	-4.2319460119
H	-0.4663258254	2.0648725881	-2.5674047843
C	-3.9677268449	3.0232817482	-3.9651526989
H	-4.3086393387	4.0273200553	-2.0973412032
H	6.4306277622	3.5398377812	-5.3198993183
C	4.8683634198	-1.7816501224	4.4657310717
H	5.7430312065	-3.7208796724	4.1000436705
H	3.9831794314	0.1868152998	4.5112791127
C	1.2259986333	-4.2796737986	-1.9476431000
H	0.9209193188	-2.7130344876	-3.4107890370
H	1.7523788586	-5.6490084177	-0.3562964188
C	8.6706293225	-3.2257621450	-1.8006105887
H	9.5804388458	-1.9691129652	-0.2992879555
H	7.4850806802	-4.3718051072	-3.1924601319
H	3.7494672422	8.2248112816	1.1546463254
C	-3.8429919990	0.9704249410	2.8278450601
H	-5.6073565838	1.0499739362	1.5844593393
H	-1.9309866884	1.0489979580	3.8336083223
C	-3.9291816070	7.9258327606	0.0488105747
H	-3.1920784651	8.3185036787	-1.9417826177
H	-4.5332031490	7.2464505043	2.0060058845
C	-3.0779975488	2.2775206819	-4.7453441394
H	-1.1240043281	1.3651295299	-4.8337515790
H	-4.9457661877	3.2887119806	-4.3594414193
H	4.9331578211	-1.8685560115	5.5482139623
H	0.4702995407	-4.9411327195	-2.3768484632
H	9.6197998420	-3.6136373589	-2.1651003534
H	-4.3022174040	0.3240073121	3.5736924972
H	-4.3935858080	8.9038625548	0.1557142062
H	-3.3610918692	1.9660740754	-5.7480889187

(2S,4S)-CYr
0 1

C	-3.6818128673	-1.2695854939	0.4069723569
C	-4.5239924186	-1.9844328776	1.3755205526
C	-5.7719954413	-1.1260294796	1.6271351729
H	-4.6375794502	-3.0573837094	1.1791018887
H	-3.8621463304	-1.9138489937	2.2948425225
C	-4.2310224956	0.1265094102	0.1394366963
C	-3.1302881747	1.1841174744	0.0169613552
H	-2.4846741243	0.9768992665	-0.8461717701
H	-3.5808690514	2.1748532024	-0.1229706028
O	-0.8312552268	-2.5834153907	2.4508685600
C	-1.2588785204	-1.9463031360	3.4673079104
C	-0.3159053452	-1.4353149728	4.5332644698
N	-2.5201486797	-1.6397839486	3.6597554356
C	-1.2549979546	-0.6638474620	5.4515973156
H	0.1997047659	-2.2834312373	5.0075276032
H	0.4420765205	-0.8157476665	4.0298289356

C	-2.6323761286	-0.8654683310	4.8051389452
Br	-1.9295126970	1.3310702140	1.5404339049
H	-1.0374009968	0.4184404762	5.4788398139
H	-1.2832109823	-1.0210599383	6.4902022481
O	-3.6684598290	-0.4009005974	5.2235089236
O	-2.6108101847	-1.6834327733	-0.0522730001
C	-5.1780144004	0.2835939157	1.3484717952
H	-4.5725655191	0.5755707802	2.2216246397
H	-5.9411858998	1.0588096836	1.2129182015
C	-6.2335917578	-1.2600544760	3.0851026598
H	-6.6023197595	-2.2790925059	3.2749429701
H	-7.0512635549	-0.5612640177	3.3115281037
H	-5.4068052547	-1.0457277369	3.7805439206
C	-6.9303170497	-1.4242679948	0.6661707639
C	-6.8437972319	-2.3523376227	-0.3755241818
C	-8.1239134051	-0.6981912009	0.7884731267
C	-7.8932961681	-2.5317464403	-1.2771034864
H	-5.9332133831	-2.9345604877	-0.5219404837
C	-9.1766850326	-0.8749405666	-0.1035386820
H	-8.2286347570	0.0389126023	1.5882647993
C	-9.0643524413	-1.7921273309	-1.1482811001
H	-7.7863004163	-3.2522110194	-2.0906351801
H	-10.0906420439	-0.2887718958	0.0156109267
H	-9.8850774763	-1.9282584860	-1.8553100295
C	-4.9453261671	0.1101315763	-1.2325010452
C	-4.3970749810	-0.5894306851	-2.3141159681
C	-6.1207982270	0.8383260466	-1.4433350289
C	-5.0296948093	-0.5992264374	-3.5559523388
H	-3.4635617172	-1.1396180681	-2.1898421117
C	-6.7540118452	0.8291999645	-2.6821590590
H	-6.5720525476	1.4056108289	-0.6303713629
C	-6.2172696637	0.1018058907	-3.7426208564
H	-4.5884821436	-1.1616331767	-4.3808844780
H	-7.6811428011	1.3899225570	-2.8138233989
H	-6.7206812051	0.0870984001	-4.7109602050
Mg	-0.7485627382	-2.1238187687	0.5963493199
O	0.6898426640	-1.0025573553	0.0293663984
P	1.5661451332	-0.0560765823	0.8573232732
O	1.1731771275	0.3277316425	2.2317248277
O	3.0757486262	-0.6926212130	0.9311438007
O	1.8125115881	1.1928355051	-0.1715985668
C	3.8258007989	-0.8298552239	-0.2072285700
C	2.8367209847	2.0725776706	0.0930187534
C	4.3620490137	0.3117515853	-0.7752759200
C	4.0309852364	-2.1308677172	-0.7418215235
C	4.1330584354	1.6353070319	-0.1286809006
C	2.5334243746	3.3843356459	0.5581934507
C	5.0881742183	0.1899779734	-2.0059115150
C	4.7867335156	-2.2489417710	-1.8865564258
Si	3.2895845022	-3.6798288406	0.0415137001
C	5.2238822421	2.4695694867	0.2816728973
C	3.5910830092	4.2024371350	0.8870589344
Si	0.8142525331	4.1781085654	0.6244890584
C	5.5682326718	1.3169798998	-2.7316230849
C	5.3171059950	-1.1074841808	-2.5487754891
H	4.9871672340	-3.2298860681	-2.3363955165
C	3.6716404074	-3.9194941131	1.8542558389
C	1.4259548698	-3.7397591791	-0.2434941197
C	4.0041083922	-5.1526876645	-0.8859314155
C	6.5804646093	2.0425118510	0.2318219487

C	4.9421319117	3.7660333961	0.7953457698
H	3.4241742329	5.2283948293	1.2411153213
C	-0.0753319343	3.9561529961	2.2553511114
C	1.1161421946	6.0234586242	0.3676947605
C	-0.3375313572	3.6595893582	-0.7685809397
C	6.2648650291	1.1584452168	-3.9044716055
H	5.3721616093	2.3191461056	-2.3489065328
C	6.0518821998	-1.2381739121	-3.7594343590
C	3.6154926342	-5.2298382135	2.3706189107
C	3.9939784866	-2.8676161638	2.7231569637
C	0.8677660457	-3.1463680811	-1.3938907142
C	0.5891098092	-4.4702058754	0.6172257455
C	5.3238614322	-5.5472116674	-0.6011004788
C	3.2818465516	-5.8600358114	-1.8583752030
C	7.5962600609	2.8705194823	0.6435116785
H	6.8105308424	1.0396030836	-0.1291262891
C	6.0160628228	4.6032533185	1.2032298941
C	-1.1270693397	4.8372357079	2.5725948117
C	0.1883508508	2.8852484459	3.1222281986
C	1.2184315103	6.5376183467	-0.9352360680
C	1.2669263300	6.9001866089	1.4545696604
C	-0.0225740659	2.7273149768	-1.7658598171
C	-1.5968931679	4.2901642512	-0.8151520437
C	6.5220349445	-0.1331194833	-4.4230384067
H	6.6202343438	2.0388034797	-4.4446113141
H	6.2294942902	-2.2411157841	-4.1570531431
C	3.8695493616	-5.4758747808	3.7221838641
H	3.3801161777	-6.0768460521	1.7199678343
C	4.2486615894	-3.1143347119	4.0791269682
H	4.0496490136	-1.8325606177	2.3619414516
C	-0.4919885060	-3.3172687944	-1.6987949352
H	1.4862562285	-2.5311882037	-2.0582464660
C	-0.7735013986	-4.6517349122	0.3134486476
H	0.9829607661	-4.9139173303	1.5400394368
C	5.9062101880	-6.6246019688	-1.2761293367
H	5.9111630367	-5.0184524113	0.1559716248
C	3.8641381872	-6.9395105697	-2.5342105874
H	2.2541213601	-5.5822229648	-2.1016536079
C	7.3164374236	4.1706172936	1.1268059919
H	8.6297179699	2.5197561997	0.6023435306
H	5.7846119888	5.6009496338	1.5860648142
C	-1.9154933432	4.6315677027	3.7088400860
H	-1.3465001923	5.6972221963	1.9344678765
C	-0.6179935666	2.6652766956	4.2461340337
H	1.0236956307	2.1942301070	2.9346199818
C	1.4636908907	7.8993585026	-1.1469524328
H	1.1044836374	5.8814469749	-1.8025193030
C	1.5112373989	8.2616451525	1.2440259824
H	1.1921382871	6.5304914182	2.4807962321
C	-0.9417666287	2.4212406834	-2.7790288201
H	0.9452310440	2.2140875290	-1.7708951990
C	-2.5122533506	3.9922992394	-1.8288789562
H	-1.8801912537	5.0262592679	-0.0570172714
H	7.0830931568	-0.2451604676	-5.3530637242
C	4.1857934800	-4.4157143313	4.5798643513
H	3.8233749159	-6.4913055735	4.1089847474
H	4.4926638216	-2.2845446254	4.7403023227
C	-1.3131720580	-4.0900286375	-0.8563247447
H	-0.9073822784	-2.8700498057	-2.6024663891
H	-1.4020400703	-5.2467887301	0.9793530079

C	5.1762570893	-7.3222691804	-2.2441614363
H	6.9275945928	-6.9218879091	-1.0465142383
H	3.2929623214	-7.4814108846	-3.2850382442
H	8.1334395006	4.8207142305	1.4461100034
C	-1.6706780642	3.5343700948	4.5411275643
H	-2.7252935783	5.3186516011	3.9410614082
H	-0.4241493999	1.8017623362	4.8898201643
C	1.6099448735	8.7628257790	-0.0575680781
H	1.5394468090	8.2855011339	-2.1610677581
H	1.6228722870	8.9309958869	2.0941721324
C	-2.1858112765	3.0535098306	-2.8137476850
H	-0.6786371282	1.6915215400	-3.5411299108
H	-3.4757823180	4.4952021495	-1.8564756973
H	4.3823538171	-4.6071935382	5.6331093289
H	-2.3575390430	-4.2646412269	-1.1200795293
H	5.6291883227	-8.1616848832	-2.7684484220
H	-2.3010503815	3.3562759326	5.4106265582
H	1.7992539308	9.8215003894	-0.2216866587
H	-2.8979451325	2.8204960363	-3.6024643081

(2S,5R)-EPr
0 1

O	-0.6950646953	2.7709592945	0.9855101077
C	-0.5794381907	3.9543378577	0.2060079531
C	-1.2088459163	5.2867437645	0.5381270137
C	-0.8880200013	4.1385319485	-1.2648762364
C	-0.9461618383	5.6664318949	-0.9595528924
H	-2.2843063116	5.1658938748	0.7330772264
H	-0.7439977441	5.8874619343	1.3297513783
H	-1.9028553206	3.7818440440	-1.4839657113
H	-0.1900047642	3.7359452466	-2.0087177465
C	0.6227715855	3.3605271911	0.8147772266
C	1.5806446961	2.6078156637	-0.0748077483
H	1.0627377868	1.9818953220	-0.8119216468
H	2.2006316296	3.3424558640	-0.6046213638
Mg	-1.5411046645	0.9024054481	0.6363858286
O	-0.3041534786	-0.1807413082	1.6242343762
O	1.1100471959	-2.1283708731	2.6794208047
O	-3.2724900036	1.5309241845	-0.6148212418
C	-2.5599368499	1.3241718348	-1.6238990683
C	-2.9618473817	1.5244975938	-3.0674804777
N	-1.2967397792	0.9268341346	-1.4774664994
C	-1.6918008501	1.1250398070	-3.8153279925
H	-3.8380976771	0.9026185144	-3.2953739479
H	-3.2604327910	2.5729920658	-3.2175194113
C	-0.6633339124	0.8768932745	-2.7055439235
Br	2.7989484514	1.4563209507	0.8883544414
H	-1.3093075934	1.8877898271	-4.5065232585
H	-1.8105396816	0.1922418469	-4.3849496673
O	0.5206543923	0.7098884172	-2.8741485149
C	1.1923614657	4.0045408549	2.0411244665
C	1.9940073480	5.1407164592	1.8963659409
C	0.9666115843	3.4684440651	3.3078221996
C	2.5471848798	5.7492516146	3.0201536631
H	2.1845664270	5.5540829930	0.9026554819
C	1.5227311496	4.0792580219	4.4301726765
H	0.3740821306	2.5558948978	3.4043573748
C	2.3089856003	5.2217826911	4.2890411597
H	3.1686110026	6.6389460126	2.9006606365
H	1.3509754897	3.6519274022	5.4192890849

H	2.7451552620	5.6976144483	5.1695292818
C	-2.0503438546	6.4381548339	-1.6757829721
H	-1.8330225916	6.5085945696	-2.7523524052
H	-2.1363782380	7.4620959906	-1.2833189331
H	-3.0228492859	5.9385018817	-1.5489691230
C	0.3964986958	6.3768638062	-1.0960233446
C	0.6090077616	7.5637997570	-0.3803269685
C	1.4365541036	5.8998039788	-1.8987493185
C	1.8252274783	8.2361180362	-0.4420727886
H	-0.1872164275	7.9602400251	0.2549953122
C	2.6621779153	6.5668136241	-1.9591953774
H	1.3087721095	4.9887953093	-2.4855205601
C	2.8638579806	7.7330647422	-1.2268282888
H	1.9675470581	9.1529233976	0.1337170260
H	3.4636258187	6.1643850228	-2.5823359680
H	3.8243443085	8.2502920961	-1.2682491655
P	0.3582665886	-1.5579164747	1.5510236298
O	1.1534493120	-1.5236800015	0.1073167968
O	-0.8671330763	-2.6101232941	1.1619991637
C	1.4488981686	-2.7025882212	-0.5263111559
C	-1.5537649082	-2.4032512880	0.0038935532
C	0.4092461428	-3.3832998275	-1.1432111208
C	2.7930222818	-3.1793425130	-0.5540353387
C	-0.9438378547	-2.7656925237	-1.1861703496
C	-2.8586069173	-1.8291738436	0.0517646808
C	0.6512181766	-4.6829842433	-1.6963420073
C	3.0267434184	-4.3987939550	-1.1482051706
Si	4.3170045114	-2.1557694733	-0.0887790139
C	-1.6320693379	-2.5255927270	-2.4168118770
C	-3.5559585289	-1.6988527381	-1.1322454223
Si	-3.7016325557	-1.2796857341	1.6533670069
C	-0.3856107051	-5.5117470051	-2.2107746161
C	1.9795232553	-5.1905161349	-1.6954767273
H	4.0412862849	-4.8122550331	-1.2146438561
C	4.5387359035	-0.8552566034	-1.4207929046
C	4.3215326127	-1.3080860494	1.5767102766
C	5.7957908099	-3.3268140175	-0.1022893762
C	-1.0312128096	-2.7461015715	-3.6894432858
C	-2.9711155077	-2.0399692684	-2.3795347190
H	-4.5758318373	-1.2964052893	-1.1519006927
C	-5.5147999370	-0.9981490165	1.2171674873
C	-3.6394639788	-2.5802030062	2.9993602473
C	-3.0155755020	0.3036775849	2.4285685391
C	-0.1076215707	-6.7552752729	-2.7243143960
H	-1.4165236571	-5.1575091705	-2.1844764337
C	2.2370600335	-6.4767452511	-2.2423520382
C	5.8169977039	-0.4691268333	-1.8609531494
C	3.4139176915	-0.2128594149	-1.9625944169
C	3.7150097571	-1.8656977182	2.7102200027
C	5.0094206994	-0.0840750570	1.7043094897
C	6.4177725432	-3.6891943560	-1.3100930986
C	6.2918259951	-3.8602822766	1.0973762160
C	-1.7465236728	-2.5680825386	-4.8474739363
H	0.0124961893	-3.0574257241	-3.7349181627
C	-3.6935832237	-1.8871852839	-3.5968660930
C	-5.9520915992	0.1442217069	0.5253276094
C	-6.4617503747	-1.9812780814	1.5495673468
C	-4.3908529837	-2.3352226892	4.1671561040
C	-2.9020604585	-3.7679302275	2.9095637329
C	-3.5079202867	1.6005597635	2.1521871922

C	-2.1090320328	0.1585662887	3.5080080923
C	1.2195791956	-7.2442995727	-2.7521490617
H	-0.9207122678	-7.3750674682	-3.1084705254
H	3.2655903015	-6.8475809102	-2.2424468587
C	5.9671910401	0.5204486803	-2.8373877833
H	6.7128398403	-0.9350609488	-1.4440978792
C	3.5652170033	0.7743246994	-2.9453887269
H	2.3951197931	-0.4591736365	-1.6269717664
C	3.7469384978	-1.1887173724	3.9383020921
H	3.1820968785	-2.8238029973	2.6564280115
C	5.0330589407	0.5927868593	2.9281240294
H	5.5452639517	0.3475327043	0.8522741764
C	7.5105941618	-4.5616233275	-1.3171845708
H	6.0547929978	-3.2913808043	-2.2617419654
C	7.3850932394	-4.7357895760	1.0914985869
H	5.8344340679	-3.5977332905	2.0555512322
C	-3.1017384246	-2.1541407890	-4.8054037124
H	-1.2650509956	-2.7438048353	-5.8116009603
H	-4.7326363483	-1.5491553966	-3.5492361737
C	-7.2981976430	0.3089068711	0.1871109494
H	-5.2349883171	0.9283277753	0.2382984195
C	-7.8110160845	-1.8197485434	1.2107305627
H	-6.1623540099	-2.8917622899	2.0781827143
C	-4.4056518917	-3.2609870245	5.2124768681
H	-4.9731813762	-1.4152644342	4.2712173327
C	-2.9152759261	-4.6965587424	3.9601694565
H	-2.2889680299	-3.9863039131	2.0267049808
C	-3.1205767147	2.7018437617	2.9272527542
H	-4.2493985273	1.7598419851	1.3487270626
C	-1.7362191272	1.2581552913	4.2864008032
H	-1.7023224269	-0.8275429805	3.7706005291
H	1.4280940935	-8.2324015176	-3.1670470458
C	4.8384863337	1.1400037116	-3.3854440826
H	6.9605287973	0.8077314507	-3.1710559180
H	2.6742888650	1.2439985393	-3.3638305858
C	4.3958249427	0.0417638950	4.0466389152
H	3.2363347366	-1.6224338841	4.7965564433
H	5.5479911408	1.5468469501	3.0110539557
C	7.9958714705	-5.0867141196	-0.1148698344
H	7.9846619534	-4.8310879796	-2.2580751106
H	7.7588230448	-5.1409791609	2.0292784688
H	-3.6639942250	-2.0352768697	-5.7336433188
C	-8.2318960883	-0.6740516047	0.5314464563
H	-7.6201986330	1.2020315594	-0.3433092218
H	-8.5317309493	-2.5900000616	1.4781859760
C	-3.6659402406	-4.4456903922	5.1092788894
H	-4.9885349978	-3.0620348517	6.1085926437
H	-2.3285410331	-5.6098714851	3.8777123244
C	-2.2429369835	2.5299951305	3.9995755579
H	-3.5153787170	3.6891916524	2.6979353316
H	-1.0433700760	1.1183345648	5.1161524262
H	4.9549309092	1.9028907442	-4.1508138641
H	4.4058128141	0.5740372385	4.9945557056
H	8.8461782089	-5.7650091577	-0.1198181198
H	-9.2805474122	-0.5469921899	0.2706616625
H	-3.6743632582	-5.1654332462	5.9255231222
H	-1.9512470626	3.3844015136	4.6101255978

(2S,5S)-EPr
0 1

O	-1.8125134809	1.6107974218	0.4427158059
C	-2.8747020360	2.3977099147	-0.0870006982
C	-3.2097275356	2.6005154346	-1.5464841624
C	-4.3469956523	2.3691038076	0.2650701813
C	-4.5519986745	3.2041232917	-1.0415975642
H	-3.3778150358	1.6258917363	-2.0312657761
H	-2.5360660755	3.1935854424	-2.1710292519
H	-4.7532133565	1.3544164215	0.1304030693
H	-4.6785338657	2.7865454456	1.2243212363
C	-1.7795128521	3.0520676864	0.6590409283
C	-1.9821639866	3.3605805201	2.1211555456
H	-2.8790305122	2.8636831225	2.5071387399
H	-2.0500327859	4.4333806303	2.3241517533
Mg	-0.3488068125	0.2621981689	1.3229248007
O	0.8399213795	0.6571784719	-0.2332282244
O	-0.4511350857	0.0195622727	-2.4580172330
O	0.6292038280	-0.7710228846	2.6641070767
C	1.4493790097	-0.4091995870	3.5674519361
C	2.3027759833	-1.4234226699	4.3008503734
N	1.6189615137	0.8308470993	3.9562996539
C	3.1484068507	-0.5391467389	5.2058980909
H	2.8736918076	-2.0163982148	3.5746451009
H	1.6486932351	-2.1268343900	4.8375426361
C	2.5870601795	0.8723092833	4.9564143114
Br	-0.4635177591	2.6105290407	3.1037006992
H	3.0807696742	-0.7768850674	6.2766428873
H	4.2165956428	-0.5430288567	4.9408502065
O	2.9395962745	1.8652180948	5.5418826822
C	-0.7411026758	3.8358305383	-0.0926693955
C	-0.3911891650	5.1252249671	0.3240414322
C	-0.1794930222	3.3313814991	-1.2706904651
C	0.4938801430	5.8975799954	-0.4237997054
H	-0.8097632381	5.5549810143	1.2338422254
C	0.7051158222	4.1052641033	-2.0157984623
H	-0.4173425220	2.3243995778	-1.6119164194
C	1.0440305592	5.3907819332	-1.5987069864
H	0.7562404539	6.8995244203	-0.0795675587
H	1.1379048475	3.6838400937	-2.9262501019
H	1.7431634030	5.9929407138	-2.1821376546
C	-5.7878727894	2.8877545598	-1.8746216139
H	-6.7082102496	3.1978895649	-1.3590320714
H	-5.7440917680	3.4170749346	-2.8373865362
H	-5.8506479585	1.8063172812	-2.0781302493
C	-4.4650474507	4.6972488224	-0.7243130583
C	-3.5038544659	5.5453141055	-1.2816303841
C	-5.3943050029	5.2534163805	0.1678323648
C	-3.4633240397	6.9007857881	-0.9500756412
H	-2.7543567239	5.1579152642	-1.9725812337
C	-5.3611441512	6.6047904541	0.4976683354
H	-6.1600608221	4.6145638125	0.6168836007
C	-4.3888406172	7.4362414501	-0.0598845704
H	-2.6915340160	7.5353321035	-1.3905583357
H	-6.0957888444	7.0111815354	1.1960245159
H	-4.3547493593	8.4958791897	0.2009059890
P	0.3991974877	-0.3303998393	-1.3036644983
O	1.6120226692	-1.3006431599	-1.7519347446
O	-0.4301440802	-1.3831381903	-0.2129656465
C	2.4211749604	-1.8040410356	-0.7532127954
C	-0.5216848616	-2.7471790252	-0.4208058263
C	1.9494024775	-2.8950487816	-0.0548493071

C	3.6654573607	-1.1794333759	-0.4847223808
C	0.6414498548	-3.5011811582	-0.4297326747
C	-1.8085024987	-3.3355378693	-0.5823555996
C	2.7313628499	-3.4018219413	1.0355680107
C	4.4445985809	-1.7145595766	0.5131179368
Si	4.3210171445	0.3732335475	-1.3470476123
C	0.5627459928	-4.8815255017	-0.8153187715
C	-1.8805237612	-4.6798321363	-0.8695097265
Si	-3.4386644149	-2.4555387214	-0.2327911994
C	2.2796316049	-4.4425383440	1.8936063505
C	4.0081601308	-2.8246505916	1.2899131865
H	5.4238120471	-1.2828923893	0.7544813281
C	3.4651786028	0.8251554584	-2.9486542770
C	4.2591690404	1.7974712784	-0.1395628516
C	6.1142432214	-0.0186475503	-1.7667341296
C	1.7206052973	-5.6845177771	-1.0174840172
C	-0.7125542716	-5.4715520060	-1.0363695697
H	-2.8485228403	-5.1843254703	-0.9842959944
C	-4.1088126872	-1.5026353772	-1.6926968626
C	-4.6918045897	-3.7802159437	0.2331276342
C	-3.2820752888	-1.3389465183	1.2789331802
C	3.0731718745	-4.9138017583	2.9115584195
H	1.2867414230	-4.8657003732	1.7410573186
C	4.8114966864	-3.3440431359	2.3417445849
C	3.4051425889	2.1760324229	-3.3314463452
C	2.9596266250	-0.1487355735	-3.8259598429
C	3.3061784348	1.8355076682	0.8897453888
C	5.1746118418	2.8606872673	-0.2576805251
C	6.4403375376	-0.5989124285	-3.0032887478
C	7.1458501001	0.2304035096	-0.8456036362
C	1.6096604315	-7.0015434043	-1.3904107080
H	2.7074057607	-5.2397461925	-0.8848616376
C	-0.7957479577	-6.8408561161	-1.4096410381
C	-5.4948695684	-1.2718951531	-1.7963884355
C	-3.2703059437	-1.0390388745	-2.7158731152
C	-4.8389270887	-4.1791024957	1.5713789276
C	-5.4717357853	-4.4119516189	-0.7507009969
C	-2.1423580815	-1.3204658159	2.1108413892
C	-4.3860114431	-0.5543476213	1.6358783250
C	4.3610305946	-4.3720127891	3.1324969899
H	2.7037850649	-5.7112058894	3.5595416430
H	5.7940797945	-2.8985756531	2.5172760006
C	2.8617468408	2.5433609515	-4.5682446394
H	3.7785141175	2.9610189372	-2.6672340335
C	2.4010448004	0.2192363781	-5.0548831184
H	2.9764219014	-1.2091579332	-3.5520273508
C	3.2839725710	2.9009822331	1.7997132140
H	2.5544857835	1.0410009770	0.9961619365
C	5.1471599252	3.9264837047	0.6450640615
H	5.9215979801	2.8652307224	-1.0549246627
C	7.7665541991	-0.9248028094	-3.3116112642
H	5.6642388214	-0.8010019600	-3.7461489221
C	8.4715465070	-0.0938473745	-1.1528770057
H	6.9283328461	0.6886041567	0.1228882249
C	0.3384947036	-7.5938855504	-1.5801347567
H	2.5121674591	-7.5961119110	-1.5470810195
H	-1.7839163223	-7.2817738305	-1.5648663673
C	-6.0269901334	-0.6065055028	-2.9031281498
H	-6.1777932380	-1.6232559533	-1.0187118921
C	-3.8035635035	-0.3605665075	-3.8216301100

H	-2.1802988687	-1.1855233010	-2.6788137890
C	-5.7496913497	-5.1835962497	1.9199790590
H	-4.2453964096	-3.7116449536	2.3615357028
C	-6.3820222338	-5.4154460317	-0.4032222939
H	-5.3792469355	-4.1260622931	-1.8023464856
C	-2.0934678998	-0.4708563226	3.2262318804
H	-1.3210036369	-2.0483025366	1.9549342916
C	-4.3431841326	0.2827147316	2.7629234927
H	-5.3075630671	-0.5881721384	1.0464483196
H	4.9843907066	-4.7598159243	3.9402855926
C	2.3559716929	1.5653191113	-5.4293078625
H	2.8348138815	3.5901750478	-4.8602727537
H	1.9924214524	-0.5437848582	-5.7138003026
C	4.2037540655	3.9437137018	1.6797975827
H	2.5480460775	2.9058078744	2.6079470422
H	5.8599451378	4.7410179450	0.5477492561
C	8.7835158217	-0.6730726404	-2.3867863663
H	8.0048072551	-1.3730775278	-4.2736064923
H	9.2607792430	0.1070014591	-0.4319348090
H	0.2649297045	-8.6436797907	-1.8707022688
C	-5.1788986037	-0.1493734720	-3.9201272394
H	-7.1004478544	-0.4510972318	-2.9816499558
H	-3.1310469418	-0.0038334081	-4.6019380615
C	-6.5224233784	-5.8020924122	0.9334461206
H	-5.8548599261	-5.4831305159	2.9608454879
H	-6.9819303800	-5.8955735811	-1.1737479718
C	-3.1924140469	0.3420122531	3.5445053779
H	-1.2292129701	-0.4916072614	3.9036586230
H	-5.2170507779	0.8749644004	3.0291314667
H	1.9247904147	1.8506341207	-6.3864609551
H	4.1884923559	4.7672647759	2.3910939235
H	9.8142628145	-0.9252196754	-2.6262210926
H	-5.5936874693	0.3652956176	-4.7848496585
H	-7.2310120375	-6.5827549637	1.2040787272
H	-3.1440807603	0.9941246738	4.4165107114

ECr
0 1

O	-2.1867530339	1.3223010795	0.9396434406
C	-2.6656458197	2.4715672201	0.3933827916
C	-2.6012878950	2.6120500501	-1.1560934505
C	-4.2123130701	2.6079422720	0.2441995632
C	-4.0133342330	3.2529826883	-1.1504431019
H	-2.6276081011	1.6020422481	-1.5923964829
H	-1.7738421271	3.1648917248	-1.6166321665
H	-4.6290068604	1.5972898270	0.1267256675
H	-4.7815033286	3.1431109719	1.0162995798
C	-2.0867896104	3.6921170290	1.1576196449
C	-2.5269965076	3.9769543080	2.3988470315
H	-3.3463435214	3.4094374457	2.8475065116
H	-2.1200472908	4.8161068985	2.9710854686
Mg	-0.8709123384	0.0310020889	1.0457971093
O	0.5045649284	0.4845004197	-0.4415076564
O	-0.5406106811	-0.5486608114	-2.6658502296
O	0.6021695709	-0.3756037352	2.4674575977
C	1.4329031079	0.1692794212	3.1839508634
C	2.7862923382	-0.3702395873	3.5477349047
N	1.2706009089	1.3872536726	3.7698968932
C	3.3802108688	0.6609105899	4.5076548797
H	3.3460394173	-0.4543965370	2.6020796682

H	2.6944753381	-1.3902477380	3.9443511736
C	2.3760144205	1.7986019147	4.5562120474
Br	-0.1948408965	2.4587481811	3.3606616869
H	3.5266227333	0.2805522131	5.5277949431
H	4.3428229792	1.0703544091	4.1673247293
O	2.4452584584	2.8413415714	5.1295726052
C	-0.9990437598	4.5054691458	0.5566117025
C	-1.0548668827	5.9041490241	0.6022278197
C	0.0997282070	3.8927667887	-0.0643205896
C	-0.0326997492	6.6754844034	0.0542172797
H	-1.9347853039	6.3883154090	1.0320030976
C	1.1243648150	4.6666348375	-0.6026187637
H	0.1518135669	2.8029430337	-0.1396934820
C	1.0642599886	6.0591856719	-0.5451974482
H	-0.1027989059	7.7650626734	0.0843420328
H	1.9779312209	4.1670035121	-1.0774028875
H	1.8669222319	6.6622628337	-0.9759477343
C	-4.9733495621	2.7846072082	-2.2433120798
H	-5.9925826513	3.1578612536	-2.0575992623
H	-4.6547944158	3.1500982103	-3.2313742075
H	-5.0064996445	1.6829514837	-2.2828621924
C	-3.9593792234	4.7784372297	-1.1483600985
C	-3.1848218476	5.4459875240	-2.1067681466
C	-4.6962121062	5.5568566722	-0.2500345123
C	-3.1328933598	6.8357942495	-2.1570197469
H	-2.5964275628	4.8640687209	-2.8201200755
C	-4.6476216158	6.9506506423	-0.2929376948
H	-5.3149287805	5.0715140847	0.5067237854
C	-3.8619840423	7.5973753533	-1.2443314044
H	-2.5089898192	7.3272069954	-2.9068202431
H	-5.2280624317	7.5337374924	0.4261090203
H	-3.8179683316	8.6883338334	-1.2752017392
P	0.2633787630	-0.6382110370	-1.4373299124
O	1.5979560837	-1.5274635699	-1.7209516104
O	-0.4841267266	-1.6918980404	-0.2723799386
C	2.3604292910	-1.9821690089	-0.6788743915
C	-0.5335645274	-3.0556261062	-0.4717512295
C	1.9114290050	-3.0925379585	0.0113550260
C	3.5773953358	-1.3203681277	-0.3666132811
C	0.6530640878	-3.7673712674	-0.4130858841
C	-1.7935803703	-3.6812112135	-0.6817454994
C	2.6562977128	-3.5383216241	1.1535108134
C	4.3406499065	-1.8156747990	0.6643733474
Si	4.1771744777	0.2543996682	-1.2282228062
C	0.6358852318	-5.1573348610	-0.7671138576
C	-1.8118793951	-5.0308657888	-0.9459189335
Si	-3.4287754535	-2.7965736035	-0.3483976736
C	2.1869530255	-4.5603198748	2.0263671086
C	3.8997475957	-2.9129898740	1.4567278110
H	5.2959636570	-1.3448748976	0.9304134732
C	3.0767898426	0.8183297735	-2.6191752633
C	4.2899644680	1.5267400711	0.1462872044
C	5.8829750841	-0.0681864666	-1.9403622756
C	1.8240361841	-5.9313860481	-0.8859388422
C	-0.6111785541	-5.7880649420	-1.0394462839
H	-2.7591838507	-5.5643610972	-1.0904732367
C	-4.1267894905	-1.8665288006	-1.8033383578
C	-4.6633791462	-4.1276578537	0.1459074623
C	-3.2075822917	-1.6848680317	1.1578208664
C	2.9222929320	-4.9510047496	3.1191745007

H	1.2244100149	-5.0310261511	1.8247526499
C	4.6482439552	-3.3550653207	2.5815945441
C	2.4157459172	2.0547990613	-2.6471618727
C	2.8877025903	-0.0779494650	-3.6900965666
C	3.1035374065	2.0727214580	0.6648366453
C	5.5204097869	1.9517740835	0.6718118225
C	6.5890920023	1.0233977577	-2.4779942077
C	6.4627653944	-1.3440104103	-1.9984791932
C	1.7695140039	-7.2596956587	-1.2312112441
H	2.7897405985	-5.4554259499	-0.7122537471
C	-0.6349387108	-7.1668676090	-1.3840463388
C	-5.4971129519	-1.5352257508	-1.8092515815
C	-3.3342620424	-1.4820274588	-2.8932822923
C	-4.7951866733	-4.5244695567	1.4861808048
C	-5.4507492675	-4.7636271380	-0.8290200004
C	-2.1156590983	-1.8263840716	2.0438782161
C	-4.1994006435	-0.7394058647	1.4585040587
C	4.1736432081	-4.3513511308	3.3984434312
H	2.5380407272	-5.7321260108	3.7780497044
H	5.6071910008	-2.8742842641	2.7932994024
C	1.5608328119	2.3822976322	-3.7058818206
H	2.5436162683	2.7840775198	-1.8373509696
C	2.0369727348	0.2509961774	-4.7492942829
H	3.3821095282	-1.0542714413	-3.6946494011
C	3.1532529279	3.0587882057	1.6556138717
H	2.1194205283	1.7387515989	0.2928252870
C	5.5672214456	2.9145259806	1.6868607229
H	6.4594905052	1.5425802409	0.2912800429
C	7.8533639535	0.8419552199	-3.0476027808
H	6.1559579975	2.0280396751	-2.4632503347
C	7.7284439019	-1.5262603413	-2.5694148699
H	5.9339161368	-2.2151892187	-1.6057169091
C	0.5272242189	-7.8911415056	-1.4752937975
H	2.6944916327	-7.8323285889	-1.3243210765
H	-1.6003503928	-7.6396514041	-1.5813201729
C	-6.0558048398	-0.8337330294	-2.8793264942
H	-6.1450085276	-1.8299095385	-0.9798967201
C	-3.8954682727	-0.7728192941	-3.9655386737
H	-2.2594853615	-1.7129019851	-2.9323070414
C	-5.6951649179	-5.5348834556	1.8452849071
H	-4.1994801056	-4.0484424225	2.2689637559
C	-6.3499123419	-5.7737698947	-0.4711491644
H	-5.3751215867	-4.4729196353	-1.8808772062
C	-2.0004880399	-0.9897014448	3.1695845427
H	-1.3790829762	-2.6297285035	1.9060474276
C	-4.0988524690	0.0709892701	2.5992745815
H	-5.0612420671	-0.6062978218	0.7977692941
H	4.7543076060	-4.6798853274	4.2624580775
C	1.3658373813	1.4784016023	-4.7533441770
H	1.0353853608	3.3351008241	-3.7031317697
H	1.8774069947	-0.4583084698	-5.5585840905
C	4.3831768423	3.4770282298	2.1719814953
H	2.2295643573	3.5227062970	2.0061609915
H	6.5263781022	3.2381684963	2.0847965442
C	8.4252789969	-0.4338159611	-3.0921690262
H	8.3903954550	1.6929684907	-3.4612694927
H	8.1670512907	-2.5209203457	-2.6096083044
H	0.4977767822	-8.9485079347	-1.7446053653
C	-5.2522386038	-0.4496418050	-3.9609707824
H	-7.1140286673	-0.5846035819	-2.8753531628

H	-3.2574482136	-0.4689717425	-4.7946241927
C	-6.4729996463	-6.1605177900	0.8670668545
H	-5.7898267178	-5.8321323287	2.8874270543
H	-6.9560305566	-6.2569539206	-1.2346742578
C	-2.9936239054	-0.0387256493	3.4440218423
H	-1.1642431511	-1.1167940852	3.8589088242
H	-4.8713053738	0.8102829336	2.8060401229
H	0.6830882196	1.7224689426	-5.5652822969
H	4.4163107813	4.2463282922	2.9425186968
H	9.4083259086	-0.5750537894	-3.5370152201
H	-5.6873316751	0.1005555136	-4.7929763911
H	-7.1735087814	-6.9452582394	1.1457959995
H	-2.8954882447	0.6153126495	4.3088901948

TS2a1
0 1

O	-1.8678890600	3.2172095359	0.4699399625
C	-1.2201518751	4.4031346661	0.2847676746
C	-1.2663473256	5.5693601575	1.3068992138
C	-1.7491071778	5.4090466289	-0.7579765600
C	-1.1787966838	6.5586371394	0.1184739165
H	-2.2715840645	5.5965945603	1.7504031158
H	-0.5228474681	5.6236478220	2.1116216700
H	-2.8461442090	5.3816936423	-0.7046208052
H	-1.4493169639	5.3257411023	-1.8113594058
C	0.1146488073	3.7508255806	0.0465067023
C	0.5876554875	3.6058881095	-1.3565592133
H	0.4155420979	4.5275011592	-1.9206840155
H	1.6445840799	3.3350395591	-1.4206476874
Mg	-2.0979322268	1.3154470156	0.1093027510
O	-0.6285192972	0.3749310618	0.8898728263
O	1.0353381601	-0.7587944374	2.5683564106
O	-3.4791157815	0.8161270585	-1.2112958558
C	-3.3720590671	0.4450784059	-2.4224318389
C	-4.5995834056	0.0955676463	-3.2482367521
N	-2.2396388841	0.3315678507	-3.0746965105
C	-4.0053615861	-0.1618853332	-4.6255304599
H	-5.0888146484	-0.7850545438	-2.8032790328
H	-5.3236544057	0.9213620190	-3.2030606148
C	-2.4887123050	-0.0579749696	-4.3871868161
Br	-0.4078179767	2.1708101032	-2.2608181769
H	-4.2898317793	0.5960358368	-5.3703731798
H	-4.2413652049	-1.1448752762	-5.0524848389
O	-1.6486511177	-0.2584289341	-5.2274370897
C	0.8613925929	3.1766924550	1.1331606271
C	2.1596510523	2.6489036508	0.9348562547
C	0.3032235824	3.0813603391	2.4343246738
C	2.8563523390	2.0665875942	1.9792401659
H	2.6454738702	2.6904172910	-0.0404044542
C	0.9955379813	2.4779178466	3.4646914257
H	-0.7201038690	3.4112425147	2.5968761686
C	2.2720663244	1.9581162420	3.2379044629
H	3.8545711742	1.6591118822	1.8021410427
H	0.5293022326	2.3698889342	4.4430629308
H	2.8067069926	1.4441514808	4.0388732052
C	-2.0077434153	7.8337811480	0.2352860667
H	-2.0264866303	8.3886897903	-0.7146465794
H	-1.5874411938	8.5000294854	1.0039645827
H	-3.0462403858	7.5973650863	0.5116014897
C	0.2526918242	6.8689791194	-0.3162756256

C	1.3697280014	6.6669904453	0.5002283955
C	0.4718666166	7.3439477067	-1.6191349591
C	2.6621498571	6.9091008280	0.0277069365
H	1.2483507409	6.3024544219	1.5209755290
C	1.7559561302	7.5913246070	-2.0930469073
H	-0.3820101373	7.5081107559	-2.2820025126
C	2.8617122270	7.3675727993	-1.2701594384
H	3.5163584521	6.7308807906	0.6847109638
H	1.8968526050	7.9542136707	-3.1132984004
H	3.8716168113	7.5514167814	-1.6418432929
P	0.2721626837	-0.7830289528	1.3033618559
O	1.2101973700	-1.0332624526	-0.0305134944
O	-0.6610662314	-2.1415117900	1.2797698810
C	1.7331805313	-2.2761904509	-0.2882184853
C	-1.3166205819	-2.4519582469	0.1141069355
C	0.8683248162	-3.2724884355	-0.7129554026
C	3.1349537623	-2.5028859753	-0.1501059588
C	-0.5705570066	-2.9655296697	-0.9310613447
C	-2.7234146762	-2.2603979743	0.0270435707
C	1.3644456950	-4.6066047530	-0.8907740387
C	3.6135305887	-3.7728653756	-0.3831081126
Si	4.4483595229	-1.1944568488	0.2017357121
C	-1.2106739153	-3.2008160374	-2.1897860664
C	-3.3522014524	-2.6110406146	-1.1478908832
Si	-3.7827789501	-1.6163990994	1.4462496005
C	0.5184782924	-5.7125271563	-1.1888721973
C	2.7556954346	-4.8518698099	-0.7278113574
H	4.6855078445	-3.9995329488	-0.3020362217
C	4.1895507677	0.3698233230	-0.8065597587
C	4.6611274780	-0.8240799395	2.0238881110
C	6.0896482958	-1.8928117087	-0.4106423453
C	-0.4901255295	-3.5507059408	-3.3648443252
C	-2.6234443030	-3.0595746561	-2.2800075903
H	-4.4409059551	-2.5267509714	-1.2575583136
C	-5.5714080927	-1.7184248522	0.8532185084
C	-3.6080733461	-2.6781269497	2.9827331913
C	-3.4191631141	0.1515714319	2.0009652357
C	1.0352676732	-6.9751995542	-1.3474642253
H	-0.5556868736	-5.5534325841	-1.2836123523
C	3.2626076999	-6.1676474180	-0.9083654677
C	5.1306679605	1.4109937263	-0.6990026328
C	3.1701357763	0.4814672165	-1.7635415114
C	3.7695275464	-1.2983099843	2.9952251047
C	5.7749915733	-0.0670793687	2.4393821111
C	6.4190967733	-1.8023862286	-1.7734765676
C	6.9890840189	-2.5267701935	0.4617045034
C	-1.1432198993	-3.7761750856	-4.5505177118
H	0.5973856052	-3.6179579852	-3.3199933066
C	-3.2745530092	-3.3397901103	-3.5124727607
C	-6.0932580774	-0.7766300654	-0.0500228396
C	-6.3907257458	-2.7861280882	1.2541102711
C	-4.5566926666	-2.5302375924	4.0130094204
C	-2.5599135320	-3.5927506055	3.1568102824
C	-4.0833879257	1.2883019317	1.4881426670
C	-2.5893129183	0.3156210009	3.1312310667
C	2.4246742443	-7.2095262158	-1.2170490463
H	0.3651627887	-7.8079590787	-1.5722272687
H	4.3369379434	-6.3345302881	-0.7907330228
C	5.0600607443	2.5288675376	-1.5355602211
H	5.9401995292	1.3548220204	0.0339313259

C	3.1152958498	1.5886717286	-2.6212078368
H	2.3917311524	-0.2874749240	-1.8533157711
C	3.9785269468	-1.0135537992	4.3534867449
H	2.8797409313	-1.8805803846	2.7183057164
C	5.9832218415	0.2159131042	3.7908509385
H	6.4982657058	0.3031432041	1.7074292799
C	7.6228774965	-2.3315056339	-2.2523864181
H	5.7399055042	-1.3152829772	-2.4791236316
C	8.1933761761	-3.0556922604	-0.0161518848
H	6.7614804454	-2.6142122953	1.5274612471
C	-2.5534566344	-3.6885799600	-4.6256525013
H	-0.5695891431	-4.0156981373	-5.4477610213
H	-4.3639386162	-3.2514311988	-3.5600972578
C	-7.4016057305	-0.8875075525	-0.5286284550
H	-5.4704501491	0.0665492426	-0.3906658926
C	-7.7022077123	-2.9002787391	0.7760884662
H	-6.0177894125	-3.5493353420	1.9435853052
C	-4.4657753146	-3.2925180433	5.1806026184
H	-5.3772370439	-1.8142857275	3.9146145433
C	-2.4684571290	-4.3573615033	4.3277971568
H	-1.7861572589	-3.7139734276	2.3890856754
C	-3.9013000408	2.5510973718	2.0802596322
H	-4.7928296956	1.1971986482	0.6428514191
C	-2.4552850204	1.5621447651	3.7469324265
H	-2.0494130879	-0.5398247737	3.5576630842
H	2.8229457075	-8.2172244565	-1.3521196704
C	4.0575356911	2.6127884541	-2.5083873076
H	5.7959312461	3.3240269450	-1.4440900480
H	2.3385247088	1.6356855575	-3.3859889182
C	5.0814926508	-0.2587186722	4.7520373670
H	3.2672334700	-1.3843284165	5.0899618900
H	6.8480707646	0.7975277013	4.0994154997
C	8.5115392408	-2.9584360875	-1.3739733106
H	7.8665006417	-2.2544923904	-3.3098853719
H	8.8824433647	-3.5426305647	0.6702754634
H	-3.0593893497	-3.8793869597	-5.5740734317
C	-8.2103131178	-1.9508183549	-0.1138063609
H	-7.7925805076	-0.1461394412	-1.2216170182
H	-8.3252369254	-3.7319237077	1.0990111001
C	-3.4210023766	-4.2106846326	5.3378252579
H	-5.2051033070	-3.1715737368	5.9686300476
H	-1.6478686494	-5.0620222589	4.4481270376
C	-3.1135416638	2.6819736969	3.2256560615
H	-4.3873728737	3.4241669579	1.6460421741
H	-1.8344267266	1.6584181776	4.6362186665
H	4.0215921276	3.4662913630	-3.1819534648
H	5.2463375037	-0.0427051962	5.8053592147
H	9.4476945101	-3.3694125206	-1.7460551523
H	-9.2298951879	-2.0382301082	-0.4834228232
H	-3.3504796043	-4.8049624771	6.2464941736
H	-3.0089054511	3.6517987783	3.7068396741

TS2a2

0 1

O	-3.3349282661	-1.3765051542	-0.9012585557
C	-4.1355904664	-1.9118116307	0.0690505073
C	-5.2143627800	-1.1114617961	0.8509257607
C	-5.2764650682	-2.8645317149	-0.3411536630
C	-6.0621990043	-2.4039121065	0.9148954431
H	-5.6722746071	-0.3827523690	0.1644858359

H	-4.9568575542	-0.5958412413	1.7827776381
H	-5.7255376289	-2.4491920254	-1.2540876317
H	-5.0846914081	-3.9333738371	-0.5031498889
C	-2.9735139041	-2.4667249769	0.8525116776
C	-2.6275342025	-3.8962458037	0.6893068968
H	-3.4924544383	-4.5030605786	0.4171876736
H	-2.1726707440	-4.3048966417	1.5962617677
O	-0.6762190142	-2.0039444939	-3.2965772052
C	0.3198863181	-2.7886326130	-3.3660736656
C	1.5462712134	-2.4436812510	-4.1823155297
N	0.4097498970	-3.9307778300	-2.7207662194
C	2.3958811324	-3.6996003688	-4.0392626632
H	1.9924891650	-1.5494048704	-3.7140057001
H	1.2656046428	-2.1791656453	-5.2103809304
C	1.6260609341	-4.5414476464	-3.0123331156
Br	-1.2884068165	-4.0833972892	-0.7675653150
H	2.4871845667	-4.2787051494	-4.9698861003
H	3.4164580984	-3.5153931138	-3.6699313783
O	2.0134875358	-5.5757120486	-2.5266802870
C	-2.1101971676	-1.6026017380	1.6234197417
C	-0.9616266492	-2.1180700493	2.2724022697
C	-2.3465597452	-0.2097175328	1.7155673363
C	-0.1259025655	-1.2929060617	3.0056623491
H	-0.6941716542	-3.1711751024	2.1858851363
C	-1.5234991328	0.6038963535	2.4721523625
H	-3.1500891603	0.2427278009	1.1385381615
C	-0.4089912348	0.0673429943	3.1154662991
H	0.7697264125	-1.7092389279	3.4702265653
H	-1.7176919592	1.6734235718	2.5279951016
H	0.2551941264	0.7264545789	3.6784848682
C	-7.5724441732	-2.2384734615	0.7843419457
H	-8.0730651092	-3.2107299262	0.6636384216
H	-7.9882614834	-1.7562417258	1.6819436340
H	-7.8195815041	-1.6159856577	-0.0890095498
C	-5.7043701743	-3.3047971672	2.0973205805
C	-5.0672227310	-2.8436486179	3.2538541451
C	-5.9989312813	-4.6747505713	2.0146450417
C	-4.7165219948	-3.7208311222	4.2829409318
H	-4.8221537099	-1.7865901356	3.3644786617
C	-5.6558535681	-5.5519408230	3.0379114377
H	-6.4965028594	-5.0648884444	1.1225472288
C	-5.0044843753	-5.0776197933	4.1783054768
H	-4.2107060859	-3.3336000448	5.1700265756
H	-5.8916976895	-6.6140522553	2.9437508864
H	-4.7260203539	-5.7647419095	4.9797299500
Mg	-1.6323144764	-1.1726606160	-1.8222839285
O	1.8047498008	0.2658331150	-2.0265854912
O	-0.4296957490	-0.1618980244	-0.7163049447
P	0.9076881327	0.5500884392	-0.8857872503
O	0.5875580357	2.1775765296	-0.8803159996
O	1.6182030753	0.3942536696	0.5854688505
C	0.1086528955	2.8049884677	0.2341444788
C	2.6737805315	1.2308450276	0.8591888960
C	0.9607840399	2.9714166027	1.3169757393
C	-1.2191035294	3.3244190229	0.2396963297
C	2.3799417494	2.5392527667	1.2071083221
C	4.0083026173	0.7479640213	0.7644484681
C	0.4556012015	3.5661095909	2.5181897933
C	-1.6496742327	4.0211283448	1.3473427069
Si	-2.4256919773	3.2611140339	-1.2080323282

C	3.4539442024	3.4720789160	1.3935046849
C	5.0336116907	1.6322726659	1.0099603730
Si	4.5151642865	-1.0210823539	0.3380387653
C	1.2036317961	3.5993039761	3.7302867142
C	-0.8526752959	4.1277982384	2.5193973608
H	-2.6407582595	4.4932075093	1.3682065172
C	-2.1485853830	1.8756489486	-2.4345985032
C	-4.1962546278	3.0488390229	-0.5900785462
C	-2.2200300773	4.9218355954	-2.0532962689
C	3.2444668184	4.8625647828	1.6172579532
C	4.7938702215	3.0006830841	1.3118096926
H	6.0817265297	1.3088709728	0.9599038746
C	4.8351271510	-1.2393798066	-1.4924653802
C	6.1605520837	-1.2660902081	1.2371394549
C	3.3242099064	-2.3309675382	0.9487228065
C	0.6883416584	4.1808124399	4.8628615627
H	2.1974772209	3.1497959524	3.7521452240
C	-1.3490746738	4.7430118979	3.7017659871
C	-3.0819597625	0.8305547918	-2.5690826117
C	-1.0346289973	1.9049130249	-3.2901813857
C	-4.5308530485	2.6109189196	0.6989977572
C	-5.2352051219	3.2789798360	-1.5117079283
C	-0.9754833753	5.2030586233	-2.6471896595
C	-3.2141771576	5.9114197067	-2.0706568735
C	4.3045619786	5.7184145580	1.7920828533
H	2.2275894323	5.2546281735	1.6382995460
C	5.8696432766	3.9088753438	1.5083034308
C	5.4709519523	-2.4224592797	-1.9189952166
C	4.4944336743	-0.2724956741	-2.4476338046
C	6.1953838357	-1.4044225956	2.6351244029
C	7.3773003458	-1.2694980178	0.5358137613
C	3.1283694316	-2.5632235570	2.3220393771
C	2.6992110819	-3.1864080110	0.0277735568
C	-0.5980496984	4.7723067259	4.8497662810
H	1.2770962037	4.1880554961	5.7825130292
H	-2.3498297455	5.1824164898	3.6823371909
C	-2.9254245940	-0.1449323909	-3.5757647118
H	-3.9602501391	0.7612905172	-1.9115227017
C	-0.8540278797	0.9195447372	-4.2663201146
H	-0.2759322345	2.6946976924	-3.1906945704
C	-5.8685718062	2.4208875337	1.0683529517
H	-3.7530364708	2.4097467935	1.4381719699
C	-6.5714081193	3.0914696901	-1.1450234372
H	-5.0115868101	3.6039721868	-2.5331757385
C	-0.7396766025	6.4379018171	-3.2587260946
H	-0.1704284888	4.4563163832	-2.6347294415
C	-2.9789356735	7.1485160550	-2.6833215330
H	-4.1847747291	5.7375127653	-1.6021951850
C	5.6349373377	5.2389222463	1.7509586190
H	4.1177556028	6.7815500845	1.9577645725
H	6.8922393193	3.5261383960	1.4538445884
C	5.7829581043	-2.6177671997	-3.2664879245
H	5.7292843697	-3.2057006126	-1.1994741053
C	4.8008887294	-0.4726336750	-3.8020569868
H	3.9604124357	0.6421534951	-2.1597094341
C	7.4094066715	-1.5529621220	3.3134828205
H	5.2663355816	-1.4021335723	3.2155674127
C	8.5943967372	-1.4184505146	1.2115515071
H	7.3957024203	-1.1556641735	-0.5516523230
C	2.3619788571	-3.6459431881	2.7629429595

H	3.5911457612	-1.9049613096	3.0638640052
C	1.9441148147	-4.2818865762	0.4674149098
H	2.7971948267	-3.0178561688	-1.0492550992
H	-0.9910031482	5.2406054770	5.7541855178
C	-1.7994745407	-0.0985421150	-4.4201554656
H	-3.7014878785	-0.9010124372	-3.7324603737
H	0.0359979631	0.9368812634	-4.8950324139
C	-6.8907181215	2.6636110981	0.1478728333
H	-6.1120033096	2.0884893525	2.0751745889
H	-7.3643054121	3.2785451838	-1.8669532161
C	-1.7432661884	7.4120130013	-3.2798831492
H	0.2268155657	6.6420509776	-3.7155120575
H	-3.7594772144	7.9060693154	-2.6924562622
H	6.4680944822	5.9288298013	1.8977478922
C	5.4501080466	-1.6380314148	-4.2105175893
H	6.2832699572	-3.5299974327	-3.5825669299
H	4.5235701167	0.2851725190	-4.5312910553
C	8.6126479057	-1.5617904708	2.6012909935
H	7.4176860246	-1.6638086627	4.3952468016
H	9.5271157882	-1.4245934621	0.6516885253
C	1.7758536778	-4.5133935395	1.8333893452
H	2.2396296726	-3.8289727382	3.8269458007
H	1.5080829025	-4.9651550927	-0.2700354061
H	-1.6661546862	-0.8625242008	-5.1890093980
H	-7.9309376681	2.5218420630	0.4345231572
H	-1.5603124277	8.3729236391	-3.7567929900
H	5.6950477174	-1.7893634479	-5.2595747667
H	9.5576559016	-1.6805283287	3.1266315978
H	1.2047644052	-5.3741369154	2.1726685729

TS2b1
0 1

O	-2.5965405453	0.0725331975	-0.1240065408
C	-3.7883840327	-0.1976658965	-0.6092394322
C	-4.2001925942	0.4808338668	-2.1007989921
C	-4.9655417215	0.6359506754	-0.0405821334
C	-5.3695667885	1.2187249688	-1.4206697378
H	-3.3174213703	1.0929995743	-2.3338111367
H	-4.4310872481	-0.1585293777	-2.9623446074
H	-4.5434781604	1.3945055720	0.6326256399
H	-5.7275520701	0.0805821486	0.5192609185
C	-4.0570575689	-1.5564690229	-1.0980901240
C	-5.3947778750	-2.1435888507	-0.8304302560
H	-6.2142044353	-1.4206561910	-0.8354468300
H	-5.6687973454	-3.0111173292	-1.4326752085
O	-2.3214279748	-0.6489110000	3.1230966161
C	-3.1286895847	-1.2197647759	3.9222854798
C	-3.1966794723	-0.7967962936	5.3861809094
N	-3.9517036865	-2.1919117334	3.6097780674
C	-4.2582112122	-1.7284942542	5.9518416557
H	-2.2021965209	-0.9101735548	5.8421357614
H	-3.4553976606	0.2717624705	5.4440227616
C	-4.6950435283	-2.5517827628	4.7275869700
Br	-5.0303198295	-2.6472135924	1.0388030743
H	-5.1344827031	-1.2151207193	6.3719772138
H	-3.8835406813	-2.4133891987	6.7259416007
O	-5.5656751709	-3.3863541405	4.7389898782
C	-5.2289558662	2.7427796119	-1.4983851901
H	-5.9813633316	3.2300026441	-0.8599902281
H	-5.3772131786	3.0948090830	-2.5305222477

H	-4.2283132070	3.0585165851	-1.1644705106
C	-6.7539586311	0.8188967982	-1.9130799664
C	-6.9959862115	0.5588851417	-3.2666678941
C	-7.8376997657	0.7697555597	-1.0272805258
C	-8.2745898328	0.2358478024	-3.7190564394
H	-6.1765507296	0.6135525455	-3.9878493984
C	-9.1173941171	0.4462857296	-1.4747694683
H	-7.6833478212	0.9860439011	0.0328289668
C	-9.3399775650	0.1723186434	-2.8232328435
H	-8.4376738947	0.0326480892	-4.7796409691
H	-9.9450616108	0.4074063085	-0.7634607889
H	-10.3407810088	-0.0861729804	-3.1746916263
C	-3.0430030839	-2.3423242807	-1.7864997773
C	-3.3172979189	-3.6679496528	-2.2061714965
C	-1.7730837501	-1.8001971442	-2.0921821950
C	-2.3729643896	-4.4040962453	-2.9008286310
H	-4.2648202499	-4.1473119539	-1.9653150902
C	-0.8345790916	-2.5407020252	-2.8021898790
H	-1.5035203623	-0.8015583399	-1.7516931044
C	-1.1298668310	-3.8386447268	-3.2067734513
H	-2.6003180274	-5.4280970816	-3.2016502717
H	0.1451463277	-2.0923857607	-2.9689008415
H	-0.3802719378	-4.4242384762	-3.7462615073
Mg	-1.4293144742	-0.1497271523	1.4100880195
O	0.3293708177	0.4287500882	0.8199087735
P	1.4130247512	0.1448294003	-0.2126403872
O	1.1665986510	-0.7631064649	-1.3604402688
O	1.9981919148	1.5489760616	-0.8339302500
O	2.6991414097	-0.2632333787	0.7273333746
C	2.7902206865	2.4187461341	-0.1315564095
C	3.8947511916	-0.4122173135	0.0713782111
C	4.0876489731	2.0530308846	0.1966468966
C	2.2524547126	3.6935051315	0.1892217473
C	4.6142309012	0.7295799752	-0.2371326448
C	4.3138314348	-1.7157530597	-0.2987485046
C	4.8754290668	2.9607549547	0.9808732059
C	3.0436695000	4.5949583820	0.8621211123
Si	0.4265001671	4.0692829528	-0.0910481159
C	5.8115976175	0.5957327978	-1.0159178646
C	5.5022132093	-1.8444028713	-0.9787392015
Si	3.2776795882	-3.2725149814	-0.0318977411
C	6.1534882226	2.6123677416	1.5041190599
C	4.3584052272	4.2538394699	1.2820496228
H	2.6680452633	5.5916018822	1.1259513242
C	-0.2879091252	3.6782503504	-1.7769641710
C	-0.4984639608	3.1674733923	1.2655990728
C	0.1931141751	5.9186446324	0.1598378337
C	6.5453635554	1.7142249422	-1.5037595875
C	6.2712457165	-0.7092128442	-1.3572703807
H	5.8711914083	-2.8316195805	-1.2860879495
C	3.0967066846	-4.0395534520	-1.7360145544
C	4.2344371270	-4.4380104101	1.0873801835
C	1.5581137694	-3.0396443205	0.6855028362
C	6.8873304320	3.5100426044	2.2398696394
H	6.5474693231	1.6120754840	1.3220427141
C	5.1489808484	5.1661505604	2.0339452245
C	-1.5803130753	4.1662187972	-2.0546980614
C	0.3763287646	2.9331012462	-2.7601924790
C	0.0395551780	3.1793884841	2.5630827559
C	-1.7278552246	2.5242314655	1.0423568133

C	0.7120737136	6.7926181811	-0.8121226023
C	-0.4723915696	6.4586845344	1.2699920222
C	7.6903991874	1.5430848619	-2.2424843708
H	6.1848553421	2.7209619479	-1.2924562850
C	7.4673628481	-0.8528309663	-2.1128874534
C	3.3959630414	-5.3799798953	-2.0170352627
C	2.6134766592	-3.2150940921	-2.7683289583
C	3.6727499226	-4.9383324750	2.2724579282
C	5.5371342873	-4.8371602601	0.7384577251
C	1.3085888820	-2.3084886923	1.8543444545
C	0.4699372235	-3.6249326996	0.0080575643
C	6.3894473876	4.8093328643	2.4989949111
H	7.8624455828	3.2156176796	2.6339563191
H	4.7419746400	6.1588209845	2.2443481821
C	-2.1956578823	3.8988156948	-3.2806050937
H	-2.1164258409	4.7716620540	-1.3182885499
C	-0.2409790419	2.6638371827	-3.9896039426
H	1.3867977945	2.5426761365	-2.5832178036
C	-0.6483681614	2.5860436033	3.6290111055
H	1.0107011791	3.6435924850	2.7612487361
C	-2.4452859086	1.9735684131	2.1196134376
H	-2.1522805716	2.4516933019	0.0313739925
C	0.5705706176	8.1769428993	-0.6721290140
H	1.2302756715	6.4006077492	-1.6921697213
C	-0.6151759772	7.8447102744	1.4103219224
H	-0.8902294857	5.8062585322	2.0401849268
C	8.1691094135	0.2455498929	-2.5416876134
H	8.2330510007	2.4176575597	-2.6077896229
H	7.8121762179	-1.8608832360	-2.3576866597
C	3.2211206832	-5.8909345236	-3.3094324135
H	3.7682589168	-6.0426040225	-1.2320509238
C	2.4410017831	-3.7256605068	-4.0588048549
H	2.3572356684	-2.1600570564	-2.5663424052
C	4.3957486466	-5.8147576381	3.0915749647
H	2.6614593438	-4.6540244527	2.5750977169
C	6.2600468251	-5.7123020363	1.5548414603
H	6.0049685523	-4.4661952898	-0.1774516806
C	-0.0042677209	-2.1408894486	2.3325085853
H	2.1332433687	-1.8400286119	2.4052785943
C	-0.8315866525	-3.4719778558	0.4849132406
H	0.6281757319	-4.2061005087	-0.9054357546
H	6.9861222952	5.5163709881	3.0788455161
C	-1.5258051426	3.1433627858	-4.2500630354
H	-3.1908973422	4.2849808405	-3.4858423467
H	0.2868892147	2.0769648974	-4.7386684139
C	-1.8935930359	1.9951930208	3.4110228196
H	-0.2099398580	2.5857873627	4.6245072650
H	-3.4572891487	1.5734422236	1.9572170793
C	-0.0933755699	8.7044997232	0.4403333447
H	0.9755223848	8.8440716781	-1.4301273460
H	-1.1337042663	8.2516653537	2.2756062196
H	9.0841585550	0.1228463467	-3.1245842091
C	2.7464373781	-5.0635855607	-4.3306469020
H	3.4586520171	-6.9313866267	-3.5177115717
H	2.0806095388	-3.0769227237	-4.8539195237
C	5.6893694729	-6.2022538289	2.7343221427
H	3.9474741840	-6.1933187240	4.0077965125
H	7.2675583449	-6.0110521648	1.2744489510
C	-1.0762547836	-2.7296983317	1.6477773871
H	-0.1685573318	-1.6148750222	3.2818240995

H	-1.6636016794	-3.9447789351	-0.0343708813
H	-2.0032647959	2.9369905621	-5.2052821998
H	-2.4446299711	1.5512230192	4.2410056315
H	-0.2039384811	9.7815363470	0.5487274960
H	2.6195547178	-5.4579306873	-5.3365013850
H	6.2518943992	-6.8820907193	3.3708847572
H	-2.1000400974	-2.7013454123	2.0524236572

TS2b2
0 1

O	-2.7139192827	0.1724743427	0.3560995530
C	-4.0248449090	0.0122096137	0.3943398918
C	-4.9313646817	1.3226180293	0.8827731641
C	-4.7956509747	0.1218482297	-0.9488383341
C	-5.6808996266	1.3007527413	-0.4653328781
H	-4.1904035158	2.1276676777	0.9918391987
H	-5.5328818454	1.2606841253	1.7997680448
H	-4.0801442665	0.4189991257	-1.7290159582
H	-5.3032522168	-0.7865292377	-1.2949029632
C	-4.5613441986	-0.9124655656	1.4160466693
C	-5.7262757686	-1.7543012351	1.1091401443
H	-6.4275602077	-1.3308830434	0.3856857842
H	-6.2612707794	-2.1732546196	1.9643447273
Mg	-1.0706980106	-0.6740832412	-0.3430766696
O	0.2068811748	0.6585933509	0.7866790131
O	1.0165981425	-0.7631977188	-1.0739850794
O	-1.8151933464	-2.1230448193	-1.5053312287
C	-2.0626070397	-3.2419927756	-2.0317706307
C	-1.4212155160	-3.6569447603	-3.3497323678
N	-2.8706669825	-4.1552808558	-1.5284833554
C	-1.9662198928	-5.0628399966	-3.5586109763
H	-0.3258441576	-3.5972490099	-3.2544157500
H	-1.7148282268	-2.9418874117	-4.1323260597
C	-2.9234695143	-5.2590900038	-2.3703286626
Br	-4.6326431506	-3.1209281980	0.1539982751
H	-2.5203395112	-5.2041210946	-4.4971125533
H	-1.1929625436	-5.8446358072	-3.5202647388
O	-3.6160340819	-6.2317361027	-2.2007065957
C	-5.4799285256	2.5835289244	-1.2779487125
H	-5.8891698612	2.4644480368	-2.2927202260
H	-5.9931451646	3.4331423082	-0.8013423827
H	-4.4095047270	2.8273928165	-1.3619339682
C	-7.1695782997	0.9974361334	-0.3703456341
C	-7.9527443881	1.5162344500	0.6669061848
C	-7.8066828232	0.2432983112	-1.3639726089
C	-9.3240225563	1.2706924389	0.7247069291
H	-7.4900291403	2.1299367529	1.4441064126
C	-9.1768822001	-0.0059620486	-1.3098433367
H	-7.2245016620	-0.1590056491	-2.1967765912
C	-9.9410246350	0.5027542469	-0.2608682449
H	-9.9133832867	1.6835662666	1.5464415375
H	-9.6492975117	-0.6024630261	-2.0932456442
H	-11.0137887187	0.3047433378	-0.2143396126
C	-3.9466307940	-0.9907930389	2.7417154270
C	-4.3102409184	-2.0075725322	3.6506790748
C	-3.0197169588	-0.0128271958	3.1642761208
C	-3.7774039066	-2.0396062624	4.9313043902
H	-4.9837685696	-2.8092446178	3.3465860329
C	-2.5161968763	-0.0324699667	4.4595083405
H	-2.6832962155	0.7424345236	2.4537970560

C	-2.8904108037	-1.0433581877	5.3437288622
H	-4.0553397674	-2.8454798819	5.6128831838
H	-1.8066293740	0.7361938631	4.7709054356
H	-2.4797810859	-1.0646388319	6.3556496865
P	1.4114330201	0.2755688024	-0.0475184439
O	2.1395275262	1.5068330184	-0.8230856643
O	2.6186622542	-0.1352121970	0.9594010578
C	2.6936459377	2.4963334335	-0.0393102837
C	3.8955730509	-0.2206262750	0.4442585462
C	3.9219893851	2.2498855714	0.5497075479
C	1.9601345031	3.6949465066	0.1503930274
C	4.5983017496	0.9564521401	0.2487139670
C	4.4070647276	-1.4994572126	0.1054466865
C	4.4480975601	3.2274544612	1.4549653167
C	2.4963834215	4.6538657503	0.9776573911
Si	0.2816939084	3.9953436400	-0.6717514398
C	5.9020754839	0.8824332502	-0.3427982130
C	5.6773492258	-1.5682918780	-0.4171661773
Si	3.3921984457	-3.0911044729	0.2317814925
C	5.6399851793	3.0155394869	2.2036099064
C	3.7325317512	4.4452250239	1.6517595801
H	1.9677020999	5.5969882818	1.1609286834
C	0.4307711506	5.5083096552	-1.7777106311
C	-0.2432705767	2.5806988688	-1.7763453823
C	-0.9644105296	4.3333823536	0.6841025808
C	6.6597642818	2.0383864534	-0.6846625578
C	6.4515279889	-0.3973600919	-0.6479750244
H	6.1144625551	-2.5326537946	-0.7033867062
C	3.2447040141	-3.7923939480	-1.4969899271
C	4.3075438212	-4.3038136263	1.3377575407
C	1.7244870547	-2.8127195770	1.0284682398
C	6.1122783220	3.9737000695	3.0666131117
H	6.1768836169	2.0730574853	2.0902422643
C	4.2545407282	5.4204860755	2.5454958948
C	-0.7188341389	5.9330966998	-2.4691309078
C	1.6308147995	6.2077043516	-1.9703321952
C	-1.3859885121	1.7911799746	-1.5612917639
C	0.6034165063	2.2710312944	-2.8561195842
C	-1.5332949093	5.6038290368	0.8672210593
C	-1.3143003055	3.2969489655	1.5673402072
C	7.9063901075	1.9258395983	-1.2497482960
H	6.2372603514	3.0261675983	-0.5001454126
C	7.7487426821	-0.4792365392	-1.2248535663
C	2.5203172727	-3.0744610877	-2.4647636353
C	3.8589262658	-4.9994423318	-1.8680291434
C	5.4989667203	-3.9916355275	2.0081746961
C	3.7394716449	-5.5777519062	1.5236383670
C	1.7290611781	-2.3187006057	2.3454503003
C	0.4944621286	-3.0308155349	0.3851231827
C	5.4200153023	5.1966612888	3.2344717506
H	7.0271375993	3.7877770672	3.6330117842
H	3.7009365180	6.3531054981	2.6817586407
C	-0.6691415049	7.0394558738	-3.3227248423
H	-1.6662351195	5.3995730609	-2.3518525748
C	1.6816977638	7.3159050638	-2.8254935911
H	2.5459011581	5.8963383567	-1.4617812453
C	-1.6620801956	0.7017712577	-2.4025151526
H	-2.0818428094	2.0180494969	-0.7382544430
C	0.3422442157	1.1668684210	-3.6782244176
H	1.4980326572	2.8711738951	-3.0536931277

C	-2.4397280303	5.8371751454	1.9082892293
H	-1.2765547291	6.4315520835	0.2011860384
C	-2.2160178981	3.5335879094	2.6109053388
H	-0.8831427365	2.2852460531	1.4489208644
C	8.4669267509	0.6536488930	-1.5135611656
H	8.4672530498	2.8274015960	-1.5046088430
H	8.1599073155	-1.4675399499	-1.4458664771
C	2.4115009708	-3.5576886539	-3.7731678336
H	2.0259327470	-2.1212270301	-2.2088107264
C	3.7464917214	-5.4844294702	-3.1763443188
H	4.4347280355	-5.5791030392	-1.1425876947
C	6.1196789222	-4.9348568794	2.8369388913
H	5.9585576044	-3.0070144355	1.8995152112
C	4.3586983403	-6.5200818470	2.3506251431
H	2.8021728447	-5.8468538059	1.0270187853
C	0.5271810510	-2.0371141897	3.0048857592
H	2.6731805498	-2.1283047313	2.8667258417
C	-0.7135401506	-2.7730853616	1.0539070151
H	0.4613659688	-3.4146552439	-0.6406192644
H	5.8081611570	5.9519617343	3.9205195118
C	0.5322721802	7.7338293314	-3.5004844397
H	-1.5641241094	7.3586581704	-3.8522074523
H	2.6193221599	7.8494523771	-2.9656540535
C	-0.7813083924	0.3736898565	-3.4426534807
H	-2.6022032017	0.1336889792	-2.2873805289
H	1.0276554137	0.9194534843	-4.4857117271
C	-2.7803173956	4.8015120261	2.7821985671
H	-2.8745533552	6.8251664507	2.0382236123
H	-2.4703095174	2.7319467937	3.2990430940
H	9.4604098922	0.5778776689	-1.9595965570
C	3.0228006105	-4.7634223459	-4.1302605706
H	1.8567647104	-2.9878944380	-4.5152727510
H	4.2236314549	-6.4223836936	-3.4501653724
C	5.5517975555	-6.1998922165	3.0070488725
H	7.0443641314	-4.6802037470	3.3499843017
H	3.9105484660	-7.5022225785	2.4858115747
C	-0.6891335029	-2.2711850241	2.3607936123
H	0.5434765726	-1.6340861042	4.0140257403
H	-1.6727546872	-3.0679125916	0.5988132279
H	0.5712530342	8.5944189677	-4.1651299409
H	-0.9795147766	-0.4982892254	-4.0637332888
H	-3.4790727552	4.9829808095	3.5957946780
H	2.9373763204	-5.1385075983	-5.1479945727
H	6.0338418917	-6.9334034091	3.6499791412
H	-1.6244795181	-2.0949221518	2.8867751580

TS2c1
0 1

O	-2.6042633426	0.6803082257	0.0488968659
C	-3.1910993631	-0.1933617252	-0.7853914975
C	-2.4309881064	-1.5131631655	-1.1165724768
C	-3.0870374679	0.1658788311	-2.3411701363
C	-2.4575215726	-1.2157107587	-2.6414769973
H	-1.4099922403	-1.4578144493	-0.7160645813
H	-2.8978368276	-2.4467442450	-0.7838653561
H	-2.3794687951	1.0050137482	-2.4117543154
H	-4.0075795695	0.4293452853	-2.8818975685
C	-1.0606931435	-1.1294182915	-3.2666590251
H	-1.1230607602	-0.7226476238	-4.2883991749
H	-0.6054800698	-2.1292137820	-3.3180443078

H	-0.4017580490	-0.4966044127	-2.6562753654
C	-3.3187308580	-2.1245261315	-3.5058693145
C	-3.4505679884	-3.4896511853	-3.2370592543
C	-3.9272464726	-1.6225319737	-4.6637415337
C	-4.1838935619	-4.3219386029	-4.0820231722
H	-2.9743766941	-3.9147487009	-2.3506002918
C	-4.6594232590	-2.4485912349	-5.5126976006
H	-3.8223342154	-0.5621599002	-4.9099653463
C	-4.7953860791	-3.8054544686	-5.2219163347
H	-4.2780848239	-5.3839941532	-3.8438079889
H	-5.1247455198	-2.0305590186	-6.4083942545
C	-4.6819516184	-0.3067571798	-0.5853359978
C	-5.3711595549	0.8023945266	-0.0427603946
H	-4.8184310273	1.7427845022	0.0051255313
H	-6.4562199094	0.8498479503	-0.1631589400
C	-5.4723165020	-1.4661057097	-1.0595251126
C	-5.3950749577	-2.7135421943	-0.4268324184
C	-6.3158362537	-1.3066313342	-2.1651820758
C	-6.1499007605	-3.7813494455	-0.8979669945
H	-4.7631107022	-2.8346935099	0.4543365110
C	-7.0379156253	-2.3897197942	-2.6579338443
H	-6.3812276497	-0.3369099349	-2.6636248746
C	-6.9593277163	-3.6260384563	-2.0225568520
H	-6.0976879355	-4.7448365821	-0.3878191069
H	-7.6629831610	-2.2647225954	-3.5433456925
O	-2.1277703940	0.0411040931	3.3969003044
C	-2.9509014430	-0.4572093767	4.1966367033
C	-2.5447210418	-0.9627275519	5.5694256569
N	-4.2517791618	-0.6038150939	3.9789721768
C	-3.8545798443	-1.4672182299	6.1633271191
H	-1.7707322704	-1.7420790386	5.4355119091
H	-2.0750386584	-0.1423205357	6.1296647035
C	-4.8917099970	-1.1975997874	5.0664861480
Br	-4.9762384243	-0.0398006527	1.8807743374
H	-4.1617779728	-0.9421864619	7.0782948444
H	-3.8553284900	-2.5419777920	6.3924190886
O	-6.0642446941	-1.4512344766	5.1254246798
H	-5.3707868978	-4.4570406706	-5.8830491918
H	-7.5311134388	-4.4732820479	-2.4055983833
Mg	-1.5736329094	0.8066781785	1.6318098563
O	0.3460695916	0.6658123888	1.3782602825
P	1.2131378141	0.0613690864	0.2771318852
O	0.7540411523	-1.1095702057	-0.5077022671
O	1.5893662876	1.2687067114	-0.7889762996
O	2.6787044884	-0.1309615752	0.9824214048
C	2.3911413666	2.3138876250	-0.4095061310
C	3.7302879263	-0.3661142254	0.1254888161
C	3.7454249441	2.0826541619	-0.2212628931
C	1.8124517335	3.6030263292	-0.2464018306
C	4.2925845518	0.7289029061	-0.5074280190
C	4.1639594685	-1.6975562370	-0.1106612418
C	4.5701068899	3.1492365490	0.2630512850
C	2.6250793757	4.6435243177	0.1455250965
Si	-0.0242757763	3.9428124801	-0.5033195025
C	5.3261615939	0.5165458880	-1.4776072426
C	5.1949105883	-1.8899423172	-1.0010519089
Si	3.3973622060	-3.2498222922	0.6461693022
C	5.9356521878	2.9607868886	0.6203712373
C	4.0040358091	4.4455860334	0.4267601558
H	2.2259804191	5.6581994292	0.2746504552

C	-0.7088727954	3.3650909914	-2.1455656290
C	-0.9918563709	3.3029004465	0.9755985830
C	-0.2429557300	5.8142736561	-0.4777784527
C	5.8800710108	1.5684679125	-2.2612043544
C	5.7945769886	-0.8090141953	-1.7036350712
H	5.5690007975	-2.9011056077	-1.2157519007
C	2.7700478137	-4.3206506804	-0.7555350544
C	4.7782669904	-4.1688643409	1.5426601241
C	2.0326733493	-2.9530002407	1.8900004553
C	6.6977672538	4.0079437330	1.0764869068
H	6.3742712699	1.9657076769	0.5353786853
C	4.8228937959	5.5123941650	0.8897342896
C	-1.9232214230	3.9206380957	-2.5946414826
C	-0.0380208857	2.4551968554	-2.9748025852
C	-0.3277071997	3.0450674364	2.1947805029
C	-2.3984619583	3.2350831964	0.9373095227
C	0.0933661406	6.5515625041	-1.6268852442
C	-0.7061556313	6.5015036326	0.6543997485
C	6.8695635246	1.3198202102	-3.1806487795
H	5.5059638693	2.5840988934	-2.1315977461
C	6.8268831229	-1.0321844606	-2.6556407853
C	3.1843343649	-5.6496118714	-0.9376254059
C	1.8293687825	-3.7728414532	-1.6451252188
C	4.6725829270	-4.4912557174	2.9046426016
C	5.9350699372	-4.5666377940	0.8486157549
C	2.2042080555	-2.0207778364	2.9284433739
C	0.8507668371	-3.7086759647	1.8407790167
C	6.1421361279	5.3039696090	1.2036486727
H	7.7418619681	3.8380564879	1.3487050162
H	4.3764995089	6.5046415964	0.9986615818
C	-2.4472016679	3.5772643517	-3.8438152908
H	-2.4679421241	4.6404464710	-1.9782439741
C	-0.5572034638	2.1200336675	-4.2330648164
H	0.9006511651	1.9854325650	-2.6526551431
C	-1.0621134442	2.7614054779	3.3578410641
H	0.7684652917	3.0551531689	2.2496194011
C	-3.1311481744	2.9638810915	2.1047130907
H	-2.9356622929	3.3887184929	-0.0040925603
C	-0.0320819202	7.9445349185	-1.6416833640
H	0.4548400532	6.0444626849	-2.5264782281
C	-0.8331113273	7.8958570316	0.6401850036
H	-0.9760164173	5.9593658006	1.5636434701
C	7.3606005819	0.0070163030	-3.3754938856
H	7.2769919485	2.1429859539	-3.7716236426
H	7.1817982076	-2.0543230529	-2.8113537752
C	2.6781601755	-6.4150778083	-1.9944641151
H	3.9042600101	-6.1072678440	-0.2553232394
C	1.3237822587	-4.5389306126	-2.7012704682
H	1.4692314382	-2.7362775979	-1.5170957703
C	5.6966012608	-5.1894250719	3.5575605346
H	3.7879539964	-4.2044717941	3.4795484170
C	6.9584105434	-5.2639475052	1.4972053998
H	6.0509490434	-4.3311803016	-0.2142738327
C	1.2214824892	-1.8646853398	3.9108765561
H	3.0981581869	-1.3884827789	2.9649766404
C	-0.1302642821	-3.5584261716	2.8289938052
H	0.6765298655	-4.4183904196	1.0271015823
H	6.7612511121	6.1293032760	1.5612787527
C	-1.7599992003	2.6788322271	-4.6684049563
H	-3.3831811966	4.0157038827	-4.1805611843

H	-0.0170055274	1.4202428571	-4.8685333260
C	-2.4643477392	2.7407403900	3.3161485480
H	-0.5396835819	2.5666201810	4.2941384496
H	-4.2202223597	2.9383749648	2.0744318220
C	-0.4960298600	8.6184743313	-0.5073801769
H	0.2306453391	8.5041371847	-2.5370633558
H	-1.1949694840	8.4166339995	1.5240746735
H	8.1508059623	-0.1764883090	-4.1063191801
C	1.7489411637	-5.8588207229	-2.8785874643
H	3.0072486698	-7.4424369656	-2.1266517609
H	0.5973863583	-4.1037844755	-3.3845561361
C	6.8403725604	-5.5764504310	2.8556238772
H	5.5989539448	-5.4299861537	4.6138304273
H	7.8472907425	-5.5628330022	0.9466139934
C	0.0606104019	-2.6425565734	3.8655631955
H	1.3569226875	-1.1297113963	4.6999915525
H	-1.0395225049	-4.1516066277	2.7837428359
H	-2.1611338307	2.4198721900	-5.6459511104
H	-3.0359670099	2.5491669515	4.2241433079
H	-0.5944562624	9.7021465276	-0.5191546011
H	1.3568264751	-6.4521455429	-3.7015735000
H	7.6361725824	-6.1180920915	3.3619994621
H	-0.7031091143	-2.5246202961	4.6383926227

TS2c2
0 1

O	-0.9487964326	-3.4097662783	-1.0164605411
C	-2.2023096366	-3.0306463661	-1.2034189397
C	-2.4695039059	-1.6710843753	-1.8975951342
C	-2.9494450662	-3.7308597205	-2.5313056267
C	-3.2183696367	-2.3158634608	-3.0924375825
H	-1.5088235796	-1.2237062805	-2.1761424831
H	-3.0237205496	-0.9338404763	-1.3076693549
H	-2.1212576067	-4.2759229689	-2.9973600618
H	-3.8142329737	-4.3976519786	-2.4223842164
C	-2.5184744305	-2.0685299779	-4.4336243693
H	-2.9683815240	-2.6773740311	-5.2325543132
H	-2.6153969621	-1.0095319586	-4.7161281501
H	-1.4484087518	-2.3127460864	-4.3640612145
C	-4.7011522065	-1.9961750863	-3.2340563766
C	-5.2505997822	-0.7904734304	-2.7953684692
C	-5.5445865248	-2.9075893388	-3.8835816180
C	-6.6044052558	-0.5100157779	-2.9794661195
H	-4.6203724803	-0.0505408512	-2.2933132286
C	-6.8963026189	-2.6330574380	-4.0706231927
H	-5.1386095164	-3.8537331119	-4.2525433704
C	-7.4344290841	-1.4304598258	-3.6134285415
H	-7.0085820769	0.4381242328	-2.6177078636
H	-7.5329297976	-3.3617546763	-4.5769310600
C	-3.2127784326	-3.5770689313	-0.2946850140
C	-2.8899892218	-4.7965133358	0.4122074459
H	-2.1105928686	-5.3997187215	-0.0600029167
H	-3.7434002757	-5.3594685638	0.8015439717
C	-4.5370155435	-2.9614910640	-0.0829942953
C	-4.6370223236	-1.6939518094	0.5060164698
C	-5.7018201161	-3.6552188798	-0.4363363609
C	-5.8897146920	-1.1270297927	0.7175926394
H	-3.7347216071	-1.1660611510	0.8287476629
C	-6.9479369344	-3.0648483637	-0.2624834305
H	-5.6298039195	-4.6486575687	-0.8847305501

C	-7.0417670509	-1.8001758851	0.3161792139
H	-5.9635379336	-0.1450732392	1.1888784877
H	-7.8480089708	-3.5937611859	-0.5787044163
Mg	0.5253970492	-2.6695871051	-0.0123181843
O	0.3494976826	0.3912842571	1.8573886287
O	0.7549034139	-0.7910886153	-0.4444273568
O	1.1863826711	-2.8904732237	1.8157439790
C	0.8141578302	-2.5319226749	2.9717875963
C	1.6924470182	-1.6969242470	3.8758010059
N	-0.3519776490	-2.8351125277	3.5105556700
C	0.7689839095	-1.3941499243	5.0480172977
H	2.0137788474	-0.8103514387	3.3128373967
H	2.5928017238	-2.2743956058	4.1415557045
C	-0.4852630883	-2.2265036244	4.7529856747
Br	-1.9293922696	-3.8794386736	1.9684971490
H	1.1671159813	-1.6545236930	6.0377146900
H	0.4603342034	-0.3361029540	5.0695326513
O	-1.4481575546	-2.3180395542	5.4729488063
H	-8.4948712445	-1.2121182831	-3.7541028249
H	-8.0205025009	-1.3384105656	0.4586546086
P	0.8972725478	0.4142612792	0.4839679628
O	2.4935182543	0.8063329608	0.5927809974
O	0.3622669219	1.6525857330	-0.4570767337
C	3.1936712853	1.1500216323	-0.5324651411
C	0.7234710125	2.9422236893	-0.1475085624
C	2.9557216782	2.3903377592	-1.0982990913
C	4.1487782870	0.2369963400	-1.0568792384
C	2.0242173059	3.3347120539	-0.4212568304
C	-0.2404064130	3.8252879809	0.4187012921
C	3.6080325105	2.7204979124	-2.3313085472
C	4.8292867652	0.5903008283	-2.2001171035
Si	4.4925313943	-1.4391198877	-0.2595099743
C	2.4591588068	4.6365491934	-0.0053682155
C	0.1682123926	5.0970267825	0.7505251107
Si	-2.0660377284	3.3934655693	0.6739870465
C	3.3178046894	3.9090157401	-3.0594871954
C	4.5657431163	1.8153969499	-2.8729548221
H	5.5851614033	-0.0741909656	-2.6382438248
C	4.7936554982	-1.3883292963	1.5868002263
C	3.1326361795	-2.6473242814	-0.7310961788
C	6.0757654754	-2.0966434768	-1.0358937555
C	3.8109692111	5.0697564136	-0.1132897397
C	1.5127631849	5.5259142168	0.5754926992
H	-0.5322886832	5.8238375749	1.1822487834
C	-2.3452696394	2.1185347240	2.0004907734
C	-2.9185941653	4.9792954701	1.2375833500
C	-2.8866212307	2.8986553394	-0.9435398002
C	3.9661855033	4.1935326841	-4.2361505929
H	2.5627765165	4.5961694178	-2.6755968113
C	5.2288171580	2.1451827096	-4.0871030663
C	5.3427402522	-2.5353436821	2.1934880715
C	4.5371569293	-0.2642759296	2.3842286068
C	2.4261640922	-2.4491764852	-1.9355024881
C	2.8847762025	-3.8126242812	0.0190991163
C	7.3056950692	-1.5615922089	-0.6136622418
C	6.0742372909	-3.0873431684	-2.0293237604
C	4.1862555594	6.3249120538	0.2996487547
H	4.5583703480	4.3892709348	-0.5215613920
C	1.9294774849	6.8224444308	0.9832473488
C	-3.5693101301	1.4288482485	2.0727636719

C	-1.3725107476	1.8784944310	2.9849181878
C	-3.1591946166	6.0155494963	0.3178480782
C	-3.3146873528	5.1549709793	2.5718677043
C	-2.1918413711	2.2575696625	-1.9794660933
C	-4.2455656056	3.2096206860	-1.1372099216
C	4.9422259688	3.3092662840	-4.7547313205
H	3.7230536305	5.1094933183	-4.7792600164
H	5.9701883656	1.4463167584	-4.4839596925
C	5.6144971861	-2.5578182816	3.5641257572
H	5.5757538034	-3.4233734354	1.5992470684
C	4.8164437397	-0.2836738671	3.7576202259
H	4.1071129663	0.6474395982	1.9512493892
C	1.5195026542	-3.4139244905	-2.3974764981
H	2.5666015416	-1.5322205638	-2.5196365251
C	1.9651038896	-4.7757111025	-0.4369414074
H	3.3911553766	-3.9825503544	0.9760215198
C	8.5064791984	-2.0079551226	-1.1751277413
H	7.3398023996	-0.7915783903	0.1628290568
C	7.2759782316	-3.5353561875	-2.5911466778
H	5.1361414084	-3.5258345612	-2.3771611968
C	3.2357801527	7.2199321937	0.8451185268
H	5.2304017776	6.6326817716	0.2111660289
H	1.1868970841	7.4968837898	1.4184094049
C	-3.8114952240	0.5134384167	3.1009603121
H	-4.3448284193	1.6036256336	1.3247917633
C	-1.6175380587	0.9587753026	4.0139673817
H	-0.4018904711	2.3827899374	2.9548015218
C	-3.7813505281	7.1997176915	0.7246361066
H	-2.8643957498	5.9059478622	-0.7292858978
C	-3.9380801649	6.3409766847	2.9805058608
H	-3.1449753978	4.3671121772	3.3116317944
C	-2.8295551542	1.9566260633	-3.1897771345
H	-1.1361093745	1.9813767980	-1.8609556510
C	-4.8843528418	2.9085988583	-2.3441984152
H	-4.8212792319	3.7030346405	-0.3489020436
H	5.4556624963	3.5508635499	-5.6876486273
C	5.3520753875	-1.4291800208	4.3490362500
H	6.0362211753	-3.4503294400	4.0207951063
H	4.6112727207	0.5984074777	4.3615333401
C	1.2939501576	-4.5839943292	-1.6542049946
H	0.9774187211	-3.2519677627	-3.3283659063
H	1.7889901495	-5.6768232819	0.1510195063
C	8.4926203326	-2.9958904325	-2.1650453877
H	9.4523613070	-1.5875712813	-0.8397622309
H	7.2619104169	-4.3055437916	-3.3591753454
H	3.5460096737	8.2167986858	1.1646803703
C	-2.8285454030	0.2681953719	4.0675501683
H	-4.7591125661	-0.0148689080	3.1565352377
H	-0.8382817609	0.7571317998	4.7513312900
C	-4.1716085161	7.3637535894	2.0581998449
H	-3.9632101697	7.9937544607	0.0039803563
H	-4.2408205409	6.4635513119	4.0182147232
C	-4.1732470427	2.2890291383	-3.3772935476
H	-2.2716901903	1.4694466539	-3.9863434582
H	-5.9312653111	3.1660053513	-2.4838704542
H	5.5684236247	-1.4446273932	5.4154685017
H	0.5850664652	-5.3295868916	-2.0149098961
H	9.4270148350	-3.3441456022	-2.6007234701
H	-2.9989541300	-0.4744894757	4.8510981669
H	-4.6560299906	8.2849586374	2.3748444178

H	-4.6661359775	2.0646121532	-4.3211196944

TS2d1
0 1

C	3.9743791950	1.3730508631	-1.1950737590
C	4.9092448584	2.2189133728	-2.1142403448
C	5.7667653723	2.7574345809	-0.9415658170
H	5.4439382030	1.6903201553	-2.9085991011
H	4.2779626542	2.9885011097	-2.5705024771
C	4.2636022232	-0.0527124087	-0.9359129099
C	3.1698796103	-0.8787270724	-0.4740913301
H	2.2484757369	-0.6811548290	-1.0424992544
H	3.4014813258	-1.9470580761	-0.4055214840
O	1.0332128519	2.5829179615	1.7305551168
C	0.8614637854	1.9440030699	2.8107933184
C	-0.3282306404	2.1997726766	3.7091093951
N	1.6587331414	0.9926514197	3.2578095448
C	-0.2255553297	1.0711941166	4.7253561249
H	-0.2509563749	3.2087436014	4.1431990881
H	-1.2411333198	2.1750738382	3.0952760171
C	1.1189841365	0.4149178627	4.4040049360
Br	2.6745404320	-0.1944255350	1.3880788261
H	-1.0000139491	0.3087846311	4.5650608430
H	-0.2565896151	1.3826597970	5.7777911308
O	1.6264764372	-0.4812657434	5.0305797685
O	2.6944657661	1.7202879667	-1.2168235170
C	4.8572508813	2.0249875843	0.0631398015
H	4.1404501075	2.6635348713	0.5933068976
H	5.3220874557	1.3440349443	0.7820212811
C	5.7068265390	4.2804916882	-0.7846725284
H	6.2284993614	4.7772986675	-1.6168046520
H	6.1920108395	4.5908473617	0.1532465589
H	4.6636395893	4.6300251107	-0.7685373141
C	7.2242806411	2.3174159901	-0.9755947077
C	7.9598203768	2.4777641615	-2.1572760042
C	7.8838687539	1.8123427164	0.1473211084
C	9.3033057495	2.1199829300	-2.2240114516
H	7.4714777066	2.8866820034	-3.0462918570
C	9.2292385513	1.4490979533	0.0850235153
H	7.3473107669	1.6870673447	1.0901075078
C	9.9435726402	1.5963009050	-1.1010516705
H	9.8539049061	2.2506672182	-3.1582575418
H	9.7192312750	1.0455489202	0.9737653138
H	10.9960663627	1.3096032514	-1.1499505714
C	5.6078493641	-0.6539685142	-0.9436558076
C	6.4487604937	-0.6081649244	-2.0641675230
C	6.0452736856	-1.3322134861	0.2100198733
C	7.7044955142	-1.2056685022	-2.0254002628
H	6.1131580182	-0.1355612383	-2.9853416368
C	7.3137194456	-1.8956974029	0.2541044909
H	5.3963747207	-1.3815391562	1.0878873931
C	8.1466462419	-1.8318239621	-0.8633164151
H	8.3460703585	-1.1660414108	-2.9066474935
H	7.6515405630	-2.3915128375	1.1656736189
H	9.1425876202	-2.2770881102	-0.8288515875
Mg	1.1224542170	1.9760033087	-0.1342023617
O	-0.4446524512	0.8968160000	-0.4799201797
P	-1.2718725785	-0.0107904292	0.4300282775
O	-0.7508082886	-0.4595184290	1.7414120891
O	-2.7362108199	0.6880839554	0.6942150255

O	-1.7017301493	-1.2218389075	-0.5917772235
C	-3.6460243171	0.8417024727	-0.3187065648
C	-2.6924575856	-2.0846237017	-0.1916339846
C	-4.2886911546	-0.2837564516	-0.8037898374
C	-3.9150703769	2.1504740066	-0.8035767218
C	-3.9971590807	-1.6187886329	-0.2093177604
C	-2.3510647035	-3.4069070491	0.2084169084
C	-5.1933408278	-0.1356844153	-1.9071858192
C	-4.8373612418	2.2961220890	-1.8142215677
Si	-3.0376083354	3.6678636977	-0.1049235627
C	-5.0302725417	-2.4356162562	0.3556585198
C	-3.3616079178	-4.2076634801	0.6907228068
Si	-0.6639032866	-4.2411427808	0.0036459667
C	-5.7995322237	-1.2469835389	-2.5592890280
C	-5.4815999120	1.1706193543	-2.3979982770
H	-5.0894527920	3.2868252204	-2.2135680593
C	-3.2234693644	3.8469041009	1.7491579046
C	-1.2383605455	3.6849875761	-0.6376126549
C	-3.8423987880	5.1800408770	-0.8836595691
C	-6.3694573386	-1.9815329398	0.5164090820
C	-4.7009411739	-3.7428288767	0.8115978763
H	-3.1630790812	-5.2398801622	1.0070421694
C	0.3019371472	-4.3508435992	1.5967285306
C	-1.0802521033	-5.9754655396	-0.6174624910
C	0.4720475476	-3.5158049863	-1.3096916429
C	-6.6666946963	-1.0653228537	-3.6086580785
H	-5.5632846987	-2.2562944002	-2.2204903059
C	-6.3925512689	1.3257223602	-3.4789442284
C	-2.9414302887	5.0973491419	2.3322395660
C	-3.6560575969	2.7999623755	2.5757802701
C	-0.8750894163	3.0608899929	-1.8471033239
C	-0.2625980900	4.3976222496	0.0776397303
C	-5.0846085820	5.6179860976	-0.3913870098
C	-3.2551733408	5.8745956333	-1.9519773688
C	-7.3277850214	-2.7928709914	1.0733824138
H	-6.6306833605	-0.9710973219	0.2004210113
C	-5.7175826880	-4.5618688191	1.3739892658
C	1.0843219481	-5.4877216310	1.8799384922
C	0.3370121224	-3.2674989967	2.4871700533
C	-1.1558773712	-6.2173507217	-1.9988311416
C	-1.3530618398	-7.0327309136	0.2668506177
C	0.0826559218	-2.5894244745	-2.2861978371
C	1.7859757881	-4.0220216846	-1.3496732219
C	-6.9790364673	0.2354690944	-4.0706741747
H	-7.1162804755	-1.9344433388	-4.0941354911
H	-6.6120545561	2.3357002614	-3.8358384473
C	-3.0809810332	5.2898839432	3.7094872098
H	-2.6188141974	5.9399345013	1.7144897898
C	-3.8013643264	2.9943899679	3.9560498213
H	-3.8768292848	1.8087753375	2.1599275470
C	0.4225931273	3.1997465657	-2.3610870081
H	-1.5962007926	2.4461419862	-2.3974529235
C	1.0399145473	4.5411706788	-0.4320553616
H	-0.4954773861	4.8482333895	1.0476145070
C	-5.7243839383	6.7255420686	-0.9572719773
H	-5.5652368260	5.1000478745	0.4438851980
C	-3.8947675270	6.9838921486	-2.5186395736
H	-2.2893092893	5.5619865676	-2.3556550354
C	-7.0049463079	-4.1027441875	1.5003478313
H	-8.3478663036	-2.4212237961	1.1925956293

H	-5.4516590481	-5.5675206255	1.7107748651
C	1.8731407487	-5.5423743518	3.0310943063
H	1.0893608407	-6.3447925398	1.2033160127
C	1.1385337457	-3.3189854186	3.6358772882
H	-0.2500145729	-2.3527534029	2.3024469214
C	-1.4921535595	-7.4865956427	-2.4849042557
H	-0.9511466966	-5.4180427840	-2.7163600786
C	-1.6876337904	-8.3018188411	-0.2170990548
H	-1.3044449915	-6.8791783081	1.3481932902
C	0.9823663703	-2.1703331943	-3.2757109558
H	-0.9306271135	-2.1701327887	-2.2896724637
C	2.6802590012	-3.6174262586	-2.3459728355
H	2.1270939799	-4.7448894410	-0.6018820699
H	-7.6754536908	0.3663741851	-4.9015053079
C	-3.5129265521	4.2368614691	4.5239019457
H	-2.8593330653	6.2598219691	4.1486982875
H	-4.1374873151	2.1709551674	4.5838509504
C	1.3782716838	3.9631529489	-1.6671219273
H	0.6937222457	2.7163863082	-3.2997552734
H	1.7778050611	5.1177394884	0.1251841002
C	-5.1296100839	7.4098568558	-2.0222712947
H	-6.6848103545	7.0561106091	-0.5672802178
H	-3.4285642090	7.5149548932	-3.3455934163
H	-7.7770656305	-4.7394247054	1.9370180984
C	1.9018945178	-4.4535080735	3.9113116610
H	2.4659672602	-6.4275500896	3.2437950513
H	1.1748006028	-2.4564700092	4.3102046295
C	-1.7576453617	-8.5305959561	-1.5948777890
H	-1.5465204546	-7.6590234767	-3.5574518922
H	-1.8931770373	-9.1116901455	0.4791822226
C	2.2785023083	-2.6883767187	-3.3118535898
H	0.6635419307	-1.4417367355	-4.0180254760
H	3.6838448765	-4.0337617042	-2.3759464716
H	-3.6251890834	4.3875928166	5.5958367568
H	2.3788918948	4.0929941616	-2.0838289624
H	-5.6269400811	8.2721880378	-2.4617877173
H	2.5201696407	-4.4896964349	4.8058927121
H	-2.0181090115	-9.5171071375	-1.9717370377
H	2.9694895487	-2.3781271508	-4.0913977601

TS2d2
0 1

C	-4.2652531333	-0.7343638590	-0.2046975305
C	-4.7834061253	-0.0450557223	1.0947550420
C	-4.5395358131	-1.2847523734	1.9898071325
H	-5.8018781275	0.3549335068	1.0781022188
H	-4.0888441907	0.7782119976	1.3013304376
C	-5.2086080039	-1.3886044028	-1.1322371302
C	-4.7721401116	-1.5651653367	-2.4942536962
H	-4.2456977119	-0.6654144609	-2.8504950100
H	-5.5305911231	-1.9548913882	-3.1782989055
Mg	-1.2745580064	-0.4033263884	-0.9540504266
O	-0.2366523818	0.9294592583	0.2862624623
O	0.7538647744	-0.0531303745	-1.7653414704
O	-0.7696439756	-2.2439273771	-0.4074985958
C	-0.5327795343	-3.4002556606	-0.8287463062
C	0.5525930091	-4.2683597381	-0.2310634179
N	-1.1843457446	-4.0101510615	-1.8127891069
C	0.4420427563	-5.5642656613	-1.0250176426
H	0.3898852234	-4.3692473584	0.8529071043

H	1.5172926195	-3.7469458329	-0.3529572513
C	-0.7092698870	-5.3037507791	-2.0026364280
Br	-3.1794387142	-2.8920165831	-2.3895170513
H	1.3486739208	-5.8006247760	-1.6027447931
H	0.2033363961	-6.4471406510	-0.4162348110
O	-1.1414563094	-6.0930301002	-2.8030199530
O	-3.1989755552	-0.1724273492	-0.7613668111
C	-3.9462333102	-2.0419932382	0.7866661654
H	-2.8533570127	-2.1319392532	0.7860739592
H	-4.3821494820	-3.0099940236	0.5214565816
C	-3.5032732784	-1.0474162973	3.0934427997
H	-3.9028812021	-0.3676330970	3.8613315385
H	-3.2401725750	-1.9970288146	3.5844684830
H	-2.5853800786	-0.6055555411	2.6769705274
C	-5.8019019926	-1.8769565331	2.6000479252
C	-6.7053060673	-1.0339613536	3.2605996202
C	-6.0758555964	-3.2466393001	2.5788383706
C	-7.8582295165	-1.5377622529	3.8560844462
H	-6.5056734848	0.0404406538	3.3058367900
C	-7.2309716480	-3.7567700628	3.1713562320
H	-5.3869684302	-3.9359750514	2.0861690575
C	-8.1300121908	-2.9049597839	3.8077667980
H	-8.5489810596	-0.8589170573	4.3614222625
H	-7.4277382392	-4.8304100249	3.1310152826
H	-9.0356112891	-3.3039614171	4.2695229986
C	-6.4761160093	-2.0269944479	-0.7335874914
C	-7.5010975483	-1.3297321578	-0.0803323471
C	-6.6686017295	-3.3853010502	-1.0476212223
C	-8.6816925699	-1.9785998623	0.2684072083
H	-7.3938632972	-0.2677275066	0.1343038530
C	-7.8354195146	-4.0356616761	-0.6668063426
H	-5.8772819615	-3.9385710511	-1.5592395050
C	-8.8442021073	-3.3335256021	-0.0065414028
H	-9.4711403926	-1.4219441668	0.7756220481
H	-7.9583537812	-5.0971898566	-0.8891508740
H	-9.7613651994	-3.8448063532	0.2920295576
P	0.9990570683	0.8728275564	-0.5974752354
O	2.2687052352	0.4777358342	0.3431565190
O	1.4801364526	2.3171541987	-1.1590239440
C	3.5713812778	0.8549206540	0.0885974827
C	1.6933175019	3.2781770541	-0.1903389047
C	3.8982892877	2.2036216377	0.1079273593
C	4.5463290659	-0.1618092774	-0.1387096201
C	2.8849218308	3.2216189189	0.5006870622
C	0.6737688432	4.2260730177	0.0756493911
C	5.2170149473	2.6053817714	-0.2902618548
C	5.8271151184	0.2370192311	-0.4496898011
Si	4.3202229745	-2.0313634817	0.0596148546
C	3.0826881435	4.1283397228	1.5942865307
C	0.8907877687	5.1318542045	1.0869987882
Si	-0.9823236163	4.2581834383	-0.8372858258
C	5.5878843823	3.9695867004	-0.4575114059
C	6.1883704578	1.6063638080	-0.5686731300
H	6.6232029151	-0.4978300566	-0.6283640817
C	3.2439031395	-2.5504753865	1.5052143507
C	3.6700685127	-2.8551099738	-1.4896789412
C	6.0397349158	-2.7188375064	0.4262067771
C	4.2234657838	4.0744763716	2.4430606129
C	2.0794094885	5.1026239355	1.8698627847
H	0.1327168146	5.8834168031	1.3397807713

C	-2.3081746007	4.1355238089	0.4774288606
C	-1.1379380368	5.8913273016	-1.7516655432
C	-1.1258746222	2.9047029639	-2.1182564317
C	6.8595461540	4.3126427066	-0.8474620547
H	4.8471616611	4.7500975464	-0.2831957491
C	7.4995571084	1.9919483378	-0.9591370867
C	3.2197020742	-3.9277452163	1.8066666867
C	2.4860548657	-1.6790790991	2.2973493855
C	2.8184587107	-2.1933631778	-2.3853429106
C	3.9926676575	-4.2040891702	-1.7383939770
C	6.4908115808	-2.7859406352	1.7545126196
C	6.8989755380	-3.1442244314	-0.6007933052
C	4.3730261990	4.9603905483	3.4820090414
H	4.9831597752	3.3123555800	2.2662149515
C	2.2697107015	6.0109629010	2.9470451734
C	-3.2824265640	5.1277837839	0.6673234668
C	-2.2800766528	3.0329210091	1.3499655107
C	-2.2816566665	6.0932859311	-2.5462413248
C	-0.1670693704	6.9017381713	-1.6969387929
C	-0.1824727047	2.8817786294	-3.1589853008
C	-2.0997908593	1.8915702891	-2.0594873069
C	7.8337685877	3.3162807132	-1.0923789295
H	7.1192029025	5.3656328231	-0.9750314181
H	8.2371437044	1.2097874988	-1.1578686807
C	2.4488253018	-4.4154124535	2.8649063802
H	3.8077507485	-4.6377073257	1.2180837653
C	1.7090358506	-2.1674563729	3.3574429998
H	2.4801985927	-0.6006071248	2.1025435358
C	2.2893494928	-2.8649000019	-3.4965496893
H	2.5322554128	-1.1447930959	-2.2358329161
C	3.4760822791	-4.8698385393	-2.8527007832
H	4.6553542757	-4.7526163327	-1.0642272269
C	7.7697497349	-3.2725332430	2.0501557198
H	5.8477161307	-2.4617212913	2.5778351345
C	8.1765629276	-3.6321031728	-0.3065659800
H	6.5798679442	-3.1009089970	-1.6456987038
C	3.3921911581	5.9485597100	3.7340867607
H	5.2549356233	4.8974783066	4.1230160284
H	1.4955879464	6.7572339355	3.1438973150
C	-4.2085836061	5.0264841799	1.7123592488
H	-3.3300327198	5.9961235775	0.0060151011
C	-3.1978934847	2.9392774718	2.4019170241
H	-1.5355717483	2.2275951597	1.2186302581
C	-2.4531839220	7.2842013184	-3.2586081599
H	-3.0493398642	5.3179573337	-2.6223161888
C	-0.3378046510	8.0947510102	-2.4107623297
H	0.7398961709	6.7724176646	-1.1024294969
C	-0.1704200667	1.8354342270	-4.0930086040
H	0.5791091752	3.6649656496	-3.2391612001
C	-2.0963737081	0.8522835957	-3.0049195702
H	-2.8905101529	1.9128687511	-1.2899983984
H	8.8424334416	3.6030648107	-1.3960028384
C	1.6884110523	-3.5334958545	3.6418386901
H	2.4422652798	-5.4795151983	3.0881589076
H	1.1188803603	-1.4748418815	3.9542588935
C	2.6167863226	-4.2005642303	-3.7326082839
H	1.6119551384	-2.3374728889	-4.1672479377
H	3.7412739950	-5.9078539586	-3.0392719221
C	8.6134985297	-3.6972213237	1.0202348497
H	8.1059165506	-3.3204553338	3.0837633827

H	8.8303630694	-3.9623295572	-1.1108808113
H	3.5251580949	6.6491321873	4.5608524046
C	-4.1627619956	3.9347756690	2.5838904293
H	-4.9588015417	5.8012623541	1.8492355940
H	-3.1521683590	2.0923450450	3.0839443128
C	-1.4810550950	8.2878855206	-3.1899974246
H	-3.3411622808	7.4293428190	-3.8698777346
H	0.4231783844	8.8704628102	-2.3595352647
C	-1.1124425564	0.8110847227	-4.0049452468
H	0.5903482947	1.8134897760	-4.8707098583
H	-2.9145561940	0.1134465723	-3.0114933926
H	1.0868953819	-3.9126650829	4.4650546496
H	2.2030584913	-4.7223178362	-4.5942314111
H	9.6068175076	-4.0774190367	1.2495786142
H	-4.8732130840	3.8624559812	3.4043560286
H	-1.6141416089	9.2144221529	-3.7447488847
H	-1.0915001598	-0.0182465171	-4.7114905746
